# Alkyl Cyclopropyl Ketones in Catalytic Formal [3 + 2] Cycloadditions: The Role of SmI_2_ Catalyst Stabilization

**DOI:** 10.1021/jacs.4c03073

**Published:** 2024-04-25

**Authors:** Jack I. Mansell, Song Yu, Muze Li, Emma Pye, Chaofan Yin, Frédéric Beltran, James A. Rossi-Ashton, Ciro Romano, Nikolas Kaltsoyannis, David J. Procter

**Affiliations:** Department of Chemistry, University of Manchester, Oxford Road, Manchester M13 9PL, U.K.

## Abstract

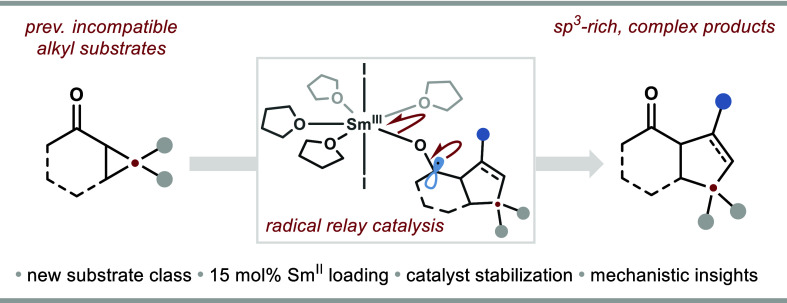

Alkyl cyclopropyl ketones are introduced as versatile substrates for catalytic formal [3 + 2] cycloadditions with alkenes and alkynes and previously unexplored enyne partners, efficiently delivering complex, sp^3^-rich products. The key to effectively engaging this relatively unreactive new substrate class is the use of SmI_2_ as a catalyst in combination with substoichiometric amounts of Sm^0^; the latter likely acting to prevent catalyst deactivation by returning Sm^III^ to the catalytic cycle. In the absence of Sm^0^, background degradation of the SmI_2_ catalyst can outrun product formation. For the most recalcitrant alkyl cyclopropyl ketones, catalysis is “switched-on” using these new robust conditions, and otherwise unattainable products are delivered. Combined experimental and computational studies have been used to identify and probe reactivity trends among alkyl cyclopropyl ketones, including more complex bicyclic alkyl cyclopropyl ketones, which react quickly with various partners to give complex products. In addition to establishing alkyl cyclopropyl ketones as a new substrate class in a burgeoning field of catalysis, our study provides vital mechanistic insight and robust, practical approaches for the nascent field of catalysis with SmI_2_.

## Introduction

**Figure 1:**
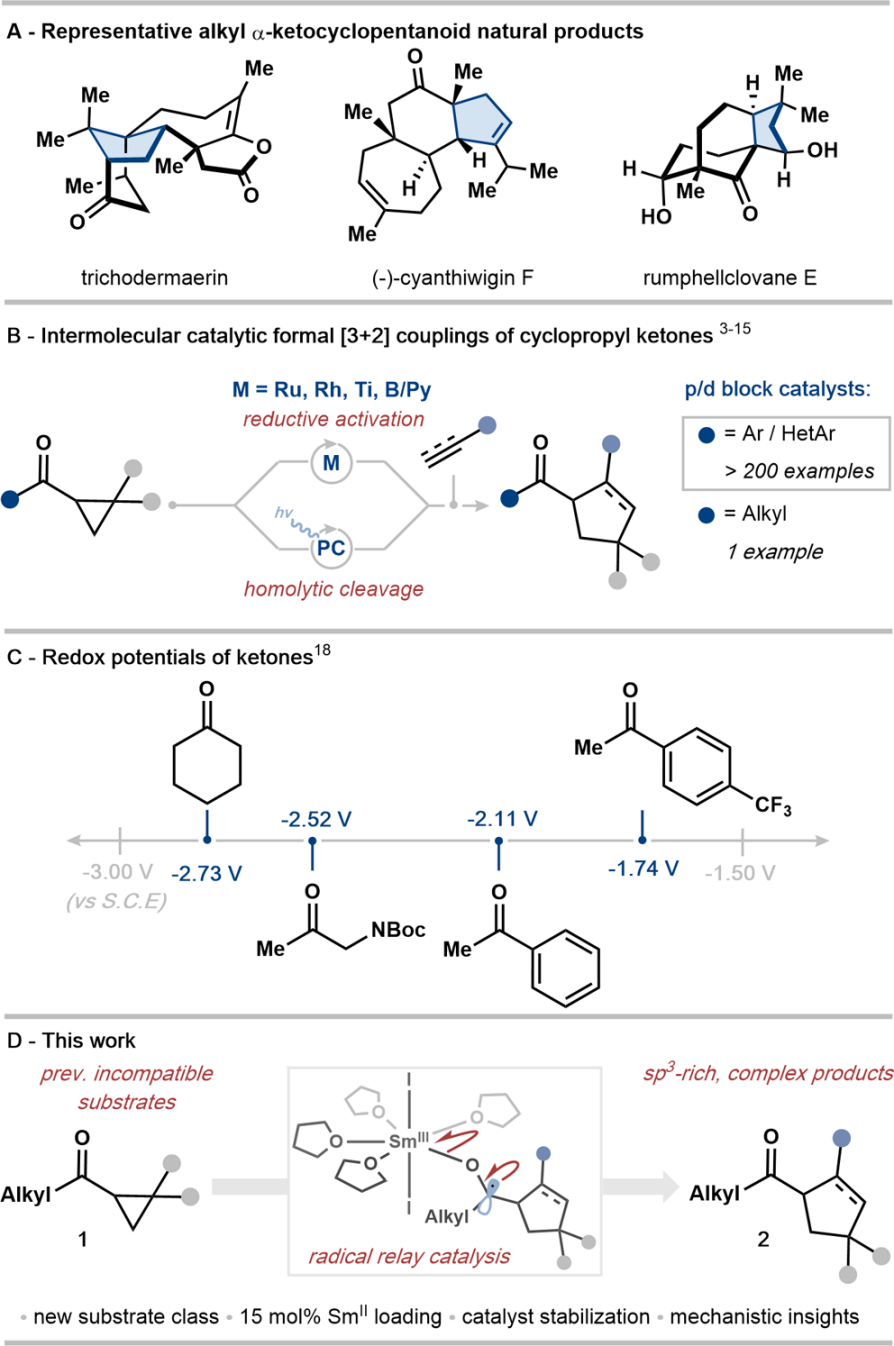
(A) Representative natural products possessing alkyl α-ketocyclopentanoid motifs. (B) Existing intermolecular catalytic couplings of cyclopropyl ketones with alkenes and alkynes are limited to aryl cyclopropyl ketones. (C) Redox potentials of alkyl and aryl ketones. (D) SmI_2_ catalysis facilitates the first formal [3 + 2] cycloadditions using alkyl cyclopropyl ketones.

The ability to construct complex molecular frameworks akin to Nature’s building blocks is a fundamental goal of synthetic organic chemistry. Modular routes to such frameworks—especially those involving catalytic reactions—are particularly sought after, as they enable the efficient construction of highly relevant compound libraries that can be used to unlock the correlation between compound structure and biological activity in, for example, the pharmaceutical and agrochemical industries (Figure 1A).^1,2^ Recent years have seen intense interest in the exploitation of cyclopropyl ketones in formal [3 + 2] cycloaddition reactions with alkene and alkyne partners under radical and photochemical/photocatalytic conditions.^3−17^ Crucially, advancements in this area have been firmly limited to employing *aryl* cyclopropyl ketone starting materials (Figure 1B), leading to products largely incongruent with Nature’s sp^3^-rich building blocks. The reason for this significant limitation and the major challenge associated with the reduction of unactivated aliphatic carbonyl compounds is their low standard redox potential (e.g., cyclohexanone *E*_1/2_ = −2.73 V vs SCE).^18^ In contrast, aryl ketones are easier to reduce by single-electron transfer (SET) (e.g., acetophenone *E*_1/2_ = −2.11 V vs SCE) (Figure 1C).^19^ In this study, we overcome this limitation and introduce *alkyl* cyclopropyl ketones **1** as versatile substrates for catalytic formal [3 + 2] cycloadditions. Pivotal to our approach is the use of SmI_2_ as a catalyst.

Using “stoichiometric reagents” in a catalytic fashion is vital, as sustainability becomes the primary aspect of organic synthesis.^20,21^ In this context, we have recently showcased the catalytic potential of SmI_2_, a reagent used in a superstoichiometric fashion for more than 50 years.^22−24^ The use of SmI_2_ in substoichiometric amounts not only reduces cost and waste but also has the potential to unveil reactivity paradigms that are hidden when excess SmI_2_ is present. We have reported the first processes that operate solely with SmI_2_ as a catalyst and do not require an excess of metal coreductants and additives (e.g., Mg and TMSCl) that compromises the atom economy gained by the move to catalysis.^25−40^ Our mechanism-based approach to catalysis with SmI_2_ hinges on the exploitation of radical relays; an electron is donated from and subsequently returned to the samarium center amid a series of bond-breaking and forming steps.

Our studies in SmI_2_ catalysis have centered on formal [3 + 2] cycloaddition reactions, as exemplified in intra- and intermolecular couplings, and exploited in the synthesis of bioisosteres.^25−27^ However, to date, the SmI_2_-catalyzed [3 + 2] cycloaddition reactions of cyclopropyl ketones are limited to aromatic ketone substrates—in line with other catalytic approaches to the transformation, as previously discussed (Figure 1B). This limitation with SmI_2_ is at first surprising, given that the reagent is known to reduce dialkyl ketones, albeit when used in superstoichiometric amounts and in combination with activating additives and proton sources that increase the reactivity of the reagent.^41^ Cognizant that such additives could lead to the reduction of radical intermediates, thus undermining the radical-relay approach that underpins SmI_2_ catalysis, we set out to improve our understanding of working with SmI_2_ as a catalyst, with the aim of developing catalytic [3 + 2] cycloaddition reactions that embrace alkyl cyclopropyl ketone substrates.^25,26^

Here, we introduce alkyl cyclopropyl ketones, both simple and more complex, as highly versatile substrates in catalytic [3 + 2] cycloaddition reactions with alkene and alkynes and thus address an unmet challenge (Figure 1D). The SmI_2_-catalyzed protocol not only expands the scope of cyclopropyl ketone feedstocks that can be engaged in formal [3 + 2] cycloadditions, thus allowing access to sp^3^-rich architectures, but also has led to the development of robust SmI_2_ reaction conditions that allow catalyst stabilization. For the most recalcitrant alkyl cyclopropyl ketone substrates, catalysis is “switched-on” using these new robust conditions. Combined experimental and computational studies have identified and probed reactivity trends among aliphatic cyclopropyl ketones, including more complex bicyclic cyclopropyl ketones, which react quickly with various partners to give complex products.

## Results and Discussion

### Reaction Development

Applying our previously reported catalytic SmI_2_ conditions in the coupling of hindered aliphatic cyclopropyl ketone **1a** with phenylacetylene gave cyclopentene **2a** in low yield (Table 1, entry 1).^26^ We rationalized that the low yield arises from a slow initial SET to the ketone carbonyl resulting from the low redox potential of the alkyl cyclopropyl ketone. In this slow-reacting system, we propose that the rate of SmI_2_ catalyst deactivation becomes significant compared to the rate of substrate reduction. This hypothesis was corroborated by the observation of samarium salts precipitating out of the solution during the reaction. Although SmI_2_ solutions can be successfully used over several days when an excess of the reagent is employed, we reasoned that the use of catalytic amounts of SmI_2_ would be more sensitive to the speciation of the SmI_2_ solutions and thus be dependent on reagent age. Consequently, fresh SmI_2_ solution was prepared and used immediately in catalysis to give a significantly improved 65% yield of **2a** (Table 1, entry 2), supporting the notion that improving the stability of SmI_2_ (increasing catalyst half-life) is likely the key to extending SmI_2_ catalysis to less-reactive substrates, including alkyl cyclopropyl ketones. To stabilize the SmI_2_ catalyst to oxidative degradation to SmI_3_, perhaps due to trace oxygen, an equal substoichiometric loading of Sm^0^ was added. The use of SmI_2_/Sm^0^ (15 mol %) had a marked effect, increasing the yield of the product to 77%, even when a 3-day-old batch of SmI_2_ was employed (Table 1, entries 3 and 4).^42−44^ Thus, the SmI_2_/Sm^0^ catalyst system offers more consistent performance in the catalytic formal [3 + 2] cycloaddition using less-reactive substrates, such as alkyl cyclopropyl ketones. Of note, a significant amount of Sm^0^ remains after reaction workup, suggesting that a lower Sm^0^ loading could be employed, although for the practicality of handling, a loading of 15 mol % was used. Control experiments confirmed SmI_2_ to be the active catalyst; the use of Sm^0^ or SmI_3_ resulted in no formation of **2a**, and starting materials were recovered (Table 1, entries 5–6). Product **2a** was also not obtained when Sm^III^/Sm^0^ (30 mol %) was employed, suggesting that comproportionation of SmI_3_ and Sm^0^ to form active SmI_2_ catalyst does not occur efficiently under the reaction conditions, perhaps due to the insolubility of both species when present in substantial amounts (Table 1, entry 7).

**Table 1:**
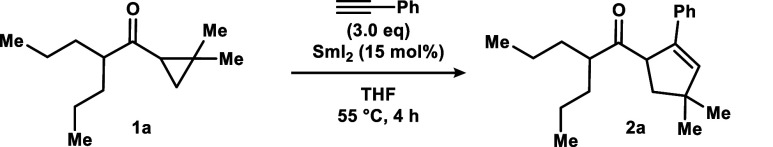
Optimization of the SmI_2_-Catalyzed Formal [3 + 2] Cycloaddition of Alkyl Cyclopropyl Ketone **1a**^a^

entry	deviation from above	^1^H NMR yield (%)^b^
1	none	11
2^c^	freshly prepared Sml_2_ (15 mol %)	65
3^d^,^e^	Sml_2_ (15 mol %) + Sm^0^ (15 mol %)	77
4^c^	Sml_2_ (30 mol %)	65
5	Sm^0^ instead of Sml_2_	n.d.
6^f^	Sml_3_ (15 mol %) instead of Sml_2_	n.d.
7^d^,^f^	Sml_3_ (15 mol %) + Sm^0^ (15 mol %)	n.d.

a Reaction conditions: **1a** (0.10 mmol), phenylacetylene (0.30 mmol), SmI_2_ (0.015 mmol) in THF (0.65 mL) under nitrogen at 55 °C for 4 h.

b Yields were determined by ^1^H qNMR with CH_2_Br_2_ as internal standard (0.05 mmol).

c SmI_2_ was prepared, titrated (see the Supporting Information), and used immediately.

d Sm metal (0.015 mmol) was added prior to SmI_2_.

e Using SmI_2_ that was stirred under nitrogen for 72 h prior to use.

f SmI_3_ (0.015 mmol, 15 mol %) was added prior to the solvent. n.d., not detected.

**Figure 2:**
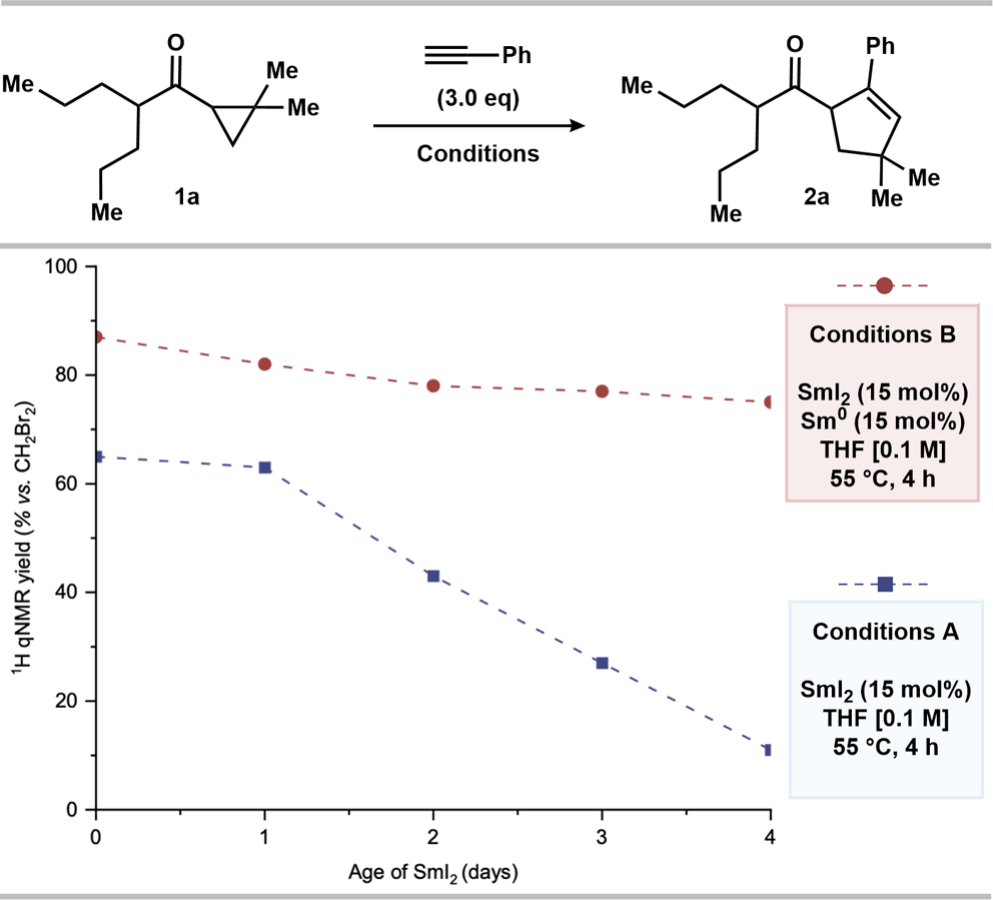
Catalyst aging study. Reaction conditions: **1a** (0.10 mmol, 1.0 equiv), phenylacetylene (0.30 mmol), and SmI_2_ (15 mol %) in THF (0.65 mL) under nitrogen at 55 °C for 4 h. Yields were determined by ^1^H qNMR with CH_2_Br_2_ as internal standard (0.05 mmol). The stock SmI_2_ solution in THF (0.1 M) was stirred vigorously under nitrogen.

Focusing on the discrepancies in yield obtained using “stored” and freshly prepared SmI_2_ solutions (Table 1, entries 1 and 2) and aiming to underline the beneficial presence of substoichiometric Sm^0^, we conducted a catalyst aging study; parallel reactions were performed using conditions A (no Sm^0^) and B (15 mol % Sm^0^) while varying the age of the SmI_2_ solution employed (Figure 2). Notably, with freshly prepared SmI_2_, the product yield was consistent with previous results (see Table 1, entries 2 and 3). However, over 4 days, using the same stock solution of SmI_2_, the yield of **2a** under conditions A steadily decreased. In these cases, the reaction mixtures underwent a gradual color change from deep blue to yellow, associated with the conversion of Sm^II^ to Sm^III^—typically within the first 2 h. In stark contrast, using conditions B, the yield of **2a** remained constant (within the error associated with quantitative ^1^H NMR [^1^H qNMR]) as the stock SmI_2_ solution aged. A color change was not observed in the reactions using conditions B, and visible metallic Sm^0^ remained in the reaction vessel after the workup. We propose that the addition of substoichiometric Sm^0^ aids the stabilization of SmI_2_ by recovering off-cycle Sm^III^ species that form by catalyst decomposition.^45−50^

### Substrate Scope

**Scheme 1:**
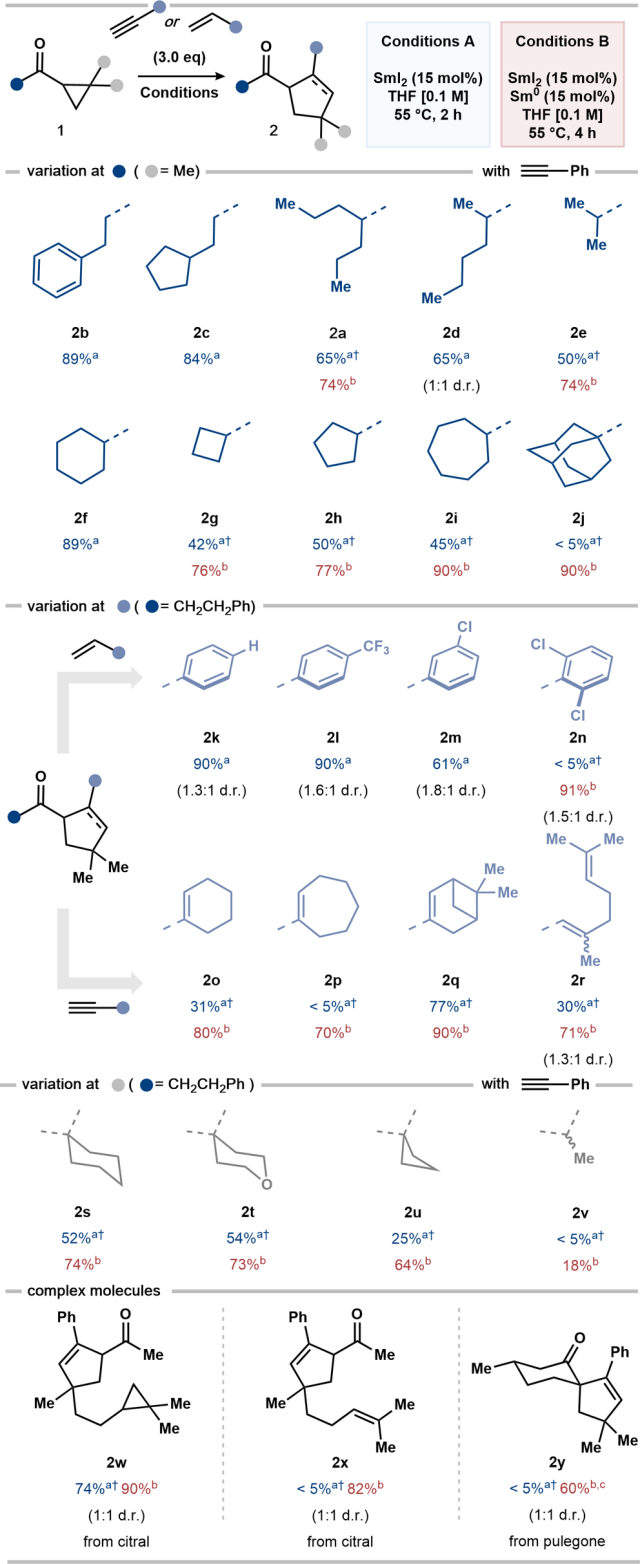
Scope of the SmI_2_-Catalyzed Coupling of Alkyl Cyclopropyl Ketones **1** Reaction conditions A were used on a 0.10 mmol scale, and the isolated yield is reported unless otherwise stated. Reaction conditions B were used on a 0.20 mmol scale, and the isolated yield is reported unless otherwise stated. Sm^0^ (30 mol %) was used. ^†^ Yields were determined by ^1^H qNMR with CH_2_Br_2_ as internal standard (0.05 mmol).

With the optimized conditions in hand, we first examined the scope of SmI_2_-catalyzed couplings with regard to the aliphatic substituent on the carbonyl group in alkyl cyclopropyl ketones (Scheme 1). Cyclopropyl ketones bearing primary alkyl groups gave products in good yield under conditions A (**2b**, **2c**). However, conditions B were typically required to give acceptable yields when working with less-reactive secondary alkyl cyclopropyl ketone substrates (**2a**, **2d–i**). For example, cycloheptyl-containing product **2i** was obtained in 45% yield using conditions A and 90% using conditions B. Strikingly, using a tertiary alkyl cyclopropyl ketone substrate, conditions B were essential to “switch-on” catalysis and **2j** was obtained in 90% yield; only traces of **2j** were obtained using conditions A.

To confirm that the newly developed, SmI_2_-catalyzed coupling of alkyl cyclopropyl ketones extended to other unsaturated partners, we assessed the use of both validated, styrene, and novel, enyne, 2π components. Coupling with a range of styrenes gave ketocyclopentane products (**2k**-**2m**) in 61–90% yield. Again, in the more challenging coupling of 2,6-dichlorostyrene to give **2n**, the use of conditions B proved to be essential. Alkyl cyclopropyl ketones also underwent efficient coupling to previously unexplored enyne partners, and keto-cyclopentene products **2o–r** were obtained in 70–90% yield using conditions B. In the formation of **2p**, the use of Sm^0^ to stabilize the SmI_2_ catalyst improved the yield from 5 to 70%. Coupling to give **2r** highlights the ability to form highly functionalized polyene products with complete selectivity for catalysis at the terminal alkyne unit. Simple alkyl alkyne partners do not undergo productive couplings with alkyl cyclopropyl ketones under either set of conditions (see the Supporting Information.)

Variation of substitution on the cyclopropane ring was next explored. Spirocyclic alkyl cyclopropyl ketones underwent smooth coupling to give **2s–u** in 64–74% yield using conditions B for SmI_2_ catalysis; the use of conditions A led to lower yields that were more sensitive to the nature of the substrate, presumably due to varying rates of reaction of the substrates with SmI_2_. Monoalkyl substitution on the cyclopropane is currently not well-tolerated; conditions B gave **2v** in low yield. Finally, the use of more complex alkyl cyclopropyl ketones, prepared from monoterpenoids citral and pulegone, with differing or additional alkyl substitution on the cyclopropane was investigated. Cyclopropane-containing **2w** was formed efficiently with complete selectivity for the opening of the cyclopropane unit adjacent to the ketone carbonyl. Formation of **2x** again showcased the compatibility of SmI_2_ catalysis with unsaturation elsewhere in the alkyne partner. Finally, a tetrasubstituted alkyl cyclopropyl ketone substrate underwent challenging catalytic coupling to give spirocycle **2y**, bearing a quaternary stereocenter, in 60% yield. Again, in all three cases, the use of conditions B proved to be important, with our strategy for catalyst stabilization being particularly effective in the formation of **2x** and **2y**; only traces of product were observed in the absence of Sm^0^.

**Scheme 2:**
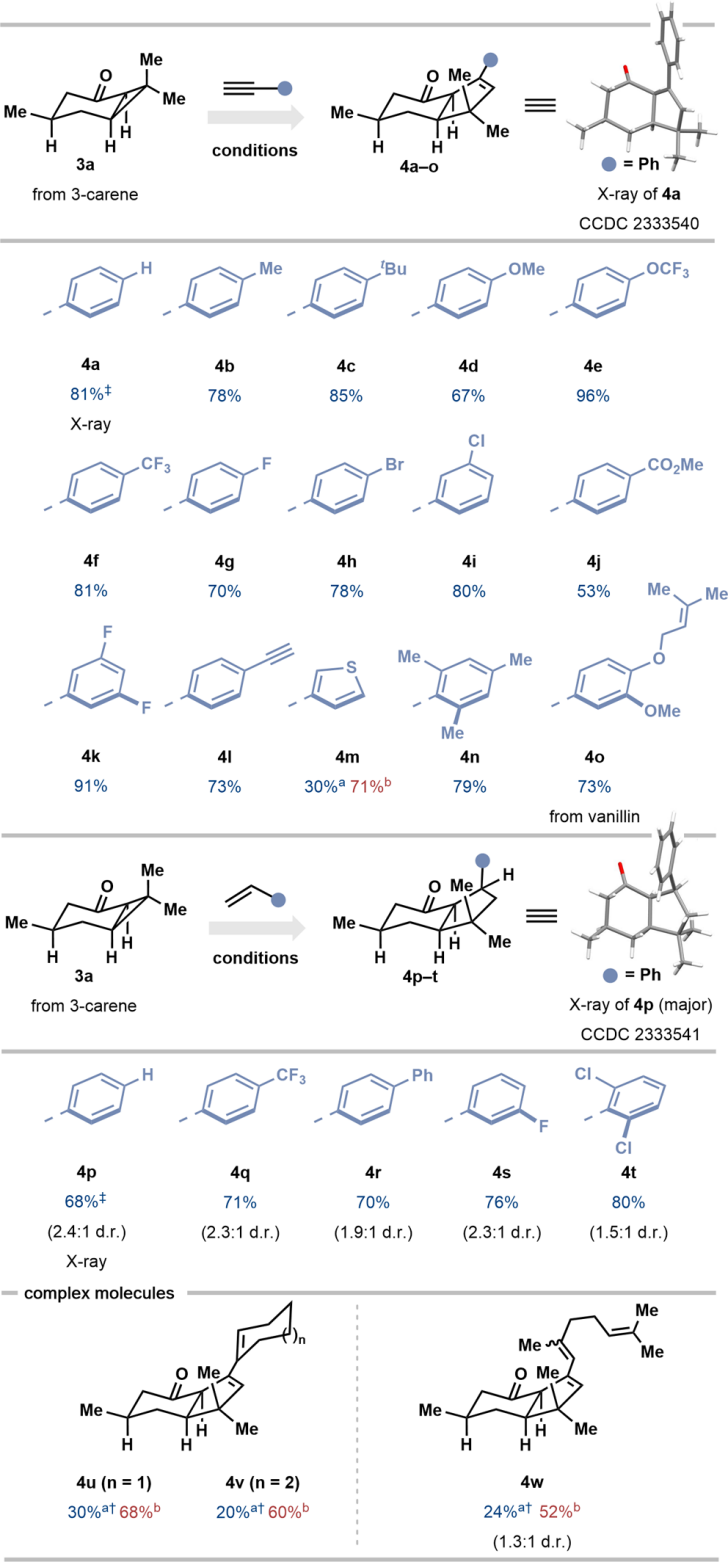
Scope of the SmI_2_-Catalyzed Coupling of Bicyclic Alkyl Cyclopropyl Ketone **3a** Reaction conditions A were used on a 0.10 mmol scale. Reaction conditions B were used on a 0.20 mmol scale. ^†^ Yields were determined by ^1^H qNMR with CH_2_Br_2_ as internal standard (0.05 mmol). ^‡^ Reaction run on a 1.0 mmol scale.

We next sought to exploit our new-found ability to work with alkyl cyclopropyl ketones by studying the preparation of fused bicyclic architectures akin to those found in Nature using bicyclic alkyl cyclopropyl ketone substrates (Scheme 2). Complex alkyl cyclopropyl ketone **3a**, readily prepared from the monoterpene 3-carene,^51^ was subjected to conditions A with phenylacetylene as a partner, forming ketone **4a** in 81% yield and as a single diastereoisomer. The structure of **4a** was confirmed by X-ray crystallography. A range of arylacetylenes bearing electron-donating alkyl (**4b**, **4c, 4n**) and alkoxy (**4d**, **4e, 4o**) substituents, or electron-withdrawing trifluoromethyl (**4f**), fluoro (**4g**, **4k**), bromo (**4h**), chloro (**4i**), and carbomethoxy (**4j**) groups, proved compatible with catalysis, affording the corresponding products in moderate to high yield. Interestingly, bicyclic alkyl cyclopropyl ketone **3a** proved sufficiently reactive that the use of conditions B was necessary only to obtain a satisfactory yield of thiophene-containing product **4m**.

Bicyclic alkyl cyclopropyl ketone **3a** also underwent smooth coupling with styrenes to form products **4p–4t** as mixtures of two diastereoisomers with moderate diastereocontrol. In the coupling with styrene to form **4p**, the relative stereochemistry of the major diastereoisomer was confirmed by X-ray crystallography. Good functional group compatibility was again observed with the trifluoromethyl (**4q**), fluoro (**4s**), and chloro (**4t**) substituents tolerated. Enynes were also competent coupling partners with bicyclic alkyl cyclopropyl ketone **3a**, delivering complex products **4u**-**w** in 52–68%; for these more challenging couplings, the use of conditions B was crucial in giving satisfactory yields (Scheme 2).

**Scheme 3:**
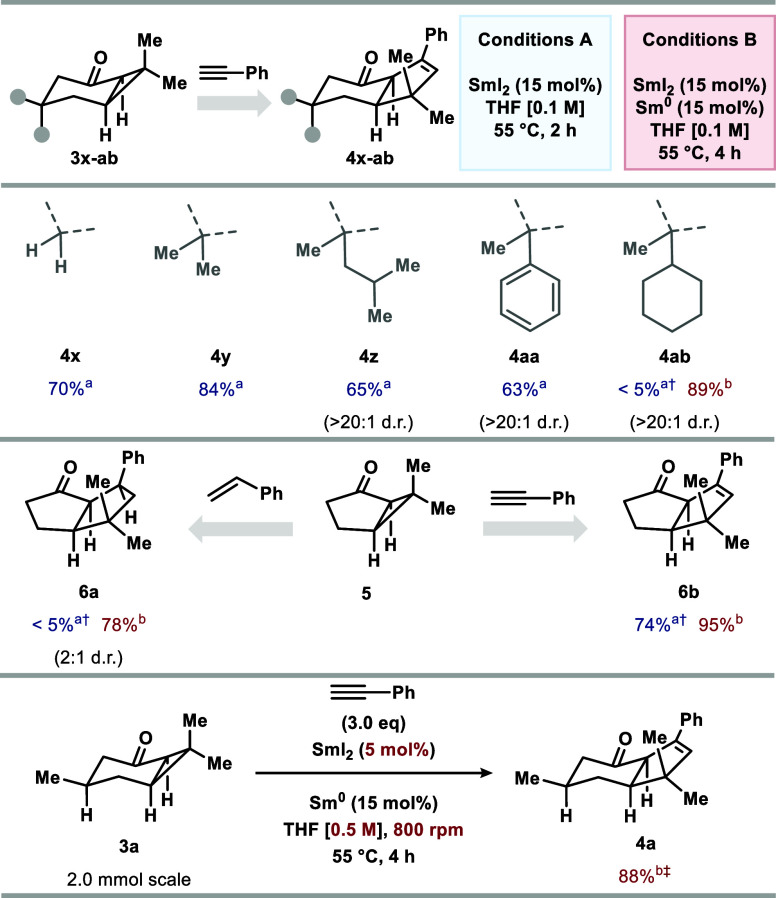
Variation of Fused Bicyclic Alkyl Cyclopropyl Ketones **3** Reaction conditions A were used on a 0.10 mmol scale Reaction conditions B were used on a 0.20 mmol scale. ^†^ Yields were determined by ^1^H qNMR with CH_2_Br_2_ as internal standard (0.05 mmol). ^‡^ Modified conditions B: SmI_2_ (5 mol %) and THF (0.5 M) with 800 rpm stirring on a 2.0 mmol scale.

The bicyclic alkyl cyclopropyl ketone substrates could also be varied. Tuning the substitution at the 5-position of substrates **3** allowed access to products **4x-ab** in good yield upon coupling with phenylacetylene (Scheme 3). The beneficial impact on catalysis of adding substoichiometric Sm^0^ was particularly striking in the preparation of **4ab**, with only traces of product obtained using conditions A. 5-Membered bicyclic alkyl cyclopropyl ketone **5** also underwent efficient coupling with both phenylacetylene and styrene, giving **6a** and **6b**, respectively, in good yield. The use of conditions B was particularly crucial for efficient SmI_2_ catalysis in the formation of **6a**.

The robustness of the protocol was illustrated by scale-up experiments that, for example, enabled the use of significantly lower SmI_2_ loading (5 mol %) under slightly modified conditions B for the formation of **4a** in 88% yield (2.0 mmol scale, 452 mg of **4a** prepared) (Scheme 3). Of note, increasing the concentration and stirring speed proved essential for full conversion of **3a**; presumably, this helps ensure the return of off-cycle Sm^III^ species to the catalytic cycle.

### Mechanistic Study

**Figure 3:**
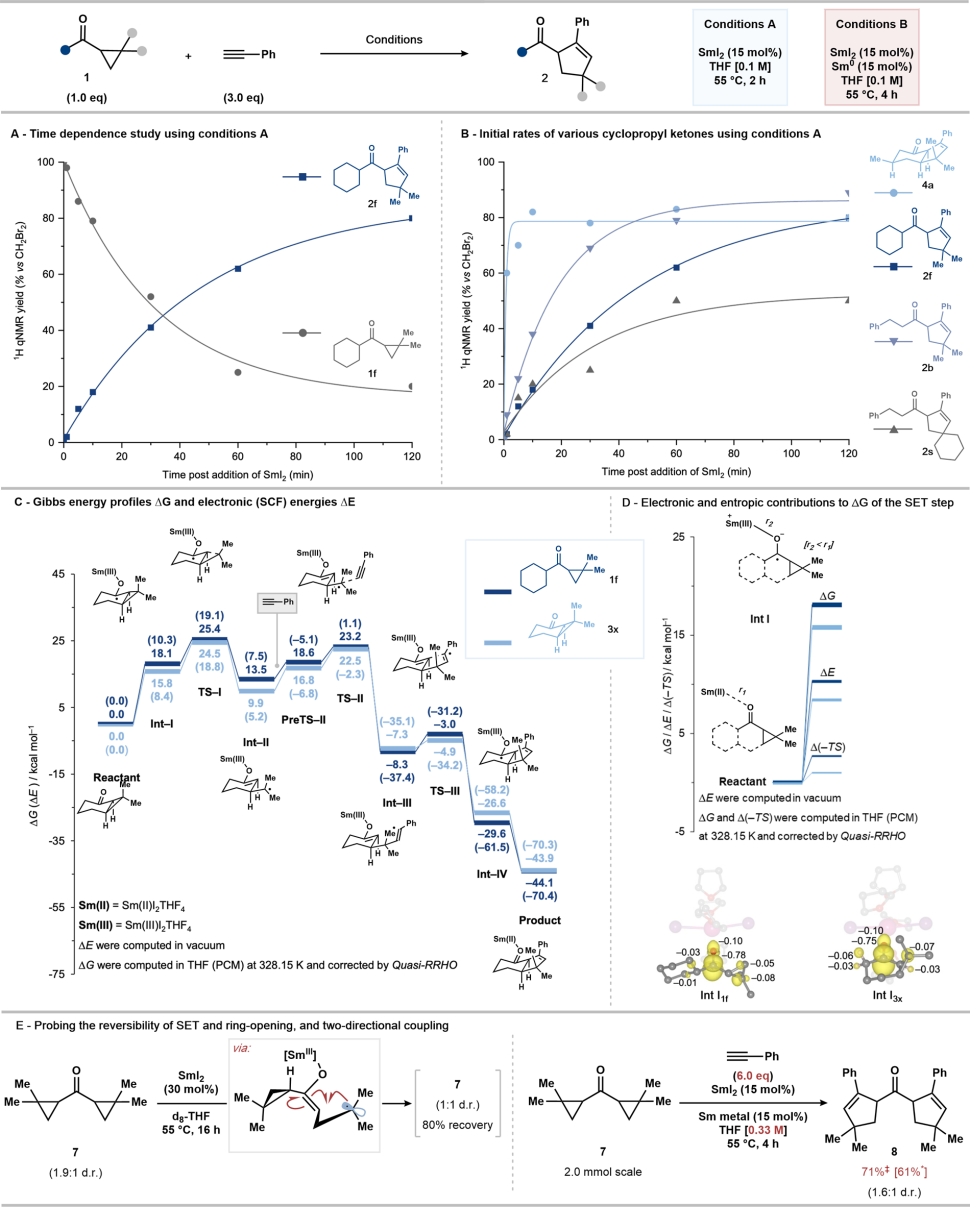
Mechanistic studies. (A) Reaction progress monitoring using conditions A with substrate **1f**; gray circles and squares correspond to the conversion of the starting material and the product formation, respectively, determined by ^1^H qNMR using CH_2_Br_2_ as internal standard; all lines represent nonlinear best-fit curves. (B) Reaction progress monitoring using conditions A with substrates **3a** (circle), **1f** (square), **1s** (right-side up triangle), and **1b** (upside down triangle); all lines represent nonlinear best-fit curves. (C) Gibbs energy profiles Δ*G* and, in parentheses, electronic (SCF) energies Δ*E* of the SmI_2_-catalyzed coupling reactions of **1f** and **3x** with phenylacetylene (See the Supporting Information for computational details). (D) Electronic and entropic contributions to the Gibbs energy changes of the SET step and spin density distributions (isovalue = 0.005 e^–^/au^3^) with numerical values (in e^–^) of key atoms of **Int-I**_**1f**_ and **Int-I**_**3x**_. (E) Epimerization of diastereoisomerically enriched *bis*-cyclopropyl ketone **7** serves as a probe for the reversibility of ketone reduction and cyclopropane ring-opening. Reaction on a 0.10 mmol scale with mass balance determined by ^1^H qNMR with CH_2_Br_2_ as internal standard (0.10 mmol). Larger scale two-directional coupling using *bis*-cyclopropyl ketone **7** and recycling of the Sm metal. ^‡^Modified conditions B: phenylacetylene (6.0 equiv) and THF (0.33 M) on a 2.0 mmol scale. *Sm metal was recycled and used again on a 2.0 mmol scale.

Intrigued by the fact that efficient catalytic conversion of some alkyl cyclopropyl ketone substrates required the presence of a substoichiometric amount of Sm^0^ in addition to SmI_2_ (conditions B) while other substrates did not (conditions A), reaction progress was monitored by ^1^H NMR in a series of processes involving representative substrates. For example, monitoring the conversion of cyclopropyl ketone **1f** to cyclopentene **2f** under conditions A, using freshly prepared and titrated SmI_2_, showed an exponential decay of the starting material with concomitant formation of the product (Figure 3A).

Similar reaction monitoring using a variety of alkyl cyclopropyl ketone substrates clearly illustrated that primary alkyl cyclopropyl ketones (such as **1b**) typically react more quickly than analogous secondary alkyl cyclopropyl ketones (such as **1f**) (Figure 3B). This is presumably due to steric hindrance impeding coordination of the SmI_2_ catalyst to the ketone carbonyl prior to inner-sphere SET.^52^ One exception is primary spirocyclic alkyl cyclopropyl ketone **1s**, which reacts slowly, presumably due to steric hindrance associated with the tertiary radical intermediate hindering coupling. In stark contrast, the reaction of bicyclic alkyl cyclopropyl ketone **3a** was found to proceed to completion within 5 min. These results fit well with the hypothesis that SmI_2_ catalyst stabilization, using Sm^0^ (conditions B), becomes crucial with less-reactive substrates; the slower-reacting spirocyclic alkyl cyclopropyl ketone **1s** required the use of conditions B to achieve satisfactory yields of the product while the faster-reacting bicyclic alkyl cyclopropyl ketone **3a** did not.

Computational studies have been employed to probe the origin of the high reactivity of bicyclic alkyl cyclopropyl ketones **3**. In so doing, we have employed a modification of our previous computational approach,^27^ now applying the Douglas–Kroll–Hess second-order scalar relativistic Hamiltonian coupled with all-electron basis sets. This revised methodology is based on our further investigation of a previously studied catalytic cycle,^26^ as set out in the Supporting Information, Section 3.1, exploring the differences between reaction energy profiles obtained at the ECP and all-electron (both scalar and spin–orbit) levels: all-electron SET barriers better match experimental observations. The Gibbs energy profiles (Figure 3C) reveal that the reaction involving bicyclic alkyl cyclopropyl ketone **3x** has a slightly lower overall reaction barrier at **TS-I** (24.5 kcal mol^–1^) compared with that of cyclohexyl cyclopropyl ketone **1f** (25.4 kcal mol^–1^). This aligns well with experimental findings showing that bicyclic alkyl cyclopropyl ketones exhibit more favorable kinetics in the SmI_2_-catalyzed reactions (Figure 3B). Importantly, the reduced barrier associated with bicyclic compound **3x** does not originate from more facile ring fragmentation (**Int-I** to **TS-I**) but is attributable to a more favorable initial SET from Sm(II) to the substrate (**Reactant** to **Int I**). This latter difference can be ascribed to smaller electronic and entropic terms contributing to a reduced Gibbs energy change during SET to **3x** (Figure 3C). The lower electronic energy (Δ*E*) observed for **3x** may derive from two factors: (i) **3x** has lower electron density at the carbonyl carbon and a more polarized carbonyl group, which manifests as a slightly lower LUMO energy (see the Supporting Information) and thus greater reactivity toward reductive SET; (ii) **Int-I** of **3x** benefits from a small hyperconjugation effect that disperses the spin density of the ketyl radical over the adjacent C–C bond of the cyclopropane ring, consequently stabilizing this intermediate (Figure 3D). Furthermore, the SET step is concomitant with a decrease in the length of the Sm–O bond, owing to an increased electrostatic attraction between the more positive trivalent metal and substrate (the Sm–O distance varies from 2.50 to 2.10 Å for **1f** and from 2.57 to 2.09 Å for **3x**). This results in a more compact intermediate complex with a decrease in the entropy and an increase in the −TS term. Bicyclic compound **3x** exhibits a less-pronounced increase in Δ(−TS), indicative of a smaller decrease in the entropy. This is likely because the compact and rigid skeleton of **3x** undergoes minimal geometric changes during the shortening of the Sm–O distance associated with the SET step.

An additional mechanistic experiment using *bis*-cyclopropyl ketone **7** was conducted – in the absence of a coupling partner – to probe the reversibility of SET to the ketone and cyclopropyl ring-opening; **7** (dr 1.9:1) was found to undergo epimerization—presumably by ketone SET reduction, ring-opening, ring-closure, and BET to Sm(III)—to give **7** (dr 1:1) after exposure to catalytic SmI_2_ (30 mol % SmI_2_ in *d*_8_-THF, 55 °C, 16 h, 80% recovery by ^1^H qNMR) (Figure 3E). Finally, the same substrate **7** was used in a two-directional coupling on a 2.0 mmol scale to give *bis*-cyclopentene **8** in 71% yield. The Sm metal from the coupling was recovered and reused in conjunction with 15 mol % SmI_2_ to convert **7** into **8** in 61% yield, also on a 2.0 mmol scale.

## Conclusions

We have introduced alkyl cyclopropyl ketones as versatile substrates for catalytic formal [3 + 2] cycloadditions with alkenes, alkynes, and new enyne partners, efficiently delivering complex, sp^3^-rich products. The key to effectively engaging this relatively unreactive new substrate class is the use of SmI_2_ as a catalyst in combination with Sm^0^, the latter likely acting to prevent catalyst deactivation by returning Sm^III^ to the catalytic cycle. In the absence of Sm^0^, background degradation of the SmI_2_ catalyst can outrun product formation. In several examples involving the most recalcitrant alkyl cyclopropyl ketones, the use of these new robust conditions for SmI_2_ catalysis resulted in a dramatic “switching-on” of the reactivity and delivery of otherwise unattainable products. Combined experimental and computational studies have been used to probe the reactivity of different alkyl cyclopropyl ketones, including more complex bicyclic cyclopropyl ketones, which react quickly with various partners to give complex products. Crucially, in addition to establishing alkyl cyclopropyl ketones as a new substrate class in a burgeoning field of catalysis, our study provides valuable mechanistic insight and robust, practical approaches for the nascent field of catalysis with SmI_2_.

## Supporting Information Available

Additional experiments, experimental procedures, characterization data, X-ray data, and NMR spectra for all new compounds (PDF)
