# Supplementary material for: Alkyl Cyclopropyl Ketones in Catalytic Formal [3 + 2] Cycloadditions: The Role of SmI2 Catalyst Stabilization
Source: J Am Chem Soc. 2024 Apr 25;146(18):12799–807. doi: 10.1021/jacs.4c03073 (PMC11082888; doi:10.1021/jacs.4c03073)

Alkyl Cyclopropyl Ketones in Catalytic Formal [3+2] Cycloadditions: The Role of SmI_2 Catalyst Stabilization

Jack I. Mansell[1], Song Yu[1], Muze Li[1], Emma Pye[1], Chaofan Yin[1], Frédéric Beltran[1], James A. Rossi-Ashton[1], Ciro Romano[1], Nikolas Kaltsoyannis[1], and David J. Procter[1*]

1 – Department of Chemistry, The University of Manchester, Oxford Road, Manchester, M13 9PL (UK).

*email: david.j.procter@manchester.ac.uk

Supporting information

1. General information Page 3

1.1 Abbreviations Page 5

1.2 Preparation of reagents Page 7

1.3 Synthesis of radical traps Page 12

1.4. Starting material preparation Page 18

1.4.1 General Procedures Page 18

1.4.2. Synthesis and characterisation data for starting materials Page 21

2. SmI_2-catalyzed cross-coupling of alkyl cyclopropyl ketones and alkynes/alkenes Page 41

2.1. General Procedures Page 41

2.2. Characterisation data for the products of SmI_2-catalysis Page 42

2.3 Scale-up experiments Page 80

2.3.1 Scale-up synthesis of 1,1,6-trimethyl-3-phenyloctahydro-4*H*-inden-4-one (**4a**) Page 80

2.3.2 Scale-up synthesis of bis(4,4-dimethyl-2-phenylcyclopent-2-en-1-yl)methanone (**8**) Page 80

2.4.1 Summary of unsuccessful reactions Page 82

2.4.2 Miscellaneous Information Page 82

2.5 Time dependence study using conditions A and ketones **1b**, **1f**, **1s**, and **3a** Page 83

2.5.1 Procedure for monitoring SmI_2-catalyzed intermolecular coupling reactions Page 83

2.5.2 Time-dependence study using conditions A and ketone **1f** Page 83

2.5.3 Time dependence study using conditions A and ketones **1b**, **1f**, **1s**, and **3a** Page 85

2.6 SmI_2-catayzed epimerization of *bis*-cyclopropylketone **7** Page 87

3. Computational studies Page 89

3.1 Re-evaluation of computational methodology Page 89

3.2 Computational details Page 91

3.3 Coordinates and energies Page 92

3.4 Additional computational figures Page 118

4. Data for X-ray Structures Page 119

4.1 **4a** CCDC 2333540 Page 119

4.2 **4p** CCDC 2333541 Page 120

5. References Page 121

6. ^{1}H, $^{13}C\{H\}$, ^{19}F, and ^{31}P NMR Spectra Page 123

1. General information

All reactions were carried out in oven-dried glassware, cleaned using base ($KOH_{(aq)}$, ^{i}PrOH) and acid ($HCl_{(aq)}$) baths. All reactions were considered air/moisture sensitive and performed under a dry nitrogen atmosphere, unless otherwise noted. Reactions were magnetically stirred. All reported reaction temperatures correspond to external bath temperatures. Room temperature was approximately 23°C. Yields refer to chromatographically and spectroscopically (^{1}H and ^{13}C NMR) homogeneous materials, unless otherwise stated. Reagents were purchased at the highest commercial quality and used without further purification, unless otherwise stated. Diiodoethane was dissolved in Et_2O and washed with saturated aqueous sodium thiosulfate, then dried under vacuum (rt, 24 h, dark) before use. All organolithium reagents were titrated against diphenylacetic acid (recrystalized from PhMe) in THF prior to use. 4-Methylmorpholine *N*-oxide (NMO) was recrystalized from acetone prior to use. 2,2,6,6-Tetramethylpiperidine (TMP) was distilled over CaH_2 prior to use and stored under an inert atmosphere. Triethylamine (Et_3N) was distilled over CaH_2 prior to use in titrations of SmI_2 and was stored under an inert atmosphere. Water (H_2O) was degassed prior to use in titrations of SmI_2 and was stored under an inert atmosphere. Pyridine was distilled over CaH_2 prior to use and stored under an inert atmosphere. 3-Ethynylthiophene was purified by column chromatography in petroleum ether prior to use. (+)-Pulegone was purified by column chromatography using petroleum ether/Et_2O eluent prior to use.

^{1}H, ^{13}C, ^{19}F and ^{31}P NMR spectra were recorded on Bruker Avance III 400 MHz and 500 MHz instruments fitted with 5 mm Prodigy cryoprobes except for spectra obtained for the epimerisation study involving biscyclopropyl ketone **7**, which were recorded on Bruker Avance Neo 700 MHz. All NMR spectra were processed using Mestrenova© NMR software. Chemical shifts are reported in parts per million (ppm). ^{1}H and ^{13}C, NMR spectra were referenced to the residual solvent peak ($CHCl_3$: ^{1}H = 7.26 ppm, ^{13}C = 77.00 ppm, C_6H_6: ^{1}H = 7.16 ppm, ^{13}C = 128.06 ppm, d_8-THF: ^{1}H = 1.73 ppm and 3.58 ppm). The following abbreviations were used to explain the multiplicities of signals: s = singlet, d = doublet, t = triplet, q = quartet, p = pentet, sx = sextet, h = heptet, m = multiplet. Coupling constants, *J*, are reported in Hertz and are rounded to the nearest 0.1 Hz. Integration of peaks is provided with the assignments indicated where appropriate. ^{1}H NMR and ^{13}C NMR spectra were assigned with the aid of COSY, HSQC, HMBC, DEPT-135 and nOe NMR experiments and stereochemistry assigned with the aid of X-ray crystallography. Stereochemical assignments for structurally similar products were made by

analogy. Infrared (IR) spectra were recorded on a FTIR spectrometer and mass spectra were obtained using positive or negative electrospray ionisation (ESI) and atmospheric pressure chemical ionization (APCI) techniques. High-resolution mass data are reported in the form of m/z (intensity relative to the base peak = 100).

All reactions were monitored by thin-layer chromatography (TLC) carried out on 0.20 mm precoated POLYGRAM SIL G/U254 plates using UV light as a visualising agent, and potassium permanganate ($KMnO_4$) and cerium(IV) sulfate (CAS), cerium ammonium molybdate (CAM), and heat as developing agents. Substrates were purified using Supleco silica gel (60 Å, technical grade, 400 mesh, particle size 0.040-0.063 mm) for column chromatography. Preparative thin-layer chromatography separations were carried out on 0.50 mm E. Merck silica gel plates (60F-254).

All solvents for air- and moisture- sensitive techniques were purchased at the highest commercial grade with ACROS seals and used as received or after distillation from sodium/benzophenone under nitrogen (ethereal solvents). Solvents for filtration, transfers, chromatography, and recrystallisation were acetone (Supleco), dichloromethane (CH_2Cl_2) (Sigma, amylene stabilised, HPLC grade), diethyl ether (Et_2O) (Sigma, BHT stabilised ACS grade), EtOH (Sigma, HPLC grade), ethyl acetate (EtOAc) (Sigma, ACS grade), hexane (Sigma, HPLC grade), methanol (MeOH) (Sigma, ACS grade), pentane (ACS grade), petroleum ether 40-60 °C (Fisher, Analytical grade).

1.1 Abbreviations

**Ac**	acetyl
**APCI**	atmospheric pressure chemical ionization
**bpy**	2,2′-bipyridine
**CAM**	cerium ammonium molybdate
**cat**	catalyst
**CAS**	cerium(IV) sulfate
**CCDC**	Cambridge Crystallographic Data Centre
**cc-pVDZ**	Dunning's correlation-consistent double-zeta + polarization basis sets
**CDI**	1,1'-carbonyldiimidazole
**DHK**	Douglas-Kroll-Hess 2nd order scalar relativistic calculations
**DHKSO**	Douglas-Kroll-Hess 4th order relativistic calculations incorporating spin-orbit coupling
**DMAP**	4-dimethylaminopyridine
**DMSO**	dimethylsulfoxide
**ECP**	effective core potential
**eq**	equivalents
**ESI**	electrospray ionisation
**Et**	ethyl
**Grubbs-II**	Grubbs catalyst 2nd generation
**h**	hour
**HRMS**	High resolution mass spectrometry
**^{i}Pr**	isopropyl
**LDA**	lithium diisopropylamide
**LTMP**	lithium tetramethylpiperidide
**Me**	methyl
**min**	minute
**MS**	molecular sieves
**n.d.**	not determined
**^{n}Bu**	normal-butyl

**NMO**	*N*-methylmorpholine *N*-oxide
**NMR**	nuclear magnetic resonance
**Ph**	phenyl
**R_f**	retention factor
**rt**	room temperature
**^{t}Bu**	tertiary-butyl
**TFA**	trifluoroacetyl
**THF**	tetrahydrofuran
**TLC**	thin layer chromatography
**TMEDA**	tetramethylethylenediamine
**TMP**	2,2,6,6-tetramethylpiperidine
**TPAP**	tetrapropylammonium perruthenate
**TS**	transition state
**SARC**	Segmented all-electron relativistically contracted
**SET**	single electron transfer
**SOC**	spin orbit coupling

1.2 Preparation of reagents

Samarium(II) diiodide (SmI_2) [1]

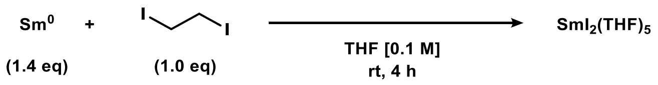

An oven-dried Schlenk flask, equipped with a stirrer bar, was flushed with a strong flow of N_2 (30 min). Subsequently, the flask was loaded with samarium metal (~40 mesh, 1.4 eq) and purified diiodoethane (1.0 eq). The flask was flushed with N_2 (30 min), before freshly distilled and degassed THF (0.1 M) was added followed by continuous vigorous stirring (> 4 h, rt). Finally, prior to use the resultant deep blue mixture was allowed to settle (>15 min) prior to use.

Volumetric titration procedure

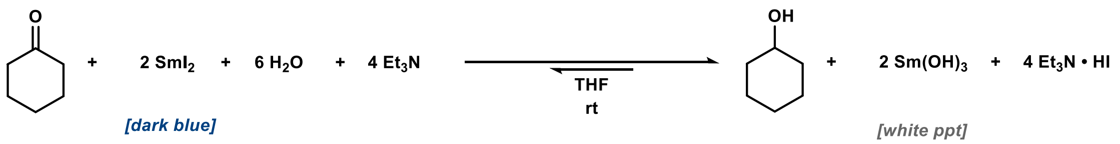

Procedure adapted from that of Hilmersson and co-workers [2]

An oven-dried vial, equipped with a stirrer bar, was flushed with a strong flow of N_2 (10 min). Subsequently, the vial was charged Et_3N (100 µL, 0.72 mmol), H_2O (20 µL) and the freshly prepared SmI_2 (2.5 mL, unknown concentration in THF) was added followed by continuous vigorous stirring where a deep purple solution formed. In a separate oven-dried vial, equipped with a stirrer bar, was added cyclohexanone (100 µL, 94.8 mg) and freshly distilled and degassed THF (4 mL) and the solution stirred (rt, 5 min). Then the titrant ketone solution was added dropwise with vigorous stirring until the endpoint – deep blue/purple solution had turned a pale green, and a white precipitate had formed – and the volume noted. The final calculation of [SmI_2] could then be carried out using the balanced equation, shown above, simplified to **Eq.1**.

$$\frac{\text{Mass of ketone (mg)} \times \text{Titrant vol added (mL)}}{\left(\frac{\text{MW of ketone}\left(\text{g mol}^{-1}\right) \times \text{Total titrant vol (4 mL)}}{2}\right) \times \text{SmI}_2 \text{ vol (2.5 mL)}} = [\text{SmI}_2]\text{ M} \qquad \textbf{Eq. 1}$$

Change in concentration of SmI_2 in THF over time

To investigate the change in concentration of SmI_2 in THF, batches of SmI_2 (5 x 5 mL) were prepared and each batch was titrated once using the above procedure after a time-interval aligning with those in **Figure 2.** Day 0 [0.095 M]; Day 1 [0.085 M]; Day 2 [0.081 M]; Day 3 [0.083 M]; and Day 4 [0.075 M].

1-Propenylmagnesium bromide

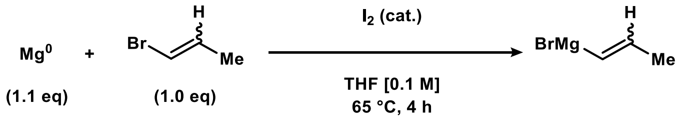

Procedure adapted from that of Feringa and co-workers.[3]

A flame-dried Schlenk flask, equipped with a stirrer bar, and charged with magnesium turnings (284 mg, 11.7 mmol, 1.1 eq) was flame-dried under vacuum (15 min), then under a nitrogen atmosphere, a single I_2 crystal was added with vigorous stirring and gentle heating until a purple vapour was observed. Dry THF (12 mL) was added, followed by 1-bromoprop-1-ene (902 µL, 10.5 mmol, 1.0 eq), and a dark olive solution formed. The mixture was heated (65 °C, 4 h), then cooled (rt) prior to use.

Lithium diisopropylamide (LDA)

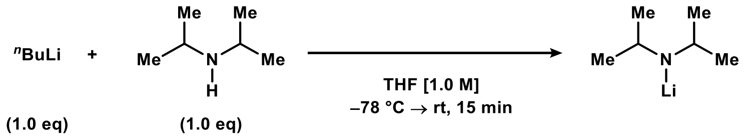

An oven-dried two-neck flask, equipped with a stirrer bar, was flushed with a strong flow of N_2 (15 min) then sealed. Subsequently, the flask was charged with diisopropylamine (24 mmol, 1.0 eq) followed by THF (24 mL). The resultant solution was cooled with stirring (-78 ºC, 30 min) followed by dropwise addition of ^{n}BuLi (24 mmol, 1.0 M in hexane, 1.0 eq). The light-yellow solution was warmed (–78 ºC → rt, 15 min) before being cooled in an ice bath prior to use.

Lithium tetramethylpiperidide (LTMP) [4]

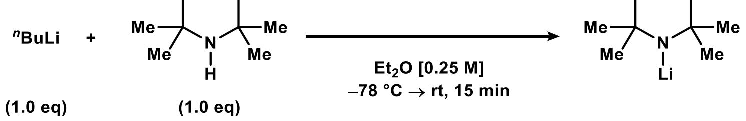

An oven-dried two-neck flask, equipped with a stirrer bar, was flushed with a strong flow of N_2 (15 min) then sealed. Subsequently, the flask was charged with 2,2,6,6-tetramethylpiperidine (26 mmol, 1.0 eq) followed by Et_2O (100 mL). The resultant solution was cooled with stirring (–78 ºC, 30 min) followed by

dropwise addition of ⁿBuLi (26 mmol, 1.0 M in hexane, 1.0 eq). The light-yellow solution was warmed (-78 ºC → rt, 15 min) before being cooled in an ice bath prior to use.

Note: *The resultant LTMP solution must be prepared as a dilute solution [<0.25 M] to prevent precipitation of lithiated aggregates.*

Dimethyl 1-diazo-2-oxopropylphosphonate (SI-1)

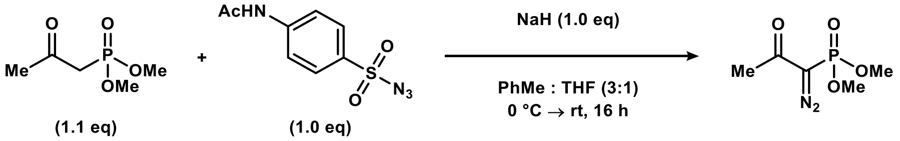

2-Oxopropylphosphonate and of *p*-acetamidobenzenesulfonyl azide were both synthesised according to literature procedures.[5] An oven-dried three-necked flask was charged with dimethyl 2-oxopropylphosphonate (5.40 g, 32.5 mmol, 1.1 eq) in toluene (30 mL) and the solution cooled (0°C, 10 min). NaH (1.31 g of 55% in paraffin, 30.0 mmol, 1.0 eq) was added portion wise. After the gas evolution had ceased, a solution of *p*-acetamidobenzenesulfonyl azide (7.18 g, 30.0 mmol, 1.0 eq) in THF (10 mL) was added dropwise; the highly viscous suspension slowly discoloured to yellow-brown and the mixture stirred and gradually warmed (0°C to rt, 16 h). The mixture was diluted with petroleum ether (100 mL), filtered through a pad of Celite, rinsed thoroughly with Et_2O (500 mL), and the solvents removed *in vacuo*. The resultant crude yellow oil was used directly without further purification. For characterisation, data a sample was purified by column chromatography R_f = 0.25 (silica gel, petroleum ether:EtOAc = 1:1) to afford the target product **SI-1** as a yellow oil.

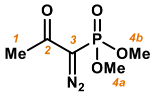

^{1}H NMR (500 MHz, $CDCl_3$) δ 3.83 (d, *J* = 11.8 Hz, 6H, *3H-4a* + *3H-4b*), 2.26 (d, *J* = 1.7 Hz, 3H, *3H-1*).

^{13}C{H} NMR (126 MHz, $CDCl_3$) δ 190.0 (d, *J* = 13.0 Hz, *C-2*), 60.8 (br, *C-3*), 53.7 (d, *J* = 5.5 Hz, (*C-4*), 27.3 (*C-1*).

Data in accordance with those previously reported.[5]

Dimethyl (2-oxo-4-phenylbutyl)phosphonate (SI-2)

Procedure adapted from Procter and co-workers. [6]

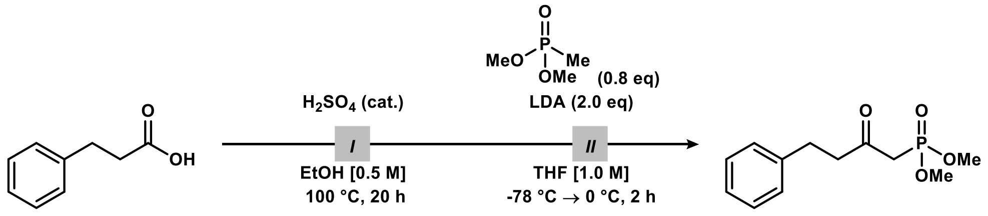

Step I: To a solution of hydrocinnamic acid (3.00 g, 22.0 mmol, 1.0 eq) in EtOH (95%, 40 mL) was added H_2SO_4 (0.1 mL, 2.0 mmol, 10 mol%) and the solution heated under reflux in air (100 ºC, 20 h). Subsequently, the reaction mixture was cooled to room temperature and neutralized with saturated aqueous Na_2CO_3 (20 mL). The mixture was then extracted with CH_2Cl_2 (3 × 15 mL) and the combined organic phases were dried over anhydrous $MgSO_4$ and concentrated *in vacuo*. The target Ethyl hydrocinnamate was obtained as a viscous oil and was used directly without further purification.

Step II: An oven dried flask and stirrer bar was cooled under vacuum and back-filled with N_2 (three cycles). The flask was charged with ethyl hydrocinnamate (3.00 g, 16.8 mmol, 1.4. eq), methyl dimethylphosphonate (1.30 mL, 12.0 mmol, 1.0 eq), and dry THF (15 mL) and cooled (0 ºC, 30 min). Freshly prepared LDA in THF (24 mmol, 24 mL, 1.0 M, 2 eq) was cooled (0 ºC) and added dropwise via cannula (over 60 min) and the resultant yellow solution was further stirred on complete addition (0 ºC, 2 h). The reaction mixture was quenched with saturated aqueous NH_4Cl (40 mL), the phases were separated, and the aqueous phase was extracted with Et_2O (3 × 40 mL). The combined organic phases were dried over anhydrous $MgSO_4$ and concentrated in vacuo. The target phosphonate ester **SI-2** was isolated by column chromatography on silica gel R_f = 0.30 (silica gel, petroleum ether:EtOAc = 1:1).

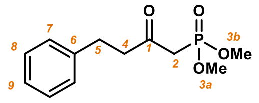

^{1}H NMR (400 MHz, $CDCl_3$) δ 7.34 – 7.24 (m, 2H, 2*H-8*), 7.23 – 7.14 (m, 3H, 2*H-7* + *H-9*), 3.75 (d, *J* = 11.3 Hz, 6H, 3*H-3a* + 3*H-3b*), 3.08 (d, *J* = 22.8 Hz, 2H, 2*H-2*), 3.00 – 2.86 (m, 4H, 2*H-4* + 2*H-5*).

^{13}C{H} NMR (101 MHz, $CDCl_3$) δ 201.1 (*C-1*), 140.7 (*C-6*), 128.6 (*C-8*), 128.5 (*C-7*), 126.3 (*C-9*), 53.2 (*C-3a*), 53.2 (*C-3b*), 45.7 (*C-5*), 41.5 (d, *J* = 128.0 Hz, *C-2*), 29.5 (*C-4*).

^{31}P NMR (162 MHz, $CDCl_3$) δ 22.5 (nonet, *J* = 11.3 Hz).

IR (neat, cm^{-1}): 3027, 2955, 2853, 1715, 1604, 1497, 1454, 1402 (fingerprint region excluded).

Date in accordance with those previously reported.[7]

Diphenylisopropylsulfonium tetrafluoroborate (SI-3)

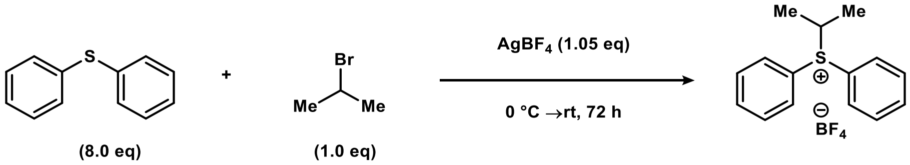

Procedure adapted from Barbasiewicz and co-workers: [8]

A flask was charged with $AgBF_4$ (2.04 g, 10.5 mmol, 1.05 eq), purged (N_2 gas, 15 min), immersed in ice-water bath (ca. 2-3°C, 10 min), and protected from light with aluminium foil. A mixture of diphenyl sulfide (13.4 mL, 80.0 mmol, 8.0 eq) and isopropyl bromide (950 µL, 10 mmol, 1.0 eq) was added dropwise and the mixture was left to gradually warm (0 ºC → rt, 72 h). Acetone (100 mL) was added, the mixture was filtered through a pad of silica gel (4 x 4 cm), eluted with acetone (150 mL) and the filtrate concentrated *in vacuo*. The resultant residue was diluted in CH_2Cl_2 (250 mL), filtered through a pad of celite (4 x 4 cm), and the filtrate concentrated *in vacuo*. Et_2O (10 mL) was added to the resultant slurry and sonicated to precipitate out the sulfonium salt which was recrystallised from CH_2Cl_2:Et_2O (1:10, 10 mL) to yield **SI-3** (1.04 g, 3.30 mmol, 33% yield) as pale-yellow crystals.

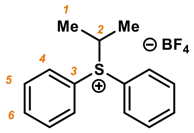

^{1}H NMR (500 MHz, $CDCl_3$) δ 8.11 - 8.05 (m, 4H, 4*H-4*), 7.78 - 7.67 (m, 6H, 4*H-5* + 2*H-6*), 5.16 (m, 1H, *H-2*), 1.52 (d, *J* = 6.6 Hz, 6H, *6H-1*).

^{13}C{H} NMR (126 MHz, $CDCl_3$) δ 135.1 (*2C-6*), 131.9 (*4C-4*), 131.4 (*4C-5*), 123.8 (*2C-3*), 51.1 (*C-2*), 18.8 (*C-1*).

^{19}F NMR (471 MHz, $CDCl_3$) δ -152.07.

Melting Point (from CH_2Cl_2: Et_2O): 111 - 113 °C (lit. 113 °C).

Data in accordance with those previously reported.[8]

1.3 Synthesis of radical traps

Seyferth-Gilbert Homologation with the Ohira-Bestmann Modification

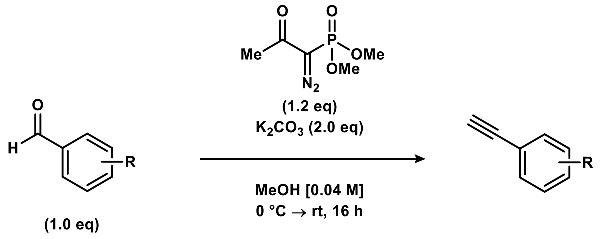

An oven-dried flask equipped with a magnetic stir bar was charged with aldehyde (1.00 mmol, 1.0 eq) and anhydrous MeOH (0.04 M) and cooled (0 °C, 15 min). K_2CO_3 (2.00 mmol, 2.00 eq) was added and the mixture stirred (0 °C, 15 min) followed by dropwise addition of dimethyl 1-diazo-2-oxopropylphosphonate (1.2 mmol, 1.2 eq). The resulting yellow solution was then gradually warmed and stirred continuously (0 °C to rt, 16 h). The reaction contents were quenched dilution with Et_2O (30 mL) and the dropwise addition of saturated aqueous $NaHCO_3$ (10 mL). The phases were separated, and the aqueous phase was extracted with Et_2O (3 × 30 mL). The combined ethereal phases were dried over anhydrous MgSO4, filtered, and concentrated *in vacuo*. The crude product was isolated by column chromatography on silica gel using petroleum ether and EtOAc as the eluent mixture to afford the target alkyne.

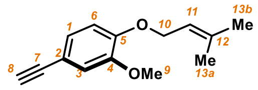

4-Ethynyl-2-methoxy-1-((3-methylbut-2-en-1-yl)oxy)benzene (SI-4)

3-Methoxy-4-((3-methylbut-2-en-1-yl)oxy)benzaldehyde was prepared according to the literature procedure from Snyder and co-workers.[9] Prepared according to the above procedure from 3-methoxy-4-((3-methylbut-2-en-1-yl)oxy)benzaldehyde (440 mg, 2.00 mmol), K_2CO_3 (552 mg, 4.00 mmol, 2.0 eq), and dimethyl 1-diazo-2-oxopropylphosphonate (460 mg, 2.40 mmol, 1.2 eq). The resulting crude residue was purified by column chromatography R_f = 0.60 (silica gel, petroleum ether:EtOAc = 9:1) to afford the target alkyne **SI-4** as a colorless amorphous solid (230 mg, 1.06 mmol, 51% yield).

^{1}H NMR (400 MHz, $CDCl_3$) δ 7.07 (dd, *J* = 8.2, 1.9 Hz, 1H, *H-6*), 6.98 (d, *J* = 1.9 Hz, 1H, *H-3*), 6.80 (d, *J* = 8.3 Hz, 1H, *H-1*), 5.50 (tq, *J* = 5.3, 1.4 Hz, 1H, *H-11*), 4.59 (d, *J* = 6.7 Hz, 2H, *2H-10*), 3.86 (s, 3H, *3H-9*), 3.00 (s, 1H, *H-8*), 1.80 – 1.71 (m, 6H, *3H-13a* + *3H-13b*).

^{13}C{H} NMR (101 MHz, $CDCl_3$) δ 149.2 (*C-5*), 149.0 (*C-4*), 138.1 (*C-12*), 125.4 (*C-6*), 119.5 (*C-11*), 114.9 (*C-3*), 114.1 (*C-2*), 112.6 (*C-1*), 83.9 (*C-7*), 75.6 (*C-8*), 65.7 (*C-10*), 55.9 (*C-9*), 25.9 (*C-13a*), 18.3 (*C-13b*).

Melting Point (from petroleum ether: EtOAc): 38 – 40 °C.

IR (neat, cm^{-1}): 3285, 2968, 2933, 2861, 2104, 1598, 1577, 1507, 1462, 1446, 1409 (fingerprint region excluded).

HRMS (ESI+): calculated for $C_{14}H_{16}O_2Na$ (M^+Na^+): 216.1150 Found: 216.1150.

6-Ethynylquinoline (SI-5)

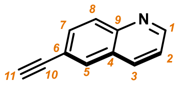

Prepared according to the above procedure from quinoline-6-carbaldehyde (157 mg, 1.00 mmol), K_2CO_3 (276 mg, 2.00 mmol, 2.0 eq), and dimethyl 1-diazo-2-oxopropylphosphonate (230 mg, 1.20 mmol, 1.2 eq). The resulting crude residue was purified by column chromatography R_f = 0.70 (silica gel, petroleum ether:EtOAc = 1:1) to afford the target alkyne **SI-5** as a yellow amorphous solid (129 mg, 0.84 mmol, 84% yield).

^{1}H NMR (400 MHz, $CDCl_3$) δ 8.93 (dd, *J* = 4.3, 1.8 Hz, 1H), 8.12 (dt, *J* = 8.4, 1.4 Hz, 1H), 8.05 (dt, *J* = 8.6, 0.8 Hz, 1H), 8.00 (d, *J* = 1.9 Hz, 1H), 7.76 (dd, *J* = 8.7, 1.9 Hz, 1H), 7.43 (dd, *J* = 8.3, 4.2 Hz, 1H), 3.19 (s, 1H, *H-11*).

^{13}C{H} NMR (101 MHz, $CDCl_3$) δ 151.4 (*C-1*), 148.0 (*C-9*), 135.9, 132.4, 132.2, 129.8 (*C-4*), 128.0, 122.0, 120.5 (*C-6*), 83.3 (*C-11*), 78.6 (*C-10*).

Data in accordance with those previously reported.[10]

1,3,5-Tribromo-2-ethynyl-4-methoxybenzene (SI-6)

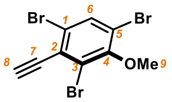

Prepared according to the above procedure from 2,4,6-tribromo-3-methoxybenzaldehyde (746 mg, 2.00 mmol, 1.0 eq), K_2CO_3 (552 mg, 4.00 mmol, 2.0 eq), and dimethyl 1-diazo-2-oxopropylphosphonate (460 mg, 2.40 mmol, 1.2 eq). The resulting crude residue was purified by column chromatography R_f = 0.60 (silica gel, petroleum ether:EtOAc = 9:1) to afford the target alkyne **SI-6** as a white amorphous solid (525 mg, 1.42 mmol, 72% yield).

^{1}H NMR (400 MHz, $CDCl_3$) δ ^{1}H NMR (400 MHz, $CDCl_3$) δ 7.79 (s, 1H, *H-6*), 3.88 (s, 3H, *3H-9*), 3.73 (s, 1H, *H-8*).

^{13}C{H} NMR (101 MHz, $CDCl_3$) δ 154.3 (*C-4*), 135.4 (*C-6*), 127.0, 123.1, 121.5, 119.3, 87.6 (*C-8*), 80.6 (*C-7*), 60.7 (*C-9*).

Data were in accordance with those previously reported.[11]

Preparation of 1-ethynylcyclohept-2-ene (SI-7)

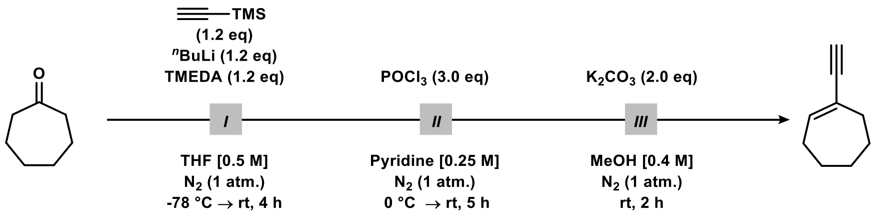

Procedures adapted from B. Chattopadhyay and co-workers: [12]

Step I: A flame-dried 100 mL 2-neck round-bottom flask was charged with trimethylsilylacetylene (1.7 mL, 12 mmol, 1.2 eq) and TMEDA (1.8 mL, 12 mmol, 1.2 eq) followed by dry THF (15 mL) and cooled (–78 °C, 15 min). ^{n}BuLi [titrated prior to use ~2.3 M in hexanes] (5.2 mL, 12 mmol, 1.2 eq) was added dropwise and the resulting pale-yellow solution was stirred (–78 °C, 2 h). The corresponding ketone (10 mmol, 1.0 eq) in dry THF (5 mL) was added dropwise and, on complete addition, the reaction mixture was allowed to gradually warm (–78 °C → r.t, 2 h) and then stirred (rt, 1 h). The reaction mixture was quenched by addition of saturated aqueous NH_4Cl (25 mL) and the reaction mixture was then extracted with EtOAc (2 x 50 mL), the organic phases were pooled and washed successively with H_2O (50 mL) and saturated aqueous NaCl (50 mL). The organic phase was dried over anhydrous $MgSO_4$ and concentrated *in vacuo* and the crude product was isolated by column chromatography on silica gel using petroleum ether and EtOAc as the eluent mixture to afford the target alcohol.

Step II: A flame-dried 2-neck round-bottom flask was charged with the corresponding alcohol (5.0 mmol, 1.0 eq) and distilled pyridine (20 mL, 0.25 M). The reaction mixture was cooled (0 °C, 15 min) and $POCl_3$ (1.4 mL, 15 mmol, 3.0 eq) was added dropwise. On complete addition the cold bath was removed, and the reaction mixture stirred (rt, 5 h). The reaction mixture was then cooled (0 °C, 15 min) and was quenched by slow addition of H_2O (50 mL) and allowed to gradually warm (0 °C → rt, 15 min). The reaction mixture was then extracted with Et_2O (2 x 50 mL) and the combined organic phases were washed successively with 1N HCl (2 x 50 mL) and saturated aqueous NaCl (50 mL). The organic phase

was dried over anhydrous $MgSO_4$ and concentrated *in vacuo* and the crude product was used without any further purification.

Step III: A flame-dried flask was charged with the corresponding olefin (5.0 mmol, 1.0 eq) and dry MeOH (10 mL). K_2CO_3 (1.11 g, 8.0 mmol, 2.0 eq) was added in one-portion and the reaction mixture was stirred (rt, 2h). The resultant mixture was extracted with pentane (2 x 50 mL), the combined organic phases were washed successively with H_2O (50 mL) and saturated aqueous NaCl (50 mL). The organic phase was dried over anhydrous $MgSO_4$ and concentrated *in vacuo* and the crude product was used without any further purification.

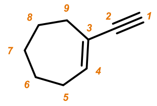

1-Ethynylcyclohept-1-ene (SI-7)

Prepared from cycloheptanone (1.50 g, 10 mmol). The resulting crude residue was purified by column chromatography R_f = 0.45 (silica gel, pentane) to afford the target alkyne **SI-7** as a colorless oil (533 mg, 4.43 mmol, 88% yield, over 3 steps).

^{1}H NMR (400 MHz, $CDCl_3$) δ 6.39 (t, *J* = 6.7 Hz, 1H, *H-4*), 2.85 (s, 1H, *H-1*), 2.38 – 2.29 (s, 2H, *CH_2*), 2.25 – 2.14 (m, 2H, *CH_2*), 1.79 – 1.69 (m, 2H, *CH_2*), 1.63 – 1.46 (m, 4H, 2 *x CH_2*).

^{13}C{H} NMR (101 MHz, $CDCl_3$) δ 141.8 (*C-4*), 126.0 (*C-3*), 87.5 (*C-2*), 74.4 (*C-1*), 34.3 (*CH_2*), 32.2 (*CH_2*), 29.3 (*CH_2*), 26.6 (*CH_2*), 26.5 (*CH_2*).

IR (neat, cm^{-1}): 3295, 3027, 2921, 2850, 2090, 1447 (fingerprint region excluded).

HRMS (APCI+): calculated for C_9H_{13} (M^+H^+): 121.1012 Found: 121.1013.

Synthesis of (1*R*,5*S*)-2-ethynyl-6,6-dimethylbicyclo[3.1.1]hept-2-ene (SI-8)

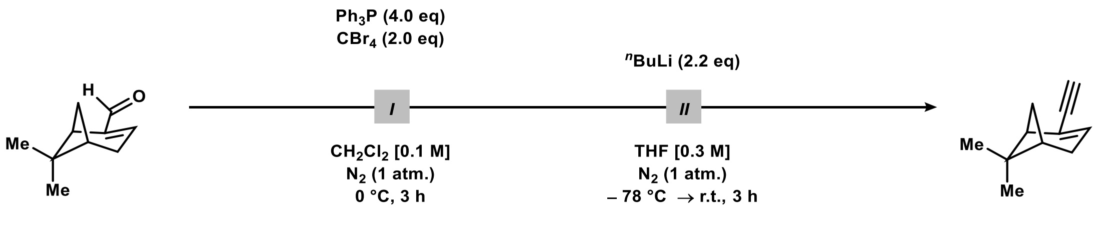

(-)-myrtenal *SI - 8*

Procedure adapted from S. Laschat and co-workers: [13]

Step I: A flame-dried flask was charged with Ph_3P (8.40 g, 32.0 mmol, 4.0 eq) and CH_2Cl_2 (40 mL), and the solution was cooled (0 °C, 10 min). Then, CBr_4 (5.30 g, 16.0 mmol, 2.0 eq) in dry CH_2Cl_2 (15 mL) was added dropwise to the cooled reaction mixture and stirred (0 °C, 15 min). (-)-myrtenal (1.22 mL,

8.00 mmol, 1.0 eq) was diluted in dry CH_2Cl_2 (15 mL) and added dropwise to the reaction mixture, followed by vigorous stirring (0 °C, 2 h). After this time, the cold bath was removed and the suspension was allowed to warm with stirring (rt, 1 h), then, hexane (250 mL) was added, and the resulting suspension filtered through a Celite pad, and the filtrate concentrated *in vacuo*. The resultant brown residue was resuspended in hexane (150 mL) and refiltered through a Celite pad, and the filtrate concentrated *in vacuo*. The resultant pale brown oil was used directly in the next step without further purification.

Step II: A flame-dried flask was charged with (1*R*,5*S*)-2-(2,2-dibromovinyl)-6,6-dimethylbicyclo[3.1.1]hept-2-ene (2.93 g, 8.0 mmol, 1.0 eq) and dry THF (20 mL). The resultant solution was cooled (–78 °C, 15 min), and ^{n}BuLi [titrated prior to use ~2.5 M in hexanes] (7.0 mL, 17.6 mmol, 2.2 eq) was added dropwise and the resulting pale-yellow solution was stirred and gradually warmed (–78 °C → rt, 3 h). The reaction mixture was quenched by addition of saturated aqueous NH_4Cl (25 mL) and the reaction mixture was then extracted with Et_2O (2 x 50 mL), the organic phases were pooled and washed successively with H_2O (50 mL) and saturated aqueous NaCl (50 mL). The organic phase was dried over anhydrous $MgSO_4$ and concentrated *in vacuo* and the crude product was purified column chromatography R_f = 0.50 (silica gel, pentane) eluent to afford the target alkyne **SI-8** as a colorless fragrant oil (482 mg, 3.30 mmol, 41% yield).

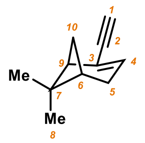

^{1}H NMR (400 MHz, $CDCl_3$) δ 6.08 (tt, *J* = 3.3, 1.5 Hz, 1H, *H-4*), 2.98 (q, *J* = 0.9 Hz, 1H, *H-1*), 2.42 (dt, *J* = 9.0, 5.6 Hz, 1H, *H-10b*), 2.36 – 2.29 (m, 2H, *2H-5*), 2.28 (td, *J* = 5.7, 1.5 Hz, 1H, *H-9*), 2.11 (ttd, *J* = 5.9, 2.9, 1.3 Hz, 1H, *H-6*), 1.29 (s, 3H, *3H-8a*), 1.24 (d, *J* = 9.0 Hz, 1H, *H-10a*), 0.89 (s, 3H, *3H-8b*).

^{13}C{H} NMR (101 MHz, $CDCl_3$) δ ^{13}C NMR (101 MHz, $CDCl_3$) δ 132.9 (*C-4*), 129.2 (*C-3*), 84.6 (*C-2*), 76.7 (*C-1*), 46.9 (*C-9*), 40.3 (*C-6*), 38.1 (*C-7*), 32.2 (*C-5*), 31.5 (*C-10*), 26.1 (*C-8a*), 21.1 (*C-8b*).

IR (neat, cm^{-1}): 3295, 2921, 2849, 2090, 1446 (fingerprint region excluded).

Synthesis of 4,8-dimethylnona-3,7-dien-1-yne (SI-9)

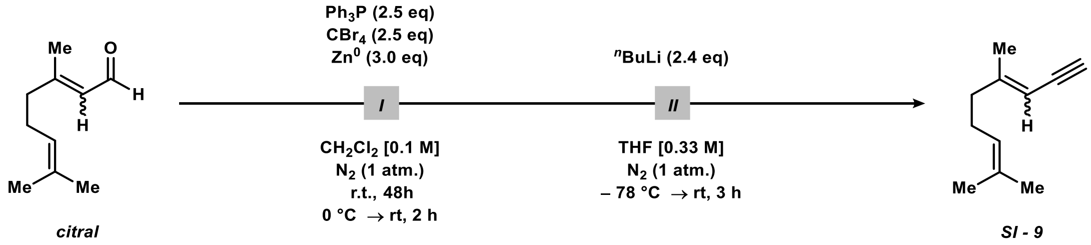

Procedures adapted from A. Suzuki and co-workers: [14]

Step I: A flame-dried flask was charged with Ph_3P (6.60 g, 25.0 mmol, 2.5 eq), CBr_4 (8.30 g, 25.0 mmol, 2.5 eq), and Zn powder (1.95 g, 30.0 mmol, 3.0 eq) followed by dry CH_2Cl_2 (115 mL) and the resultant suspension was stirred vigorously (rt, 48 h). The brown suspension was cooled (0 °C, 30 min) and citral (95% purity, 2.0 mL, 10 mmol, 1.0 eq) in CH_2Cl_2 (10 mL) was added dropwise followed by vigorous stirring (0 °C, 1 h). After this time, the cold bath was removed and the suspension was allowed to warm and stirred (rt, 1 h), then, hexane (250 mL) was added, and the suspension was filtered through a celite pad, and the filtrate concentrated *in vacuo*. The resultant brown residue was resuspended in hexane (150 mL) and refiltered through a celite pad, and the filtrate concentrated in vacuo. The resultant pale brown oil was used directly in the next step without further purification.

Step II: A flame-dried flask was charged with 1,1-dibromo-4,8-dimethyl-1,3,7-nonatriene (3.49 g, 10.0 mmol, 1.0 eq) and dry THF (30 mL). The resultant solution was cooled (–78 °C, 15 min), and ^{n}BuLi [titrated prior to use ~2.3 M in hexanes] (10.4 mL, 24.0 mmol, 2.4 eq) was added dropwise and the resulting pale-yellow solution was stirred and gradually warmed (–78 °C → rt, 3 h). The reaction mixture was quenched by addition of saturated aqueous NH_4Cl (25 mL) and the reaction mixture was then extracted with Et_2O (2 x 50 mL), the organic phases were pooled and washed successively with H_2O (50 mL) and saturated aqueous NaCl (50 mL). The organic phase was dried over anhydrous $MgSO_4$ and concentrated *in vacuo*. The crude product was purified by Kugelrohr distillation (78 °C, 15 mbar) to yield the target alkyne **SI-9** a colorless fragrant oil and as a mixture of double bond isomers (1.13 g, 7.63 mmol, 76% yield, d.r. = 1.3:1).

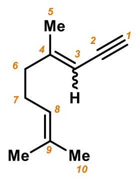

^{1}H NMR (400 MHz, $CDCl_3$) δ 5.29 – 5.23 (m, 2H, *H-3 major* + *H-3 minor*), 5.17 – 5.11 (m, 1H, *H-8 minor*), 5.11 – 5.03 (m, 1H, *H-8 major*), 3.02 (d, *J* = 2.2 Hz, 1H, *H-1 major*), 2.98 (d, *J* = 2.2 Hz, 1H, *H-1 minor*), 2.38 – 2.30 (m, 2H, *H-6a major* + *H-6a minor*), 2.19 – 2.06 (m, 6H, *H-6b major* + *H-6b minor* + *2H-7*

major + 2H-7 minor), 1.94 – 1.89 (m, 3H), 1.82 – 1.77 (m, 3H), 1.72 – 1.64 (m, 6H), 1.62 (s, 3H), 1.60 (s, 3H).

^{13}C{H} NMR (101 MHz, $CDCl_3$) δ 154.5 (*C-4 minor*), 154.3 (*C-4 major*), 132.3 (*C-9 major*), 132.2 (*C-9 minor*), 123.6 (*C-8 minor*), 123.3 (*C-8 major*), 104.4 (*C-3 minor*), 103.8 (*C-3 major*), 81.9 (*C-2 major*), 81.6 (*C-2 minor*), 79.5 (*C-1 major*), 79.2 (*C-1 minor*), 38.7, 34.9, 26.3, 26.1, 25.74, 25.71, 22.7, 19.4, 17.7, 17.6.

IR (neat, cm^{-1}): 3307, 2968, 2916, 2856, 2090, 1443 (fingerprint region excluded).

HRMS (APCI+): calculated for $C_{11}H_{17}$ (M^+H^+): 149.1325 Found: 149.1329.

1.4. Starting material preparation

1.4.1 General Procedures

General Procedure 1: 3-step synthesis of alkyl cyclopropyl ketones

Procedure adapted from Procter and co-workers: [6]

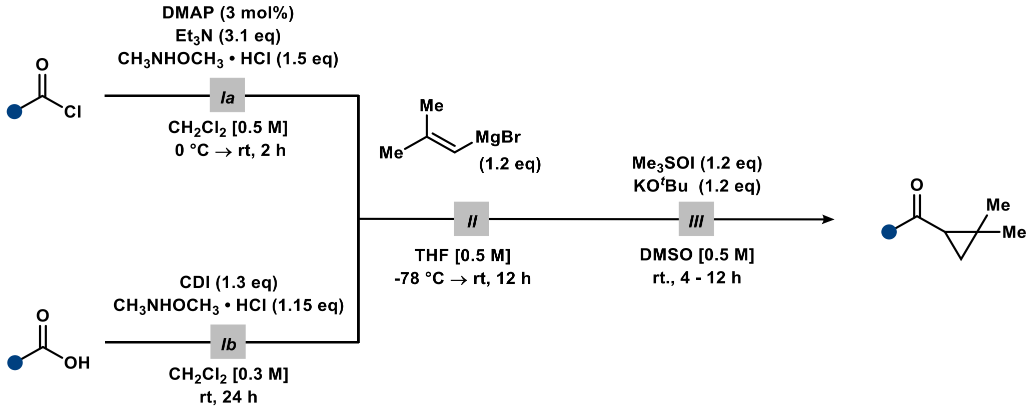

Step Ia: To a solution of the corresponding acyl chloride (10.0 mmol, 1.0 eq) in CH_2Cl_2 (20 mL) was added 4-dimethylaminopyridine (DMAP) (40 mg, 0.30 mmol, 3 mol%), *N,O*-dimethylhydroxylamine hydrochloride (1.46 g, 15.0 mmol, 1.5 eq) and the resultant solution was cooled (0 °C, 15 min). Triethylamine (3.12 g, 31 mmol, 3.1 eq) was added dropwise to the vigorously stirred reaction mixture. On complete addition, the reaction mixture was allowed to gradually warm (0°C to r.t, 2 h). The reaction mixture was diluted with CH_2Cl_2 (60 mL) and washed with HCl (1 N, 2 x 30 mL). The organic phase was separated and washed successively with a saturated aqueous $NaHCO_3$ (2 x 30 mL) and saturated aqueous NaCl (1 x 30 mL). The organic phase was dried over anhydrous $MgSO_4$ and concentrated *in vacuo* to afford the crude product which was used directly without further purification.

Step Ib: To a solution of the corresponding carboxylic acid (10.0 mmol, 1.0 eq) in dry CH_2Cl_2 (30 mL) was added carbonyldiimidazole (CDI) (1.87 g, 11.5 mmol, 1.15 eq) portion wise and the solution stirred (rt, 1 h). After this time, N_2 was bubbled through the solution (30 min). *N,O*-Dimethylhydroxylamine hydrochloride (1.27 g, 13.0 mmol, 1.3 eq) was added in one portion and the reaction mixture was stirred (rt, 24 h). The reaction mixture was quenched by addition of saturated aqueous NH_4Cl (30 mL) and extracted with CH_2Cl_2 (3 × 70 mL). The combined organic phase was dried over anhydrous $MgSO_4$ and concentrated *in vacuo* to afford the crude product which was used directly without further purification.

Step II: The corresponding Weinreb amide was transferred to a round bottom flask under N_2 followed by dry THF (5 mL $mmol^{-1}$). The flask was cooled (−78 ºC, 15 min) and commercially available 2-methyl-1-propenylmagnesium bromide solution (12.0 mmol, 0.5 M in THF, 1.20 eq) was added dropwise to the reaction mixture followed by stirring (−78 ºC, 15 min). The reaction mixture was gradually warmed (−78 ºC to rt, 12 h) and was quenched by addition of saturated aqueous NH_4Cl (20 mL). The phases were separated, and the aqueous phase was extracted with CH_2Cl_2 (3 × 70 mL). The organic phases were dried over anhydrous $MgSO_4$ and concentrated *in vacuo*. The resultant crude material was purified by column chromatography on silica gel with petroleum ether and EtOAc as the eluent mixture to afford the target enone.

Step III: To K*t*-BuO (1.2 eq) and trimethylsulfoxonium iodide (1.2 eq) under N_2 in a round bottom flask was added dry DMSO (3.5 mL $mmol^{-1}$) and the reaction mixture stirred until the solution became clear (rt., 15 min). The enone from step II (1.0 eq) was dissolved in dry DMSO (2 mL $mmol^{-1}$) and the solution added (dropwise) into the reaction mixture. The reaction mixture was allowed to stir (rt, 4 - 12 h) before dilution with Et_2O (30 mL) and addition of ice-cold water (equal volume to DMSO). The aqueous phase was extracted with Et_2O (3 × 15 mL), and the organic phase was dried over anhydrous $MgSO_4$ and concentrated *in vacuo.* The and the crude product was isolated by column chromatography on silica gel using petroleum ether and either Et_2O or EtOAc as the eluent mixture to afford the target cyclopropyl ketone.

General Procedure 2: 2-step synthesis of alkyl cyclopropyl ketones

Procedure adapted from Procter and co-workers: [6]

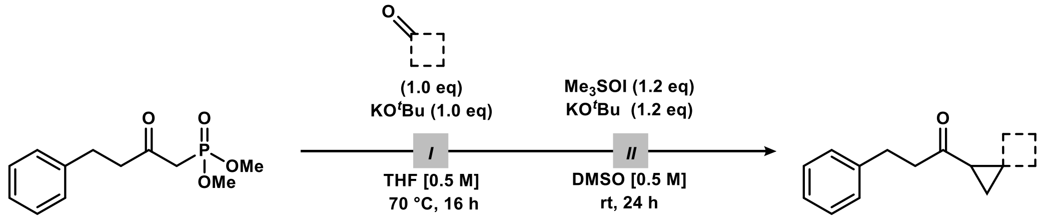

Step I: An oven-dried microwave vial and stirrer bar was cooled under N_2, charged with K*t*-BuO (224 mg, 2.0 mmol, 1.0 eq) and sealed. Dry THF (4 mL) was added, and the mixture was vigorously stirred (rt, 2 min). The phosphonate ester **SI-2** (512 mg, 2.0 mmol, 1.0 eq) was added dropwise and the mixture was stirred (rt, 2 h) during which a white precipitate formed. Subsequently, the corresponding ketone (2.0 mmol, 1.0 eq) was added in one portion and the mixture heated (70 ºC, 16 h). The reaction mixture was then filtered through cotton wool and diluted with Et_2O (15 mL) followed by the addition of saturated aqueous NaCl (20 mL). The aqueous phase was extracted with Et_2O (3 × 10 mL) and the combined organic phases were dried over anhydrous $MgSO_4$ and concentrated *in vacuo* to give the crude product. The targeted enone was isolated by column chromatography on silica using petroleum ether and Et_2O as the eluent mixture.

Step II: To K*t*-BuO (1.2 eq) and trimethylsulfoxonium iodide (1.2 eq) under N_2 in a round bottom flask was added dry DMSO (3.5 mL $mmol^{-1}$) and the reaction mixture stirred until the solution became clear (rt., 15 min). The corresponding enone from step III (1.0 eq) was dissolved in dry DMSO (2 mL $mmol^{-1}$) and the solution added (dropwise) into the reaction mixture. The reaction mixture was allowed to stir (rt, 24-48 h) before dilution with Et_2O (30 mL) and addition of ice-cold water (equal volume to DMSO). The aqueous phase was extracted with Et_2O (3 × 15 mL), and the organic phase was dried over anhydrous $MgSO_4$. The combined ethereal phase was concentrated *in vacuo* and the crude product was isolated by column chromatography on silica gel using petroleum ether and either Et_2O or EtOAc as the eluent mixture to afford the target cyclopropyl ketone.

1.4.2. Synthesis and characterisation data for starting materials

1-(2,2-Dimethylcyclopropyl)-2-propylpentan-1-one (1a)

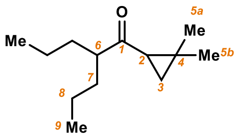

Prepared in 3 steps according to General Procedure 1 using valproic acid (1.44 g, 10.0 mmol). Step Ib: Weinreb amide obtained as a pale-yellow oil (1.87 g, 10.0 mmol, >99% yield). Step II: The resultant crude material was purified by column chromatography R_f = 0.25 (silica gel, petroleum ether:Et_2O = 19:1) to afford the target enone as a yellow oil (894 mg, 4.90 mmol, 49% yield). Step III: The resultant crude material was purified by column chromatography R_f = 0.30 (silica gel, petroleum ether: Et_2O = 19:1) to afford the target cyclopropyl ketone **1a** (541 mg, 2.76 mmol, 56% yield, 27% yield over 3 steps).

^{1}H NMR (400 MHz, $CDCl_3$) δ 2.55 (tt, *J* = 8.3, 5.4 Hz, 1H, *H-6*), 1.84 (dd, *J* = 7.6, 5.6 Hz, 1H, *H-2*), 1.68 – 1.53 (m, 2H, *2H-7a*), 1.44 – 1.21 (m, 6H, *2H-7b* + *2H-8a* + *2H-8b*), 1.19 (m, 4H, *H-3a* + *3H-5a*), 1.09 (s, 3H, *3H-5b*), 0.89 (t, *J* = 7.3 Hz, 6H, *3H-9a* + *3H-9b*), 0.81 (dd, *J* = 7.6, 3.8 Hz, 1H, *H-3b*).

^{13}C{H} NMR (101 MHz, $CDCl_3$) δ 212.7 (*C-1*), 53.8 (*C-6*), 34.4 (*C-2*), 34.2 (*C-7a*), 34.0 (*C-7b*), 27.5 (*C-5a*), 27.2 (*C-4*), 23.5 (*C-3*), 20.9 (*C-8a*), 20.7 (*C-8b*), 18.4 (*C-5b*), 14.4 (*C-9a*), 14.3 (*C-9b*).

IR (neat, cm^{-1}): 2956, 2931, 2873, 1688, 1459, 1434 (fingerprint region excluded).

HRMS (APCI+): calculated for $C_{13}H_{25}O$ (M^+H^+): 197.1900 Found: 197.1894.

1-(2,2-Dimethylcyclopropyl)-3-phenylpropan-1-one (1b)

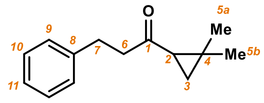

Prepared in 3 steps according to General Procedure 1 using hydrocinnamic acid (1.50 g, 10.0 mmol). Step Ib: Weinreb amide obtained as a pale-yellow oil (1.80 g, 9.33 mmol, 93% yield). Step II: The resultant crude material was purified by column chromatography R_f = 0.30 (silica gel, petroleum ether:EtOAc = 4:1) to afford the target enone as a yellow oil (1.15 g, 6.11 mmol, 65% yield). Step IIIa: The resultant crude material was purified by column chromatography R_f = 0.50 (silica gel, petroleum ether:EtOAc = 9:1) to afford the target cyclopropyl ketone **1b** (994 mg, 4.91 mmol, 80% yield, 48% yield over 3 steps).

^{1}H NMR (400 MHz, $CDCl_3$) δ 7.34 - 7.25 (m, 2H, *2H-9*), 7.25 - 7.13 (m, 3H, *2H-10* + *H-11*), 2.96 - 2.88 (m, 2H, *2H-6*), 2.88 - 2.79 (m, 2H, *2H-7*), 1.83 (dd, *J* = 7.5, 5.5 Hz, 1H, *H-2*), 1.27 (dd, *J* = 5.5, 3.9 Hz, 1H, *H-3a*), 1.17 (s, 3H, 3*H-5a*), 1.05 (s, 3H, *3H-5b*), 0.82 (dd, *J* = 7.6, 3.9 Hz, 1H, *H-3b*).

^{13}C{H} NMR (101 MHz, $CDCl_3$) δ 207.9 (*C-1*), 141.4 (*C-8*), 128.9 (2*C-9* + *2C-10*), 126.1 (*C-11*), 46.6 (*C-6*), 35.3 (*C-2*), 30.2 (*C-7*), 27.4 (*C-4*), 26.9 (*C-5a*), 23.3 (*C-3*), 18.3 (*C-5b*).

IR (neat, cm^{-1}): 3063, 3027, 2996, 2948, 2870, 1692, 1604 (fingerprint region excluded).

Data were in accordance with those previously reported.[6]

3-Cyclopentyl-1-(2,2-dimethylcyclopropyl)propan-1-one (1c)

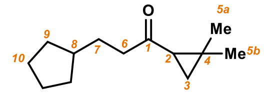

Prepared in 3 steps according to General Procedure 1 using 3-cyclopentylpropanoic acid (1.42 g, 10.0 mmol). Step Ib: Weinreb amide obtained as a yellow oil (1.52 g, 8.23 mmol, 82% yield). Step II: The resultant crude material was purified by column chromatography R_f = 0.30 (silica gel, petroleum ether:Et_2O = 9:1) to afford the target enone as a yellow oil (805 mg, 4.47 mmol, 56% yield). Step IIIa: The resultant crude material was purified by column chromatography R_f = 0.50 (silica gel, petroleum ether:EtOAc = 19:1) to afford the target cyclopropyl ketone **1c** (517 mg, 2.66 mmol, 60% yield, 27% yield over 3 steps).

^{1}H NMR (400 MHz, $CDCl_3$) δ 2.57 – 2.44 (m, 2H, *2H-6*), 1.84 (dd, *J* = 7.5, 5.5 Hz, 1H, *H-2*), 1.80 – 1.69 (m, 2H, *H-7* + *H-8*), 1.65 – 1.46 (m, 8H, *4H-9* + *4H-10*), 1.24 (dd, *J* = 5.6, 3.9 Hz, 1H, *H-3a*), 1.19 (s, 3H, 3*H-5a*), 1.07 (s, 4H, 3*H-5b* + *H-7*), 0.80 (dd, *J* = 7.6, 3.9 Hz, 1H, *H-3b*).

^{13}C{H} NMR (101 MHz, $CDCl_3$) δ 209.3 (*C-1*), 44.6 (*C-6*), 39.9 (*C-8*), 35.2 (*C-2*), 32.7 (*C-7*), 30.5 (*2C-9*), 27.4 (*C-4*), 26.6 (*C-5a*), 25.3 (*2C-10*), 23.0 (*C-3*), 18.4 (*C-5b*).

IR (neat, cm^{-1}): 2946, 2867, 1693, 1452, 1434 (fingerprint region excluded).

HRMS (ESI+): calculated for $C_{13}H_{22}O$ (M^+H^+): 194.1671 Found: 194.1667.

1-(2,2-Dimethylcyclopropyl)-2-methylhexan-1-one (1d)

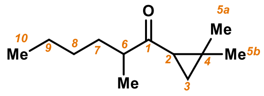

Prepared in 3 steps according to General Procedure 1 using 2-methylhexanoic acid (1.30 g, 10.0 mmol). Step Ib: The resultant crude material was purified by column chromatography R_f = 0.35 (silica gel, petroleum ether:EtOAc = 9:1) to afford the target Weinreb amide as a colorless oil (1.18 g, 6.80 mmol, 68% yield). Step II: The resultant crude material was purified by column chromatography R_f = 0.65 (silica gel, petroleum ether:Et_2O = 9:1) to afford the target enone as a yellow oil (783 mg, 4.65 mmol, 68% yield). Step IIIa: The resultant crude material was purified by column chromatography R_f = 0.60 (silica gel, petroleum ether: Et_2O = 19:1) to afford the target cyclopropyl ketone **1d** (376 mg, 2.06 mmol, 44% yield, 20% yield over 3 steps).

^{1}H NMR (400 MHz, $CDCl_3$) δ 2.58 (ddt, *J* = 10.3, 6.9, 3.4 Hz, 1H, *H-6*), 1.85 (ddd, *J* = 7.4, 5.6, 1.6 Hz, 1H, *H-2*), 1.68 (dddd, *J* = 14.7, 7.9, 4.6, 2.6 Hz, 1H, *H-7a*), 1.36 – 1.22 (m, 6H, *H-3b + H-7b + 2H-8 + 2H-9)*, 1.20 (s, 3H, *H-5a*), 1.11 – 1.02 (m, 6H), 0.89 (td, *J* = 7.1, 2.4 Hz, 3H), 0.80 (dd, *J* = 7.6, 3.9 Hz, 1H, *H-3a*).

^{13}C{H} NMR (101 MHz, $CDCl_3$) δ 212.4 (*C-1*), 212.3 (*C-1*), 48.0 (*C-6*), 47.9 (*C-6*), 34.3, 34.0, 32.9, 32.6, 29.8, 29.6, 27.3, 26.8, 26.7, 23.0 (*C-8 or C-9*), 22.9 (*C-8 or C-9*), 22.9 (*C-3*), 22.8 (*C-3*), 18.5, 18.4, 16.4, 15.9, 14.1, 14.1.

IR (neat, cm^{-1}): 2958, 2930, 2873, 1692, 1459, 1434 (fingerprint region excluded).

HRMS (ESI+): calculated for $C_{12}H_{22}O$ (M^+H^+): 182.1671 Found: 182.1664.

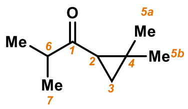

1-(2,2-Dimethylcyclopropyl)-2-methylpropan-1-one (1e)

Prepared in 3 steps according to General Procedure 1 using isobutyryl chloride (1.05 mL, 10.0 mmol). Step Ia: Weinreb amide obtained as a pale-yellow oil (1.07 g, 8.12 mmol, 81% yield). Step II: The resultant crude material was purified by column chromatography R_f = 0.25 (silica gel, petroleum ether:Et_2O = 19:1) to afford the target enone as a yellow oil (520 mg, 4.12 mmol, 51% yield). Step III: The resultant crude material was purified by column chromatography R_f = 0.60 (silica gel, petroleum ether: Et_2O = 19:1) to afford the target cyclopropyl ketone **1e** (579 mg, 4.12 mmol, 99% yield, 41% yield over 3 steps).

^{1}H NMR (500 MHz, $CDCl_3$) δ 2.68 (hept, *J* = 7.0 Hz, 1H, *H-6*), 1.86 (dd, *J* = 7.5, 5.6 Hz, 1H, *H-2*), 1.24 (dd, *J* = 5.6, 4.0 Hz, 1H, *H-3a*), 1.20 (s, 3H, *3H-5a*), 1.11 (d, *J* = 7.1 Hz, 3H, *3H-7a*), 1.09 (d, *J* = 6.8 Hz, 3H, *3H-7b*), 1.03 (s, 3H, *3H-5b*), 0.79 (dd, *J* = 7.6, 3.9 Hz, 1H, *H-3b*).

^{13}C{H} NMR (126 MHz, $CDCl_3$) δ 212.2 (*C-1*), 42.3 (*C-6*), 33.9 (*C-2*), 27.3 (*C-7*), 26.5 (*C-4*), 22.7 (*C-7*), 18.5 (*C-3*), 18.3 (*C-5a*), 17.8 (*C-5b*).

IR (neat, cm^{-1}): 2995, 2948, 2869, 1689, 1451, 1433 (fingerprint region excluded).

HRMS (APCI+): calculated for $C_9H_{17}O$ (M^+H^+): 141.1274 Found: 141.1271.

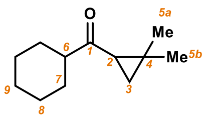

Cyclohexyl(2,2-dimethylcyclopropyl)methanone (1f)

Prepared in 3 steps according to General Procedure 1 using cyclohexanecarbonyl chloride (1.46 g, 10.0 mmol). Step Ia: Weinreb amide as obtained as a yellow oil (1.42 g, 9.01 mmol, 90% yield). Step II: The resultant

crude material was purified by column chromatography R_f = 0.30 (silica gel, petroleum ether:Et_2O = 19:1) to afford the target enone as a yellow oil (966 mg, 6.35 mmol, 70% yield). Step III: The resultant crude material was purified by column chromatography R_f = 0.40 (silica gel, petroleum ether: Et_2O = 97:3) to afford the target product to afford the target cyclopropyl ketone **1f** (1.01 mg, 5.57 mmol, 82% yield, 56% yield over 3 steps).

^{1}H NMR (500 MHz, $CDCl_3$) δ 2.42 (tt, *J* = 11.3, 3.4 Hz, 1H, *H-6*), 1.96 – 1.64 (m, 3H, *H-2* + *2H-7*), 1.42 – 1.22 (m, 8H, *2H-7* + *4H-8* + *2H-9*), 1.22 – 1.17 (m, 4H, *H-3a* + *3H-5a*), 1.02 (s, 3H, *3H-5b*), 0.77 (dd, *J* = 7.6, 3.9 Hz, 1H, *H-3b*).

^{13}C{H} NMR (126 MHz, $CDCl_3$) δ 211.6 (*C-1*), 52.3 (*C-6*), 34.3 (*C-2*), 28.6 (*C-7a*), 28.1 (*C-7b*), 27.3 (*C-5a*), 26.6 (*C-4*), 26.2 (*C-8a*), 26.1 (*C-8b*), 25.7 (*C-9*), 22.6 (*C-3*), 18.6 (*C-5b*).

IR (neat, cm^{-1}): 2927, 2853, 1688, 1449 (fingerprint region excluded).

HRMS (APCI+): calculated for $C_{12}H_{21}O$ (M^+H^+): 181.1587 Found: 181.1582.

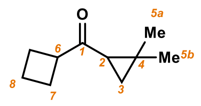

Cyclobutyl(2,2-dimethylcyclopropyl)methanone (1g)

Prepared in 3 steps according to General Procedure 1 using cyclobutanecarboxylic acid (1.00 g, 10.0 mmol). Step Ib: Weinreb amide obtained as an orange oil (1.19 g, 8.32 mmol, 83% yield). Step II: The resultant crude material was purified by column chromatography R_f = 0.50 (silica gel, petroleum ether:Et_2O = 9:1) to afford the target enone as a yellow oil (545 mg, 3.94 mmol, 47% yield). Step III: The resultant crude material was purified by column chromatography R_f = 0.65 (silica gel, petroleum ether: Et_2O = 9:1) to afford the target product to afford the target cyclopropyl ketone **1g** (260 mg, 1.71 mmol, 43% yield, 17% yield over 3 steps).

^{1}H NMR (500 MHz, $CDCl_3$) δ 3.37 – 3.27 (m, 1H, *H-6*), 2.32 – 2.22 (m, 1H, *H-2*), 2.19 – 2.10 (m, 4H, 2*H-7a* + *2H-7b*), 2.02 – 1.86 (m, 1H, *H-8a*), 1.84 – 1.77 (m, 1H, *H-8b*), 1.74 (dd, *J* = 7.6, 5.6 Hz, 1H, *H-2*), 1.25 (dd, *J* = 5.7, 3.9 Hz, 1H, *H-3a*), 1.18 (s, 3H, *3H-5a*), 1.08 (s, 3H, *3H-5b*), 0.80 (dd, *J* = 7.6, 3.9 Hz, 1H, *H-3b*).

^{13}C{H} NMR (126 MHz, $CDCl_3$) δ 209.8 (*C-1*), 47.1 (*C-6*), 33.3 (*C-2*), 27.3 (*C-5a*), 26.5 (*C-4*), 24.3 (*C-7a*), 24.3 (*C-7b*), 22.9 (*C-3*), 18.5 (*C-5b*), 17.8 (*C-6*).

IR (neat, cm^{-1}): 2944, 2868, 1689, 1624, 1434 (fingerprint region excluded).

HRMS (ESI+): calculated for $C_{10}H_{16}ONa$ (M^+Na^+): 175.1093 Found: 175.1089.

Cyclopentyl(2,2-dimethylcyclopropyl)methanone (1h)

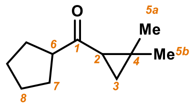

Prepared in 3 steps according to General Procedure 1 using cyclopentanecarbonyl chloride (1.33 g, 10.0 mmol). Step Ia: Weinreb amide obtained as a yellow oil (1.51 g, 8.80 mmol, 88% yield). Step II: The resultant crude material was purified by column chromatography R_f = 0.40 (silica gel, petroleum ether:Et_2O = 9:1) to afford the target enone as a yellow oil (1.13 g, 6.80 mmol, 77% yield). Step III: The resultant crude material was purified by column chromatography R_f = 0.40 (silica gel, petroleum ether: Et_2O = 97:3) to afford the target cyclopropyl ketone **1h** (711 mg, 4.27 mmol, 67% yield, 43% yield over 3 steps).

^{1}H NMR (500 MHz, $CDCl_3$) δ 2.95 (tt, *J* = 8.7, 7.3 Hz, 1H, *H-6*), 1.84 (dd, *J* = 7.6, 5.5 Hz, 1H, *H-2*), 1.92 – 1.50 (m, 8H, *4H-7* + *4H-8*), 1.24 (dd, *J* = 5.6, 3.9 Hz, 1H, *H-3a*), 1.19 (s, 3H, *3H-5a*), 1.04 (s, 3H, *3H-5b*), 0.79 (dd, *J* = 7.6, 3.9 Hz, 1H, *H-3b*).

^{13}C{H} NMR (126 MHz, $CDCl_3$) δ 210.8 (*C-1*), 53.2 (*C-6*), 34.7 (*C-2*), 29.0 (*C-7a*), 28.1 (*C-7b*), 27.3 (*C-5a*), 26.5 (*C-4*), 26.1 (*C-8a*), 26.0 (*C-8b*), 22.9 (*C-3*), 18.5 (*C-5b*).

IR (neat, cm^{-1}): 2967, 2874, 1692, 1466, 1434 (fingerprint region excluded).

HRMS (APCI+): calculated for $C_{11}H_{19}O$ (M^+H^+): 167.1430 Found: 167.1426.

Cycloheptyl(2,2-dimethylcyclopropyl)methanone (1i)

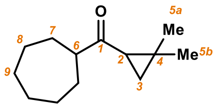

Prepared in 3 steps according to General Procedure 1 using cyclohexanecarboxylic acid (994 mg, 7.00 mmol). Step Ib: Weinreb amide obtained as a yellow oil (1.30 g, 7.00 mmol, >99% yield). Step II: The resultant crude material was purified by column chromatography R_f = 0.50 (silica gel, petroleum ether:Et_2O = 9:1) to afford the target enone as a yellow oil (627 mg, 3.47 mmol, 49% yield). Step III: The resultant crude material was purified by column chromatography R_f = 0.65 (silica gel, petroleum ether: Et_2O = 19:1) to afford the target cyclopropyl ketone **1i** (627 mg, 3.22 mmol, 97% yield, 32% yield over 3 steps).

^{1}H NMR (500 MHz, $CDCl_3$) δ 2.59 (tt, *J* = 9.6, 4.2 Hz, 1H, *H-6*), 1.97 – 1.79 (m, 3H, 2*H-7* + *H-2*),1.76 – 1.39 (m, 10H, *2H-7* + *4H-8* + *4H-9*), 1.22 (dd, *J* = 5.6, 3.9 Hz, 1H, *H-3a*), 1.19 (s, 3H, *3H-5a*), 1.04 (s, 3H, *3H-5b*), 0.78 (dd, *J* = 7.5, 3.9 Hz, 1H, *H-3b*).

^{13}C{H} NMR (126 MHz, $CDCl_3$) δ 212.0 (*C-1*), 54.0 (*C-6*), 34.2 (*C-2*), 30.1 (*C-7a*), 29.5 (*CH_2*), 28.6 (*CH_2*), 28.4 (*CH_2*), 27.3 (*C-5a*), 27.1 (*CH_2*), 26.8 (*C-7b*), 26.5 (*C-4*), 22.8 (*C-3*), 18.4 (*C-5b*).

IR (neat, cm^{-1}): 2922, 2855, 1688, 1459, 1433 (fingerprint region excluded).

HRMS (APCI+): calculated for $C_{13}H_{23}O$ (M^+H^+): 195.1743 Found: 195.1739.

Adamantan-1-yl)(2,2-dimethylcyclopropyl)methanone (1j)

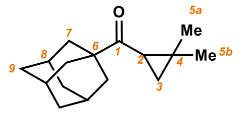

Prepared in 3 steps according to General Procedure 1 using 1-adamantane carboxylic acid (1.80 g, 10.0 mmol). Step Ib: Weinreb amide obtained as a pale yellow solid (2.32 g, 10.0 mmol, >99% yield). Step II: The reaction was run using only half the material obtained in Step I. The resultant crude material was passed through a silica pad with a petroleum ether:Et_2O = 9:1 eluent to afford the target enone as a crude mixture which was used in the next step directly (approximate yield ~20%). Step III: The resultant crude material was purified by column chromatography R_f = 0.45 (silica gel, petroleum ether: Et_2O = 19:1) to afford the target product to afford the target cyclopropyl ketone **1j** (204 mg, 0.88 mmol, 70% yield, 18% yield over 3 steps).

Note: *1j readily decomposed in weakly acidic media (deactivated silica, $CDCl_3$) hence it was required to run the ^{13}C spectrum in C_6D_6. 1j also appears to be light/temperature sensitive (colourless solid turns red on leaving at room temperature >1h) and shows signs of decomposition during acquisition of the 2D-NMR spectra.*

^{1}H NMR (400 MHz, $CDCl_3$) δ 2.13 – 1.98 (m, 4H, *H-2* + 3 x *H-8*), 1.84 – 1.57 (m, 12H *6CH₂*), 1.28 – 1.23 (m, 1H, *H-3a*), 1.20 (s, 3H, *3H-5a*), 0.96 (s, 3H, *3H-5b*), 0.74 (dd, *J* = 7.4, 3.9 Hz, 1H, *H-3b*).

^{13}C{H} NMR (126 MHz, C_6D_6) δ 210.2 (*C-1*), 46.2, 38.5, 37.0, 30.6, 28.5, 26.9 (*C-5a*), 25.4, 21.6 (*C-3*), 18.62 (*C-5b*).

IR (neat, cm^{-1}): 2901, 2848, 1673, 1450, 1429 (fingerprint region excluded).

HRMS (ESI+): calculated for $C_{16}H_{25}O$ (M^+H^+): 233.1900 Found: 233.1906.

3-Phenyl-1-(spiro[2.5]octan-1-yl)propan-1-one (1s)

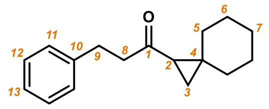

Prepared in 2 steps according to General Procedure 2 using cyclohexanone (207 µL, 2.00 mmol). The crude residue was purified by column chromatography R_f = 0.25 (silica gel, petroleum ether:Et_2O = 19:1) to afford the target cyclopropyl ketone **1s** as a colorless oil (191 mg, 0.79 mmol, 40% yield over 2 steps).

^{1}H NMR (400 MHz, $CDCl_3$) δ 7.35 – 7.21 (m, 2H, *2H-11*), 7.21 – 6.99 (m, 3H, *2H-12* + *H-13*), 2.94 – 2.89 (m, 4H, *2H-8* + *2H-9*), 1.79 (dd, *J* = 7.4, 5.5 Hz, 1H, *H-2*), 1.65 – 1.36 (m, 10H, 2*H-5* + *4H-6* + *2H-*

7), 1.28 (dd, *J* = 5.5, 4.0 Hz, 2H, *H-3a* + *H-5*), 1.19 – 1.05 (m, 1H, *H-5*), 0.80 (dd, *J* = 7.4, 4.0 Hz, 1H, *H-3b*).

^{13}C{H} NMR (101 MHz, $CDCl_3$) δ 207.5 (*C-1*), 141.4 (*C-10*), 128.4 (*C-11*), 128.3 (*C-12*), 126.0 (*C-13*), 46.2 (*C-8*), 37.7 (*CH_2*), 34.9 (*C-4*), 34.5 (*C-2*), 30.2 (*C-9*), 28.0 (*CH_2*), 26.1 (*CH_2*), 26.0 (*CH_2*), 25.9 (*CH_2*), 21.9 (*C-3*).

IR (neat, cm^{-1}): 2922, 2850, 1692, 1496, 1445 (fingerprint region excluded).

HRMS (ESI+): calculated for $C_{17}H_{22}ONa$ ($M^{+}Na^{+}$): 265.1563 Found: 265.1570.

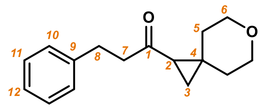

3-Phenyl-1-(6-oxaspiro[2.5]octan-1-yl)propan-1-one (1t)

Prepared in 2 steps according to General Procedure 2 using tetrahydro-4*H*-pyran-4-one (185 µL, 2.00 mmol). The crude residue was purified by column chromatography R_f = 0.20 (silica gel, petroleum ether:Et_2O = 9:1) to afford the target cyclopropyl ketone **1t** as a colorless oil (282 mg, 1.15 mmol, 58% yield over 2 steps).

^{1}H NMR (500 MHz, $CDCl_3$) δ 7.32 – 7.24 (m, 2H, *2H-10*), 7.23 – 7.14 (m, 3H, *2H-11* + *H-12*), 3.75 – 3.68 (m, 2H. *2H-6a* + *H-6b*), 3.60 (ddd, *J* = 10.9, 7.1, 3.5 Hz, 1H, *H-6a*), 3.41 (td, *J* = 7.2, 3.5 Hz, 1H, *H-6b*), 2.98 – 2.87 (m, 4H, *2H-8* + *2H-7*), 1.88 (dd, *J* = 7.6, 5.4 Hz, 1H, *H-2*), 1.71– 1.43 (m, 4H, *2H-5a* + *2H-5b*), 1.34 (dd, *J* = 5.5, 4.2 Hz, 1H, *H-3a*), 0.91 (dd, *J* = 7.6, 4.2 Hz, 1H, *H-3b*).

^{13}C{H} NMR (126 MHz, $CDCl_3$) δ 207.2 (*C-1*), 141.2 (*C-9*), 128.6 (*C-11*), 128.5 (*C-10*), 126.2 (*C-12*), 68.1 (*C-6a*), 67.5 (*C-6b*), 46.4 (*C-8*), 37.5 (*C-7*), 33.7 (*C-2*), 31.8 (*C-4*), 30.1 (*C-5a*), 28.7 (*C-5b*), 21.4 (*C-3*).

IR (neat, cm^{-1}): 3026, 2955, 2914, 2843, 1692, 1603, 1496, 1453, 1439, 1401 (fingerprint region excluded).

HRMS (ESI+): calculated for $C_{16}H_{20}O_2$ ($M^{+}H^{+}$): 245.1536 Found: 245.1536.

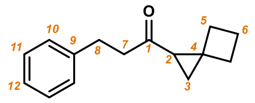

3-Phenyl-1-(spiro[2.3]hexan-1-yl)propan-1-one (1u)

Prepared in 2 steps according to General Procedure 2 using cyclobutanone (150 µL, 2.00 mmol). The crude residue was purified by column chromatography R_f = 0.35 (silica gel, petroleum ether:Et_2O = 19:1) eluent

mixture to afford the target cyclopropyl ketone **1u** as a colorless oil (135 mg, 0.63 mmol, 32% yield over 2 steps).

^{1}H NMR (500 MHz, $CDCl_3$) δ 7.36 – 7.14 (m, 5H, *2H-10* + *2H-11* + *H-12*), 3.01 – 2.78 (m, 4H, *2H-8* + *2H-7*), 2.26 – 1.77 (m, 7H, *H-2* + *4H-5* + *2-H-6*), 1.32 (dd, *J* = 5.4, 4.2 Hz, 1H, *H-3a*), 1.03 (dd, *J* = 8.1, 4.2 Hz, 1H, *H-3b*).

^{13}C{H} NMR (126 MHz, $CDCl_3$) δ 207.8 (*C-1*), 141.4 (*C-9*), 128.6 (*C-10*), 128.5 (*C-11*), 126.1 (*C-12*), 45.9 (*C-8*), 34.1 (*C-4*), 33.6 (*C-2*), 31.1 (*C-5a*), 30.1 (*C-7*), 27.7 (*C-5b*), 23.1 (*C-3*), 16.8 (*C-6*).

IR (neat, cm^{-1}): 3062, 3026, 2981, 2930, 2852, 1670, 1603, 1496, 1453, 1429 (fingerprint region excluded).

HRMS (APCI+): calculated for $C_{15}H_{19}O$ (M^+H^+): 215.1436 Found: 215.1428.

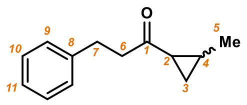

1 1-(2-Methylcyclopropyl)-3-phenylpropan-1-one (1v)

Prepared in 3 steps according to General Procedure 1 using hydrocinnamic acid (1.50 g, 10.0 mmol). Step Ib: Weinreb amide obtained as a pale-yellow oil (1.80 g, 9.33 mmol, 93% yield). Step II: Using freshly prepared 1-propenylmagnesium bromide solution in THF (1.2 eq), see section 1.2. The resultant crude material was purified by column chromatography R_f = 0.60 (silica gel, petroleum ether:EtOAc = 9:1) to afford the target enone as a pale-yellow oil and a mixture of diastereoisomers (550 mg, 3.16 mmol, 63% yield). Step III: The resultant crude material was purified by column chromatography R_f = 0.50 (silica gel, petroleum ether:Et_2O = 9:1) to afford the target cyclopropyl ketone **1v** (360 mg, 1.91 mmol, 60% yield, 38% yield over 3 steps).

^{1}H NMR (400 MHz, $CDCl_3$) δ 7.33 – 7.25 (m, 2H, *2H-9*), 7.21 – 7.18 (m, 3H, *2H-10* + *H-11*), 2.95 – 2.89 (m, 2H, *2H-7*), 2.88 – 2.82 (m, 2H, *2H-6*), 1.64 (dt, *J* = 8.1, 4.3 Hz, 1H, *H-2*), 1.39 (dtd, *J* = 8.6, 6.2, 3.9 Hz, 1H, *H-4*), 1.24 (ddd, *J* = 8.4, 4.6, 3.6 Hz, 1H, *H-3a*), 1.09 (d, *J* = 6.0 Hz, 3H, *3H-5*), 0.70 (ddd, *J* = 7.8, 6.4, 3.6 Hz, 1H, *H-3b*).

^{13}C{H} NMR (101 MHz, $CDCl_3$) δ 209.6 (*C-1*), 141.4 (*C-8*), 128.6 (*C-9*), 128.5 (*C-10*), 126.2 (*C-11*), 45.2 (*C-6*), 30.2 (*C-7*), 29.9 (*C-2*), 20.2 (*C-4*), 19.5 (*C-3*), 18.2 (*C-5*).

IR (neat, cm^{-1}) 3027, 2956, 2928, 2867, 1692, 1496, 1453, 1402 (fingerprint region excluded).

HRMS (APCI+): calculated for $C_{13}H_{17}O$ (M^+H^+): 189.1274 Found: 189.1267.

Synthesis of cyclopropyl ketone substrates 1w and 1x

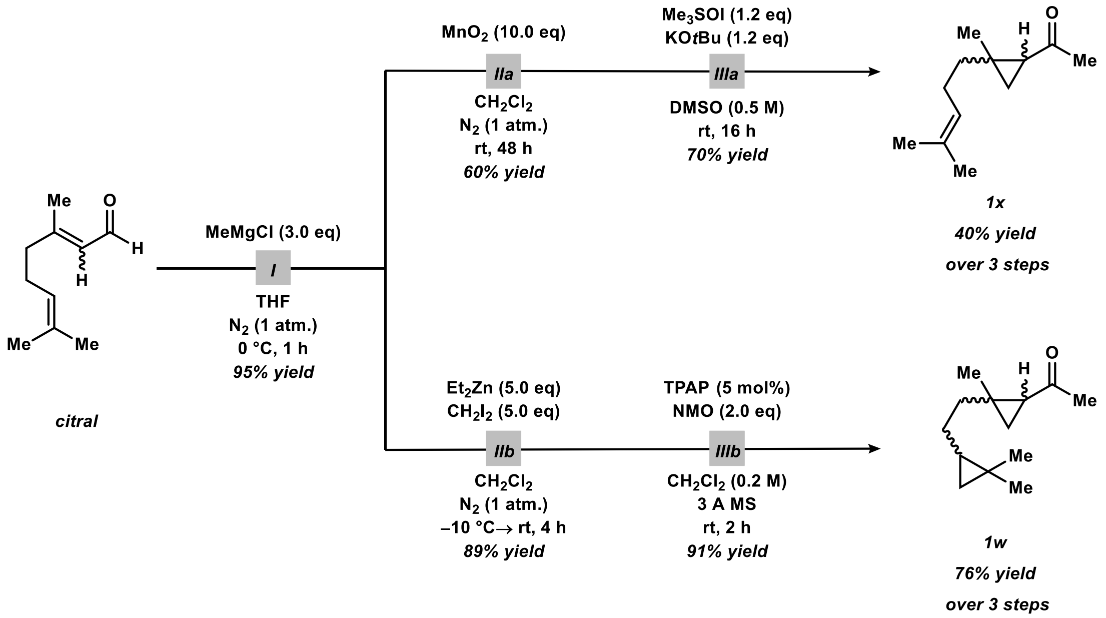

Step I: Procedure adapted from Kobayashi and co-workers [15]

A solution of citral (4.20 mL, 95% purity, 21.0 mmol, 1.0 eq) in THF (60 mL) was cooled (0 °C, 15 min) and MeMgCl (14.5 mL, 3.0 M in THF, 42.0 mmol, 2.0 eq) was added dropwise. The reaction mixture was stirred with cooling (0 °C, 1 h). The reaction mixture was quenched by addition of saturated aqueous NH_4Cl (20 mL). The phases were separated, and the aqueous phase was washed with EtOAc (3 x 60 mL). The combined organic phase was washed with saturated aqueous NaCl (20 mL), dried over $MgSO_4$, and evaporated *in vacuo*. The crude residue was purified by column chromatography R_f = 0.20 (silica gel, petroleum ether:EtOAc = 9:1) to afford the target alcohol **SI-10** as a colorless oil (3.10 g, 19 mmol, >95% yield) and an inseparable mixture of double bond isomers.

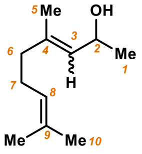

4,8-Dimethylnona-3,7-dien-2-ol (SI-10)

^{1}H NMR (400 MHz, $CDCl_3$) δ 5.27 – 5.18 (m, 1H, *H-3*), 5.15 – 5.04 (m, 1H, *H-8*), 4.63 – 4.47 (m, 1H, *H-2*), 2.14 – 1.95 (m, 4H, *2H-6* + *2H-7*), 1.74 – 1.66 (m, 6H, *3H-5* + *3H-10b*), 1.60 (d, *J* = 1.9 Hz, 3H, *3H-10a*), 1.22 (d, *J* = 6.3 Hz, 3H, *3H-1*).

^{13}C{H} NMR (101 MHz, $CDCl_3$) δ 137.9 (*C-4 Z-isomer*), 137.8 (*C-4 E-isomer*) 132.7 (*C-1 Z-isomer*), 131.9 (*C-1 E-isomer*), 130.4 (*C-3 Z-isomer*), 129.3 (*C-3 E-isomer*), 124.1 (*C-8*), 64.9 (*C-2 E-isomer*) , 64.4 (*C-2 Z-isomer*), 39.6 (*C-6 E-isomer*), 32.4 (*C-6 Z-isomer*), 26.6 (*C-7*), 25.8 (*C-10a*), 23.6 (*C-10b*), 23.4 (*C-5 Z-isomer*), 17.8 (*C-5 E-isomer*), 17.8 (*C-1 Z-isomer*), 16.6 (*C-1 E-isomer*).

HRMS (ESI+): calculated for $C_{11}H_{20}ONa$ ($M^{+}Na^{+}$): 191.1406 Found: 191.1401.

Data in accordance with those previously reported.[16]

Step IIa: Procedure adapted from Procter and co-workers [6]

To a solution of **SI-10** (~2.0 g, 1.0 mmol, 1.0 eq) in CH_2Cl_2 (30 mL) was added MnO_2 (8.9 g, 10.0 mmol, 10 eq). The reaction mixture was stirred vigorously until TLC indicated all the starting material had been consumed (rt, 48 h). The reaction mixture was filtered through a Celite pad with EtOAc (100 mL) as the eluent and the filtrate was evaporated *in vacuo*. The crude residue was purified by column chromatography R_f = 0.30 (silica gel, petroleum ether:EtOAc = 9:1) to afford the target enone **SI-11** as a yellow oil (985 mg, 6.0 mmol, 60% yield) as an inseparable mixture of double bond isomers.

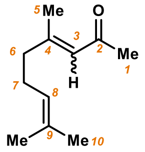

4,8-Dimethylnona-3,7-dien-2-one (SI-11)

^{1}H NMR (400 MHz, $CDCl_3$) δ 6.08 – 6.03 (m, 1H, *H-2 both isomers*), 5.17 – 5.03 (m, 1H, *H-8 both isomers*), 2.62 – 2.54 (m, 1H, *2H-7 Z-isomer*), 2.22 – 2.08 (m, 8H, *2H-7 E-isomer + 2H-8 Z-isomer + 2H-8 E-isomer + 3H-5 E-isomer + 3H-1 both isomers*), 1.87 (d, *J* = 1.3 Hz, 1H, *3H-5 Z-isomer*), 1.68 (dd, *J* = 3.6, 1.5 Hz, 3H, *3H-10a both isomers*), 1.62 (dd, *J* = 8.2, 1.3 Hz, 3H, *3H-10b both isomers*).

^{13}C{H} NMR (101 MHz, $CDCl_3$) δ 199.0 (*C-2 E-isomer*), 198.4 (*C-2 Z-isomer*), 159.0 (*C-4 Z-isomer*), 158.5 (*C-4 E-isomer*), 132.7 (*C-9 E-isomer*), 132.3 (*C-9 Z-isomer*), 124.4 (*C-8 Z-isomer*), 123.9 (*C-8 E-isomer*) , 123.8 (*C-3 Z-isomer*), 123.2 (*C-3 E-isomer*), 41.3 (*C-7 E-isomer*), 33.9 (*C-7 Z-isomer*), 31.94 (*C-1 E-isomer*), 31.86 (*C-1 Z-isomer*), 26.9 (*C-6 Z-isomer*), 26.3 (*C-6 E-isomer*), 25.84 (*CH_3*), 25.82 (*CH_3*), 25.7 (*CH_3*), 19.4 (*CH_3*), 17.84 (*CH_3*), 17.75 (*CH_3*).

HRMS (ESI+): calculated for $C_{11}H_{18}ONa$ ($M^{+}Na^{+}$): 191.1406 Found: 191.1401.

Data in accordance with those previously reported.[17]

Step IIIa: Using General Procedure 1 Step III

Modified preparation involved **SI-11** (665 mg, 4.00 mmol, 1.0 eq), K*t*-BuO (449 mg, 4.00 mmol, 1.0 eq), and trimethylsulfoxonium iodide (880 mg, 4.00 mmol, 1.0 eq). The crude residue was purified by column chromatography R_f = 0.35 (silica gel, petroleum ether:Et_2O = 9:1) to afford the target product **1x** as a yellow oil and as an inseparable mixture of diastereoisomers (470 mg, 2.80 mmol, 70% yield, d.r. = 1:1).

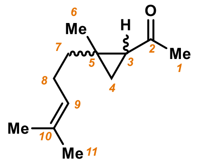

1-(2-Methyl-2-(4-methylpent-3-en-1-yl)cyclopropyl)ethan-1-one (1x)

^{1}H NMR (500 MHz, $CDCl_3$) δ 5.14 – 4.99 (m, 1H), 2.26 – 2.21 (m, 3H, CH_3), 2.19 – 2.06 (m, 2H), 1.71 – 1.64 (m, 3H), 1.62 – 1.58 (m, 3H), 1.56 – 1.38 (m, 2H), 1.24 (ddd, *J* = 22.9, 5.6, 4.1 Hz, 2H), 1.19 (s, 3H, CH_3), 1.07 (s, 3H, CH_3), 0.81 (ddd, *J* = 7.0, 4.1, 2.5 Hz, 1H, *H-4*).

^{13}C{H} NMR (126 MHz, $CDCl_3$) δ 206.7 (*C-2*), 206.6 (*C-2*), 131.9 (*C-10*), 131.6 (*C-10*), 124.3 (*C-9*), 124.1 (*C-9*), 41.4, 35.7, 35.3, 32.4, 32.1, 31.5 (*C-1*), 31.2 (*C-1*), 30.5, 26.0, 25.9, 25.8, 25.6, 24.5, 23.3, 22.4, 17.7, 17.6, 15.4.

IR (neat, cm^{-1}): 2964, 2916, 2856, 1695, 1617, 1437 (fingerprint region excluded).

HRMS (ESI+): calculated for $C_{11}H_{20}ONa$ (M^+Na^+): 191.1406 Found: 191.1401.

Step IIb: Procedure adapted from Charette and co-workers [18]

A solution of **SI-10** (673 mg, 4.00 mmol, 1.0 eq) in CH_2Cl_2 (40 mL) was cooled (–10 °C, 15 min) and Et_2Zn (20.0 mL, 1.0 M, 5.0 eq) was added drop-wise followed by CH_2I_2 (1.62 mL, 20.0 mmol, 5.0 eq). The reaction mixture was stirred with gradual warming (–10 °C → rt, 3 h) then stirred at this temperature (rt, 1h). Saturated aqueous NH_4Cl (20 mL) was added, and the mixture was diluted with Et_2O (160 mL) and 10% aqueous HCl (1N, 20 mL). The phases were separated, and the organic phase was then successively washed with saturated aqueous Na_2SO_3 (40 mL), saturated aqueous $NaHCO_3$ (40 mL) and saturated aqueous NaCl (40 mL). The organic phase was dried over anhydrous $MgSO_4$ and evaporated *in vacuo*. The crude residue was purified by column chromatography R_f = 0.25 (silica gel, petroleum ether:EtOAc = 9:1) to afford the target cyclopropyl alcohol **SI-12** as a colorless oil and as a mixture of diastereoisomers (689 mg, 3.55 mmol, 89% yield, d.r. = 1.3:1).

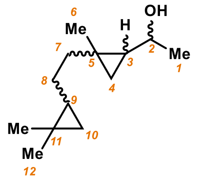

1-(2-(2-(2,2-dimethylcyclopropyl)ethyl)-2-methylcyclopropyl)ethan-1-ol (SI-12)

^{1}H NMR (500 MHz, $CDCl_3$) δ 3.47 – 3.31 (m, 1H), 1.52 – 1.17 (m, 7H), 1.07 – 0.94 (m, 9H), 0.71 – 0.60 (m, 1H), 0.54 – 0.38 (m, 2H), 0.34 (td, *J* = 8.3, 4.1 Hz, 1H), 0.28 – 0.20 (m, 1H), -0.11 – -0.19 (m, 1H).

^{13}C{H} NMR (126 MHz, $CDCl_3$) δ 69.8, 69.3, 41.9, 41.8, 34.7, 33.9, 33.8, 32.2, 27.6, 27.6, 27.33, 27.31, 26.9, 26.9, 24.8, 24.8, 24.6, 24.5, 23.9, 23.63, 23.60, 20.8, 20.6, 20.2, 19.84, 19.8, 19.7, 19.6, 18.3, 18.3, 17.7, 17.7, 17.6, 17.6, 15.5, 15.4, 15.4.

IR (neat, cm^{-1}): 3346, 3055, 2970, 2923, 2866, 1456, 1412 (fingerprint region excluded).

HRMS (ESI+): +): calculated for $C_{13}H_{23}O$ (M^+H^+): 195.1743 Found: 195.1739.

Step IIIb: Procedure adapted from Hodgson and co-workers [4]

TPAP (68 mg, 0.2 mmol, 5 mol%) was added to a stirred solution of XX (689 mg, 3.55 mmol, 1.0 eq), 4-methylmorpholine *N*-oxide (832 mg, 7.10 mmol, 2.0 eq), and powdered 3Å molecular sieves (~1.0 g) in CH_2Cl_2 (30 mL) and the mixture stirred (rt, 2 h). The mixture was filtered through a short pad of silica (2.5 cm × 8 cm) eluting with Et_2O (100 mL). The filtrate was concentrated *in vacuo* and the crude residue was purified by column chromatography R_f = 0.20 (silica gel, petroleum ether:Et_2O = 9:1) to afford the target cyclopropyl ketone **1w** as a colorless oil and as a mixture of diastereoisomers (374 mg, 1.81 mmol, 91% yield, 86% yield over 3 steps, d.r. = 1.1:1).

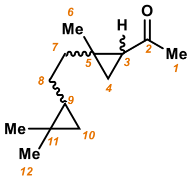

1-(2-(2-(2,2-Dimethylcyclopropyl)ethyl)-2-methylcyclopropyl)ethan-1-one (1w)

^{1}H NMR (400 MHz, $CDCl_3$) δ 2.24 (m, 3H), 1.90 – 1.79 (m, 1H), 1.72 – 1.11 (m, 6H), 1.10 – 0.97 (m, 8H), 0.81 (ddd, *J* = 7.5, 6.2, 4.0 Hz, 1H), 0.50 – 0.28 (m, 2H), -0.09 – -0.22 (m, 1H).

^{13}C{H} NMR (101 MHz, $CDCl_3$) δ 206.7, 206.7, 206.5, 206.5, 41.9, 41.8, 35.8, 35.7, 35.3, 35.3, 32.4, 32.2, 32.2, 32.0, 31.9, 31.5, 31.3, 30.8, 30.7, 27.8, 27.8, 27.8, 27.7, 27.3, 27.2, 24.7, 24.6, 24.6, 24.6, 23.4, 23.2, 22.6, 19.9, 19.9, 19.9, 19.8, 19.8, 15.6, 15.6, 15.5, 15.5, 15.4.

IR (neat, cm^{-1}): 3924, 2864, 1696, 1455, 1390 (fingerprint region excluded).

HRMS (ESI+): calculated for $C_{13}H_{23}O$ (M^+H^+): 195.1743 Found: 195.1735.

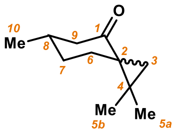

1,1,6-Trimethylspiro[2.5]octan-4-one (1y)

Prepared in 1 step according to General Procedure 1 step III using purified pulegone (761 mg, 5.00 mmol). The resultant crude material was purified by column chromatography R_f = 0.60 (silica gel, petroleum ether: Et_2O = 19:1) to afford the target product to afford the target cyclopropyl ketone **1y** a colorless oil (707 mg, 4.25 mmol, 85% yield, d.r. = 2.4:1.).

^{1}H NMR (400 MHz, $CDCl_3$) δ 2.50 (ddd, *J* = 14.5, 4.2, 2.2 Hz, 1H, *H-9a minor*), 2.32 – 2.14 (m, 6H, *2H-9 major + H-8 major*), 2.10 – 1.97 (m, 3H), 1.96 – 1.78 (m, 5H), 1.75 – 1.57 (m, 5H), 1.55 – 1.45 (m,

2H), 1.42 – 1.35 (m, 3H), 1.35 – 1.15 (m, 12H), 1.04 – 1.00 (m, 10H), 0.97 – 0.95 (m, 7H), 0.93 (s, 3H, *3H-10 minor*), 0.28 (d, *J* = 4.3 Hz, 2H, *H-3a major*), 0.23 (d, *J* = 4.4 Hz, 1H, *H-3a minor*).

^{13}C{H} NMR (126 MHz, $CDCl_3$) δ 211.4 (*C-1 major*), 210.1 (*C-1 minor*), 51.5 (*C-9 minor*), 49.1 (*C-9 major*), 38.7 (*C-2 major*), 38.6 (*C-2 minor*), 33.9 (*C-6 minor*), 33.7 (*C-8 minor*), 30.64 (*C-7 minor*), 30.55 (*C-6 major*), 29.7 (*C-8 major*), 26.9 (*C-4 major*), 26.3 (*C-7 major*), 26.0 (*C-4 minor*), 24.5 (*C-3 major*), 23.05 (*C-3 minor*), 22.7 (*C-5a minor*), 21.3 (*C-5a major*), 20.9 (*C-5b minor*), 20.7 (*C-10 minor*), 20.6 (*C-5b major*), 20.1 (*C-10 major*).

IR (neat, cm^{-1}): 2987, 2925, 2871, 1695, 1456, 1417 (fingerprint region excluded).

HRMS (ESI+): calculated for $C_{11}H_{19}O$ (M^+H^+): 167.1430 Found: 167.1428.

Synthesis of 4,7,7-trimethylbicyclo[4.1.0]heptan-2-one (3a)

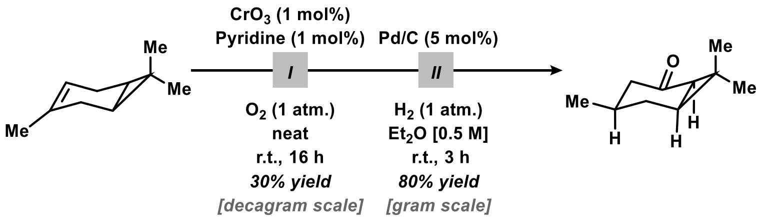

Procedures adapted from Kocovsky and co-workers**:** [19]

Step I: The reaction was generally run in triplicate to maximise O_2 diffusion. A flask was charged with 3-carene (7.00 mL, 40 mmol, 90% purity) and pyridine (35 µL, 0.4 mmol, 1 mol%), and the resultant mixture was stirred (rt, 1 min). Chromium(VI) oxide (40 mg, 0.4 mmol, 1 mol%) was added in one portion with vigorous stirring and O_2 was bubbled through the solution (1 balloon, 30 min). The colorless mixture turned violet, and the reaction mixture was stirred with a new O_2 balloon (rt, 16 h). The reaction mixture was quenched by addition of CH_2Cl_2:H_2O (1:1, 50 mL) and the phases separated. The aqueous phase was extracted with CH_2Cl_2 (3 × 70 mL). The combined organic phase was washed with a sat. NaCl solution (20 mL), dried over anhydrous $MgSO_4$ and concentrated *in vacuo*. The crude residue was purified by column chromatography R_f = 0.20 (silica gel, petroleum ether:EtOAc = 17:3) to afford the target enone **SI-13** as a pale-yellow oil (1.80 g, 12 mmol, 30% yield). Typically, co-elution of target enone and the over oxidised product occurred, and these mixed fractions were concentrated separately and used in the next step directly and accounted for ~30% yield of product.

4,7,7-Trimethylbicyclo[4.1.0]hept-3-en-2-one (SI-13)

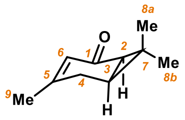

^{1}H NMR (400 MHz, $CDCl_3$) δ 5.82 (s, 1H, *H-6*), 2.63 (dddd, *J* = 20.8, 8.2, 2.1, 1.1 Hz, 1H, *H-4a*), 2.32 (d, *J* = 21.0 Hz, 1H *H-4b*), 1.87 (d, *J* = 1.1 Hz, 3H, *3H-9*), 1.58 - 1.40 (m, 2H, *H-2* + *H-3*), 1.18 (s, 3H, *3H-8a*), 1.04 (s, 3H, *3H-8b*).

^{13}C{H} NMR (126 MHz, $CDCl_3$) δ 196.8 (*C-1*), 159.0 (*C-5*), 126.6 (*C-6*), 33.0 (*C-2*), 28.6 (*C-3*), 28.0 (*C-9*), 26.0 (*C-4*), 23.8 (*C-8a*), 22.7 (*C-7*), 14.5 (*C-8b*).

Data in accordance with those previously reported.[20]

Step II. A solution of **SI-13** (3.00 g, 20 mmol) in a Et_2O (40 mL) was stirred vigorously under a pressure of 1 bar of hydrogen, in the presence of palladium on charcoal (10 wt%, 1.00 g, 5 mol%) with TLC monitoring (rt, 3 h). The catalyst was removed by filtering through a Celite pad, and the solvent was removed *in vacuo*. The crude residue was purified by column chromatography R_f = 0.40 (silica gel, petroleum ether:EtOAc = 9:1) to afford the target ketone **3a** as a colorless oil (2.43 g, 16 mmol, 80% yield).

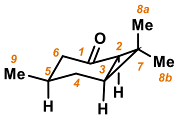

^{1}H NMR (500 MHz, $CDCl_3$) δ 2.24 (dddd, *J* = 16.8, 4.0, 2.8, 1.6 Hz, 1H**,** *H-6a*), 2.06 (dddd, *J* = 14.2, 9.5, 4.7, 2.7 Hz, 1H, *H-4a*), 2.00 - 1.87 (m, 1H, *H-5*), 1.66 - 1.50 (m, 2H**,** *H-6b, H-3*), 1.41 (d, *J* = 7.6 Hz, 1H, *H-2*), 1.17 – 1.13 (m, 6H, *3H-8a, 3H-8b*), 1.14 - 1.08 (m, 1H, *H-4b*), 0.95 (d, *J* = 6.5 Hz, 3H, 3*H-9*).

^{13}C{H} NMR (126 MHz, $CDCl_3$) δ 210.4 (*C-1*), 48.5 (*C-6*), 34.8 (*C-2*), 34.3 (*C-3*), 30.8 (*C-5*), 29.3 (*C-9*), 28.0 (*C-7)*, 27.8 (*C-4*), 21.3 (*C-8a*), 16.8 (*C-8b*).

Data were in accordance with those previously reported.[19]

7,7-Dimethylbicyclo[4.1.0]heptan-2-one (3x)

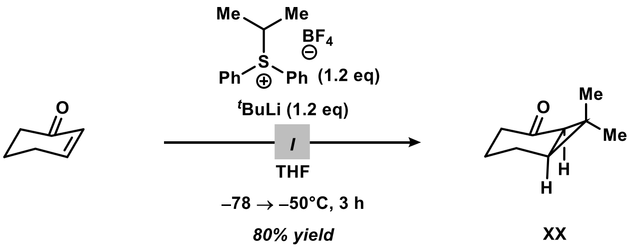

Procedure adapted from Corey and co-workers**:** [21]

To an oven-dried flask was added diphenylisopropylsulfonium tetrafluoroborate **SI-3** (500 mg, 1.58 mmol, 1.2 eq) and the flask was flushed with N_2 (15 min). Freshly distilled THF (16 mL) was added, and the resultant suspension was cooled (–78 ºC, 15 min) then ^{t}BuLi (930 µL, 1.5 M in hexanes, 1.58 mmol,

1.2 eq) was added dropwise to the cooled solution. To the resultant orange solution was added cyclohexenone (128 µL, 1.31 mmol, 1.0 eq) and the mixture was gradually warmed (–78 ºC to –50 ºC, 120 min) and stirred until the orange colour had faded completely (60 min). H_2O (5 mL) was added, and the mixture left to warm gradually (–50 ºC to rt, 30 min) followed by addition of Et_2O (20 mL) , the phases were separated, and the aqueous phase was extracted with Et_2O (3 x 20 mL). The combined organic phase was washed with a saturated aqueous NaCl solution (20 mL), dried over anhydrous $MgSO_4$ and concentrated *in vacuo*. The crude residue was purified by column chromatography R_f = 0.20 (silica gel, pentane:Et_2O = 4:1) to afford the target ketone **3x** as a colorless oil (150 mg, 1.0 mmol, 80% yield).

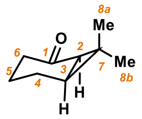

^{1}H NMR (400 MHz, $CDCl_3$) δ 2.29 (dtd, *J* = 15.8, 3.2, 1.7 Hz, 1H, *H-6a*), 2.13 – 2.01 (m, 1H, *H-4a*), 1.97 – 1.81 (m, 2H, *H-5a* + *H-6b*), 1.73 (ddt, *J* = 13.4, 12.3, 4.7 Hz, 1H, *H-5b*), 1.62 – 1.39 (m, 3H, *H-2* + *H-3* + *H-4b*), 1.18 – 1.14 (m, 6H, *3H-8a* + *3H-8b*).

^{13}C{H} NMR (126 MHz, $CDCl_3$) δ 210.3 (*C-1*), 40.2 (*C-6*), 34.7 (*C-2*), 31.1 (*C-3*), 29.4 (*C-8a*), 28.0 (*C-7*), 26.0 (*C-5*), 19.0 (*C-4*), 16.9 (*C-8b*).

IR (neat, cm^{-1}): 2997, 2942, 2867, 1676, 1453, 1420 (fingerprint region excluded).

HRMS (ESI$^+$): calculated for $C_9H_{15}O$ (M^+H^+): 139.1117 Found: 139.1115.

General Procedure 2: Synthesis of fused cyclopropyl ketones by 1,4-nucleophilic addition

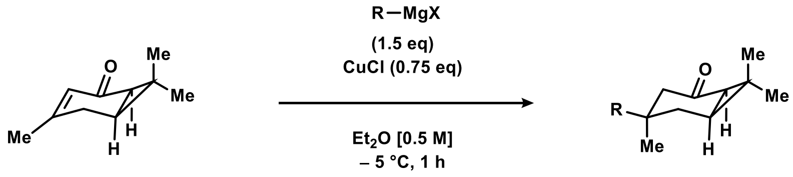

Procedure adapted from Kalvinsh and co-workers: [22]

To an oven-dried flask was added a solution of the corresponding alkylmagnesium halide (7.5 mmol, 1.5 eq) in dry Et_2O (1.0 M) and the mixture was cooled (–5 °C, 5 min). To the solution was added anhydrous CuCl (3.75 mmol, 0.75 eq) in one portion under a strong positive pressure of N_2, and the mixture was stirred vigorously (–5 °C, 5 min). Then, **SI-13** (5 mmol, 1.0 eq) in dry Et_2O (5 mL) was added dropwise and the solution was stirred (-5 °C, 1 h). The reaction mixture was then quenched by slow addition of saturated aqueous NH_4Cl (10 mL). The phases were separated, and the aqueous phase was extracted with Et_2O (2 x 10 mL), the combined ethereal phases were dried over anhydrous $MgSO_4$, filtered, and concentrated *in vacuo*. The crude product was isolated by column chromatography

on silica gel using petroleum ether and EtOAc as the eluent mixture to afford the target cyclopropyl ketones.

4,4,7,7-Tetramethylbicyclo[4.1.0]heptan-2-one (3y)

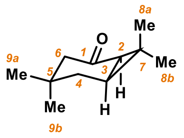

Prepared according to General Procedure 2 using 4,7,7-trimethylbicyclo[4.1.0]hept-3-en-2-one **SI-13** (750 mg, 5.0 mmol). The crude residue was purified by column chromatography R_f = 0.30 (silica gel, petroleum ether:EtOAc = 19:1) to afford the target ketone **3y** as a colorless oil (690 mg, 4.20 mmol, 85% yield).

^{1}H NMR (400 MHz, $CDCl_3$) δ 1.98 (ddd, J = 16.6, 3.2, 1.2 Hz, 1H, *H-6*), 1.84 – 1.77 (m, 1H, *H-6*), 1.77 – 1.71 (m, 1H, *H-4*), 1.52 – 1.45 (m, 2H, *H-2* + *H-3*), 1.44 – 1.38 (m, 1H, *H-4*), 1.17 (s, 3H, *3H-8a*), 1.14 (s, 3H, *3H-8b*), 0.98 (s, 3H, *3H-9a*), 0.96 (s, 3H, *3H-9b*).

^{13}C{H} NMR (101 MHz, $CDCl_3$) δ 209.9 (*C-1*), 52.9 (*C-6*), 37.1 (*C-5*), 34.8 (*C-2*), 32.3 (*C-4*), 30.3 (*C-9a*), 29.1 (*C-8a*), 28.9 (*C-3*), 28.5 (*C-7*), 26.2 (*C-9b*), 16.0 (*C-8b*).

IR (neat, cm^{-1}): 2953, 2868, 1680, 1460, 1386, 1242 (fingerprint region excluded).

HRMS (ESI$^+$): calculated for $C_{11}H_{18}ONa$ (M^+Na^+): 189.1250 Found: 189.1246.

4-*iso*-Butyl-4,7,7-trimethylbicyclo[4.1.0]heptan-2-one (3z)

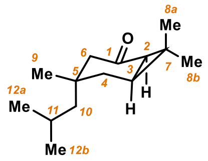

Prepared according to General Procedure 2 using 4,7,7-trimethylbicyclo[4.1.0]hept-3-en-2-one **SI-13** (465 mg, 3.0 mmol). The crude residue was purified by column chromatography R_f = 0.20 (silica gel, petroleum ether:EtOAc = 9:1) to afford the target ketone **3z** as a pale-yellow oil (313 mg, 1.51 mmol, 50% yield).

^{1}H NMR (400 MHz, $CDCl_3$) δ 2.12 (ddd, *J* = 16.8, 3.3, 1.5 Hz, 1H, *H-6a*), 1.91 (ddd, *J* = 14.6, 9.1, 3.3 Hz, 1H, *H-4a*), 1.76 – 1.58 (m, 2H, *H-6b* + *H-11*), 1.52 – 1.46 (m, 1H, *H-2*), 1.45 – 1.37 (m, 1H, *H-3*), 1.30 (dd, *J* = 14.7, 4.8 Hz, 1H, *H-4b*), 1.27 – 1.22 (m, 2H, *2H-10*), 1.17 (s, 3H, *3H-8a*), 1.14 (s, 3H, *3H-8b*), 0.97 (s, 3H, *3H-9*), 0.93 (d, *J* = 6.7 Hz, 3H, *3H-12a*), 0.88 (d, *J* = 6.7 Hz, 3H, *3H-12b*).

^{13}C{H} NMR (126 MHz, $CDCl_3$) δ 210.1 (*C-1*), 52.7 (*C-6*), 47.0 (*C-10*), 40.5 (*C-5*), 35.2 (*C-2*), 31.5 (*C-4*), 29.2 (*C-8a*), 28.9 (*C-3*), 28.8 (*C-7*), 27.6 (*C-9*), 25.4 (*C-12a*), 25.3 (*C-12b*), 24.0 (*C-11*), 16.3 (*C-8b*).

IR (neat, cm^{-1}): 2952, 2869, 1680, 1458 (fingerprint region excluded).

HRMS (ESI+): calculated for $C_{14}H_{25}O$ (M^+H^+): 209.1900 Found: 209.1903.

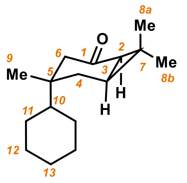

4-Cyclohexyl-4,7,7-trimethylbicyclo[4.1.0]heptan-2-one (3ab)

Prepared according to General Procedure 2 using 4,7,7-trimethylbicyclo[4.1.0]hept-3-en-2-one **SI-13** (465 mg, 3.0 mmol). The crude residue was purified by column chromatography R_f = 0.25 (silica gel, petroleum ether:EtOAc = 9:1) to afford the target ketone **3ab** as a yellow solid (438 mg, 1.84 mmol, 61% yield).

^{1}H NMR (400 MHz, $CDCl_3$) δ 2.38 (ddd, *J* = 16.3, 4.1, 1.6 Hz, 1H, *H-6a*), 2.15 (ddd, *J* = 13.8, 9.2, 3.7 Hz, 1H, *H-4a*), 1.84 – 1.69 (m, 4H, *2CH$_2$*), 1.62 (dd, *J* = 15.0, 7.9 Hz, 3H, *H-4b* + *H-6b* + *H-10*), 1.55 (s, 3H, *3H-8a*), 1.47 (d, *J* = 7.6 Hz, 1H, *H-2*), 1.42 – 0.84 (m, 10H, *H-3* + *3H-8b* + *3CH$_2$*), 0.81 (s, 3H, *3H-9*).

^{13}C{H} NMR (101 MHz, $CDCl_3$) δ 210.3 (*C-1*), 51.4 (*C-6*), 43.0 (*C-5*), 41.4 (*C-10*), 35.6 (*C-2*), 29.9 (*C-4*), 29.4 (*C-7*), 29.2 (*C-3*), 28.7 (*C-8a*), 27.3 (*CH$_2$*), 27.2 (*CH$_2$*), 27.2 (*CH$_2$*), 26.7 (*CH$_2$*), 26.6 (*CH$_2$*), 23.0 (*C-9*), 16.4 (*C-8b*).

Melting Point (from petroleum ether: Et_2O): 53 - 55 °C.

IR (neat, cm^{-1}): 2925, 2853, 1673, 1446, 1424, 1378 (fingerprint region excluded).

HRMS (ESI+): calculated for $C_{16}H_{27}O$ (M^+H^+): 235.2056 Found: 235.2069.

Synthesis of 4,7,7-Trimethyl-4-phenylbicyclo[4.1.0]heptan-2-one (3aa)

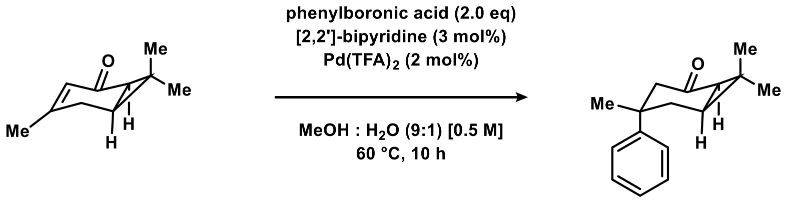

Procedure adapted from Minnaard and co-workers: [23]

To an oven-dried schlenk tube was added palladium(II) trifluoroacetate (33.2 mg, 0.1 mmol, 2 mol%) and 2,2'-bipyridine (23.4 mg, 0.15 mmol, 3 mol%), and the flask was evacuated and backfilled with N_2 three times. The flask was charged with a MeOH:H_2O (9:1) solution (8 mL), and the mixture was stirred vigorously (60 °C, 15 min). Trimethylbicyclo[4.1.0]hept-3-en-2-one **SI-13** (750 mg, 5 mmol, 1.0 eq) was added neat, followed by a solution of phenylboronic acid (1.21 g, 10 mmol, 2 eq) in 2 mL of MeOH:H_2O

(9:1), and the solution was stirred (60 °C, 10 h). The reaction mixture was cooled, filtered through a pad of silica, and the filtrate was dried over anhydrous $MgSO_4$ and concentrated *in vacuo*. The crude product was purified by column chromatography R_f = 0.40 (silica gel, hexane:EtOAc = 9:1) to afford the target cyclopropyl ketone **3aa** as a yellow oil (150 mg, 0.65 mmol, 13% yield).

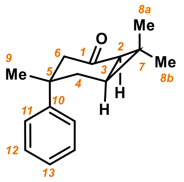

^{1}H NMR (400 MHz, $CDCl_3$) δ 7.31 (d, *J* = 4.3 Hz, 4H, *H-11* + *H-12*), 7.25 – 7.13 (m, 1H, *H-13*), 2.92 (ddd, *J* = 17.2, 3.7, 1.6 Hz, 1H, *H-6*), 2.69 (ddd, *J* = 15.0, 9.2, 3.6 Hz, 1H, *H-4*), 2.03 (d, J = 17.2 Hz, 1H, *H-6*), 1.61 (dd, J = 15.0, 5.4 Hz, 1H, *H-4*), 1.36 (ddd, *J* = 9.2, 7.6, 5.4 Hz, 1H, *H-3*), 1.26 (s, 3H, *H-8a*), 1.20 (s, 3H, *H-8b*), 1.19 – 1.15 (m, 1H, *H-2*), 1.10 (s, 3H, *H-9*).

^{13}C{H} NMR (101 MHz, $CDCl_3$) δ 209.2 (*C-1*), 146.1 (*C-10*), 128.4 (*C-12*), 125.9 (*C-11*), 125.9 (*C-13*), 52.1 (*C-6*), 44.4 (*C-5*), 34.5 (*C-2*), 32.1 (*C-4*), 32.1 (*C-8a*), 29.1 (*C-9*), 28.8 (*C-3*), 28.5 (*C-7*), 16.5 (*C-8b*).

IR (neat, cm^{-1}): 2952, 2868, 1681, 1444, 1343, 763, 700 (fingerprint region excluded).

HRMS (ESI+): calculated for $C_{16}H_{20}ONa$ (M^+Na^+): 251.1406 Found: 251.1401.

Synthesis of 6,6-Dimethylbicyclo[3.1.0]hexan-2-one (5)

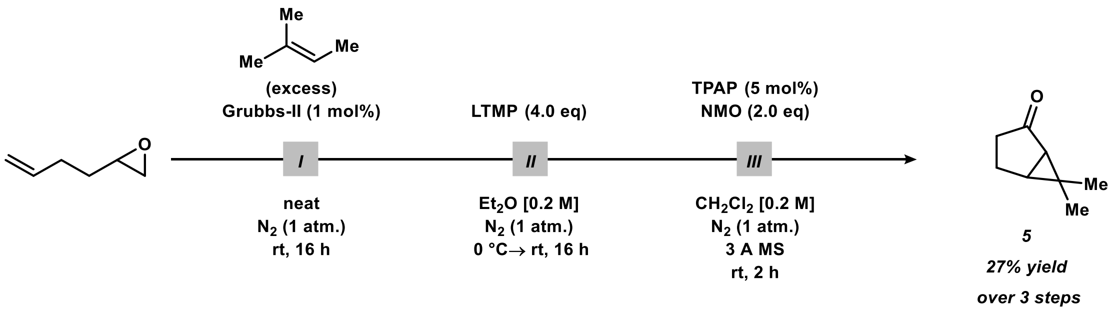

Procedures adapted from Hodgson and co-workers [4]

Step I: A solution of 1,2-epoxy-5-hexene (563 µL, 5.00 mmol, 1.0 eq) and Grubb's 2nd generation catalyst M204 (42 mg, 0.05 mmol, 1 mol%) in isoamylene (10 mL) was stirred (rt, 24 h). The volatile compounds were removed *in vacuo* to give a black oil. The crude residue was purified by column chromatography R_f = 0.40 (silica gel, pentane:Et_2O = 9:1) to afford the target epoxide **SI-14** as a colorless oil (547 mg, 4.40 mmol, 88% yield).

1,2-Epoxy-6,6-dimethyl-5-hexene (SI-14)

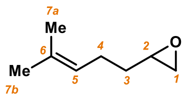

^{1}H NMR (400 MHz, $CDCl_3$) δ 5.14 (ddt, *J* = 8.6, 5.8, 1.5 Hz, 1H, *H-5*), 2.92 (tdd, *J* = 5.6, 3.9, 2.7 Hz, 1H, *H-2*), 2.47 (dd, *J* = 5.0, 2.8 Hz, 2H, *H-1*), 2.14 (q, *J* = 7.5 Hz, 2H, *2H-4*), 1.69 (s, 3H, *3H-7a*), 1.62 (s, 3H, *3H-7b*), 1.56 (td, *J* = 7.5, 5.6 Hz, 2H, *2H-3*).

^{13}C{H} NMR (101 MHz, $CDCl_3$) δ 132.5 (*C-6*), 123.4 (*C-5*), 52.2 (*C-2*), 47.3 (*C-1*), 32.9 (*C-3*), 25.8 (*C-7a*), 24.7 (*C-4*), 17.8 (*C-7b*).

Data in accordance with those previously reported.[4]

Step II: To a stirred cooled solution of 1,2-epoxy-6,6-dimethyl-5-hexene **SI-14** (818 mg, 6.50 mmol, 1.0 eq) in Et_2O (20 mL) was added a freshly prepared solution of LTMP (26 mmol, 0.22 M, 4.0 eq) *via* cannula bath (0 ºC, 60 min). The resultant mixture was stirred and gradually warmed (0 ºC to rt, 16 h). The reaction mixture was quenched with MeOH (10 mL), the solvent removed *in vacuo* and the residue adsorbed onto a small amount of silica and purified by column chromatography R_f = 0.10 (silica gel, petroleum ether:Et_2O = 7:3) to afford the target alcohol **SI-15** as a pale-yellow oil (742 mg, 5.87 mmol, 90% yield).

Note: *On larger scale preparations, 2,2,6,6-tetramethylpiperidine (TMP) typically co-eluted with the product during column chromatography. In these cases, the fractions were collected and dried in vacuo, the crude residue was then diluted in Et_2O (50 mL) and washed successively with saturated aqueous NH_4Cl (10 x 10 mL). The organic phase was then dried over anhydrous $MgSO_4$ and concentrated in vacuo to afford the target alcohol.*

6,6-Dimethylbicyclo[3.1.0]hexan-2-ol (SI-15)

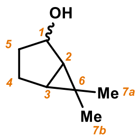

^{1}H NMR (500 MHz, $CDCl_3$) δ 4.14 (dd, *J* = 6.2, 1.5 Hz, 1H, *H-1*), 2.04 (dddd, *J* = 13.4, 11.2, 8.5, 5.9 Hz, 1H, *H-5a*), 1.92 – 1.78 (m, 1H, *H-5b*), 1.77 – 1.67 (m, *H-4a*), 1.55 (ddt, *J* = 12.9, 9.4, 3.3 Hz, 2H, *H-4b* + *OH*), 1.27 (t, *J* = 6.1 Hz, 1H, *H-2*), 1.13 (dd, *J* = 6.3, 1.4 Hz, 1H, *H-3*), 0.98 (s, 3H, 3*H-7b*), 0.92 (s, 3H, 3*H-7a*).

^{13}C{H} NMR (126 MHz, $CDCl_3$) δ 74.4 (*C-1*), 39.2 (*C-2*), 38.3 (*C-4*), 30.9 (*C-3*), 28.3 (*C-7a*), 23.5 (*C-6*), 18.6 (*C-5*), 15.1 (*C-7b*).

IR (neat, cm^{-1}): 3318 (br.), 2941, 2867, 1458 (fingerprint region excluded).

Data were in accordance with those previously reported.[4]

Step III: TPAP (105 mg, 0.30 mmol, 5 mol%) was added to a stirred solution of 6,6-dimethylbicyclo[3.1.0]hexan-2-ol **SI-15** (742 mg, 5.87 mmol), *N*-methylmorpholine *N*-oxide (NMO) (1.38 g, 11.8 mmol, 2.0 eq), and powdered activated 3Å molecular sieves (1.65 g) in CH_2Cl_2 (30 mL) and the mixture stirred (rt, 2 h). The mixture was filtered through a short pad of silica (2.5 cm × 6 cm) and eluted through with Et_2O (60 mL). The filtrate was concentrated *in vacuo* and the crude residue was purified by column chromatography R_f = 0.20 (silica gel, pentane:Et_2O = 9:1) eluent mixture to afford the target cyclopropyl ketone **5** as a colorless oil (379 mg, 3.05 mmol, 52% yield).

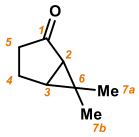

6,6-Dimethylbicyclo[3.1.0]hexan-2-one (5)

^{1}H NMR (400 MHz, $CDCl_3$) δ 2.35 - 2.13 (m, 2H, *H-5a* + *H-4b*), 2.03 (ddd, *J* = 17.3, 9.4, 4.5 Hz, 1H, *H-6b*), 1.96 - 1.81 (m, 2H, *H-3* + *H-4a*), 1.67 - 1.62 (m, 1H, *H-2*), 1.15 (s, 3H, *3H-7b*), 1.11 (s, 3H, *3H-7a*).

^{13}C{H} NMR (101 MHz, $CDCl_3$) δ 215.4 (*C-1*), 41.4 (*C-2*), 38.0 (*C-5*), 35.7 (*C-3*), 27.3 (*C-7a)*, 26.2 (*C-6)*, 19.7 (*C-4)*, 16.0 (*C-7b)*.

IR (neat, cm^{-1}): 2950, 2888, 1715, 1456, 1415 (fingerprint region excluded).

HRMS (ESI+): <u>not informative due to low molecular weight.</u>

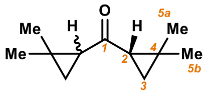

(2,2-Dimethylcyclopropyl)(2,2-dimethylcyclopropyl)methanone (7)

Prepared in 1 step according to General Procedure 1 step III using phorone (691 mg, 5.00 mmol). The resultant crude material was purified by column chromatography R_f = 0.45 (silica gel, petroleum ether: Et_2O = 19:1) to afford the target product to afford the target biscyclopropyl ketone **7** as a colorless oil and as a mixture of diastereoisomers (725 mg, 4.36 mmol, 87% yield, d.r. = 1.0:1.0).

^{1}H NMR (500 MHz, $CDCl_3$) δ 1.99 (td, *J* = 7.5, 5.5 Hz, 2H, *2H-2*), 1.25 (ddd, *J* = 5.5, 3.9, 1.6 Hz, 2H, *2H-3a*), 1.21 (s, 6H, *6H-5a*), 1.08 (d, *J* = 10.1 Hz, 6H, *6H-5b*), 0.78 (ddd, *J* = 12.7, 7.6, 3.9 Hz, 2H, *2H-3b*).

^{13}C{H} NMR (126 MHz, $CDCl_3$) δ 206.8 (*C-1*), 206.4 (*C-1*), 37.4 (*C-2*), 37.3 (*C-2*), 27.7 (*C-5a*), 27.5 (*C-5a*), 27.3 (*C-4*), 26.2 (*C-4*), 23.4 (*C-3*), 22.8 (*C-3*), 18.8 (*C-5b*), 18.5 (*C-5b*).

IR (neat, cm^{-1}): 2996, 2947, 2871, 1675, 1459, 1433, 1400 (fingerprint region excluded).

HRMS (APCI+): calculated for $C_{11}H_{19}O$ ($M^{+}H^{+}$): 167.1430 : Found: 167.1437.

2. SmI_2-catalyzed cross-coupling of alkylcyclopropyl ketones and alkynes/alkenes

2.1. General Procedures

General Procedure 3a: SmI_2-catalyzed intermolecular coupling reactions

An oven-dried microwave reaction vial containing a magnetic stirrer-bar was cooled under a stream of N_2 (1 min), immediately sealed and placed under a positive pressure of N_2. The vial was charged with the corresponding cyclopropyl ketone (0.10 mmol, 1.0 eq), and radical trap (0.30 mmol, 3.0 eq) further flushed with N_2 (15 min). The vial was charged with THF (0.50 mL) and placed in a pre-heated oil bath (55 °C, 1 min). Freshly prepared SmI_2 (0.150 mL, 0.10 M, 15 mol%) was introduced by syringe and the reaction was stirred and heated (400 rpm, 55 °C, 120 min). The reaction was quenched by injection of compressed air and cooled (rt, 5 min) and filtered through a silica gel pad (1.5 cm x 4 cm) using CH_2Cl_2 (15 mL) as the eluent.

General Procedure 3b: SmI_2/Sm-catalyzed intermolecular coupling reactions

An oven-dried microwave reaction vial containing a magnetic stirrer-bar was cooled under a high-pressure stream of N_2 (1 min), then charged with Sm metal (5 mg, 0.030 mmol, 15 mol%), immediately sealed and placed under vacuum. The vial was cycled between vacuum and N_2 (3 times) and left under a positive pressure of N_2. The vial was charged with the corresponding cyclopropyl ketone (0.20 mmol, 1.0 eq), and radical trap (0.60 mmol, 3.0 eq) and the vial was further flushed with N_2 (5 min). The vial was charged with THF (1.00 mL) and placed in a pre-heated oil bath (55 °C, 1 min). Freshly prepared SmI_2 (0.300 mL, 0.10 M, 15 mol%) was introduced by syringe and the reaction was stirred and heated (400 rpm, 55 °C, 240 min). The reaction was quenched by injection of compressed air, often resulting in a colour change from blue to yellow (Sm^{II} to Sm^{III}) and was cooled (rt, 15 min) and filtered through a silica gel pad (1.5 cm x 4 cm) using CH_2Cl_2 (15 mL) as the eluent.

To obtain ^{1}H qNMR yields for reactions, the filtrate was concentrated *in vacuo* and CH_2Br_2 in $CDCl_3$ (1 mL, 0.05 M, 0.05 mmol) was added to the crude residue and samples were submitted directly for quantitative ^{1}H NMR. For isolated yields the filtrate was concentrated *in vacuo* and the crude reside was purified by column chromatography using silica gel or by preparative TLC, using petroleum ether:Et_2O mixtures as eluent.

2.2. Characterisation data for the products of SmI_2-catalysis

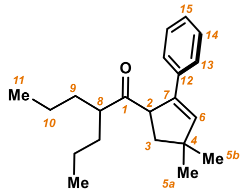

1-(4,4-Dimethyl-2-phenylcyclopent-2-en-1-yl)-2-propylpentan-1-one (2a)

Prepared by following the General Procedure 3b from **1a** (39.0 mg, 0.20 mmol, 1.0 eq), phenylacetylene (66 µL, 0.60 mmol, 3.0 eq), Sm metal (5.0 mg, 0.03 mmol, 15 mol%) and SmI_2 (300 µL, 0.1 M, 15 mol%). The crude residue was purified by column chromatography R_f = 0.50 (silica gel, petroleum ether:Et_2O = 9:1) to afford the target product **2a** as a colorless oil (44.0 mg, 0.147 mmol, 74% yield).

^{1}H NMR (400 MHz, $CDCl_3$) δ 7.31 – 7.13 (m, 5H, *2H-13* + *2H-14* + *H-15*), 6.00 (d, *J* = 1.6 Hz, 1H, *H-6*), 4.28 (ddd, *J* = 9.7, 5.4, 1.6 Hz, 1H, *H-2*), 2.69 – 2.59 (m, 1H, *H-8*), 2.13 (dd, *J* = 12.9, 9.7 Hz, 1H, *H-3a*), 1.88 (dd, *J* = 13.0, 5.4 Hz, 1H, *H-3b*), 1.67 – 1.20 (m, 8H, *2H-9a* + 2*H-9b* + 2*H-10a* + 2*H-10b*), 1.19 – 1.12 (m, 6H, *3H-5a* + *3H-5b*), 0.96 – 0.63 (m, 6H, 3*H-11a* + 3*H-11b*).

^{13}C{H} NMR (101 MHz, $CDCl_3$) δ 214.7 (*C-1*), 141.0 (*C-6*), 138.9 (*C-12*), 136.6 (*C-7*), 128.4 (*C-13*), 127.2 (*C-15*), 126.2 (*C-14*), 57.9 (*C-2*), 50.5 (*C-8*), 45.3 (*C-4*), 42.9 (*C-3*), 33.6 (*C-9a*), 32.9 (*C-9b*), 29.5 (*C-5a*), 29.0 (*C-5b*), 20.7 (*C-10a*), 20.5 (*C-10b*), 14.3 (*C-11*).

IR (neat, cm^{-1}): 2955, 2930, 2870, 1708, 1463, 1447 (fingerprint region excluded).

HRMS (ESI+): calculated for $C_{21}H_{30}ONa$ (M^+Na^+): 321.2189 Found: 321.2189.

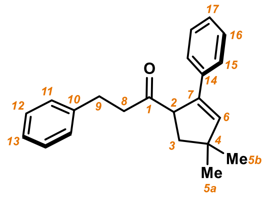

1-(4,4-Dimethyl-2-phenylcyclopent-2-en-1-yl)-3-phenylpropan-1-one (2b)

Prepared by following the General Procedure 3a from **1b** (21 µL, 0.10 mmol, 1.0 eq), phenylacetylene (33 µL, 0.30 mmol, 3.0 eq), and SmI_2 (150 µL, 0.1 M, 15 mol%). The crude residue was purified by column chromatography R_f = 0.30 (silica gel, petroleum ether:Et_2O = 9:1) to afford the target product **2b** as a yellow oil (27 mg, 0.089 mmol, 89% yield).

^{1}H NMR (400 MHz, $CDCl_3$) δ 7.32 – 7.11 (m, 8H, *2H-11* + *2H-12* + *H-13* + *2H-16* + *H-17*), 7.05 (m, 2H, *2H-15*), 6.10 (d, *J* = 1.7 Hz, 1H, *H-6*), 4.14 (ddd, *J* = 9.5, 6.6, 1.8 Hz, 1H, *H-2*), 2.81 (dddd, *J* = 17.5, 7.6, 6.2, 1.8 Hz, 2H, 2*H-8*), 2.70 (ddd, *J* = 12.0, 4.9, 2.0 Hz, 1H, *H-9a*), 2.61 – 2.48 (m, 1H, *H-9b*), 2.10 (ddd, *J* = 13.2, 9.5, 1.8 Hz, 1H, *H-3a*), 1.69 (ddd, *J* = 13.2, 6.6, 1.8 Hz, 1H, *H-3b*), 1.16 (d, *J* = 1.7 Hz, 3H, *3H-5a*), 1.12 (d, *J* = 1.7 Hz, 3H, *3H-5b*).

^{13}C{H} NMR (126 MHz, $CDCl_3$) δ 211.9 (*C-1*), 141.4 (*C-14*), 141.1 (*C-6*), 138.3 (*C-10*), 135.8 (*C-7*), 128.7 (*C-15*), 128.5 (*C-11 + C-12*), 127.5 (*C-17*), 126.1 (*C-12*), 125.9 (*2C-16*), 59.4 (*C-2*), 45.5 (*C-4*), 42.6 (*C-8*), 40.9 (*C-3*), 30.0 (*C-9*), 29.1 (*C-5a*), 29.1 (*C-5b*).

Data in accordance with those previously reported.[6]

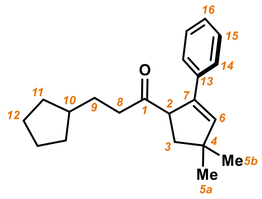

3-Cyclopentyl-1-(4,4-dimethyl-2-phenylcyclopent-2-en-1-yl)propan-1-one (2c)

Prepared by following the General Procedure 3a from **1c** (20 µL, 0.1 mmol, 1.0 eq), phenylacetylene (33 µL, 0.3 mmol, 3.0 eq), and SmI_2 (150 µL, 0.1 M, 15 mol%). The crude residue was purified by preparative thin layer chromatography R_f = 0.40 (silica gel, petroleum ether:Et_2O = 19:1) to afford the target product **2c** as a yellow oil (25 mg, 0.084 mmol, 84% yield).

^{1}H NMR (400 MHz, $CDCl_3$) δ 7.34 – 7.16 (m, 5H, *2H-14* + *2H-15* + *H-16*), 6.10 (d, *J* = 1.7 Hz, 1H, *H-6*), 4.16 (ddd, *J* = 9.4, 6.5, 1.8 Hz, 1H, *H-2*), 2.49 (ddd, *J* = 17.1, 9.0, 6.1 Hz, 1H, *H-8a*), 2.31 – 2.13 (m, 2H, *H-3a* + *H-8b*), 1.84 (dd, *J* = 13.1, 6.5 Hz,1H, *H-3b*), 1.69 – 1.38 (m, 9H, 2*H-9* + *H-10* + *2H-11a* + *4H-12*), 1.23 (s, 3H, *3H-5a*), 1.15 (s, 3H, *3H-5b*), 1.02 – 0.84 (m, 2H, *2H-11b*).

^{13}C{H} NMR (101 MHz, $CDCl_3$) δ 213.3 (*C-1*), 140.9 (*C-6*), 138.6 (*C-13*), 135.9 (*C-7*), 128.6 (*C-14*), 127.5 (*C-16*), 125.9 (*C-15*), 59.4 (*C-2*), 45.5 (*C-4*), 43.1 (*C-3*), 39.6 (*C-10*), 38.5 (*C-8*), 32.6 (*C-12*), 29.9 (*C-9*), 29.2 (*C-5a* + *C-5b*), 25.2 (*C-11*).

IR (neat, cm^{-1}): 2949, 2863, 1704, 1447, 1360 (fingerprint region excluded).

HRMS (ESI+): calculated for $C_{21}H_{28}ONa$ (M^+Na^+): 296.2140 Found: 296.2149.

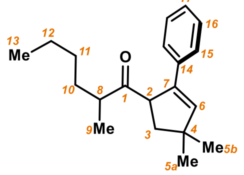

1-(4,4-Dimethyl-2-phenylcyclopent-2-en-1-yl)-2-methylhexan-1-one (2d)

Prepared by following the General Procedure 3a from **1d** (20 µL, 0.1 mmol, 1.0 eq), phenylacetylene (33 µL, 0.3 mmol, 3.0 eq), and SmI_2 (150 µL, 0.1 M, 15 mol%). The crude residue was purified by preparative thin layer chromatography R_f = 0.50 (silica gel, petroleum ether:Et_2O = 19:1) to afford the target product **2d** as a yellow oil and as a mixture of diastereoisomers (19 mg, 0.065 mmol, 65% yield, 1:1 d.r.).

^{1}**H NMR** (400 MHz, $CDCl_3$) δ 7.32 – 7.23 (m, 4H, *4H-Ar*), 7.21 – 7.15 (m, 1H, *H-Ar*), 6.08 – 6.03 (m, 1H, *H-6*), 4.35 – 4.24 (m, 1H, *H-2*), 2.79 – 2.63 (m, 1H, *H-8*), 2.22 – 2.12 (m, 1H, *H-3a*), 1.94 – 1.82 (m, 1H, *H-3b*), 1.70 – 1.03 (m, 12H, *3H-5a* + *3H-5b* *2H-10* + *2H-11* + *2H-12*), 0.94 – 0.78 (m, 6H, *3H-9* + *3H-13*).

^{13}**C{H} NMR** (101 MHz, $CDCl_3$) δ 216.1 (*C-1*), 215.5 (*C-1*), 140.8 (*C-6*), 140.7 (*C-6*), 138.7 (*C-14*), 138.6 (*C-14*), 136.2 (*C-7*), 128.3 (*C-15*), 127.2 (*C-17*), 126.0 (*C-16*), 125.9 (*C-16*), 58.1 (*C-2*), 57.5 (*C-2*), 45.3 (*C-4*), 45.2 (*C-4*), 44.7 (*C-8*), 44.2 (*C-8*), 43.0 (*C-3*), 43.0 (*C-3*), 33.2 (*CH_2*), 32.3 (*CH_2*), 29.4 (*C-5a*), 29.2 (*C-5a*), 29.20 (*C-5b*), 28.8 (*CH_2*), 22.7 (*CH_2*), 17.2 (*C-9*), 16.7 (*C-9*), 14.0 (*C-13*), 13.9 (*C-13*).

IR (neat, cm^{-1}): 2954, 2929, 2861, 1708, 1459, 1446, 1360 (fingerprint region excluded).

HRMS (ESI+): calculated for $C_{20}H_{28}ONa$ (M^+Na^+): 284.2140 Found: 284.2152.

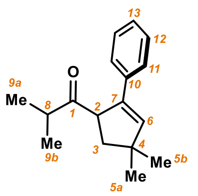

1-(4,4-Dimethyl-2-phenylcyclopent-2-en-1-yl)-2-methylpropan-1-one (2e)

Prepared by following the General Procedure 3b from **1e** (28 mg, 0.20 mmol, 1.0 eq), phenylacetylene (66 µL, 0.60 mmol, 3.0 eq), Sm metal (5 mg, 0.03 mmol, 15 mol%) and SmI_2 (300 µL, 0.1 M, 15 mol%). The crude residue was purified by column chromatography R_f = 0.50 (silica gel, petroleum ether:Et_2O = 19:1) to afford the target product **2e** as a pale-yellow oil (36.0 mg, 0.145 mmol, 74% yield).

^{1}**H NMR** (400 MHz, $CDCl_3$) δ 7.30 – 7.14 (m, 5H, *2H-11* + *2H-12* + *H-13*), 6.07 (d, *J* = 1.6 Hz, 1H, *H-6*), 4.29 (ddd, *J* = 9.6, 6.1, 1.7 Hz, 1H, *H-2*), 2.90 – 2.75 (m, 1H, *H-8*), 2.17 (dd, *J* = 13.0, 9.5 Hz, 1H, *H-3a*), 1.84 (dd, *J* = 13.0, 6.2 Hz, 1H, *H-3b*), 1.19 (s, 3H, *3H-5a*), 1.13 (s, 3H, *3H-5b*), 1.09 (d, *J* = 6.8 Hz, 3H, *3H-9a*), 0.91 (d, *J* = 6.8 Hz, 3H, *3H-9b*).

^{13}**C{H} NMR** (126 MHz, $CDCl_3$) δ 216.6 (*C-1*), 140.9 (*C-6*), 138.7 (*C-10*), 136.2 (*C-7*), 128.6 (*C-11*), 127.3 (*C-13*), 126.0 (*C-12*), 57.7 (*C-2*), 45.5 (*C-4*), 43.4 (*C-3*), 38.9 (*C-8*), 29.3 (*C-5a*), 29.0 (*C-5b*), 19.6 (*C-9a*), 18.9 (*C-9b*).

IR (neat, cm^{-1}): 3030, 2956, 2931, 2866, 1707, 1494, 1465, 1446 (fingerprint region excluded).

HRMS (ESI+): calculated for $C_{17}H_{22}ONa$ (M^+Na^+): 265.1563 Found: 265.1558.

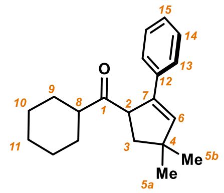

Cyclohexyl(4,4-dimethyl-2-phenylcyclopent-2-en-1-yl)methanone (2f)

Prepared by following the General Procedure 3a from **1f** (18.0 mg, 0.10 mmol, 1.0 eq), phenylacetylene (33 µL, 0.30 mmol, 3.0 eq), and SmI_2 (150 µL, 0.1 M, 15 mol%). The crude residue was purified by column chromatography R_f = 0.50 (silica gel, petroleum ether:Et_2O = 9:1) to afford the target product **2f** as a white amorphous solid (25.0 mg, 0.089 mmol, 89% yield).

^{1}H NMR (400 MHz, $CDCl_3$) δ 7.30 – 7.12 (m, 5H, *2H-13* + *2H-14* + *H-15*), 6.07 (d, *J* = 1.7 Hz, 1H, *H-6*), 4.28 (ddd, *J* = 9.6, 6.0, 1.7 Hz, 1H, *H-2*), 2.61 – 2.49 (m, 1H, *H-8*), 2.16 (dd, *J* = 13.0, 9.6 Hz, 1H, *H-3a*), 1.98 – 1.47 (m, 6H, *H-3b* + *4H-9* + *H-11*), 1.45 – 1.01 (m, 11H, *4H-10* + *H-11* + *3H-5a* + *3H-5b*).

^{13}C{H} NMR (101 MHz, $CDCl_3$) δ 215.5 (*C-1*), 140.8 (*C-6*), 138.7 (*C-12*), 136.2 (*C-7*), 128.5 (*C-13*), 127.3 (*C-15*), 126.0 (*C-14*), 57.6 (*C-2*), 49.3 (*C-8*), 45.5 (*C-4*), 43.4 (*C-3*), 29.7 (*C-9a*), 29.3 (*C-5a*), 29.0 (*C-5b*), 28.9 (*C-9b*), 25.9 (*C-10*), 25.8 (*C-11*).

Melting Point (from petroleum ether: Et_2O): 53 – 56 °C.

IR (neat, cm^{-1}): 2927, 2854, 1703, 1494,1446, (fingerprint region excluded).

HRMS (ESI+): calculated for $C_{20}H_{26}ONa$ (M^+Na^+): 305.1876 Found: 305.1889.

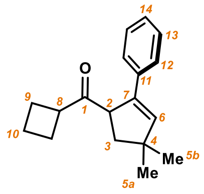

Cyclobutyl(4,4-dimethyl-2-phenylcyclopent-2-en-1-yl)methanone (2g)

Prepared by following the General Procedure 3b from **1g** (30.5 mg, 0.20 mmol, 1.0 eq), phenylacetylene (66 µL, 0.60 mmol, 3.0 eq), Sm metal (5 mg, 0.03 mmol, 15 mol%), and SmI_2 (300 µL, 0.1 M, 15 mol%). The crude residue was purified by column chromatography R_f = 0.35 (silica gel, petroleum ether:Et_2O = 19:1) to afford the target product **2g** as a colorless oil (38.6 mg, 0.150 mmol, 76% yield).

^{1}H NMR (400 MHz, $CDCl_3$) δ 7.34 – 7.01 (m, 5H, *2H-12* + *2H-13* + *H-14*), 6.07 (d, *J* = 1.7 Hz, 1H, *H-6*), 4.13 (ddd, *J* = 9.4, 6.5, 1.8 Hz, 1H, *H-2*), 3.47 – 3.34 (m, 1H, *H-8*), 2.36 – 1.58 (m, 8H, *H-3a* + *H-3b* + *2H-9a* + *2H-9b* + *2H-10*), 1.20 (s, 3H, *3H-5a*), 1.12 (s, 3H, *3H-5b*).

^{13}C{H} NMR (101 MHz, $CDCl_3$) δ 216.7 (*C-1*), 140.9 (*C-6*), 138.8 (*C-11*), 136.1 (*C-7*), 128.5 (*C-12*), 127.4 (*C-14*), 126.0 (*C-13*), 59.2 (*C-2*), 48.5 (*C-8*), 45.5 (*C-4*), 43.0 (*C-3*), 31.5 (*C-9a*), 30.7 (*C-9b*), 29.1 (*C-5*), 26.4 (*C-10a*), 26.3 (*C-10b*).

IR (neat, cm^{-1}): 2950, 2864, 1697, 1494, 1462, 1446, (fingerprint region excluded).

HRMS (ESI+): calculated for $C_{18}H_{22}ONa$ (M^+Na^+): 277.1563 Found: 277.1558.

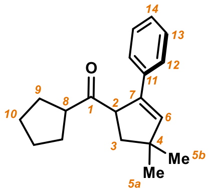

Cyclopentyl(4,4-dimethyl-2-phenylcyclopent-2-en-1-yl)methanone (2h)

Prepared by following the General Procedure 3b from **1h** (33.0 mg, 0.20 mmol, 1.0 eq), phenylacetylene (66 µL, 0.60 mmol, 3.0 eq), Sm metal (5 mg, 0.03 mmol, 15 mol%) and SmI_2 (300 µL, 0.1 M, 15 mol%). The crude residue was purified by column chromatography R_f = 0.45 (silica gel, petroleum ether:Et_2O = 9:1) to afford the target product **2h** as a colorless oil (41.5 mg, 0.155 mmol, 77% yield).

^{1}H NMR (400 MHz, $CDCl_3$) δ 7.32 – 7.12 (m, 5H, *2H-12* + *2H-13* + *H-14*), 6.07 (d, *J* = 1.7 Hz, 1H, *H-6*), 4.21 (ddd, *J* = 8.7, 6.6, 1.7 Hz, 1H, *H-2*), 3.05 – 2.92 (m, 1H, *H-8*), 2.15 (dd, *J* = 13.0, 9.4 Hz, 1H, *H-3a*), 1.84 (dd, *J* = 13.1, 6.6 Hz, 1H, *H-3b*), 1.80 – 1.31 (m, 8H, 2*H-9a* + 2*H-9b* + 2*H-10a* + 2*H-10b*), 1.19 (s, 3H, *3H-5a*), 1.13 (s, 3H, *3H-5b*).

^{13}C{H} NMR (101 MHz, $CDCl_3$) δ 216.7 (*C-1*), 140.9 (*C-6*), 138.8 (*C-11*), 136.1 (*C-7*), 128.5 (*C-12*), 127.4 (*C-14*), 126.0 (*C-13*), 59.2 (*C-2*), 48.5 (*C-8*), 45.5 (*C-4*), 43.0 (*C-3*), 31.5 (*C-9a*), 30.7 (*C-9b*), 29.1 (*C-5a* + *C-5b two overlapping signals*), 26.4 (*C-10a*), 26.3 (*C-10b*).

IR (neat, cm^{-1}): 3029, 2952, 2865, 1700, 1576, 1494.

HRMS (ESI+): calculated for $C_{19}H_{24}ONa$ (M^+Na^+): 291.1719 Found: 291.1713.

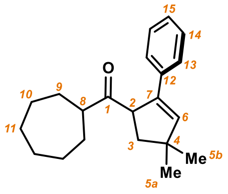

Cycloheptyl(4,4-dimethyl-2-phenylcyclopent-2-en-1-yl)methanone (2i)

Prepared by following the General Procedure 3b from **1i** (39.0 mg, 0.20 mmol, 1.0 eq), phenylacetylene (66 µL, 0.60 mmol, 3.0 eq), Sm metal (5 mg, 0.03 mmol, 15 mol%) and SmI_2 (300 µL, 0.1 M, 15 mol%). The crude residue was purified by column chromatography R_f = 0.25 (silica gel, petroleum ether:Et_2O = 9:1) to afford the target product **2i** as a white amorphous solid (53.3 mg, 0.180 mmol, 90% yield).

^{1}H NMR (400 MHz, $CDCl_3$) δ 7.30 – 7.12 (m, 5H, *2H-13* + *2H-14* + *H-15*), 6.04 (d, *J* = 1.7 Hz, 1H, *H-6*), 4.25 (ddd, *J* = 9.5, 6.0, 1.7 Hz, 1H, *H-2*), 2.70 (tt, *J* = 9.6, 4.1 Hz, 1H, *H-8*), 2.15 (dd, *J* = 13.0, 9.5 Hz, 1H, *H-3a*), 1.91 – 1.79 (m, 1H, *H-3b*), 1.83 – 1.25 (m, 12H, 2*H-9a* + 2*H-9b* + 2*H-10a* + 2*H-10b* + 2*H-11a* + 2*H-11b*), 1.18 (s, 3H, *3H-5a*), 1.13 (s, 3H, *3H-5b*).

^{13}C{H} NMR (101 MHz, $CDCl_3$) δ 216.2 (*C-1*), 140.9 (*C-6*), 138.9 (*C-12*), 136.3 (*C-7*), 128.5 (*C-13*), 127.3 (*C-15*), 126.0 (*C-14*), 58.2 (*C-2*), 50.3 (*C-8*), 45.4 (*C-4*), 43.4 (*C-3*), 31.1 (*C-9a*), 30.4 (*C-9b*), 29.3 (*C-5a*), 29.0 (*C-5b*), 28.3 (*C-11a*), 28.3 (*C-11b*), 26.9 (*C-10a*), 26.8 (*C-10b*).

Melting Point (from petroleum ether: Et_2O): 50 – 52 °C.

IR (neat, cm^{-1}): 2924, 2859, 1706, 1446 (fingerprint region excluded).

HRMS (ESI+): calculated for $C_{21}H_{28}ONa$ (M^+Na^+): 319.2032 Found: 319.2048.

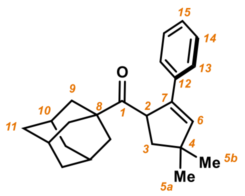

Adamantan-1-yl)(4,4-dimethyl-2-phenylcyclopent-2-en-1-yl)methanone (2j)

Prepared by following the General Procedure 3b from **1j** (46.4 mg, 0.20 mmol, 1.0 eq), phenylacetylene (66 µL, 0.60 mmol, 3.0 eq), Sm metal (5 mg, 0.03 mmol, 15 mol%) and SmI_2 (300 µL, 0.1 M, 15 mol%). The crude residue was purified by column chromatography R_f = 0.20 (silica gel, petroleum ether:Et_2O = 9:1) to afford the target product **2j** as a white solid (60.3 mg, 0.180 mmol, 90% yield).

^{1}H NMR (500 MHz, $CDCl_3$) δ 7.25 – 7.21 (m, 4H, *2H-13* + *2H-14*), 7.20 – 7.14 (m, 1H, *H-15*), 6.03 (d, *J* = 1.6 Hz, 1H, *H-6*), 4.62 (ddd, *J* = 9.5, 6.3, 1.6 Hz, 1H, *H-2*), 2.17 (dd, *J* = 12.8, 9.6 Hz, 1H, *H-3a*), 2.07 (m, 3H, *3H-10*), 1.99 – 1.81 (m, 6H, *6H-11*), 1.81 – 1.60 (m, 7H, *H-3b* + *6H-9*), 1.16 (s, 3H, *3H-5a*), 1.14 (s, 3H, *3H-5a*).

^{13}C{H} NMR (126 MHz, $CDCl_3$) δ 216.2 (*C-1*), 140.9 (*C-6*), 139.4 (*C-12*), 136.6 (*C-7*), 128.3 (*C-13*), 127.0 (*C-15*), 126.1 (*C-14*), 52.0 (*C-2*), 46.8 (*C-8*), 45.6 (*C-4*), 45.2 (*C-3*), 38.9 (*C-11*), 36.7 (*C-9*), 29.4 (*C-5a*), 29.2 (*C-5b*), 28.2 (*C-10*).

IR (neat, cm^{-1}): 2902, 2849, 1694, 1495, 1449 (fingerprint region excluded).

HRMS (ESI+): calculated for $C_{24}H_{30}ONa$ (M^+Na^+): 357.2189 Found: 357.2189.

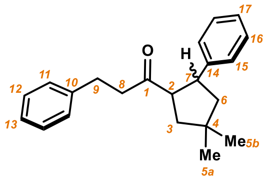

1-(4,4-Dimethyl-2-phenylcyclopentyl)-3-phenylpropan-1-one (2k)

Prepared by following the General Procedure 3a from **1b** (21 µL, 0.10 mmol, 1.0 eq), styrene (34 µL, 0.30 mmol, 3.0 eq) and SmI_2 (150 µL, 0.1 M, 15 mol%). The crude residue was purified by column chromatography R_f = 0.45 (silica gel, petroleum ether:Et_2O = 9:1) to afford the target product **2k** as a colorless oil and as a mixture of diastereoisomers (27.7 mg, 0.090 mmol, 90% yield, d.r. = 1.3:1).

^{1}**H NMR** (400 MHz, $CDCl_3$) δ 7.48 – 7.18 (m, 21H), 7.17 – 7.08 (m, 2H), 6.92 (dd, *J* = 7.2, 1.7 Hz, 2H), 3.87 – 3.66 (m, 1H, *H-2 major*), 3.62 – 3.45 (m, 2H, *H-2 minor* + *H-7 major*), 3.21 (q, *J* = 9.2 Hz, 1H, *H-7 minor*), 2.91 – 2.76 (m, 2H), 2.74 – 2.46 (m, 4H), 2.34 – 2.14 (m, 3H), 2.14 – 1.72 (m, 11H), 1.69 – 1.59 (m, 2H), 1.31 (s, 4H, *3H-5a major*), 1.22 (s, 3H, *3H-5a minor*), 1.19 – 1.12 (m, 7H, *3H-5b major* + *3H-5b minor*).

^{13}**C{H} NMR** (101 MHz, $CDCl_3$) δ 211.9 (*C-1 major*), 211.5 (*C-1 minor*), 143.9 (*Cq-Ar*), 142.1(*Cq-Ar*), 141.5 (*Cq-Ar*), 141.3 (*Cq-Ar*), 128.7 (*CH-Ar*), 128.6 (*CH-Ar*), 128.53 (*CH-Ar*), 128.50 (*CH-Ar*), 128.43 (*CH-Ar*), 128.38 (*CH-Ar*), 128.3 (*CH-Ar*), 127.4 (*CH-Ar*), 126.8 (*CH-Ar*), 126.5 (*CH-Ar*), 126.1 (*CH-Ar*), 125.9 (*CH-Ar*), 59.8 (*C-7 minor*), 56.3 (*C-7 major*), 50.3 (*CH_2*), 48.3 (*C-2 minor*), 47.8 (*C-2 major* + *CH_2*, overlapping), 46.5 (*CH_2*), 44.7 (*CH_2*), 44.5 (*CH_2*), 42.4 (*CH_2*), 38.52 (*C-4*), 38.45 (*C-4*), 30.7 (*C-5a minor*), 29.9 (*C-5b minor*), 29.6 (*CH_2*), 29.5 (*CH_2*), 29.4 (*C-5a major*), 28.7 (*C-5b major*).

IR (neat, cm^{-1}): 3027, 2951, 2864, 1706, 1495, 1453 (fingerprint region excluded).

HRMS (APCI+): calculated for $C_{22}H_{27}O$ ($M^{+}H^{+}$): 307.2056 Found: 307.2070.

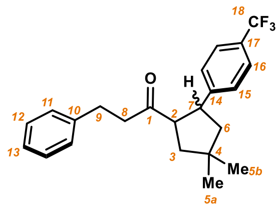

1-(4,4-Dimethyl-2-(4-(trifluoromethyl)phenyl)cyclopentyl)-3-phenylpropan-1-one (2l)

Prepared by following the General Procedure XX from **1b** (21 µL, 0.10 mmol, 1.0 eq), 4-trifluoromethylstyrene (44 µL, 0.30 mmol, 3.0 eq) and SmI_2 (150 µL, 0.1 M, 15 mol%). The crude residue was purified by column chromatography R_f = 0.45 (silica gel, petroleum ether:Et_2O = 9:1) to afford the target product **2l** as a colorless oil and as a mixture of diastereoisomers (33.7 mg, 0.090 mmol, 90% yield, d.r. = 1.6:1).

^{1}**H NMR** (400 MHz, $CDCl_3$) δ 7.52 (dd, *J* = 10.3, 8.0 Hz, 6H), 7.30 – 7.08 (m, 15H), 7.07 – 7.00 (m, 2H), 6.85 – 6.78 (m, 3H), 3.71 (ddd, *J* = 11.8, 10.0, 6.9 Hz, 2H, *H-2 major*), 3.60 (td, *J* = 11.0, 7.9 Hz, 1H, *H-2 minor*), 3.45 (dt, *J* = 10.1, 7.7 Hz, 2H, *H-7 major*), 3.09 (q, *J* = 9.4 Hz, 1H, *H-7 minor*), 2.79 (t, *J* = 7.4 Hz, 2H), 2.71 – 2.40 (m, 4H), 2.29 (ddd, *J* = 17.6, 10.1, 5.7 Hz, 2H), 2.09 (ddd, *J* = 13.0, 10.0, 6.1 Hz, 3H), 2.00 – 1.85 (m, 6H), 1.79 (ddd, *J* = 12.5, 6.9, 1.7 Hz, 2H), 1.72 – 1.54 (m, 5H), 1.23 (s, 6H, *3H-5a major*), 1.14 (s, 3H, *3H-5a minor*), 1.10 – 1.04 (m, 8H, *3H-5b minor* + *3H-5b major*).

^{13}**C{H} NMR** (126 MHz, $CDCl_3$) δ 211.3 (*C-1 major*), 210.8 (*C-1 minor*), 148.0 (*Cq-Ar*), 141.0, (*CH-Ar*) 128.4 (*CH-Ar*), 128.34 (*CH-Ar*), 128.27 (*CH-Ar*), 128.1 (*CH-Ar*), 127.61 (*CH-Ar*), 126.1 (*CH-Ar*), 125.9 (*CH-Ar*), 125.4 (*CH-Ar*), 125.30 (*CH-Ar*), 125.27 (*CH-Ar*), 59.6 (*C-7 minor*), 55.9 (*C-7 major*), 49.7

(*CH*$_2$), 47.4 (*C-2 major*), 47.3 (*CH*$_2$), 47.1 (*C-2 minor*), 46.5 (*CH*$_2$), 44.5 (*CH*$_2$), 44.4 (*CH*$_2$), 42.5 (*CH*$_2$), 38.6 (*C-4 minor*), 38.5 (*C-4 major*), 30.5 (*C-5a minor*), 29.7 (*C-5b minor*), 29.5 (*CH*$_2$), 29.3 (*C-5a major* + *CH*$_2$), 28.7 (*C-5b major*).

^{19}F NMR (376 MHz, CDCl$_3$) δ -62.38, -62.39.

IR (neat, cm^{-1}): 2953, 2867, 1707, 1618, 1453, 1323 (fingerprint region excluded).

HRMS (APCI+): calculated for $C_{23}H_{26}OF_3$ (M^+H^+): 375.1930 Found: 375.1938.

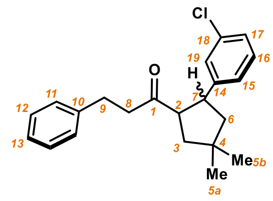

1-(2-(3-Chlorophenyl)-4,4-dimethylcyclopentyl)-3-phenylpropan-1-one (2m)

Prepared by following the General Procedure 3a from **1b** (21 µL, 0.10 mmol, 1.0 eq), 3-chlorostyrene (38 mg, 0.30 mmol, 3.0 eq) and SmI$_2$ (150 µL, 0.1 M, 15 mol%). The crude residue was purified by column chromatography R_f = 0.40 (silica gel, petroleum ether:Et$_2$O = 9:1) to afford the target product **2m** as a colorless oil and as a mixture of diastereoisomers (20.6 mg, 0.061 mmol, 61% yield, d.r. = 1.8:1).

^{1}H NMR (500 MHz, CDCl$_3$) δ 7.26 – 7.02 (m, 20H, *8H-Ar minor* + *8H-Ar major*), 6.90 – 6.84 (m, 3H, *H-Ar minor* + *H-Ar major*), 3.64 (ddd, *J* = 11.8, 10.2, 6.9 Hz, 2H, *H-2 major*), 3.52 – 3.45 (m, 1H, *H-2 minor*), 3.41 (dt, *J* = 10.2, 7.7 Hz, 2H, *H-7 major*), 3.05 (q, *J* = 9.1 Hz, 1H, *H-7 minor*), 2.82 – 2.75 (m, 2H, *H-3* major), 2.67 – 2.53 (m, 3H), 2.51 – 2.41 (m, 1H), 2.33 – 2.22 (m, 2H), 2.15 (ddd, *J* = 14.5, 10.0, 5.0 Hz, 2H), 2.10 – 1.94 (m, 4H), 1.94 – 1.82 (m, 4H), 1.80 – 1.72 (m, 2H), 1.71 – 1.54 (m, 5H), 1.22 (s, 5H, *3H-5a major*), 1.13 (s, 3H, *3H-5a minor*), 1.09 – 1.05 (m, 8H, *3H-5b major* + *3H-5b minor*).

^{13}C{H} NMR (126 MHz, CDCl$_3$) δ 211.6 (*C-1 major*), 211.1 (*C-1 minor*), 142.4 (*Cq-Ar*), 141.3 (*Cq-Ar*), 141.2 (*Cq-Ar*), 140.6 (*Cq-Ar*), 132.6 (*Cq-Ar*), 132.1 (*Cq-Ar*), 129.8 (*CH-Ar*), 128.75 (*CH-Ar*), 128.74 (*CH-Ar*), 128.64 (*CH-Ar*), 128.56 (*CH-Ar*), 128.5 (*CH-Ar*), 128.4 (*CH-Ar*), 128.3 (*CH-Ar*), 126.2 (*CH-Ar*), 126.0 (*CH-Ar*), 59.8 (*C-7 minor*), 56.0 (*C-7 major*), 50.1 (*CH*$_2$), 47.7 (*CH*$_2$), 47.22 (*C-2 major*), 47.20 (*C-2 minor*), 46.6 (*CH*$_2$), 44.6 (*CH*$_2$), 42.6 (*CH*$_2$), 38.6 (*C-4 minor*), 38.5 (*C-4 major*), 30.7 (*C-5a minor*), 29.9 (*C-5a major*), 29.7 (*CH*$_2$), 29.52 (*CH*$_2$), 29.46 (*C-5b major*), 28.8 (*C-5b minor*).

IR (neat, cm^{-1}): 2952, 2865, 1708, 1492, 1463, 1453 (fingerprint region excluded).

HRMS (APCI+): calculated for $C_{22}H_{26}OCl$ (M^+H^+): 341.1667 Found: 341.1671.

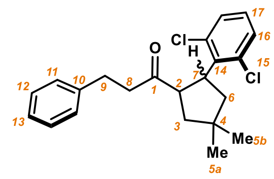

1-(2-(2,6-Dichlorophenyl)-4,4-dimethylcyclopentyl)-3-phenylpropan-1-one (2n)

Prepared by following the General Procedure 3b from **1b** (42 µL, 0.20 mmol, 1.0 eq), 2,6-dichlorostyrene (82 µL, 0.60 mmol, 3.0 eq), Sm metal (5 mg, 0.03, 15 mol%) and SmI_2 (300 µL, 0.1 M, 15 mol%). The crude residue was purified by column chromatography R_f = 0.60 (silica gel, petroleum ether:Et_2O = 9:1) to afford the target product **2n** as a colorless oil and as a mixture of diastereoisomers (68.3 mg, 0.182 mmol, 91% yield, d.r. = 1.5:1).

^{1}H NMR (500 MHz, $CDCl_3$) δ 7.29 – 7.24 (m, 6H), 7.23 – 7.18 (m, 5H), 7.16 – 7.11 (m, 2H), 7.09 – 7.02 (m, 6H), 7.00 – 6.96 (m, 2H), 4.67 (ddd, *J* = 12.5, 11.0, 8.5 Hz, 1H, *H-2 minor*), 4.51 (ddd, *J* = 12.2, 9.2, 8.1 Hz, 2H, *H-2 major*), 3.82 (ddd, *J* = 10.4, 9.2, 5.5 Hz, 2H, *H-7 major*), 3.36 (td, *J* = 11.9, 7.2 Hz, 1H, *H-7 minor*), 2.93 – 2.23 (m, 13H), 2.16 (d, *J* = 12.2 Hz, 2H), 2.00 (dd, *J* = 12.9, 10.3 Hz, 2H), 1.78 (ddd, *J* = 12.9, 5.5, 1.2 Hz, 2H), 1.72 – 1.60 (m, 4H), 1.20 (s, 3H, *3H-5a minor*), 1.14 (s, 5H, *3H-5a major*), 1.06 (s, 3H, *3H-5b minor*), 1.02 (s, 5H, *3H-5b major*).

^{13}C{H} NMR (126 MHz, $CDCl_3$) δ 210.9 (*C-1 major*), 209.1 (*C-1 minor*), 141.4 (*Cq-Ar*), 141.3 (*Cq-Ar*), 138.3 (*Cq-Ar*), 137.7 (*Cq-Ar*), 135.9 (*Cq-Ar*), 129.6 (*CH-Ar*), 129.5 (*CH-Ar*), 128.50 (*CH-Ar*), 128.46 (*CH-Ar*), 128.40 (*CH-Ar*), 128.37 (*CH-Ar*), 128.04 (*CH-Ar*), 127.97 (*CH-Ar*), 126.1 (*CH-Ar*), 126.0 (*CH-Ar*), 54.91 (*C-7 major*), 54.86 (*C-7 minor*), 44.9 (*CH_2*), 43.9 (*CH_2*), 43.7 (*CH_2*), 43.4 (*CH_2*), 43.2 (*CH_2*), 42.9 (*C-2 major*), 42.5 (*CH_2*), 41.0 (*C-2 minor*), 39.7 (*C-4 major*), 39.2 (*C-4 minor*), 30.2 (*C-5a major*), 30.00 (*CH_2*), 29.95 (*CH_2*), 29.0 (*C-5b major*), 28.9 (*C-5a minor*), 27.6 (*C-5b minor*).

IR (neat, cm^{-1}): 2952, 2931, 2866, 1708, 1559, 1463, 1434 (fingerprint region excluded).

HRMS (APCI+): calculated for $C_{22}H_{23}OCl_2$ (M^+H^+): 375.1277 Found: 375.1284.

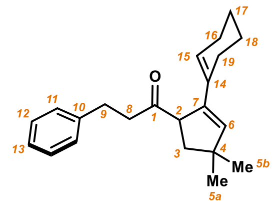

1-(2-(Cyclohex-1-en-1-yl)-4,4-dimethylcyclopent-2-en-1-yl)-3-phenylpropan-1-one (2o)

Prepared by following the General Procedure 3b from **1b** (42 µL, 0.20 mmol, 1.0 eq), 1-ethynylcyclohex-2-ene (70 µL, 0.60 mmol, 3.0 eq), Sm metal (5 mg, 0.03 mmol, 15 mol%) and SmI_2 (300 µL, 0.1 M, 15 mol%). The crude residue was purified by column chromatography R_f = 0.50 (silica gel, petroleum ether:Et_2O = 9:1) to afford the target product **2o** as a colorless oil (49.0 mg, 0.159 mmol, 80% yield).

^{1}H NMR (500 MHz, $CDCl_3$) δ 7.31 – 7.23 (m, 2H, *2H-Ar*), 7.22 – 7.13 (m, 3H, *3H-Ar*), 5.63 (d, *J* = 1.5 Hz, 1H, *H-6*), 5.40 (td, *J* = 4.0, 1.9 Hz, 1H, *H-15*), 3.84 (ddd, *J* = 10.0, 5.9, 1.5 Hz, 1H, *H-2*), 3.01 – 2.55 (m, 4H, *2H-8* + *2H-9*), 2.27 – 2.10 (m, 2H, CH_2), 2.05 – 1.92 (m, 3H, *H-3a* + CH_2), 1.64 (qd, *J* = 6.2, 2.5 Hz, 2H, CH_2), 1.57 – 1.50 (m, 3H, *H-3b* + CH_2), 1.08 (s, 3H, *3H-5a*), 1.05 (s, 3H, *3H-5b*).

^{13}C{H} NMR (126 MHz, $CDCl_3$) δ 212.6 (*C-1*), 141.5 (*C-10*), 139.9 (*C-7*), 138.2 (*C-6*), 132.9 (*C-14*), 128.5 (*C-ArH*), 128.4 (*C-ArH*), 126.0 (*C-ArH*), 125.6 (*C-15*), 58.7 (*C-2*), 45.0 (*C-4*), 42.2 (*C-3*), 40.0 (*C-9*), 30.0 (*C-8*), 29.6 (*C-5a*), 29.1 (*C-5b*), 26.3 (*C-16*), 25.6 (*C-19*), 22.6 (*C-17*), 22.2 (*C-18*).

IR (neat, cm^{-1}): 3030, 2926, 2861, 1704, 1604, 1495 (fingerprint region excluded).

HRMS (ESI+): calculated for $C_{22}H_{28}ONa$ (M^+Na^+): 331.2032 Found: 331.2049.

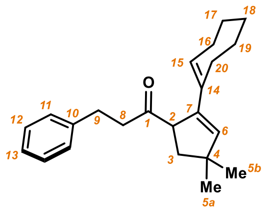

1-(2-(Cyclohept-1-en-1-yl)-4,4-dimethylcyclopent-2-en-1-yl)-3-phenylpropan-1-one (2p)

Prepared by following the General Procedure 3b from **1b** (42 µL, 0.20 mmol, 1.0 eq), 1-ethynylcyclohept-2-ene **SI-7** (72 mg, 0.60 mmol, 3.0 eq), Sm metal (5 mg, 0.03 mmol, 15 mol%) and SmI_2 (300 µL, 0.1 M, 15 mol%). The crude residue was purified by column chromatography R_f = 0.30 (silica gel, petroleum ether:Et_2O = 9:1) to afford the target product **2p** as a colorless oil (45.4 mg, 0.0141 mmol, 70% yield).

^{1}H NMR (400 MHz, $CDCl_3$) δ 7.33 – 7.21 (m, 2H, *2H-12*), 7.21 – 7.06 (m, 3H, 2*H-11* + *H-13*), 5.66 (d, *J* = 1.5 Hz, 1H, *H-6*), 5.59 (t, *J* = 6.8 Hz, 1H, *H-15*), 3.87 – 3.78 (m, 1H, *H-2*), 3.00 – 2.61 (m, 4H, *2H-8* + *2H-9*), 2.40 – 2.33 (m, 2H, CH_2), 2.16 – 2.07 (m, 2H, CH_2), 2.00 (dd, *J* = 13.3, 9.8 Hz, 1H, *H-3a*), 1.73 (q, *J* = 6.0 Hz, 2H, CH_2), 1.60 – 1.37 (m, 5H, *H-3b* + *2*CH_2), 1.09 (s, 3H, *3H-5a*), 1.05 (s, 3H, *3H-5a*).

^{13}C{H} NMR (101 MHz, $CDCl_3$) δ 212.8 (*C-1*), 141.6 (*C-10*), 140.7 (*C-7*), 140.6 (*C-14*), 139.1 (*C-6*), 130.0 (*C-15*), 128.6 (*C-12*), 128.5 (*C-11*), 126.1 (*C-13*), 59.2 (*C-2*), 45.1 (*C-4*), 42.4 (*C-3*), 39.8 (CH_2), 32.5 (CH_2), 30.3 (CH_2), 30.2 (CH_2), 29.5 (*C-5a*), 29.3 (*C-5b*), 28.5 (CH_2), 26.8 (CH_2), 26.5 (CH_2).

IR (neat, cm^{-1}): 3029, 2951, 2920, 2850, 1703, 1603, 1495, 1453 (fingerprint region excluded).

HRMS (ESI+): calculated for $C_{23}H_{31}O$ (M^+H^+): 323.2369 Found: 323.2364.

1-(2-(6,6-Dimethylbicyclo[3.1.1]hept-2-en-3-yl)-4,4-dimethylcyclopent-2-en-1-yl)-3-phenylpropan-1-one (2q)

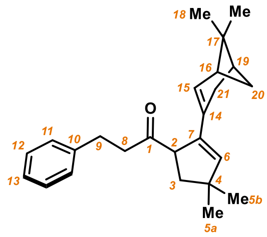

Prepared by following the General Procedure 3b from **1b** (42 µL, 0.20 mmol, 1.0 eq), (1*R*,5*S*)-2-ethynyl-6,6-dimethylbicyclo[3.1.1]hept-2-ene **SI-8** (88 mg, 0.60 mmol, 3.0 eq), Sm metal (5 mg, 0.03 mmol, 15 mol%) and SmI_2 (300 µL, 0.1 M, 15 mol%). The crude residue was purified by column chromatography R_f = 0.30 (silica gel, petroleum ether:Et_2O = 19:1) to afford the target product **2q** as a colorless oil and as a mixture of diastereoisomers (62.1 mg, 0.179 mmol, 90% yield, d.r. = 1.2:1).

^{1}H NMR (400 MHz, $CDCl_3$) δ 7.25 (m, 2H, *2H-12*), 7.16 (m, 3H, *2H-11* + *H-13*), 5.57 (m, 1H, *H-6*), 5.29 – 5.20 (m, 1H, *H-15*), 3.88 – 3.79 (m, 1H, *H-2*), 2.97 – 2.63 (m, 4H, *2H-8* + *2H-9*), 2.51 – 2.15 (m, 5H, *H-19* + *2H-20* + *2H-21*), 2.08 (td, *J* = 6.7, 3.2 Hz, 1H, *H-16*), 2.00 (ddt, *J* = 12.9, 9.7, 1.4 Hz, 1H, *H-3a*), 1.55 (ddt, *J* = 13.5, 6.5, 3.1 Hz, 1H, *H-3b*), 1.30 (s, 6H, *2CH₃*), 1.12 – 1.08 (m, 6H, $2CH_3$), 1.07 – 0.99 (m, 6H, $2CH_3$), 0.79 (s, 3H, CH_3), 0.76 (s, 3H, CH_3).

^{13}C{H} NMR (101 MHz, $CDCl_3$) δ 212.9 (*C-1*), 143.7, 141.6, 139.0, 138.6 (*C-6*), 138.4 (*C-6*), 128.6, 128.58, 128.53, 126.1 (*C-13*), 120.7 (*C-15*), 120.4 (*C-15*), 59.0 (*C-2*), 58.8 (*C-2*), 45.13 (*C-4*), 45.10 (*C-4*), 43.9 (*C-17*) , 43.4 (*C-17*), 42.4 (*C-3*), 42.3 (*C-3*), 40.7 (CH_2), 39.7 (CH_2), 39.6 (CH_2), 38.0 (CH_2), 37.7 (CH_2), 32.0 (CH_2), 31.9 (CH_2), 31.43 (CH_2), 31.39 (CH_2), 30.2 (CH_2), 30.0 (CH_2), 29.8 (CH_3), 29.6 (CH_3), 29.3 (CH_2), 26.5 (CH_3), 26.4 (CH_3), 21.2 (CH_3), 20.8 (CH_3).

IR (neat, cm^{-1}): 2951, 2918, 2866, 2828, 1703, 1496, 1453 (fingerprint region excluded).

HRMS (APCI+): calculated for $C_{25}H_{33}O$ (M^+H^+): 349.2526 Found: 349.2536.

1-(2-(2,6-Dimethylhepta-1,5-dien-1-yl)-4,4-dimethylcyclopent-2-en-1-yl)-3-phenylpropan-1-one (2r)

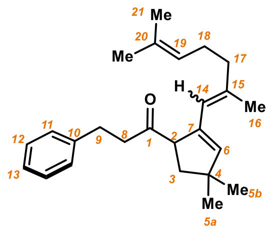

Prepared by following the General Procedure 3b from **1b** (42 µL, 0.20 mmol, 1.0 eq), 4,8-dimethylnona-3,7-dien-1-yne **SI-9** (89 mg, 0.60 mmol, 3.0 eq), Sm metal (5 mg, 0.03 mmol, 15 mol%) and SmI_2 (300 µL, 0.1 M, 15 mol%). The crude residue was purified by column chromatography R_f = 0.80 (silica gel, petroleum ether:Et_2O = 9:1) to afford the target product **2r** as a colorless oil and as a mixture of double bond isomers (50 mg, 0.140 mmol, 70% yield, d.r. = 1.3:1).

^{1}H NMR (400 MHz, $CDCl_3$) δ 7.31 – 7.12 (m, 5H, *5H-Ar*), 5.54 – 5.43 (m, 2H, *H-6* + *H-14 both isomers*), 5.16 – 4.97 (m, 1H, *H-19 both isomers*), 3.71 (dddd, *J* = 15.3, 8.9, 6.6, 1.6 Hz, 1H, *H-2 both isomers*), 2.93 – 2.83 (m, 1H), 2.22 – 1.98 (m, 1H), 1.90 (ddd, *J* = 13.7, 9.1, 4.8 Hz, 1H, *H-3a both isomers*), 1.77 – 1.53 (m, 5H), 1.08 (dd, *J* = 18.4, 3.8 Hz, 3H).

^{13}C{H} NMR (101 MHz, $CDCl_3$) δ 211.8 (*C-1 two overlapping signals*), 142.2, 141.4, 140.5, 135.9, 135.5, 128.4, 126.0, 124.0, 123.9, 120.3, 119.6, 61.4 (*C-2 two overlapping signals*), 45.2 (*C-4*), 45.1 (*C-4'*), 42.1, 42.0, 41.8 (*C-3 two overlapping signals*), 40.7, 33.4, 29.9 (*CH_3*), 29.2 (*CH_3*), 29.1 (*CH_3*), 29.06 *(CH_3)*, 29.02 (*CH_3*), 26.7, 25.7, 24.2, 18.4, 17.7**.**

IR (neat, cm^{-1}): 3027, 2953, 2926, 2862, 1707, 1604, 1496, 1452 (fingerprint region excluded).

HRMS (ESI+): calculated for $C_{25}H_{34}ONa$ ($M^{+}Na^{+}$): 373.2502 Found: 373.2502.

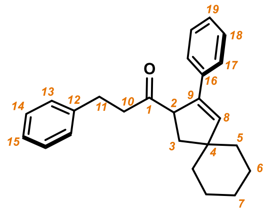

3-Phenyl-1-(3-phenylspiro[4.5]dec-3-en-2-yl)propan-1-one (2s)

Prepared by following the General Procedure 3b from **1s** (50 µL, 0.20 mmol, 1.0 eq), phenylacetylene (66 µL, 0.60 mmol, 3.0 eq), Sm metal (5 mg, 0.03 mmol, 15 mol%) and SmI_2 (300 µL, 0.1 M, 15 mol%). The crude residue was purified by column chromatography R_f = 0.30 (silica gel, petroleum ether:Et_2O = 19:1) to afford the target product **2s** as a white solid (50 mg, 0.141 mmol, 73% yield).

^{1}H NMR (400 MHz, $CDCl_3$) δ 7.33 – 7.17 (m, 8H, *8H-Ar*), 7.09 – 7.01 (m, 2H, *2H-Ar*), 6.24 (d, *J* = 1.7 Hz, 1H, *H-8*), 4.10 (ddd, *J* = 9.8, 6.3, 1.7 Hz, 1H, *H-2*), 2.96 – 2.63 (m, 3H, *2H-10* + *H-11*), 2.58 – 2.46 (m, 1H, *H-11*), 2.13 (dd, *J* = 13.4, 9.7 Hz, 1H, *H-3a*), 1.64 – 1.29 (m, 11H, *H-3b* + *4H-5* + *4H-6* + *2H-7*).

^{13}C{H} NMR (101 MHz, $CDCl_3$) δ 211.9 (*C-1*), 141.2 (*C-12*), 139.6 (*C-8*), 138.4 (*C-9*), 135.7 (*C-16*), 128.5 (*C-ArH*) , 128.4 (*C-ArH*), 128.3 (*C-ArH*), 127.4 (*C-ArH*), 126.0 (*C-ArH*), 125.7 (*C-ArH*), 58.5 (*C-2*), 49.6 (*C-4*), 40.6 (*C-11*), 38.0 (*C-3*), 37.7 (*CH_2*), 29.8 (*C-10*), 25.8 (*CH_2*), 23.3 (*CH_2*).

Melting Point (from petroleum ether: Et_2O): 61 - 63 °C.

IR (neat, cm^{-1}): 3027, 2922, 2851, 1704, 1602, 1494, 1447, 1405 (fingerprint region excluded).

HRMS (ESI+): calculated for $C_{24}H_{30}ONa$ ($M^{+}Na^{+}$): 367.2032 Found: 367.2046.

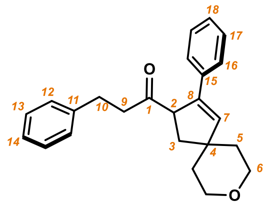

3-Phenyl-1-(3-phenyl-8-oxaspiro[4.5]dec-3-en-2-yl)propan-1-one (2t)

Prepared by following the General Procedure 3b from **1t** (50 µL, 0.20 mmol, 1.0 eq), phenylacetylene (66 µL, 0.60 mmol, 3.0 eq), Sm metal (5 mg, 0.03 mmol, 15 mol%) and SmI_2 (300 µL, 0.1 M, 15 mol%). The crude residue was purified by column chromatography R_f = 0.30 (silica gel, petroleum ether:Et_2O = 9:1) to afford the target product **2t** as a colorless oil (51 mg, 0.147 mmol, 74% yield).

^{1}H NMR (400 MHz, $CDCl_3$) δ 7.31 – 7.10 (m, 8H, *8H-Ar*), 7.07 – 7.00 (m, 2H, *2H-Ar*), 6.21 (d, *J* = 1.7 Hz, 1H, *H-7*), 4.11 (ddd, *J* = 9.8, 5.7, 1.7 Hz, 1H, *H-2*), 3.81 – 3.69 (m, 2H, 2*H-6*), 3.56 (ddd, *J* = 11.9, 9.0, 3.0 Hz, 1H, *H-6*), 3.46 (ddd, *J* = 11.9, 9.0, 3.0 Hz, 1H, *H-6*), 2.92 – 2.65 (m, 3H, 2*H-9* + *H-10*), 2.63 – 2.48 (m, 1H, *H-10*), 2.16 (dd, *J* = 13.5, 9.8 Hz, 1H, *H-3a*), 1.78 – 1.62 (m, 3H, *H-3b* + *2H-5*), 1.48 – 1.35 (m, 2H, *2H-5*).

^{13}C{H} NMR (101 MHz, $CDCl_3$) δ 211.3 (*C-1*), 141.3 (*C-11*), 139.8 (*C-8*), 137.6 (*C-7*), 135.4 (*C-15*), 128.8 (*C-ArH*), 128.6 (*C-ArH*), 128.5 (*C-ArH*), 127.9 (*C-ArH*), 126.2 (*C-ArH*), 126.0 (*C-ArH*), 65.3 (*C-6a*), 65.2 (*C-6b*), 58.4 (*C-2*), 47.2 (*C-4*), 41.5 (*C-10*), 38.8 (*C-3*), 37.7 (*C-5*), 29.7 (*C-9*).

IR (neat, cm^{-1}): 3027, 2925, 2846, 1705, 1602, 1495, 1467, 1446, 1406 (fingerprint region excluded).

HRMS (ESI+): calculated for $C_{24}H_{26}O_2Na$ (M^+Na^+): 369.1825 Found: 369.1834.

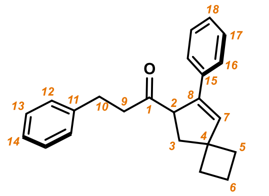

3-Phenyl-1-(7-phenylspiro[3.4]oct-7-en-6-yl)propan-1-one (2u)

Prepared by following the General Procedure 3b from **1u** (42 µL, 0.20 mmol, 1.0 eq), phenylacetylene (66 µL, 0.60 mmol, 3.0 eq), Sm metal (5 mg, 0.03 mmol, 15 mol%), and SmI_2 (150 µL, 0.1 M, 15 mol%). The crude residue was purified by column chromatography R_f = 0.45 (silica gel, petroleum ether:Et_2O = 9:1) to afford the target product **2u** as a yellow oil (40.2 mg, 0.127 mmol, 64% yield).

^{1}H NMR (400 MHz, $CDCl_3$) δ 7.36 – 7.15 (m, 8H, *8H-Ar*), 7.11 – 7.03 (m, 2H, *2H-Ar*), 6.52 (d, *J* = 1.5 Hz, 1H, *H-7*), 4.03 (ddd, *J* = 9.6, 4.4, 1.5 Hz, 1H, *H-2*), 2.86 – 2.68 (m, 3H, *2H-9* + *H-10a*), 2.56 – 2.48 (m, 1H, *H-10b*), 2.40 (dd, *J* = 13.8, 9.6 Hz, 1H, *H-3a*), 2.20 (dddd, *J* = 16.9, 8.6, 5.6, 3.0 Hz, 2H, *2H-5a*), 2.10 – 1.98 (m, 3H, *H-3b* + *2H-5b*), 1.91 (dddd, *J* = 18.1, 11.2, 5.6, 3.0 Hz, 2H, *2H-6*).

^{13}C{H} NMR (101 MHz, $CDCl_3$) δ 211.9 (*C-1*), 141.4 (*C-11*), 139.1 (*C-8*), 138.6 (*C-7*), 135.4 (*C-15*), 128.7 (*C-ArH*), 128.5 (2*C-ArH, 2 overlapping signals*), 127.6 (*C-ArH*), 126.1 (*C-ArH*), 125.9 (*C-ArH*), 59.6 (*C-2*), 52.6 (*C-4*), 42.5 (*C-3*), 40.3 (*C-10*), 35.4 (*C-5a*), 35.1 (*C-5b*), 29.8 (*C-9*), 16.6 (*C-6*).

IR (neat, cm^{-1}): 3027, 2926, 1703, 1495, 1446, 1358 (fingerprint region excluded).

HRMS (APCI+): calculated for $C_{23}H_{25}O$ (M^+H^+): 317.1900 Found: 317.1897.

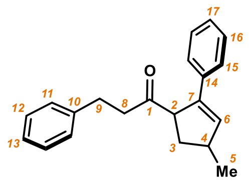

1-(4-Methyl-2-phenylcyclopent-2-en-1-yl)-3-phenylpropan-1-one (2v)

Prepared by following the General Procedure 3b from **1v** (42 µL, 0.20 mmol, 1.0 eq), phenylacetylene (66 µL, 0.60 mmol, 3.0 eq), Sm metal (5 mg, 0.03 mmol, 15 mol%), and SmI_2 (300 µL, 0.1 M, 15 mol%). The crude residue was purified by column chromatography R_f = 0.20 (silica gel, petroleum ether:CH_2Cl_2 = 3:2) to afford the target product **2v** as a colorless oil and as a mixture of diastereoisomers (11.0 mg, 0.037 mmol, 19% yield, d.r.= 1.0:1).

^{1}H NMR (400 MHz, $CDCl_3$) δ 7.34 – 7.11 (m, 16H, *8H-Ar* + *8H-Ar'*), 7.11 – 7.00 (m, 4H, *2H-Ar* + *2H-Ar'*), 6.27 (dd, *J* = 2.3, 1.3 Hz, 1H, *H-6*), 6.19 (t, *J* = 2.2 Hz, 1H, *H-6'*), 4.06 (dddd, *J* = 11.6, 7.1, 3.7, 1.7 Hz, 2H, *H-2* + *H-2'*), 3.11 – 2.45 (m, 11H, *H-3a'* + *H-4* + *H-4'* + *2H-8* + *2H-8'* + *2H-9* + *2H-9'*), 2.16 (ddd, *J* = 13.5, 8.0, 3.3 Hz, 1H, *H-3a*), 1.82 (ddd, *J* = 13.4, 9.8, 7.1 Hz, 1H, *H-3b*), 1.39 (dt, *J* = 13.4, 6.7 Hz, 1H, *H-3b'*), 1.14 – 1.11 (m, 6H, *3H-5* + *3H-5'*)

^{13}C{H} NMR (101 MHz, $CDCl_3$) δ 211.9 (*C-1*), 211.7 (*C-1'*), 141.3 (*C-10, 2 overlapping signals*), 140.4 (*C-7*), 140.2 (*C-7'*), 136.5 (*C-6*), 136.3 (*C-6'*), 135.6 (*C-14*), 135.3 (*C-14'*), 128.6 (*C-ArH*), 128.6 (*C-ArH*), 128.5 (*C-ArH*), 128.4 (*C-ArH*), 128.3 (*C-ArH*), 127.5 (*C-ArH*), 127.4 (*C-ArH*), 126.99 (*C-ArH*), 125.97 (*C-ArH*), 125.80 (*C-ArH*), 125.76 (*C-ArH*), 59.8 (*C-2*), 59.7 (*C-2'*), 40.7 (*C-9'*), 40.6 (*C-9*), 39.99 (*C-4*), 39.97 (*C-4'*), 36.8 (*C-3'*), 36.0 (*C-3*), 29.9 (*C-8*), 29.7 (*C-8'*), 21.1 (*C-5'*), 21.0 (*C-5*).

IR (neat, cm^{-1}): 2956, 2926, 92868, 1705, 1495, 1493 (fingerprint region excluded).

HRMS (APCI+): calculated for $C_{21}H_{23}O$ (M^+H^+): 291.1743 Found: 291.1743.

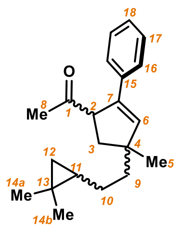

1-(4-((2,2-Dimethylcyclopropyl)methyl)-4-methyl-2-phenylcyclopent-2-en-1-yl)ethan-1-one (2w)

Prepared by following the General Procedure 3b from **1w** (39.0 mg, 0.20 mmol, 1.0 eq), phenylacetylene (66 µL, 0.60 mmol, 3.0 eq), Sm metal (5 mg, 0.03 mmol, 15 mol%), and SmI_2 (300 µL, 0.1 M, 15 mol%). The crude residue was purified by column chromatography R_f = 0.25 (silica gel, petroleum ether:Et_2O = 9:1) to afford the

target product **2w** as a pale-yellow oil and as a mixture of diastereoisomers (53.2 mg, 0.180 mmol, 90% yield, d.r. 1:1).

^{1}H NMR (400 MHz, $CDCl_3$) δ 7.36 – 7.17 (m, 5H, *5H-Ar in all diastereoisomers*), 6.18 – 6.09 (m, 1H, *H-6 in all diastereoisomers*), 4.16 – 4.02 (m, 1H, H-2 *in all diastereoisomers*), 2.33 – 2.21 (m, 1H), 2.15 – 2.04 (m, 1H), 2.04 – 1.96 (m, 3H), 1.94 – 1.88 (m, 0H), 1.77 (ddd, *J* = 13.5, 6.4, 1.5 Hz, 1H), 1.63 – 1.22 (m, 4H), 1.24 – 0.96 (m, 10H), 0.52 – 0.30 (m, 3H, *H-11a* + *2H-12 in all diastereoisomers*), -0.05 – -0.21 (m, 1H, *H-11b in all diastereoisomers*).

^{13}C{H} NMR (126 MHz, $CDCl_3$) δ 211.33 (*C-1*), 211.30 (*C-1'*), 140.1, 140.0, 139.7, 139.6, 138.9, 138.8, 135.58, 135.56, 128.60, 128.58, 127.4, 125.74, 125.65, 60.0, 59.9, 59.3, 48.8, 48.83, 48.73, 48.72, 42.69, 42.62, 42.27, 42.25, 40.75, 40.71, 40.66, 40.58, 29.71, 27.67, 27.64, 27.35, 27.34, 26.42, 26.40, 26.34, 26.31, 26.29, 25.69, 25.39, 25.37, 25.09, 25.05, 19.90, 19.88, 19.86, 19.77, 19.70, 19.68, 19.63, 15.49, 15.46, 15.43, 15.40.

IR (neat, cm^{-1}): 3055, 2923, 3864, 1706, 1495, 1447 (fingerprint region excluded).

HRMS (ESI+): calculated for $C_{21}H_{28}ONa$ ($M^{+}Na^{+}$): 319.3032 Found: 319.2040.

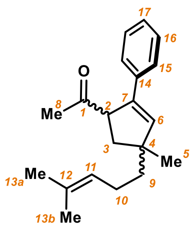

1-(4-Methyl-4-(4-methylpent-3-en-1-yl)-2-phenylcyclopent-2-en-1-yl)ethan-1-one (2x)

Prepared by following the General Procedure 3b from **1x** (36.0 mg, 0.20 mmol, 1.0 eq), phenylacetylene (66 µL, 0.60 mmol, 3.0 eq), Sm metal (5 mg, 0.03 mmol, 15 mol%), and SmI_2 (300 µL, 0.1 M, 15 mol%). The crude residue was purified by column chromatography R_f = 0.20 (silica gel, petroleum ether:Et_2O = 9:1) to afford the target product **2x** as a yellow oil and as a mixture of diastereoisomers (46.4 mg, 0.164 mmol, 82% yield, d.r. 1:1).

^{1}H NMR (400 MHz, $CDCl_3$) δ 7.45 – 6.96 (m, 10H, *5H-Ar* + *5H-Ar'*), 6.15 (d, *J* = 1.9 Hz, 1H, *H-6*), 6.13 (d, *J* = 1.8 Hz, 1H, *H-6'*), 5.17 – 5.12 (m, 1H, *H-11*), 5.10 – 5.03 (m, 1H, *H-11'*), 4.17 – 4.04 (m, 2H, *H-2* + *H-2'*), 2.31 (dd, *J* = 13.6, 9.7 Hz, 1H, *H-3a*), 2.15 – 1.91 (m, 12H, 2*H-3* + *2CH*$_2$ + *2CH*$_3$), 1.79 (dd, *J* = 13.5, 6.4 Hz, 1H, *H-3b*), 1.71 – 1.58 (m, 12H, *4CH*$_3$), 1.54 – 1.40 (m, 4H, *2CH*$_2$), 1.23 (s, 3H, *CH*$_3$), 1.13 (s, 3H, *CH*$_3$).

^{13}C{H} NMR (101 MHz, $CDCl_3$) δ 211.3 (*C-1* + *C-1' two overlapping signals*), 140.0 (*C-6*), 139.5 (*C-6'*), 139.3 (*C-14*), 139.1 (*C-14*), 135.9 (*C-7*), 135.7 (*C-7*), 131.7 (*C-12*), 131.6 (*C-12'*), 128.74 (*C-ArH*),

128.71 (*C-ArH*), 127.60 (*C-ArH*), 125.9 (*C-ArH*), 125.8 (*C-ArH*), 124.74 (*C-11*), 124.67 (*C-11*), 60.1 (*C-2*), 59.4 (*C-2'*), 49.1 (*C-4*), 48.9 (*C-4*), 42.4 (*CH_2*), 41.9 (*CH_2*), 40.9 (*CH_2*), 40.8 (*CH_2*), 27.5 (*CH_3*), 26.6 (*CH_3*), 26.47 (*CH_3*), 26.43 (*CH_3*), 25.9 (*CH_3*), 25.8 (*CH_3*), 24.2 (*CH_2*), 23.9 (*CH_2*), 17.82 (*CH_3*), 17.79 (*CH_3*).

IR (neat, cm^{-1}): 2902, 2849, 1694, 1495, 1449 (fingerprint region excluded).

HRMS (ESI+): calculated for $C_{20}H_{26}ONa$ (M^+Na^+): 305.1876 Found: 305.1870.

3,3,8-Trimethyl-1-phenylspiro[4.5]dec-1-en-6-one (2y)

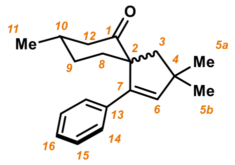

Prepared by following the General Procedure 3b from **1y** (33.2 mg, 0.20 mmol, 1.0 eq), phenylacetylene (66 µL, 0.60 mmol, 3.0 eq), Sm metal (10 mg, 0.06 mmol, 30 mol%), and SmI_2 (300 µL, 0.1 M, 15 mol%). The crude residue was purified by column chromatography R_f = 0.15 (silica gel, petroleum ether:Et_2O = 19:1) to afford the target product **2y** as a pale-yellow oil and as a mixture of diastereoisomers (32.6 mg, 0.121 mmol, 61% yield, d.r. = 1:1).

^{1}H NMR (500 MHz, $CDCl_3$) δ 7.34 – 7.15 (m, 10H, *5H-Ar* + *5H-Ar*), 5.99 (s, 1H, *H-6*), 5.91 (s, 1H, *H-6*), 2.83 (dd, *J* = 14.9, 5.9 Hz, 1H), 2.50 (dd, *J* = 4.2, 2.3 Hz, 1H), 2.42 – 2.25 (m, 5H), 2.22 – 2.06 (m, 3H), 1.96 (dt, *J* = 13.2, 6.2 Hz, 4H), 1.79 – 1.70 (m, 1H), 1.66 (dt, *J* = 13.8, 3.2 Hz, 1H), 1.57 – 1.36 (m, 3H), 1.19 – 1.17 (m, 6H, *3H-5a* + *3H-5a*), 1.11 – 1.03 (m, 6H, *3H-5b* + *3H-5b*), 1.06 – 0.99 (m, 6H, *3H-11* + *3H-11*).

^{13}C{H} NMR (126 MHz, $CDCl_3$) δ 215.0 (*C-1*), 214.0 (*C-1*), 143.3 (*C-7*), 142.6 (*C-7*), 141.0 (*C-6*), 140.7 (*C-6*), 136.5 (*C-13*), 135.9 (*C-13*), 128.20 (*C-ArH*), 128.18 (*C-ArH*), 128.09 (*C-ArH*), 128.07 (*C-ArH*), 127.09 (*C-ArH*), 127.01 (*C-ArH*), 65.5 (*C-2*), 64.4 (*C-2*), 53.1 (*C-3*), 52.9 (*C-3*), 48.1 (*C-6*), 46.2 (*C-6*), 44.0 (*C-4*), 43.9 (*C-4*), 37.6 (*C-8*), 33.9 (*C-10*), 33.5 (*C-8*), 30.9 (*C-5a*), 30.8 (*C-9*), 30.8 (*C-10*), 29.2 (*C-5b*), 29.1 (*C-5b*), 27.8 (*C-9*), 22.6 (*C-11*), 19.8 (*C-11*).

IR (neat, cm^{-1}): 2953, 2927, 2864, 1700, 1492, 1455, 1442 (fingerprint region excluded).

HRMS (ESI+): calculated for $C_{19}H_{24}ONa$ (M^+Na^+): 291.1719 Found: 291.1730.

1,1,6-Trimethyl-3-phenyloctahydro-4*H*-inden-4-one (4a)

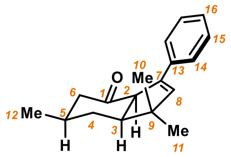

Prepared by following the General Procedure 3a from **3a** (16 µL, 0.10 mmol, 1.0 eq), phenylacetylene (33 µL, 0.30 mmol, 3.0 eq), and SmI_2 (150 µL, 0.1 M, 15 mol%). The crude residue was purified by column chromatography R_f = 0.30 (silica gel, petroleum ether:Et_2O = 9:1) to afford the target product as a colorless oil (20 mg, 0.078 mmol, 78% yield). On standing the oil solidified and was recrystallised from petroleum ether to afford colorless crystals (19 mg, 0.075 mmol, 75% yield). The reaction was carried out on larger-scale using from **4a** (160 µL, 1.0 mmol, 1.0 eq), phenylacetylene (330 µL, 3.0 mmol, 3.0 eq), and SmI_2 (1.5 mL, 0.1 M, 15 mol%) and obtained colorless crystals (232 mg, 0.81 mmol, 81% yield).

^{1}H NMR: (400 MHz, $CDCl_3$) δ 7.38 - 7.31 (m, 2H, *2H-14*), 7.29 - 7.24 (m, 2H, *2H-15*), 7.23 - 7.17 (m, 1H, *H-16*), 5.98 (d, *J* = 2.3 Hz, 1H, *H-8*), 3.99 (ddd, *J* = 8.0 Hz, 1H, *H-2*), 2.53 (ddd, *J* = 11.9, 8.0, 6.6 Hz, 1H, *H-3*), 2.35 (dddd, *J* = 14.6, 3.9, 2.3, 1.2 Hz, 1H, *H-6a*), 2.05 - 1.89 (m, 1H, *H-5*), 1.88 - 1.78 (m, 1H, *H-4a*), 1.77 - 1.67 (m, 1H, *H-6b*), 1.37 - 1.21 (q, *J* = 12.9 Hz, 1H, *H-4b*), 1.12 (s, 3H, *3H-11*), 1.05 (s, 3H, *3H-10*), 1.02 (d, *J* = 6.5 Hz, 3H, *3H-12*).

^{13}C{H} NMR: (101 MHz, $CDCl_3$) δ 213.9 (*C-1*), 139.7 (*C-8*), 138.3 (*C-7*), 136.0 (*C-13*), 128.4 (*C-14*), 127.4 (*C-16*), 126.2 (*C-15*), 58.5 (*C-2*), 52.1 (*C-3*), 49.0 (*C-6*), 46.9 (*C-9*), 33.1 (*C-4*), 31.9 (*C-5*), 26.8 (*C-11*), 23.3 (*C-10*), 22.4 (*C-12*).

Melting Point (from petroleum ether: Et_2O): 60 – 63 °C.

IR (neat, cm^{-1}): 2955, 2927, 2869, 1704, 1494, 1457 (fingerprint region excluded).

HRMS (ESI+): calculated for $C_{18}H_{22}ONa$ (M^+Na^+): 277.1563 Found: 277.1559.

1,1,6-Trimethyl-3-(p-tolyl)octahydro-4*H*-inden-4-one (4b)

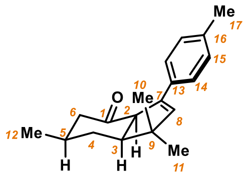

Prepared by following the General Procedure 3a from **3a** (16 µL, 0.10 mmol, 1.0 eq), 4-methylphenylacetylene (38 µL, 0.30 mmol, 3.0 eq), and SmI_2 (150 µL, 0.1 M, 15 mol%). The crude residue was purified by column chromatography R_f = 0.45 (silica gel, petroleum ether:Et_2O = 9:1) to afford the target product **4b** as a colorless oil (21 mg, 0.078 mmol, 78% yield).

^{1}H NMR (400 MHz, $CDCl_3$) δ 7.23 (d, *J* = 8.2 Hz, 2H, 2*H-15*), 7.08 (dt, *J* = 7.9, 0.7 Hz, 2H, 2*H-14*), 5.91 (d, *J* = 2.3 Hz, 1H, *H-8*), 3.97 (ddd, *J* = 7.8, 2.4, 1.2 Hz, 1H, *H-2*), 2.56 - 2.45 (m, 1H, *H-3*), 2.38 - 2.25 (m, 4H, *H-6a* + *H-17*), 2.06 - 1.89 (m, 1H, *H-5*), 1.89 - 1.78 (m, 1H, *H-6b*), 1.73 (ddt, *J* = 13.3, 6.6, 2.5

Hz, 1H, *H-4a*), 1.36 - 1.23 (m, 1H, *H-4b*), 1.10 (s, 3H, 3*H-11*), 1.05 (s, 3H, 3*H-10*), 1.01 (d, *J* = 6.4 Hz, 3H, 3*H-12*).

^{13}C{H} NMR (101 MHz, $CDCl_3$) δ 214.0 (*C-1*), 138.7 (*C-7*), 138.2 (*C-8*), 137.1 (*C-13*), 133.1 (*C-16*), 129.1 (*C-15*), 126.1 (*C-14*), 58.6 (*C-2*), 52.2 (*C-3*), 49.0 (*C-6*), 46.9 (*C-9*), 33.1 (*C-4*), 32.0 (*C-5*), 26.8 (*C-11*), 23.3 (*C-10*), 22.4 (*C-12*), 21.3 (*C-17*).

IR (neat, cm^{-1}): 3026, 2952, 2924, 2867, 1707, 1655, 1569, 1513, 1454, 1422 (fingerprint region excluded).

HRMS (ESI+): calculated for $C_{19}H_{24}ONa$ ($M^{+}Na^{+}$): 291.1719 Found: 291.1714.

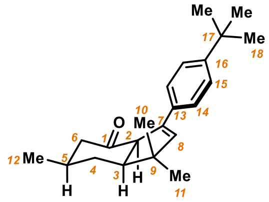

3-(4-(*tert*-Butyl)phenyl)-1,1,6-trimethyloctahydro-4*H*-inden-4-one (4c)

Prepared by following the General Procedure 3a from **3a** (16 µL, 0.10 mmol, 1.0 eq), 4-*tert*-butyl phenylacetylene (54 µL, 0.30 mmol, 3.0 eq), and SmI_2 (150 µL, 0.1 M, 15 mol%). The crude residue was purified by column chromatography R_f = 0.45 (silica gel, petroleum ether:Et_2O = 9:1) to afford the target product **4c** as a colorless oil (26 mg, 0.082 mmol, 82% yield).

^{1}H NMR (400 MHz, $CDCl_3$) δ 7.34 - 7.21 (m, 4H, 2*H-14* + 2*H-15*), 5.94 (d, *J* = 2.3 Hz, 1H, *H-8*), 3.97 (ddd, *J* = 7.9, 2.3, 1.2 Hz, 1H, *H-2*), 2.51 (ddd, *J* = 11.9, 7.9, 6.5 Hz, 1H, *H-3*), 2.40 - 2.30 (m, 1H, *H-6a*), 2.03 - 1.92 (m, 1H, *H-5*), 1.84 (dd, *J* = 14.3, 12.4 Hz, 1H, *H-6b*), 1.74 (ddt, *J* = 13.3, 6.6, 2.5 Hz, 1H, *H-4a*), 1.29 (s, 10H, *H-4b* + 9*H-18*), 1.10 (s, 3H, 3*H-11*), 1.05 (s, 3H, 3*H-10*), 1.01 (d, *J* = 6.4 Hz, 3H, 3*H-12*).

^{13}C{H} NMR (101 MHz, $CDCl_3$) δ 214.1 (*C-1*), 150.4 (*C-16*), 139.0 (*C-8*), 138.0 (*C-7*), 133.1 (*C-13*), 125.9 (*C-14*), 125.4 (*C-15*), 58.5 (*C-2*), 52.2 (*C-3*), 49.0 (*C-6*), 46.9 (*C-9*), 34.6 (*C-17*), 33.1 (*C-4*), 32.0 (*C-5*), 31.4 (*C-18*), 26.8 (*C-11*), 23.3 (*C-10*), 22.4 (*C-12*).

IR (neat, cm^{-1}): 2953, 2927, 2904, 2867, 1707, 1456 (fingerprint region excluded).

HRMS (ESI+): calculated for $C_{22}H_{30}O$ ($M^{+}Na^{+}$): 310.2297 Found: 310.2309.

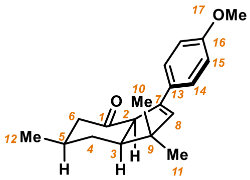

3-(4-Methoxyphenyl)-1,1,6-trimethyloctahydro-4*H*-inden-4-one (4d)

Prepared by following the General Procedure 3a from **3a** (16 µL, 0.10 mmol, 1.0 eq), 4-ethynylanisole (40 mg, 0.30 mmol, 3.0 eq), and SmI_2 (150 µL, 0.1 M, 15 mol%). The crude residue was purified by column chromatography R_f = 0.45 (silica gel, petroleum ether:Et_2O = 7:3) to afford the target product **4d** as a colorless oil (19 mg, 0.067 mmol, 67% yield).

^{1}H NMR (500 MHz, $CDCl_3$) δ 7.27 (d, *J* = 8.8 Hz, 2H, *H-15*), 6.81 (d, *J* = 8.8 Hz, 2H, *H-14*), 5.85 (d, *J* = 2.3 Hz, 1H, *H-8*), 3.95 (ddd, *J* = 7.8, 2.3, 1.2 Hz, 1H, *H-2*), 3.78 (s, 3H, *H-17*), 2.50 (ddd, *J* = 11.9, 7.9, 6.5 Hz, 1H, *H-3*), 2.38 - 2.30 (m, 1H, *H-6a*), 2.02 - 1.90 (m, 1H, *H-5*), 1.81 (dd, *J* = 14.6, 12.4 Hz, 1H, *H-6b*), 1.74 (ddt, *J* = 13.3, 6.6, 2.5 Hz, 1H, *H-4a*), 1.35 - 1.22 (m, 1H, *H-4b*), 1.10 (s, 3H, 3*H-11*), 1.04 (s, 3H, 3*H-10*), 1.01 (d, *J* = 6.4 Hz, 3H, *3H-12*).

^{13}C{H} NMR (126 MHz, $CDCl_3$) δ 214.2 (*C-1*), 159.0 (*C-16*), 137.8 (*C-7*), 137.7 (*C-8*), 128.7 (*C-13*), 127.5 (*C-15*), 113.8 (*C-14*), 58.6 (*C-2*), 55.4 (*C-17*), 52.2 (*C-3*), 49.0 (*C-6*), 46.9 (*C-9*), 33.1 (*C-4*), 32.0 (*C-5*), 26.9 (*C-11*), 23.3 (*C-10*), 22.4 (*C-12*).

IR (neat, cm^{-1}): 2953, 2927, 2868, 1705, 1607, 1511, 1455 (fingerprint region excluded).

HRMS (ESI+): calculated for $C_{19}H_{24}O_2Na$ (M^+Na^+): 307.1669 Found: 307.1663.

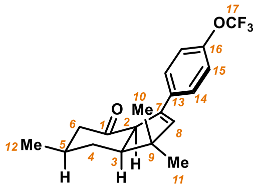

1,1,6-Trimethyl-3-(4-(trifluoromethoxy)phenyl)octahydro-4*H*-inden-4-one (4e)

Prepared by following the General Procedure 3a from **3a** (16 µL, 0.10 mmol, 1.0 eq), 4-trifluoromethoxyphenylacetylene (56 mg, 0.30 mmol, 3.0 eq), and SmI_2 (150 µL, 0.1 M, 15 mol%). The crude residue was purified by column chromatography R_f = 0.40 (silica gel, petroleum ether:Et_2O = 9:1) to afford the target product **4e** as a colorless oil (33 mg, 0.096 mmol, 96% yield).

^{1}H NMR (400 MHz, $CDCl_3$) δ 7.40 - 7.30 (m, 2H, 2*H-15*), 7.11 (dt, *J* = 7.9, 1.0 Hz, 2H, 2*H-14*), 5.99 (d, *J* = 2.2 Hz, 1H, *H-8*), 3.95 (ddd, *J* = 8.2, 2.2, 1.1 Hz, 1H, *H-2*), 2.56 (ddd, *J* = 11.8, 8.3, 6.7 Hz, 1H, *H-3*), 2.44 - 2.35 (m, 1H, *H-6a*), 2.00 (dddd, *J* = 12.3, 10.5, 6.1, 3.4 Hz, 1H, *H-5*), 1.86 - 1.71 (m, 2H, *H-6b* + *H-4a*), 1.35 - 1.19 (m, 1H, *H-4b*), 1.12 (s, 3H, 3*H-10*), 1.05 – 1.01 (m, 6H, 3*H-11* + 3*H-12*).

^{13}C{H} NMR (101 MHz, $CDCl_3$) δ 213.6 (*C-1*), 148.4 (q, *J* = 1.8 Hz, *C-16*), 140.8 (*C-8*), 136.9 (*C-7*), 134.8 (*C-13*), 127.6 (*C-15*), 120.9 (*C-14*), 120.6 (q, *J* = 257.3 Hz, *C-17*), 58.4 (*C-2*), 51.8 (*C-3*), 48.9 (*C-6*), 47.1 (*C-9*), 32.9 (*C-4*), 31.6 (*C-5*), 26.9 (*C-11*), 23.3 (*C-10*), 22.3 (*C-12*).

^{19}F NMR (471 MHz, $CDCl_3$) δ -57.83.

IR (neat, cm^{-1}): 2956, 2929, 2870, 1707, 1508, 1456 (fingerprint region excluded).

HRMS (ESI-): calculated for $C_{19}H_{21}O_2F_3$ (M^+H^+): 338.1489 Found: 337.1417.

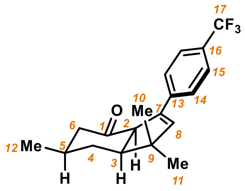

1,1,6-Trimethyl-3-(4-(trifluoromethyl)phenyl)octahydro-4*H*-inden-4-one (4f)

Prepared by following the General Procedure 3a from **3a** (16 µL, 0.10 mmol, 1.0 eq), 4-trifluoromethylphenylacetylene (49 µL, 0.30 mmol, 3.0 eq), and SmI_2 (150 µL, 0.1 M, 15 mol%). The crude residue was purified by column chromatography R_f = 0.45 (silica gel, petroleum ether:Et_2O = 9:1) to afford the target product **4f** as a colorless oil (27 mg, 0.082 mmol, 82% yield).

^{1}H NMR (400 MHz, $CDCl_3$) δ 7.52 (dt, *J* = 8.1, 0.8 Hz, 2H, *H-14*), 7.46 - 7.39 (m, 2H, *H-15*), 6.10 (d, *J* = 2.2 Hz, 1H, *H-8*), 3.98 (ddd, *J* = 8.4, 2.3, 1.1 Hz, 1H, *H-2*), 2.53 (ddd, *J* = 11.9, 8.0, 6.6 Hz, 1H, *H-3*), 2.40 (dddd, *J* = 15.2, 4.4, 2.3, 1.1 Hz, 1H, *H-6a*), 2.08 - 1.94 (m, 1H, *H-5*), 1.85 - 1.72 (m, 2H, *H-6b* and *H-4a*), 1.32 - 1.21 (m, 1H, *H-4b*), 1.13 (s, 3H, 3*H-11*), 1.05 (s, 3H, 3*H-10*), 1.03 (d, *J* = 6.6 Hz, 3H, 3*H-12*).

^{13}C{H} NMR (101 MHz, $CDCl_3$) δ 213.3 (*C-1*), 142.3 (*C-8*), 139.5 (*C-7*), 137.1 (*C-13*), 129.2 (q, *J* = 32.5 Hz, *C-16*), 126.5 (*C-14*), 125.3 (q, *J* = 3.9 Hz, *C-15*), 124.3 (q, *J* = 265.3 Hz, *C-17*), 58.2 (*C-2*), 51.6 (*C-3*), 48.8 (*C-6*), 47.2 (*C-9*), 32.8 (*C-4*), 31.5 (*C-5*), 26.9 (*C-11*), 23.3 (*C-10*), 22.3 (*C-12*).

^{19}F NMR (376 MHz, $CDCl_3$) δ -62.53.

IR (neat, cm^{-1}): 2956, 2929, 2870, 1706, 1616, 1575, 2457, 1411 (fingerprint region excluded).

HRMS (ESI+): calculated for $C_{19}H_{21}OF_3Na$ (M^+Na^+): 345.1437 Found: 345.1434.

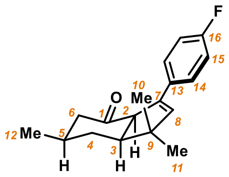

3-(4-Fluorophenyl)-1,1,6-trimethyloctahydro-4*H*-inden-4-one (4g)

Prepared by following the General Procedure 3a from **3a** (16 µL, 0.10 mmol, 1.0 eq), 4-fluorophenylacetylene (34 µL, 0.30 mmol, 3.0 eq), and SmI_2 (150 µL, 0.1 M, 15 mol%). The crude residue was purified by column chromatography R_f = 0.45 (silica gel, petroleum ether:Et_2O = 9:1) to afford the target product **4g** a colorless oil (19 mg, 0.070 mmol, 70% yield).

^{1}H NMR (400 MHz, $CDCl_3$) δ 7.35 - 7.25 (m, 2H, 2*H-14*), 6.95 (t, *J* = 8.8 Hz, 2H, *2H-15*), 5.91 (d, *J* = 2.3 Hz, 1H, *H-8*), 3.94 (ddd, *J* = 8.1, 2.2, 1.1 Hz, 1H, *H-2*), 2.54 (ddd, *J* = 11.9, 8.1, 6.7 Hz, 1H, *H-3*), 2.46 - 2.31 (m, 1H, *H-6a*), 1.99 (tddd, *J* = 12.4, 6.5, 4.1, 2.5 Hz, 1H, *H-5*), 1.90 - 1.70 (m, 2H, *H-4a H-6b*), 1.30 - 1.18 (m, 1H, *H-4b*), 1.11 (s, 3H, 3*H-11*), 1.05 - 0.98 (m, 6H, 3*H-10* + *3H-12*).

^{13}C{H} NMR (101 MHz, $CDCl_3$) δ 213.8 (*C-1*), 162.2 (d, *J* = 246.5 Hz, *C-16*), 139.4 (*C-7*), 137.2 (*C-8*), 132.1 (d, *J* = 3.4 Hz, *C-13*), 127.9 (d, *J* = 7.9 H, *C-14*), 115.3 (d, *J* = 21.4 Hz, *C-15*), 58.5 (*C-2*), 51.9 (*C-3*), 48.9 (*C-6*), 47.0 (*C-9*), 33.0 (*C-4*), 31.7 (*C-5*), 26.9 (*C-11*), 23.3 (*C-10*), 22.4 (*C-12*).

^{19}F NMR (376 MHz, $CDCl_3$) δ -115.01.

IR (neat, cm^{-1}): 2954, 2927, 2868, 1707, 1508, 1455 (fingerprint region excluded).

HRMS (ESI+): calculated for $C_{18}H_{21}OFNa$ (M^+Na^+): 272.1576 Found: 272.1587.

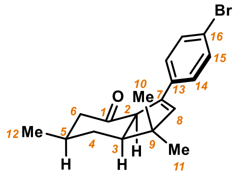

3-(4-Bromophenyl)-1,1,6-trimethyloctahydro-4*H*-inden-4-one (4h)

Prepared by following the General Procedure 3a from **3a** (16 µL, 0.10 mmol, 1.0 eq), 4-ethynylbromobenzene (54 mg, 0.30 mmol, 3.0 eq), and SmI_2 (150 µL, 0.1 M, 15 mol%). The crude residue was purified by column chromatography R_f = 0.25 (silica gel, petroleum ether:Et_2O = 9:1) to afford the target product **4h** as a colorless oil (26 mg, 0.078 mmol, 78% yield).

^{1}H NMR (500 MHz, $CDCl_3$) δ 7.38 (d, *J* = 8.5 Hz, 2H, 2*H-14*), 7.20 (d, *J* = 8.5 Hz, 2H, *2H-15*), 5.99 (d, *J* = 2.2 Hz, 1H, *H-8*), 3.94 (ddd, *J* = 8.2, 2.2, 1.1 Hz, 1H, *H-2*), 2.54 (ddd, *J* = 11.9, 8.2, 6.7 Hz, 1H, *H-3*), 2.37 (dddd, *J* = 15.0, 4.1, 2.4, 1.1 Hz, 1H, *H-6a*), 2.06 - 1.92 (m, 1H, *H-5*), 1.83 - 1.71 (m, 2H, *H-4a* + *H-6b*), 1.34 - 1.19 (m, 1H, *H-4b*), 1.11 (s, 3H, *3H-11*), 1.06 - 0.98 (m, 6H, *3H-10* + *3H-12*).

^{13}C NMR (126 MHz, $CDCl_3$) δ 213.6 (*C-1*), 140.6 (*C-8*), 137.2 (*C-7*), 134.9 (*C-13*), 131.5(*C-14*), 127.9 (*C-15*), 121.3 (*C-16*), 58.3 (*C-2*), 51.8 (*C-3*), 48.9 (*C-6*), 47.1 (*C-9*), 32.9 (*C-4*), 31.6 (*C-5*), 26.8 (*C-11*), 23.3 (*C-10*), 22.3 (*C-12*).

IR (neat, cm^{-1}): 2952, 2925, 2866, 1704, 1488, 1454 (fingerprint region excluded).

HRMS (ESI+): calculated for $C_{18}H_{21}OBrNa$ ($M^{+}Na^{+}$): 332.0776 Found: 332.0779.

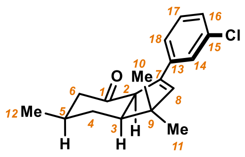

3-(3-Chlorophenyl)-1,1,6-trimethyloctahydro-4*H*-inden-4-one (4i)

Prepared by following the General Procedure 3a from **3a** (16 µL, 0.10 mmol, 1.0 eq), 3-chlorophenylacetylene (37 µL, 0.30 mmol, 3.0 eq), and SmI_2 (150 µL, 0.1 M, 15 mol%). The crude residue was purified by column chromatography R_f = 0.45 (silica gel, petroleum ether:Et_2O = 9:1) to afford the target product **4i** as a colorless oil (26 mg, 0.080 mmol, 80% yield).

^{1}H NMR (400 MHz, $CDCl_3$) δ 7.34 (dt, *J* = 2.5, 1.1 Hz, 1H, *H-14*), 7.24 - 7.13 (m, 3H, *H-16* + *H-17* + *H-18*), 6.03 (d, *J* = 2.2 Hz, 1H, *H-8*), 3.94 (ddd, *J* = 8.4, 2.2, 1.1 Hz, 1H, *H-2*), 2.56 (ddd, *J* = 11.9, 8.4, 6.8 Hz, 1H, *H-3*), 2.45 - 2.35 (m, 1H, *H-6a*), 2.11 - 1.97 (m, 1H, *H-5*), 1.86 - 1.71 (m, 2H, *H-4a* + *H-6b*), 1.25 (q, *J* = 12.5 Hz, 1H, *H-4b*), 1.12 (s, 3H, 3*H-11*), 1.04 – 1.00 (m, 6H, 3*H-10* + 3*H-12*).

^{13}C{H} NMR (101 MHz, $CDCl_3$) δ 213.4 (*C-1*), 141.3 (*C-8*), 137.9 (*C-7*), 137.0 (*C-13*), 134.3 (*C-15*), 129.7 (*C-16*), 127.4 (*C-14*), 126.4 (*C-18*), 124.4 (*C-17*), 58.2 (*C-2*), 51.7 (*C-3*), 48.9 (*C-6*), 47.0 (*C-9*), 32.8 (*C-4*), 31.5 (*C-5*), 26.9 (*C-11*), 23.3 (*C-10*), 22.3 (*C-12*).

IR (neat, cm^{-1}): 2953, 2926, 2867, 1706, 1593, 1562, 1455 (fingerprint region excluded).

HRMS (ESI+): calculated for $C_{18}H_{21}OClNa$ ($M^{+}Na^{+}$): 288.1281 Found: 288.1287.

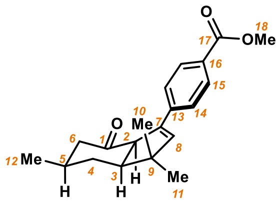

Methyl 4-(3,3,5-trimethyl-7-oxooctahydro-1H-inden-1-yl)benzoate (4j)

Prepared by following the General Procedure 3a from **3a** (16 µL, 0.10 mmol, 1.0 eq), methyl 4-ethynylbenzoate (48 mg, 0.30 mmol, 3.0 eq), and SmI_2 (150 µL, 0.1 M, 15 mol%). The crude residue was purified by column chromatography R_f = 0.40 (silica gel, petroleum ether:Et_2O = 7:3) to afford the target product **4j** as an amber oil (17 mg, 0.053 mmol, 53% yield).

^{1}H NMR (500 MHz, $CDCl_3$) δ 7.97 – 7.90 (m, 2H, 2*H-15*), 7.42 – 7.36 (m, 2H, 2*H-14*), 6.14 (d, J = 2.1 Hz, 1H, *H-8*), 4.02 – 3.96 (m, 1H, *H-2*), 3.89 (s, 3H, 3*H-18*), 2.58 (ddd, J = 11.9, 8.4, 6.8 Hz, 1H, *H-3*), 2.44 – 2.34 (m, 1H, *H-6a*), 2.07 – 1.96 (m, 5H, *H-5*), 1.85 – 1.73 (m, 2H, *H-4a* + *H-6b*), 1.33 - 1.19 (m, 1H, *H-4b*), 1.14 (s, 3H, 3*H-11*), 1.06 – 0.99 (m, 6H, 3*H-10* + 3*H-12*).

^{13}C{H} NMR (101 MHz, $CDCl_3$) δ 213.4 (*C-1*), 167.1 (*C-17*), 142.4 (*C-8*), 140.5 (*C-7*), 137.4 (*C-13*), 129.8 (*C-15*), 128.8 (*C-16*), 126.2 (*C-14*), 58.2 (*C-2*), 52.3 (*C-18*), 51.6 (*C-3*), 48.8 (*C-6*), 47.2 (*C-9*), 32.7 (*C-4*), 31.5 (*C-5*), 26.9 (*C-11*), 23.3 (*C-10*), 22.3 (*C-12*).

IR (neat, cm^{-1}): 2954, 2927, 2870, 1702, 1607, 1564, 1456, 1436, 1411 (fingerprint region excluded).

HRMS (ESI+): calculated for $C_{20}H_{24}O_2Na$ (M^+Na^+): 335.1618 Found: 335.1604.

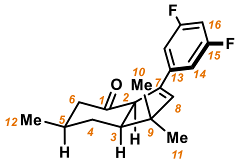

3-(3,5-Difluorophenyl)-1,1,6-trimethyloctahydro-4*H*-inden-4-one (4k)

Prepared by following the General Procedure 3a from **3a** (16 µL, 0.10 mmol, 1.0 eq), 3,5-difluorophenylacetylene (37 µL, 0.30 mmol, 3.0 eq), and SmI_2 (150 µL, 0.1 M, 15 mol%). The crude residue was purified by column chromatography R_f = 0.50 (silica gel, petroleum ether:Et_2O = 9:1) to afford the target product **4k** as a colorless oil (27 mg, 0.091 mmol, 91% yield).

^{1}H NMR (400 MHz, $CDCl_3$) δ 6.89 - 6.79 (m, 2H, 2*H-14*), 6.64 (tt, *J* = 8.9, 2.3 Hz, 1H, *H-16*), 6.07 (d, *J* = 2.0 Hz, 1H, *H-8*), 3.92 - 3.85 (m, 1H, *H-2*), 2.59 (ddd, *J* = 11.8, 8.7, 7.0 Hz, 1H, *H-3*), 2.50 - 2.39 (m, 1H, *H-6a*), 2.15 - 1.97 (m, 1H, *H-5*), 1.87 - 1.72 (m, 2H, *H-4a* + *H-6b*), 1.29 - 1.15 (m, 1H, *H-4b*), 1.12 (s, 3H, *3H-10*), 1.07 - 0.98 (m, 6H, *3H-11* + *3H-12*).

^{13}C{H} NMR (126 MHz, $CDCl_3$) δ 213.1 (*C-1*), 163.1 (dd, *J* = 247.3, 13.1 Hz, *C-15*), 142.5 (*C-8*), 139.3 (*C-13*), 136.3 (*C-7*), 109.1 (d, *J* = 25.9 Hz, *C-14*), 102.6 (t, *J* = 25.5 Hz, *C-16*), 58.0 (*C-2*), 51.3 (*C-3*), 48.8 (*C-6*), 47.1 (*C-9*), 32.6 (*C-4*), 31.2 (*C-5*), 27.0 (*C-11*), 23.4 (*C-10*), 22.2 (*C-12*).

^{19}F NMR (471 MHz, $CDCl_3$) δ -110.40 (t, *J* = 8.1 Hz).

IR (neat, cm^{-1}): 2955, 2928, 2869, 1707, 1621, 1588, 1441 (fingerprint region excluded).

HRMS (ESI-): calculated for $C_{18}H_{20}F_2O$ (M^+H^+): 290.1482 Found: 290.1474.

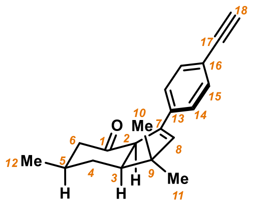

3-(4-Ethynylphenyl)-1,1,6-trimethyloctahydro-4*H*-inden-4-one (4l)

Prepared by following the General Procedure 3a from **3a** (16 µL, 0.10 mmol, 1.0 eq), 4-ethynylphenylacetylene (39 mg, 0.30 mmol, 3.0 eq), and SmI_2 (150 µL, 0.1 M, 15 mol%). The crude residue was purified by column chromatography R_f = 0.30 (silica gel, petroleum ether:Et_2O = 9:1) to afford the target product **4l** as a yellow oil (20 mg, 0.073 mmol, 73% yield).

^{1}H NMR (400 MHz, $CDCl_3$) δ 7.42 - 7.35 (m, 2H, 2*H-15*), 7.32 - 7.24 (m, 2H, 2*H-14*), 6.04 (d, *J* = 2.2 Hz, 1H, *H-8*), 3.95 (d, *J* = 8.3 Hz, 1H, *H-2*), 3.10 - 3.05 (s, 1H, *H-18*), 2.61 - 2.49 (m, 1H, *H-3*), 2.38 (d, *J* = 15.6 Hz, 1H, *H-6a*), 1.99 (m, 1H, *H-5*), 1.86 - 1.70 (m, 2H, *H-6b and H-4a*), 1.33 - 1.14 (m, 1H, *H-4b*), 1.12 (d, *J* = 1.9 Hz, 3H, 3*H-11*), 1.05 - 0.98 (m, 6H, 3*H-10 and 3H-12*).

^{13}C{H} NMR (126 MHz, $CDCl_3$) δ 213.6 (*C-1*), 141.1 (*C-8*), 137.6 (*C-7*), 136.5 (*C-13*), 132.2 (*C-15*), 126.1 (*C-14*), 120.9 (*C-16*), 83.9 (*C-17*), 77.7 (*C-18*), 58.3 (*C-2*), 51.8 (*C-3*), 48.9 (*C-6*), 47.1 (*C-9*), 32.9 (*C-4*), 31.7 (*C-5*), 26.9 (*C-11*), 23.3 (*C-10*), 22.3 (*C-12*).

IR (neat, cm^{-1}): 3291, 2953, 2926, 2867, 2096, 1704, 1506, 1455, 1408 (fingerprint region excluded).

HRMS (ESI+): calculated for $C_{20}H_{22}ONa$ (M^+Na^+): 278.1671 Found: 278.1669.

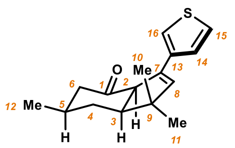

1,1,6-Trimethyl-3-(thiophen-3-yl)octahydro-4*H*-inden-4-one (4m)

Prepared by following the General Procedure 3b from **3a** (32 µL, 0.20 mmol, 1.0 eq), 3-ethynylthiophene (60 µL, 0.60 mmol, 3.0 eq), Sm metal (5.0 mg, 15 mol%), and SmI_2 (150 µL, 0.1 M, 15 mol%). The crude residue was purified by column chromatography R_f = 0.25 (silica gel, petroleum ether:Et_2O = 9:1) to afford the target product **4m** as a pale-yellow waxy solid (37.0 mg, 0.142 mmol, 71% yield).

***Note: 4m** appears to be light/temperature sensitive (yellow solid turns brown) and decomposition was observed during acquisition of the 2D-NMR spectra.*

^{1}H NMR (400 MHz, $CDCl_3$) δ 7.23 (dd, *J* = 5.1, 2.9 Hz, 1H, *H-15*), 7.17 (dd, *J* = 5.1, 1.3 Hz, 1H, *H-16*), 7.09 (dd, *J* = 2.9, 1.3 Hz, 1H, *H-14*), 5.89 (d, *J* = 2.4 Hz, 1H, *H-8*), 3.88 (dt, *J* = 7.6, 2.0 Hz, 1H, *H-2*), 2.48 (ddd, *J* = 11.9, 7.7, 6.4 Hz, 1H, *H-3*), 2.42 – 2.34 (m, 1H, *H-6a*), 2.02 – 1.82 (m, 2H, *H-5, H-6b*), 1.79 – 1.67 (m, 1H, *H-4a*), 1.36 – 1.22 (m, 1H, *H-4b*), 1.09 (s, 3H, *3H-10*), 1.06 (s, 3H, *3H-11*), 1.02 (d, *J* = 6.0 Hz, 3H, *3H-12*).

^{13}C{H} NMR (126 MHz, $CDCl_3$) δ 214.5 (*C-1*), 138.7 (*C-8*), 137.5 (*C-13*), 133.2 (*C-7*), 126.3 (*C-15*), 125.5 (*C-16*), 121.0 (*C-14*), 59.0 (*C-2*), 52.3 (*C-3*), 49.0 (*C-6*), 46.9 (*C-9*), 33.2 (*C-4*), 32.0 (*C-5*), 26.7 (*C-10*), 23.2 (*C-11*), 22.4 (*C-12*).

Melting Point (from petroleum ether: Et_2O): 75 – 80 °C (decomposed).

IR (neat, cm^{-1}): 2952, 2925, 2868, 1703, 1455 (fingerprint region excluded).

HRMS (APCI+): calculated for $C_{16}H_{21}OS$ (M^+H^+): 261.1308 Found: 261.1315.

3-Mesityl-1,1,6-trimethyloctahydro-4*H*-inden-4-one (4n)

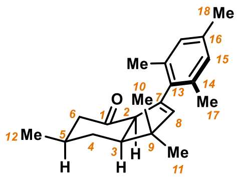

Prepared by following the General Procedure 3a from **3a** (16 µL, 0.10 mmol, 1.0 eq), 2,4,6-trimethylphenylacetylene (47 µL, 0.30 mmol, 3.0 eq), and SmI_2 (150 µL, 0.1 M, 15 mol%). The crude residue was purified by column chromatography R_f = 0.30 (silica gel, petroleum ether:Et_2O = 9:1) to afford the target product **XX** as a yellow oil (24 mg, 0.079 mmol, 79% yield).

^{1}H NMR (400 MHz, $CDCl_3$) δ 6.82 - 6.78 (m, 2H, 2*H-15*), 5.43 (d, *J* = 3.1 Hz, 1H, *H-8*), 3.97 (ddd, *J* = 6.8, 3.1, 1.6 Hz, 1H, *H-2*), 2.42 - 2.31 (m, 1H, *H-3*), 2.31 – 2.15 (m, 11H, *H-5* + *H6a* + *6H-17* + *3H-18*), 1.85 - 1.70 (m, 2H, *H-4a* + *H-6b*), 1.50 - 1.36 (m, 1H, *H-4b*), 1.17 – 1.13 (m, 6H, *3H-10* + *3H-11*), 1.01 (d, *J* = 4.0 Hz, 3H, *3H-12*).

^{13}C{H} NMR (101 MHz, $CDCl_3$) δ 211.7 (*C-1*), 141.3 (*C-8*), 138.2 (*C-7*), 136.4 (*C-13*), 135.8 (*C-16*), 132.4 (*C-14*), 128.5 (*C-15*), 59.6 (*C-2*), 51.6 (*C-3*), 48.8 (*C-6*), 47.3 (*C-9*), 33.9 (*C-4*), 31.2 (*C-5*), 26.7 (*C-11*), 22.72 (*C-10*), 22.66 (*C-12*), 21.1 (*C-17*), 20.8 (*C-18*).

IR (neat, cm^{-1}): 2955, 2924, 2869, 1689, 1454 (fingerprint region excluded).

HRMS (ESI+): calculated for $C_{21}H_{28}ONa$ (M^+Na^+): 296.2140 Found: 296.2149.

3-(3-Methoxy-4-((3-methylbut-2-en-1-yl)oxy)phenyl)-1,1,6-trimethyl-1,3a,5,6,7,7a-hexahydro-4H-inden-4-one (4o)

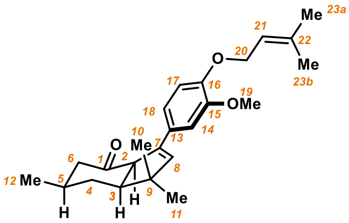

Prepared by following the General Procedure 3a from **3a** (16 µL, 0.10 mmol, 1.0 eq), 4-ethynyl-2-methoxy-1-((3-methylbut-2-en-1-yl)oxy)benzene **SI-4** (65 mg, 0.30 mmol, 3.0 eq), and SmI_2 (150 µL, 0.1 M, 15 mol%). The crude residue was purified by column chromatography R_f = 0.20 (silica gel, petroleum ether:Et_2O = 9:1) to afford the target product **4o** as a pale-yellow oil (28 mg, 0.076 mmol, 76% yield).

^{1}H NMR (500 MHz, $CDCl_3$) δ 6.92 (d, *J* = 2.1 Hz, 1H, *H-14*), 6.83 (dd, *J* = 8.3, 2.1 Hz, 1H, *H-18*), 6.77 (d, *J* = 8.3 Hz, 1H, *H-17*), 5.86 (d, *J* = 2.3 Hz, 1H, *H-8*), 5.49 (dddd, *J* = 6.7, 5.3, 2.9, 1.4 Hz, 1H, *H-21*), 4.55 (d, *J* = 6.7 Hz, 2H, *H-20*), 3.96 (ddd, *J* = 7.8, 2.4, 1.3 Hz, 1H, *H-2*), 3.87 (s, 3H, *3H-19*), 2.50 (ddd, *J* = 11.9, 7.8, 6.5 Hz, 1H, *H-3*), 2.36 – 2.29 (m, 1H, *H-6a*), 1.95 (dtd, *J* = 12.5, 6.2, 3.1 Hz, 1H, *H-5*), 1.83 (dd, *J* = 14.2, 12.5 Hz, 1H, *H-6b*), 1.78 – 1.70 (m, 7H, *H-4a* + *3H-23a* +*3H-23b*), 1.36 – 1.14 (m, 1H, *H-4b*), 1.11 (s, 3H, *3H-10*), 1.06 (s, 3H, *3H-11*), 1.01 (d, *J* = 6.4 Hz, 3H, *3H-12*).

^{13}C{H} NMR (126 MHz, $CDCl_3$) δ 214.3 (*C-1*), 149.2 (*C-15*), 147.9 (*C-16*), 138.1 (*C-7*), 137.9 (*C-8*), 137.6 (*C-13*), 129.0 (*C-13*), 120.16 (*C-21*), 118.7 (*C-17*), 112.8 (*C-14*), 109.6 (*C-18*), 65.9 (*C-20*), 58.7 (*C-2*), 56.0 (*C-19*), 52.4 (*C-3*), 49.0 (*C-6*), 46.9 (*C-9*), 33.2 (*C-4*), 32.2 (*C-5*), 26.7 (*C-12*), 26.0 (*C-23a*), 23.3 (*C-10*), 22.5 (*C-11*), 18.38 (*C-23b*).

IR (neat, cm^{-1}): 2952, 2926, 2866, 1704, 1601, 1510, 1454, 1418 (fingerprint region excluded).

HRMS (ESI+): calculated for $C_{24}H_{32}O_3Na$ (M^+Na^+): 391.2244 Found: 391.2260

1,1,6-Trimethyl-3-phenyloctahydro-4*H*-inden-4-one (4p and 4p')

Prepared by following the General Procedure 3a from **3a** (160 µL, 1.00 mmol, 1.0 eq), styrene (350 µL, 3.0 mmol, 3.0 eq), and SmI_2 (1.5 mL, 0.1 M, 15 mol%). The crude residue was purified by column chromatography to afford two diastereoisomers (d.r. = 2.4:1).

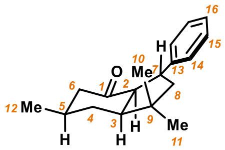

MINOR

R_f = 0.30 (silica gel, petroleum ether:Et_2O = 9:1) gave the minor diastereoisomer **4p'** as a colorless oil (51 mg, 0.20 mmol, 20% yield).

^{1}H NMR (400 MHz, $CDCl_3$) δ 7.33 – 7.12 (m, 5H, 2*H-14* + 2*H-15* + *H-16*), 3.70 (dt, *J* = 10.8, 8.5 Hz, 1H, *H-7*), 2.88 (dd, *J* = 10.5, 8.6 Hz, 1H, *H-2*), 2.46 – 2.25 (m, 2H, *H-6a* + *H-3*), 2.09 – 1.89 (m, 3H, *H-5* + *H-6b* + *H-8a*), 1.81 – 1.65 (m, 2H, *H-4a* + *H-8b*), 1.10 (s, 3H, *3H-10*), 1.04 (m, 4H, *H-4b* + *3H-12*), 0.87 (s, 3H, *3H-11*).

^{13}C{H} NMR (101 MHz, $CDCl_3$) δ 214.0 (*C-1*), 145.2 (*C-13*), 128.6 (*C-14*), 127.5 (*C-15*), 126.2 (*C-16*), 59.5 (*C-2*), 52.1 (*C-3*), 49.3 (*C-8*), 47.5 (*C-6*), 43.4 (*C-7*), 41.7 (*C-9*), 32.4 (*C-4*), 30.6 (*C-5*), 29.2 (*C-11*), 23.4 (*C-12*), 22.3 (*C-10*).

IR (neat, cm^{-1}): 2950, 2927, 2867, 1703, 1494, 1455 (fingerprint region excluded).

HRMS (ESI+): calculated for $C_{18}H_{24}ONa$ (M^+Na^+): 279.1706 Found: 279.1702.

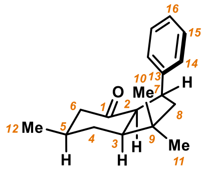

MAJOR

R_f = 0.25 (silica gel, petroleum ether:Et_2O = 9:1) gave the major diastereoisomer **4p** as colorless crystals (124 mg, 0.48 mmol, 48% yield).

^{1}H NMR (400 MHz, $CDCl_3$) δ 7.31 – 7.11 (m, 5H, *2H-14* + *2H-15* + *H-16*), 3.88 (ddd, *J* = 12.9, 10.6, 8.3 Hz, 1H, *H-7*), 3.37 (ddt, *J* = 12.9, 7.1, 1.6 Hz, 1H, *H-2*), 2.22 (dd, *J* = 13.3, 10.5 Hz, 1H, *H-8a*), 2.01 –

1.85 (m, 2H, *H-3* + *H-6a*), 1.85 – 1.74 (m, 2H, *H-4a* + *H-8b*), 1.69 – 1.58 (m, *H-5*), 1.18 – 1.04 (m, 7H, *H-4b* + *3H-10* + *3H-11*), 0.98 (dd, *J* = 15.4, 13.2 Hz, 1H, *H-6b*), 0.93 (d, *J* = 6.5 Hz, 3H, *3H-12*).

^{13}C{H} NMR (101 MHz, $CDCl_3$) δ 214.1 (*C-1*), 140.7 (*C-13*), 129.0 (*C-14*), 128.3 (*C-15*), 126.7 (*C-16*), 56.0 (*C-2*), 51.9 (*C-3*), 50.2 (*C-6*), 46.3 (*C-7*), 42.4 (*C-8*), 42.3 (*C-9*) 34.4 (*C-4*), 31.5 (*C-5*), 28.4 (*C-10*), 24.6 (*C-11*), 22.6 (*C-12*).

Melting point (from petroleum ether: Et_2O): 85 – 87 °C.

IR (neat, cm^{-1}): 2951, 2869, 1698, 1495, 1457, 1416 (fingerprint region excluded).

HRMS (ESI+): calculated for $C_{18}H_{24}ONa$ (M^+Na^+): 279.1706 Found: 279.1717.

1,1,6-Trimethyl-3-(4-(trifluoromethyl)phenyl)octahydro-4*H*-inden-4-one (4q and 4q')

Prepared by following the General Procedure 3a from **3a** (16 µL, 0.10 mmol, 1.0 eq), 4-(trifluoromethyl)styrene (44 µL, 0.30 mmol, 3.0 eq), and SmI_2 (150 µL, 0.1 M, 15 mol%). The crude residue was purified by column chromatography to afford two diastereoisomers (d.r. = 2.3:1).

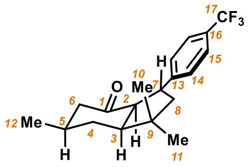

MINOR

R_f = 0.45 (silica gel, petroleum ether:Et_2O = 9:1) gave the minor diastereoisomer **4q'** as a colorless oil (6.7 mg, 0.021 mmol, 21% yield).

^{1}H NMR (500 MHz, $CDCl_3$) δ 7.52 (d, *J* = 8.1 Hz, 2H, *2H-15*), 7.36 (d, *J* = 8.1 Hz, 2H, *2H-14*), 3.75 (dt, *J* = 11.2, 8.4 Hz, 1H, *H-7*), 2.85 (dd, *J* = 10.9, 8.5 Hz, 1H, *H-2*), 2.48 – 2.43 (m, 1H, *H-6a*), 2.41 – 2.35 (m, 1H, *H-3*), 2.10 – 1.98 (m, 2H, *H-5* + *H-8a*), 1.95 – 1.89 (m, 1H, *H-6b*), 1.77 (ddt, *J* = 13.1, 6.8, 2.4 Hz, 1H, *H-4a*), 1.70 (dd, *J* = 12.6, 11.1 Hz, 1H, *H-8b*), 1.11 (s, 3H, *3H-10*), 1.05 (d, *J* = 6.5 Hz, 3H, *3H-12*), 1.02 – 0.93 (m, 1H, *H-4b*), 0.86 (s, 3H, *3H-11*).

^{13}C{H} NMR (126 MHz, $CDCl_3$) δ 213.4 (*C-1*), 149.1 (*C-13*), 128.2 (q, J = 32.3 Hz, *C-16*), 127.6 (*C-14*), 125.2 (q, J = 21.4 Hz, *C-15*), 58.9 (*C-2*), 51.5 (*C-3*), 48.8 (*C-8*), 47.2 (*C-6*), 42.7 (*C-7*), 41.4 (*C-9*), 32.0 (*C-4*), 30.0 (*C-5*), 28.6 (*C-11*), 22.9 (*C-12*), 21.9 (*C-10*). [C-17 signal not visible]

^{19}F NMR (471 MHz, $CDCl_3$) δ -62.35.

IR (neat, cm^{-1}): 2957, 2873, 1703, 1618, 1323 (fingerprint region excluded).

HRMS (APCI+): calculated for $C_{19}H_{24}F_3O$ (M^+H^+): 325.1774 Found: 325.1782.

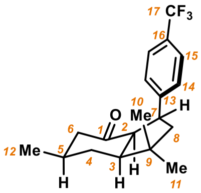

MAJOR

R_f = 0.30 (silica gel, petroleum ether:Et_2O = 9:1) gave the major diastereoisomer **4q** as a white solid (16.0 mg, 0.50 mmol, 50% yield).

^{1}H NMR (500 MHz, $CDCl_3$) δ 7.51 (d, J = 8.1 Hz, 2H, *2H-14*), 7.32 (d, *J* = 8.1 Hz, 2H, *2H-15*), 3.90 (ddd, *J* = 12.9, 10.8, 8.1 Hz, 1H, *H-7*), 3.45 – 3.37 (m, 1H, *H-2*), 2.19 (dd, *J* = 13.2, 10.9 Hz, 1H, *H-8a*), 2.08 – 1.92 (m, 2H, *H-3* + *H-6a*), 1.87 – 1.77 (m, 2H, *H-4a* + *H-8b*), 1.72 – 1.60 (m, 1H, *H-5*), 1.17 (s, 3H, *3H-10*), 1.11 (s, 3H, *3H-11*), 1.10 – 0.97 (m, 2H, *H-4b* + *H-6b*), 0.95 (d, *J* = 6.4 Hz, 3H, *3H-12*).

^{13}C{H} NMR (126 MHz, $CDCl_3$) δ 213.2 (*C-1*), 144.9 (*C-13*), 129.1 (*C-14*), 128.7 (q, *J* = 32.3 Hz, *C-16*), 125.0 (q, *J* = 21.4 Hz, *C-15*), 124.1 (q, *J* = 272.1 Hz, *C-17*), 55.6 (*C-2*), 51.5 (*C-3*), 50.3 (*C-6*), 45.9 (*C-7*), 42.4 (*C-9*), 42.3 (*C-8*), 34.3 (*C-4*), 31.1 (*C-5*), 28.1 (*C-10*), 24.4 (*C-11*), 22.4 (*C-12*).

^{19}F NMR (471 MHz, $CDCl_3$) δ -62.44.

Melting Point (from petroleum ether: Et_2O): 87 – 92 °C**.**

IR (neat, cm^{-1}): 2957, 2873, 1703, 1618, 1323 (fingerprint region excluded).

HRMS (APCI+): calculated for $C_{19}H_{23}F_3ONa$ (M^+Na^+): 347.1598 Found: 347.1606.

3-([1,1'-Biphenyl]-4-yl)-1,1,6-trimethyloctahydro-4*H*-inden-4-one (4r and 4r')

Prepared by following the General Procedure 3a from **3a** (16 µL, 0.10 mmol, 1.0 eq), 4-vinylbiphenyl (54 mg, 0.30 mmol, 3.0 eq), and SmI_2 (150 µL, 0.1 M, 15 mol%). The crude residue was purified by column chromatography to afford two diastereoisomers (d.r. = 1.9:1).

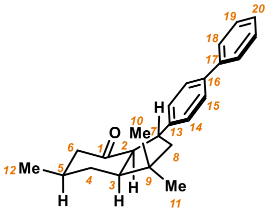

MINOR

R_f = 0.30 (silica gel, petroleum ether:Et_2O = 9:1) gave the major diastereoisomer **4r'** as a colorless oil (16.0 mg, 0.25 mmol, 25% yield).

^{1}H NMR (400 MHz, $CDCl_3$) δ 7.60 – 7.28 (m, 9H, *2H-14* + *2H*-15 + *2H-18* + *2H-19* + *H-20*), 3.74 (dt, *J* = 10.8, 8.5 Hz, 1H, *H-7*), 2.92 (dd, J = 10.5, 8.6 Hz, 1H, *H-2*), 2.49 – 2.32 (m, 2H, *H-3* + *H-6a*), 2.12 – 1.91 (m, 3H, *H-5* + *H-6b* + *H-8a*), 1.83 – 1.70 (m, 2H, *H-4a* + *H-8b*), 1.12 (s, 3H, *3H-10*), 1.10 – 0.96 (m, 4H, *H-4b* + *3H-12*), 0.89 (s, 3H, *3H-11*).

^{13}C{H} NMR (101 MHz, $CDCl_3$) δ 213.8 (*C-1*), 144.1 (*C-17*), 141.0 (*C-13*), 139.0 (*C-16*), 128.7 (*C-14*), 127.7 (*C-15*), 127.2 (*C-18*), 127.0 (*C-19*), 127.0 (*C-20*), 59.3 (*C-2*), 51.9 (*C-3*), 49.1 (*C-8*), 47.4 (*C-6*), 43.0 (*C-7*), 41.5 (*C-9*), 32.3 (*C-4*), 30.5 (*C-5*), 29.0 (*C-11),* 23.2 (*C-12*), 22.1 (*C-10*).

IR (neat, cm^{-1}): 2957, 2849, 1680, 1536, 1493, 1459 (fingerprint region excluded).

HRMS (ESI+): calculated for $C_{24}H_{29}O$ (M^+H^+): 333.2213 Found: 333.2205.

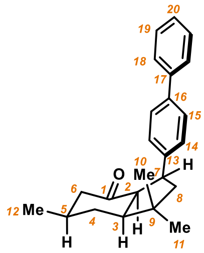

MAJOR

R_f = 0.25 (silica gel, petroleum ether:Et_2O = 9:1) gave the major diastereoisomer **4r** as colorless needles (15.0 mg, 0.45 mmol, 45% yield).

^{1}H NMR (400 MHz, $CDCl_3$) δ 7.61 – 7.26 (m, 9H, *2H-14* + *2H*-15 + *2H-18* + *2H-19* + *H-20*), 3.92 (ddd, *J* = 12.9, 10.6, 8.3 Hz, 1H, *H-7*), 3.41 (dd, *J* = 12.9, 7.1 Hz, 1H, *H-2*), 2.25 (dd, J = 13.2, 10.5 Hz, 1H, *H-8a*), 2.07 – 1.88 (m, 2H, *H-3* + *H-6a*), 1.85 – 1.79 (m, 2H, *H-4a*+ *H-8b*), 1.65 (dddt, *J* = 15.9, 9.7, 6.5, 3.3 Hz, 1H, *H-5*), 1.19 – 1.01 (m, 8H, *H-4b* + *H-6b, 3H-10* + *3H-11*), 0.94 (d, J = 6.5 Hz, 3H, *3H-12*).

^{13}C{H} NMR (101 MHz, $CDCl_3$) δ 214.0 (*C-1*), 140.6 (*C-17*), 139.7 (*C-13*), 139.2 (*C-16*), 129.2 (*C-14*), 128.7 (*C-15*), 127.1 (*C-18*), 126.9 (*C-19*), 126.8 (*C-20*), 55.9 (*C-2*), 51.8 (*C-3*), 50.2 (*C-6*), 45.8 (*C-7*), 42.4 (*C-8*), 42.3 (*C-9*), 34.2 (*C-4*), 31.3 (*C-5*), 28.2 (*C-11*), 24.5 (*C-10*), 22.5 (*C-12*).

Melting Point (from petroleum ether: Et_2O): 87 – 89 °C.

IR (neat, cm^{-1}): 2951, 2927, 2868, 1699, 1486, 1454 (fingerprint region excluded).

HRMS (APCI+): calculated for $C_{24}H_{28}ONa$ (M^+Na^+): 355.2038 Found: 355.2027**.**

3-(3-Fluorophenyl)-1,1,6-trimethyloctahydro-4*H*-inden-4-one (4s and 4s')

Prepared by following the General Procedure 3a from **3a** (16 µL, 0.10 mmol, 1.0 eq), 3-fluorostyrene (37 µL, 0.30 mmol, 3.0 eq), and SmI_2 (150 µL, 0.1 M, 15 mol%). The crude residue was purified by column chromatography to afford two diastereoisomers (d.r. = 2.3:1).

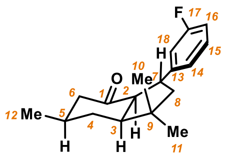

MINOR

R_f = 0.45 (silica gel, petroleum ether:Et_2O = 9:1) gave the minor diastereoisomer **4s'** as a colorless oil (6.7 mg, 0.025 mmol, 25% yield).

^{1}H NMR (500 MHz, $CDCl_3$) δ 7.22 (td, *J* = 7.9, 6.1 Hz, 1H, *H-15*), 7.02 (dt, *J* = 7.7, 1.2 Hz, 1H, *H-14*), 6.95 (dt, *J* = 10.4, 2.2 Hz, 1H, *H-18*), 6.85 (tdd, *J* = 8.3, 2.6, 1.0 Hz, 1H, *H-16*),

3.69 (dt, J = 11.1, 8.4 Hz, 1H, *H-7*), 2.84 (dd, J = 10.8, 8.5 Hz, 1H, *H-2*), 2.48 – 2.31 (m, 2H, *H-3* + *H-6a*), 2.11 – 1.88 (m, 3H, *H-5* + *H-6b* + *H-8a*), 1.81 – 1.72 (m, 1H, *H-4a*), 1.67 (dd, J = 12.6, 11.0 Hz, 1H, *H-6b*), 1.09 (s, 3H, *3H-10*), 1.04 (d, J = 6.5 Hz, 3H, *3H-12*), 1.07 – 0.93 (m, 1H, *H-4b*), 0.84 (s, 3H, *3H-11*).

^{13}C{H} NMR (126 MHz, $CDCl_3$) δ 213.7 (*C-1*), 163.0 (d, J = 245.0 Hz, *C-17*), 147.8 (d, J = 6.9 Hz, *C-13*), 129.8 (d, J = 8.3 Hz, *C-15*), 123.1 (d, J = 2.7 Hz *C-14*), 114.2 (d, J = 21.2 Hz, *C-18*), 112.9 (d, J = 21.0 Hz, *C-16*), 59.1 (*C-2*), 51.7 (*C-3*), 49.0 (*C-8*), 47.4 (*C-6*), 42.8 (*C-7*), 41.5 (*C-9*), 32.2 (*C-4*), 30.2 (*C-5*), 28.8 (*C-10*), 23.1 (*C-11*), 22.1 (*C-12*).

^{19}F NMR (471 MHz, $CDCl_3$) δ -113.50 (ddd, J = 10.5, 8.6, 6.1 Hz).

IR (neat, cm^{-1}): 2957, 2931, 2866, 1704, 1494, 1465, 1446 fingerprint region excluded).

HRMS (APCI+): calculated for $C_{18}H_{23}FONa$ (M^+Na^+): 297.1625 Found: 297.1623.

MAJOR

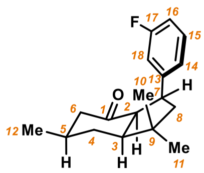

R_f = 0.35 (silica gel, petroleum ether:Et_2O = 9:1) gave the major diastereoisomer **4s** as a white amorphous waxy solid (13.8 mg, 0.050 mmol, 50% yield).

^{1}H NMR (400 MHz, $CDCl_3$) δ 7.26 – 7.16 (m, 1H, *H-15*), 7.03 – 6.91 (m, 2H, *H-14* + *H-18*), 6.88 – 6.73 (m, 1H, *H-16*), 3.85 (ddd, J = 12.8, 10.6, 8.2 Hz, 1H, *H-7*), 3.37 (ddt, J = 13.0, 7.1, 1.6 Hz, 1H, *H-2*), 2.13 (dd, J = 13.3, 10.6 Hz, 1H, *H-6a*), 2.02 (ddt, J = 15.5, 3.9, 2.0 Hz, 1H, *H-8a*), 1.92 (ddd, J = 12.8, 7.1, 5.2 Hz, 1H, *H-3*), 1.85 – 1.75 (m, 2H, *H-4a* + *H-6b*), 1.72 – 1.59 (m, 1H, *H-5*), 1.16 (s, 3H, *3H-10*), 1.10 (s, 3H, *3H-11*), 1.07 – 0.99 (m, 2H, *H-4b* + *H-8b*), 0.94 (d, J = 6.5 Hz, 3H, *3H-12*).

^{13}C{H} NMR (126 MHz, $CDCl_3$) δ 213.6 (*C-1*), 162.8 (d, J = 245.5 Hz, *C-17*), 143.6 (d, J = 6.8 Hz, *C-13*), 129.7 (d, J = 8.3 Hz, *C-15*), 125.0 (d, J = 2.6 Hz, *C-14*), 115.6 (d, J = 21.4 Hz, *C-18*), 113.6 (d, J = 21.1 Hz, *C-16*), 55.8 (*C-2*), 51.8 (*C-3*), 50.3 (*C-6*), 46.0 (*C-7*), 42.6 (*C-9*), 42.4 (*C-8*), 34.5 (*C-4*), 31.4 (*C-5*), 28.3 (*C-10*), 24.6 (*C-11*), 22.6 (*C-12*).

^{19}F NMR (471 MHz, $CDCl_3$) δ -113.19 – -113.31 (m).

Melting Point (from petroleum ether: Et_2O): 86 – 90 °C.

IR (neat, cm^{-1}): 2953, 2931, 2871, 1699, 1489, 1458 (fingerprint region excluded).

HRMS (APCI+): calculated for $C_{18}H_{23}FONa$ (M^+Na^+): 297.1625 Found: 297.1615.

3-(2,6-Dichlorophenyl)-1,1,6-trimethyloctahydro-4*H*-inden-4-one (4t and 4t')

Prepared by following the General Procedure 3a from **3a** (16 µL, 0.10 mmol, 1.0 eq), 2,6-dichlorostyrene (41 µL, 0.30 mmol, 3.0 eq), and SmI_2 (150 µL, 0.1 M, 15 mol%). The crude residue was purified by column chromatography to afford two diastereoisomers (d.r. = 1.5:1).

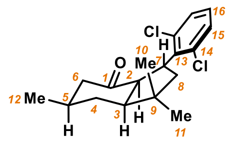

MINOR

R_f = 0.35 (silica gel, petroleum ether:Et_2O = 9:1) obtained the minor diastereoisomer **4t'** as a colorless oil (11 mg, 0.033 mmol, 33% yield).

^{1}H NMR (500 MHz, $CDCl_3$) δ 7.30 – 7.22 (m, 2H, *2H-15*), 7.02 (t, J = 8.0 Hz, 1H, *H-16*), 4.66 (dt, J = 12.3, 8.0 Hz, 1H, *H-7*), 3.54 – 3.45 (m, 1H, *H-2*), 2.73 (td, J = 11.9, 7.5 Hz, 1H, *H-8a*), 2.44 (ddd, J = 18.1, 5.7, 2.4 Hz, 1H, *H-6a*), 2.20 – 2.07 (m, 2H, *H-6b* + *H-3*), 1.88 – 1.74 (m, 2H, *H-4a* + *H-5*), 1.47 – 1.36 (m, 2H, *H-4b* + *H-8b*), 1.14 (s, 3H, *3H-10*), 1.13 (s, 3H, *3H-11*), 1.02 (d, *J* = 6.4 Hz, 3H, *3H-12*).

^{13}C{H} NMR (126 MHz, $CDCl_3$) δ 212.9 (*C-1*), 135.8 (*C-13* + *C-14 two overlapping signals*), 128.0 (*C-15* + *C-16 two overlapping signals*), 52.0 (*C-2*), 50.3 (*C-3*), 47.2 (*C-6*), 43.5 (*C-7*), 42.2 (*C-9*), 39.8 (*C-8*), 34.4 (*C-4*), 30.0 (*C-5*), 28.9 (*C-10*), 24.7 (*C-11*), 22.6 (*C-12*).

IR (neat, cm^{-1}): 2957, 2872, 1721, 1573, 1482, 1225 (fingerprint region excluded).

HRMS (ESI+): calculated for $C_{18}H_{23}Cl_2O$ (M^+H^+): 325.1120 Found: 325.1122.

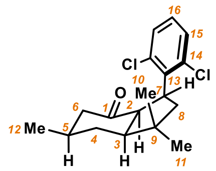

MAJOR

R_f = 0.30 (silica gel, petroleum ether:Et_2O = 9:1) obtained the major diastereoisomer **4t** as a colorless oil (15 mg, 0.047 mmol, 47% yield).

^{1}H NMR (500 MHz, $CDCl_3$) δ 7.29 – 7.22 (m, 2H, *2H-15*), 7.02 (t, *J* = 8.0 Hz, 1H, *H-16*), 4.66 (dt, *J* = 12.4, 8.0 Hz, 1H, *H-2*), 3.50 (dd, *J* = 11.8, 7.8 Hz, 1H, *H-7*), 2.73 (td, *J* = 11.9, 7.5 Hz, 1H, *H-3*), 2.44 (ddd, *J* = 18.1, 5.7, 2.4 Hz, 1H, *H-6a*), 2.20 – 2.07 (m, 2H, *H-5* + *H-8a*), 1.89 – 1.74 (m, 2H, *H-4a* + *H-6b*), 1.68 (dd, *J* = 12.1, 8.1 Hz, 1H, *H-8b*), 1.10 (s, 3H, *3H-10*), 1.03 (d, *J* = 6.5 Hz, 3H, *3H-12*), 1.02 – 0.92 (m, 1H, *H-4b*), 0.80 (s, 3H, *3H-11*).

^{13}C{H} NMR (126 MHz, $CDCl_3$) δ 214.0 (*C-1*), 138.4 (*C-13*), 129.5 (*C-14* + *C-15 two overlapping signals*), 127.7 (*C-16*), 55.0 (*C-2*), 50.4 (*C-3*), 47.8 (*C-6*), 45.3 (*C-8*), 42.2 (*C-9*), 38.4 (*C-7*), 32.0 (*C-4*), 29.5 (*C-5*), 28.3 (*C-10*), 23.2 (*C-11*), 22.0 (*C-12*).

IR (neat, cm^{-1}): 2953, 2869, 1710, 1560, 1434, 1388, 1212, 1086 (fingerprint region excluded).

HRMS (APCI+): calculated for $C_{18}H_{22}Cl_2ONa$ (M^+Na^+): 347.0945 Found: 347.0951.

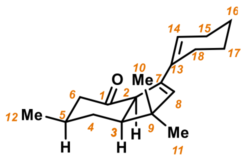

3-(Cyclohex-1-en-1-yl)-1,1,6-trimethyl-1,3a,5,6,7,7a-hexahydro-4*H*-inden-4-one (4u)

Prepared by following the General Procedure 3b from **3a** (32 µL, 0.20 mmol, 1.0 eq), 1-ethynylcyclohex-2-ene (70 µL, 0.60 mmol, 3.0 eq), Sm metal (5.0 mg, 15 mol%), and SmI_2 (150 µL, 0.1 M, 15 mol%). The crude residue was purified by column chromatography R_f = 0.20 (silica gel, petroleum ether:Et_2O = 19:1) to afford the target product **4u** as a pale-yellow oil (34.9 mg, 0.135 mmol, 68% yield).

***Note: 4u** appears to be light/temperature sensitive (turns bright yellow) under ambient conditions after 1 h.*

^{1}H NMR (500 MHz, $CDCl_3$) δ 5.58 – 5.52 (m, 2H, *H-8* + *H-14*), 3.71 – 3.65 (m, 1H, *H-2*), 2.50 – 2.36 (m, 2H, *H-3* + *H-6a*), 2.24 – 1.95 (m, 5H, *H-5* + *2CH₂*), 1.88 (dd, *J* = 15.0, 11.9 Hz, 1H, *H-6b*), 1.76 – 1.44 (m, 5H, *H-4a* + *2CH₂*), 1.21 – 1.13 (m, 1H, *H-4b*), 1.07 – 0.99 (m, 6H, *3H-10* + *3H-12*), 0.90 (d, *J* = 4.8 Hz, 3H, *3H-11*).

^{13}C{H} NMR (126 MHz, $CDCl_3$) δ 214.8 (*C-1*), 139.3 (*C-7*), 136.8 (*C-8*), 132.7 (*C-13*), 125.6 (*C-14*), 57.7 (*C-2*), 51.7 (*C-3*), 48.8 (*C-6*), 46.2 (*C-9*), 32.5 (*C-4*), 31.6 (*C-5*), 27.3 (*C-10*), 26.2 (*CH_2*), 25.6 (*CH_2*), 23.6 (*C-10*), 22.7 (*CH_2*), 22.2 (*CH_2 + C-12, two overlapping signals*).

IR (neat, cm^{-1}): 2950, 2925, 2865, 1707, 1455 (fingerprint region excluded).

HRMS (APCI+): calculated for $C_{18}H_{27}O$ (M^+H^+): 259.2056 Found: 259.2062.

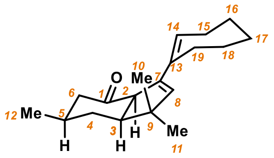

3-(Cyclohept-1-en-1-yl)-1,1,6-trimethyl-1,3a,5,6,7,7a-hexahydro-4*H*-inden-4-one (4v)

Prepared by following the General Procedure 3b from **3a** (32 µL, 0.20 mmol, 1.0 eq), 1-ethynylcyclohept-2-ene (72 mg, 0.60 mmol, 3.0 eq), Sm metal (5.0 mg, 15 mol%), and SmI_2 (150 µL, 0.1 M, 15 mol%). The crude residue was purified by column chromatography R_f = 0.20 (silica gel, petroleum ether:Et_2O = 19:1) to afford the target product **4v** as a pale-yellow oil (32.7 mg, 0.120 mmol, 60% yield).

Note:* 4v *appears to be light/temperature sensitive (turns bright yellow) under ambient conditions after 30 min and decomposed during acquisition of the 2D-NMR spectra.

^{1}H NMR (500 MHz, $CDCl_3$) δ 5.71 (t, *J* = 6.8 Hz, 1H, *H-14*), 5.60 – 5.50 (m, 1H, *H-8*), 3.70 (d, *J* = 8.1 Hz, 1H, *H-2*), 2.45 – 2.39 (m, 1H, *H-3*), 2.37 – 2.33 (m, 3H, *H-6a* + *CH₂*), 2.18 – 2.08 (m, 2H, *CH₂*), 1.93 (ddd, *J* = 9.7, 3.6, 1.8 Hz, 1H, *H-5*), 1.87 (dd, *J* = 13.8, 12.1 Hz, 1H, *H-6b*), 1.80 – 1.63 (m, 3H, *H-4a* + *CH₂*), 1.58 – 1.32 (m, 4H, *CH₂* + *CH₂*), 1.19 (dt, *J* = 13.4, 12.0 Hz, 1H, *H-4b*), 1.04 (s, 3H, *3H-10*), 1.01 (d, *J* = 6.3 Hz, 3H, *3H-12*), 0.95 (s, 3H, *3H-11*).

^{13}C{H} NMR (126 MHz, $CDCl_3$) δ 214.8 (*C-1*), 140.6 (*C-7* + *C-13 two overlapping signals*), 137.1 (*C-8*), 130.0 (*C-14*), 58.4 (*C-2*), 52.4 (*C-3*), 48.9 (*C-6*), 46.5 (*C-9*), 33.1 (*C-4*), 32.6 (*C-5*), 32.3 (*CH₂*), 30.6 (*CH₂*), 28.6 (*CH₂*), 27.0 (*CH₂*), 26.9 (*CH₂*), 26.7 (*C-10*), 23.4 (*C-12*), 22.5 (*C-11*).

IR (neat, cm^{-1}): 2952, 2921, 2850, 1707, 1454 (fingerprint region excluded).

HRMS (APCI+): calculated for $C_{19}H_{29}O$ (M^+H^+): 273.2213 Found: 273.2218.

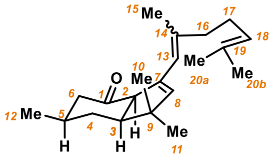

3-(2,6-Dimethylhepta-1,5-dien-1-yl)-1,1,6-trimethyl-1,3a,5,6,7,7a-hexahydro-4*H*-inden-4-one (4w)

Prepared by following the General Procedure 3b from **3a** (32 µL, 0.20 mmol, 1.0 eq), 4,8-dimethylnona-3,7-dien-1-yne (108 µL, 0.60 mmol, 3.0 eq), Sm metal (5.0 mg, 15 mol%), and SmI_2 (150 µL, 0.1 M, 15 mol%). The crude residue was purified by column chromatography R_f = 0.20 (silica gel, petroleum ether:Et_2O = 19:1) to afford the target product **4w** as a pale-yellow oil and as a mixture of double bond isomers (31.0 mg, 0.103 mmol, 52% yield, d.r. = 1.3:1).

Note:* 4w *appears to be light/temperature sensitive (turns bright yellow) under ambient conditions after 30 min and decomposed during acquisition of the 2D-NMR spectra.

^{1}H NMR (400 MHz, $CDCl_3$) δ 5.45 – 5.38 (m, 2H, *H-8* + *H-13*), 5.14 – 4.98 (m, 1H, *H-18*), 3.59 – 3.48 (m, 1H, *H-2*), 2.36 – 2.15 (m, 3H, *H-3* + *H-16a* + *H-6a*), 2.12 – 1.98 (m, 3H, H-*5* + *H-16b* + *CH₂*), 1.85 – 1.53 (m, 10H, *H-4a* + *3H-12* + *3H-20a* + *3H-20b*), 1.13 – 0.93 (m, 10H, *H-4b* + *3H-10* + *3H-11* + *3H-12*).

^{13}C{H} NMR (101 MHz, $CDCl_3$) δ 213.62 (*C-1*), 213.59 (*C-1*), 141.5 (*C-19*), 141.4 (*C-19*), 139.0 (*C-8*), 138.3 (*C-8*), 135.1 (*C-7*), 134.8 (*C-7*), 131.9 (*C-14*), 131.8 (*C-14*), 124.2 (*C-18*), 124.0 (*C-18*), 119.1 (*C-13*), 118.3 (*C-13*), 60.34 (*C-2*), 60.32 (*C-2*), 51.1 (*C-3*), 48.9 (*C-6*), 48.8 (*C-6*), 47.3 (*C-9*), 40.9 (*C-9*), 33.54 (*C-16*), 33.49 (*C-4*), 33.47 (*C-4*), 31.5 (*C-5*), 31.4 (*C-5*), 26.83 (*C-17*), 26.79 (*C-17*), 26.64

(CH_3), 26.56 (CH_3), 25.9 (CH_3), 25.8 (CH_3), 24.3 (CH_3), 23.1 (CH_3), 22.9 (CH_3), 22.5 (CH_3), 18.7 (CH_3), 17.9 (CH_3), 17.8 (CH_3).

IR (neat, cm^{-1}): 2952, 2925, 2870, 1706, 1454 (fingerprint region excluded).

HRMS (APCI+): calculated for $C_{21}H_{33}O$ (M^+H^+): 301.2526 Found: 301.2531.

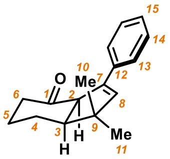

1,1-Dimethyl-3-phenyl-1,3a,5,6,7,7a-hexahydro-4*H*-inden-4-one (4x)

Prepared by following the General Procedure 3a from **3x** (15 µL, 0.10 mmol, 1.0 eq), phenylacetylene (33 µL, 0.30 mmol, 3.0 eq), and SmI_2 (150 µL, 0.1 M, 15 mol%). The crude residue was purified by column chromatography R_f = 0.20 (silica gel, petroleum ether:Et_2O = 9:1) to afford the target product **4x** as a white amorphous solid (17.0 mg, 0.070 mmol, 70% yield).

^{1}H NMR (400 MHz, $CDCl_3$) δ 7.36 – 7.31 (m, 2H, *2H-13*), 7.29 – 7.22 (m, 2H, *2H-14*), 7.20 – 7.15 (m, 1H, *H-15*), 6.06 (d, *J* = 1.8 Hz, 1H, *H-8*), 3.94 (dt, *J* = 8.8, 1.5 Hz, 1H, *H-2*), 2.60 (td, *J* = 8.6, 6.9 Hz, 1H, *H-3*), 2.50 – 2.36 (m, 1H, *H-6a*), 2.22 (ddd, *J* = 14.7, 9.4, 5.4 Hz, 1H, *H-6b*), 2.08 – 1.94 (m, 1H, *H-4a*), 1.93 – 1.78 (m, 2H, *H-4b* + *H-5a*), 1.72 – 1.58 (m, 1H, *H-5b*), 1.11 (s, 3H, *3H-10*), 1.00 (s, 3H, *3H-11*).

^{13}C{H} NMR (101 MHz, $CDCl_3$) δ 213.9 (*C-1*), 139.6 (*C-8*), 138.3 (*C-7*), 136.0 (*C-12*), 128.4 (*C-14*), 127.3 (*C-15*), 126.2 (*C-13*), 58.9 (*C-2*), 52.8 (*C-3*), 47.2 (*C-9*), 41.1 (*C-6*), 27.5 (*C-10*), 24.8 (*C-4*), 23.7(*C-11*), 23.6 (*C-5*).

Melting Point (from petroleum ether: Et_2O): 58 – 60 °C.

IR (neat, cm^{-1}): 2951, 2866, 1706, 1494, 1445 (fingerprint region excluded).

HRMS (APCI+): calculated for $C_{17}H_{20}ONa$ (M^+H^+): 263.1406 Found: 263.1401.

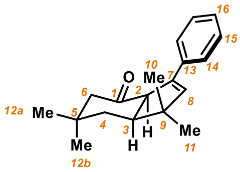

1,1,6,6-Tetramethyl-3-phenyl-1,3a,5,6,7,7a-hexahydro-4*H*-inden-4-one (4y)

Prepared by following the General Procedure 3a from **3y** (16 µL, 0.10 mmol, 1.0 eq), phenylacetylene (33 µL, 0.30 mmol, 3.0 eq), and SmI_2 (150 µL, 0.1 M, 15 mol%). The crude residue was purified by column chromatography R_f = 0.40 (silica gel, petroleum

ether:EtOAc = 19:1) to afford the target product **4y** as a white amorphous solid (23.6 mg, 0.088 mmol, 88% yield).

^{1}H NMR (400 MHz, $CDCl_3$) δ 7.37 – 7.31 (m, 2H, *H-14*), 7.29 – 7.24 (m, 2H, *H-15*), 7.22 – 7.15 (m, 1H, *H-16*), 5.85 (dd, *J* = 2.9, 0.8 Hz, 1H, *H-8*), 4.03 (ddd, *J* = 6.1, 2.8, 1.6 Hz, 1H, *H-2*), 2.52 – 2.41 (m, 1H, *H-3*), 2.01 (d, *J* = 13.0 Hz, 1H, *H-6a*), 1.91 (ddd, *J* = 13.0, 2.6, 1.7 Hz, 1H, *H-6b*), 1.70 – 1.55 (m, 1H, *H-4a*), 1.43 (ddt, *J* = 11.3, 5.7, 2.5 Hz, 1H, *H-4b*), 1.14 (s, 3H, *3H-10*), 1.10 (s, 3H, *3H-11*), 1.00 (s, 3H, *3H-12a*), 0.88 (s, 3H, *3H-12b*).

^{13}C{H} NMR (101 MHz, $CDCl_3$) δ 213.9 (*C-1*), 139.7 (*C-8*), 139.2 (*C-7*), 136.1 (*C-13*), 128.4 (*C-14*), 127.4 (*C-16*), 126.4 (*C-15*), 58.8 (*C-2*), 53.6 (*C-6*), 50.7 (*C-3*), 47.0 (*C-9*), 38.2 (*C-4*), 35.8 (*C-5*), 32.3 (*C-12a*), 26.0 (*C-10*), 24.9 (*C-12b*), 22.5 (*C-11*).

Melting Point (from petroleum ether: Et_2O): 59 – 62 °C.

IR (neat, cm^{-1}): 2954, 2926, 2868, 1703, 1465 (fingerprint region excluded).

HRMS (ESI+): calculated for $C_{19}H_{24}ONa$ (M^+Na^+): 291.1719 Found: 291.1713.

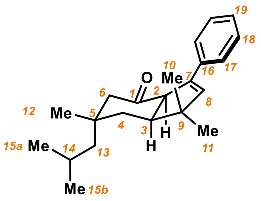

6-*iso*-Butyl-1,1,6-trimethyl-3-phenyl-1,3a,5,6,7,7a-hexahydro-4*H*-inden-4-one (4z)

Prepared by following the General Procedure 3a from **3z** (41.6 mg, 0.20 mmol, 1.0 eq), phenylacetylene (66 µL, 0.60 mmol, 3.0 eq), Sm metal (5 mg, 0.030, 15 mol%) and SmI_2 (300 µL, 0.1 M, 15 mol%). The crude residue was purified by column chromatography R_f = 0.25 (silica gel, petroleum ether:Et_2O = 19:1) to afford the target product **4z** as a white colorless oil (37.3 mg, 0.12 mmol, 60% yield).

^{1}H NMR (400 MHz, $CDCl_3$) δ 7.38 – 7.30 (m, 2H, *2H-Ar*), 7.29 – 7.23 (m, 2H, *2H-Ar*), 7.23 – 7.12 (m, 1H, *H-19*), 5.84 (d, *J* = 2.9 Hz, 1H, *H-8*), 4.05 – 3.98 (m, 1H, *H-2*), 2.45 (dt, *J* = 12.1, 6.3 Hz, 1H, *H-3*), 2.04 (dt, *J* = 13.1, 2.0 Hz, 1H, *H-6a*), 1.91 (d, *J* = 13.1 Hz, 1H, *H-6b*), 1.74 – 1.59 (m, 1H, *H-14*), 1.59 (m, 2H, *H-4*), 1.17 – 1.10 (m, 5H, *2H-13* + *3H-10*), 1.09 (s, 3H, *3H-11*), 0.99 (s, 3H, *3H-12*), 0.96 – 0.88 (m, 6H, *3H-15a* + *3H-15b*).

^{13}C{H} NMR (101 MHz, $CDCl_3$) δ 213.9 (*C-1*), 139.7 (*C-8*), 139.3 (*C-7*), 136.1 (*C-16*), 128.4 (*C-17*), 127.4 (*C-19*), 126.4 (*C-18*), 58.8 (*C-2*), 53.3 (*C-6*), 50.3 (*C-3*), 47.0 (*C-9*), 45.7 (*C-13*), 39.1 (*C-5*), 37.2 (*C-4*), 29.0 (*C-12*), 26.2 (*C-10*), 25.4 (*C-15a*), 25.3 (*C-15b*), 24.3 (*C-14*), 22.5 (*C-11*).

IR (neat, cm^{-1}): 3057, 3029, 2927, 2869, 1703, 1493, 1464 (fingerprint region excluded).

HRMS (APCI+): calculated for $C_{22}H_{31}O$ ($M^{+}H^{+}$): 311.2369 Found: 311.2377.

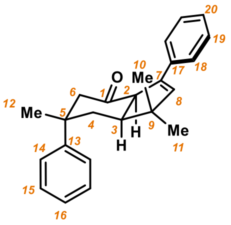

1,1,6-Trimethyl-3,6-diphenyl-1,3a,5,6,7,7a-hexahydro-4*H*-inden-4-one (4aa)

Prepared by following the General Procedure 3a from **3aa** (23 mg, 0.10 mmol, 1.0 eq), phenylacetylene (33 µL, 0.30 mmol, 3.0 eq), and SmI_2 (150 µL, 0.1 M, 15 mol%). (The crude residue was purified by column chromatography R_f = 0.4 (silica gel, hexane:EtOAc = 19:1), to afford the target ketone **4aa** as an amorphous white waxy solid (25.0 mg, 0.076 mmol, 76%).

^{1}H NMR (400 MHz, $CDCl_3$) δ 7.38 – 7.23 (m, 8H, 2*H-14* + *2H-15* + *2H-18* + *2H-19*), 7.23 – 7.17 (m, 2H, *H-16* + *H-20*), 5.87 (d, *J* = 2.6 Hz, 1H, *H-8*), 3.74 (ddd, *J* = 7.1, 2.7, 1.3 Hz, 1H, *H-2*), 2.97 (ddd, *J* = 15.3, 3.1, 1.4 Hz, 1H, *H-3*), 2.34 (ddd, *J* = 13.7, 5.7, 3.0 Hz, 1H, *H-6a*), 2.25 – 2.14 (m, 2H, *2H-4*), 1.85 (dd, *J* = 13.7, 12.2 Hz, 1H, *H-6b*), 1.28 (s, 3H*, 3H-10*), 1.14 (s, 3H, *3H-11*), 1.01 (s, 3H, *3H-12*).

^{13}C{H} NMR (101 MHz, $CDCl_3$) δ 211.8 (*C-1*), 145.3 (*C-17*), 139.5 (*C-8*), 138.7 (*C-7*), 135.6 (*C-13*), 128.5 (*C-18*), 127.9 (*C-19*), 127.1 (*C-20*), 126.2 (*C-14*), 125.9 (*C-16*), 125.7 (*C-15*), 57.6 (*C-2*), 51.1 (*C-3*), 48.8 (*C-6*), 46.4 (*C-5*), 41.8 (*C-9*), 36.9 (*C-4*), 34.4 (*C-10*), 26.0 (*C-12*), 22.7 (*C-11*).

IR (neat, cm^{-1}): 2954, 1703, 1495, 1444 (fingerprint region excluded).

HRMS (ESI+): calculated for $C_{24}H_{27}O$ ($M^{+}H$): 331.2056 Found: 331.2046.

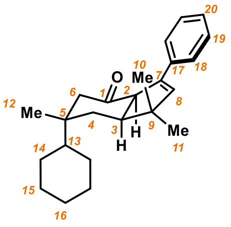

6-Cyclohexyl-1,1,6-trimethyl-3-phenyl-1,3a,5,6,7,7a-hexahydro-4*H*-inden-4-one (4ab)

Prepared by following the General Procedure 3a from **3ab** (46.8 mg, 0.20 mmol, 1.0 eq), phenylacetylene (66 µL, 0.60 mmol, 3.0 eq), Sm metal (5 mg, 0.030, 15 mol%) and SmI_2 (300 µL, 0.1 M, 15 mol%). The crude residue was purified by column chromatography R_f = 0.25 (silica gel, petroleum ether:Et_2O = 19:1) to afford the target product **4ab** as a white amorphous wax (59.1 mg, 0.176 mmol, 89% yield).

^{1}H NMR (400 MHz, $CDCl_3$) δ 7.38 – 7.32 (m, 2H, *2H-19*), 7.30 – 7.23 (m, 2H, *2H-18*), 7.23 – 7.15 (m, 1H, *H-20*), 5.84 (dd, *J* = 2.9, 0.8 Hz, 1H, *H-8*), 4.02 (ddd, *J* = 6.1, 2.8, 1.6 Hz, 1H, *H-2*), 2.38 (d, *J* = 12.8 Hz, 1H, *H-3*), 2.28 (dd, *J* = 13.1, 1.1 Hz, 1H, *H-6a*), 1.83 (d, *J* = 13.1 Hz, 1H, *H-6b*), 1.82 – 1.61 (m, 5H,

H-4a + *2CH₂),* 1.57 – 1.39 (m, 1H, *H-4b*), 1.29 – 0.83 (m, 13H, *3H-10* + *3H-11* + *H-13* + *3CH₂*), 0.81 (s, 3H, *3H-12*).

^{13}C{H} NMR (101 MHz, $CDCl_3$) ^{13}C NMR (101 MHz, $CDCl_3$) δ 213.5 (*C-1*), 139.9 (*C-7*), 139.6 (*C-8*), 136.1 (*C-17*), 128.4 (*C-19*), 127.4 (*C-20*), 126.5 (*C-18*), 59.0 (*C-2*), 51.1 (*C-6*), 49.7 (*C-3*), 46.9 (*C-9*), 40.8 (*C-5*), 40.4 (*C-13*), 35.6 (*C-4*), 27.1 (*CH_2*), 27.0 (*CH_2*), 26.7 (*CH_2*), 26.6 (*CH_2*), 26.4 (*C-10*), 26.2 (*CH_2*), 23.6 (*C-12*), 22.5 (*C-11*).

IR (neat, cm^{-1}): 2925, 2853, 1703, 1493, 1445 (fingerprint region excluded).

HRMS (APCI+): calculated for $C_{24}H_{32}ONa$ (M^+Na^+): 359.2345 Found: 359.2353.

4,4-Dimethyl-6-phenylhexahydropentalen-1(2*H*)-one (6a and 6a’)

Prepared by following the General Procedure 3b from **5** (24.8 mg, 0.20 mmol, 1.0 eq), styrene (69 µL, 0.60 mmol, 3.0 eq), Sm metal (5 mg, 0.03 mmol, 15 mol%) and SmI_2 (300 µL, 0.1 M, 15 mol%). The crude residue was purified by column chromatography to afford two product diastereoisomers (d.r. = 2.0:1).

MINOR

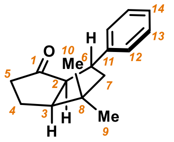

R_f = 0.35 (silica gel, petroleum ether:Et_2O = 17:3) gave the minor diastereoisomer **6a’** as a colorless oil (10.7 mg, 0.047 mmol, 23% yield).

^{1}H NMR (500 MHz, $CDCl_3$) δ 7.30 – 7.21 (m, 4H, *2H-12* + *2H-13*), 7.18 – 7.09 (m, 1H, *H-14*), 3.30 (ddd, *J* = 10.7, 7.7, 6.3 Hz, 1H, *H-6*), 2.75 (dd, *J* = 10.1, 6.3 Hz, 1H, *H-2*), 2.53 (ddd, *J* = 10.1, 8.4, 3.0 Hz, 1H, *H-3*), 2.34 – 2.20 (m, 2H, *2H-5*), 2.06 – 1.93 (m, 2H, *H-4a* + *H-7a*), 1.81 (dddt, *J* = 14.4, 8.9, 5.8, 3.1 Hz, 1H, *H-7b*), 1.74 – 1.67 (m, 1H, *H-4b*), 1.06 (s, 3H, *3H-9*), 0.82 (s, 3H, *3H-10*).

^{13}C{H} NMR (126 MHz, $CDCl_3$) δ 222.7 (*C-1*), 145.6 (*C-11*), 128.6 (*C-12*), 127.4 (*C-13*), 126.2 (*C-14*), 60.9 (*C-2*), 52.4 (*C-3*), 50.4 (*C-6*), 45.8 (*C-7*), 42.5 (*C-8*), 38.0 (*C-5*), 29.0 (*C-9*), 22.7 (*C-4*), 20.4 (*C-10*).

IR (neat, cm^{-1}): 3028, 2954, 2868, 1734, 1602, 1496, 1464, 1404 (fingerprint region excluded).

HRMS (ESI+): calculated for $C_{16}H_{20}ONa$ (M^+Na^+): 251.1406 Found: 251.1413.

MAJOR

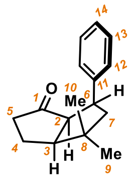

R_f = 0.15 (silica gel, petroleum ether:Et_2O = 17:3) obtained the major diastereoisomer **6a** as a colorless oil (25.1 mg, 0.11 mmol, 55% yield).

^{1}**H NMR** (500 MHz, $CDCl_3$) δ 7.32 – 7.23 (m, 2H, *2H-13*), 7.22 – 7.14 (m,1H, *H-14*), 7.12 – 7.06 (m, 2H, *2H-12*), 3.72 (td, *J* = 12.2, 7.5 Hz, 1H, *H-6*), 3.03 (ddd, *J* = 12.4, 8.1, 1.5 Hz, 1H, *H-2*), 2.47 – 2.38 (m, 1H, *H-3*), 2.18 – 2.11 (m, 2H, *2H-5*), 2.09 – 2.02 (m, 1H, 4*H-a*), 1.99 (t, *J* = 12.5 Hz, 1H, *H-7a*), 1.90 – 1.76 (m, 2H, *H-4b* + *H-7b*), 1.16 (s, 3H, *3H-9*), 1.13 (s, 3H, *3H-10*).

^{13}**C{H} NMR** (126 MHz, $CDCl_3$) δ 220.4 (*C-1*), 140.9 (*C-11*), 128.5 (*C-12*), 128.4 (*C-13*), 126.6 (*C-14*), 56.7 (*C-2*), 53.6 (*C-3*), 47.6 (*C-6*), 45.7 (*C-7*), 41.8 (*C-8*), 40.3 (*C-5*), 29.4 (*C-9*), 24.5 (*C-4*), 23.9 (*C-10*).

IR (neat, cm^{-1}): 2952, 2865, 1730, 1602, 1496, , 1464, 1407 (fingerprint region excluded).

HRMS (ESI+): calculated for $C_{16}H_{20}ONa$ ($M^{+}Na^{+}$): 251.1406 Found: 251.1417.

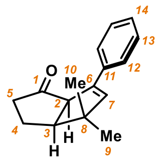

1,1-Dimethyl-3-phenyl-1,3a,5,6,7,7a-hexahydro-4*H*-inden-4-one (6b)

Prepared by following the General Procedure 3b from **5** (24.8 mg, 0.20 mmol, 1.0 eq), phenylacetylene (66 µL, 0.60 mmol, 3.0 eq), Sm metal (5 mg, 0.03 mmol, 15 mol%) and SmI_2 (300 µL, 0.1 M, 15 mol%). The crude residue was purified by column chromatography R_f = 0.25 (silica gel, petroleum ether:Et_2O = 9:1) to afford the target product **6b** as a yellow oil (42.6 mg, 0.190 mmol, 95% yield).

^{1}**H NMR** (400 MHz, $CDCl_3$) δ 7.62 – 7.53 (m, 2H, *2H-12*), 7.35 – 7.28 (m, 2H, *2H-13*), 7.25 – 7.20 (m, 1H, *H-14*), 5.92 (d, *J* = 2.5 Hz, 1H, *H-7*), 3.92 – 3.84 (m, 1H, *H-2*), 2.72 (q, *J* = 7.8 Hz, 1H, *H-3*), 2.39 – 2.15 (m, 2H, *2H-5*), 2.08 – 1.82 (m, 2H, *2H-4*), 1.18 (s, 3H, *3H-9*), 1.15 (s, 3H, *3H-10*).

^{13}**C{H} NMR** (101 MHz, $CDCl_3$) δ 217.6 (*C-1*), 139.1 (*C-7*), 137.5 (*C-6*), 135.1 (*C-11*), 128.2 (*C-12*), 127.5 (*C-14*), 127.0 (*C-13*), 58.8 (*C-2*), 51.5 (*C-3*), 46.9 (*C-8*), 39.4 (*C-5*), 29.9 (*C-9*), 23.4 (*C-4*), 22.5 (*C-10*).

IR (neat, cm^{-1}): 2956, 1735, 1494, 1467, 1445, 1407 (fingerprint region excluded).

HRMS (ESI+): calculated for $C_{16}H_{18}ONa$ ($M^{+}Na^{+}$): 249.1250 Found: 249.1260.

2.3 Scale up Procedures

2.3.1 Scale-up synthesis of 1,1,6-Trimethyl-3-phenyloctahydro-4*H*-inden-4-one (4a)

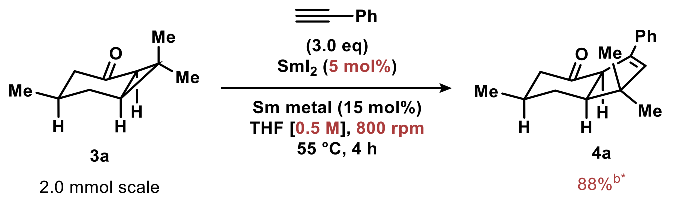

An oven-dried microwave vial containing a magnetic stirrer-bar was cooled under a high-pressure stream of N_2 (1 min), charged with Sm metal (50 mg, 0.30 mmol, 15 mol%), immediately sealed and placed under vacuum. The vial was cycled between vacuum and N_2 (3 times) and left under a positive pressure of N_2. The vial was charged with **3a** (304 mg, 2.00 mmol, 1.0 eq), phenylacetylene (666 µL, 6.0 mmol, 6.0 eq), and the vial was further flushed with N_2 (5 min). The vial was charged with THF (3.0 mL) and placed in a pre-heated oil bath (55 °C, 1 min) and SmI_2 (1.00 mL, 0.1 M, 5 mol%) was introduced by syringe and the reaction was stirred and heated (800 rpm, 55 °C, 240 min). The reaction was quenched by injection of compressed air and was cooled (rt, 15 min), CH_2Cl_2 (15 mL) was added, and the supernatant was decanted and filtered through a silica gel pad (2.5 cm x 8 cm) using CH_2Cl_2 (50 mL) as the eluent and the filtrate concentrated *in vacuo*. The crude residue was purified by column chromatography R_f = 0.30 (silica gel, petroleum ether:Et_2O = 9:1) to afford the target product as a pale-yellow oil that solidified on standing (452 mg, 1.78 mmol, 89% yield).

2.3.2 Scale-up synthesis of Bis(4,4-dimethyl-2-phenylcyclopent-2-en-1-yl)methanone (8)

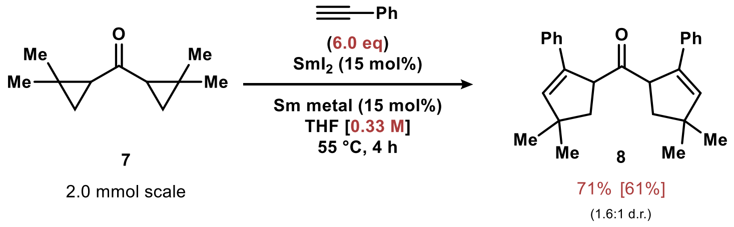

An oven-dried microwave reaction vial containing a magnetic stirrer-bar was cooled under a high-pressure stream of N_2 (1 min), then charged with Sm metal (50 mg, 0.30 mmol, 15 mol%), immediately sealed and placed under vacuum. The vial was cycled between vacuum and N_2 (3 times) and left under a positive pressure of N_2. The vial was charged with **7** (332 mg, 2.00 mmol,1.0 eq), phenylacetylene (1.32 mL, 12.0 mmol,

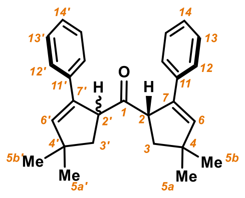

6.0 eq), and the vial was further flushed with N_2 (5 min). The vial was charged with THF (6.0 mL) and placed in a pre-heated oil bath (55 °C, 1 min) and SmI_2 (3.00 mL, 0.1 M, 15 mol%) was introduced by syringe and the reaction was stirred and heated (400 rpm, 55 °C, 240 min). The reaction was quenched by injection of compressed air and was cooled (rt, 15 min), CH_2Cl_2 (15 mL) was added, and the supernatant was decanted and filtered through a silica gel pad (2.5 cm x 8 cm) using CH_2Cl_2 (50 mL) as the eluent and the filtrate concentrated *in vacuo*. Care was taken to ensure that the Sm^0 remained in the microwave vial when decanting the solution. The crude residue was purified by column chromatography R_f = 0.20 (silica gel, petroleum ether:Et_2O = 19:1) to afford the target product **8** as a yellow solid and mixture of diastereoisomers (521 mg, 1.41 mmol, 71% yield, d.r. = 1.6:1). The resulting Sm^0 was dried under vacuum along with a stirrer bar (rt, 16 h), and used again in the above procedure and the product **8** was obtained as a yellow solid and mixture of diastereoisomers (455 mg, 1.22 mmol, 61% yield, d.r. = 1.6:1).

^{1}H NMR (400 MHz, $CDCl_3$) δ 7.26 – 7.13 (m, 26H, *10H-Ar minor* + *10H-Ar major*), 6.00 (d, *J* = 1.5 Hz, 2H, *H-6* + *H-6' minor*), 5.97 (d, *J* = 1.6 Hz, 2H, *H-6* + *H-6' major*), 4.40 – 4.31 (m, 4H, 2H-2 + 2-H-2'), 2.17 (dd, *J* = 12.9, 9.7 Hz, 2H, *H-3a* + *3a' minor*), 2.07 (dd, *J* = 13.0, 9.6 Hz, 2H, *H-3a* + *3a' major*), 1.96 (dd, *J* = 12.9, 5.3 Hz, 2H, *H-3b* + *3b' minor*), 1.89 (dd, *J* = 12.9, 5.0 Hz, 2H, *H-3b* + *3b' major*), 1.15 – 1.11 (m, 31H, *4CH$_3$ minor* + *4CH$_3$ major*).

^{13}C{H} NMR (101 MHz, $CDCl_3$) δ 213.0 (*C-1 major*), 212.1 (*C-1 minor*), 141.0 (*C-6/6' minor*), 140.9 (*C-6/6' major*), 138.9 (*C-7/7' major*), 138.6 (*C-7/7' minor*), 136.24 (*C-11/11' minor*), 136.20 (*C-11/11' major*), 128.3 (*C-13/13' major*), 128.2 (*C-13/13' minor*), 127.1 (*C-14/14' major*), 127.0 (*C-14/14' minor*), 126.2 (*C-12/12' major*), 126.0 (*C-12/12' minor*), 57.9 (*C-2/2' major*), 57.3 (*C-2/2' minor*), 45.4 (*C-4/4' major*), 45.1 (*C-4/4' minor*), 43.4 (*C-3/3' minor*), 43.1 (*C-3/3' major*), 29.4 (*C-5a/5a' major*), 29.2 (*C-5a/5a' minor*), 28.7 (*C-5b/5b' major*), 28.6 (*C-5b/5b' minor*).

IR (neat, cm^{-1}): 3055, 2952, 2863, 1709, 1463 (fingerprint region excluded).

HRMS (APCI+): calculated for $C_{27}H_{29}O$ (M^+H^+): 369.2224 Found: 369.2230.

2.4.1 Summary of unsuccessful reactions

Reactions conducted using General Procedure 3a/b. In the case of reactions involving **1b**, only conditions B were trialled as these generally gave higher yields. In the case of reactions involving **3a**, conditions A were trialled, and in some cases conditions B also, owing to the substrate generally working well under these conditions.

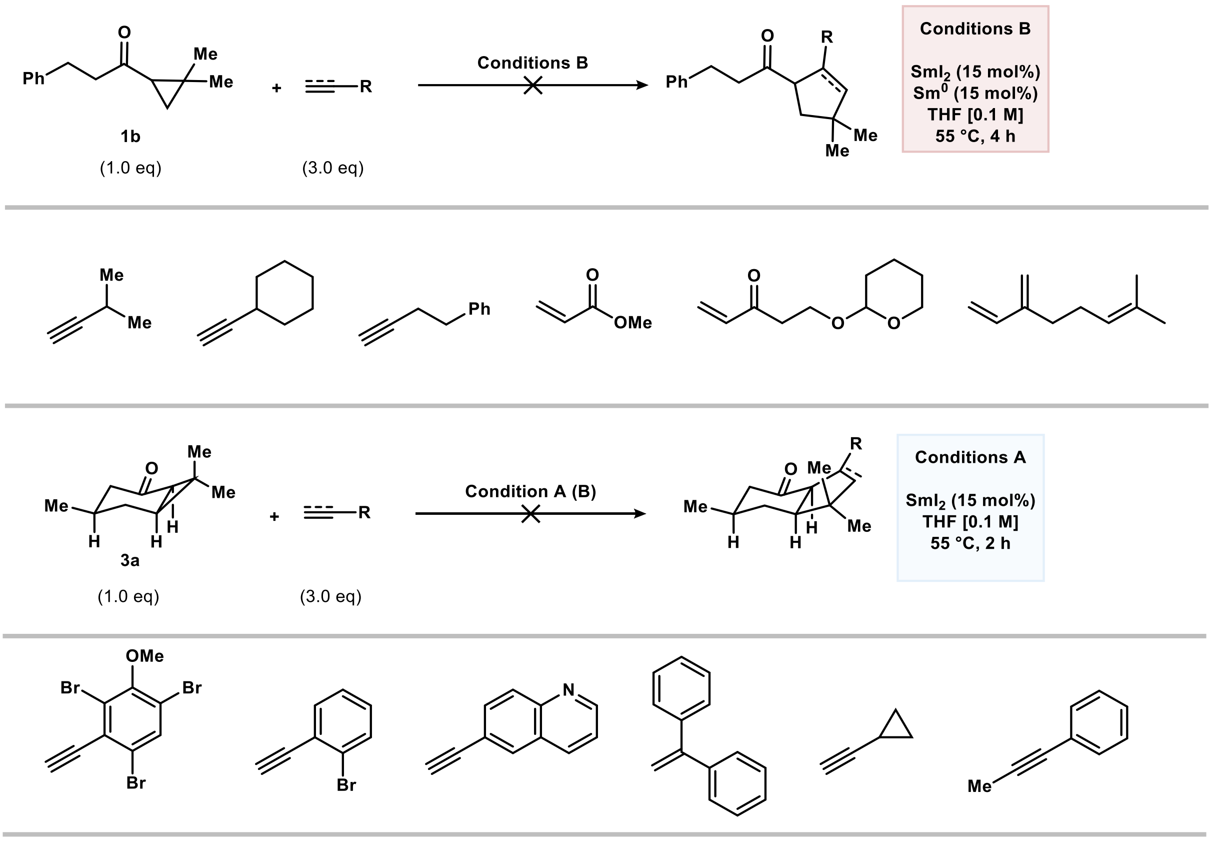

2.4.2 Miscellaneous Information

Photographs were taken during smaller scale reactions of *bis* cyclopropyl ketone **7** using conditions A and B. See **Figure SI.1** where the left vial (yellow suspension, due to the precipitation of Sm(III) salts) is of the reaction mixture prior to quenching using conditions A, and the right vial (blue solution) is of the mixture prior to quenching using conditions B.

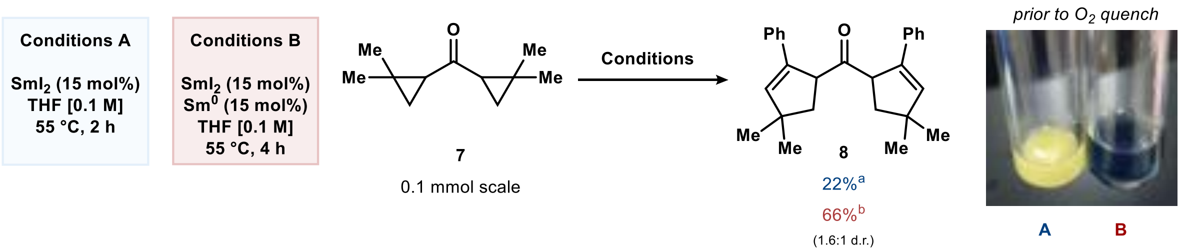

Figure SI.1 – Photographs of reactions using conditions A and B with *bis*-cyclopropyl ketone **7**.

2.5 Time dependence study using conditions A and ketones 1b, 1f, 1s, and 3a

2.5.1 Procedure for monitoring SmI_2-catalyzed intermolecular coupling reactions

An oven-dried microwave reaction vial containing a magnetic stirrer-bar was cooled under a stream of N_2 (1 min), immediately sealed and placed under a positive pressure of N_2. The vial was charged with the corresponding cyclopropyl ketone (0.10 mmol, 1.0 eq), and radical trap (0.30 mmol, 3.0 eq), and further flushed with N_2 (15 min). The vial was charged with THF (0.50 mL) and placed in a pre-heated oil bath (55 °C, 1 min). Freshly prepared SmI_2 (0.150 mL, 0.10 M, 15 mol%) was introduced by syringe and the reaction was stirred and heated (400 rpm, 55 °C, 1-120 min). The reaction was quenched by injection of compressed air and cooled (rt, 5 min) and filtered through a silica gel pad (1.5 cm x 4 cm) using CH_2Cl_2 (15 mL) as the eluent. To obtain ^1H qNMR yields for reactions, the filtrate was concentrated *in vacuo* and CH_2Br_2 in $CDCl_3$ (1 mL, 0.05 M, 0.05 mmol) was added to the crude residue and samples were submitted directly for ^1H qNMR.

2.5.2 Time-dependence study using conditions A and ketone 1f

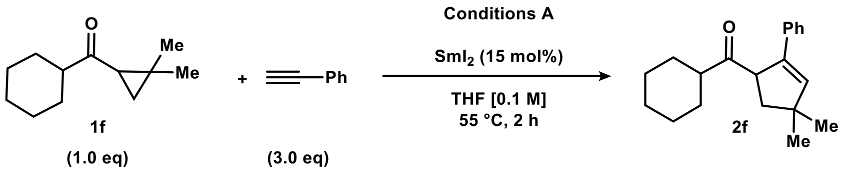

Study conducted using freshly prepared and titrated SmI_2, **1f** (18.0 mg, 0.10 mmol, 1.0 eq), phenylacetylene (33 µL, 0.30 mmol, 3.0 eq), and SmI_2 (150 µL, 0.1 M, 15 mol%). Data plotted using Origin 2022b software with single data points and non-linear fitting shown in **Figure SI.2** using chemical shifts for diagnostic protons in the starting material and product (highlighted).

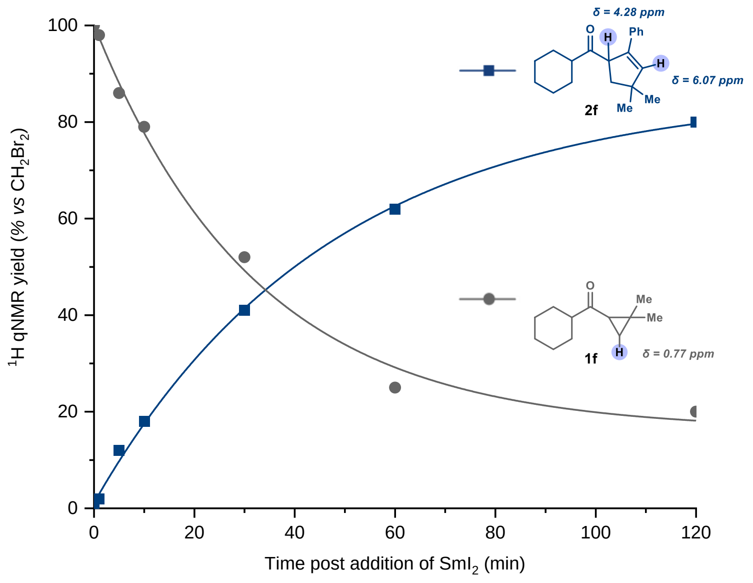

Figure SI.2 - Reaction progress monitoring using Conditions A with substrate **1f**; grey circles and squares correspond to the conversion of starting material and product formation, respectively, determined by ^{1}H qNMR using CH_2Br_2 as internal standard; all lines represent non-linear best fit curves.

Table SI.1 - Non-linear fitting data for Figure SI.2

$$y = A_1 \cdot e^{\left(\frac{-x}{t_1}\right)} + y_0$$

		Value	Standard Error	t- value	Prob>|t|	Dependency
**2f**	y_0	86.19321	2.50612	34.39316	4.26402E-6	0.95404
	A_1	-85.05314	2.39967	-35.44362	3.78179E-6	0.90712
	t_1	46.7861	3.40856	13.72607	1.63212E-4	0.835
**1f**	y_0	16.11972	3.45906	4.66014	0.00959	0.89681
	A_1	83.9909	3.52818	23.80573	1.84643E-5	0.79528
	t_1	32.24474	3.94271	8.17832	0.00122	0.72392

	Number of points	Degrees of Freedom	Reduced Chi-Sqr	Residual Sum of Residual	R-Square(COD)	Adj. R-Square
**2f**	7	4	2.0207	8.08282	0.9986	0.99791
**1f**	7	4	8.64242	34.56967	0.99487	0.99231

2.5.3 Time dependence study using conditions A and ketones 1b, 1f, 1s, and 3a

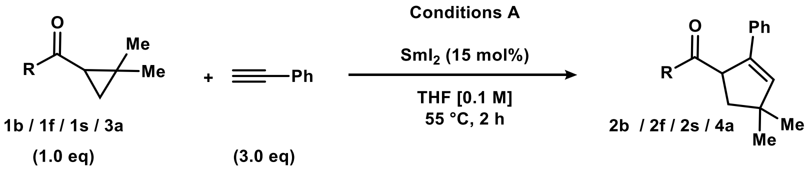

Study conducted using freshly prepared and titrated SmI_2, either **3a** (15.2 mg, 0.10 mmol, 1.0 eq), **1f** (18.0 mg, 0.10 mmol, 1.0 eq), **1b** (20.2 mg, 0.10 mmol, 1.0 eq), or **1s** (24.2 mg, 0.10 mmol, 1.0 eq) with phenylacetylene (33 µL, 0.30 mmol, 3.0 eq), and SmI_2 (150 µL, 0.1 M, 15 mol%). Data plotted using Origin 2022b software with single data points and non-linear fitting shown in **Figure SI.3** using chemical shifts for diagnostic protons in the starting material and product (highlighted).

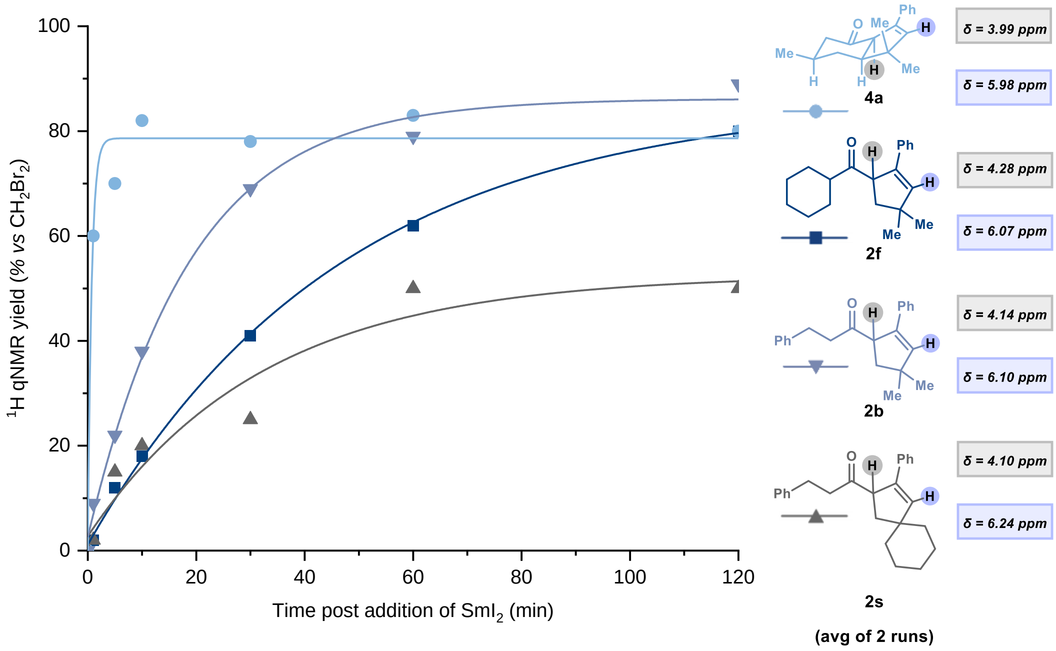

Figure SI.3 - Reaction progress monitoring using Conditions A with substrates **3a** (circle), **1f** (square), **1s** (right-side up triangle; average of two runs) and **1b** (upside down triangle); all lines represent non-linear best fit curves.

Table SI.2- Non-linear fitting data for Figure SI.3.

$$y = A_1 \cdot e^{\left(\frac{-x}{t_1}\right)} + y_0$$

		Value	Standard Error	t- value	Prob>|t|	Dependency
4a	y_0	78.63478	2.3102	34.03815	4.44419E-6	0.28949
	A_1	-78.60815	5.64339	-13.92924	1.54051E-4	0.21152
	t_1	0.69833	0.14528	4.8069	0.0086	0.15165
2f	y_0	86.19321	2.50612	34.39316	4.26402E-6	0.95404
	A_1	-85.05314	2.39967	-35.44362	3.78179E-6	0.90712
	t_1	46.7861	3.40856	13.72607	1.63212E-4	0.835
2b	y_0	86.18611	2.42716	35.50901	3.75409E-6	0.79002
	A_1	-83.62292	2.89027	-28.93252	8.49484E-6	0.64023
	t_1	18.93829	2.03359	9.31275	7.39899E-4	0.59334
2s	y_0	52.58162	7.11643	7.38876	0.00179	0.89716
	A_1	-49.82973	7.25536	-6.86799	0.00235	0.7959
	t_1	32.3067	13.6864	2.3605	0.07762	0.72448

	Number of points	Degrees of Freedom	Reduced Chi-Sqr	Residual Sum of Residual	R-Square(COD)	Adj. R-Square
4a	7	4	26.54383	106.17533	0.9799	0.96984
2f	7	4	2.0207	8.08282	0.9986	0.99791
2b	7	4	8.65919	34.63675	0.99542	0.99312
2s	7	4	36.45627	145.82508	0.94178	0.91267

2.6 SmI_2-catayzed epimerization of *bis*-cyclopropylketone 7

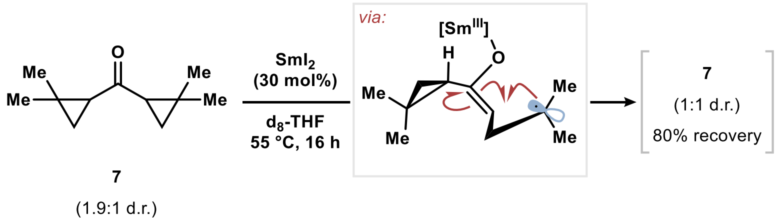

Diastereomerically enriched **7** was obtained through careful column chromatography in pentane:Et_2O (19:1). $SmI_2(d_8\text{-THF})_5$ was prepared according to section 1.2 on 1 mL scale 4 h before use using thoroughly degassed d_8-THF (Karl Fischer titrated as containing 90 ppm H_2O). Then, an oven-dried microwave reaction vial containing a magnetic stirrer-bar was cooled under a high-pressure stream of N_2 (1 min), immediately sealed and placed under vacuum. The vial was cycled between vacuum and N_2 (3 times) and left under a positive pressure of N_2. The vial was charged with **7** (16.6 mg, 0.10 mmol,1.0 eq) followed by d_8-THF (0.35 mL) and placed in a pre-heated oil bath (55 °C, 1 min) and $SmI_2(d_8\text{-THF})_5$ (300 µL, 0.1 M, 30 mol%) was introduced by syringe and the reaction was stirred and heated (400 rpm, 55 °C, 16 h). After this time, the blue solution had turned yellow and a white precipitate had formed. The contents of the vial were then passed through a small silica plug with $CDCl_3$ (0.5 mL) as the eluent directly into an oven-dried and nitrogen flushed vial and CH_2Br_2 (7.0 µL, 0.10 mmol) was added and the solution thoroughly mixed. An aliquot of the solution was taken and added to an oven-dried and nitrogen flushed NMR tube and submitted for ^1H qNMR.

Furthermore, reactions run with $SmI_2(d_8\text{-THF})_5$ (15 mol%) and a control sample (no SmI_2) were also submitted for ^1H qNMR. Subsequently, the control sample was heated (55 °C, 16 h) in d_8-THF, then resubmitted for ^1H qNMR. All spectra are shown below in Figure SI.4.

Note: Due to the modified work-up some, Sm salts were able to pass through the silica plug which meant ^1H qNMR spectra for these experiments had to be acquired on a higher field (700 MHz) instrument. Furthermore, the large H_2O signal evident in the ^1H qNMR spectra is due to water present in the silica gel and not from the d_8-THF (see control sample).

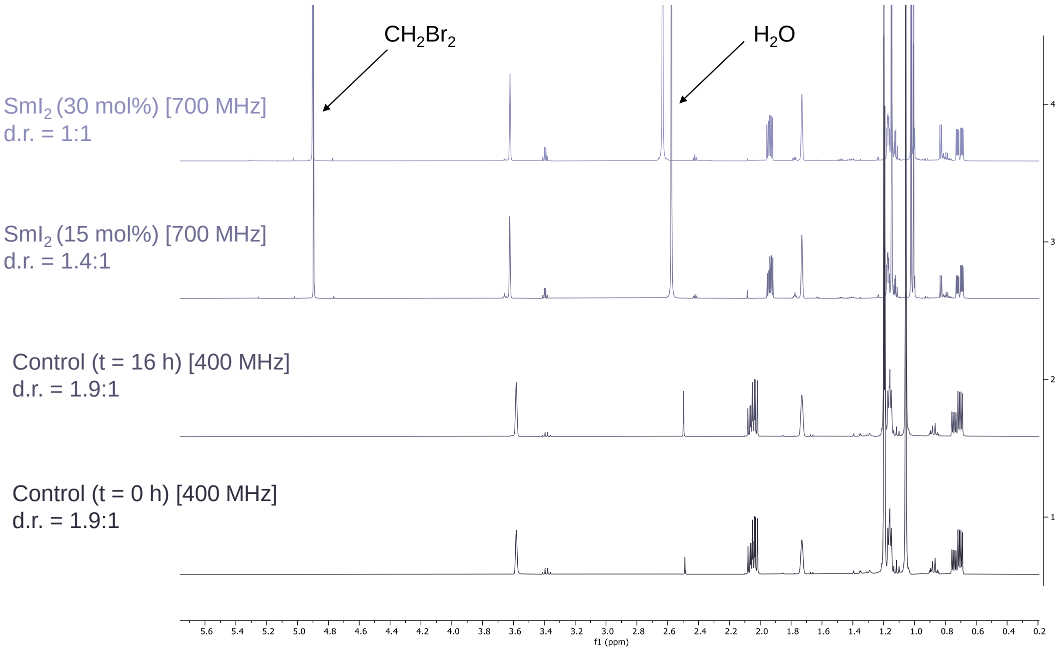

Figure SI.4 – ^{1}H qNMR spectra in d_8-THF (and $CDCl_3$) obtained for epimerization study of *bis*-cyclopropyl ketone *7*.

3. Computational studies

3.1 Re-evaluation of computational methodology

Our previous studies of SmI_2-catalyzed coupling reactions employed DFT in conjunction with effective core potentials (ECPs) and associated valence basis sets for the heavy elements, and all-electron basis sets for the lighter atoms, with corrections for solvation (PCM in THF).[24,25] It is notable that the single electron transfer (SET) step in the alkene insertion reaction described in Figure 2 of reference [6] requires more than 30 kcal/mol, rather higher than suggested experimentally. Large SET energies were also computed for the SmI_2-catalysed coupling of cyclopropyl ketones and alkynes.[6] The SET step involves transitioning between electronic states with different spin multiplicities (from a septet to a quintet) but, while our computational approach used to date incorporates the effects of scalar relativity *via* the use of ECPs, it does not consider spin-orbit coupling (SOC). Deacon and co-workers found that formation of ketyl radicals via SET in Sm-ketone adducts is endothermic when only scalar relativistic effects are included, but exothermic when SOC is taken into account.[26] We therefore undertook an assessment of SOC effects, employing Douglas-Kroll-Hess 4th order relativistic calculations incorporating spin-orbit coupling terms (DKHSO). The calculations were performed utilizing an all-electron SARC basis set for Sm and an all-electron Jorge basis set for I,[27,28] and cc-pVTZ basis sets for the remaining elements, and focused on one of our previously studied SmI_2-catalyzed coupling reactions.[6] Note that the all-electron relativistic energies were obtained from single point calculations at the previously-determined geometries from ECP basis set calculations.

Figure SI.5 shows that on replacing the cc-pTZV + ECPs method (blue profile) with the updated method incorporating DKHSO/all electron basis sets (red profile), the SET barrier is reduced by 12 kcal mol^{-1}, in closer alignment with experimental observations, whilst the energy differences between subsequent species are rather similar in most cases. This suggests that the prior method adeptly predicts energy differences between species on the quintet surface but tends to overestimate the difficulty of reaching the quintet potential energy surface. However, it is not clear at this point if we can definitively attribute the lowered SET barrier solely to SOC effects. We therefore conducted Douglas-Kroll-Hess 2nd order scalar relativistic calculations (DKH) in conjunction with the SARC basis set for Sm and Jorge basis set for I on the same reaction. The outcomes (green profile in Figure SI.5) indicate that the energies are predominantly unaffected by SOC (the energies on the red and green profiles all differ

by less than 3 kcal mol^{-1}), including the SET step, which is only 0.7 kcal mol^{-1} different between the scalar and SOC calculations. The substantial lowering of the quintet potential energy surface is therefore attributed primarily to the use of all-electron basis sets and a scalar relativistic Hamiltonian, with SOC a secondary factor. The diminished SET barriers in all-electron calculations compared to those using ECPs likely stem from the more accurate treatment of scalar relativity, and possibly correlation effects, in the former, resulting in an SET process that energetically aligns more closely with experimental observations.

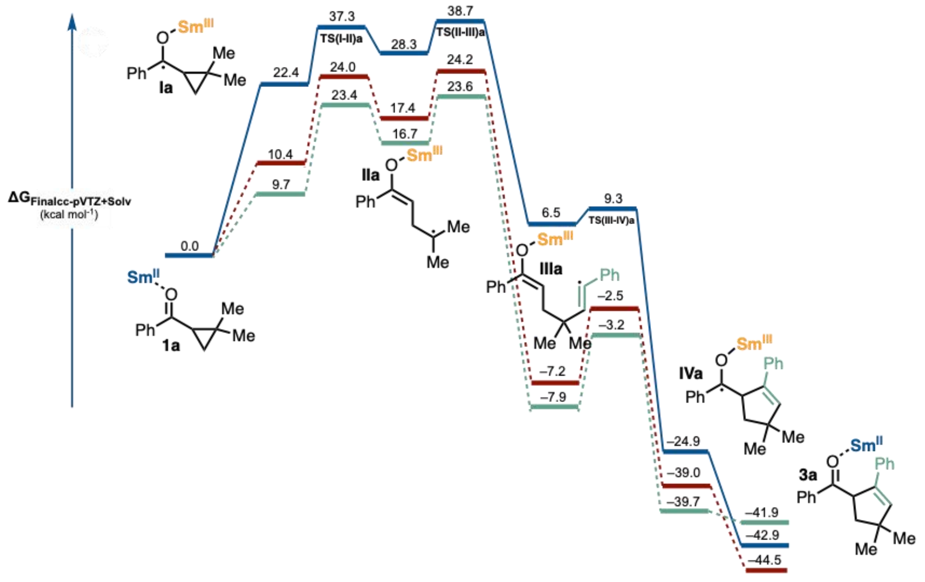

Figure SI.5 - Gibbs energy profiles of SmI_2-catalyzed intermolecular coupling reactions between cyclopropyl ketone and alkyne,[6] computed by different methods. The blue profile depicts the result calculated by cc-pVTZ + ECPs single point calculations (with PCM corrections for THF). The red profile depicts the result incorporating DKHSO and all electron basis sets for Sm and I (with PCM corrections for THF). The green profile depicts the result implementing DKH and all electron basis sets for Sm and I (with PCM corrections for THF).

In conclusion, this study establishes that both scalar and spin-orbit relativistic all-electron calculations yield a substantial reduction in the energy required to access the excited potential energy surfaces of SmI_2-catalyzed coupling reactions *vs* calculations employing ECPs along with associated valence basis sets, and that the energies of the subsequent steps around the catalytic cycle are in general largely unaffected. Furthermore, the effects of SOC are found to be minor. Noting that in some cases SCF convergence with DKHSO was more problematic to obtain than at the scalar level, we herein adopt the revised all-electron approach at the scalar (DKH) level.

3.2 Computational details

Geometry optimizations were performed using Gaussian 16, Revision C.01.,[29] employing the PBE0 functional [30] and Dunning's correlation-consistent double-zeta + polarization basis sets (cc-pVDZ) [31] for C, H, and O. For Sm and I, Stuttgart-Köln effective core potentials (ECPs) and associated valence basis sets were used. [32,33] The dispersion corrections from Grimme's D3 model [34] with Becke-Johnson damping factors [35] were incorporated during the geometry optimizations. Harmonic vibrational frequency calculations were performed at 328.15 K using the same level of theory to confirm the nature of stationary points as either true minima or transition states and to provide thermodynamic corrections.

Subsequent single-point calculations were carried out on the optimized geometries, including the Douglas-Kroll-Hess 2nd order scalar relativistic Hamiltonian, [36] and utilizing the SARC basis set for Sm, Jorge basis set for I,[27,28] and Dunning's correlation-consistent triple-zeta + polarization basis sets (cc-pVTZ) [30] for the remaining elements. The resulting energies (E_{vacuum}) were treated as pure electronic energies and subjected to analysis for spin density distributions. Further calculations were performed with the inclusion of solvent effects using the PCM [37] in THF. The energies obtained with solvent effects (E_{sol}) and the enthalpic corrections derived from frequency calculations collectively yielded the solvation enthalpies (H_{sol}). The entropic contributions were determined from frequency calculations and adjusted using the quasi-harmonic approximation proposed by Grimme.[38] Specifically, the $-TS$ terms were corrected directly using GoodVibes,[39] with a cut-off frequency set to 100 cm^{-1}.

Previous calculations on systems of this type suggest that reactivity takes place via the quintet spin state, with antiparallel coupling of ligand radical anion to Sm(III) $4f^5$ metal center. Hence the calculations have been performed with the spin multiplicity of 7 for the initial reactants and final products and with the spin multiplicity of 5 for all other intermediates and transition states.

3.3 Coordinates and Energies

Table SI.3. Cartesian coordinates (in Å) of geometries optimized at PBE0 functional with cc-pVDZ basis sets for C, H, and O, and ECPs for Sm and I, single point energies computed in vacuum E_{vacuum} and THF (PCM) E_{sol}, enthalpies H_{sol}, $-TS$ terms corrected by Grimme's method, and Gibbs energies G_{sol} (in Hartree)

1f_Reactant

$\boldsymbol{E_{vacuum}}$ = -26109.9349

$\boldsymbol{E_{sol}}$ = -26109.9579

$\boldsymbol{H_{sol}}$ = -26109.1040

$\boldsymbol{-TS}$ = -0.1460

$\boldsymbol{G_{sol}}$ = -26109.2730

H	-4.65530300	1.58807700	-1.40017200
H	-3.15284000	3.16666300	-2.66059900
H	-1.69145400	2.57344100	-1.68719700
H	-5.00653000	-4.07233700	1.52160100
H	-1.71138000	2.67299500	0.79766400
H	-5.28402800	3.92847400	0.34872900
C	-3.05474900	0.59318700	-0.33504300
C	-3.64655500	1.75097900	-1.01555900
C	-2.76825800	2.76404300	-1.72126200
C	-3.41607000	3.17901400	-0.45138500
C	-4.62210200	4.08295200	-0.51841600
O	-1.84302400	0.51562700	-0.13075300
Sm	0.59922400	-0.00743000	-0.06587500
O	-0.15383800	-0.83390700	-2.41344300
C	-0.57306100	-0.05298600	-3.52896500
C	-1.84899200	-0.73684600	-3.98713800
C	-1.55237800	-2.22428000	-3.72819300
C	-0.34769000	-2.20334100	-2.77203900
I	1.39148500	2.79700800	-1.43280100
I	-0.03806000	-2.85661200	1.31736100
O	2.75665800	0.12733000	1.39581000
C	3.96106300	0.79757900	0.99182100
C	4.81795300	0.87034900	2.24358800
C	4.41711300	-0.41248400	2.96711400
C	2.92456400	-0.46636300	2.69169700
O	0.09787300	1.31675700	2.11728600
C	0.77131200	2.52646800	2.50478600
C	0.05495200	3.00201600	3.75671100
C	-0.37833100	1.68168200	4.38826100
C	-0.78152900	0.87878200	3.16459300
O	2.59079400	-1.16175200	-1.28225600
C	3.08768600	-0.75964800	-2.56764900
C	4.39349400	-1.51363300	-2.74373000
C	4.09458900	-2.81647700	-2.00707000
C	3.29947700	-2.31913100	-0.81228900
H	4.44783200	0.19832600	0.20236600
H	3.68357200	1.77086400	0.56185100
H	2.35806700	0.13095700	3.42881100
H	2.49502500	-1.47819400	2.66336900
H	4.54446200	1.75176300	2.84657000
H	5.89096200	0.93462100	2.01574200
H	4.64731500	-0.40669500	4.04154000
H	4.92226700	-1.28162900	2.51491700
H	1.82893100	2.28463400	2.70816500
H	0.73610700	3.22431200	1.65622100
H	-1.81995400	1.09865400	2.86508800
H	-0.67035800	-0.21061700	3.27016500
H	-0.82764000	3.60460400	3.48812000
H	0.70094200	3.61161800	4.40360300
H	-1.19856000	1.78439900	5.11224000
H	0.47184800	1.20285700	4.90118900
H	2.35010400	-1.04807200	-3.33696900
H	3.17938000	0.33619400	-2.57078900
H	3.96385400	-2.01085000	0.01466200
H	2.56498800	-3.03404500	-0.41470000
H	5.21882500	-0.97416700	-2.25068200
H	4.66077600	-1.65212200	-3.80054400
H	4.99288600	-3.37594900	-1.71130100
H	3.47267500	-3.47604300	-2.63404400
H	-0.68156500	0.98461700	-3.18961200
H	0.20881200	-0.08176900	-4.31220700
H	0.56593100	-2.57667000	-3.26997600

H	-0.49083500	-2.76715700	-1.83931800
H	-2.09065900	-0.52088000	-5.03708900
H	-2.69043500	-0.39216600	-3.36726000
H	-1.29948600	-2.75243800	-4.65874300
H	-2.41446000	-2.73879600	-3.28236900
H	-2.15452800	4.39059300	0.78124000
H	-4.30270200	5.13769300	-0.51297500
H	-3.15562800	3.23251900	1.69422000
H	-5.20959100	3.91457400	-1.43308600
C	-2.56050800	3.36665400	0.77563600
C	-3.96972200	-0.51580300	0.12621100
C	-4.60323800	-2.00889300	2.05942600
C	-3.73573900	-0.84139900	1.60517100
C	-3.72785000	-1.76421600	-0.73769900
C	-4.58780600	-2.93465300	-0.27623200
C	-4.35701500	-3.24296500	1.19869100
H	-5.01527400	-0.18873100	-0.01255000
H	-5.67062100	-1.72344400	2.00368100
H	-4.39328600	-2.23327900	3.11729900
H	-2.67295100	-1.10305700	1.73440000
H	-3.93686900	0.05268800	2.21907500
H	-2.66104500	-2.03191800	-0.65146700
H	-3.93000200	-1.53235100	-1.79661300
H	-4.36473100	-3.82003700	-0.89337700
H	-5.65453300	-2.69528300	-0.44403100
H	-3.31300900	-3.57371700	1.33836400

1f_Int I

E$_{vacuum}$ = -26109.9185

E$_{sol}$ = -26109.9318

H$_{sol}$ = -26109.0892

–TS = -0.1417

G$_{sol}$ = -26109.2441

H	-4.53219100	1.29383500	-1.54701800
H	-3.10422800	2.53558800	-3.17527400
H	-1.62023100	2.26160400	-2.11475600
H	-4.84410700	-4.08534200	1.78105000
H	-1.63599900	2.91631900	0.25075600
H	-5.21845700	4.03623000	-0.42418700
C	-2.95886900	0.56068400	-0.28314000
C	-3.53236600	1.56800600	-1.19362900
C	-2.70456900	2.38081600	-2.16947400
C	-3.36731200	3.08500800	-1.02385400
C	-4.57434600	3.93934900	-1.31451900
O	-1.63032700	0.44172700	-0.15564800
Sm	0.42623200	0.02777600	-0.01402800
O	-0.18799000	-1.31542200	-2.00651700
C	-1.00358600	-0.75208500	-3.06338800
C	-1.50348300	-1.94097400	-3.86805700
C	-0.42062800	-2.98798300	-3.62530500
C	-0.07948500	-2.74036100	-2.16957100
I	1.33326000	2.40401100	-1.77220100
I	0.11993100	-2.50248000	1.73184900
O	2.62545900	0.43257900	1.32139500
C	3.78657700	1.13848600	0.84193300
C	4.61573000	1.43683800	2.07736400
C	4.29968600	0.23685900	2.96388600
C	2.82153200	0.02910800	2.69161300
O	0.12096200	1.55549200	1.92249400
C	0.74624000	2.83747100	2.13597200
C	-0.19461000	3.59762000	3.05430800
C	-0.83920000	2.47129900	3.85755500
C	-1.01762500	1.40021900	2.79998700
O	2.49290000	-1.17895800	-1.04272100
C	2.95332400	-1.00080600	-2.39826800
C	4.29659200	-1.70341700	-2.46216800
C	4.09880300	-2.84366200	-1.46895100
C	3.30852600	-2.15863500	-0.37021500
H	4.32661800	0.48273400	0.13729900
H	3.44425400	2.02336700	0.28908000
H	2.19157000	0.66686400	3.33493800
H	2.47536700	-1.00830200	2.79273200
H	4.27286500	2.36874600	2.55558400
H	5.68396300	1.54852700	1.84578900
H	4.50808800	0.40517900	4.02942500
H	4.87822800	-0.64346200	2.63946500
H	1.72711300	2.66554200	2.61044000
H	0.90321500	3.30952500	1.15576300

H	-1.93128500	1.54610600	2.20498400
H	-1.00125700	0.37062300	3.18304800
H	-0.95553800	4.13040700	2.46475600
H	0.33735000	4.33225400	3.67446300
H	-1.79284400	2.75558400	4.32292800
H	-0.15941800	2.12320900	4.65231800
H	2.21882800	-1.46434700	-3.07789600
H	2.98539200	0.07747100	-2.60457900
H	3.97368200	-1.63480000	0.33705800
H	2.64244500	-2.81761700	0.20346600
H	5.09961300	-1.03092900	-2.11905200
H	4.54476000	-2.03784300	-3.47887600
H	5.03735200	-3.27903000	-1.09904300
H	3.50509600	-3.65278200	-1.92502300
H	-1.80221700	-0.16042300	-2.59835300
H	-0.35838900	-0.08114800	-3.65237700
H	0.93790500	-3.03350000	-1.87993100
H	-0.78984200	-3.23292200	-1.48600500
H	-1.64334700	-1.69201300	-4.92873200
H	-2.46591500	-2.29540500	-3.46847000
H	0.45596500	-2.79579600	-4.26575000
H	-0.75802200	-4.01806300	-3.80496100
H	-2.11490400	4.59147000	-0.11441100
H	-4.27504900	4.95580800	-1.62299700
H	-3.06925200	3.62548100	1.04415100
H	-5.18450400	3.51208000	-2.12532500
C	-2.49733000	3.57970600	0.10104000
C	-3.81620000	-0.56603800	0.21984700
C	-4.50674900	-1.98347400	2.21250900
C	-3.65279300	-0.81882400	1.72320500
C	-3.55486500	-1.86544400	-0.56208500
C	-4.39992500	-3.03267200	-0.06458000
C	-4.20620800	-3.25725900	1.43050200
H	-4.86833600	-0.27438700	0.04220700
H	-5.57761400	-1.72961200	2.09763700
H	-4.33885000	-2.14802800	3.28960300
H	-2.59181900	-1.04089300	1.92466900
H	-3.90472100	0.10249000	2.27477100
H	-2.48481900	-2.11337700	-0.45524600
H	-3.74224100	-1.68541400	-1.63463500
H	-4.15219700	-3.94902100	-0.62692000
H	-5.46720800	-2.82261600	-0.26630200
H	-3.15949500	-3.55560800	1.61901600

1f_TS I

$\boldsymbol{E}_{\text{vacuum}}$ = -26109.9045

$\boldsymbol{E}_{\text{sol}}$ = -26109.9179

$\boldsymbol{H}_{\text{sol}}$ = -26109.07716

–*TS* = -0.1419

$\boldsymbol{G}_{\text{sol}}$ = -26109.2325

H	-4.50351300	1.09394300	-1.85194700
H	-3.02323500	2.66158700	-3.15673500
H	-1.65824200	2.37637600	-1.99891700
H	-4.85882400	-3.97717200	2.04700400
H	-2.00218100	2.70045900	0.42232500
H	-5.45431000	3.85653400	-0.51799300
C	-2.93343900	0.30302500	-0.66465100
C	-3.47931900	1.26562800	-1.51436700
C	-2.74639800	2.41273000	-2.12417600
C	-3.49051200	3.12448400	-1.05332600
C	-4.76089300	3.83683600	-1.37589700
O	-1.63817600	0.32611500	-0.31214500
Sm	0.42280500	0.02900800	-0.03860400
O	-0.03665000	-1.34458000	-2.04875700
C	-0.77749800	-0.80505700	-3.17191700
C	-1.24135200	-2.01421500	-3.96750100
C	-0.18676500	-3.06348000	-3.62841600
C	0.07077300	-2.77382100	-2.16291200
I	1.32379700	2.38013200	-1.82987400
I	0.12580300	-2.43107400	1.80944700
O	2.55757300	0.57154800	1.36296200
C	3.73095400	1.26133700	0.88762700
C	4.52439800	1.61576900	2.13225200
C	4.17480100	0.46268900	3.06691700
C	2.70370700	0.25565700	2.76147000
O	-0.02454800	1.59443800	1.84840200
C	0.49635800	2.93157300	2.00136100
C	-0.37505900	3.58869000	3.05917100

C	-0.82780700	2.39606100	3.89708300
C	-1.05031500	1.34118400	2.83251600
O	2.55765300	-1.16817400	-0.92667900
C	3.08457900	-1.00306500	-2.25911800
C	4.46870900	-1.62162000	-2.22016000
C	4.27129100	-2.75537100	-1.21939900
C	3.36878400	-2.10356100	-0.18815100
H	4.29157600	0.57731500	0.22791700
H	3.40339200	2.12146600	0.28860500
H	2.06804600	0.94444600	3.34417700
H	2.33852300	-0.76852800	2.91706700
H	4.17247900	2.57113300	2.55454300
H	5.59963700	1.71212600	1.92784000
H	4.35440300	0.68167100	4.12851100
H	4.75359400	-0.43732700	2.80242100
H	1.54737700	2.84943100	2.32443000
H	0.46967300	3.42631800	1.02051700
H	-2.03167900	1.44493000	2.34585600
H	-0.92846000	0.30442000	3.17571300
H	-1.24314000	4.07554500	2.58983100
H	0.17436100	4.34718400	3.63361600
H	-1.73600700	2.59215600	4.48318000
H	-0.03173700	2.08258000	4.59218900
H	2.42353700	-1.53379900	-2.96465600
H	3.06179200	0.06863900	-2.49874600
H	3.95066500	-1.54245400	0.56226000
H	2.69676700	-2.79214900	0.34190500
H	5.20492700	-0.89527100	-1.83888100
H	4.80425900	-1.95742700	-3.21100200
H	5.20639700	-3.12821200	-0.77919400
H	3.75876400	-3.60519000	-1.69908800
H	-1.60169600	-0.19559700	-2.77776700
H	-0.08749600	-0.16082500	-3.73918500
H	1.06779500	-3.06334800	-1.80541900
H	-0.68299800	-3.24194500	-1.50981100
H	-1.31499800	-1.79782100	-5.04193600
H	-2.23060600	-2.34563700	-3.61611200
H	0.72808300	-2.89947200	-4.22145000
H	-0.52416700	-4.09557500	-3.79610300
H	-2.31949900	4.43780900	0.19794500
H	-4.57245900	4.89305000	-1.66048400
H	-3.49196300	3.43241200	1.08397100
H	-5.28670300	3.37062500	-2.22434400
C	-2.79494100	3.43558100	0.22750600
C	-3.78708900	-0.77513300	-0.05441600
C	-4.65636300	-1.81503700	2.09136000
C	-3.79233900	-0.71864300	1.47836200
C	-3.36271600	-2.17386400	-0.52168700
C	-4.21806900	-3.27503200	0.09522000
C	-4.21175500	-3.19448600	1.61780700
H	-4.82180200	-0.60126200	-0.40298400
H	-5.71306600	-1.65340400	1.80636600
H	-4.61815900	-1.75757100	3.19188000
H	-2.75392600	-0.83954200	1.82895900
H	-4.13691000	0.27712100	1.80515400
H	-2.30872000	-2.32768500	-0.23411300
H	-3.41285700	-2.21820200	-1.62283800
H	-3.86111800	-4.26404600	-0.23746300
H	-5.25776600	-3.17874500	-0.27053000
H	-3.18768900	-3.38759600	1.98399200

1f _Int II

E_{vacuum} = -26109.9229

E_{sol} = -26109.9360

H_{sol} = -26109.0942

–*TS* = -0.1443

G_{sol} = -26109.2515

H	-4.30246000	2.29214500	-0.91577200
H	-2.49751600	3.54925700	-2.18263200
H	-1.25113100	2.73686500	-1.22433200
H	-5.64322400	-3.59204900	1.09327500
H	-2.72845500	3.18145500	1.53165600
H	-1.96363800	6.46132700	-0.05133900
C	-2.89159700	0.85696200	-0.34210400
C	-3.23500200	2.07811500	-0.81267000
C	-2.27296100	3.16601000	-1.17014800
C	-2.23071900	4.32550700	-0.21728600
C	-1.71406700	5.62624100	-0.72611700

O	-1.62184100	0.49162200	-0.14862200
Sm	0.40445300	-0.08937700	-0.07532800
O	-0.11006100	-0.38488700	-2.50638500
C	-0.53861100	0.64515300	-3.41087000
C	-1.88909300	0.16931500	-3.89633400
C	-1.66037300	-1.33586000	-4.04252900
C	-0.62527100	-1.65310600	-2.95990300
I	1.78783800	2.55422800	-0.94410200
I	-0.40567900	-2.90218400	0.95321200
O	2.54012700	-0.48572300	1.35574500
C	3.85113200	0.03310500	1.06031900
C	4.63531300	-0.10690600	2.35058500
C	4.04198000	-1.38519400	2.93365600
C	2.57138800	-1.23654200	2.58742400
O	0.21350600	0.93412400	2.17559000
C	1.03575700	1.95328500	2.78374500
C	0.24484700	2.45543400	3.98072600
C	-0.59713500	1.23835000	4.35160400
C	-0.95578300	0.68929100	2.98650100
O	2.22355400	-1.37580500	-1.37350600
C	2.84848300	-0.86691500	-2.56922700
C	4.12371600	-1.67168200	-2.71824500
C	3.68320900	-3.03571700	-2.19730800
C	2.79168500	-2.65602900	-1.02751400
H	4.29515900	-0.57380700	0.25222000
H	3.73312200	1.06269300	0.69673100
H	2.02493400	-0.66237300	3.35459500
H	2.03865000	-2.18242600	2.42095000
H	4.43789500	0.74955600	3.01567600
H	5.71907600	-0.15952400	2.17758700
H	4.20608600	-1.49841800	4.01408900
H	4.46835900	-2.26870600	2.43121600
H	1.98610600	1.48407200	3.08593900
H	1.24897700	2.71871600	2.02504700
H	-1.80748300	1.21925500	2.53497000
H	-1.15198200	-0.39121700	2.96453200
H	-0.40683100	3.29159300	3.68485100
H	0.89992100	2.80218400	4.79180800
H	-1.48673500	1.48526400	4.94692000
H	0.00223200	0.50568600	4.91641900
H	2.16676000	-1.02998300	-3.42066400
H	2.99427000	0.21422400	-2.43783700
H	3.36706700	-2.53398600	-0.09495500
H	1.96858400	-3.35538700	-0.83069600
H	4.92129100	-1.25233200	-2.08343600
H	4.48783000	-1.68979300	-3.75471600
H	4.51520400	-3.68525300	-1.89226900
H	3.10191900	-3.56758900	-2.96764900
H	-0.54989400	1.59011100	-2.85619000
H	0.19770500	0.72437600	-4.23179300
H	0.21375100	-2.25962600	-3.33926500
H	-1.05247800	-2.16583800	-2.08610500
H	-2.19356800	0.65073000	-4.83593600
H	-2.64581200	0.38850900	-3.12785100
H	-1.25661600	-1.56839500	-5.03978500
H	-2.57955400	-1.92220500	-3.90825300
H	-1.04339300	3.74473100	1.47319000
H	-0.60688400	5.60917900	-0.82023400
H	-2.34119800	4.90035100	1.86133800
H	-2.11040600	5.85987700	-1.72769200
C	-2.09101000	4.02909500	1.23442400
C	-3.94390600	-0.17974700	-0.01784500
C	-4.97938400	-1.63911400	1.77329600
C	-3.90772600	-0.60292800	1.45372800
C	-3.82773100	-1.41404600	-0.91881800
C	-4.89213300	-2.45782400	-0.59859900
C	-4.84852300	-2.86109100	0.87096000
H	-4.92365400	0.29214300	-0.21329600
H	-5.98034400	-1.18841900	1.63665800
H	-4.91302100	-1.93635100	2.83282400
H	-2.91607800	-1.03471900	1.66579400
H	-4.02681000	0.28647100	2.09517900
H	-2.82918000	-1.85825700	-0.77076400
H	-3.90261900	-1.10598400	-1.97466500
H	-4.75887900	-3.34239100	-1.24316200
H	-5.89137900	-2.04613800	-0.83474400
H	-3.88535700	-3.36024500	1.07876000

1f _PreTS II

$\boldsymbol{E}_{vacuum}$ = -26418.1850

$\boldsymbol{E}_{sol}$ = -26418.1996

$\boldsymbol{H}_{sol}$ = -26417.2350

–TS = -0.1636

$\boldsymbol{G}_{sol}$ = -26417.4131

H	1.22068200	4.78772700	-0.51852600
H	-0.89209200	2.71933300	0.43420900
H	-1.30279300	4.15666000	-0.53274800
H	6.57072000	1.76226100	-2.34964300
H	-0.41803100	2.95620000	2.86208100
H	-1.36295500	6.49153000	2.30214100
C	1.61018400	2.73557900	-0.42378100
C	0.80015500	3.80793800	-0.27419400
C	-0.61181800	3.76700000	0.23947700
C	-0.77435000	4.57707700	1.49470800
C	-0.96812600	6.05018900	1.37251700
O	1.22322600	1.48686900	-0.16092200
Sm	0.50980400	-0.49136200	0.03910300
O	2.26100400	-0.46115300	1.79277800
C	2.30810100	0.59593100	2.78154600
C	3.71932700	0.56003300	3.34435700
C	4.11645300	-0.90039700	3.14759800
C	3.49128600	-1.20732600	1.80141200
I	-1.52967300	0.10194300	2.29642300
I	2.50269200	-1.69996400	-1.97808000
O	-1.21355200	-2.19418000	-0.84423500
C	-2.33727800	-2.73158800	-0.11785100
C	-3.24841000	-3.33479800	-1.17109900
C	-2.25067600	-3.78271900	-2.23386100
C	-1.24661500	-2.64621800	-2.21416800
O	-0.93583200	0.42527400	-1.75045900
C	-2.35645900	0.64851800	-1.59792200
C	-2.69434300	1.78273500	-2.54969800
C	-1.62783600	1.63255000	-3.63044800
C	-0.41251300	1.26101900	-2.80635000
O	0.74655800	-2.80287500	1.21462700
C	0.64091600	-3.01404000	2.63681900
C	0.35083500	-4.49279000	2.79553400
C	1.16034100	-5.08492300	1.64681900
C	0.92876400	-4.06739700	0.54367100
H	-1.96058900	-3.49316800	0.58680400
H	-2.78870500	-1.91808200	0.46512800
H	-1.56607300	-1.80321400	-2.84894700
H	-0.22407600	-2.92779100	-2.50046000
H	-3.92648500	-2.56589800	-1.57472300
H	-3.86410400	-4.15168700	-0.76932000
H	-2.69739800	-3.92577400	-3.22735600
H	-1.76877400	-4.72879300	-1.93759400
H	-2.87936000	-0.28402300	-1.86665600
H	-2.56089800	0.88037500	-0.54448100
H	0.05947600	2.14648300	-2.35325300
H	0.34956400	0.67593500	-3.33949500
H	-2.59008500	2.74913200	-2.03385600
H	-3.72251600	1.70522000	-2.92689600
H	-1.46639400	2.54772500	-4.21648100
H	-1.88960700	0.81885600	-4.32672500
H	1.60131900	-2.74257000	3.10790800
H	-0.14164200	-2.34388000	3.01682800
H	0.01179400	-4.28777200	-0.02651800
H	1.75971700	-3.96001900	-0.16673200
H	-0.72450900	-4.68971000	2.65415900
H	0.64029100	-4.87303400	3.78491800
H	0.84387000	-6.09543800	1.35348700
H	2.22834900	-5.12810100	1.91522500
H	2.04478300	1.53773100	2.28111000
H	1.54175100	0.37168400	3.53962600
H	3.24091400	-2.26339400	1.63591600
H	4.12165500	-0.86730500	0.96360200
H	3.75292400	0.88601800	4.39278300
H	4.38373700	1.21389400	2.75933400
H	3.67082200	-1.53227700	3.93337500
H	5.20287500	-1.06385500	3.14950000
H	0.91628300	4.12308600	2.75044000
H	-0.01129600	6.57503800	1.16080300
H	-0.55826800	4.56202900	3.64219900
H	-1.65239700	6.30672600	0.54664300
C	-0.19070800	4.02867200	2.75106500

C	3.01995200	2.88098200	-0.95134600
C	4.56663400	2.39166500	-2.90327500
C	3.13938800	2.31896100	-2.37308800
C	4.03190800	2.20890600	-0.02062700
C	5.45537700	2.27838600	-0.55979200
C	5.54074400	1.68987700	-1.96353300
H	3.24748100	3.96138800	-0.98803100
H	4.86772000	3.45050800	-3.01406200
H	4.61610500	1.94461300	-3.90939100
H	2.81107500	1.26618200	-2.36481400
H	2.45317600	2.86900000	-3.03844200
H	3.73546800	1.15347800	0.09585500
H	3.97137900	2.67454700	0.97785700
H	6.14431600	1.75177700	0.12227100
H	5.78880900	3.33257700	-0.58503100
H	5.28787400	0.61491000	-1.92334400
C	-6.13680600	1.42085400	-1.18183800
C	-5.34227000	1.26184100	-0.03514300
C	-5.25459300	-0.00400500	0.56909000
C	-5.94526500	-1.08364000	0.02854600
C	-6.72619900	-0.92137000	-1.11649100
C	-6.82029200	0.33385800	-1.71771000
C	-4.59215400	2.35255700	0.49880100
C	-3.89500500	3.23462600	0.95716000
H	-3.22839000	3.97328400	1.36951000
H	-4.62658200	-0.12304100	1.45366200
H	-5.87385800	-2.06231600	0.50793800
H	-7.26676800	-1.77168900	-1.53708900
H	-7.43440800	0.46817900	-2.61036900
H	-6.20682000	2.40552600	-1.64661600

1f _TS II

***E*$_{vacuum}$** = -26418.1753

***E*$_{sol}$** = -26418.1935

***H*$_{sol}$** = -26417.2267

–TS = -0.1609

***G*$_{sol}$** = -26417.4059

H	-1.06399700	4.56354600	-0.55156500
H	-2.01656000	1.77372700	0.44069300
H	-3.04905500	2.88390100	-0.49410000
H	5.16315200	4.34959100	-2.10167400
H	-1.59941900	2.19024400	2.83712900
H	-3.90748200	5.07838500	2.33180700
C	0.22533500	2.92695900	-0.40480200
C	-0.99257100	3.50368300	-0.29038200
C	-2.22719600	2.83586800	0.24419300
C	-2.68221700	3.49660100	1.51368600
C	-3.38318900	4.80952900	1.39957800
O	0.45500500	1.64431100	-0.12202800
Sm	0.69517400	-0.44864800	0.01987600
O	2.25477600	0.30458400	1.79281700
C	1.83510800	1.24614700	2.80940900
C	3.11993300	1.82626700	3.37622100
C	4.11907100	0.69446300	3.15220100
C	3.68776800	0.17025400	1.79694300
I	-1.43256300	-0.82514500	2.24224600
I	3.05184500	-0.65820000	-1.95939800
O	-0.16327900	-2.70004300	-0.88914000
C	-0.96038300	-3.67289900	-0.18410500
C	-1.54842000	-4.56545600	-1.26049700
C	-0.44404800	-4.55508100	-2.31202800
C	0.01889400	-3.11123600	-2.26175100
O	-0.89418900	-0.20166400	-1.86571100
C	-2.30307500	-0.48981500	-1.71497000
C	-3.01460400	0.40687700	-2.71286600
C	-1.94747500	0.61951800	-3.78200500
C	-0.69939400	0.74916900	-2.93496300
O	1.87351800	-2.45387200	1.18019800
C	1.87095800	-2.69684200	2.60082000
C	2.20995500	-4.16601600	2.74852400
C	3.19258100	-4.36590400	1.59961800
C	2.56952900	-3.52081000	0.50194500
H	-0.29970600	-4.23605800	0.49797900
H	-1.69920100	-3.13407000	0.42347400
H	-0.59742500	-2.45785200	-2.90001400
H	1.07429900	-2.95619300	-2.52516800
H	-2.47064600	-4.11866100	-1.66691500
H	-1.79314200	-5.56800000	-0.88342000

H	-0.78359500	-4.84784600	-3.31504400
H	0.37234800	-5.23452600	-2.01732200
H	-2.45504500	-1.55916100	-1.93713000
H	-2.59353700	-0.30674400	-0.67215900
H	-0.61256200	1.75541600	-2.49761000
H	0.24069000	0.48534300	-3.43821800
H	-3.27635600	1.36424100	-2.23820500
H	-3.94132900	-0.04985900	-3.08265900
H	-2.12162200	1.50922500	-4.40261800
H	-1.87762500	-0.25707900	-4.44715600
H	2.63991200	-2.06029000	3.07172100
H	0.88423400	-2.40776800	2.98658700
H	1.83065500	-4.09284700	-0.08222300
H	3.28899300	-3.07080800	-0.19584800
H	1.30858300	-4.78304800	2.60083100
H	2.62836700	-4.40303500	3.73644300
H	3.31619000	-5.41488300	1.29682800
H	4.18489300	-3.97282000	1.87391200
H	1.17707200	1.98506900	2.33261600
H	1.25424400	0.68554900	3.55831200
H	3.92330400	-0.88602400	1.61100700
H	4.10395900	0.76621100	0.96839300
H	3.01231000	2.11376500	4.43091800
H	3.42369200	2.71736000	2.80596400
H	4.00101200	-0.08436700	3.92341000
H	5.16639400	1.02628200	3.15610100
H	-0.91405600	3.82761600	2.69013300
H	-2.66589100	5.63201200	1.19813200
H	-2.38493200	3.57927500	3.65267000
H	-4.11727200	4.80785400	0.57859700
C	-1.86303200	3.25446100	2.73691600
C	1.42845300	3.70641700	-0.88943400
C	3.12533600	3.98175400	-2.75774700
C	1.86644600	3.26080700	-2.28934700
C	2.58953400	3.58135400	0.10104100
C	3.84550100	4.29953900	-0.37649400
C	4.26414700	3.81176400	-1.75867500
H	1.13201400	4.76920700	-0.94299900
H	2.91045900	5.05980800	-2.88270400
H	3.42574700	3.60484300	-3.74877900
H	2.05838100	2.17484100	-2.27200600
H	1.04118800	3.43394600	-2.99993000
H	2.80791600	2.50830500	0.23009000
H	2.27074000	3.96631100	1.08484800
H	4.66369500	4.15388100	0.34918000
H	3.65552100	5.38827800	-0.41575300
H	4.53358900	2.74147000	-1.70129900
C	-6.16508400	0.80120800	-0.86717900
C	-5.38653300	0.41156900	0.24047400
C	-4.99187600	-0.93738400	0.34976200
C	-5.36512100	-1.85821000	-0.62225600
C	-6.13876200	-1.46334300	-1.71560300
C	-6.53646900	-0.12979600	-1.83014300
C	-5.02866900	1.34750700	1.24431600
C	-4.66600700	2.16621700	2.08135200
H	-4.63787000	2.74368100	2.98711600
H	-4.38061700	-1.23967400	1.20135000
H	-5.05332400	-2.90051100	-0.52050400
H	-6.43632300	-2.19213900	-2.47216200
H	-7.14446900	0.18784300	-2.67993200
H	-6.47639200	1.84329300	-0.95395500

1f_Int III

$\boldsymbol{E}_{\mathbf{vacuum}}$ = -26418.2366

$\boldsymbol{E}_{\mathbf{sol}}$ = -26418.2503

$\boldsymbol{H}_{\mathbf{sol}}$ = -26417.2847

–*TS* = -0.1576

$\boldsymbol{G}_{\mathbf{sol}}$ = -26417.4561

H	-1.35215700	4.48406600	-0.42723600
H	-2.16390700	1.58389900	0.33306600
H	-3.20416800	2.70049100	-0.57097700
H	4.82264500	4.70153900	-2.07803600
H	-1.81256600	1.91039700	2.81604200
H	-4.08690400	4.88691100	2.32999000
C	0.02074700	2.91974400	-0.33451500
C	-1.22728600	3.42278600	-0.19613400
C	-2.43096000	2.64551800	0.21919000
C	-3.09035900	3.11757100	1.52961000

C	-3.53987900	4.57897900	1.42458200	H	-2.43734700	1.37126400	-4.23763300
O	0.32184600	1.64076500	-0.10980500	H	-1.97084600	-0.34109200	-4.41166800
Sm	0.72758800	-0.42849100	0.00158600	H	2.72925400	-1.92440800	3.06298800
O	2.19533000	0.41915200	1.81018200	H	1.04182200	-2.50041700	2.90743900
C	1.68436200	1.30758300	2.83325500	H	2.26421600	-3.95139200	-0.16533900
C	2.91013500	1.96129700	3.44921000	H	3.58352500	-2.75259300	-0.23058400
C	3.99393500	0.91078500	3.22341200	H	1.79130000	-4.78481200	2.47499600
C	3.63347800	0.39091100	1.84594800	H	3.02223800	-4.26630400	3.65405200
I	-1.36792600	-1.05279800	2.20022100	H	3.89247000	-5.10512700	1.20702200
I	3.08695200	-0.38039500	-1.98006400	H	4.54802500	-3.57748600	1.84304900
O	0.12064000	-2.73875700	-0.96784300	H	0.99120700	2.01106400	2.35283200
C	-0.55378700	-3.81089000	-0.28063000	H	1.12181800	0.69245000	3.55274600
C	-1.03418600	-4.74768600	-1.37322200	H	3.95139800	-0.64010200	1.64244600
C	0.05458700	-4.58165500	-2.42857700	H	4.02283900	1.03696500	1.04230700
C	0.33923200	-3.09370700	-2.34956000	H	2.75571500	2.21101200	4.50768700
O	-0.93922100	-0.31080800	-1.82890300	H	3.16369400	2.88728400	2.91097000
C	-2.28690100	-0.82119700	-1.70727500	H	3.91524000	0.10598700	3.97265800
C	-3.13529000	0.04955600	-2.61444900	H	5.01387400	1.31735400	3.26144100
C	-2.13385400	0.47169600	-3.68474600	H	-1.22012000	3.57049000	2.52515600
C	-0.88810200	0.70667300	-2.85502800	H	-2.67264300	5.24785600	1.31569700
O	2.08264100	-2.34960000	1.13454900	H	-2.58289300	3.29320400	3.64582000
C	2.06908300	-2.64211200	2.54626900	H	-4.20536000	4.72782000	0.55954500
C	2.59806900	-4.05741000	2.66225700	C	-2.11999700	2.96066700	2.70269900
C	3.62326700	-4.09130000	1.53411200	C	1.17050000	3.78404200	-0.80141500
C	2.91597800	-3.30482600	0.44468500	C	2.79353400	4.23675100	-2.69878800
H	0.17051400	-4.30420800	0.39065100	C	1.59428600	3.42105100	-2.22973800
H	-1.34825400	-3.37221200	0.33733800	C	2.36194100	3.68575600	0.15399300
H	-0.35757300	-2.50706900	-2.97038700	C	3.55981000	4.49721100	-0.32390100
H	1.36546700	-2.80296800	-2.61259400	C	3.96775000	4.09510200	-1.73667800
H	-2.00649600	-4.41067800	-1.76791800	H	0.81457300	4.82984800	-0.80398300
H	-1.15178300	-5.77994000	-1.01579000	H	2.51040200	5.30376900	-2.77183900
H	-0.25360900	-4.89349300	-3.43594500	H	3.08880900	3.92051100	-3.71234000
H	0.94975100	-5.16209200	-2.15157800	H	1.85297600	2.34934800	-2.26050900
H	-2.28282700	-1.87495400	-2.03425200	H	0.73898100	3.56933500	-2.90985800
H	-2.58298300	-0.78187300	-0.65105600	H	2.64680000	2.62360700	0.23273700
H	-0.90422300	1.69406000	-2.36885500	H	2.04844200	4.01218700	1.16034100
H	0.06133100	0.58159000	-3.39374500	H	4.40536600	4.37002800	0.37324200
H	-3.49764000	0.92742500	-2.05998200	H	3.30497300	5.57329400	-0.30988600
H	-4.01142700	-0.48870300	-2.99914000	H	4.30267800	3.04201900	-1.73309900

C	-6.27408400	0.68302600	-0.80380300
C	-5.36060700	0.36387200	0.24912500
C	-5.00544500	-1.00978700	0.42842100
C	-5.52312700	-1.98304700	-0.40729800
C	-6.40862000	-1.64928400	-1.44178000
C	-6.77830900	-0.30933000	-1.62476700
C	-4.83527100	1.34657000	1.05587600
C	-4.33334900	2.28300700	1.81212500
H	-4.84872800	2.53276800	2.75509200
H	-4.30856500	-1.26754600	1.22735200
H	-5.23696600	-3.02616000	-0.25203900
H	-6.81434300	-2.42555300	-2.09285900
H	-7.47356100	-0.04235100	-2.42390000
H	-6.56087300	1.72579500	-0.94850300

1f _TS III

$\boldsymbol{E}_{\mathbf{vacuum}}$ = -26418.2267

$\boldsymbol{E}_{\mathbf{sol}}$ = -26418.2422

$\boldsymbol{H}_{\mathbf{sol}}$ = -26417.2760

–*TS* = -0.1561

$\boldsymbol{G}_{\mathbf{sol}}$ = -26417.4477

C	-2.11303500	2.79875800	0.72230300
C	-2.75799900	1.54782600	1.22797100
C	-4.18213500	1.65776100	-0.62352600
C	-4.07027300	2.96117700	-0.76065300
C	-3.10066800	3.77709400	0.06668800
C	-2.14408800	0.32084900	1.22992300
H	-6.05719600	0.30987000	0.73087500
H	-1.59563800	3.33501800	1.54099400
C	-3.89794200	4.54566400	1.12827300
C	-2.34194200	4.76762700	-0.81598200
H	-3.02922400	5.48652900	-1.29071200
H	-1.61787800	5.34421800	-0.21655500
H	-4.73150500	3.51661300	-1.44630500
H	-1.34542600	2.51892300	-0.01468400
O	-0.97850200	0.09979500	0.63899100
Sm	1.02305300	-0.04799900	-0.03281700
O	-0.02422200	-1.72173300	-1.49086100
C	-1.20537700	-1.38281100	-2.26251900
C	-1.82338000	-2.71311600	-2.65794300
C	-0.62099400	-3.65224100	-2.66947600
C	0.17725600	-3.14747100	-1.48466000
I	0.77040400	2.10505300	-2.22294300
I	1.80469300	-2.32085300	1.90997800
O	3.43264800	0.85702300	0.32766200
C	4.20280900	1.60698600	-0.63283100
C	5.38317800	2.16427000	0.14259800
C	5.60259400	1.08784300	1.20073300
C	4.17721500	0.70734000	1.55224700
O	1.15770000	1.66174900	1.75103500
C	1.51432200	3.05145300	1.59824600
C	1.08451400	3.73299700	2.89068900
C	1.06845500	2.58083400	3.89235800
C	0.54446100	1.44468900	3.04002000
O	2.75434200	-1.15920400	-1.60031700
C	2.63532400	-1.16545500	-3.03899300
C	3.98265400	-1.63887700	-3.54822600
C	4.40128300	-2.61110900	-2.45060900
C	3.93472200	-1.88660200	-1.20192500
H	4.52593100	0.91755300	-1.43137600
H	3.54568300	2.36340800	-1.08247800
H	3.74886000	1.38823300	2.30784500
H	4.04493100	-0.32549600	1.90232700
H	5.11413200	3.12073800	0.61973100
H	6.25734600	2.34108600	-0.49900500
H	6.17032900	1.43495600	2.07495600
H	6.13633800	0.22552500	0.76897600
H	2.60414600	3.09838600	1.44490400
H	1.01730600	3.44365100	0.69959000
H	-0.55067800	1.48209600	2.92365600
H	0.83758700	0.44023000	3.37504600
H	0.07329900	4.15419700	2.78289500
H	1.76322300	4.55006600	3.17151000
H	0.43319800	2.77364300	4.76758400
H	2.08706600	2.35658500	4.24878900
H	1.82892800	-1.86348400	-3.31899400
H	2.34957700	-0.15377000	-3.35709400
H	4.68758600	-1.16108000	-0.85124500

H	3.66335900	-2.53968800	-0.36156500
H	4.69033300	-0.79588200	-3.60691800
H	3.91473900	-2.09635300	-4.54480400
H	5.47921900	-2.82290500	-2.43125700
H	3.86813300	-3.56924400	-2.56280000
H	-1.85300800	-0.75998600	-1.63289800
H	-0.87077900	-0.79104100	-3.12893300
H	1.25836900	-3.33135400	-1.54348200
H	-0.19112900	-3.55351500	-0.52889700
H	-2.33839900	-2.65387500	-3.62564300
H	-2.55783200	-3.03144700	-1.90329100
H	-0.04435800	-3.53486800	-3.60185200
H	-0.89218800	-4.71181400	-2.56558800
H	-1.78458000	4.24264900	-1.60617500
H	-7.08302700	-1.84719000	0.07440400
H	-4.66025000	5.18878800	0.66025900
H	-4.41788200	3.85951200	1.81414300
H	-3.23153200	5.19223600	1.72332000
H	-3.64424600	1.63779800	1.86205400
C	-4.84009800	0.48417400	-1.05024700
C	-4.50089500	-0.14514000	-2.27254500
C	-5.08520000	-1.35178400	-2.63588300
C	-6.00875300	-1.97980400	-1.79614500
C	-6.35695700	-1.36916600	-0.58715000
C	-5.78718800	-0.16018400	-0.21654200
H	-3.77833900	0.34439800	-2.92844700
H	-4.82162000	-1.81047900	-3.59193100
H	-6.45764900	-2.93236700	-2.08282300
H	-3.19836600	-4.59188500	3.23019600
C	-2.79180000	-0.88607000	1.86077200
C	-2.67059400	-2.55477100	3.76580600
C	-2.05505100	-1.30898300	3.13850300
C	-2.86316800	-2.05709900	0.87596000
C	-3.48113700	-3.29704000	1.50794600
C	-2.72911700	-3.70730600	2.76894100
H	-3.82391200	-0.60180300	2.13248700
H	-3.69539000	-2.32354700	4.11236300
H	-2.09644100	-2.84911100	4.65943000
H	-1.00006200	-1.51011600	2.88443400
H	-2.06144100	-0.47396800	3.85932000
H	-3.49555000	-4.12472700	0.77877000
H	-4.53798800	-3.08940300	1.75834800
H	-1.69769300	-3.99844300	2.49806000
H	-1.83478300	-2.28485700	0.54867900
H	-3.43477400	-1.75477200	-0.01522800

1f _Int IV

E$_{vacuum}$ = -26418.2749

E$_{sol}$ = -26418.2892

H$_{sol}$ = -26417.3213

–TS = -0.1545

G$_{sol}$ = -26417.4900

C	-2.66557500	2.27871800	1.56177200
C	-3.26079100	0.92920400	1.10342700
C	-3.69799000	1.26967900	-0.32233000
C	-3.72633500	2.59652900	-0.51268400
C	-3.33470400	3.37991400	0.71603600
C	-2.32390400	-0.23498900	1.23137000
C	-4.59331300	3.92402600	1.40369400
H	-2.87964600	5.28607700	-0.24018300
H	-2.04412500	5.03431000	1.30724200
H	-1.50412700	4.15955300	-0.16233400
C	-2.38342600	4.52818500	0.38782400
H	-5.51647300	-2.84829900	-1.47511200
H	-4.06427100	3.08777600	-1.42897900
H	-1.59405200	2.24835000	1.31509600
O	-1.09142700	-0.10225600	0.73863900
Sm	0.85280300	0.01254400	-0.04393600
O	0.00213500	-1.88881500	-1.36840600
C	-1.28277800	-1.80367600	-2.03711700
C	-1.64828200	-3.23312200	-2.39447300
C	-0.28522100	-3.90586200	-2.52124200
C	0.49158500	-3.24078300	-1.40233300
I	0.06701400	2.08585600	-2.18153100
I	2.09697800	-2.06821700	1.88127900
O	3.15179800	1.24407600	0.06179700
C	3.69395300	2.09157800	-0.96865400
C	4.85012200	2.82611000	-0.31587400

C	5.35719800	1.78734500	0.67939200
C	4.06138500	1.17347400	1.17721900
O	0.99809000	1.71781500	1.77088800
C	1.17564600	3.13720900	1.59275100
C	0.63801400	3.77368800	2.86244700
C	0.90678100	2.69030700	3.90300300
C	0.58548300	1.42967800	3.12491400
O	2.58396600	-0.81781400	-1.79995700
C	2.33675900	-0.86053600	-3.22025000
C	3.66500000	-1.24148300	-3.84779600
C	4.27580000	-2.13018300	-2.76899900
C	3.86810800	-1.39802900	-1.50443100
H	4.03470400	1.45383300	-1.80257100
H	2.88477400	2.73383800	-1.34114700
H	3.63063100	1.74934900	2.01395900
H	4.13703100	0.12292100	1.48941300
H	4.48765000	3.72182400	0.21437600
H	5.60616000	3.14749800	-1.04546900
H	5.95846400	2.21119000	1.49546100
H	5.97044000	1.02996100	0.16440900
H	2.25270800	3.33482200	1.46181500
H	0.64919300	3.43783800	0.67587800
H	-0.49254500	1.21072800	3.11700000
H	1.12997900	0.53323000	3.45188400
H	-0.44351600	3.95254600	2.76983400
H	1.12756800	4.73191400	3.08449400
H	0.28965900	2.78821400	4.80654500
H	1.96585100	2.69803800	4.20840300
H	1.56143600	-1.62003500	-3.41351300
H	1.94584800	0.12024700	-3.52393700
H	4.57552100	-0.58501300	-1.26723000
H	3.75595300	-2.03336200	-0.61468600
H	4.28847800	-0.34628500	-4.00499700
H	3.53987500	-1.74279700	-4.81738900
H	5.36523300	-2.24580800	-2.85259600
H	3.82355600	-3.13502500	-2.79646900
H	-1.99059700	-1.32068200	-1.35390200
H	-1.15373300	-1.16373100	-2.92358300
H	1.57765900	-3.20187700	-1.55880500
H	0.30085800	-3.71207800	-0.42471800
H	-2.25514600	-3.28096100	-3.30770100
H	-2.22759900	-3.69310200	-1.57933100
H	0.17137200	-3.67664900	-3.49844000
H	-0.32128400	-4.99831900	-2.40884700
H	-5.15024600	4.59895900	0.73424000
H	-4.85633500	-1.12726100	0.17367600
H	-5.27146500	3.10670700	1.69448800
H	-2.77255700	2.44465700	2.64549200
H	-4.32896600	4.49201400	2.31115900
H	-4.16275000	0.68746100	1.69632100
C	-4.08408500	0.25019100	-1.30119500
C	-3.85884000	0.44513500	-2.67259100
C	-4.23229800	-0.52243700	-3.60055200
C	-4.83072800	-1.71040000	-3.17819800
C	-5.04991100	-1.92192000	-1.81738200
C	-4.67525100	-0.95449000	-0.88823800
H	-3.34825800	1.35253500	-3.00023000
H	-4.04352100	-0.35260000	-4.66295200
H	-5.12159600	-2.47026500	-3.90677400
H	-1.94139400	-4.74572000	4.20934300
C	-2.60125200	-1.42740900	2.09884300
C	-2.10156100	-2.58234900	4.31512000
C	-1.85238100	-1.35712600	3.44258500
C	-2.25908900	-2.74216600	1.38543500
C	-2.50021500	-3.96604000	2.26095800
C	-1.73802300	-3.86576100	3.57710300
H	-3.68607100	-1.43075100	2.32064700
H	-3.16902400	-2.61765900	4.60409600
H	-1.52685700	-2.50271100	5.25271000
H	-0.77343600	-1.28061400	3.22480700
H	-2.14464300	-0.43716400	3.97742200
H	-1.19557600	-2.70242300	1.09484400
H	-2.84537000	-2.80957300	0.45458300
H	-2.21111500	-4.88205000	1.71853100
H	-3.58218000	-4.05886500	2.47289700
H	-0.65330300	-3.86465500	3.36724700

1f _Product

$\boldsymbol{E}_{\mathbf{vacuum}}$ = -26418.2891

$\boldsymbol{E}_{\mathbf{sol}}$ = -26418.3094

$\boldsymbol{H}_{\mathbf{sol}}$ = -26417.3340

–TS = -0.1587

$\boldsymbol{G}_{\mathbf{sol}}$ = -26417.5130

C	-2.88428600	2.29846600	1.49004100
C	-3.48240500	0.94578400	1.05239900
C	-3.81424600	1.20895800	-0.41225800
C	-3.80690100	2.52537800	-0.66270900
C	-3.47935900	3.36439500	0.54385800
C	-2.48278000	-0.18425100	1.20063500
C	-4.76196900	3.97296300	1.12347900
H	-2.92435400	5.22182000	-0.44867900
H	-2.14211600	4.98129100	1.12880800
H	-1.59706500	4.04618300	-0.30425800
C	-2.47393700	4.46496200	0.21293700
H	-5.42975800	-3.00411300	-1.55012100
H	-4.05655500	2.96859800	-1.62922800
H	-1.79850100	2.24512800	1.32258700
O	-1.39036700	-0.08443100	0.65745700
Sm	0.96218900	0.04030700	-0.10999500
O	0.01860800	-2.05734100	-1.30451300
C	-1.15055900	-2.05098600	-2.14472200
C	-1.46551300	-3.51225500	-2.42109200
C	-0.08957600	-4.16440500	-2.32473100
C	0.53523500	-3.38827300	-1.17974900
I	0.07794700	2.06433500	-2.43293200
I	1.96226200	-1.96137900	2.22310700
O	3.22772900	1.33082600	0.13264100
C	3.83461300	2.07629100	-0.93509700
C	5.00996600	2.79970000	-0.30086500
C	5.43266600	1.81280300	0.78425200
C	4.08912700	1.30813000	1.28063100
O	0.73236500	1.90468800	1.70810500
C	1.02826000	3.28501300	1.44129000
C	0.66736400	4.02565600	2.71594500
C	1.00230400	2.98465500	3.78069400
C	0.52922200	1.70307900	3.11718100
O	2.77383000	-0.93526400	-1.71618200
C	2.58296000	-1.08196500	-3.13291400
C	3.92070200	-1.55996700	-3.67029200
C	4.44824000	-2.38324300	-2.49809200
C	4.01080600	-1.54055200	-1.31290900
H	4.16439700	1.36625800	-1.71341000
H	3.06790800	2.72949700	-1.37587800
H	3.66861200	1.97980800	2.05116200
H	4.09501900	0.28569800	1.68498100
H	4.67929700	3.74844900	0.15281800
H	5.80333500	3.02886900	-1.02573100
H	6.03455400	2.26449100	1.58494400
H	6.01557200	0.98646600	0.34549400
H	2.10350800	3.37523000	1.21140000
H	0.46074400	3.58986900	0.55120800
H	-0.54765000	1.53596400	3.29310500
H	1.07927300	0.79845500	3.41503100
H	-0.40963900	4.25735000	2.73292200
H	1.22221500	4.96771800	2.82596600
H	0.50925600	3.16379800	4.74618300
H	2.09008700	2.94902700	3.95496700
H	1.78767300	-1.82827500	-3.30158500
H	2.23991500	-0.11645200	-3.53190100
H	4.74307100	-0.74153100	-1.09859400
H	3.83057400	-2.10364900	-0.38511500
H	4.58579000	-0.70306100	-3.86601000
H	3.81916400	-2.13153500	-4.60321300
H	5.53479500	-2.54503700	-2.52210500
H	3.95708400	-3.36979300	-2.47059900
H	-1.95504900	-1.51933200	-1.62163400
H	-0.91157200	-1.49069100	-3.06351100
H	1.63185900	-3.33035400	-1.21292600
H	0.24897400	-3.79651000	-0.19579500
H	-1.95824500	-3.65327300	-3.39232600
H	-2.13337800	-3.91385200	-1.64242700
H	0.47844600	-4.00331400	-3.25602400
H	-0.12569700	-5.24522900	-2.12947000
H	-5.25868000	4.62229300	0.38562000
H	-4.98034100	-1.20250300	0.07822900

H	-5.47898600	3.19011800	1.41666300
H	-3.06785500	2.51990300	2.55251500
H	-4.53570500	4.58474100	2.01207700
H	-4.39496300	0.69617600	1.62064000
C	-4.09865800	0.13850200	-1.37330100
C	-3.75077100	0.28971900	-2.72508700
C	-4.01913100	-0.72096300	-3.64193200
C	-4.62418100	-1.90867400	-3.22831300
C	-4.95864600	-2.07793500	-1.88585300
C	-4.69722900	-1.06375100	-0.96739400
H	-3.21703700	1.18862300	-3.03880000
H	-3.73221300	-0.58771300	-4.68708200
H	-4.82649000	-2.70375900	-3.94881600
H	-2.06953700	-4.48782600	4.39377500
C	-2.79757400	-1.38640100	2.04838400
C	-2.41091300	-2.34691900	4.35430100
C	-2.17409800	-1.15077300	3.43968500
C	-2.25598100	-2.67777900	1.43464100
C	-2.48656300	-3.86912300	2.35536500
C	-1.87311000	-3.63016600	3.73063500
H	-3.89296300	-1.45839200	2.17175500
H	-3.49415200	-2.45243700	4.55057700
H	-1.93055000	-2.16264100	5.32850200
H	-1.08889700	-1.00523900	3.30211300
H	-2.58137000	-0.22930100	3.88958900
H	-1.17589100	-2.54753200	1.25985100
H	-2.72934200	-2.84301500	0.45323500
H	-2.05953800	-4.77677300	1.89910800
H	-3.57243000	-4.05326900	2.46063100
H	-0.77723500	-3.54541100	3.62490900

3x _Reactant

***E*vacuum** = -25992.0371

***E*sol** = -25992.0621

***H*sol** = -25991.2976

–*TS* = -0.1357

***G*sol** = -25991.4583

C	-2.92770000	-0.88500400	-0.71068000
C	-4.97734600	-2.32656700	-0.78488900
C	-4.98979100	-0.29319700	0.73246800
C	-5.81984900	-1.25224700	-0.10547100
C	-3.51343200	-0.10495500	0.38533800
C	-3.83006200	-1.71497800	-1.58835400
O	-1.70650600	-0.94825600	-0.85704100
H	-6.39156000	-0.70012800	-0.87004100
H	-3.20457100	-2.47345800	-2.07982200
H	-4.55655900	-3.00051000	-0.01821800
H	-5.60258900	-2.95101600	-1.44189300
H	-6.56902300	-1.73003300	0.54294500
H	-5.18164000	-0.32138400	1.80825400
H	-4.23094900	-1.07172900	-2.39150300
H	-2.80009700	0.06644800	1.19966400
C	-4.53372000	1.03038000	0.20596000
C	-4.94956200	1.51676000	-1.15894700
C	-4.38612500	2.14689700	1.21331000
H	-5.04808300	0.72090600	-1.90660600
H	-5.92357500	2.02871300	-1.09213900
H	-4.21933200	2.24502000	-1.54336900
H	-5.35627200	2.64614300	1.37019000
H	-4.02002500	1.77666400	2.18122900
H	-3.66960400	2.90390200	0.85980300
Sm	0.54594800	-0.08286500	0.01770200
O	0.42391400	-2.63778000	0.60073200
C	-0.70210500	-3.22188200	1.26274700
C	-1.35355500	-4.17182700	0.24398800
C	-0.38950700	-4.15030600	-0.95316100
C	0.88873900	-3.60378900	-0.34370500
I	-0.61463600	0.61493700	2.93601800
I	1.85602200	-0.67512500	-2.87444200
O	2.09279100	2.01801200	0.19995300
C	2.69369200	2.44203100	1.43799500
C	3.69381200	3.52622700	1.06156300
C	4.06318000	3.14332400	-0.36922000
C	2.72640100	2.67290700	-0.90768600
O	-0.70744300	1.94591200	-0.96217800
C	-0.86058500	3.20104000	-0.27917900
C	-1.55559600	4.12437600	-1.26786800
C	-1.08779100	3.56587500	-2.60969900

C	-1.09461200	2.07333100	-2.33771200
O	2.80603400	-0.77898300	1.13968600
C	2.86484600	-1.52454500	2.37214200
C	4.25357000	-2.15448800	2.41542100
C	4.64803900	-2.19238300	0.94088900
C	4.05295400	-0.89109500	0.44352300
H	3.17980300	1.56314300	1.89008600
H	1.89547500	2.77673700	2.11625800
H	2.10269300	3.53012000	-1.22596900
H	2.78719600	1.95136200	-1.73550500
H	3.21421400	4.51831800	1.07600600
H	4.55203400	3.55715100	1.74714900
H	4.48810500	3.97177800	-0.95277200
H	4.78603200	2.31159100	-0.37559100
H	0.14303300	3.56635000	-0.00898500
H	-1.41771500	3.02247300	0.65180700
H	-0.38439200	1.49158100	-2.94365100
H	-1.29050600	5.17926400	-1.11143100
H	-0.06352700	3.90540600	-2.83323200
H	2.06242400	-2.27559100	2.33430600
H	2.65924400	-0.84060900	3.20886300
H	4.69920100	-0.03175500	0.70776200
H	3.83786000	-0.86261100	-0.63474500
H	4.95396900	-1.51506700	2.97619400
H	4.24541200	-3.14318500	2.89493900
H	5.73248300	-2.25766100	0.77608000
H	4.16763600	-3.04129200	0.42802500
H	-1.34208500	-2.39779500	1.60595900
H	-0.34428800	-3.76760300	2.15350800
H	1.46165300	-4.38807300	0.18858000
H	1.54160800	-3.08590700	-1.06074500
H	-0.25192300	-5.13662600	-1.41728100
H	-0.73779800	-3.44620500	-1.72303200
H	-2.65080800	4.03586400	-1.17865400
H	-1.73283700	3.84582200	-3.45411900
H	-2.10485300	1.64503400	-2.46680900
H	-2.36047700	-3.83810100	-0.04120800
H	-1.45094100	-5.18241100	0.66680400

3x _Int I

E_{vacuum} = -25992.0237

E_{sol} = -25992.0379

H_{sol} = -25991.2849

–*TS* = -0.1341

G_{sol} = -25991.4332

C	2.16762679	2.11161375	-0.42131455
C	4.38463228	3.22775204	-0.73253438
C	2.53205653	4.50941798	0.38922402
C	4.01900970	4.20094402	0.38664000
C	1.58734511	3.41128841	-0.05329978
C	3.64006595	1.90797894	-0.56198821
O	1.38557840	1.03100654	-0.39147584
H	4.33494881	3.77061805	1.35276456
H	3.83735529	1.22300417	-1.40710517
H	4.11618771	3.67867561	-1.70442094
H	5.47192471	3.04995844	-0.75204356
H	4.58530979	5.13863002	0.27545170
H	2.27319886	5.51398396	0.04241859
H	4.05316673	1.37845856	0.32779852
H	0.70898651	3.71996908	-0.63482152
C	1.55807547	3.92153276	1.38591798
C	2.03440019	3.01278038	2.48679839
C	0.37190786	4.77081297	1.76878129
H	2.86826979	2.37155298	2.17089027
H	2.36474982	3.58961554	3.36744885
H	1.20870834	2.35468525	2.80106136
H	0.61123885	5.43046119	2.62022288
H	0.05054368	5.40888851	0.93125368
H	-0.48430752	4.13694291	2.05142548
Sm	-0.00515741	-0.52725949	-0.32865576
O	-0.48529957	0.45688551	-2.59042642
C	-0.46068757	1.88143068	-2.77512239
C	0.82748773	2.17904984	-3.54860066
C	1.27750579	0.79940535	-4.06728171
C	0.07879994	-0.09148727	-3.78808878
I	-2.18157378	1.26407711	0.90587488
I	1.95361017	-2.61669248	-1.52040607
O	-1.04857513	-2.47057492	1.03089722

C	-2.41690877	-2.53518075	1.47673961
C	-2.46001217	-3.68275575	2.46860583
C	-1.40662411	-4.62749284	1.89868479
C	-0.33636987	-3.65652171	1.43564583
O	1.08271554	-0.85529201	1.88002437
C	0.46076530	-0.67127215	3.16918690
C	1.60466505	-0.48578765	4.15524263
C	2.75379620	-1.22176717	3.47143794
C	2.51807911	-0.86661991	2.01842210
O	-1.87680621	-1.75744234	-1.60315858
C	-3.05731984	-1.11416411	-2.13187308
C	-3.92175499	-2.24087077	-2.66764442
C	-2.87240311	-3.25577704	-3.10990928
C	-1.85436718	-3.14316473	-1.99220135
H	-3.05980806	-2.72847133	0.60087503
H	-2.68587394	-1.55356604	1.88983349
H	0.35246199	-3.38599830	2.25392918
H	0.26316394	-4.00080289	0.58184686
H	-2.16259310	-3.33606100	3.47169992
H	-3.46064916	-4.12960070	2.54728946
H	-1.02302430	-5.35534032	2.62683394
H	-1.81608775	-5.18722277	1.04201364
H	-0.13025213	-1.57571933	3.38759535
H	-0.22431928	0.18634262	3.10549986
H	2.91140777	-1.58909030	1.29075206
H	1.35895202	-0.88005640	5.15091625
H	2.66617493	-2.31040232	3.62050976
H	-2.73930085	-0.42286492	-2.92742685
H	-3.51309290	-0.52667530	-1.32268584
H	-2.14388276	-3.75907023	-1.12256867
H	-0.82375233	-3.39948141	-2.27375003
H	-4.54937547	-2.66450693	-1.86663015
H	-4.58477360	-1.90663039	-3.47743811
H	-3.26114910	-4.27727334	-3.22210163
H	-2.42239408	-2.95148962	-4.06871468
H	-0.51985942	2.33839992	-1.78083597
H	-1.36133847	2.16078578	-3.34952430
H	-0.67991895	-0.03261504	-4.59147715
H	0.33359469	-1.14109283	-3.59228467
H	1.55106650	0.80318407	-5.13142752
H	2.13829567	0.43012485	-3.49122296
H	1.84967195	0.58175973	4.26158787
H	3.74557047	-0.90593588	3.82295679
H	2.90038914	0.13536642	1.77036411
H	1.58740375	2.61469356	-2.88496671
H	0.63479207	2.88800017	-4.36633653

3x _TS I

$\boldsymbol{E}_{\text{vacuum}}$ = -25992.0071

$\boldsymbol{E}_{\text{sol}}$ = -25992.0211

$\boldsymbol{H}_{\text{sol}}$ = -25991.27035

$-TS$ = -0.1349

$\boldsymbol{G}_{\text{solr}}$ = -25991.4192

C	-2.82346300	0.78851400	0.14714600
C	-4.53967300	2.58378700	0.33261300
C	-5.12437700	0.39763200	-0.82065900
C	-5.63025800	1.55863000	0.03967100
C	-3.65121600	0.13049800	-0.75733600
C	-3.33002600	1.92370200	0.98729700
O	-1.53506500	0.46081600	0.27826800
H	-6.02236100	1.18106900	0.99747400
H	-2.50513400	2.64372400	1.12150300
H	-4.21965700	3.05578000	-0.61353400
H	-4.93689600	3.38748900	0.97351700
H	-6.48121900	2.04333000	-0.46309200
H	-5.51201900	0.44697000	-1.84766700
H	-3.59191100	1.58987600	2.01208900
H	-3.18585300	-0.46175800	-1.54848000
C	-5.12657600	-0.98815700	-0.25771500
C	-5.34266600	-1.22605700	1.20006100
C	-5.34765200	-2.12567600	-1.20055700
H	-4.74076300	-0.54857800	1.82595200
H	-6.40192700	-1.08792400	1.49808100
H	-5.06979900	-2.25796600	1.47069600
H	-6.42865200	-2.33088200	-1.34321200
H	-4.93062300	-1.91273800	-2.19774200
H	-4.88749700	-3.06002400	-0.83855400
Sm	0.50063200	0.00777700	0.05432800

O	0.42576000	2.24425600	-1.04227200
C	-0.70259600	2.66462800	-1.83654100
C	-1.08631600	4.06070600	-1.32399800
C	-0.08068700	4.33704700	-0.19880100
C	1.07355900	3.42552300	-0.55823600
I	-0.06317500	-1.26064400	-2.71053000
I	1.45272000	1.32625200	2.68249600
O	2.23778400	-1.84391000	0.35527600
C	2.93465300	-2.54669500	-0.69384200
C	3.68338200	-3.67036100	0.00106500
C	3.96739600	-3.06245300	1.37087400
C	2.67595900	-2.31845700	1.64588800
O	-0.29907100	-1.86292700	1.49485800
C	-0.76924400	-3.03942200	0.80838000
C	-2.23100800	-3.13381800	1.18497300
C	-2.18797200	-2.70535200	2.64881400
C	-1.15760200	-1.58570100	2.63006000
O	2.78410900	0.68924500	-0.91638100
C	3.01227300	1.03323700	-2.30138200
C	4.51527600	1.20530300	-2.43216600
C	4.90115700	1.69034400	-1.03844500
C	4.00228100	0.83929300	-0.16397900
H	3.62165900	-1.83686200	-1.18435600
H	2.19599400	-2.87639300	-1.43678500
H	1.89895000	-2.98390800	2.05550000
H	2.77404000	-1.44676500	2.30775400
H	3.03659900	-4.55635900	0.10426400
H	4.58646700	-3.97071400	-0.54789300
H	4.19383100	-3.80652000	2.14691100
H	4.81430200	-2.35919800	1.31433500
H	-0.19194200	-3.91263200	1.16498500
H	-0.59023200	-2.90351500	-0.26838800
H	-0.52660000	-1.53616400	3.52824800
H	-2.64505800	-4.14000500	1.03128900
H	-1.84920900	-3.54044100	3.28307000
H	2.46911000	1.96747600	-2.51287400
H	2.58245900	0.23659000	-2.92385800
H	4.43840700	-0.16078100	0.00727500
H	3.74486300	1.28010500	0.80880800
H	4.99891100	0.23850200	-2.64721600
H	4.78451500	1.90446400	-3.23575700
H	5.96568600	1.55788200	-0.80101900
H	4.65169500	2.75695200	-0.91773200
H	-1.48919900	1.91160900	-1.70683300
H	-0.39165300	2.67620600	-2.89360400
H	1.70106600	3.85210200	-1.36447900
H	1.70274100	3.13686600	0.29359200
H	0.21588900	5.39284600	-0.13162900
H	-0.48298300	4.02772500	0.77789600
H	-2.80951900	-2.41178200	0.58721500
H	-3.15776000	-2.35966000	3.03065300
H	-1.61318300	-0.60092700	2.45582100
H	-2.12341200	4.09002000	-0.96393700
H	-0.99291400	4.80553700	-2.12794700

3x _Int II

$\boldsymbol{E}_{vacuum}$ = -25992.0287

$\boldsymbol{E}_{sol}$ = -25992.0435

$\boldsymbol{H}_{sol}$ = -25991.2906

–*TS* = -0.1372

$\boldsymbol{G}_{sol}$ = -25991.4426

C	-2.74555300	-0.87864000	-0.11417300
C	-4.53876600	-2.35097800	-1.07019200
C	-5.15773900	-0.47811000	0.51727000
C	-5.48435200	-1.18686500	-0.81241000
C	-3.67249500	-0.28353900	0.66699200
C	-3.10644100	-1.85365900	-1.20214300
O	-1.44563300	-0.63342100	0.01654400
H	-5.38814500	-0.45730700	-1.63705500
H	-2.38162600	-2.68296900	-1.17784100
H	-4.60498200	-3.06301400	-0.22787400
H	-4.83706900	-2.90387000	-1.97514100
H	-6.53350100	-1.52289100	-0.80497900
H	-5.51483500	-1.13686000	1.33593000
H	-2.94546400	-1.36632200	-2.18150700
H	-3.31895800	0.39388300	1.45071600
C	-5.91934100	0.81513600	0.64129500
C	-5.46219200	2.01262300	-0.11874600

C	-7.33594900	0.77465400	1.09838700
H	-4.37121300	1.99979100	-0.26558800
H	-5.92821700	2.06923800	-1.12612200
H	-5.73442500	2.94938800	0.39655700
H	-8.04086100	0.59675000	0.25781200
H	-7.50612700	-0.03046800	1.83079500
H	-7.64375000	1.72922000	1.55745600
Sm	0.57291500	-0.06696100	0.05691400
O	0.55613800	-2.07084200	1.55442200
C	-0.57235800	-2.28837400	2.41957800
C	-1.33379200	-3.47361300	1.81909000
C	-0.34858600	-4.08150900	0.80235900
C	0.95921500	-3.36838000	1.10388000
I	0.39122800	1.64257200	2.62223700
I	1.10117700	-1.71530000	-2.50150500
O	2.34510800	1.56402400	-0.80662900
C	3.17582800	2.40518500	0.01652300
C	3.68682300	3.48668800	-0.91496700
C	3.82690000	2.72212000	-2.22757200
C	2.60538000	1.82003500	-2.20380400
O	-0.44547800	1.81547300	-1.22819400
C	-0.37023500	3.21016800	-0.86976300
C	-1.70622900	3.79553100	-1.28184500
C	-2.03267300	2.97539500	-2.52607600
C	-1.57627800	1.59137700	-2.10555400
O	2.92795100	-0.75307000	0.79375300
C	3.33729000	-0.90107000	2.17182200
C	4.84866700	-1.01658900	2.12549400
C	5.06325600	-1.73738400	0.79859100
C	4.02611300	-1.07006400	-0.08452100
H	4.00279700	1.79549600	0.42117000
H	2.56686900	2.76444500	0.85731600
H	1.71528300	2.31066900	-2.63015900
H	2.73833400	0.85305200	-2.70790800
H	2.94054000	4.29143000	-1.01338600
H	4.62576600	3.93422800	-0.56113500
H	3.84930700	3.36783300	-3.11612900
H	4.75035400	2.12059900	-2.22223800
H	0.46135800	3.67064000	-1.43212500
H	-0.16160300	3.27350500	0.20729000
H	-1.23595000	0.94635300	-2.92762800
H	-1.65025200	4.87735700	-1.46511500
H	-1.45352000	3.33794400	-3.39077400
H	2.86729700	-1.81205900	2.57483500
H	2.95482700	-0.03618700	2.73100600
H	4.40817000	-0.13009200	-0.51842900
H	3.63950400	-1.69509000	-0.90107400
H	5.31283400	-0.01711100	2.09935700
H	5.25161500	-1.55615200	2.99357200
H	6.08091900	-1.63659300	0.39691300
H	4.84188700	-2.81152900	0.90567000
H	-1.14401000	-1.35377300	2.45501400
H	-0.18328200	-2.51226400	3.42760200
H	1.52461200	-3.86371900	1.91585200
H	1.60831600	-3.23481800	0.22855900
H	-0.25252000	-5.17249300	0.89100300
H	-0.65271400	-3.84758200	-0.22800900
H	-2.45627700	3.61492000	-0.49575600
H	-3.09816900	2.99111500	-2.79393300
H	-2.35486700	1.06977000	-1.53022700
H	-2.25619600	-3.13498000	1.32904500
H	-1.61387800	-4.19144200	2.60300400

3x _PreTS II

$\boldsymbol{E}_{vacuum}$ = -26300.2899

$\boldsymbol{E}_{sol}$ = -26300.3046

$\boldsymbol{H}_{sol}$ = -26299.4304

$-TS$ = -0.1564

$\boldsymbol{G}_{sol}$ = -26299.6014

C	-2.00094700	-1.73589000	0.64811100
C	-3.39880400	-2.93581300	2.35681300
C	-4.48711700	-1.97093100	0.29052400
C	-4.37590500	-3.19566800	1.21960300
C	-3.12514800	-1.41707200	-0.02876900
C	-1.99824300	-2.68841300	1.81447300
O	-0.81457800	-1.22427100	0.33549100
H	-4.02450800	-4.06189800	0.62983000
H	-1.32444200	-2.28548200	2.58776500

H	-3.73571100	-2.04987500	2.92458900
H	-3.39087000	-3.77869200	3.06603000
H	-5.37222400	-3.45759300	1.60990200
H	-5.05710800	-1.19552500	0.84497600
H	-1.53497000	-3.64091000	1.49557800
H	-3.04603800	-0.68462600	-0.83747000
C	-5.29457400	-2.29573500	-0.93836600
C	-4.65110700	-2.99618200	-2.08482300
C	-6.77626500	-2.39257300	-0.80209600
H	-3.60318000	-2.68701200	-2.21246900
H	-4.64716100	-4.09834600	-1.94287000
H	-5.18988500	-2.81052300	-3.02867400
H	-7.09781000	-3.40340900	-0.47194600
H	-7.16760000	-1.67991200	-0.05779500
H	-7.28935600	-2.21099200	-1.76177100
Sm	1.04825300	-0.28857100	0.06499000
O	-0.10067700	1.30935800	1.57575100
C	-1.49469900	1.62839000	1.40045400
C	-2.19465300	1.14140300	2.67160000
C	-1.04136700	0.84240500	3.64784700
C	0.16425300	1.47292500	2.97189000
I	0.14244000	1.41370900	-2.37087800
I	2.38067600	-1.71850800	2.42974400
O	3.34261900	-0.44302700	-1.07874800
C	3.87730700	0.50931600	-2.01490500
C	4.74510200	-0.31545800	-2.94091400
C	5.36429900	-1.30700300	-1.96048700
C	4.20486100	-1.60551900	-1.02098300
O	1.24893600	-2.38078800	-1.34199700
C	0.90624200	-2.36146300	-2.73468700
C	-0.53500300	-2.83508600	-2.75518500
C	-0.57771300	-3.87430400	-1.62192600
C	0.70675600	-3.61003400	-0.82570400
O	2.50054800	1.71212000	0.67139200
C	2.10866100	3.08417900	0.55455200
C	3.38291200	3.77101600	0.10709800
C	4.46254500	3.02980300	0.90853800
C	3.80337200	1.68944400	1.27314400
H	4.47999500	1.25564900	-1.46669100
H	3.02909800	1.01517200	-2.49569500
H	3.60762900	-2.46782900	-1.34894500
H	4.49748100	-1.76401800	0.02670500
H	4.11700900	-0.83714800	-3.68098900
H	5.48415600	0.29204500	-3.48121200
H	5.75233100	-2.21726300	-2.43786900
H	6.19544000	-0.83088800	-1.41600000
H	1.58087000	-3.05422100	-3.27260400
H	1.05177800	-1.33768500	-3.10374300
H	1.45998100	-4.40106800	-0.98424200
H	-0.82840900	-3.24395400	-3.73181500
H	-0.58568800	-4.90322800	-2.00936300
H	1.76673700	3.45367000	1.53851200
H	1.28124100	3.13103500	-0.16448000
H	4.31917400	0.80464900	0.88107900
H	3.69054200	1.56013800	2.36162700
H	3.51615400	3.61642800	-0.97433600
H	3.37094800	4.85300400	0.29787100
H	5.38605800	2.89194800	0.32865600
H	4.73003800	3.58466700	1.81941100
H	-1.83405100	1.13456600	0.48334100
H	-1.57523600	2.71922500	1.27602300
H	0.23844300	2.55464900	3.19249700
H	1.11834100	0.97778400	3.19494000
H	-1.20883900	1.24779400	4.65538100
H	-0.87470900	-0.24062300	3.74011200
H	-1.19348600	-1.98523500	-2.52102800
H	-1.47407700	-3.73517300	-1.00327800
H	0.56141900	-3.47224400	0.25216200
H	-2.79267000	0.24356700	2.46656400
H	-2.87150900	1.91795700	3.05486400
C	-2.83040400	3.68343600	-1.10748200
C	-3.91593200	3.26240800	-0.32305400
C	-4.19598500	3.93234000	0.87950400
C	-3.39930000	4.99665700	1.28899000
C	-2.31740400	5.40601800	0.50765500
C	-2.03759200	4.74679700	-0.68950200
C	-4.69452900	2.13612400	-0.72653200
C	-5.32764400	1.15838600	-1.06888900

H	-5.84535000	0.26453300	-1.36356900
H	-5.03887200	3.60178000	1.48823700
H	-3.62351800	5.51037200	2.22599900
H	-1.69282900	6.24034500	0.83348400
H	-1.18714100	5.05055000	-1.30273900
H	-2.59511600	3.14904700	-2.02785400

3x_TS II

$\boldsymbol{E}_{\mathbf{vacuum}}$ = -26300.2828

$\boldsymbol{E}_{\mathbf{sol}}$ = -26300.2975

$\boldsymbol{H}_{\mathbf{sol}}$ = -26299.4245

–TS = -0.1532

$\boldsymbol{G}_{\mathbf{sol}}$ = -26299.5924

C	-1.95088300	-1.46698400	0.57682200
C	-3.27463200	-2.65433300	2.34153700
C	-4.46364300	-1.58119000	0.37669600
C	-4.35503100	-2.82268000	1.28446200
C	-3.10347300	-1.06303800	0.00062000
C	-1.91289700	-2.48354200	1.68652400
O	-0.76554100	-0.99184400	0.20655300
H	-4.11038100	-3.70324800	0.66248700
H	-1.14149200	-2.17792700	2.41096200
H	-3.50248200	-1.76411800	2.95507700
H	-3.26560200	-3.51588500	3.02800700
H	-5.33039600	-3.02650700	1.75289700
H	-4.99089000	-0.79289500	0.95427400
H	-1.55719700	-3.44813900	1.27820700
H	-3.04795400	-0.29412400	-0.77394900
C	-5.34027900	-1.88311700	-0.81375700
C	-4.73525000	-2.55211200	-2.00300500
C	-6.77310700	-2.19303400	-0.52597300
H	-3.76607000	-2.10867700	-2.27206100
H	-4.55722900	-3.63094100	-1.81089000
H	-5.40201000	-2.50073100	-2.87967900
H	-6.90154200	-3.24355000	-0.19438700
H	-7.17720800	-1.54596100	0.26813200
H	-7.40692200	-2.07502600	-1.42078000
Sm	1.16850000	-0.18981900	0.02381000
O	0.17957000	1.22523500	1.80562400
C	-1.14647100	1.76735900	1.67543300
C	-1.93143600	1.23964800	2.87687700
C	-0.84992500	0.68267400	3.82287500
C	0.45212400	1.17268900	3.20832500
I	0.36837700	1.99031100	-2.04309900
I	2.40314000	-2.09236200	2.09253100
O	3.41057500	-0.35182200	-1.20851900
C	4.00802200	0.69763900	-1.98921100
C	4.73527900	-0.02789400	-3.10034200
C	5.29194500	-1.23448200	-2.35034400
C	4.16227200	-1.57709400	-1.38796000
O	1.13866100	-2.04559900	-1.69534500
C	0.68370500	-1.78096300	-3.03014600
C	-0.78517600	-2.15941700	-2.99213000
C	-0.81146400	-3.36316600	-2.03526700
C	0.54570200	-3.30422200	-1.32321500
O	2.82337300	1.52694100	0.91950900
C	2.58156500	2.93303800	1.03395300
C	3.92175700	3.54664500	0.68455400
C	4.91158400	2.57921900	1.34662900
C	4.12530100	1.26392600	1.46685100
H	4.71461600	1.26365900	-1.35537300
H	3.20151600	1.36670800	-2.31834800
H	3.46718700	-2.32222500	-1.79846800
H	4.50097700	-1.92302100	-0.40082900
H	4.01792100	-0.34362400	-3.87489200
H	5.50845700	0.59107400	-3.57597700
H	5.54719400	-2.08060300	-3.00300000
H	6.20153900	-0.94878000	-1.79834500
H	1.25976400	-2.41560300	-3.72980900
H	0.87418500	-0.72154100	-3.24774900
H	1.22410500	-4.10661800	-1.66120400
H	-1.18429300	-2.38838700	-3.98982900
H	-0.92115300	-4.31389900	-2.57658700
H	2.28015200	3.17185400	2.07032500
H	1.76603900	3.18388600	0.34387100
H	4.54620200	0.42817100	0.89474900
H	4.01173600	0.93322000	2.51137000
H	4.04329300	3.55229700	-0.40927000

H	4.02231200	4.58002300	1.04396600
H	5.83167400	2.45851400	0.75765800
H	5.20707200	2.94029300	2.34246400
H	-1.54302900	1.45212400	0.70408900
H	-1.06505900	2.86613500	1.68406100
H	0.70900400	2.19091600	3.55701400
H	1.30710400	0.50037600	3.35852500
H	-0.96521300	1.02380200	4.86108500
H	-0.86159400	-0.41647900	3.82599800
H	-1.36061500	-1.32294900	-2.56877800
H	-1.64487300	-3.27489800	-1.32613200
H	0.49266800	-3.32664200	-0.22809800
H	-2.63233200	0.45532600	2.56200500
H	-2.51826200	2.04705700	3.33654100
C	-3.46081800	2.90938000	-0.97247300
C	-4.62141200	2.29935800	-0.45816900
C	-5.14188900	2.75283500	0.76944500
C	-4.52688700	3.79791800	1.44847100
C	-3.38845800	4.41031900	0.92029500
C	-2.85734800	3.95700600	-0.28814900
C	-5.26523600	1.26442500	-1.18336400
C	-5.76838900	0.30825700	-1.76550100
H	-6.35923300	-0.22628000	-2.48766900
H	-6.03647600	2.27696000	1.17385400
H	-4.94387900	4.14353400	2.39683700
H	-2.91432700	5.23741300	1.45285800
H	-1.95390800	4.40899200	-0.70203700
H	-3.03450800	2.55074500	-1.91006400

3x_Int III

$\boldsymbol{E}_{\mathbf{vacuum}}$ = -26300.3351

$\boldsymbol{E}_{\mathbf{sol}}$ = -26300.3505

$\boldsymbol{H}_{\mathbf{sol}}$ = -26299.4738

–*TS* = -0.1506

$\boldsymbol{G}_{\mathbf{sol}}$ = -26299.6397

C	-1.93160200	-1.09426400	1.13446800
C	-3.34054100	-1.94934000	3.01402000
C	-4.41749500	-1.38813100	0.80680400
C	-4.31391400	-2.41361300	1.93825300
C	-3.05090600	-0.90604100	0.40521000
C	-1.93257300	-1.81070900	2.45522300
O	-0.74515000	-0.66218900	0.71845300
H	-3.96467100	-3.38190700	1.53615300
H	-1.26731200	-1.27162000	3.14822000
H	-3.67775800	-0.97150000	3.40262700
H	-3.34743800	-2.64575700	3.86714300
H	-5.30414000	-2.59177200	2.38340900
H	-4.99280400	-0.52427800	1.20040400
H	-1.45986700	-2.80107700	2.32004400
H	-2.94079300	-0.35787500	-0.53241200
C	-5.23597100	-1.89069400	-0.41261300
C	-4.46243000	-2.93188700	-1.22916500
C	-6.57206900	-2.49284100	0.03870900
H	-3.51380100	-2.51764200	-1.60084800
H	-4.23322100	-3.81985700	-0.61928000
H	-5.05700000	-3.26593800	-2.09429400
H	-6.42508100	-3.43740500	0.58342800
H	-7.11775100	-1.79487200	0.69281600
H	-7.21214900	-2.70805100	-0.83130800
Sm	1.14739400	-0.06774400	0.05826000
O	0.40531000	1.91911400	1.37387600
C	-0.96589400	2.34971200	1.28060300
C	-1.59005100	2.05025100	2.64438000
C	-0.38385500	1.77381400	3.56261900
C	0.81242100	2.18600700	2.71959600
I	0.22691600	1.44835000	-2.48482000
I	2.40418400	-1.44337100	2.52447800
O	3.25545800	-0.77774800	-1.19665400
C	3.82280500	-0.07383100	-2.31799800
C	4.72123100	-1.08480500	-3.00280700
C	5.24018200	-1.88925600	-1.81500400
C	4.01142400	-1.97792000	-0.92659200
O	0.79706300	-2.20895400	-1.18972500
C	0.91312600	-2.37812800	-2.61666100
C	-0.15948600	-3.38465400	-2.98295700
C	-0.17417500	-4.28072600	-1.74869000
C	-0.03028600	-3.26151600	-0.63498500
O	3.00203300	1.69557700	0.22569200

C	2.85265300	3.08319200	-0.15666200
C	4.15448100	3.76394200	0.24153100
C	4.65977700	2.87432600	1.37326400
C	4.27526400	1.50082000	0.86512200
H	4.39789800	0.78834100	-1.93844000
H	2.99543500	0.30514900	-2.93357200
H	3.37506800	-2.84236400	-1.17720200
H	4.22580900	-2.01027700	0.15026600
H	4.13180000	-1.72639200	-3.67746200
H	5.51455900	-0.60677200	-3.59376000
H	5.62865800	-2.88021600	-2.08710400
H	6.04538100	-1.33758300	-1.30351500
H	1.92211900	-2.76644800	-2.84212500
H	0.78601000	-1.39328200	-3.08728800
H	0.46915100	-3.63424900	0.27007000
H	0.06721200	-3.91692200	-3.91696800
H	0.68227300	-4.97418600	-1.76514300
H	1.98241800	3.47960400	0.38630700
H	2.63272000	3.12331900	-1.23246800
H	5.00269500	1.13192100	0.11885300
H	4.14531700	0.73513300	1.64214500
H	4.87049900	3.75339200	-0.59582400
H	3.99911100	4.81042100	0.53782800
H	5.73798900	2.96899600	1.56205100
H	4.12404300	3.09335500	2.31100200
H	-1.42928200	1.81194000	0.44677500
H	-0.96657700	3.42842800	1.05061700
H	1.03336800	3.26629100	2.81313800
H	1.72117300	1.60262800	2.91873400
H	-0.42576600	2.32585300	4.51182800
H	-0.30542500	0.70226700	3.79511300
H	-1.12991700	-2.87627900	-3.09905600
H	-1.09451900	-4.87208700	-1.64626000
H	-1.00181600	-2.82513000	-0.36132500
H	-2.25390100	1.17801500	2.58046500
H	-2.19169600	2.90310000	2.98830100
C	-3.61999100	2.29214500	-1.40893800
C	-4.84434100	1.79839700	-0.86491000
C	-5.58349800	2.65588500	0.00406700
C	-5.11207800	3.92020900	0.31050300
C	-3.90765300	4.38778800	-0.23285000
C	-3.17285400	3.56271900	-1.09425200
C	-5.29203000	0.53321100	-1.18106100
C	-5.58448900	-0.72822700	-1.34329400
H	-6.17479300	-1.02547800	-2.22614900
H	-6.52136200	2.29241000	0.42682900
H	-5.68915900	4.55924100	0.98267800
H	-3.54828200	5.38941900	0.01029200
H	-2.22842500	3.90763300	-1.52122000
H	-3.03155200	1.65517900	-2.07125800

3x_TS III

$\boldsymbol{E}_{vacuum}$ = -26300.3336

$\boldsymbol{E}_{sol}$ = -26300.3480

$\boldsymbol{H}_{sol}$ = -26299.4728

$-TS$ = -0.1488

$\boldsymbol{G}_{sol}$ = -26299.6360

C	2.17341800	-0.87803000	0.82495800
C	3.49139600	-1.92337300	2.67837400
C	4.47608100	-1.83641300	0.36010100
C	4.73632900	-1.63094100	1.85247300
C	3.18456100	-1.17579500	-0.04513200
C	2.35271100	-0.98298200	2.31119600
O	1.00352100	-0.42614700	0.40387200
H	5.04886400	-0.58535000	2.02012700
H	1.39689200	-1.29965600	2.75891700
H	3.17476600	-2.96737300	2.50134200
H	3.71553500	-1.84580600	3.75384300
H	5.56443500	-2.27503000	2.18415000
H	4.38424200	-2.92934700	0.18604100
H	2.55551100	0.03047200	2.70721800
H	2.93683200	-1.13677000	-1.10785300
C	5.63505300	-1.35698400	-0.55194100
C	5.49098200	-1.97903900	-1.94885500
C	7.00610800	-1.74829200	0.00260500
H	4.51094000	-1.75588200	-2.39508900
H	6.25961000	-1.58481800	-2.63181900
H	5.61162000	-3.07418700	-1.90085800

H	7.22842800	-1.23991600	0.95136100
H	7.06703800	-2.83574700	0.17131800
H	7.79803400	-1.47799500	-0.71363600
Sm	-1.05546300	-0.17850100	-0.02971900
O	-0.55460400	-2.48962800	-0.93179100
C	0.46055100	-2.63969100	-1.95228900
C	1.29278600	-3.85873700	-1.55808700
C	0.94032800	-4.07055500	-0.08671200
C	-0.50382300	-3.62136800	-0.04573300
I	-0.80993000	0.94600200	-2.87049300
I	-1.81074400	-1.12209300	2.81119100
O	-3.14745700	1.27874400	0.17496700
C	-4.11948300	1.51603900	-0.84667400
C	-4.51095200	2.97138200	-0.64898200
C	-4.37722300	3.17577600	0.87216400
C	-3.64042900	1.92062900	1.35839600
O	-0.51633700	2.04083900	0.87528400
C	-0.67863400	3.30646000	0.21702200
C	-0.48366300	4.35097900	1.31371300
C	0.28934200	3.60178300	2.41781400
C	0.55389600	2.22723300	1.81895600
O	-3.17644500	-1.37753600	-0.83516600
C	-3.32792500	-1.98365000	-2.12310100
C	-3.75860100	-3.42495700	-1.84319000
C	-4.28054100	-3.38058000	-0.39216200
C	-4.25093000	-1.89783300	-0.04729400
H	-4.98006100	0.84015600	-0.69173100
H	-3.64406200	1.29825400	-1.81150900
H	-2.77488000	2.11352600	2.00237000
H	-4.31285200	1.22154900	1.88288500
H	-3.81216000	3.62009500	-1.19777600
H	-5.52213900	3.18108800	-1.02387200
H	-3.81363600	4.09066000	1.10340900
H	-5.35644600	3.26405500	1.36318400
H	-1.66842500	3.30179000	-0.25137400
H	0.08669900	3.38890800	-0.56834300
H	0.49909900	1.39974600	2.53589500
H	-1.44858700	4.71963200	1.69147400
H	-0.31897800	3.51519900	3.32960900
H	-2.37860900	-1.87486200	-2.65821200
H	-4.10545500	-1.42597300	-2.67500100
H	-5.18825600	-1.39492500	-0.35085000
H	-4.03182000	-1.67549900	1.00476000
H	-4.52756600	-3.75193800	-2.55687900
H	-2.90698300	-4.11284500	-1.93656400
H	-5.28780400	-3.80556000	-0.28347000
H	-3.61029400	-3.93057300	0.28397200
H	1.03966300	-1.70732200	-1.96723000
H	-0.03985400	-2.75311600	-2.92625000
H	-1.18444400	-4.40471500	-0.42730600
H	-0.85438000	-3.28869200	0.94176200
H	1.06766500	-5.11032900	0.24490300
H	1.55327000	-3.41491600	0.54921800
H	0.07541800	5.21370200	0.92702600
H	1.23142100	4.09944200	2.68387600
H	1.51214700	2.18770600	1.27770100
H	2.36609200	-3.68345200	-1.71499800
H	1.00130300	-4.73691200	-2.15492200
C	3.87161900	2.11276100	-0.36483500
C	3.01250000	2.55571400	-1.40013500
C	2.48050100	3.83693700	-1.37716200
C	2.75856000	4.70810800	-0.31788200
C	3.56936900	4.27004400	0.73339800
H	4.75537100	2.65431600	1.53565400
C	4.44375300	0.82251500	-0.39493600
C	5.53555500	0.14778900	-0.68552100
H	6.41881600	0.66710200	-1.09389800
H	1.84123900	4.16152200	-2.20161400
H	2.35067500	5.72090200	-0.31448800
H	3.79243600	4.94116700	1.56646600
C	4.11174000	2.99153100	0.72093000
H	2.77899600	1.87944400	-2.22352700

3x_Int IV

E_{vacuum} = -26300.3719

E_{sol} = -26300.3869

H_{sol} = -26299.5086

$-TS$ = -0.1469

G_{sol} = -26299.6705

C	2.12584200	-0.53668200	1.21945000
C	2.82080400	-2.61217800	2.40196600
C	3.50913600	-2.29299500	-0.03258200
C	3.90661000	-2.81206400	1.35139000
C	3.02319400	-0.81528200	0.04914800
C	2.49305800	-1.12199700	2.54904500
O	0.82006900	-0.43128300	0.94487600
H	4.81568400	-2.28027000	1.68583600
H	1.66777500	-0.95447300	3.25718900
H	1.90745400	-3.15945200	2.11092700
H	3.14652200	-3.03497500	3.36738600
H	4.17576700	-3.87806800	1.28320300
H	2.70671700	-2.93152100	-0.43943600
H	3.39119500	-0.62291400	2.96146000
H	2.40910200	-0.59347900	-0.84246200
C	4.70113300	-2.23125700	-1.02747200
C	4.17895100	-2.28450800	-2.47165300
C	5.75589400	-3.31978900	-0.84267100
H	3.36443500	-1.56355800	-2.63677400
H	4.98500200	-2.05899200	-3.18790300
H	3.79077900	-3.28927600	-2.70608500
H	6.23817300	-3.27185200	0.14385700
H	5.31665700	-4.32336000	-0.96226900
H	6.54509400	-3.21322300	-1.60419500
Sm	-1.06346300	-0.01080800	0.11136600
O	-1.36183000	-2.44548300	0.57141800
C	-0.32892200	-3.38571700	0.19235600
C	-0.43969500	-4.54468700	1.17575200
C	-1.14571700	-3.90814000	2.37035800
C	-2.11030400	-2.96642300	1.68240600
I	-0.10904900	-0.81413400	-2.72078300
I	-2.40290800	0.81908500	2.77251600
O	-2.50305900	1.77017800	-1.06862900
C	-2.98569400	1.71324500	-2.42517100
C	-3.49121800	3.11140800	-2.72979400
C	-3.98592800	3.57019800	-1.36181500
C	-2.92180300	3.00040700	-0.44338800
O	0.02967500	2.19576700	0.00391100
C	0.55268500	2.79835300	-1.20016500
C	1.62493700	3.76975100	-0.73572100
C	1.15000000	4.13624900	0.66648700
C	0.63836500	2.80249500	1.16658700
O	-3.40172400	-0.89819500	-0.56023700
C	-3.61422800	-2.00662100	-1.45887100
C	-5.10181200	-2.00007900	-1.75789100
C	-5.67832500	-1.46825100	-0.44972000
C	-4.66557500	-0.40089300	-0.08170800
H	-3.79715500	0.96697900	-2.47372500
H	-2.16232700	1.36944000	-3.06566700
H	-2.04722000	3.66768700	-0.36828200
H	-3.26619100	2.76277100	0.57261900
H	-2.66351800	3.75310700	-3.07286000
H	-4.26788400	3.11435400	-3.50692300
H	-4.07851800	4.66083700	-1.26676800
H	-4.96871300	3.12396000	-1.13818800
H	-0.28358500	3.30935300	-1.70741900
H	0.92343000	1.99911200	-1.85734200
H	-0.13380600	2.86078000	1.94536700
H	1.72118800	4.63235800	-1.40938300
H	0.33171300	4.87465600	0.62839500
H	-3.30420300	-2.93095100	-0.94527700
H	-2.96575400	-1.86145000	-2.33407000
H	-4.88260000	0.55389400	-0.59158500
H	-4.56705700	-0.20510800	0.99455300
H	-5.32752600	-1.30822000	-2.58580000
H	-5.47530400	-2.99525500	-2.03591500
H	-6.69618200	-1.06535900	-0.54369300
H	-5.69304400	-2.26206700	0.31456200
H	0.62683400	-2.85067600	0.26806200
H	-0.48920300	-3.67136500	-0.85693400
H	-2.99864900	-3.50229600	1.29816600
H	-2.43641400	-2.11567300	2.29633000
H	-1.65177800	-4.63527300	3.02013500
H	-0.43562400	-3.32741700	2.98002300
H	2.60123900	3.26702200	-0.67827600
H	1.95316500	4.53637200	1.29874100
H	1.45523600	2.14828000	1.50869000

H	0.54463400	-4.96538700	1.42440200
H	-1.05618000	-5.35534100	0.75661700
C	4.55239300	1.36744600	0.16971400
C	5.17467700	2.20340200	-0.77166200
C	5.43363800	3.54142800	-0.48565700
C	5.07310900	4.07630400	0.75078400
C	4.44877300	3.26020600	1.69330500
H	3.70253900	1.29112100	2.15040300
C	4.33601900	-0.05492500	-0.13491900
C	5.23571900	-0.84671500	-0.74045600
H	6.23730600	-0.52889100	-1.04340500
H	5.91522100	4.17230200	-1.23589100
H	5.27719400	5.12458100	0.97835700
H	4.17037100	3.66564700	2.66884700
C	4.18266100	1.92352500	1.40449200
H	5.44117700	1.79001200	-1.74641200

3x_Product

$\boldsymbol{E}_{\mathbf{vacuum}}$ = -26300.3911

$\boldsymbol{E}_{\mathbf{sol}}$ = -26300.4118

$\boldsymbol{H}_{\mathbf{sol}}$ = -26299.5263

–*TS* = -0.1510

$\boldsymbol{G}_{\mathbf{sol}}$ = -26299.6981

C	-2.15557900	-0.89571000	-1.42967400
C	-3.29069200	-2.97481600	-2.05514500
C	-3.65163000	-2.04948300	0.28261400
C	-4.26728800	-2.78926000	-0.90540500
C	-2.93322700	-0.74236900	-0.15183800
C	-2.83008000	-1.60553500	-2.56403000
O	-0.98637800	-0.53751600	-1.51504500
H	-5.14084700	-2.22765700	-1.27994300
H	-2.13634300	-1.67036100	-3.41214400
H	-2.41445100	-3.55934000	-1.72451100
H	-3.75818800	-3.53634900	-2.87840800
H	-4.64053700	-3.76751100	-0.56730200
H	-2.92076600	-2.70532800	0.78186900
H	-3.71711400	-1.02574100	-2.87328300
H	-2.18160200	-0.46035000	0.60345500
C	-4.72212100	-1.53518000	1.28520300
C	-4.09516600	-1.35324700	2.67634800
C	-5.95709000	-2.42361200	1.41849200
H	-3.16199500	-0.77513100	2.62758700
H	-4.78936700	-0.82968800	3.35233200
H	-3.85207500	-2.33105200	3.12014900
H	-6.51626900	-2.51443800	0.47690200
H	-5.67996600	-3.43491500	1.75480500
H	-6.64148100	-2.00258600	2.17167000
Sm	1.11344700	-0.05438000	-0.06005000
O	1.72866700	-1.79954500	-1.92947700
C	1.01167200	-3.02484400	-2.13855400
C	0.62034400	-3.05202200	-3.62404700
C	1.06089800	-1.67900100	-4.14118000
C	2.19993300	-1.34316200	-3.19989500
I	0.08356000	-2.50386100	1.77537500
I	2.04381800	2.43061100	-1.86144100
O	2.24085500	1.02204900	2.02237800
C	2.79324600	0.27166600	3.11664300
C	3.24995800	1.31013400	4.12771800
C	3.62978600	2.48071600	3.22447800
C	2.53791500	2.41787800	2.17222800
O	-0.54169300	1.52927600	1.17863500
C	-0.92441200	1.30773100	2.54056300
C	-2.09524800	2.24271600	2.76050600
C	-1.63817400	3.47331700	1.98108800
C	-0.92819200	2.86412400	0.77690200
O	3.57217000	-0.84509500	0.22820000
C	4.00011200	-2.21869900	0.23445000
C	5.51969500	-2.17253300	0.20347300
C	5.77858300	-0.87347000	-0.55490900
C	4.69393400	0.02326500	0.00991700
H	3.63628800	-0.32670700	2.73241100
H	2.02140800	-0.42060400	3.48284100
H	1.62612300	2.94145200	2.51328700
H	2.81819600	2.81446300	1.18541700
H	2.41746000	1.59561000	4.79137700
H	4.07578700	0.94862400	4.75597200
H	3.66056000	3.44897800	3.74299300
H	4.61612800	2.30795900	2.76397400

H	-0.07806400	1.55807300	3.20631400
H	-1.13922100	0.23542800	2.65559800
H	-0.02825800	3.41190500	0.46198400
H	-2.28767100	2.44070000	3.82429000
H	-0.93634300	4.06576600	2.59042200
H	3.58530900	-2.70376500	-0.66282400
H	3.57483700	-2.70808800	1.12270900
H	5.00212500	0.46020100	0.97823300
H	4.37082800	0.83549100	-0.65810700
H	5.92868800	-2.10435400	1.22471300
H	5.95416000	-3.06034000	-0.27666500
H	6.78744200	-0.46512000	-0.40411700
H	5.62325600	-1.01958000	-1.63620300
H	0.16108000	-3.02405000	-1.44354900
H	1.66426100	-3.87201700	-1.86707400
H	3.12660600	-1.88784700	-3.46801200
H	2.41837600	-0.26900600	-3.10972300
H	1.36243400	-1.68846900	-5.19771600
H	0.26304400	-0.93341300	-4.00612900
H	-3.00681700	1.81486000	2.31337100
H	-2.46342700	4.13043800	1.67563800
H	-1.59430100	2.76910800	-0.09089700
H	-0.45420800	-3.23512300	-3.76520600
H	1.15934400	-3.85479200	-4.14953100
C	-3.99863800	1.64307000	-0.67924200
C	-4.64436200	2.70946100	-0.02830000
C	-4.59198700	4.00259100	-0.53563900
C	-3.88130600	4.27157000	-1.70567400
C	-3.23345400	3.22955700	-2.36324000
H	-2.76177200	1.15054200	-2.39490100
C	-4.06017100	0.28951300	-0.10327000
C	-5.03122900	-0.17807400	0.70167800
H	-5.92964900	0.38166000	0.97394300
H	-5.09643600	4.81159200	-0.00301300
H	-3.82445000	5.28934900	-2.09567700
H	-2.66054000	3.42332600	-3.27156400
C	-3.29330900	1.93337900	-1.85710700
H	-5.17163200	2.52059200	0.90842700

3.4. Mulliken Charge and LUMO of Reactant

The Mulliken charge analysis on the initial reactants bound to $SmI_2(THF)_4$ associated with the reactions of **3x** and **1f** was performed. The results indicate that the bicyclic complex **3x** has a more polarized carbonyl group than **1f** because of a depleted electron density at the carbonyl carbon. Additionally, the LUMO of **Reactant** is slightly lower for **3x** and is primarily distributed at the carbonyl group. These could suggest that **3x** has a greater ability to receive electrons and thus the corresponding SET can proceed more readily.

Figure SI.6. A) Mulliken charges at key atoms of the initial reactant **1f** and **3x**. B) DFT-computed LUMOs with corresponding orbital energies of **1f** and **3x**.

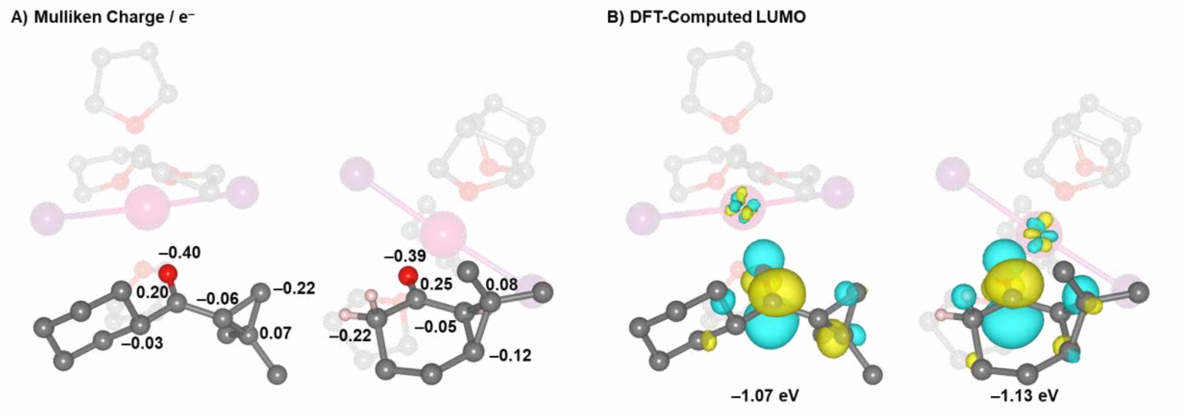

4. Data for X-ray Structures

4.1 4a CCDC 2333540

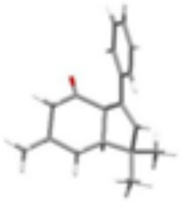

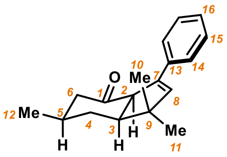

Bond precision:	C-C = 0.0022 A		Wavelength = 1.54184
Cell	a = 5.86667(7)	b = 15.36244(17)	c = 16.41745(19)
	alpha = 90	beta = 90	gamma = 90
Temperature:	200 K		

	**Calculated**	**Reported**
Volume	1479.65(3)	1479.64(3)
Space group	P 21 21 21	P 21 21 21
Hall Group	P 2ac 2ab	P 2ac 2ab
Moiety formula	C18 H22 O	C18 H22 O
Sum formula	C18 H22 O	C18 H22 O
Mr	254.36	254.35
Dx,g cm^{-3}	1.142	1.142
Z	4	4
Mu (mm^{-1})	0.524	0.524
F000	552.0	552.0
h,k,lmax	7,19,20	7,19,20
Nref	3096 [1810	2959
Tmin, Tmax	0.890,0.927	0.868,1.000
Tmin’	0.778	

Correction method= #

Reported T Limits: Tmin=0.868 Tmax=1.000

AbsCorr = MULTI-SCAN

Data completeness= 1.63/0.96

Theta(max)= 75.918

R(reflections)= 0.0327(2836)

wR2(reflections)= 0.0836(2959)

S = 1.058

Npar= 175

4.2 4p CCDC 2333541

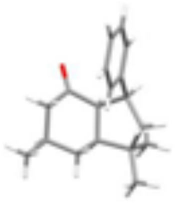

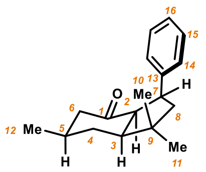

Bond precision:	C-C = 0.0039 A		Wavelength = 1.54184
Cell	a = 6.06768(16)	b=8.3694(2)	c=28.7235(8)
	alpha = 90	beta = 91.304(2)	gamma = 90
Temperature:	100 K		

	**Calculated**	**Reported**
Volume	1458.28(7)	1458.29(7)
Space group	P 21	P 1 21 1
Hall Group	P 2yb	P 2yb
Moiety formula	C18 H24 O	C18 H24 O
Sum formula	C18 H24 O	C18 H24 O
Mr	256.37	256.37
Dx,g cm^{-3}	1.168	1.168
Z	4	4
Mu (mm^{-1})	0.532	0.532
F000	560.0	560.0
F000’	561.44	
h,k,lmax	7,10,36	7,10,34
Nref	6119 [3276]	8321
Tmin, Tmax	0.891,0.924	0.695,1.000
Tmin’	0.809	

Correction method= #

Reported T Limits: Tmin=0.695 Tmax=1.000

AbsCorr = MULTI-SCAN

Data completeness= 2.54/1.36

Theta(max)= 76.311

R(reflections)= 0.0358(8167)

wR2(reflections)= 0.0836(2959)

S = 0.1041(8321)

Npar= 350

5. References

(1) Szostak, M.; Spain, M.; Procter, D. J. Preparation of Samarium(II) Iodide: Quantitative Evaluation of the Effect of Water, Oxygen, and Peroxide Content, Preparative Methods, and the Activation of Samarium Metal. *J. Org. Chem.* **2012**, *77* (7), 3049–3059. https://doi.org/10.1021/jo300135v.

(2) Dahlén, A.; Hilmersson, G. Microwave-Assisted Generation of Lanthanide(II) Halides in THF and Simple Quantitative Determination. *Eur. J. Inorg. Chem.* **2004**, *2004* (15), 3020–3024. https://doi.org/10.1002/ejic.200400309.

(3) Schulte, A. M.; Alachouzos, G.; Szymański, W.; Feringa, B. L. Strategy for Engineering High Photolysis Efficiency of Photocleavable Protecting Groups through Cation Stabilization. *J. Am. Chem. Soc.* **2022**, *144* (27), 12421–12430. https://doi.org/10.1021/jacs.2c04262.

(4) Hodgson, D. M.; Chung, Y. K.; Paris, J.-M. Intramolecular Cyclopropanation of Unsaturated Terminal Epoxides. *J. Am. Chem. Soc.* **2004**, *126* (28), 8664–8665. https://doi.org/10.1021/ja047346k.

(5) Pietruszka, J.; Witt, A. Synthesis of the Bestmann-Ohira Reagent. *Synthesis* **2006**, 4266–4268. https://doi.org/10.1055/s-2006-950307.

(6) Agasti, S.; Beattie, N. A.; McDouall, J. J. W.; Procter, D. J. SmI2-Catalyzed Intermolecular Coupling of Cyclopropyl Ketones and Alkynes: A Link between Ketone Conformation and Reactivity. *J. Am. Chem. Soc.* **2021**, *143* (9), 3655–3661. https://doi.org/10.1021/jacs.1c01356.

(7) Hamada, Y.; Kawasaki-Takasuka, T.; Yamazaki, T. Base-Promoted Isomerization of CF3-Containing Allylic Alcohols to the Corresponding Saturated Ketones under Metal-Free Conditions. *Beilstein J. Org. Chem.* **2017**, *13* (1), 1507–1512. https://doi.org/10.3762/bjoc.13.149.

(8) Antoniak, D.; Barbasiewicz, M. Corey–Chaykovsky Cyclopropanation of Nitronaphthalenes: Access to Benzonorcaradienes and Related Systems. *Org. Lett.* **2019**, *21* (23), 9320–9325. https://doi.org/10.1021/acs.orglett.9b03375.

(9) Salahi, F.; Yao, C.; Norton, J. R.; Snyder, S. A. The Synthesis of Diverse Terpene Architectures from Phenols. *Nat. Synth.* **2022**, *1* (4), 313–321. https://doi.org/10.1038/s44160-022-00051-2.

(10) Dutta, U.; Lupton, D. W.; Maiti, D. Aryl Nitriles from Alkynes Using Tert-Butyl Nitrite: Metal-Free Approach to C≡C Bond Cleavage. *Org. Lett.* **2016**, *18* (4), 860–863. https://doi.org/10.1021/acs.orglett.6b00147.

(11) Ahmad, S.; Choudhury, S.; Khan, F. A. Synthesis of Marine Brominated Alkaloid Amathamide F: A Palladium-Catalyzed Enamide Synthesis. *Tetrahedron* **2015**, *71* (24), 4192–4202. https://doi.org/10.1016/j.tet.2015.04.091.

(12) Roy, S.; Khatua, H.; Das, S. K.; Chattopadhyay, B. Iron(II)-Based Metalloradical Activation: Switch from Traditional Click Chemistry to Denitrogenative Annulation. *Angew. Chem. Int. Ed.* **2019**, *58* (33), 11439–11443. https://doi.org/10.1002/anie.201904702.

(13) Kolb, B.; Silva Dos Santos, D.; Krause, S.; Zens, A.; Laschat, S. Sequential Hydrozirconation/Pd-Catalyzed Cross Coupling of Acyl Chlorides towards Conjugated (2 *E* ,4 *E*)-Dienones. *Beilstein J. Org. Chem.* **2023**, *19*, 176–185. https://doi.org/10.3762/bjoc.19.17.

(14) Miyaura, N.; Suginome, H.; Suzuki, A. Stereospecific Synthesis of (2Z,4E,6E)-3,7,11-Trimethyl-2,4,6,10-Dodecatetraene [Trans(C10)-Allofarnesene]. *Bull. Chem. Soc. Jpn.* **1982**, *55* (7), 2221–2223. https://doi.org/10.1246/bcsj.55.2221.

(15) Ozaki, T.; Kobayashi, Y. Exploration of Aryllithium-Derived Copper Reagents for Quaternary-Stereogenic-Center-Forming Allylic Substitution of γ,γ-Disubstituted Secondary Allylic Picolinates. *Synlett* **2016**, *27* (04), 611–615. https://doi.org/10.1055/s-0035-1560907.

(16) Usuda, H.; Kuramochi, A.; Kanai, M.; Shibasaki, M. Challenge toward Structural Complexity Using Asymmetric Catalysis: Target-Oriented Development of Catalytic Enantioselective Diels−Alder Reaction. *Org. Lett.* **2004**, *6* (23), 4387–4390. https://doi.org/10.1021/ol048018s.

(17) Rasson, C.; Stouse, A.; Boreux, A.; Cirriez, V.; Riant, O. Copper-Catalyzed One-Pot Borylative Aldolisation β-Fluoride Elimination for the Formal Addition of Acrylates to Carbonyl Moieties. *Chem. – Eur. J.* **2018**, *24* (37), 9234–9237. https://doi.org/10.1002/chem.201802023.

(18) Charette, A. B.; Lebel, H. Diastereoselective Cyclopropanation of Chiral Allylic Alcohols: A More Efficient Reagent for the Relative Stereocontrol. *J. Org. Chem.* **1995**, *60* (10), 2966–2967. https://doi.org/10.1021/jo00115a008.

(19) Malkov, A. V.; Pernazza, D.; Bell, M.; Bella, M.; Massa, A.; Teplý, F.; Meghani, P.; Kočovský, P. Synthesis of New Chiral 2,2‘-Bipyridine Ligands and Their Application in Copper-Catalyzed Asymmetric Allylic Oxidation and Cyclopropanation. *J. Org. Chem.* **2003**, *68* (12), 4727–4742. https://doi.org/10.1021/jo034179i.

(20) Kolehmainen, E.; Laihia, K.; Heinänen, M.; Rissanen, K.; Fröhlich, R.; Korvola, J.; Mänttäri, P.; Kauppinen, R. Oxygen-Containing Bicyclic Monoterpenes. 1H, 13C and 17O NMR Spectroscopic and X-Ray Diffraction Studies of Seven Oxidation Products of (+)-3-Carene. *J. Chem. Soc. Perkin Trans. 2* **1993**, No. 4, 641–648. https://doi.org/10.1039/P29930000641.
(21) Corey, E. J.; Jautelat, Manfred. Construction of Ring Systems Containing the Gemdimethylcyclopropane Unit Using Diphenylsulfonium Isopropylide. *J. Am. Chem. Soc.* **1967**, *89* (15), 3912–3914. https://doi.org/10.1021/ja00991a050.
(22) Jirgensons, A.; Kauss, V.; Kalvinsh, I.; Gold, M. R.; Danysz, W.; Parsons, C. G.; Quack, G. Synthesis and Structure–Affinity Relationships of 1,3,5-Alkylsubstituted Cyclohexylamines Binding at NMDA Receptor PCP Site. *Eur. J. Med. Chem.* **2000**, *35* (6), 555–565. https://doi.org/10.1016/S0223-5234(00)00153-7.
(23) Gottumukkala, A. L.; Suljagic, J.; Matcha, K.; de Vries, J. G.; Minnaard, A. J. Efficient Formation of Benzylic Quaternary Centers via Palladium Catalysis. *ChemSusChem* **2013**, *6* (9), 1636–1639. https://doi.org/10.1002/cssc.201300276.
(24) Agasti, S.; Beltran, F.; Pye, E.; Kaltsoyannis, N.; Crisenza, G. E. M.; Procter, D. J. A Catalytic Alkene Insertion Approach to Bicyclo[2.1.1]Hexane Bioisosteres. *Nat. Chem.* **2023**, *15* (4), 535–541. https://doi.org/10.1038/s41557-023-01135-y.
(25) Morrill, C.; Péter, Á.; Amalina, I.; Pye, E.; Crisenza, G. E. M.; Kaltsoyannis, N.; Procter, D. J. Diastereoselective Radical 1,4-Ester Migration: Radical Cyclizations of Acyclic Esters with SmI2. *J. Am. Chem. Soc.* **2022**, *144* (30), 13946–13952. https://doi.org/10.1021/jacs.2c05972.
(26) Werner, D.; Zhao, X.; Best, S. P.; Maron, L.; Junk, P. C.; Deacon, G. B. Bulky Ytterbium Formamidinates Stabilise Complexes with Radical Ligands, and Related Samarium "Tetracyclone" Chemistry. *Chem. – Eur. J.* **2017**, *23* (9), 2084–2102. https://doi.org/10.1002/chem.201604203.
(27) Pantazis, D. A.; Neese, F. All-Electron Scalar Relativistic Basis Sets for the Lanthanides. *J. Chem. Theory Comput.* **2009**, *5* (9), 2229–2238. https://doi.org/10.1021/ct900090f.
(28) Campos, C. T.; Jorge, F. E. Triple Zeta Quality Basis Sets for Atoms Rb through Xe: Application in CCSD(T) Atomic and Molecular Property Calculations. *Mol. Phys.* **2013**, *111* (2), 167–173. https://doi.org/10.1080/00268976.2012.709282.
(29) Gaussian 16, Revision C.01, Frisch, M. J.; Trucks, G. W.; Schlegel, H. B.; Scuseria, G. E.; Robb, M. A.; Cheeseman, J. R.; Scalmani, G.; Barone, V.; Petersson, G. A.; Nakatsuji, H.; Li, X.; Caricato, M.; Marenich, A. V.; Bloino, J.; Janesko, B. G.; Gomperts, R.; Mennucci, B.; Hratchian, H. P.; Ortiz, J. V.; Izmaylov, A. F.; Sonnenberg, J. L.; Williams-Young, D.; Ding, F.; Lipparini, F.; Egidi, F.; Goings, J.; Peng, B.; Petrone, A.; Henderson, T.; Ranasinghe, D.; Zakrzewski, V. G.; Gao, J.; Rega, N.; Zheng, G.; Liang, W.; Hada, M.; Ehara, M.; Toyota, K.; Fukuda, R.; Hasegawa, J.; Ishida, M.; Nakajima, T.; Honda, Y.; Kitao, O.; Nakai, H.; Vreven, T.; Throssell, K.; Montgomery, J. A., Jr.; Peralta, J. E.; Ogliaro, F.; Bearpark, M. J.; Heyd, J. J.; Brothers, E. N.; Kudin, K. N.; Staroverov, V. N.; Keith, T. A.; Kobayashi, R.; Normand, J.; Raghavachari, K.; Rendell, A. P.; Burant, J. C.; Iyengar, S. S.; Tomasi, J.; Cossi, M.; Millam, J. M.; Klene, M.; Adamo, C.; Cammi, R.; Ochterski, J. W.; Martin, R. L.; Morokuma, K.; Farkas, O.; Foresman, J. B.; Fox, D. J. Gaussian, Inc., Wallingford CT, 2016.
(30) Adamo, C.; Barone, V. Toward Reliable Density Functional Methods without Adjustable Parameters: The PBE0 Model. *J. Chem. Phys.* **1999**, *110* (13), 6158–6170. https://doi.org/10.1063/1.478522.
(31) Dunning, T. H., Jr. Gaussian Basis Sets for Use in Correlated Molecular Calculations. I. The Atoms Boron through Neon and Hydrogen. *J. Chem. Phys.* **1989**, *90* (2), 1007–1023. https://doi.org/10.1063/1.456153.
(32) Martin, J. M. L.; Sundermann, A. Correlation Consistent Valence Basis Sets for Use with the Stuttgart–Dresden–Bonn Relativistic Effective Core Potentials: The Atoms Ga–Kr and In–Xe. *J. Chem. Phys.* **2001**, *114* (8), 3408–3420. https://doi.org/10.1063/1.1337864.
(33) Cao, X.; Dolg, M. Segmented Contraction Scheme for Small-Core Lanthanide Pseudopotential Basis Sets. *J. Mol. Struct. THEOCHEM* **2002**, *581* (1), 139–147. https://doi.org/10.1016/S0166-1280(01)00751-5.
(34) Grimme, S.; Antony, J.; Ehrlich, S.; Krieg, H. A Consistent and Accurate Ab Initio Parametrization of Density Functional Dispersion Correction (DFT-D) for the 94 Elements H-Pu. *J. Chem. Phys.* **2010**, *132* (15), 154104. https://doi.org/10.1063/1.3382344.
(35) Johnson, E. R.; Becke, A. D. A Post-Hartree-Fock Model of Intermolecular Interactions: Inclusion of Higher-Order Corrections. *J. Chem. Phys.* **2006**, *124* (17), 174104. https://doi.org/10.1063/1.2190220.

(36) Visscher, L.; Dyall, K. G. DIRAC–FOCK ATOMIC ELECTRONIC STRUCTURE CALCULATIONS USING DIFFERENT NUCLEAR CHARGE DISTRIBUTIONS. *At. Data Nucl. Data Tables* **1997**, *67* (2), 207–224. https://doi.org/10.1006/adnd.1997.0751.
(37) Tomasi, J.; Mennucci, B.; Cammi, R. Quantum Mechanical Continuum Solvation Models. *Chem. Rev.* **2005**, *105* (8), 2999–3094. https://doi.org/10.1021/cr9904009.
(38) Grimme, S. Supramolecular Binding Thermodynamics by Dispersion-Corrected Density Functional Theory. *Chem. – Eur. J.* **2012**, *18* (32), 9955–9964. https://doi.org/10.1002/chem.201200497.
(39) Luchini, G.; Alegre-Requena, J. V.; Funes-Ardoiz, I.; Paton, R. S. GoodVibes: Automated Thermochemistry for Heterogeneous Computational Chemistry Data. F1000Research April 24, 2020. https://doi.org/10.12688/f1000research.22758.1.

6. ^{1}H, ^{13}C{H}, ^{19}F, and ^{31}P NMR Spectra

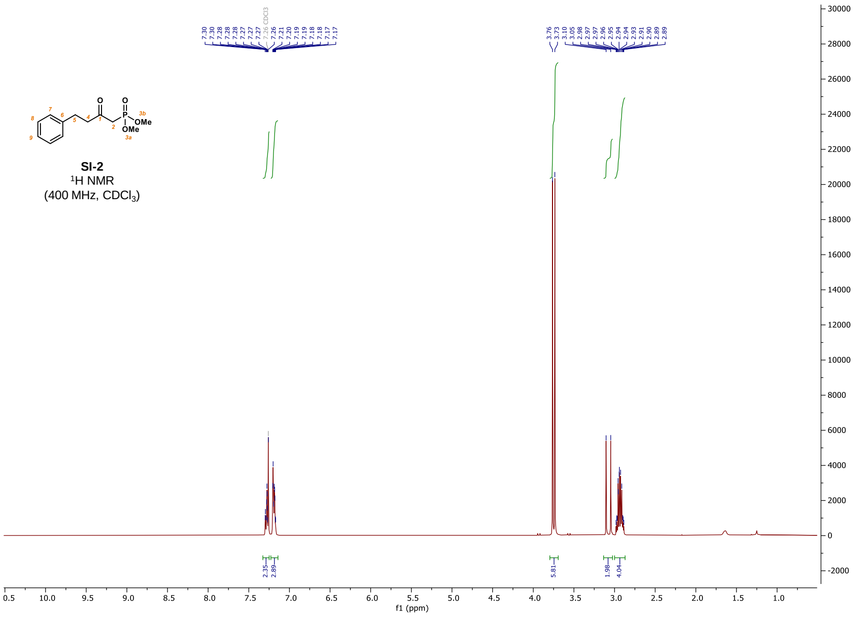

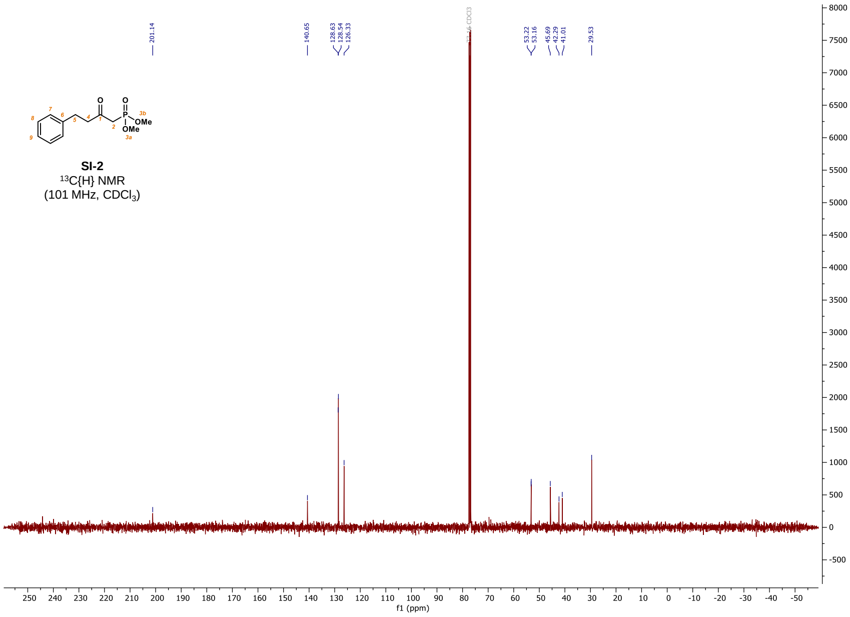
201.14
140.65
128.63
128.54
126.33
53.22
53.16
45.69
42.29
41.01
29.53
SI-2
13C{H} NMR
(101 MHz, CDCl3)
f1 (ppm)

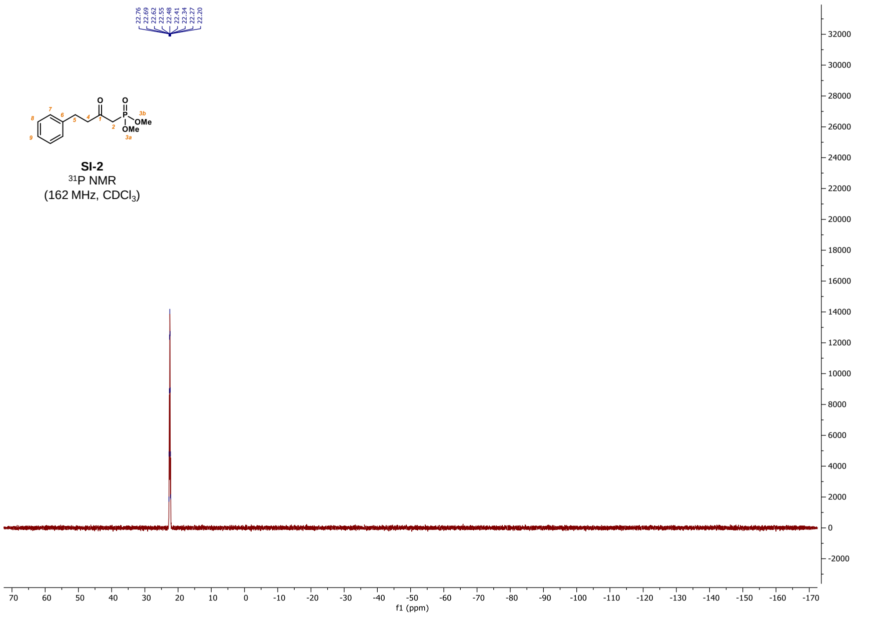
22.76
22.69
22.62
22.55
22.48
22.41
22.34
22.27
22.20
SI-2
31P NMR
(162 MHz, CDCl3)
f1 (ppm)

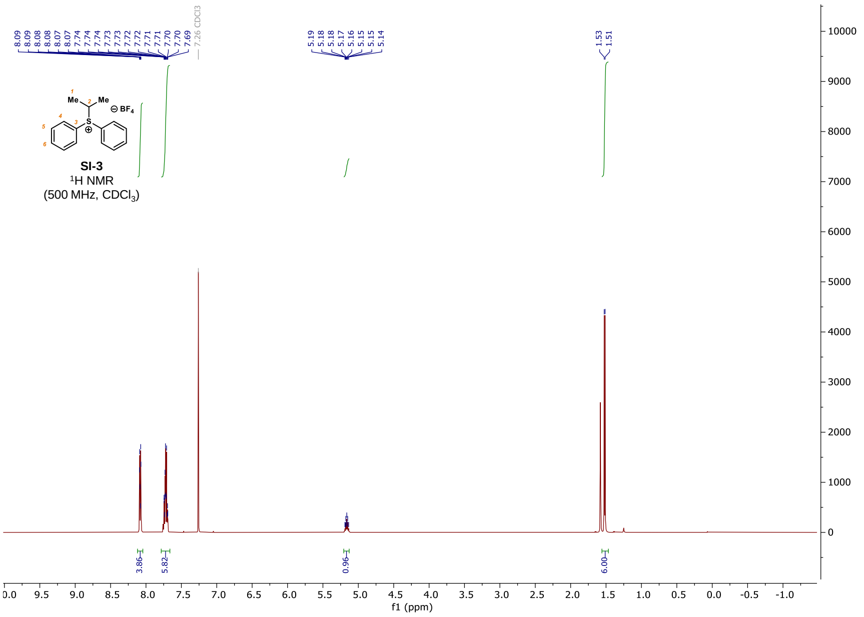
SI-3
1H NMR
(500 MHz, CDCl3)
f1 (ppm)

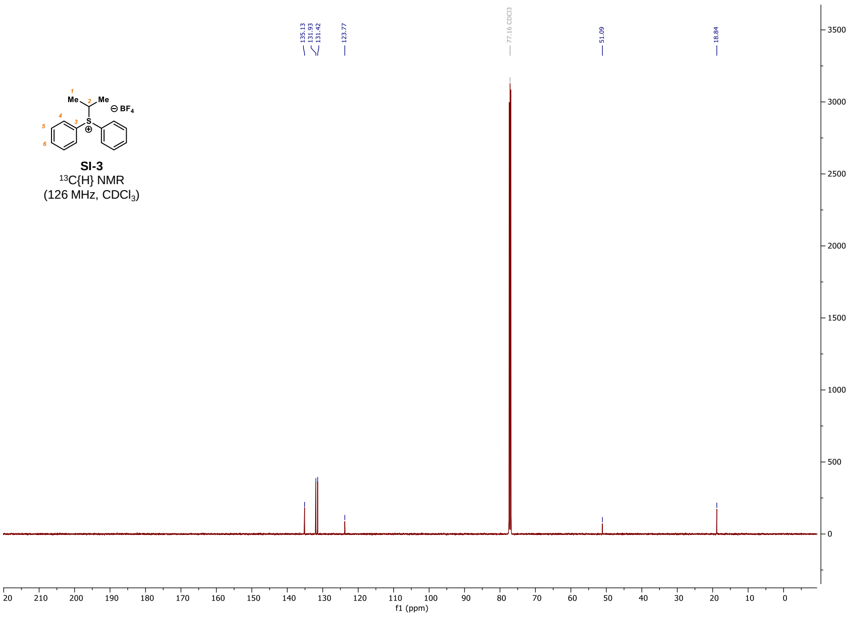
SI-3
13C{H} NMR
(126 MHz, CDCl3)
f1 (ppm)

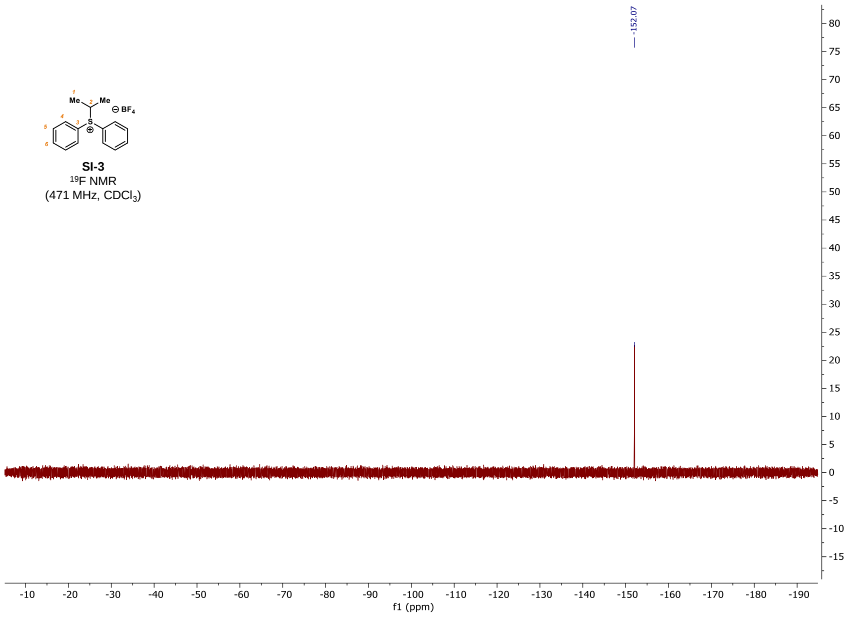
-152.07
Me
Me
BF4
S
SI-3
19F NMR
(471 MHz, CDCl3)
f1 (ppm)

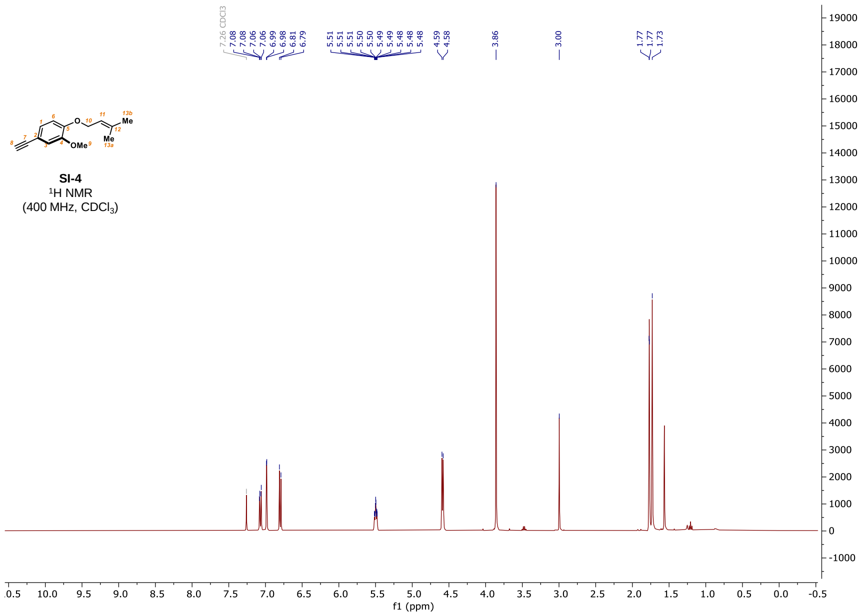
7.26 CDCl3
7.08
7.08
7.06
7.06
6.99
6.98
6.81
6.79
5.51
5.51
5.51
5.50
5.50
5.49
5.49
5.48
5.48
5.48
4.59
4.58
3.86
3.00
1.77
1.77
1.73
O
Me
Me
OMe
SI-4
1H NMR
(400 MHz, CDCl3)
f1 (ppm)

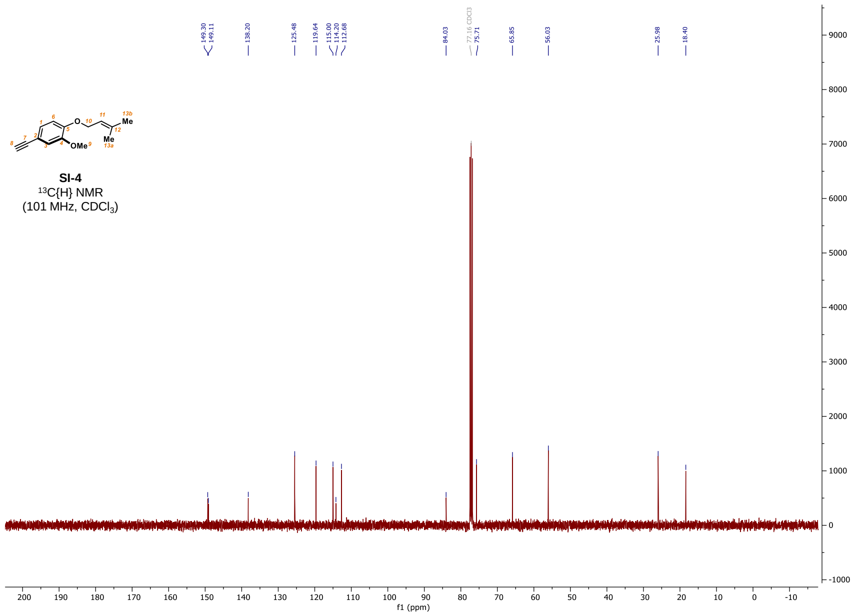

SI-4
^{13}C{H} NMR
(101 MHz, CDCl$_3$)

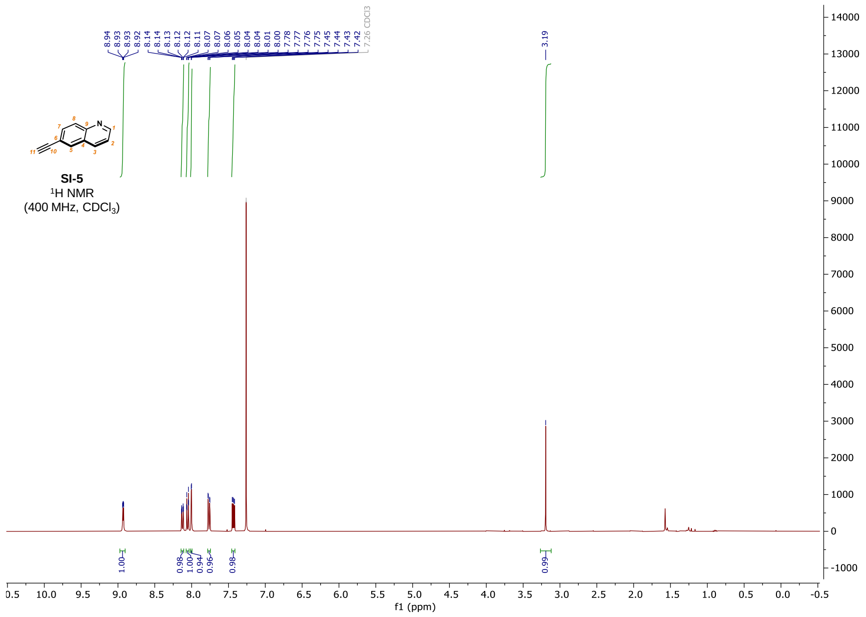
8.94
8.93
8.93
8.92
8.14
8.14
8.13
8.12
8.12
8.11
8.07
8.07
8.06
8.05
8.04
8.04
8.01
8.00
7.78
7.77
7.76
7.75
7.45
7.44
7.43
7.42
7.26 CDCl3
3.19
SI-5
1H NMR
(400 MHz, CDCl3)
1.00
0.98
1.00
0.94
0.96
0.98
0.99
f1 (ppm)

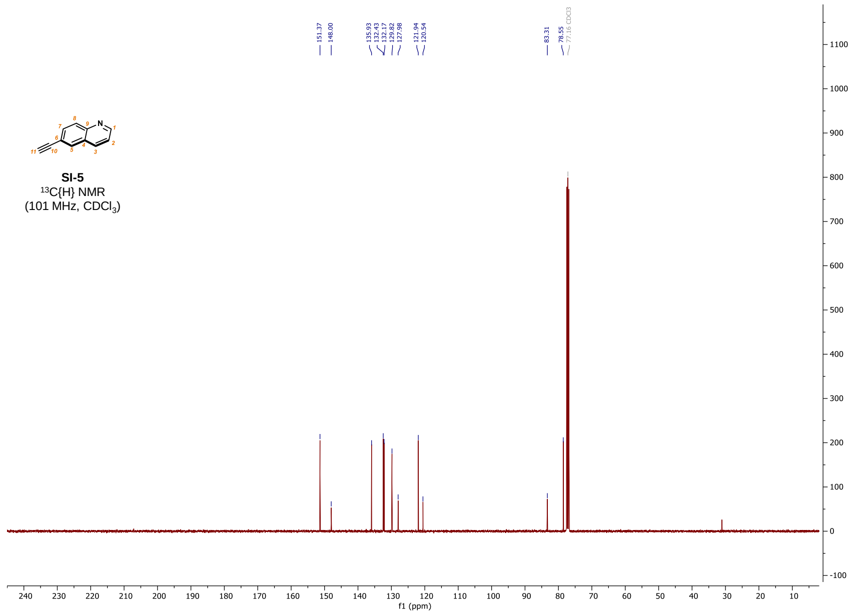
151.37
148.00
135.93
132.43
132.17
129.82
127.98
121.94
120.54
83.31
78.55
77.16 CDCl3
SI-5
13C{H} NMR
(101 MHz, CDCl3)
f1 (ppm)

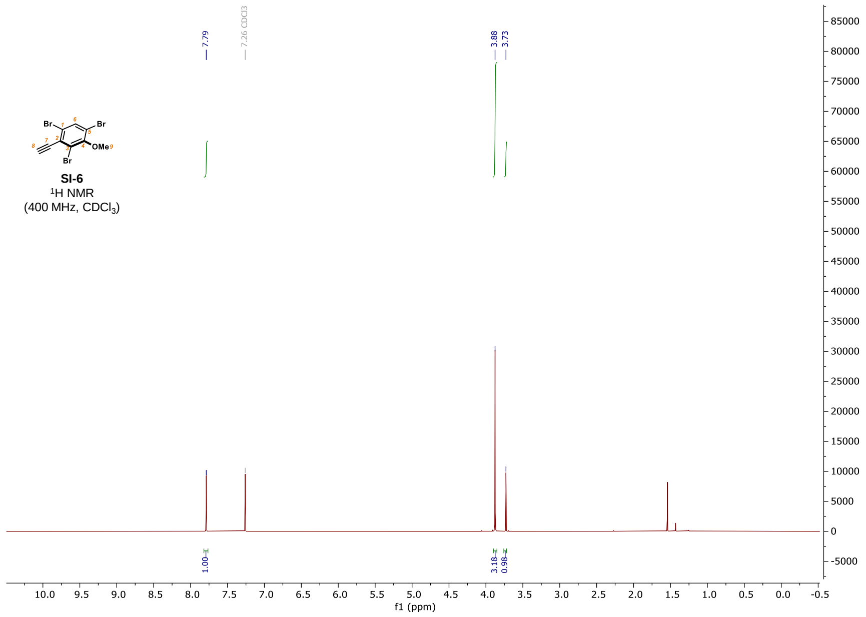
Br
Br
OMe
Br
SI-6
¹H NMR
(400 MHz, CDCl₃)
7.79
7.26 CDCl3
3.88
3.73
1.00
3.18
0.98
f1 (ppm)

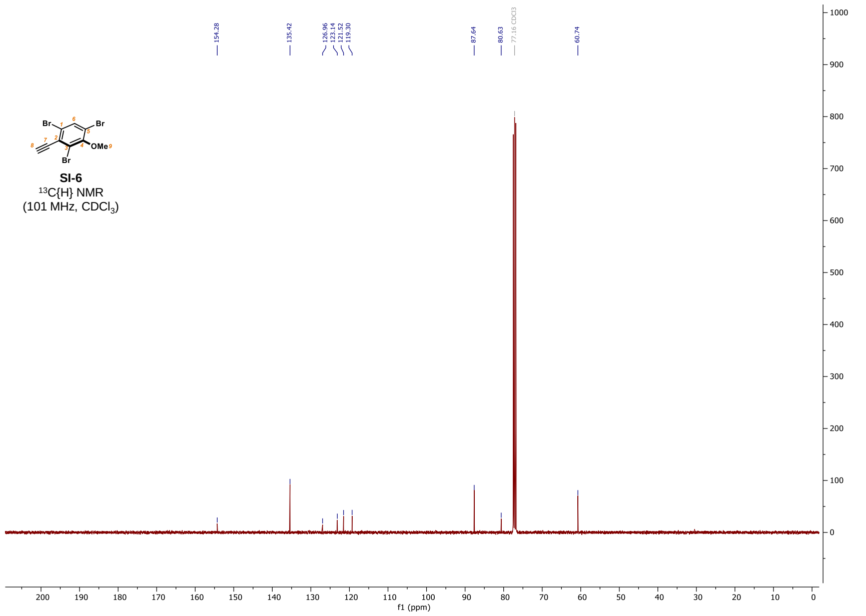
Br
Br
OMe
Br
SI-6
¹³C{H} NMR
(101 MHz, CDCl₃)
154.28
135.42
126.96
123.14
121.52
119.30
87.64
80.63
77.16 CDCl3
60.74
f1 (ppm)

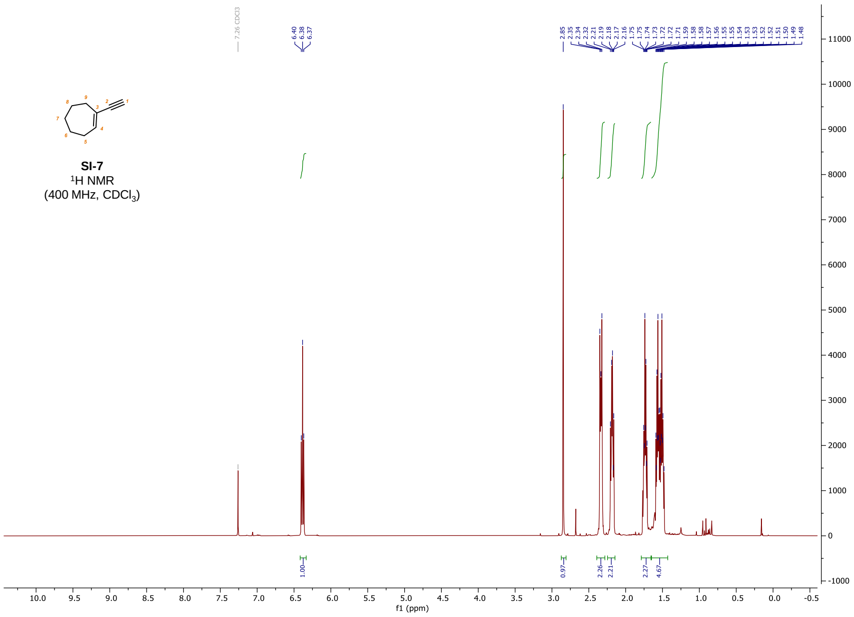
SI-7
^{1}H NMR
(400 MHz, $CDCl_3$)
f1 (ppm)

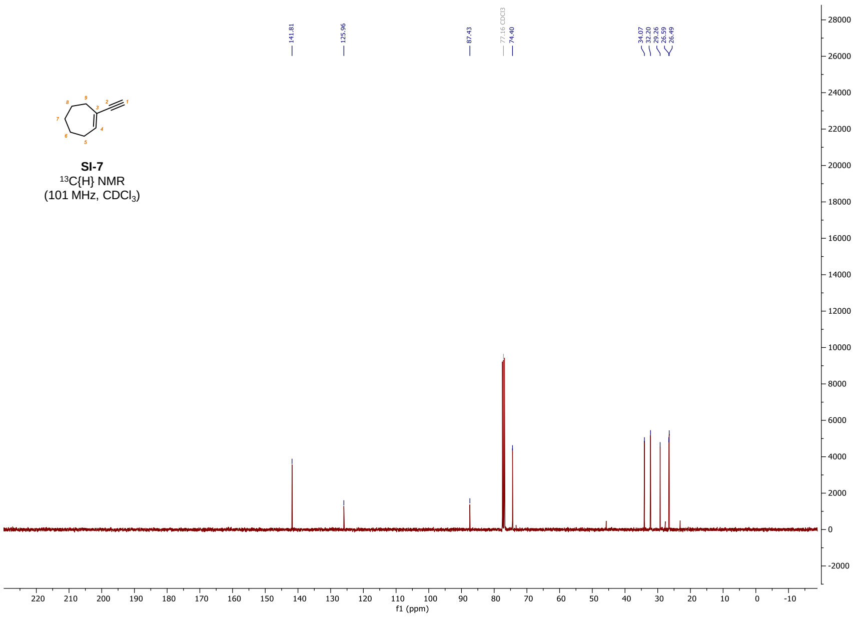
SI-7
^{13}C{H} NMR
(101 MHz, $CDCl_3$)
f1 (ppm)

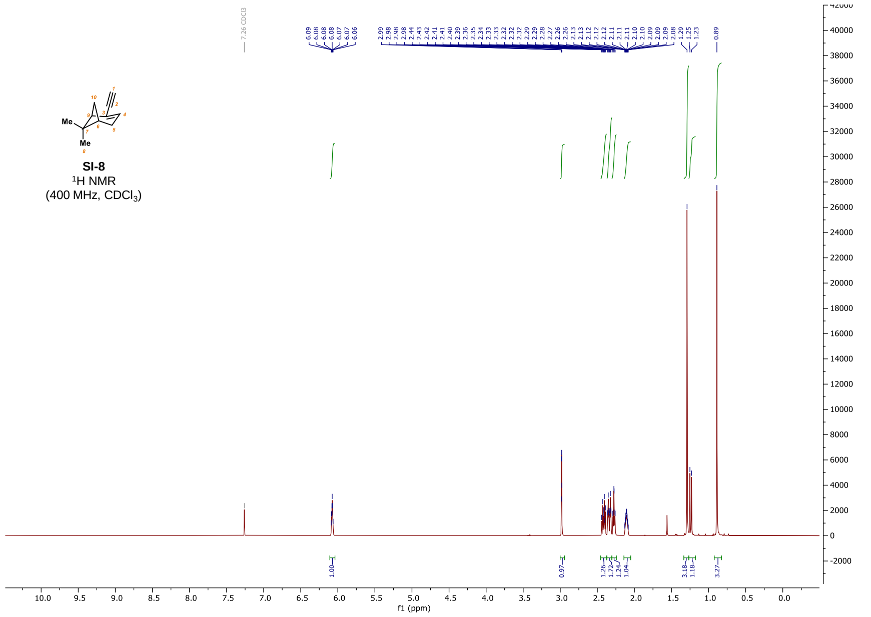
SI-8
^{1}H NMR
(400 MHz, $CDCl_3$)
f1 (ppm)

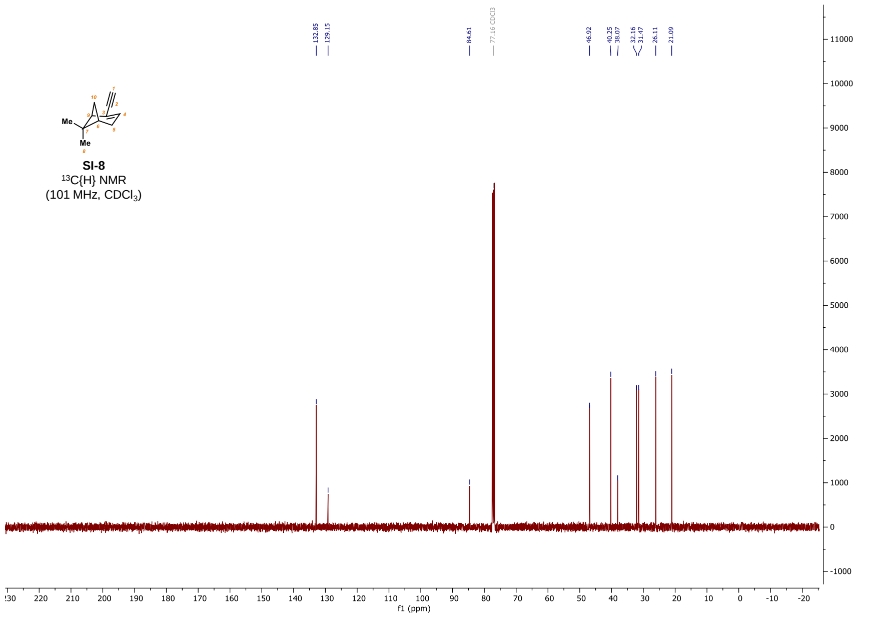
SI-8
^{13}C{H} NMR
(101 MHz, $CDCl_3$)
f1 (ppm)

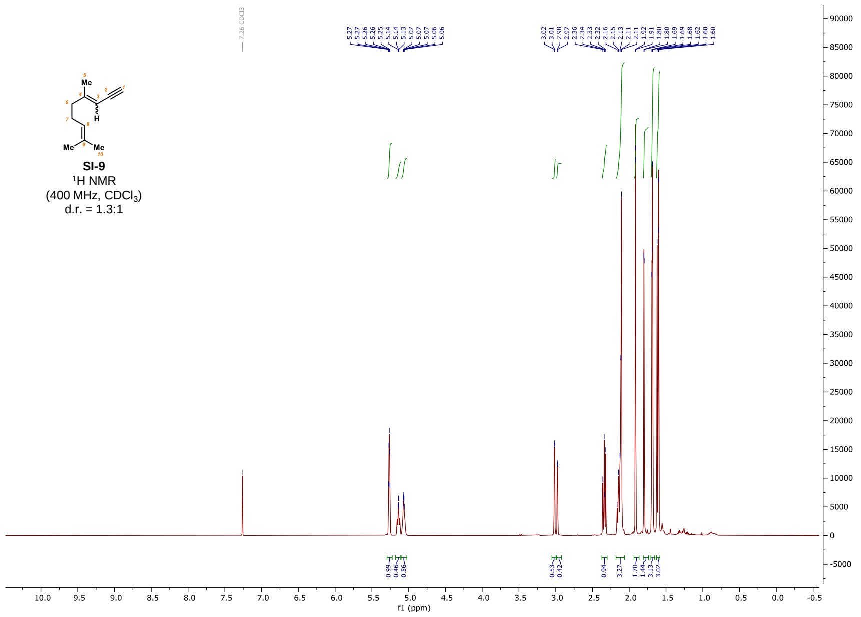
SI-9
^{1}H NMR
(400 MHz, $CDCl_3$)
d.r. = 1.3:1
f1 (ppm)

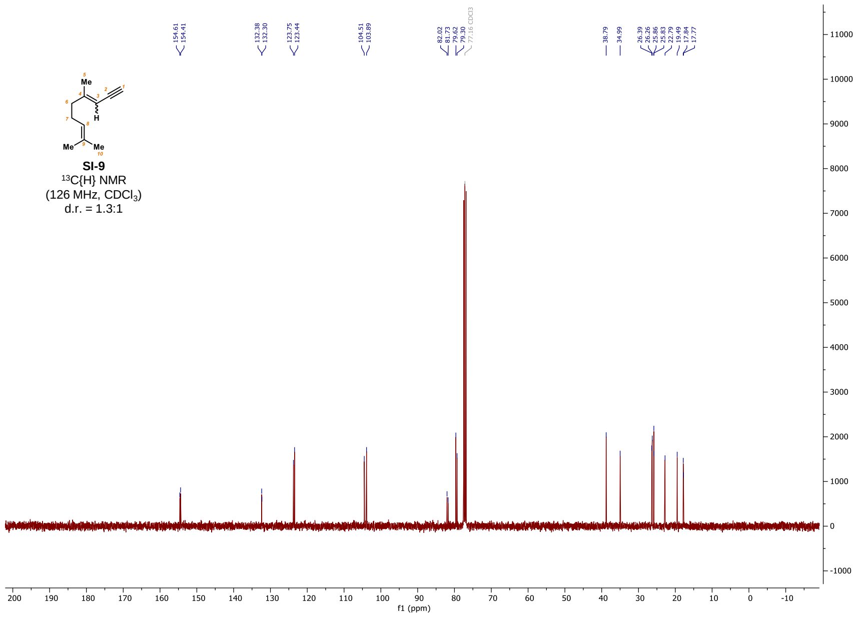
SI-9
^{13}C{H} NMR
(126 MHz, $CDCl_3$)
d.r. = 1.3:1
f1 (ppm)

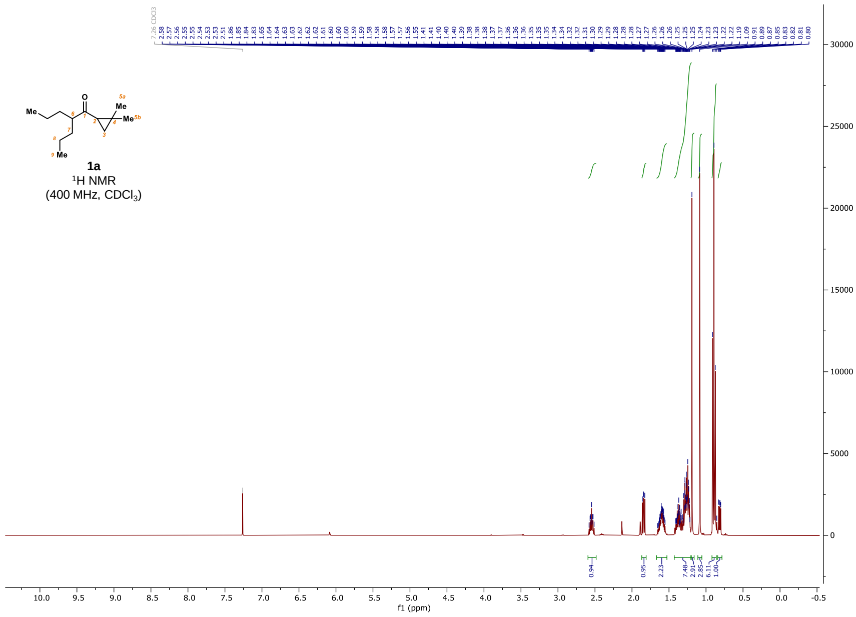

1a
^{1}H NMR
(400 MHz, $CDCl_3$)

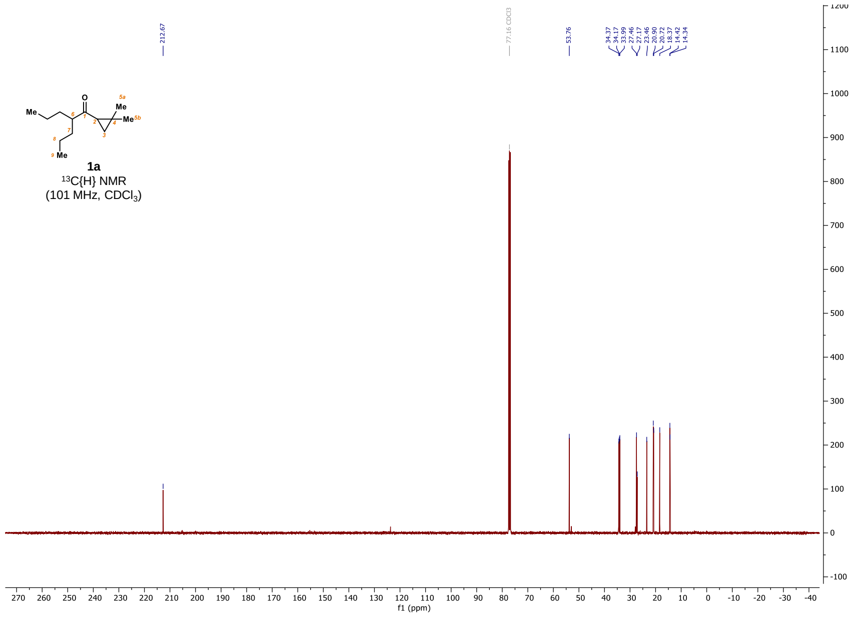

1a
$^{13}C\{H\}$ NMR
(101 MHz, $CDCl_3$)

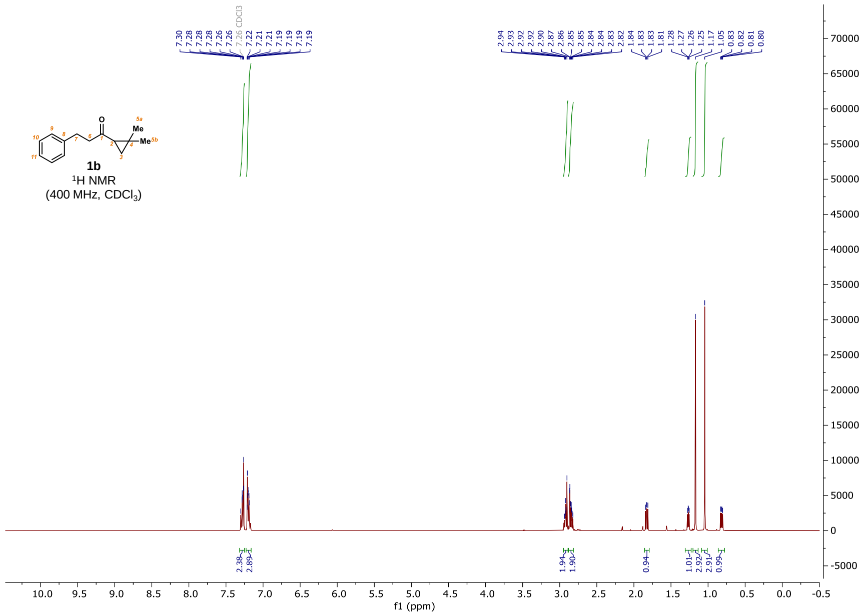
1b
¹H NMR
(400 MHz, CDCl3)
f1 (ppm)

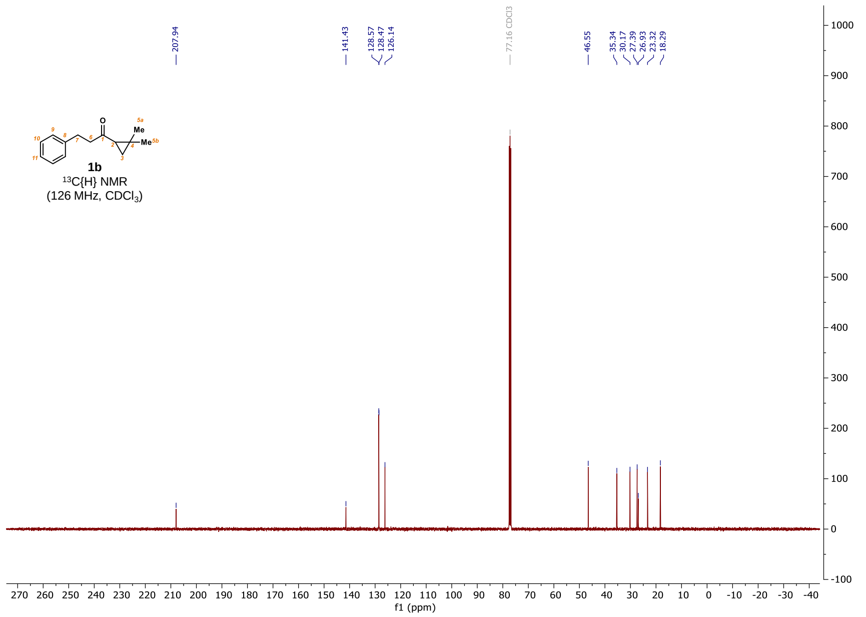
1b
¹³C{H} NMR
(126 MHz, CDCl3)
f1 (ppm)

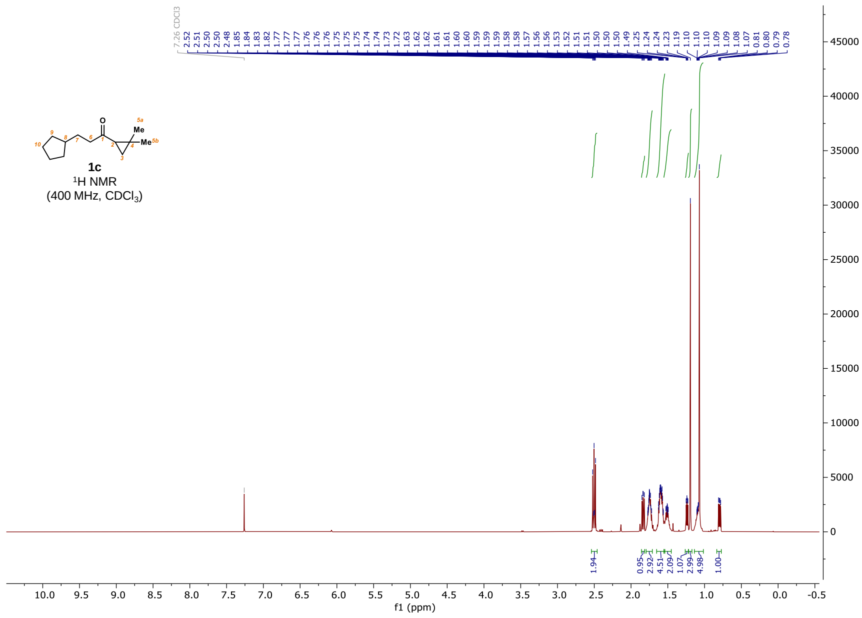
1c
¹H NMR
(400 MHz, CDCl3)
7.26 CDCl3
f1 (ppm)

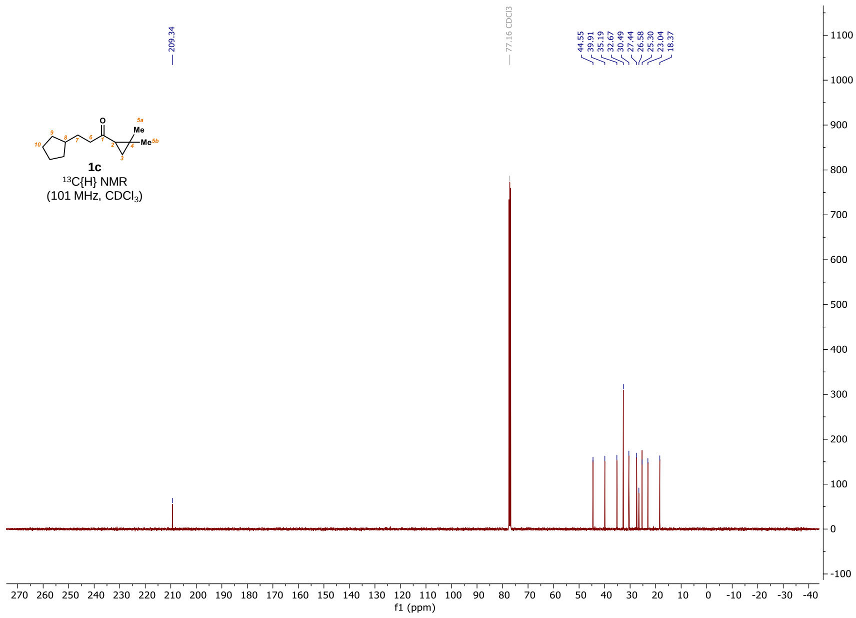
1c
¹³C{H} NMR
(101 MHz, CDCl3)
209.34
77.16 CDCl3
44.55
39.91
35.19
32.67
30.49
27.44
26.58
25.30
23.04
18.37
f1 (ppm)

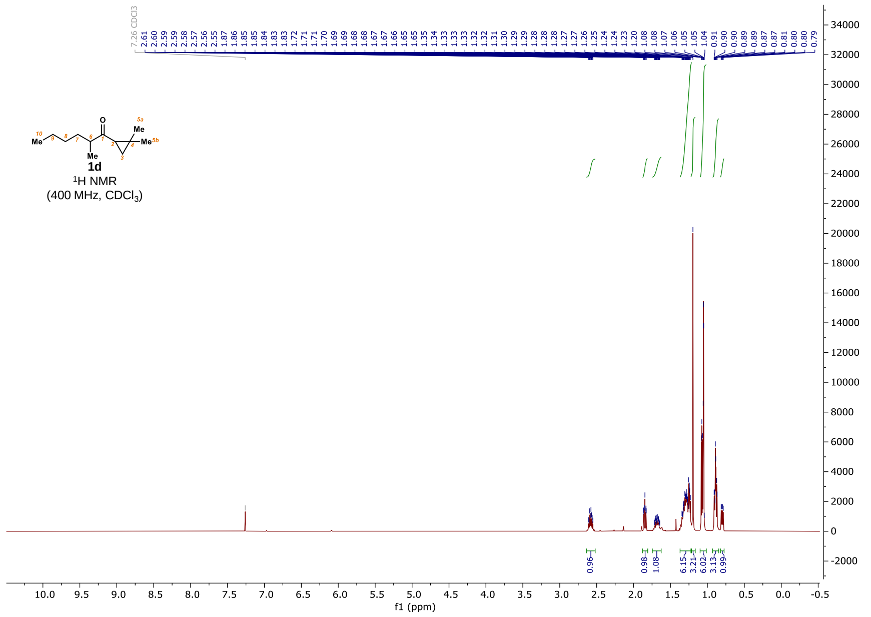

1d
^{1}H NMR
(400 MHz, $CDCl_3$)
f1 (ppm)

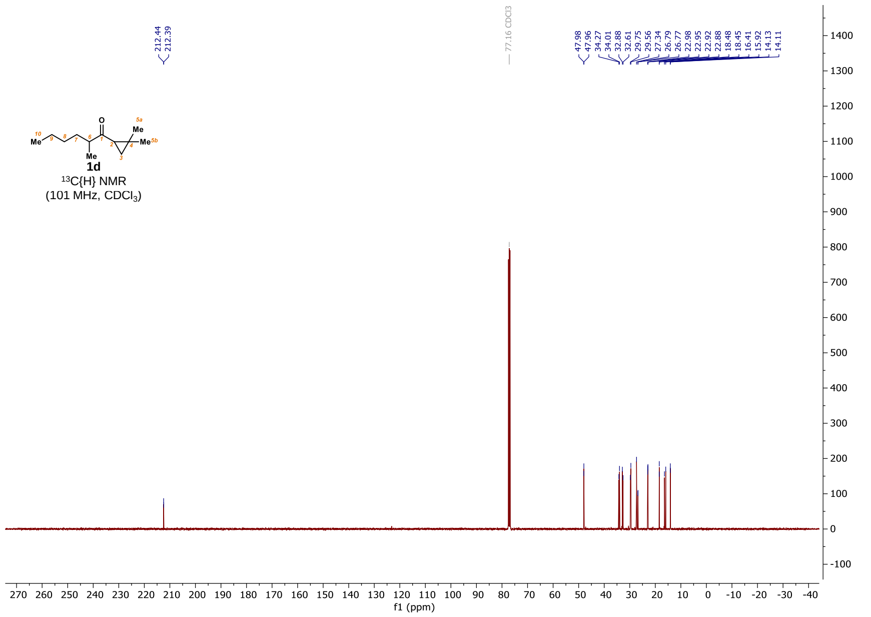

1d
^{13}C{H} NMR
(101 MHz, $CDCl_3$)
f1 (ppm)

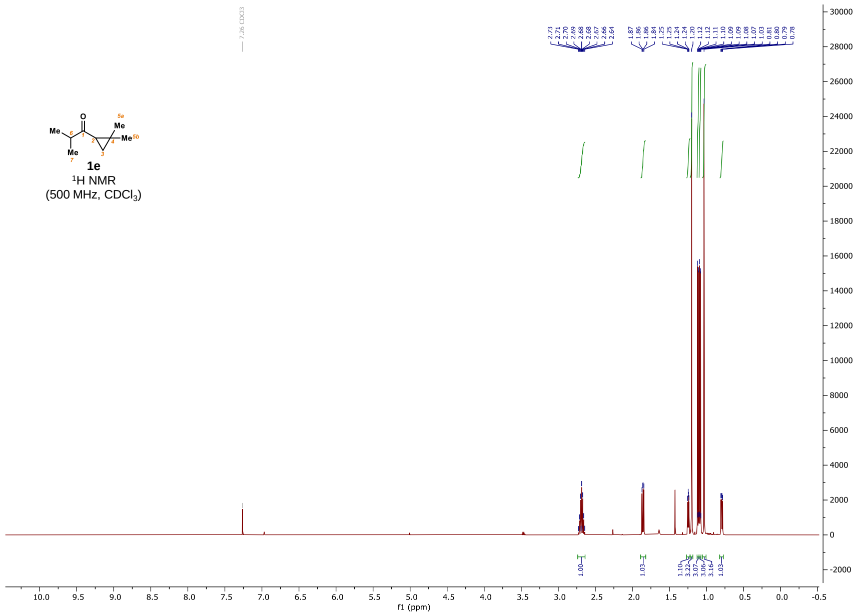
1e
^{1}H NMR
(500 MHz, CDCl$_3$)
f1 (ppm)

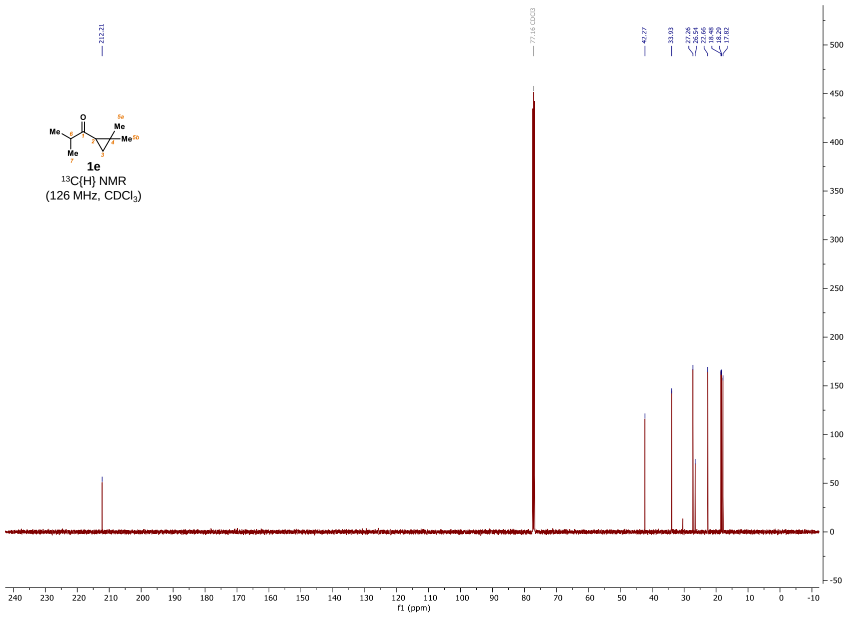
1e
^{13}C{H} NMR
(126 MHz, CDCl$_3$)
f1 (ppm)

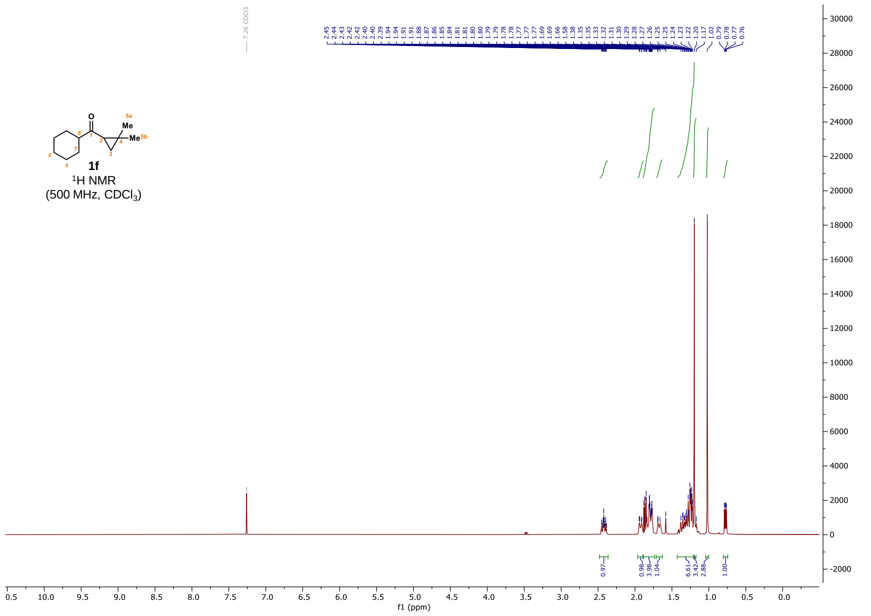
1f
^{1}H NMR
(500 MHz, $CDCl_3$)
f1 (ppm)

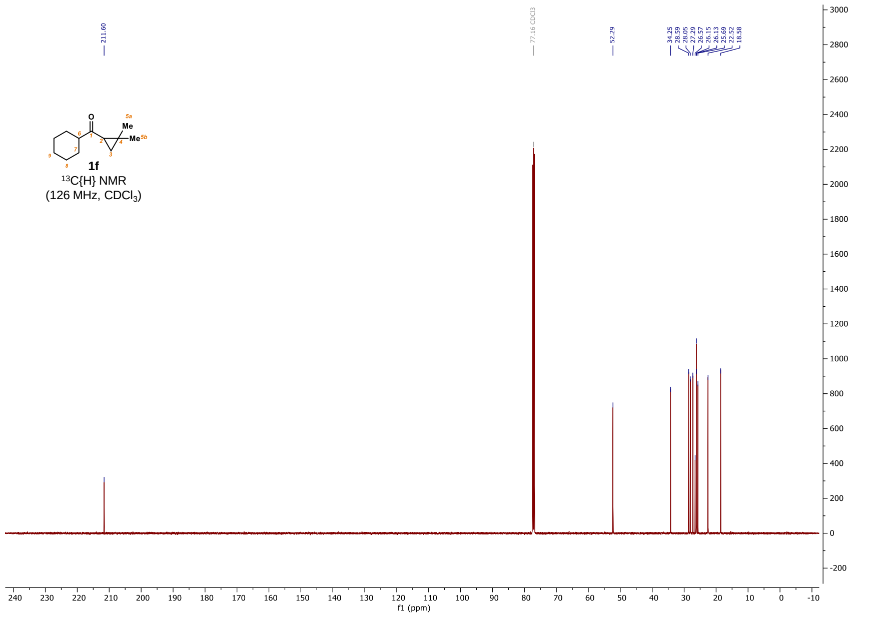
1f
^{13}C{H} NMR
(126 MHz, $CDCl_3$)
f1 (ppm)

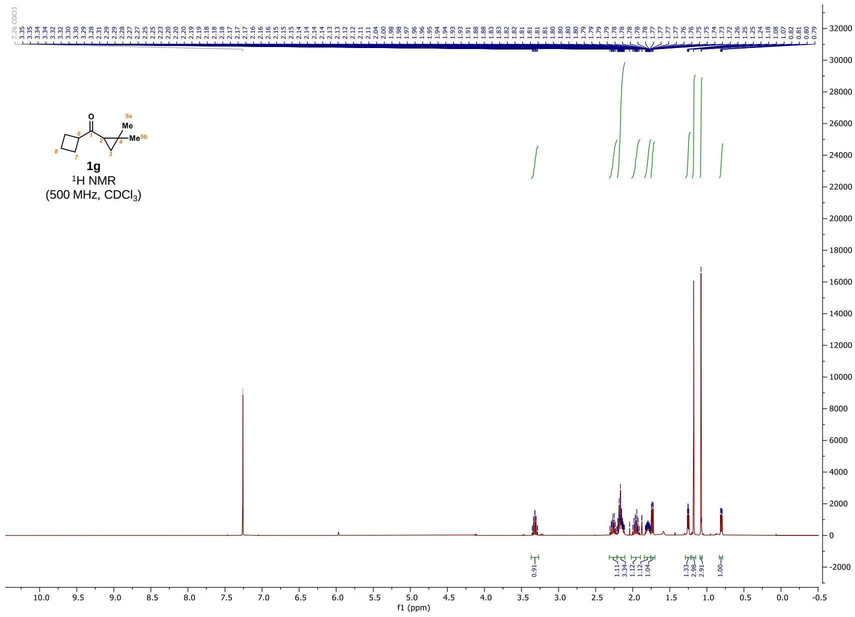
1g
^{1}H NMR
(500 MHz, $CDCl_3$)
f1 (ppm)

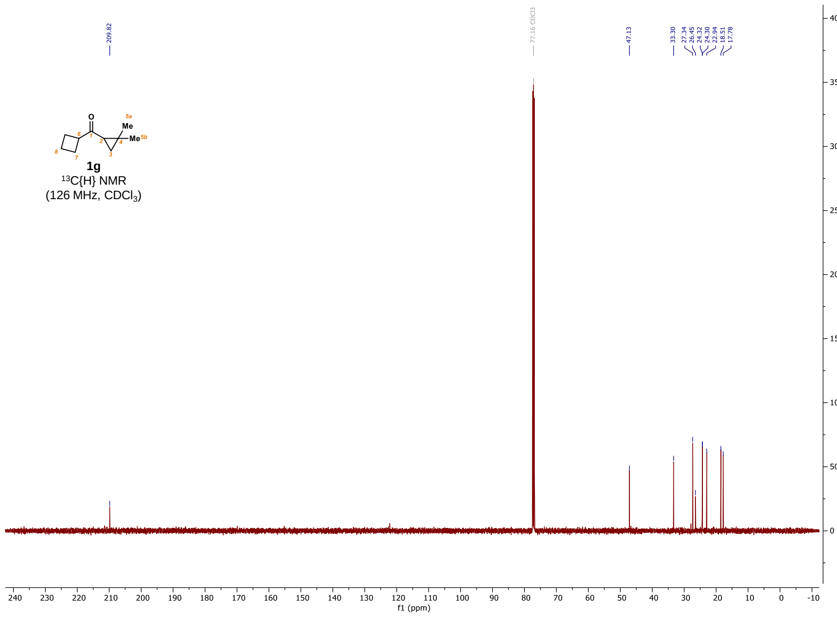
209.82
77.16 CDCl3
47.13
33.30
27.34
26.45
24.32
24.30
22.94
18.51
17.78
1g
^{13}C{H} NMR
(126 MHz, $CDCl_3$)
f1 (ppm)

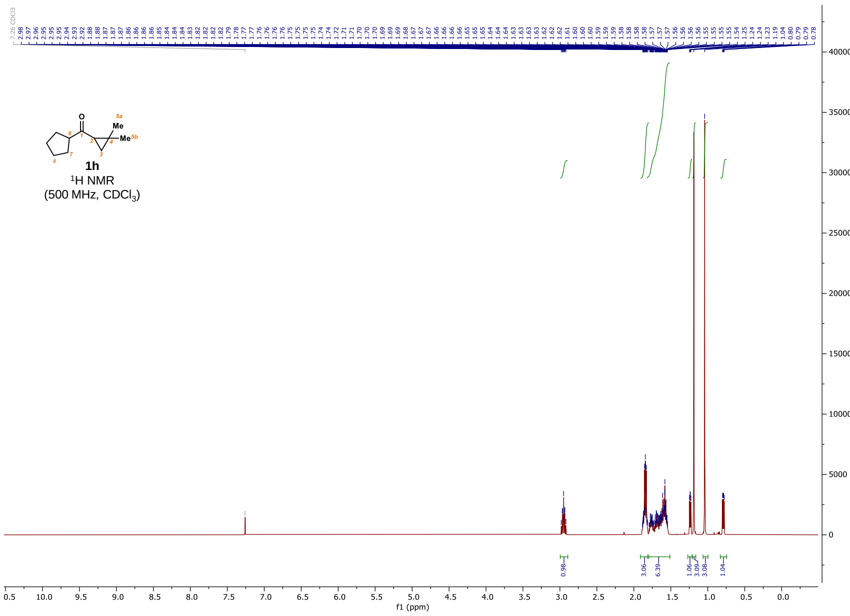
1h
1H NMR
(500 MHz, CDCl3)
f1 (ppm)

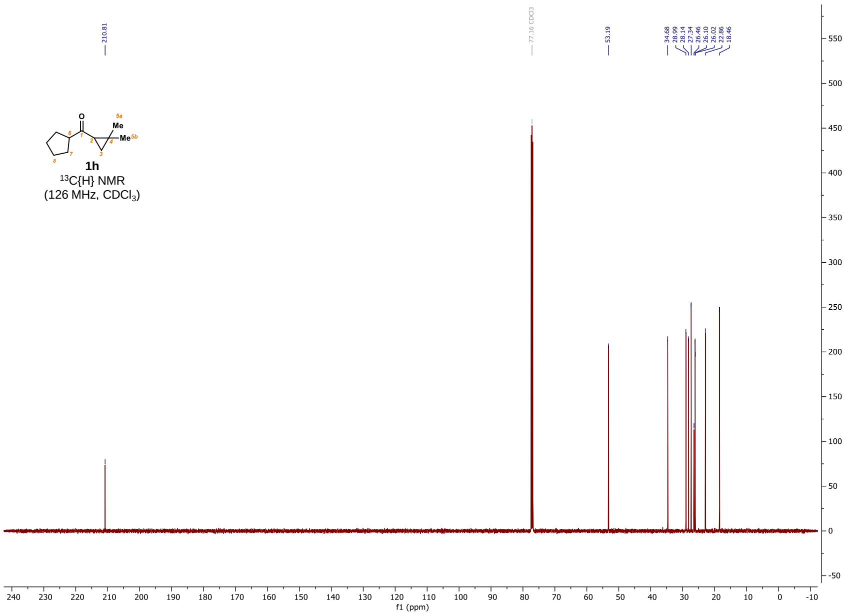
210.81
77.16 CDCl3
53.19
1h
13C{H} NMR
(126 MHz, CDCl3)
f1 (ppm)

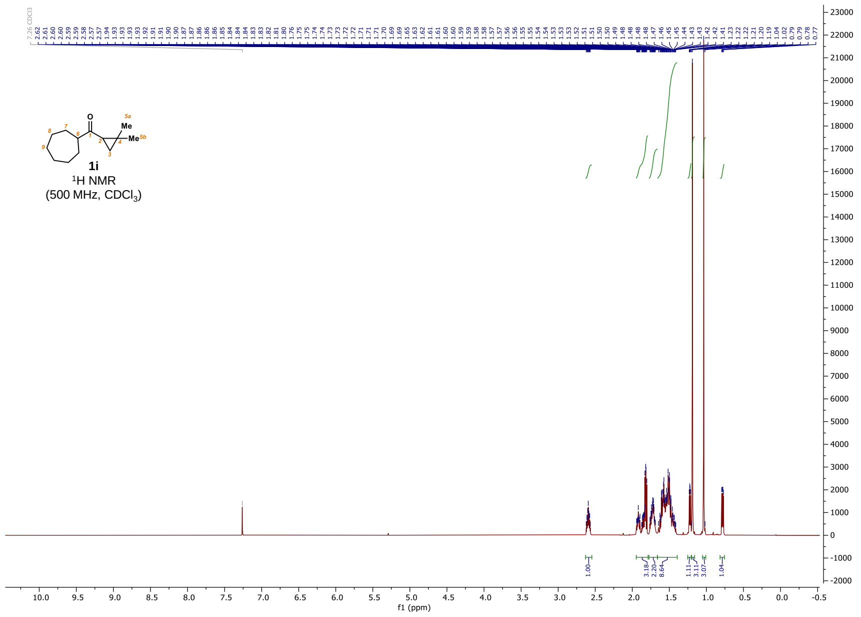
1i
^{1}H NMR
(500 MHz, CDCl$_3$)
f1 (ppm)

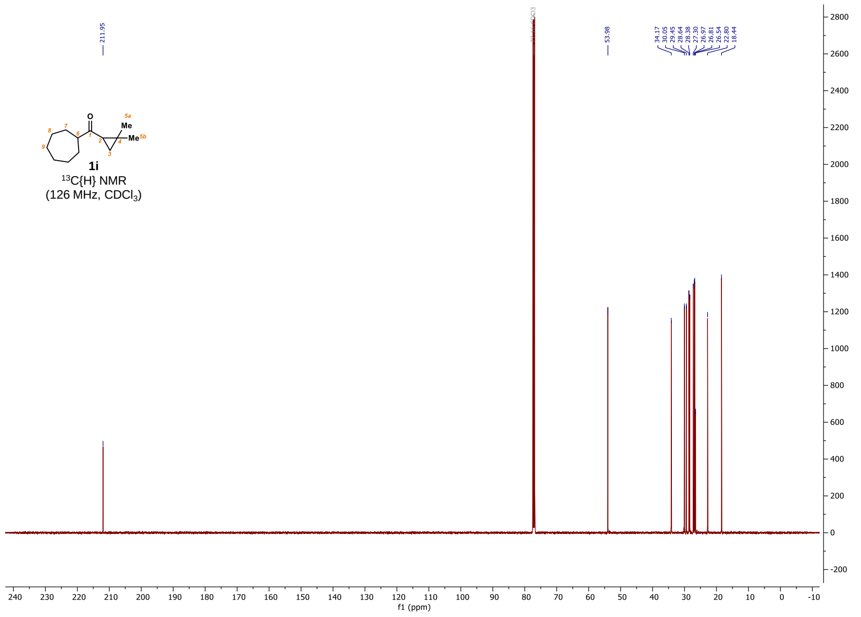
1i
^{13}C{H} NMR
(126 MHz, CDCl$_3$)
211.95
53.98
34.17
30.05
29.45
28.64
28.38
27.30
26.97
26.81
26.54
22.80
18.44
f1 (ppm)

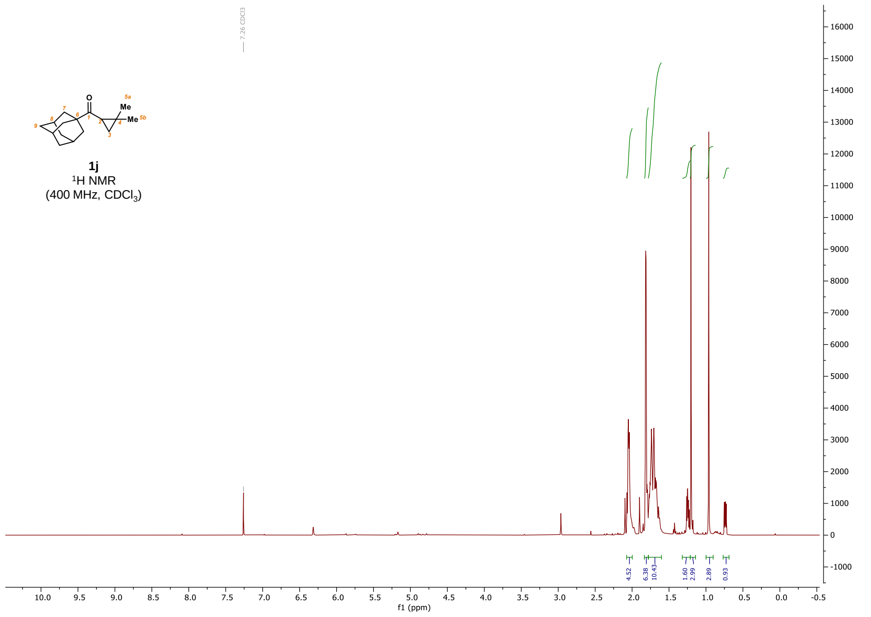

7.26 CDCl3
Me
Me
O
1j
^{1}H NMR
(400 MHz, $CDCl_3$)
4.52
6.38
10.43
1.60
2.99
2.89
0.93
f1 (ppm)

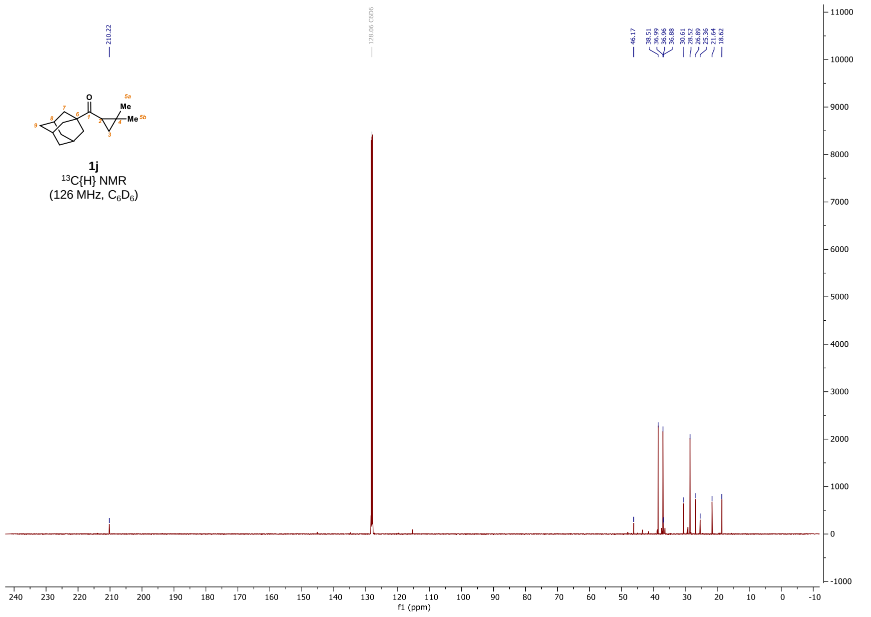

210.22
128.06 C6D6
46.17
38.51
36.99
36.96
36.88
30.61
28.52
26.89
25.36
21.64
18.62
Me
Me
O
1j
^{13}C{H} NMR
(126 MHz, C_6D_6)
f1 (ppm)

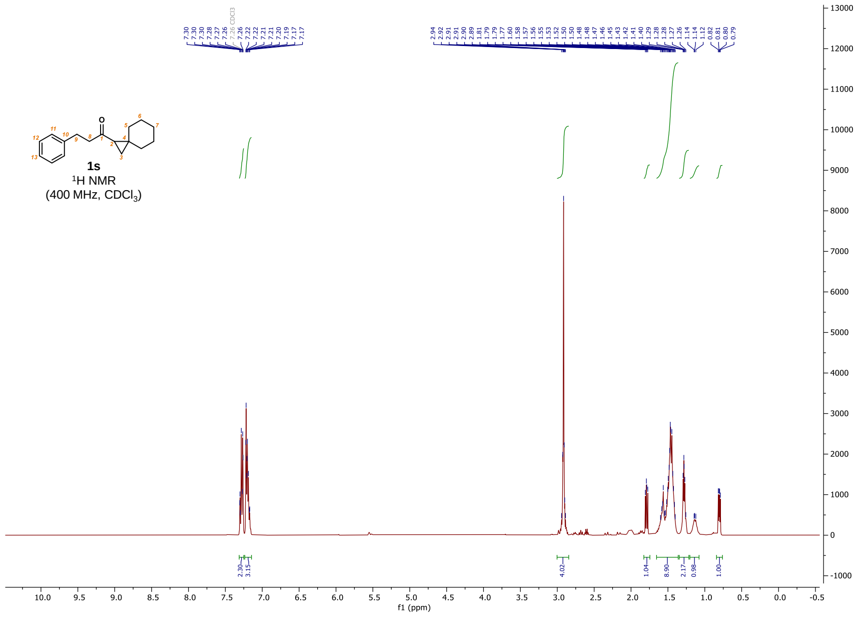
1s
1H NMR
(400 MHz, CDCl3)
f1 (ppm)

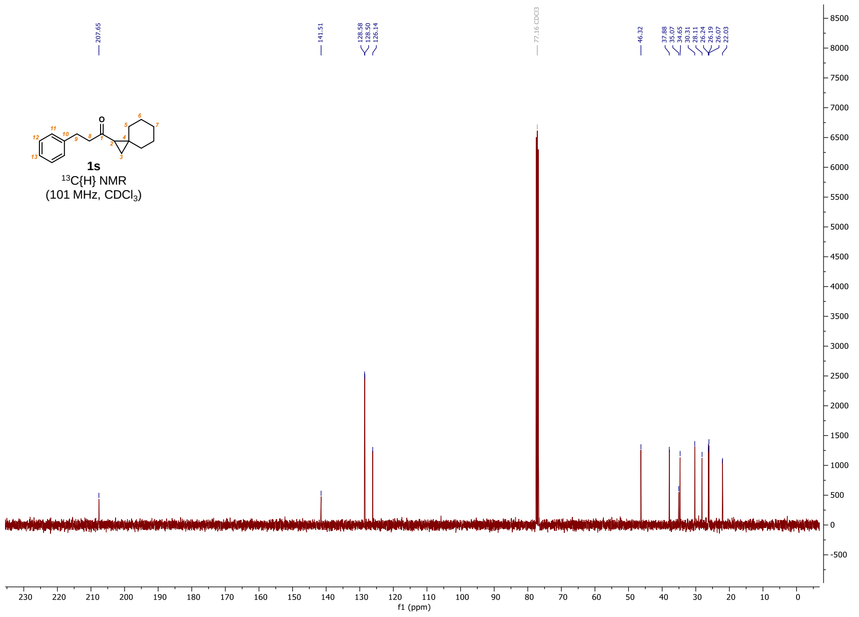
1s
13C{H} NMR
(101 MHz, CDCl3)
f1 (ppm)

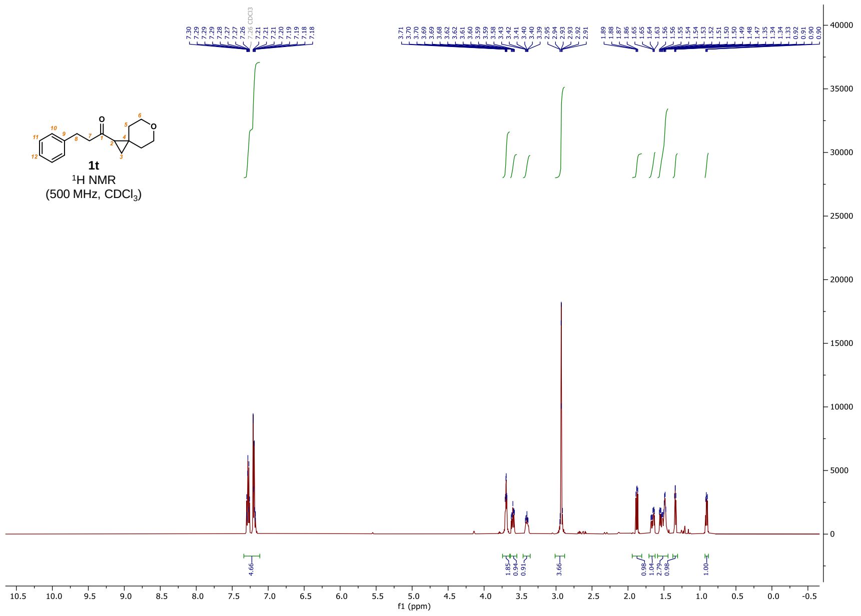

1t
^{1}H NMR
(500 MHz, $CDCl_3$)

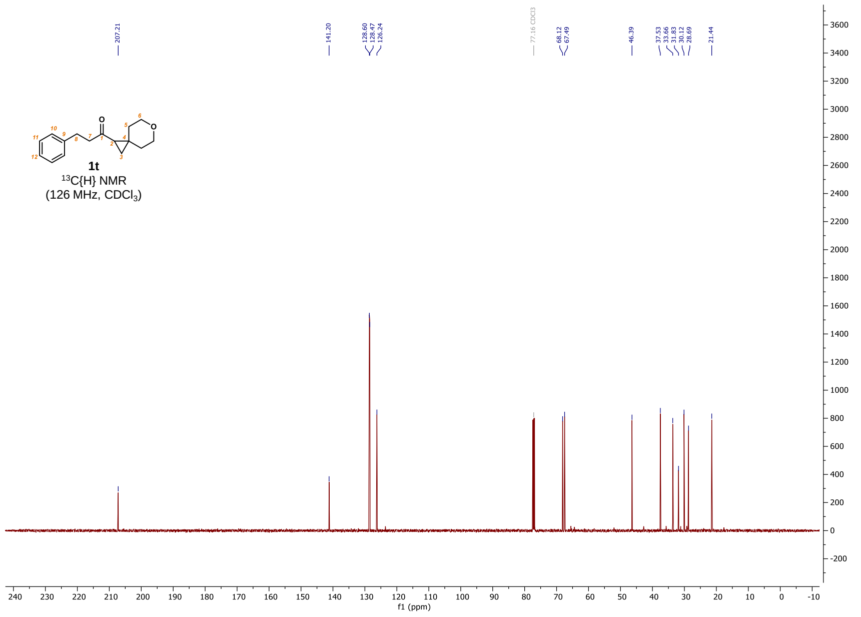

1t
^{13}C{H} NMR
(126 MHz, $CDCl_3$)

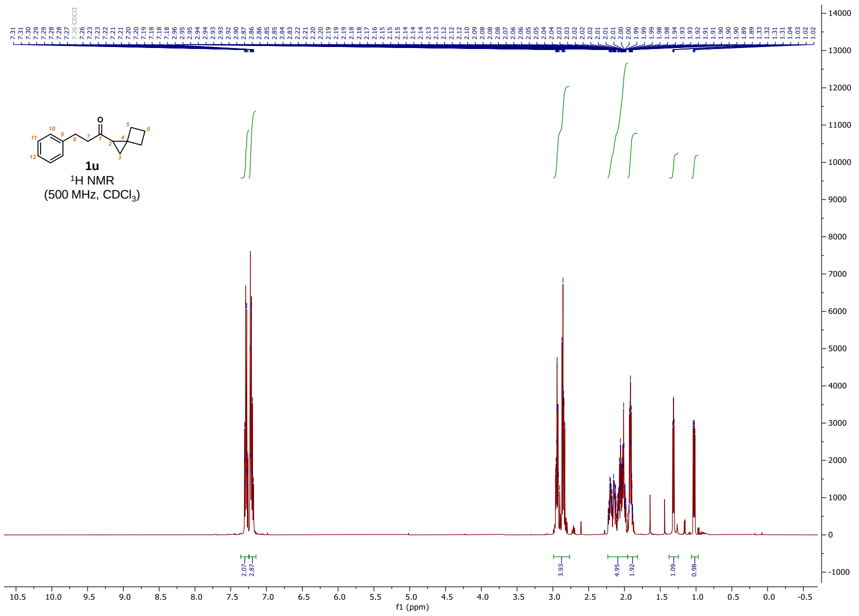
1u
1H NMR
(500 MHz, CDCl3)
f1 (ppm)

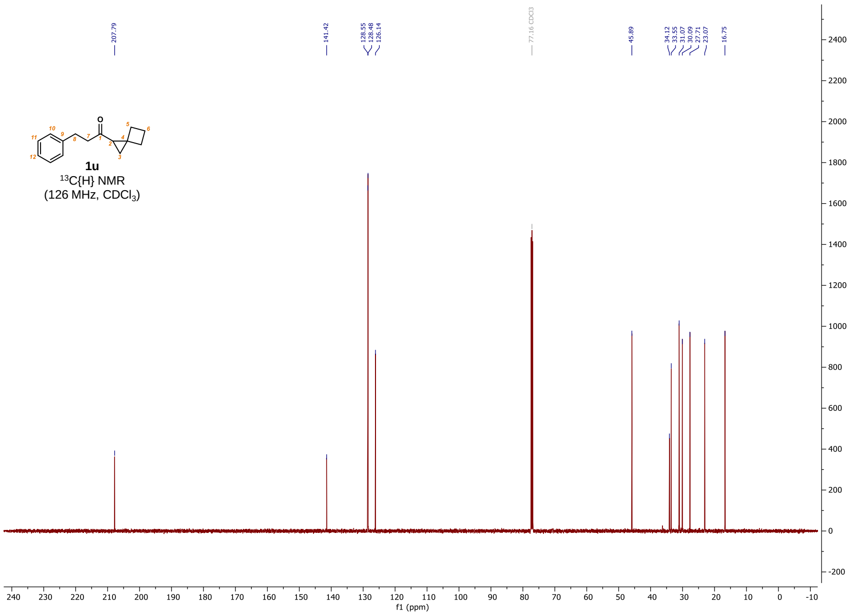
207.79
141.42
128.55
128.48
126.14
77.16 CDCl3
45.89
34.12
33.55
31.07
30.09
27.71
23.07
16.75
1u
13C{H} NMR
(126 MHz, CDCl3)
f1 (ppm)

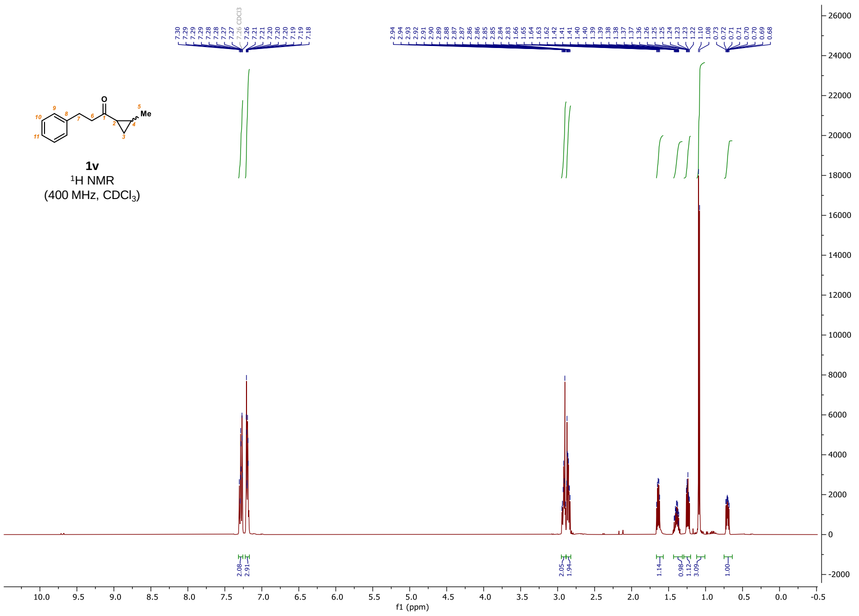

1v
^{1}H NMR
(400 MHz, $CDCl_3$)

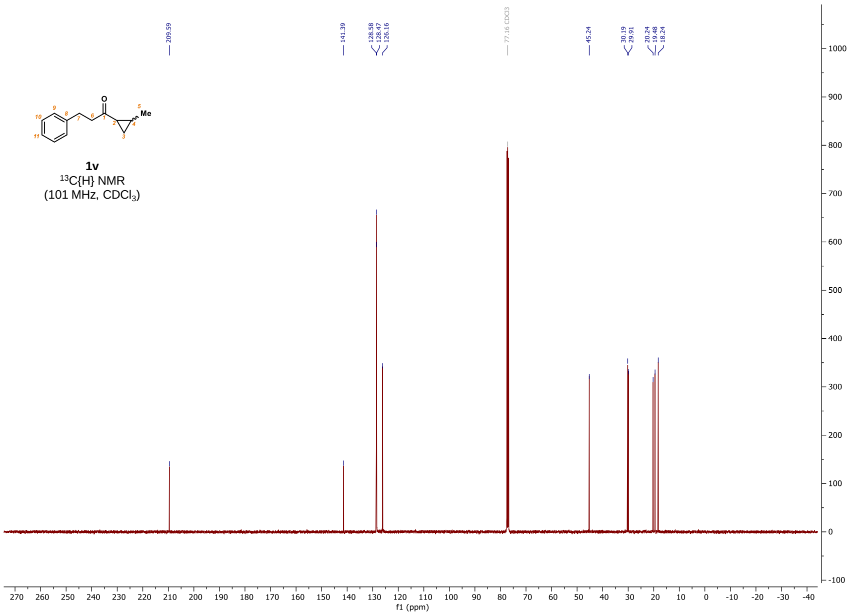

1v
$^{13}C\{H\}$ NMR
(101 MHz, $CDCl_3$)

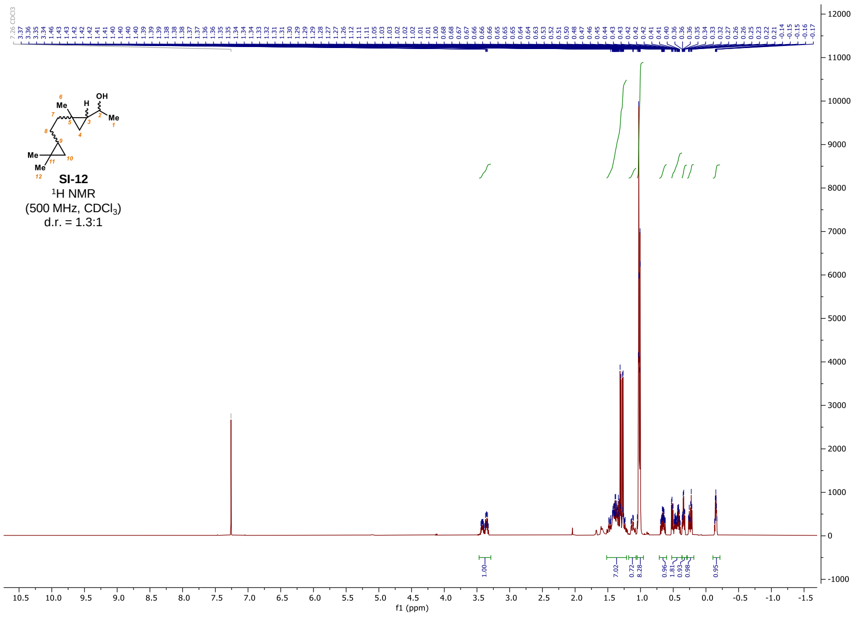
SI-12
^{1}H NMR
(500 MHz, CDCl$_3$)
d.r. = 1.3:1
f1 (ppm)

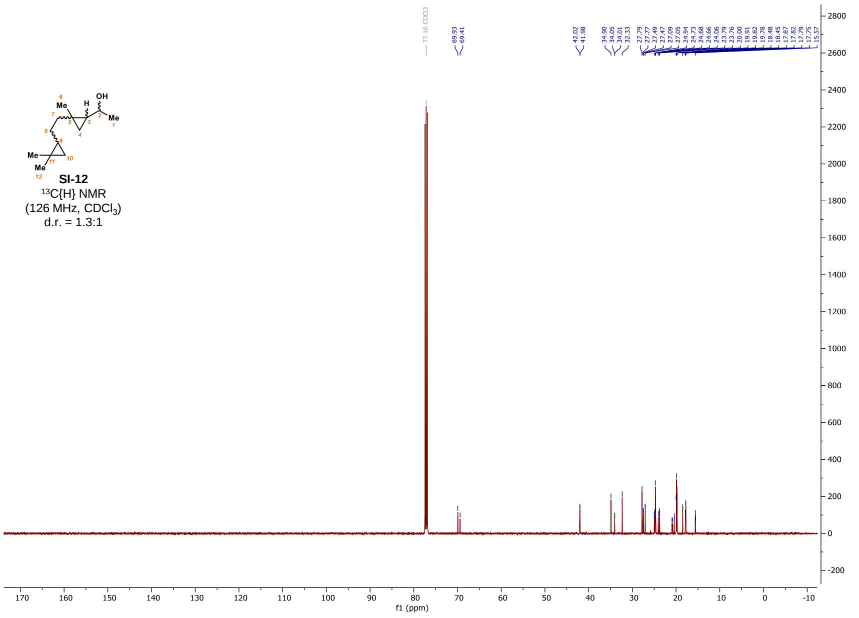
SI-12
^{13}C{H} NMR
(126 MHz, CDCl$_3$)
d.r. = 1.3:1
f1 (ppm)

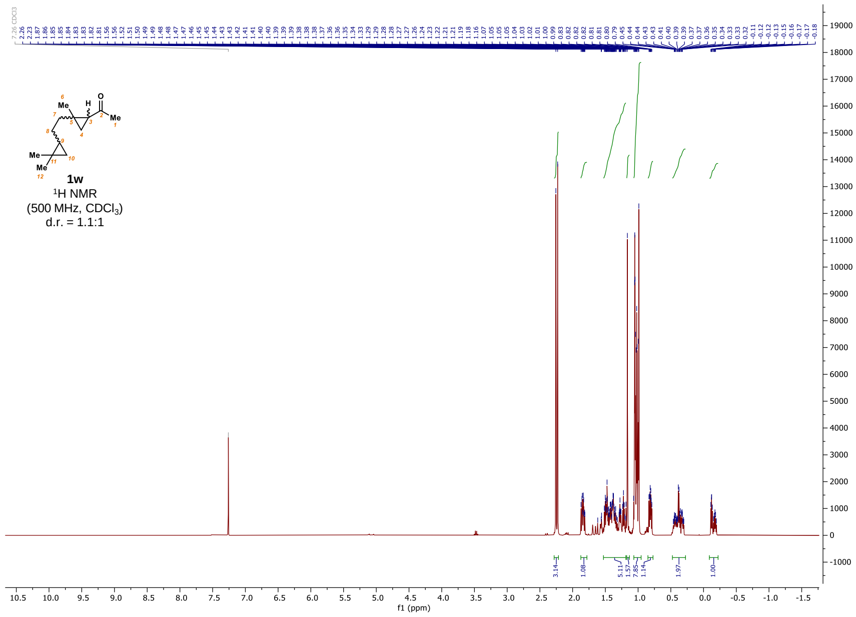
1w
1H NMR
(500 MHz, CDCl3)
d.r. = 1.1:1
f1 (ppm)

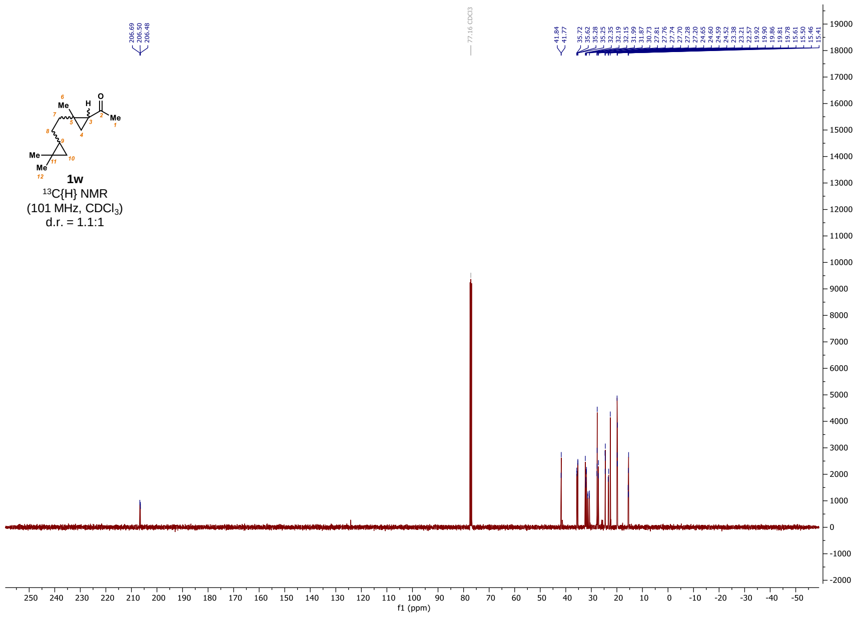
1w
13C{H} NMR
(101 MHz, CDCl3)
d.r. = 1.1:1
f1 (ppm)

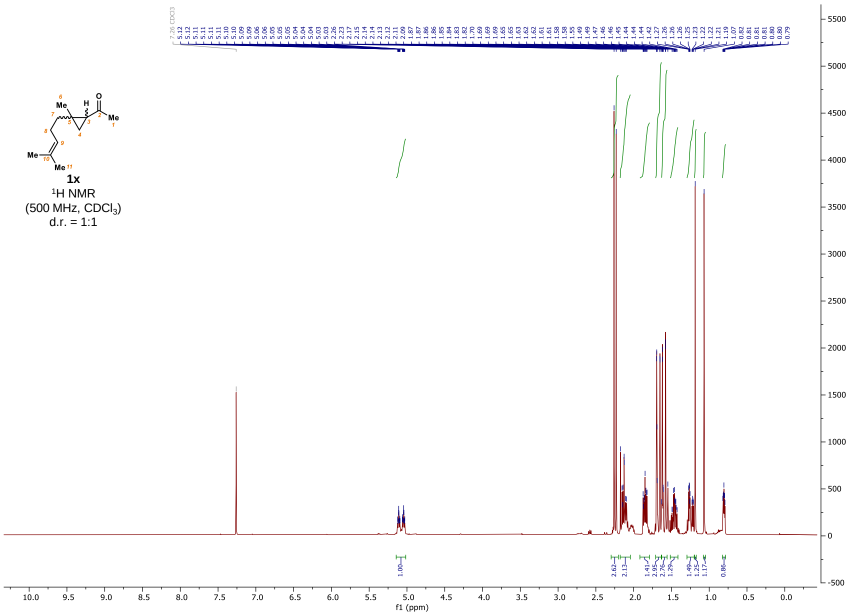
7.26 CDCl3
5.12 5.12 5.11 5.11 5.11 5.11 5.10 5.10 5.09 5.09 5.06 5.05 5.05 5.05 5.04 5.04 5.04 5.03 5.03 2.26 2.23 2.17 2.15 2.14 2.14 2.13 2.12 2.11 2.09 1.87 1.87 1.86 1.86 1.85 1.84 1.83 1.82 1.70 1.69 1.69 1.69 1.65 1.65 1.63 1.62 1.62 1.61 1.61 1.58 1.58 1.55 1.49 1.49 1.47 1.46 1.46 1.45 1.44 1.44 1.44 1.42 1.27 1.26 1.26 1.26 1.25 1.23 1.22 1.22 1.21 1.19 1.07 0.82 0.81 0.81 0.81 0.80 0.80 0.79
O
H
Me
6
7
5
3
2
Me
1
4
8
9
Me
10
Me
11
1x
1H NMR
(500 MHz, CDCl3)
d.r. = 1:1
1.00
2.62
2.13
1.41
2.95
2.76
1.29
1.49
1.25
1.17
0.86
f1 (ppm)

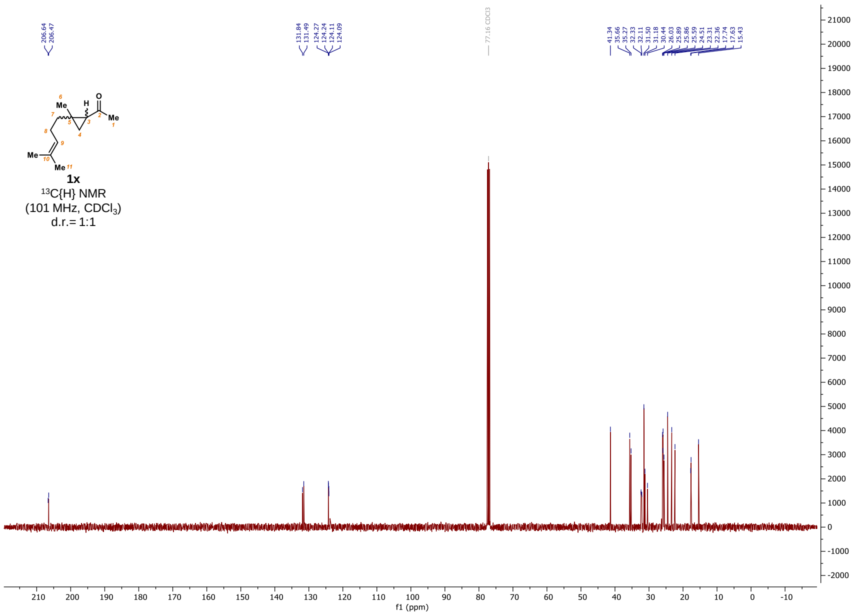
206.64
206.47
131.84
131.49
124.27
124.24
124.11
124.09
77.16 CDCl3
41.34 35.66 35.27 32.33 32.11 31.50 31.18 30.44 26.03 25.89 25.86 25.59 24.51 23.31 22.36 17.74 17.63 15.43
O
H
Me
6
7
5
3
2
Me
1
4
8
9
Me
10
Me
11
1x
13C{H} NMR
(101 MHz, CDCl3)
d.r.= 1:1
f1 (ppm)

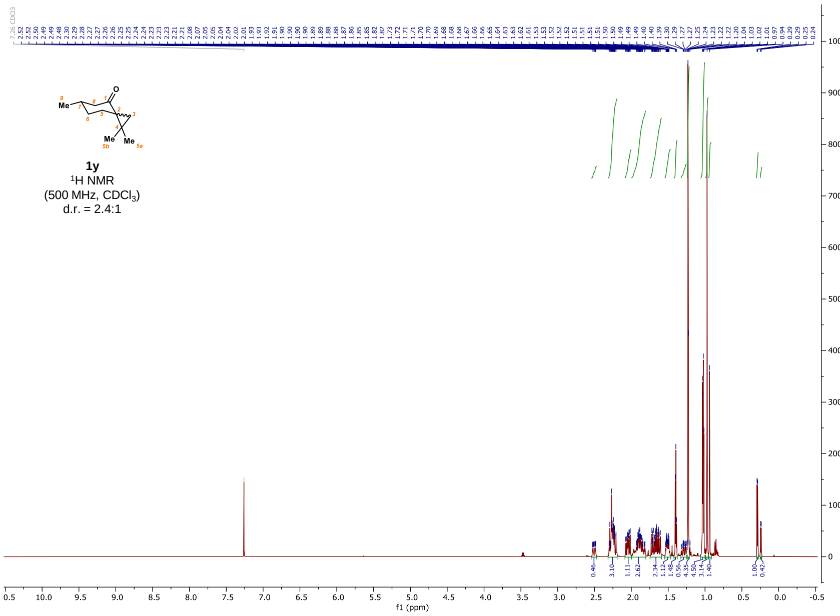
1y
¹H NMR
(500 MHz, CDCl3)
d.r. = 2.4:1
f1 (ppm)

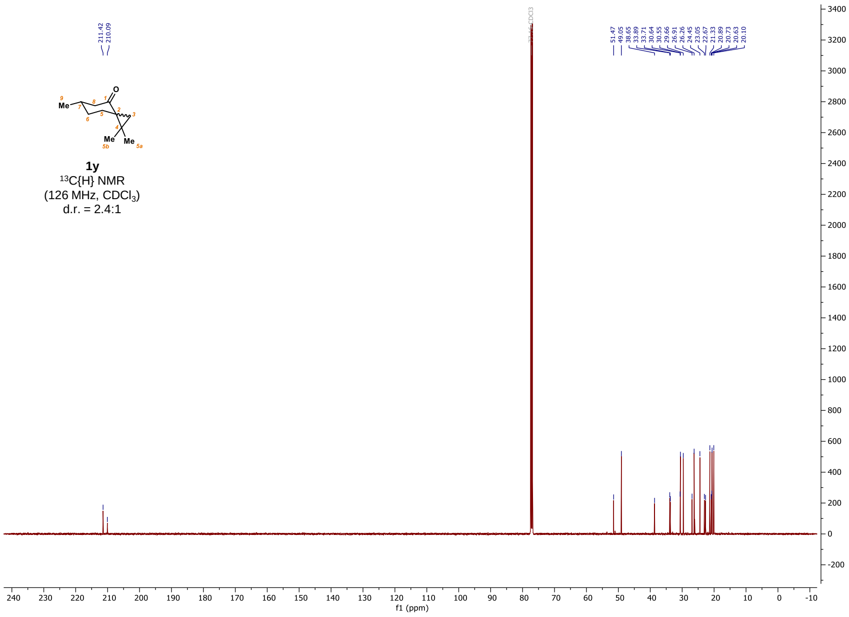
1y
¹³C{H} NMR
(126 MHz, CDCl3)
d.r. = 2.4:1
211.42
210.09
51.47
49.05
38.65
33.89
33.71
30.64
30.55
29.66
26.91
26.26
24.45
23.05
22.67
21.33
20.89
20.73
20.63
20.10
f1 (ppm)

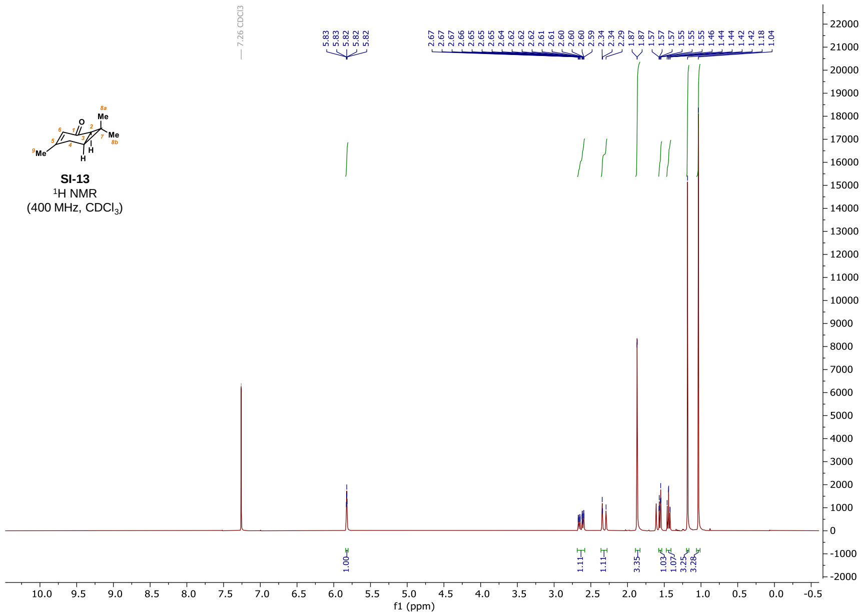
SI-13
^{1}H NMR
(400 MHz, $CDCl_3$)
7.26 CDCl3
f1 (ppm)

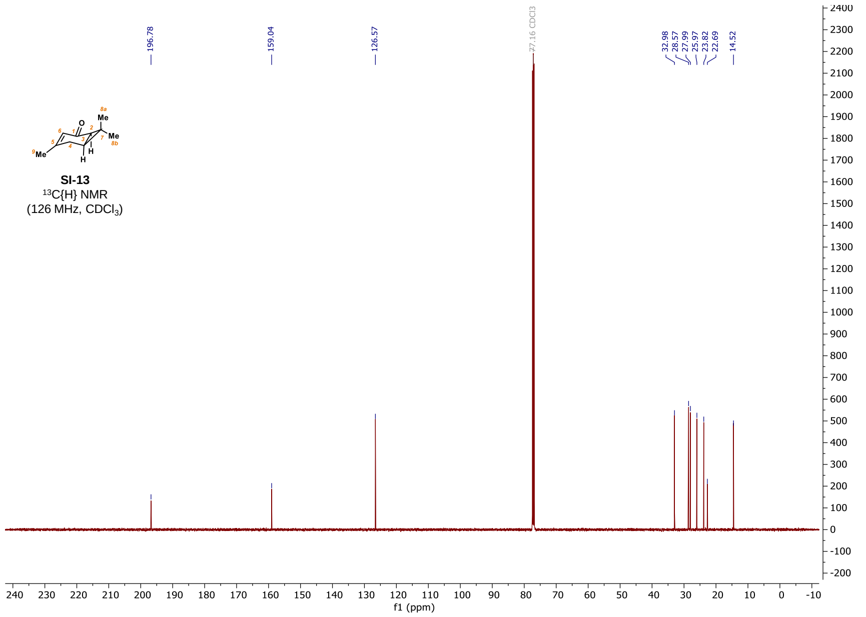
SI-13
^{13}C{H} NMR
(126 MHz, $CDCl_3$)
196.78
159.04
126.57
77.16 CDCl3
32.98
28.57
27.99
25.97
23.82
22.69
14.52
f1 (ppm)

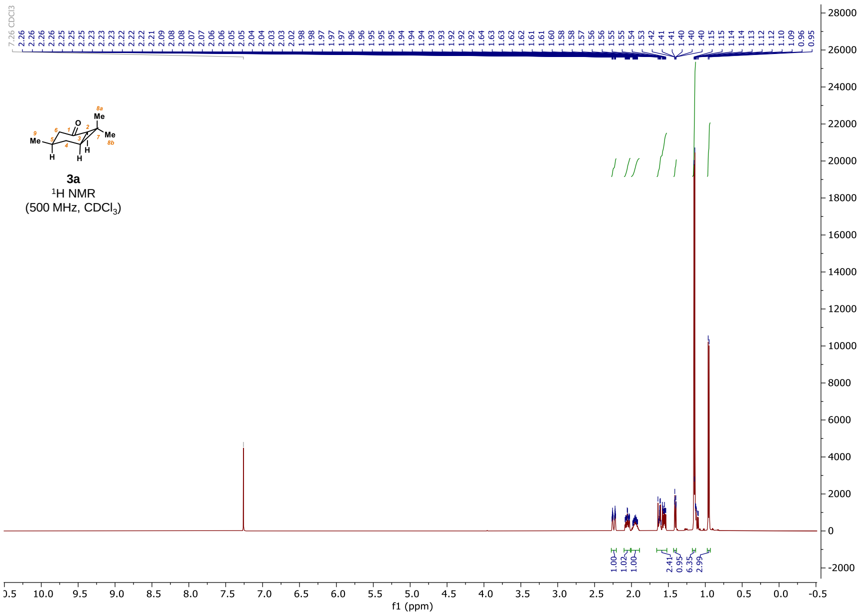
3a
^{1}H NMR
(500 MHz, CDCl$_3$)
f1 (ppm)

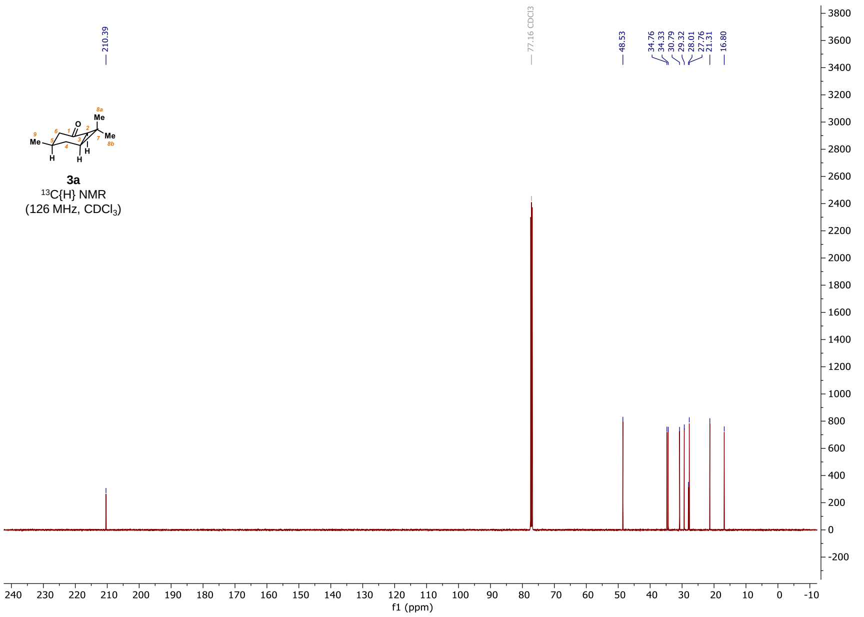
3a
^{13}C{H} NMR
(126 MHz, CDCl$_3$)
210.39
77.16 CDCl3
48.53
34.76
34.33
30.79
29.32
28.01
27.76
21.31
16.80
f1 (ppm)

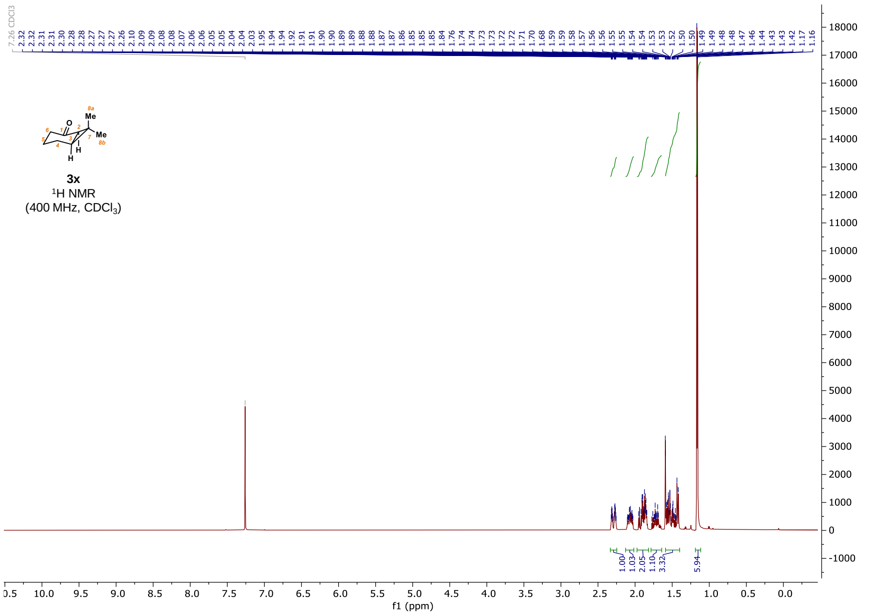

Me
8a
O
Me
8b
H
H
3x
1H NMR
(400 MHz, CDCl3)
7.26 CDCl3
1.00
1.02
2.05
1.10
3.32
5.94
f1 (ppm)

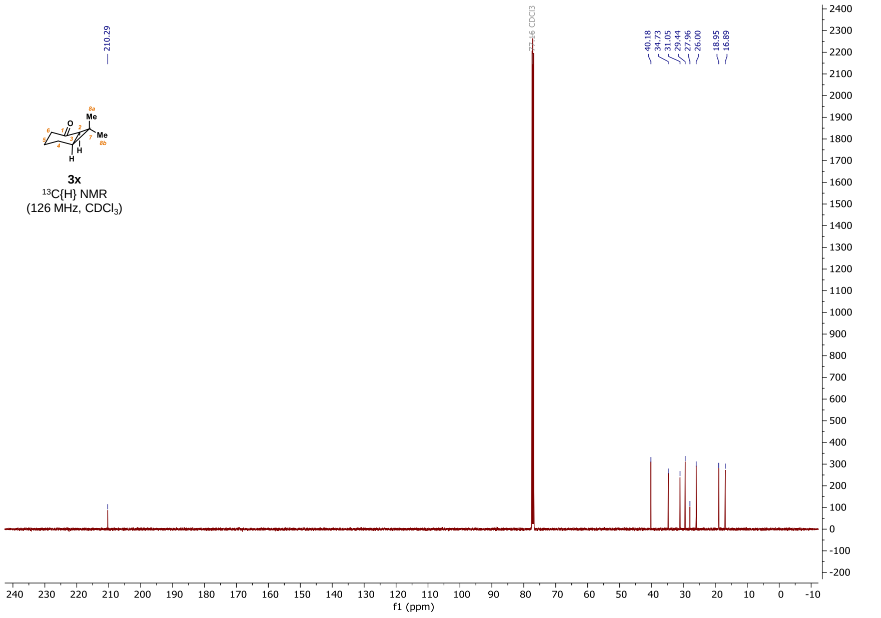

210.29
77.16 CDCl3
40.18
34.73
31.05
29.44
27.96
26.00
18.95
16.89
Me
8a
O
Me
8b
H
H
3x
13C{H} NMR
(126 MHz, CDCl3)
f1 (ppm)

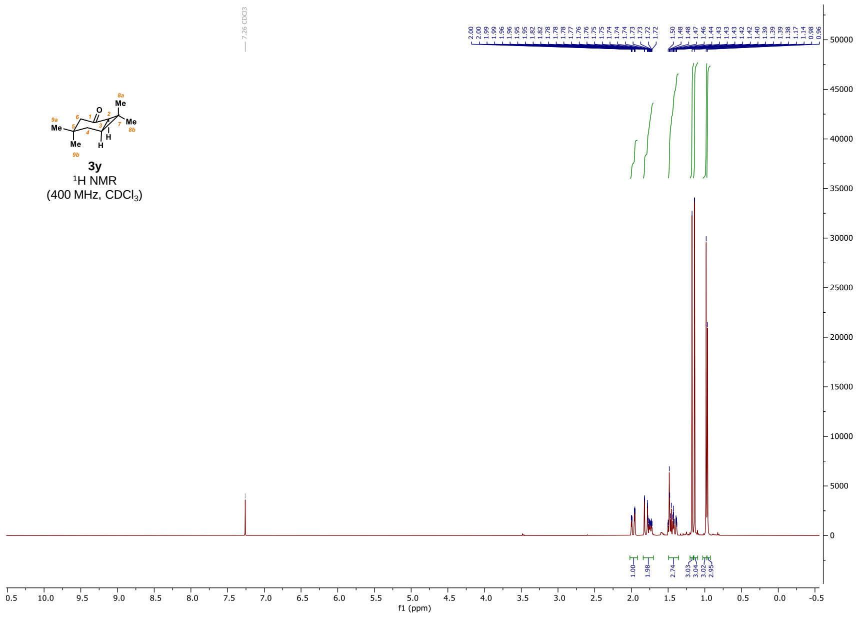

3y
^{1}H NMR
(400 MHz, CDCl$_3$)
7.26 CDCl3
f1 (ppm)

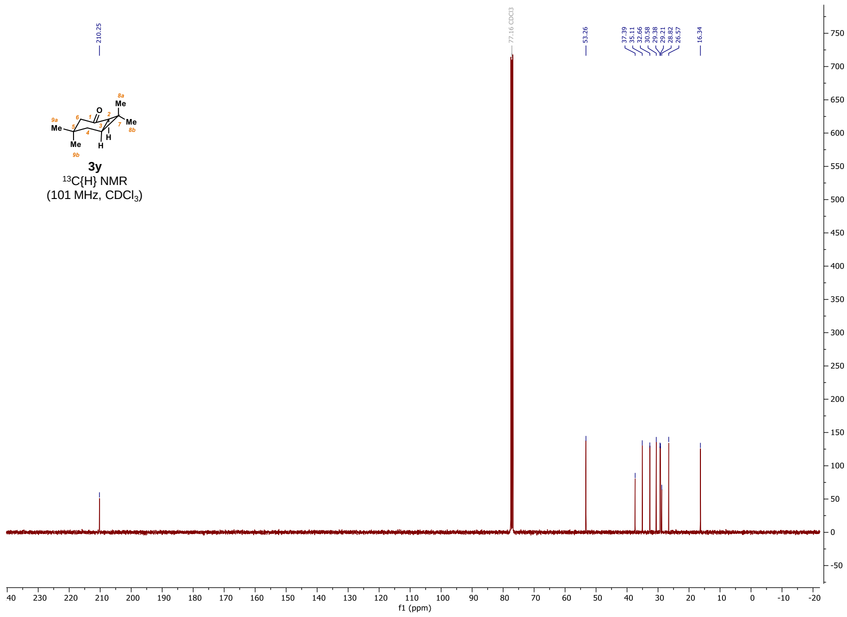

3y
^{13}C{H} NMR
(101 MHz, CDCl$_3$)
210.25
77.16 CDCl3
53.26
37.39
35.11
32.66
30.58
29.38
29.21
28.82
26.57
16.34
f1 (ppm)

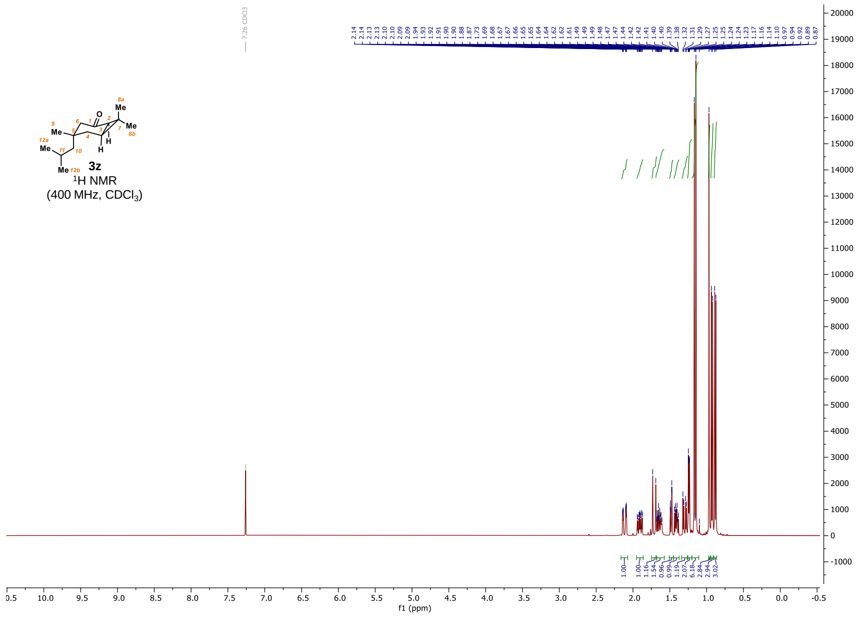
3z
1H NMR
(400 MHz, CDCl3)
f1 (ppm)

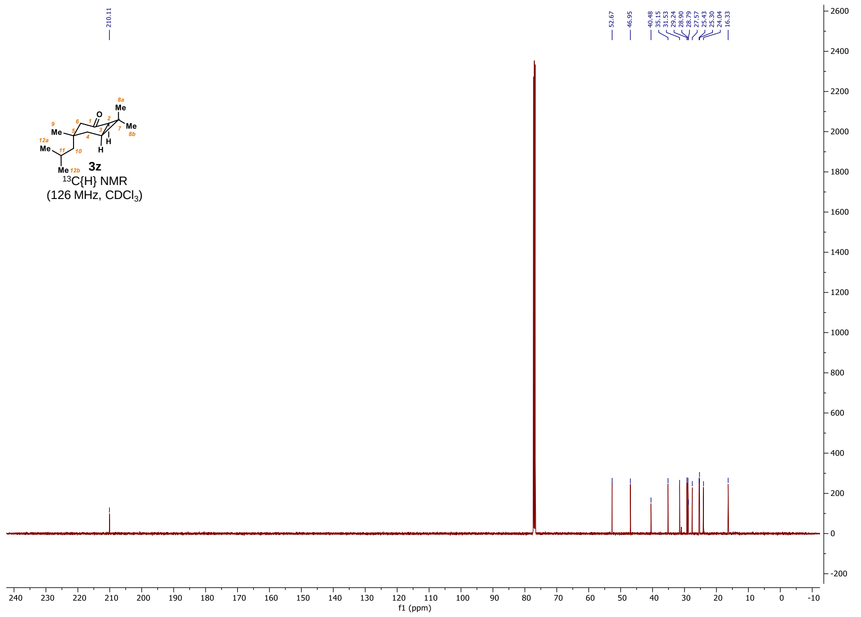
3z
13C{H} NMR
(126 MHz, CDCl3)
f1 (ppm)

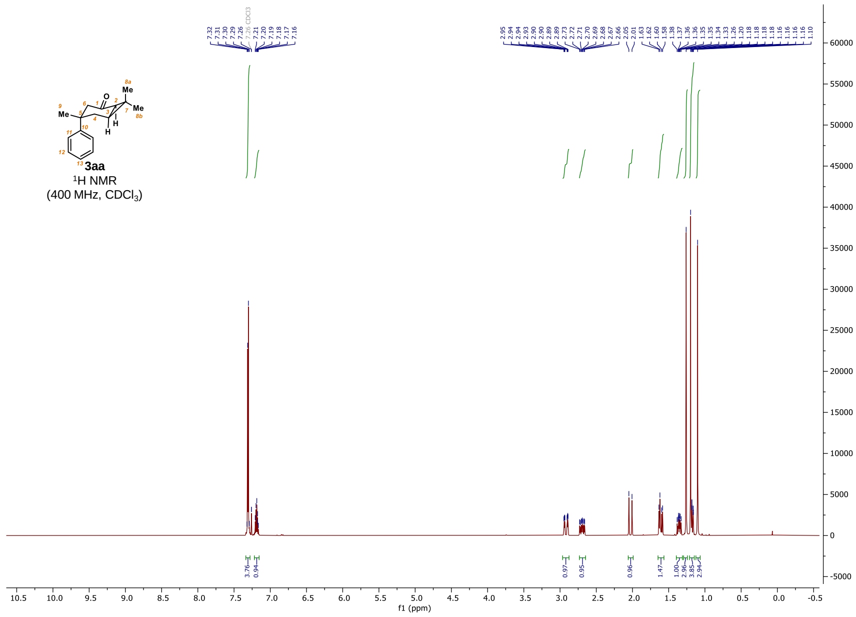
3aa
1H NMR
(400 MHz, CDCl3)
f1 (ppm)

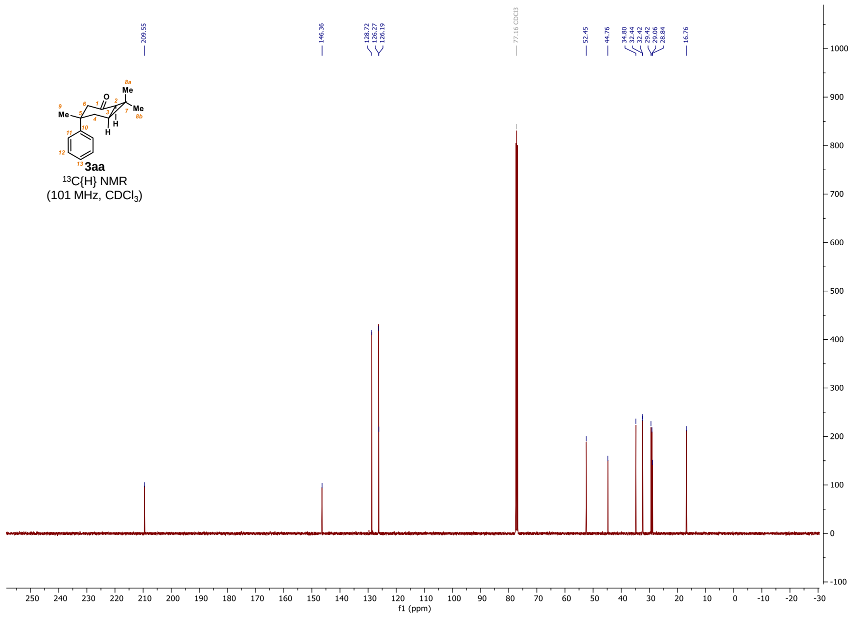
3aa
13C{H} NMR
(101 MHz, CDCl3)
f1 (ppm)

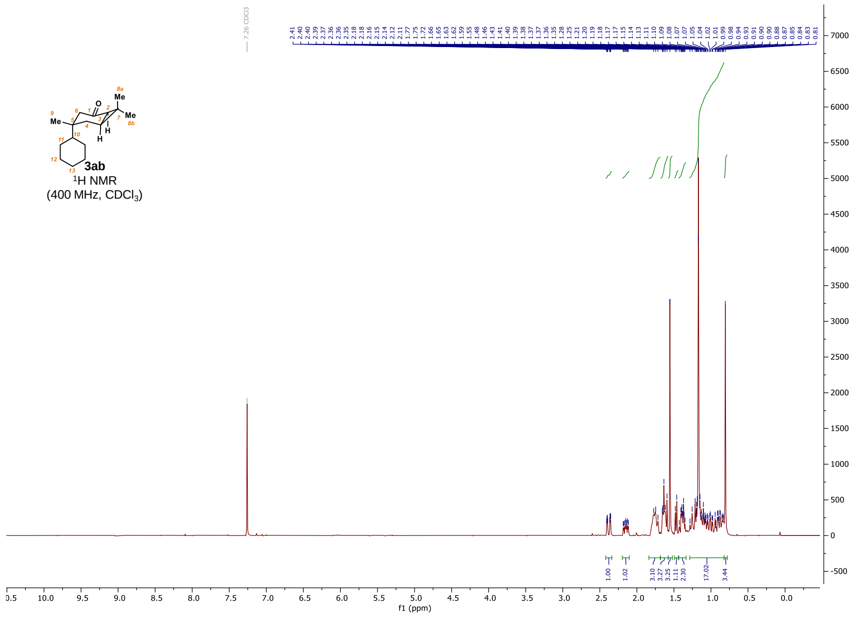
3ab
1H NMR
(400 MHz, CDCl3)
f1 (ppm)

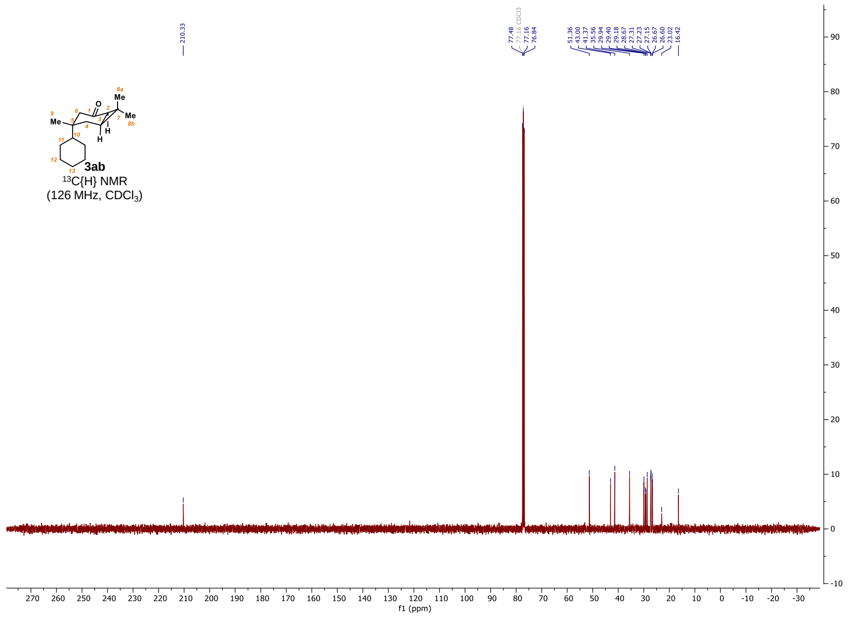
3ab
13C{H} NMR
(126 MHz, CDCl3)
f1 (ppm)

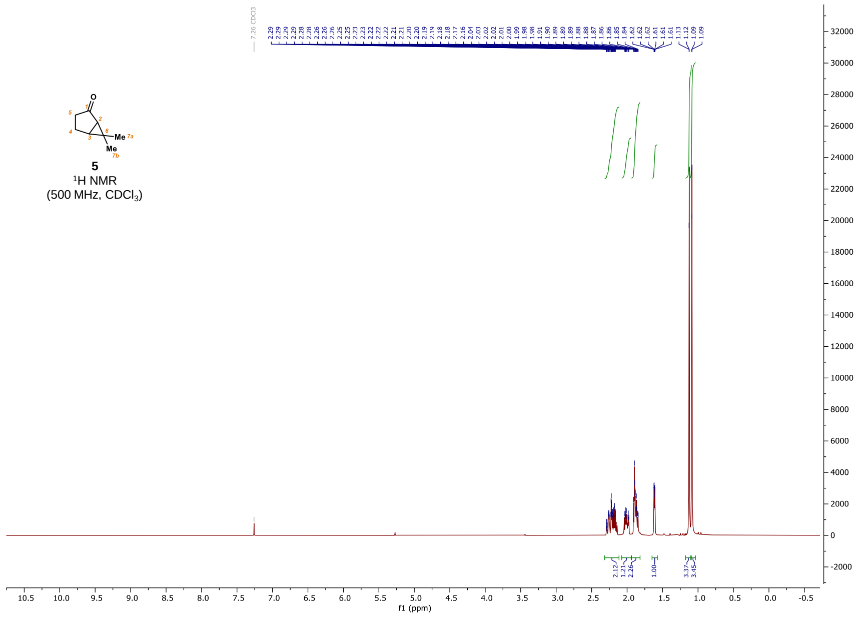

5
^{1}H NMR
(500 MHz, $CDCl_3$)
f1 (ppm)

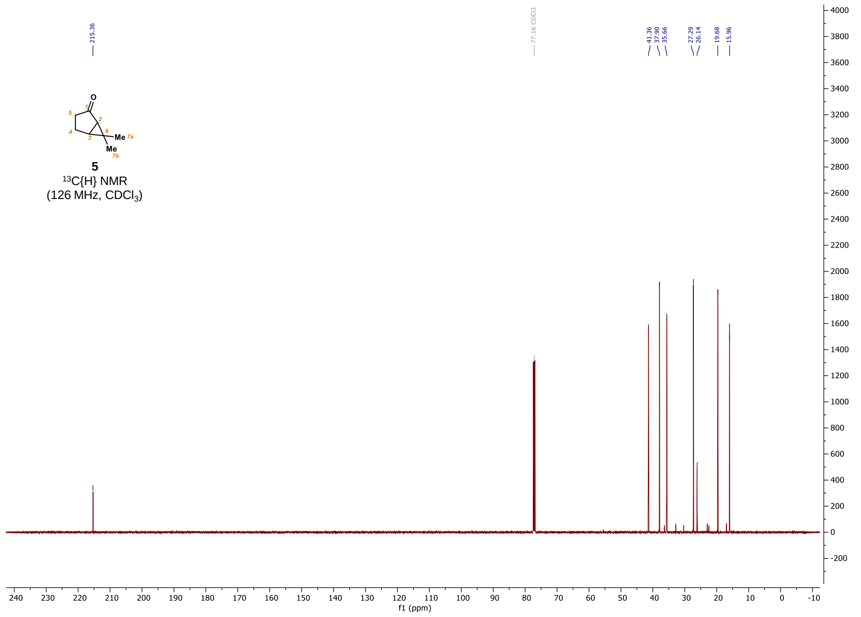

5
^{13}C{H} NMR
(126 MHz, $CDCl_3$)
215.36
41.36
37.90
35.66
27.29
26.14
19.68
15.96
f1 (ppm)

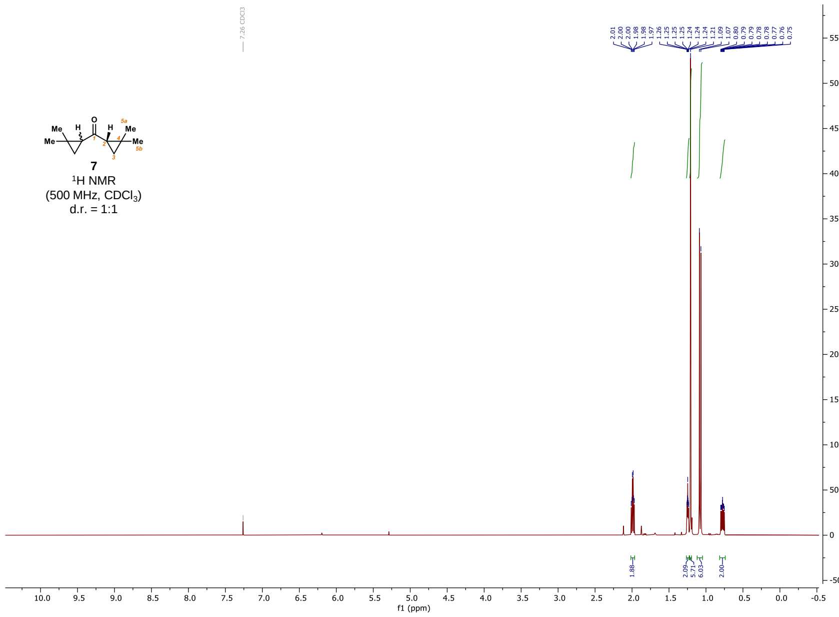
7
¹H NMR
(500 MHz, CDCl₃)
d.r. = 1:1
7.26 CDCl3
f1 (ppm)

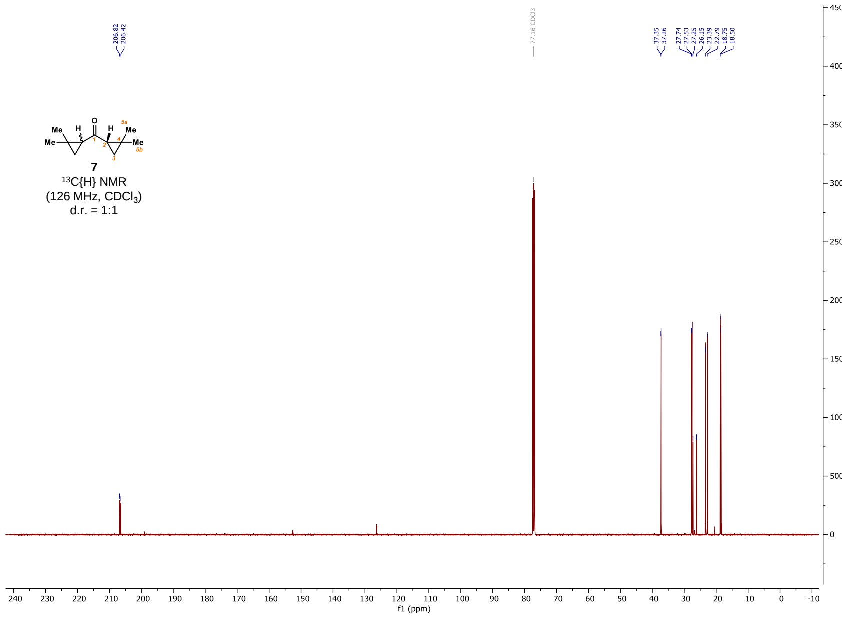
7
¹³C{H} NMR
(126 MHz, CDCl₃)
d.r. = 1:1
77.16 CDCl3
f1 (ppm)

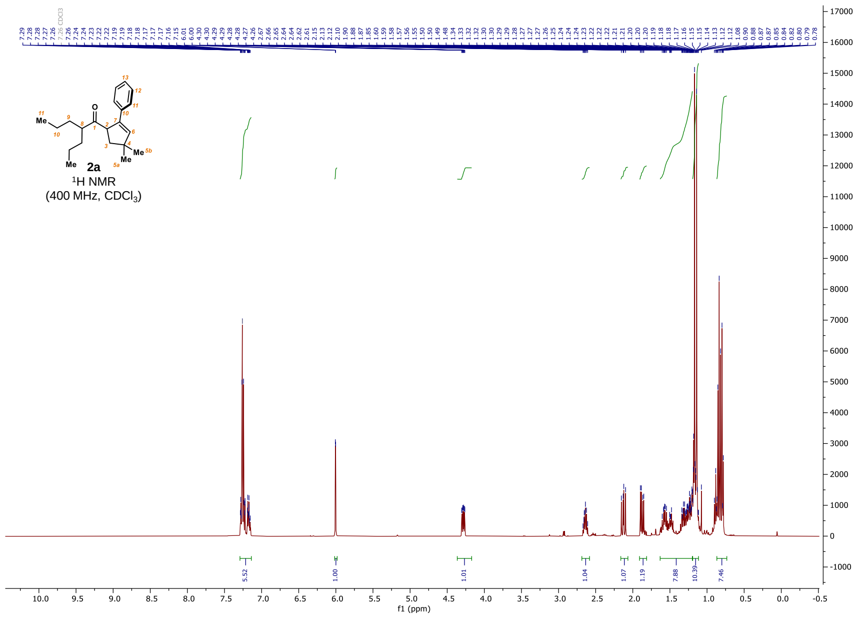
2a
^{1}H NMR
(400 MHz, CDCl$_3$)
f1 (ppm)

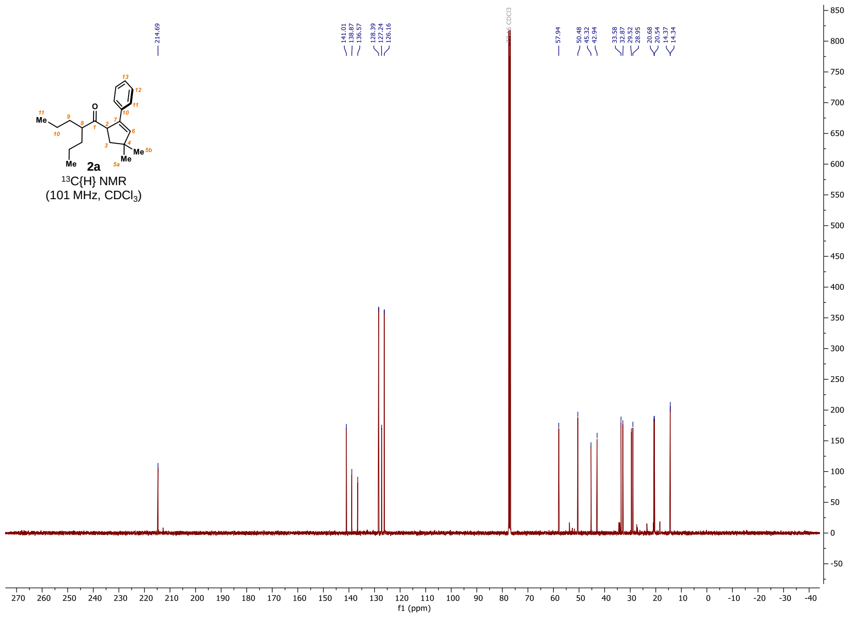
2a
^{13}C{H} NMR
(101 MHz, CDCl$_3$)
214.69
141.01
138.87
136.57
128.39
127.24
126.16
57.94
50.48
45.32
42.94
33.58
32.87
29.52
28.95
20.68
20.54
14.37
14.34
f1 (ppm)

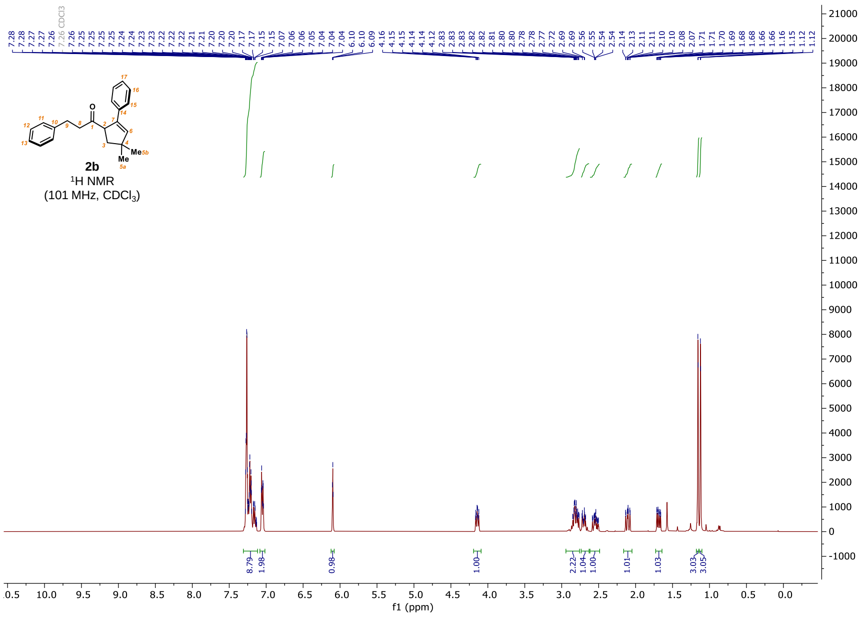
2b
^{1}H NMR
(101 MHz, CDCl$_3$)
f1 (ppm)

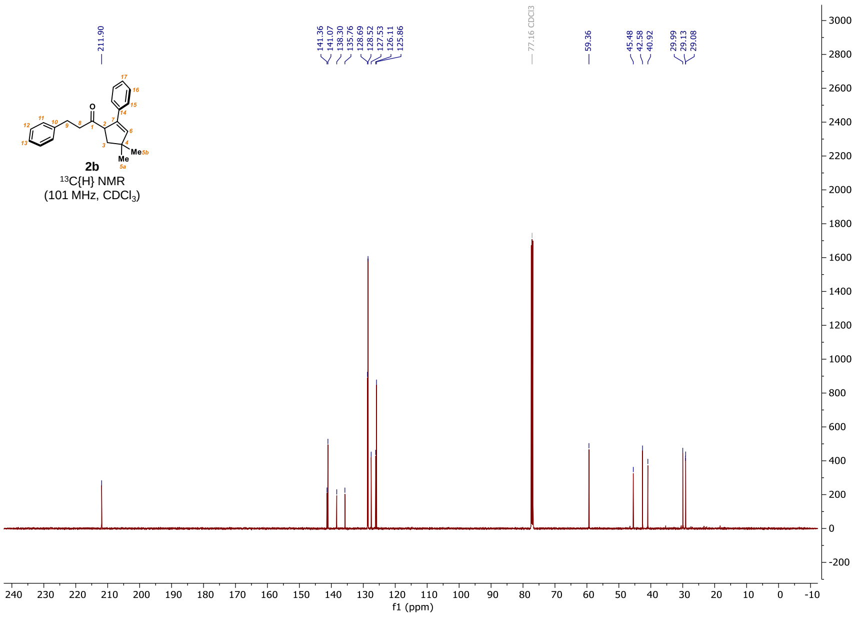
211.90
141.36
141.07
138.30
135.76
128.69
128.52
127.53
126.11
125.86
77.16 CDCl3
59.36
45.48
42.58
40.92
29.99
29.13
29.08
2b
^{13}C{H} NMR
(101 MHz, CDCl$_3$)
f1 (ppm)

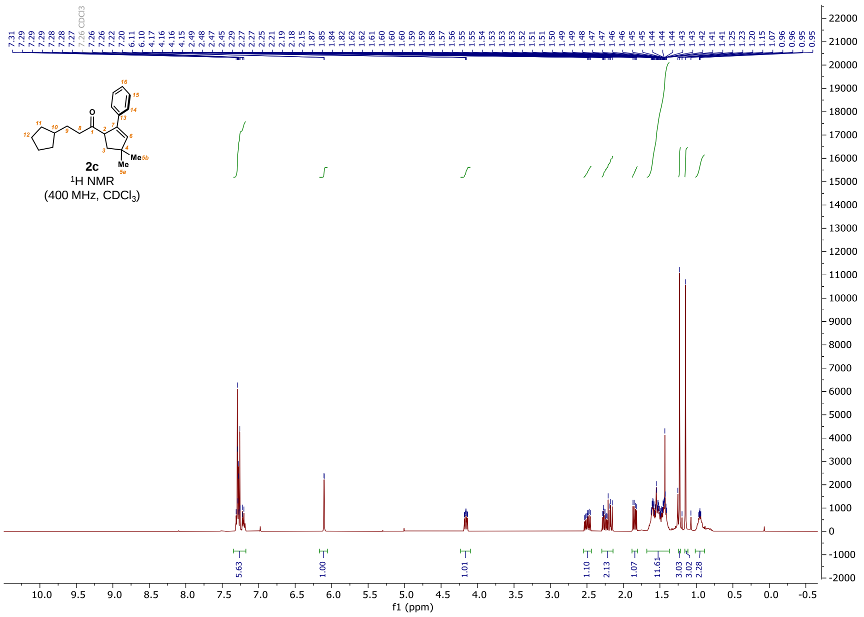
2c
^{1}H NMR
(400 MHz, CDCl$_3$)
f1 (ppm)

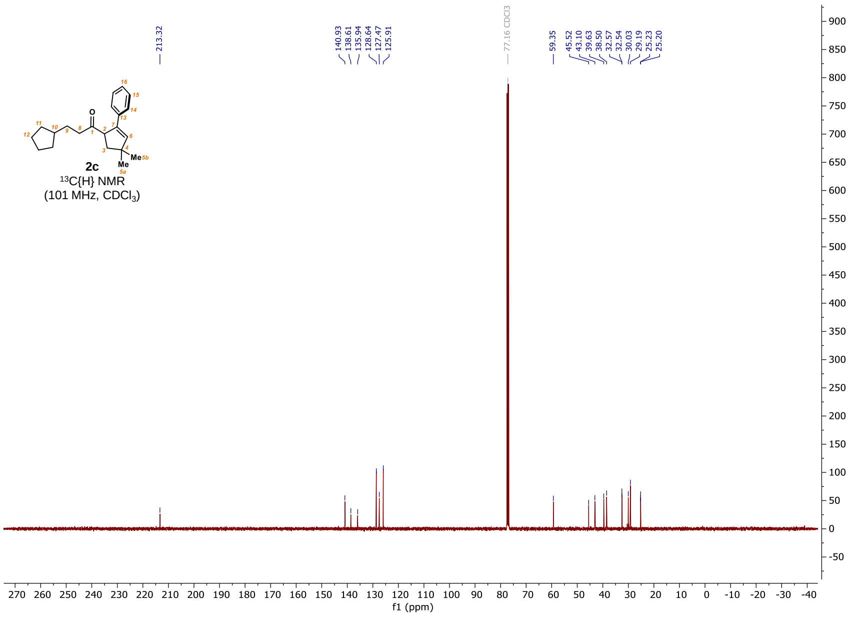
2c
^{13}C{H} NMR
(101 MHz, CDCl$_3$)
213.32
140.93
138.61
135.94
128.64
127.47
125.91
77.16 CDCl3
59.35
45.52
43.10
39.63
38.50
32.57
32.54
30.03
29.19
25.23
25.20
f1 (ppm)

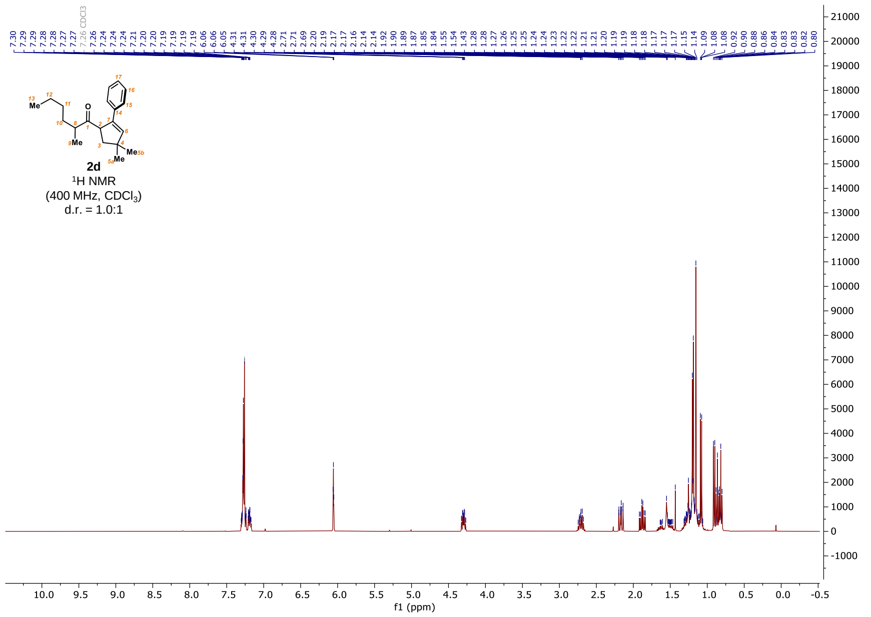
2d
¹H NMR
(400 MHz, CDCl3)
d.r. = 1.0:1
f1 (ppm)

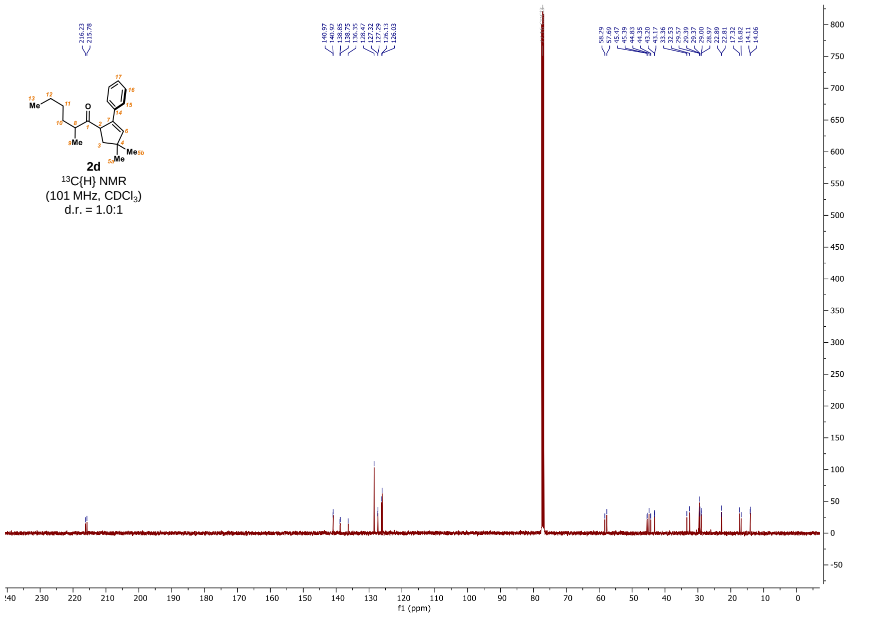
2d
¹³C{H} NMR
(101 MHz, CDCl3)
d.r. = 1.0:1
f1 (ppm)

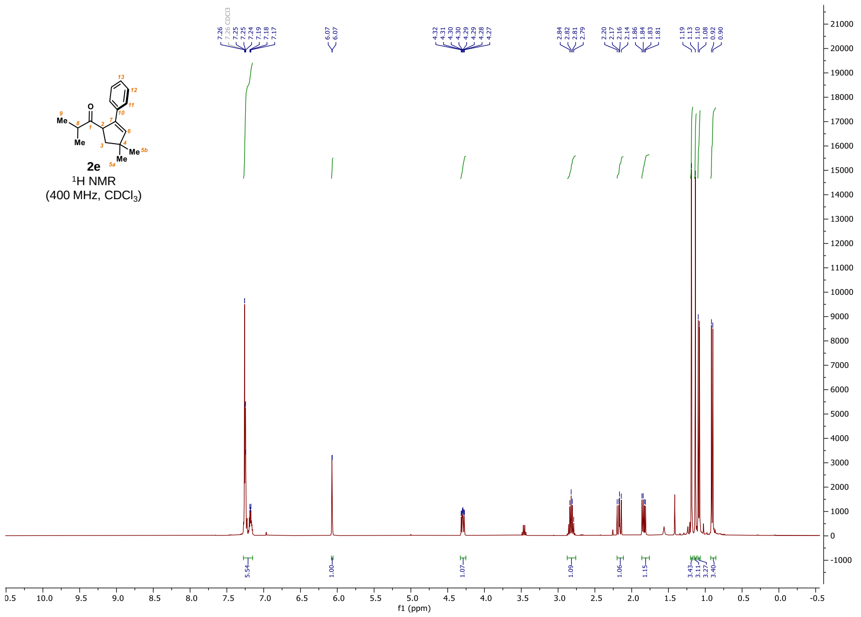
7.26
7.26 CDCl3
7.25
7.25
7.24
7.19
7.18
7.17
6.07
6.07
4.32
4.31
4.30
4.30
4.29
4.29
4.28
4.27
2.84
2.82
2.81
2.79
2.20
2.17
2.16
2.14
1.86
1.84
1.83
1.81
1.19
1.13
1.10
1.08
0.92
0.90
2e
1H NMR
(400 MHz, CDCl3)
f1 (ppm)

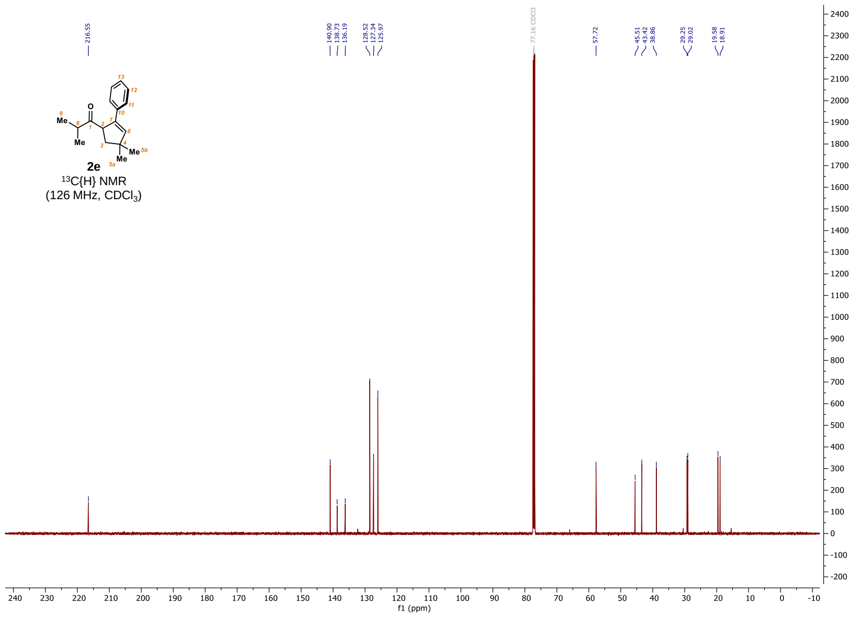
216.55
140.90
138.73
136.19
128.52
127.34
125.97
77.16 CDCl3
57.72
45.51
43.42
38.86
29.25
29.02
19.58
18.91
2e
13C{H} NMR
(126 MHz, CDCl3)
f1 (ppm)

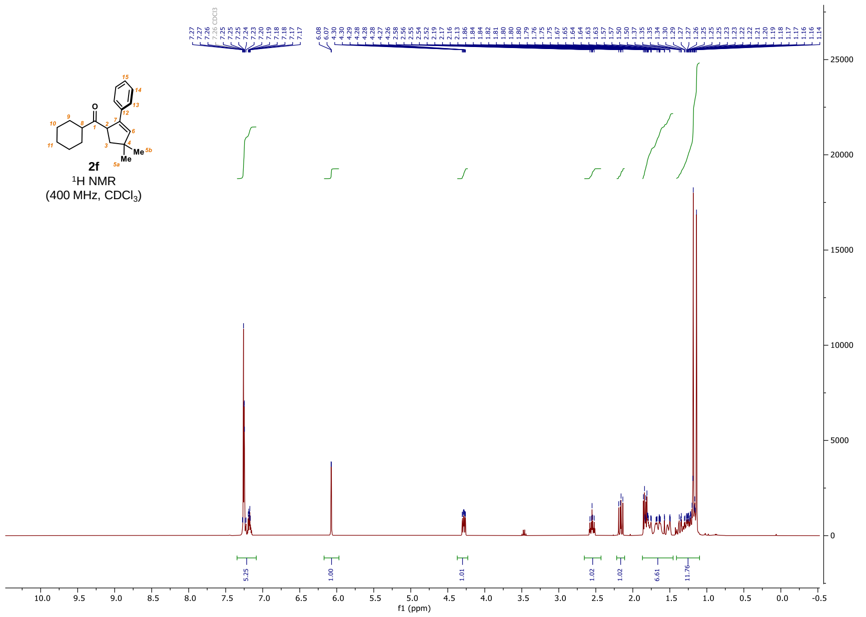
2f
¹H NMR
(400 MHz, CDCl3)
f1 (ppm)

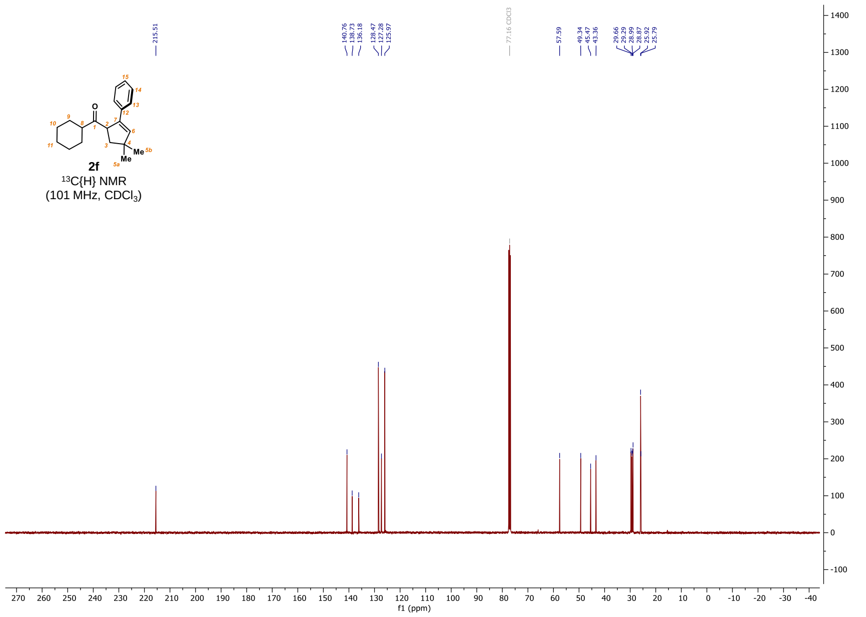
2f
¹³C{H} NMR
(101 MHz, CDCl3)
f1 (ppm)

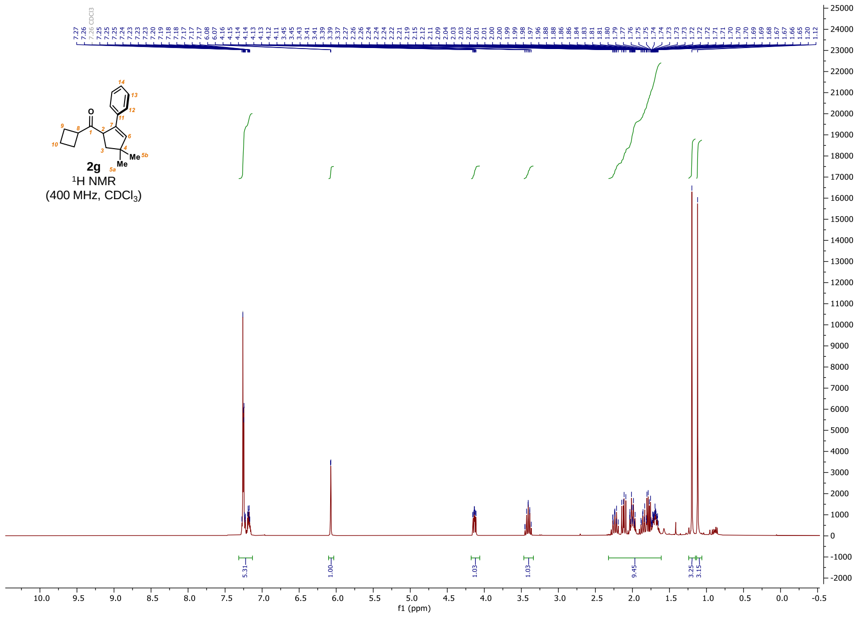
2g
^{1}H NMR
(400 MHz, $CDCl_3$)
f1 (ppm)

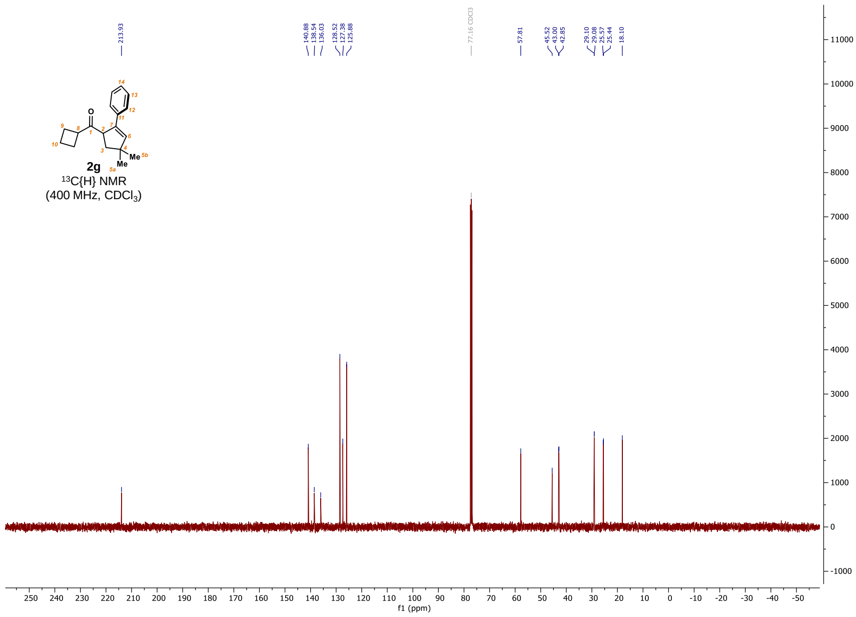
213.93
140.86
138.54
136.03
128.52
127.38
125.88
57.81
45.52
43.00
42.85
29.10
29.08
25.57
25.44
18.10
2g
^{13}C{H} NMR
(400 MHz, $CDCl_3$)
f1 (ppm)

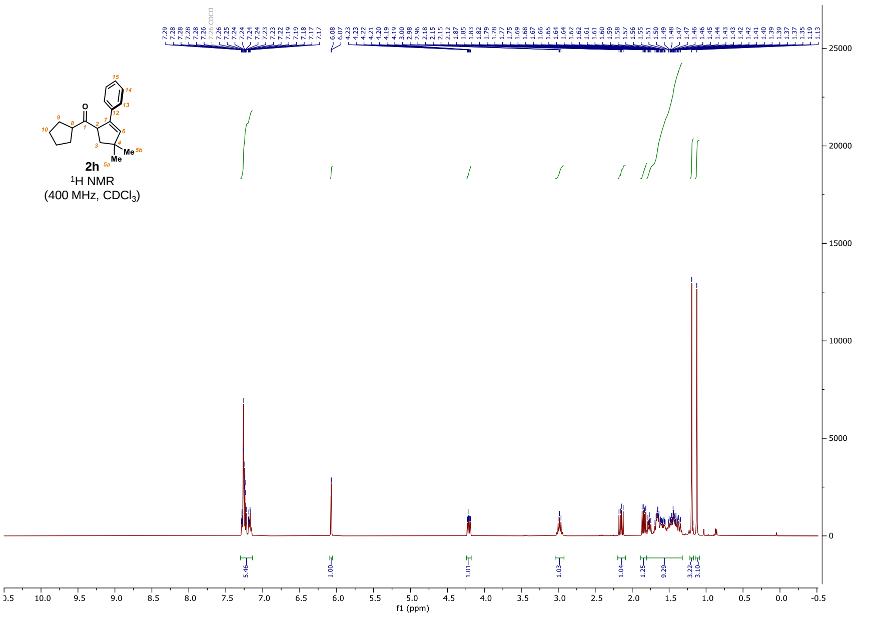
2h
1H NMR
(400 MHz, CDCl3)
5.46
1.00
1.01
1.03
1.04
1.25
9.29
3.22
3.10
f1 (ppm)

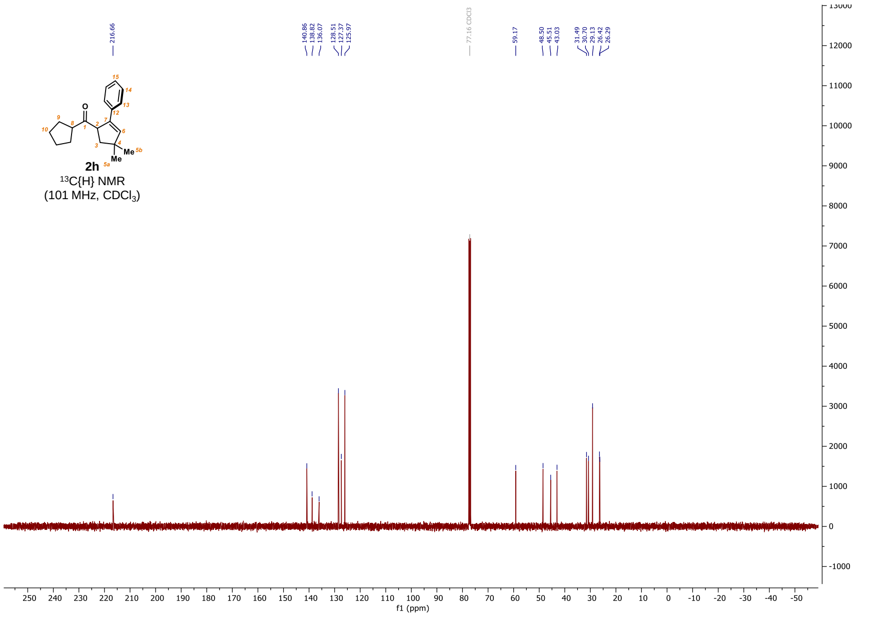
216.66
140.86
138.82
136.07
128.51
127.37
125.97
77.16 CDCl3
59.17
48.50
45.51
43.03
31.49
30.70
29.13
26.42
26.29
2h
13C{H} NMR
(101 MHz, CDCl3)
f1 (ppm)

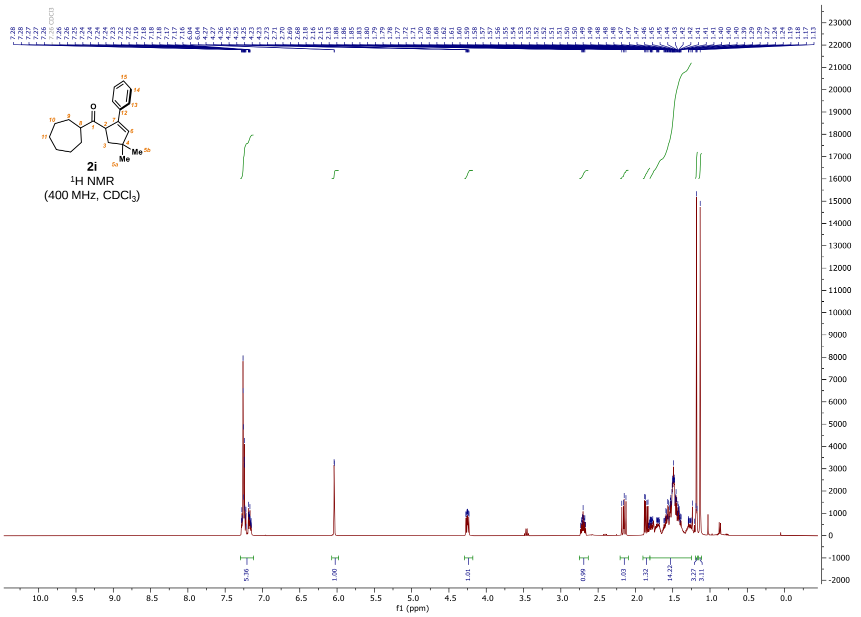
2i
1H NMR
(400 MHz, CDCl3)
f1 (ppm)

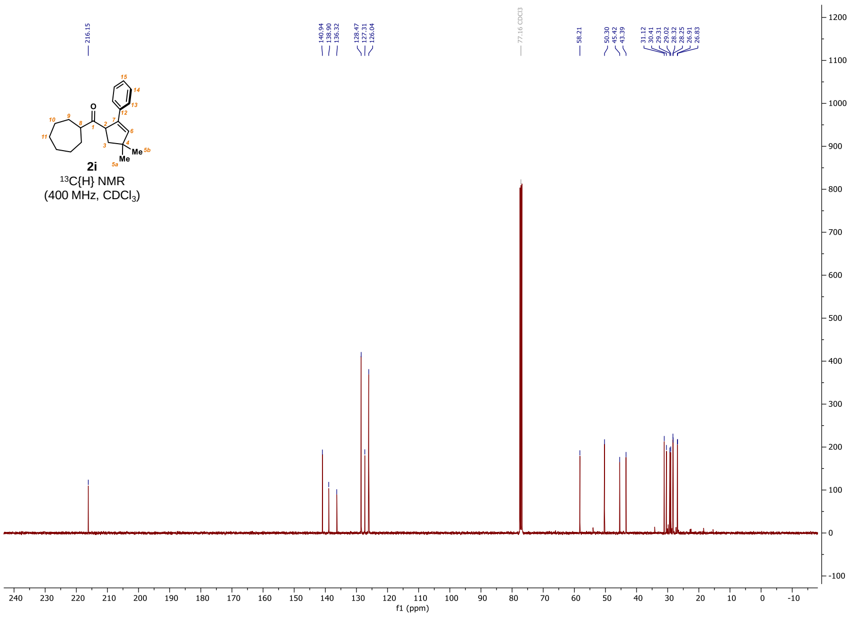
2i
13C{H} NMR
(400 MHz, CDCl3)
f1 (ppm)

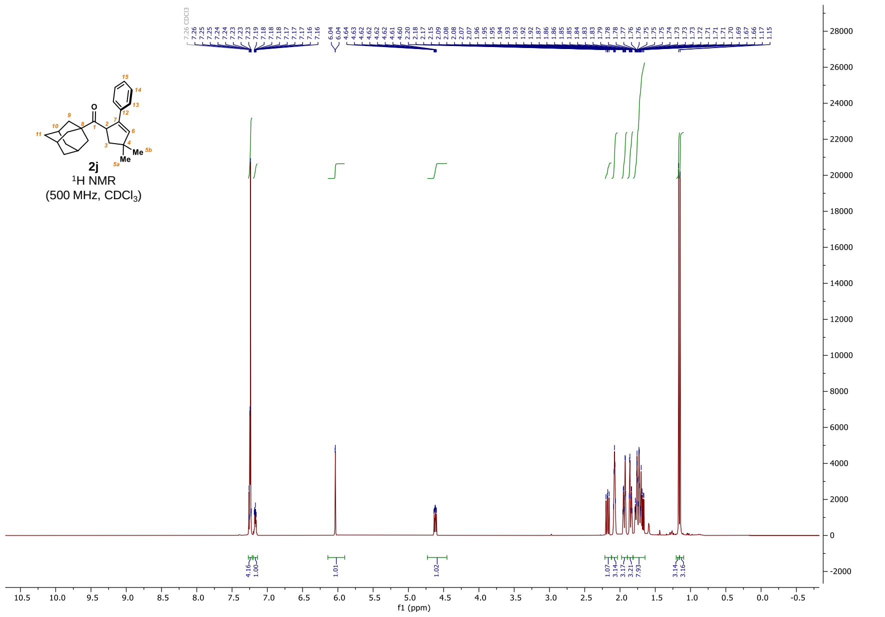

2j
^{1}H NMR
(500 MHz, CDCl$_3$)

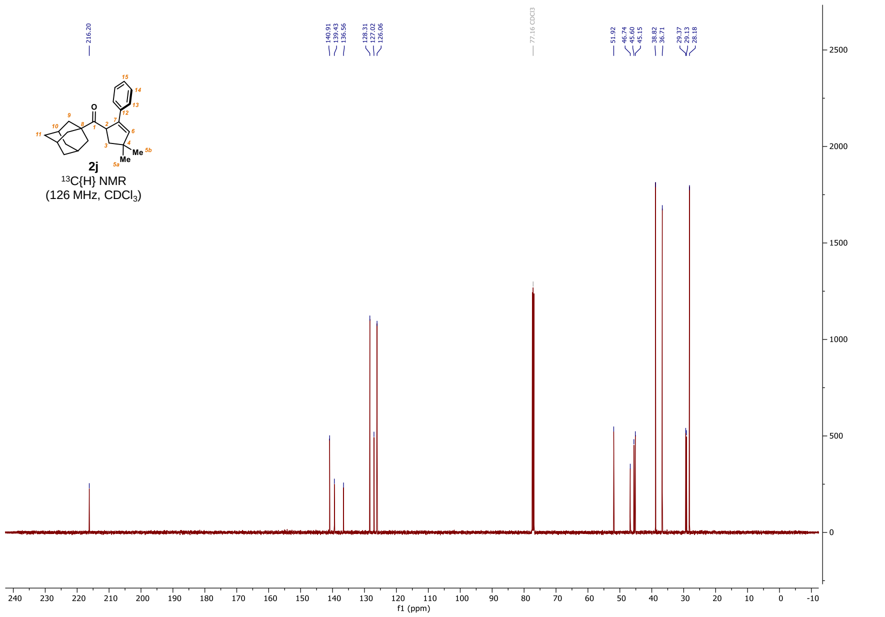

2j
^{13}C{H} NMR
(126 MHz, CDCl$_3$)

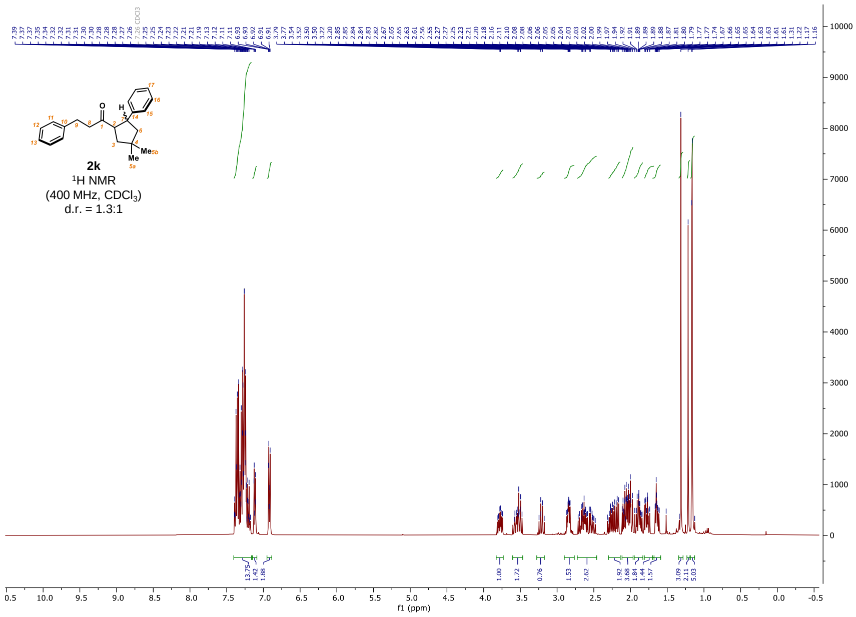
2k
^{1}H NMR
(400 MHz, $CDCl_3$)
d.r. = 1.3:1
f1 (ppm)

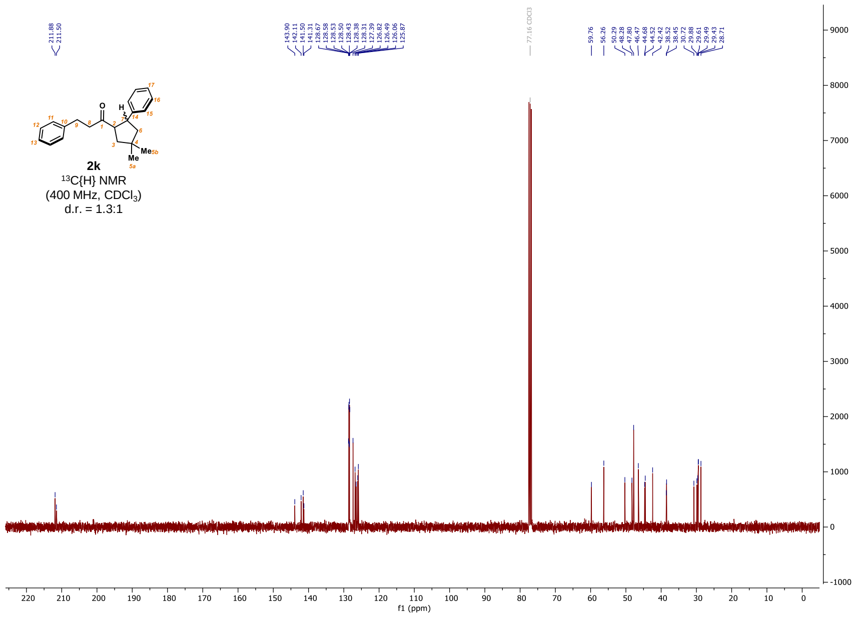
2k
^{13}C{H} NMR
(400 MHz, $CDCl_3$)
d.r. = 1.3:1
f1 (ppm)

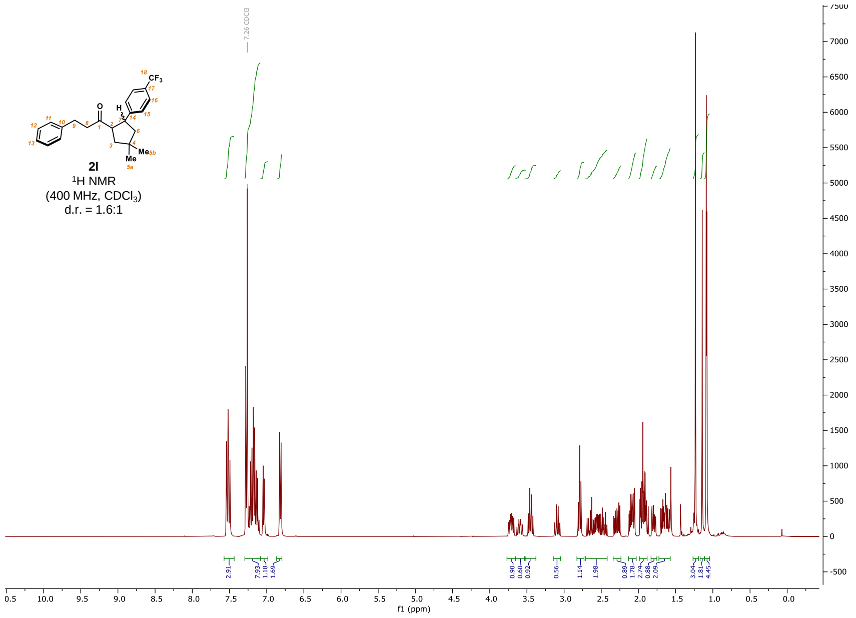
2l
^{1}H NMR
(400 MHz, $CDCl_3$)
d.r. = 1.6:1
f1 (ppm)

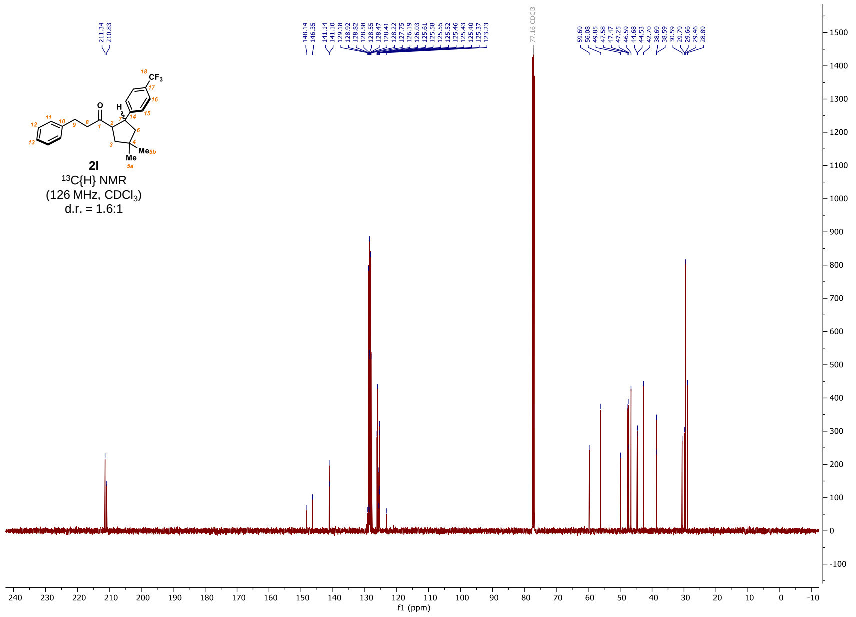
2l
^{13}C{H} NMR
(126 MHz, $CDCl_3$)
d.r. = 1.6:1
f1 (ppm)

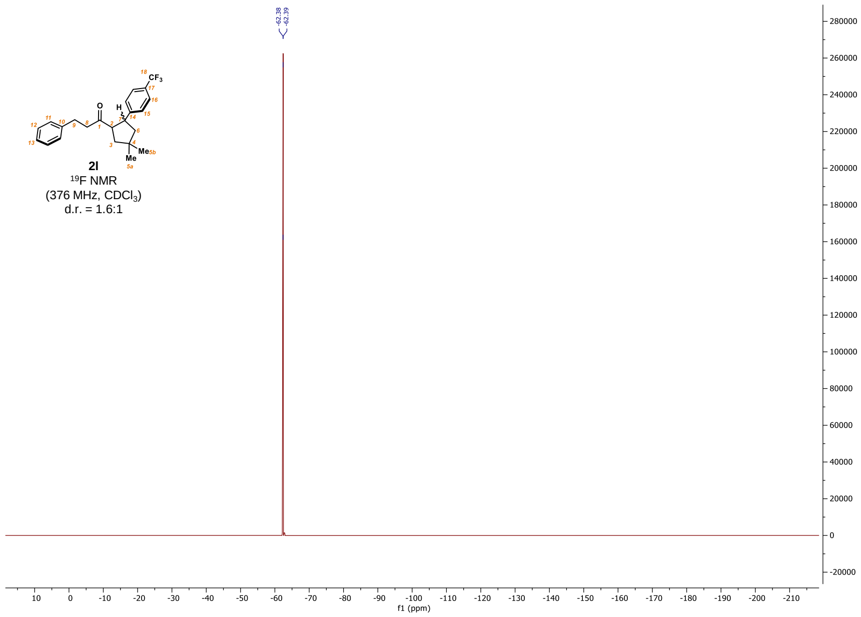

-62.38
-62.39
2l
19F NMR
(376 MHz, CDCl3)
d.r. = 1.6:1
f1 (ppm)

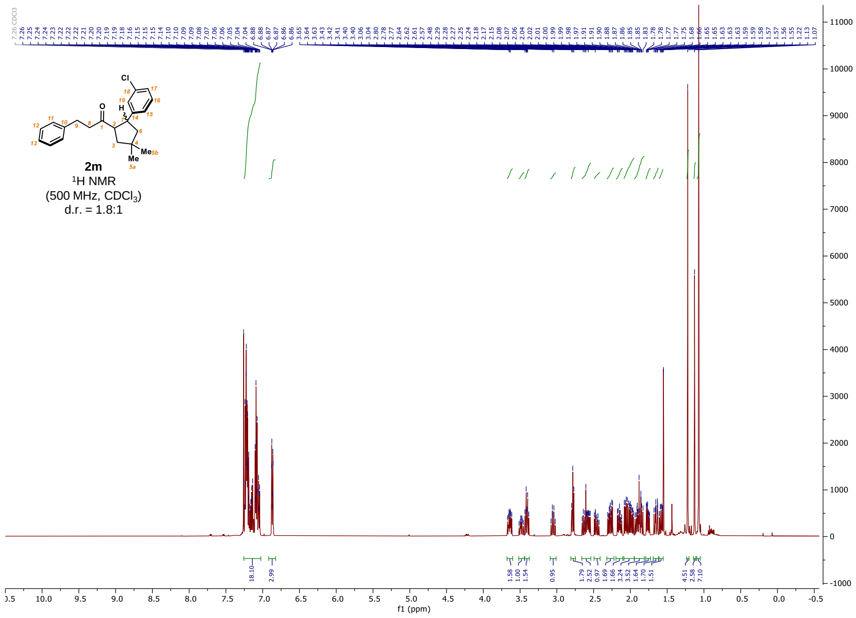

2m
1H NMR
(500 MHz, CDCl3)
d.r. = 1.8:1
f1 (ppm)

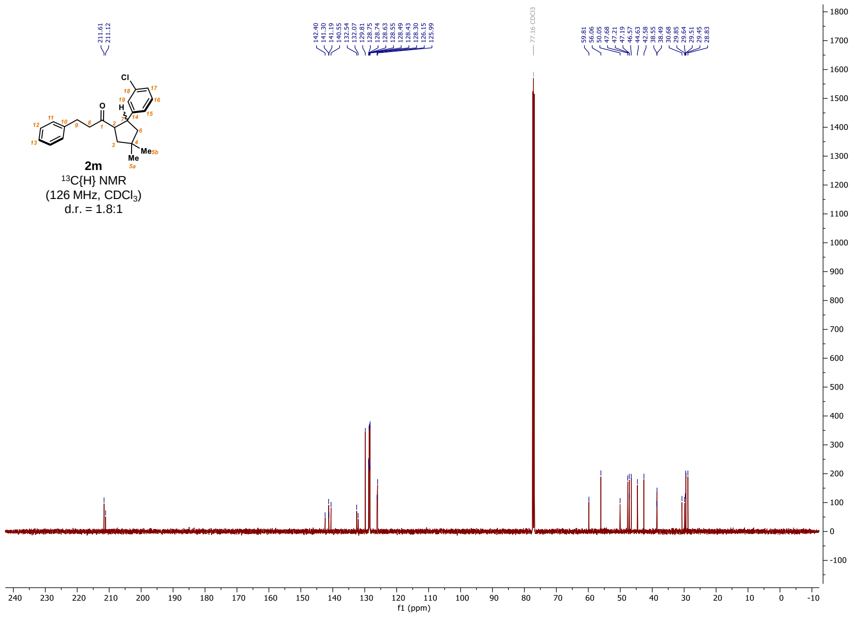
2m
^{13}C{H} NMR
(126 MHz, CDCl$_3$)
d.r. = 1.8:1
f1 (ppm)

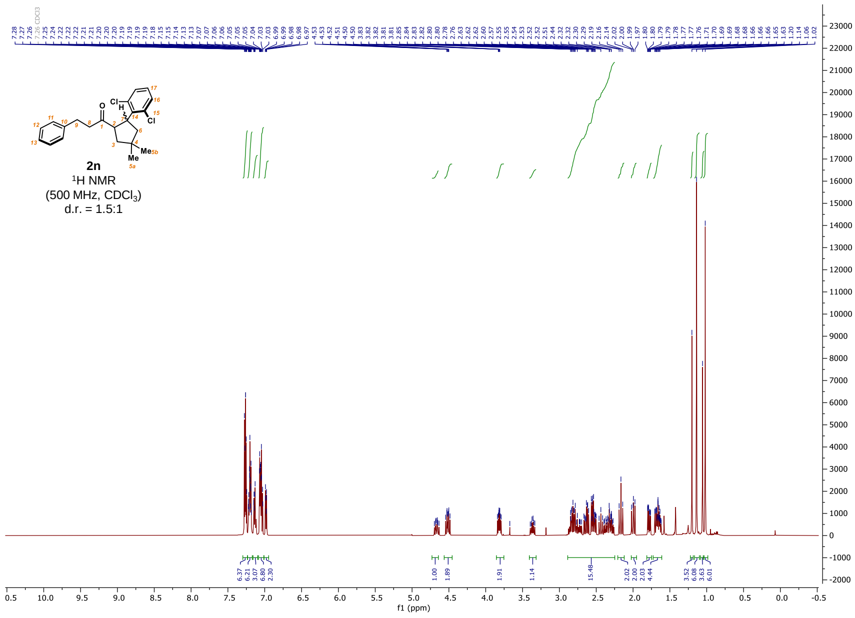
2n
^{1}H NMR
(500 MHz, CDCl$_3$)
d.r. = 1.5:1
f1 (ppm)

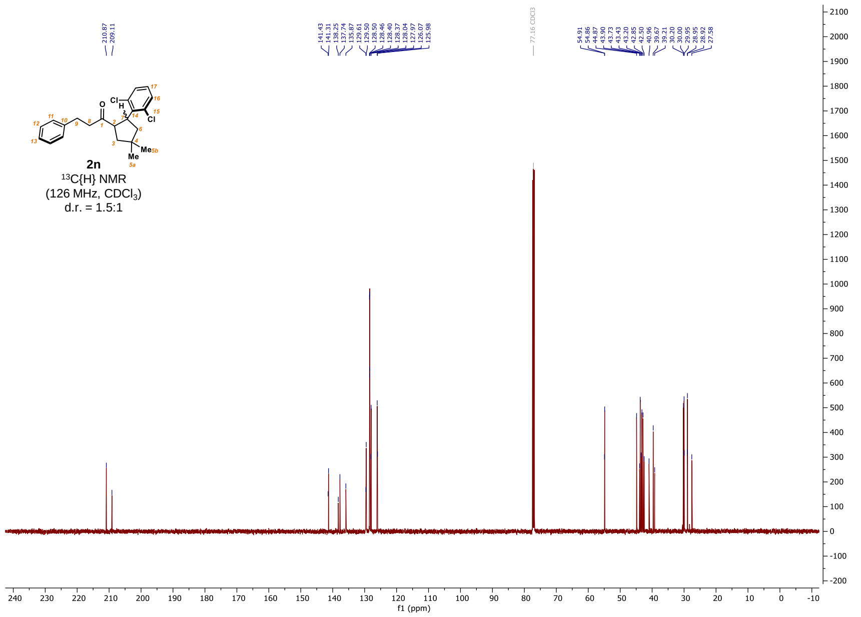
2n
$^{13}C\{H\}$ NMR
(126 MHz, $CDCl_3$)
d.r. = 1.5:1
f1 (ppm)

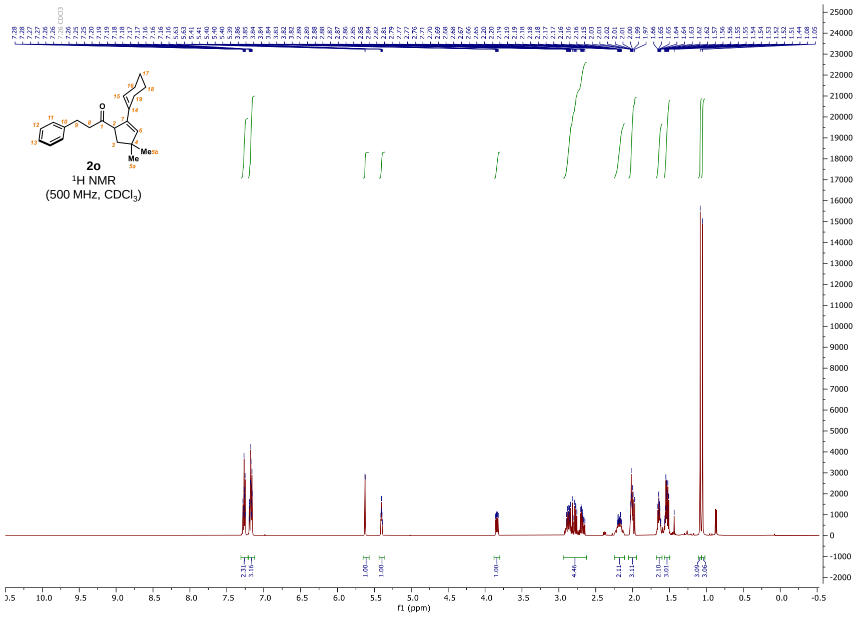
2o
^{1}H NMR
(500 MHz, $CDCl_3$)
f1 (ppm)

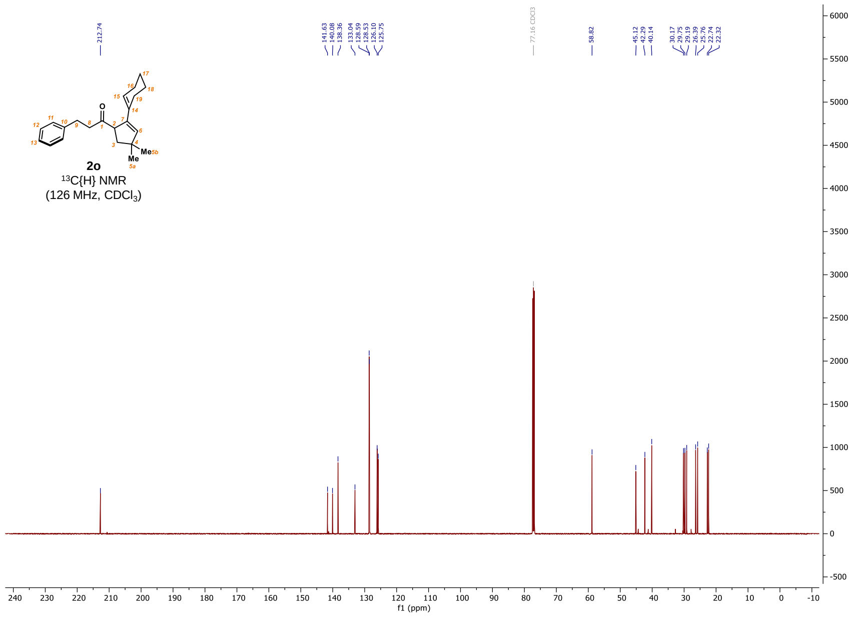
2o
^{13}C{H} NMR
(126 MHz, CDCl$_3$)
f1 (ppm)

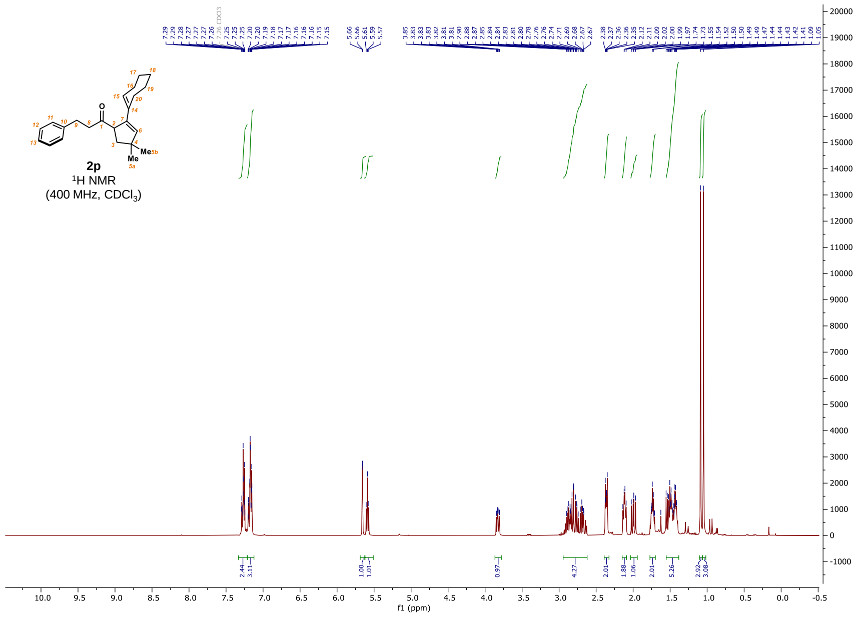
2p
^{1}H NMR
(400 MHz, CDCl$_3$)
f1 (ppm)

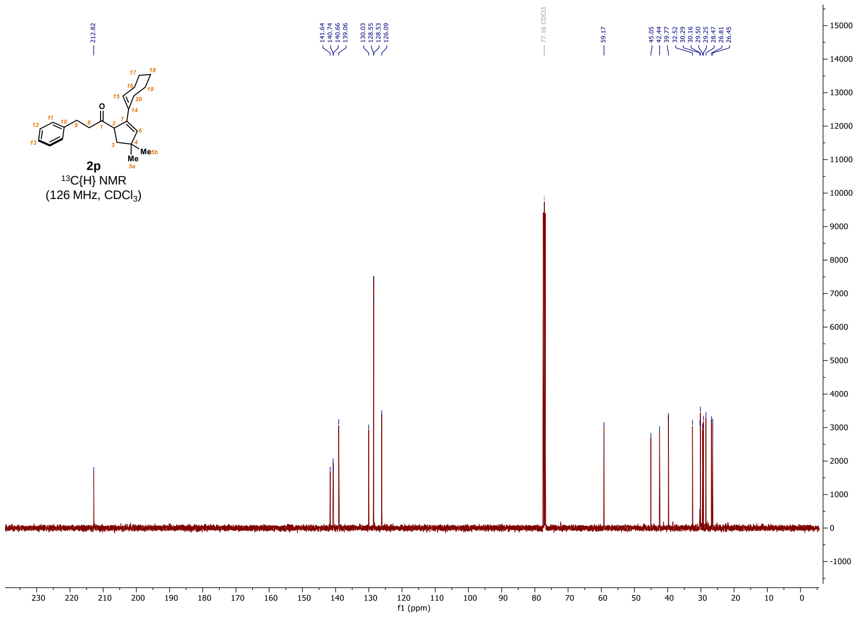
2p
13C{H} NMR
(126 MHz, CDCl3)
212.82
141.64
140.74
140.66
139.06
130.03
128.55
128.53
126.09
77.16 CDCl3
59.17
45.05
42.44
39.77
32.52
30.29
30.16
29.50
29.25
28.47
26.81
26.45
f1 (ppm)

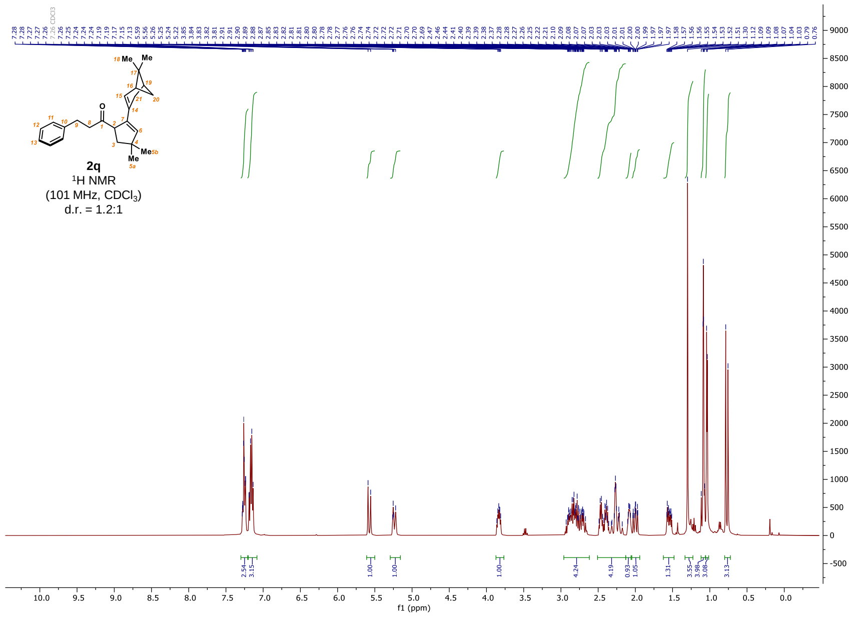
2q
1H NMR
(101 MHz, CDCl3)
d.r. = 1.2:1
f1 (ppm)

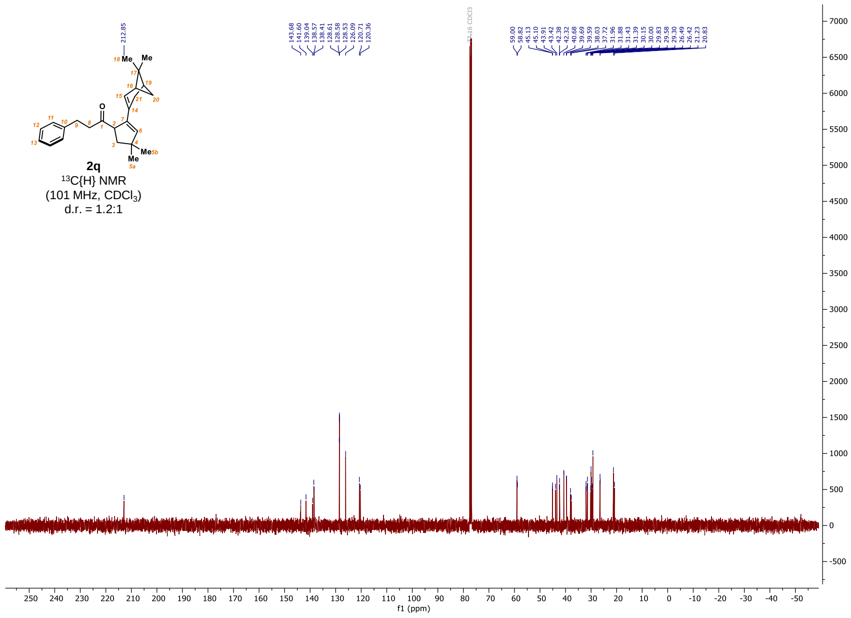
2q
13C{H} NMR
(101 MHz, CDCl3)
d.r. = 1.2:1
f1 (ppm)

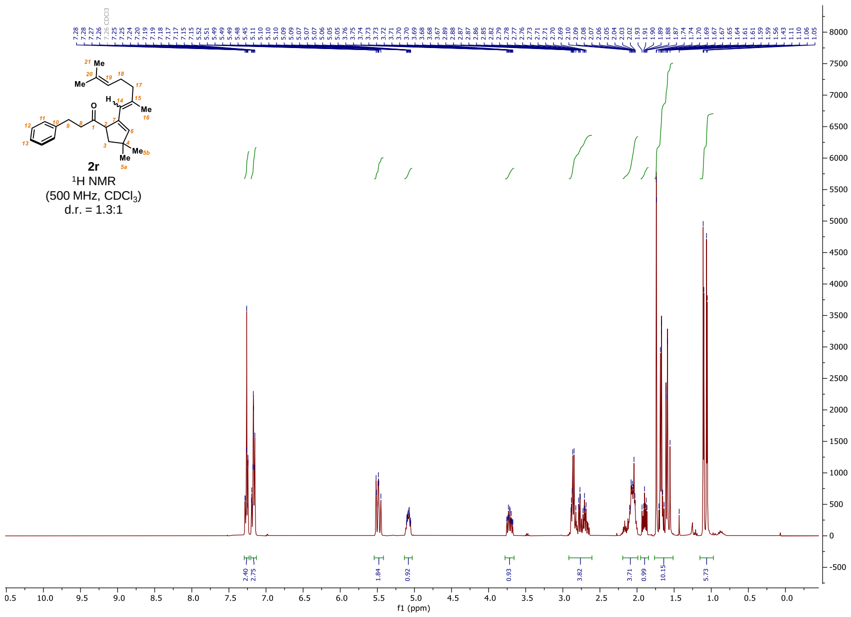
2r
1H NMR
(500 MHz, CDCl3)
d.r. = 1.3:1
f1 (ppm)

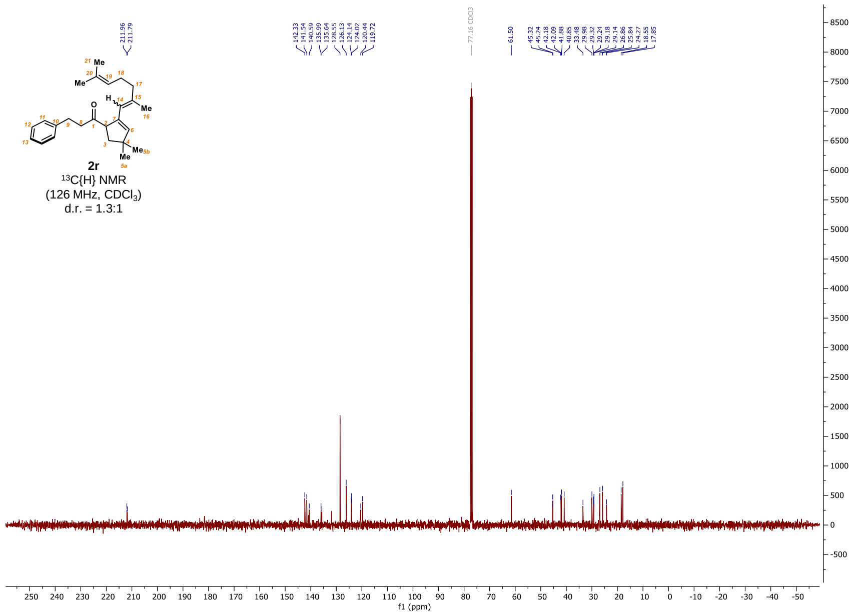

2r
$^{13}C\{H\}$ NMR
(126 MHz, $CDCl_3$)
d.r. = 1.3:1

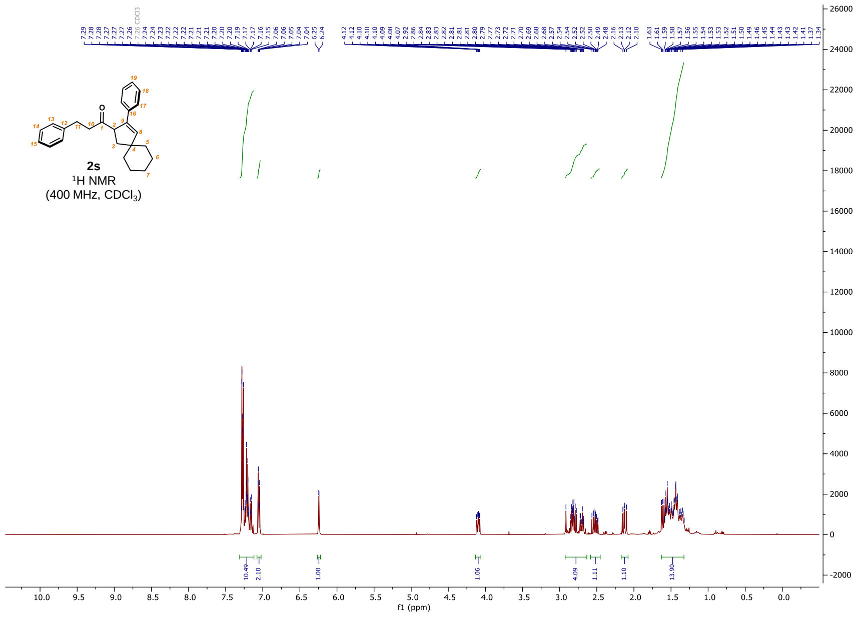

2s
^1H NMR
(400 MHz, $CDCl_3$)

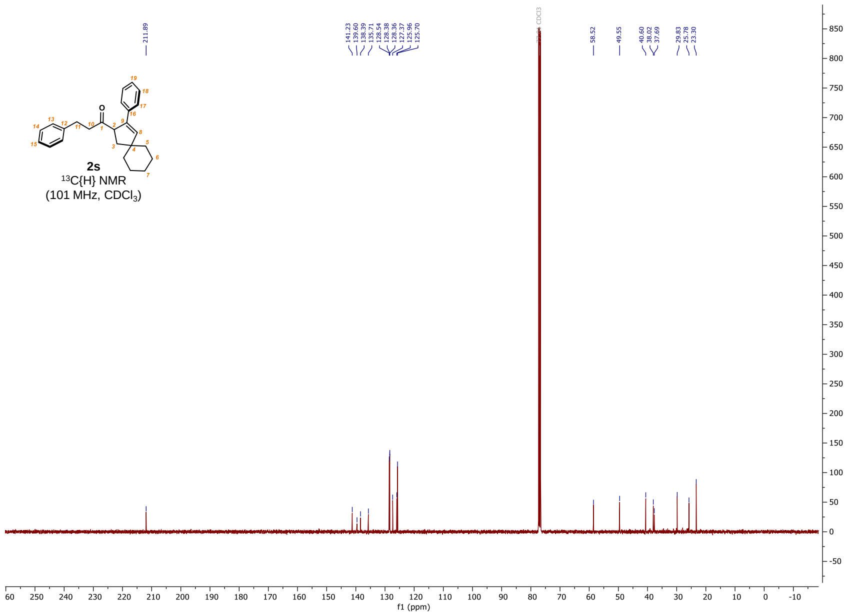

2s
^{13}C{H} NMR
(101 MHz, CDCl$_3$)
211.89
141.23
139.60
138.39
135.71
128.54
128.38
128.36
127.37
125.96
125.70
58.52
49.55
40.60
38.02
37.69
29.83
25.78
23.30
f1 (ppm)

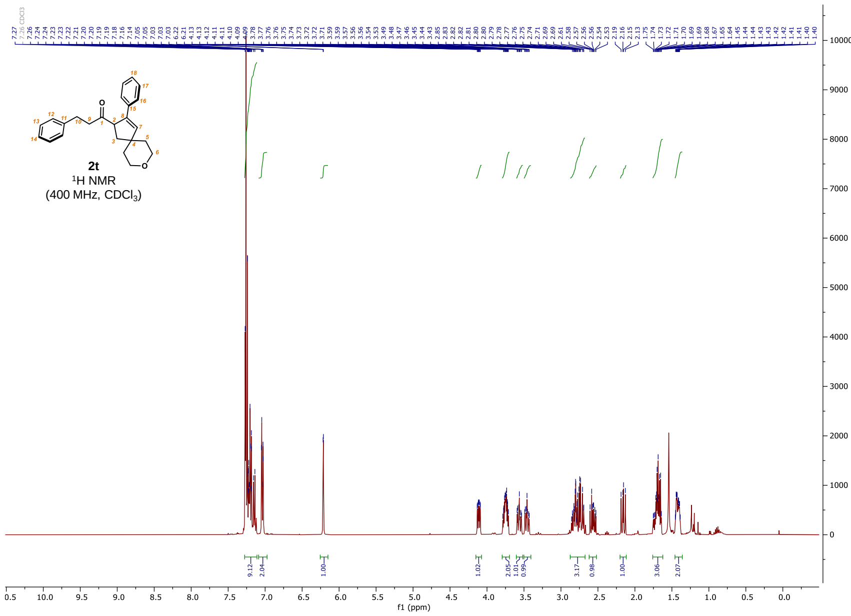

2t
^{1}H NMR
(400 MHz, CDCl$_3$)
f1 (ppm)

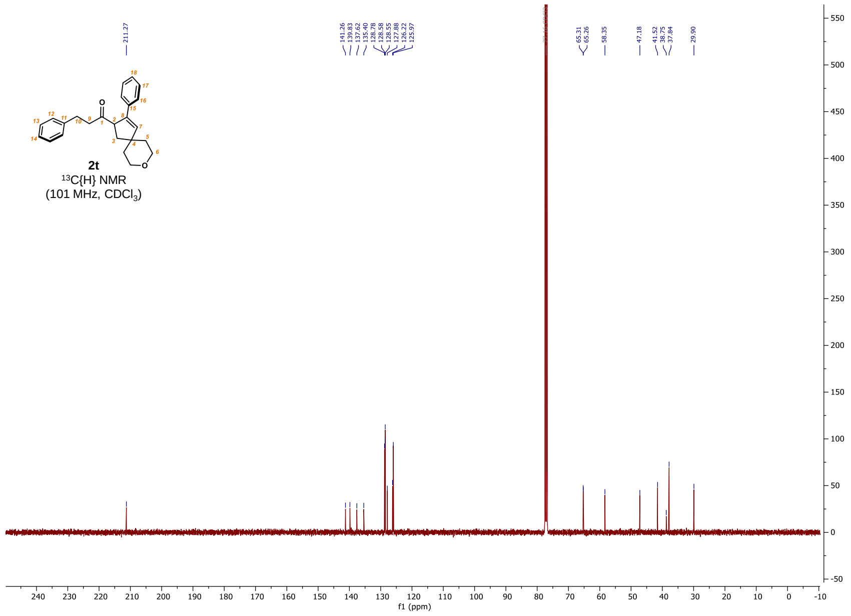
2t
^{13}C{H} NMR
(101 MHz, CDCl$_3$)
f1 (ppm)

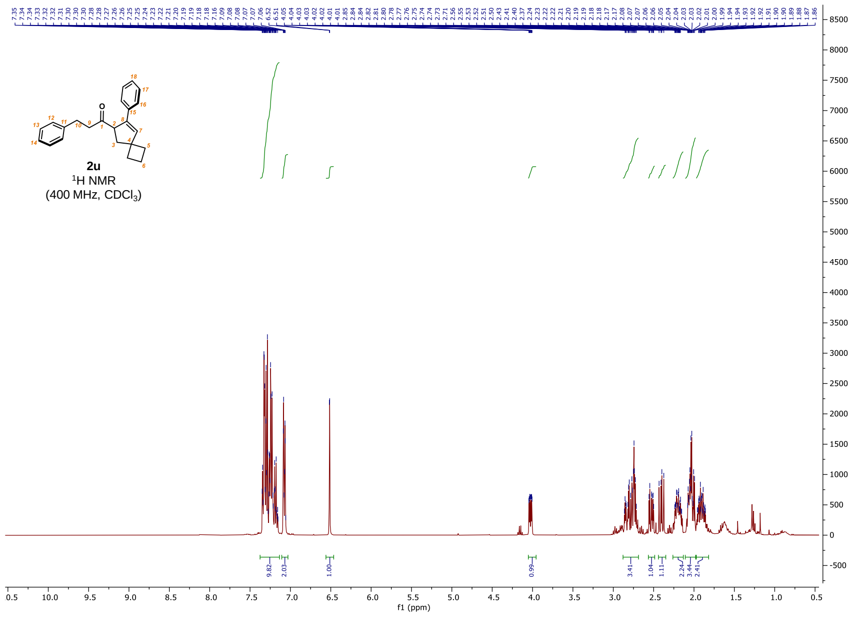
2u
^{1}H NMR
(400 MHz, CDCl$_3$)
f1 (ppm)

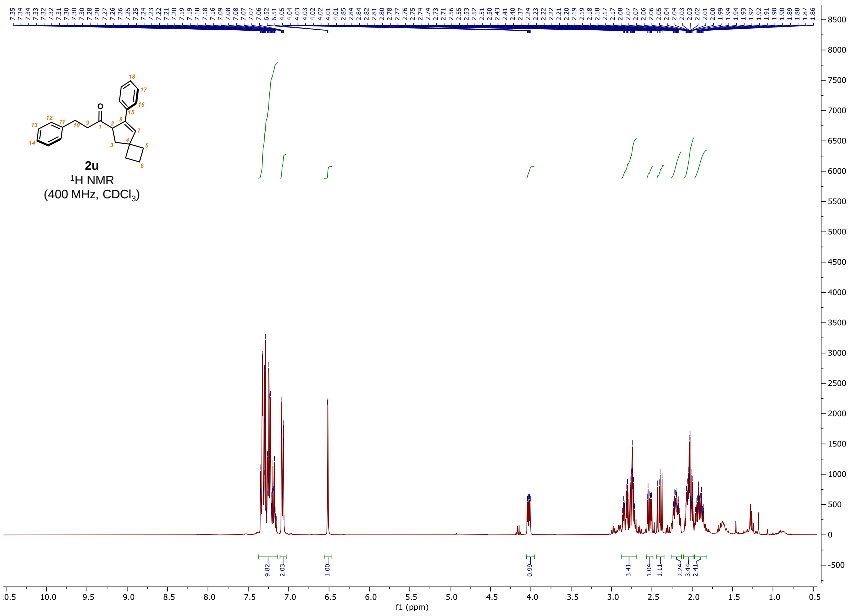
2u
^{1}H NMR
(400 MHz, CDCl$_3$)
f1 (ppm)

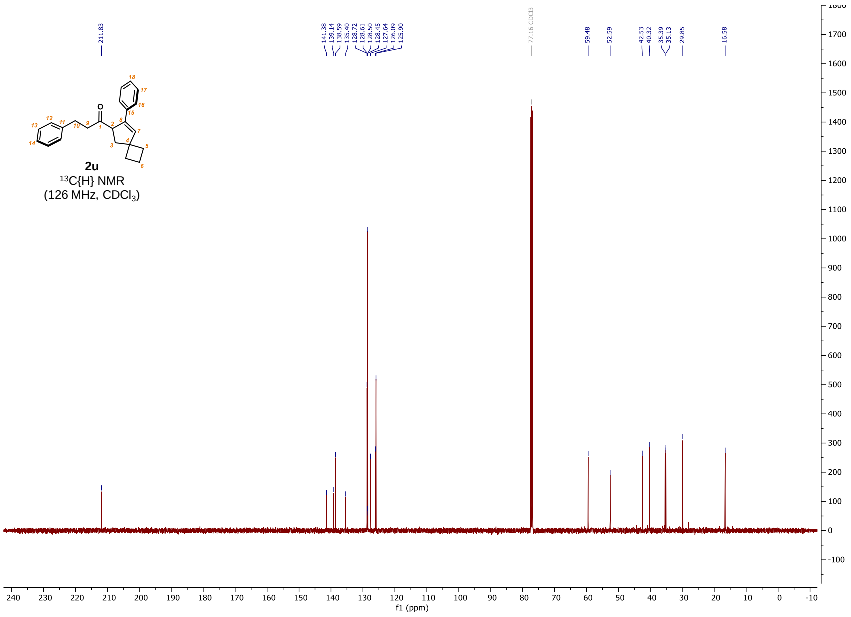
2u
^{13}C{H} NMR
(126 MHz, CDCl$_3$)
211.83
141.38
139.14
138.59
135.40
128.72
128.61
128.50
128.45
127.64
126.09
125.90
77.16 CDCl3
59.48
52.59
42.53
40.32
35.39
35.13
29.85
16.58
f1 (ppm)

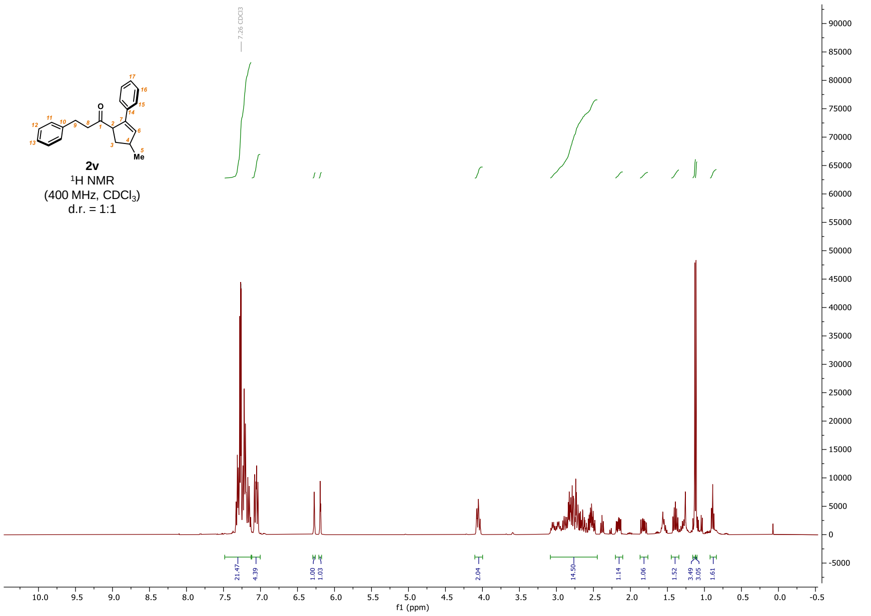
2v
^{1}H NMR
(400 MHz, $CDCl_3$)
d.r. = 1:1
f1 (ppm)

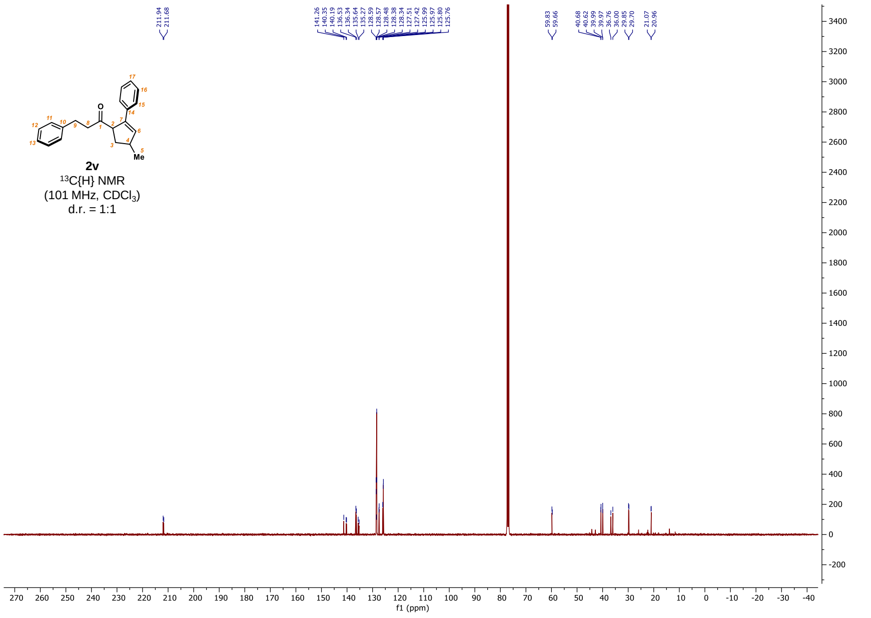
2v
^{13}C{H} NMR
(101 MHz, $CDCl_3$)
d.r. = 1:1
f1 (ppm)

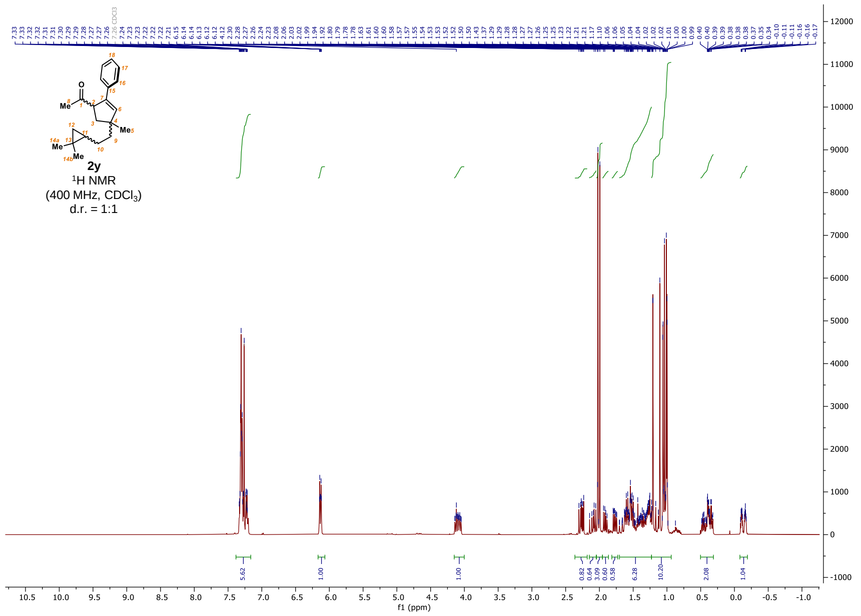
2y
¹H NMR
(400 MHz, CDCl3)
d.r. = 1:1
f1 (ppm)

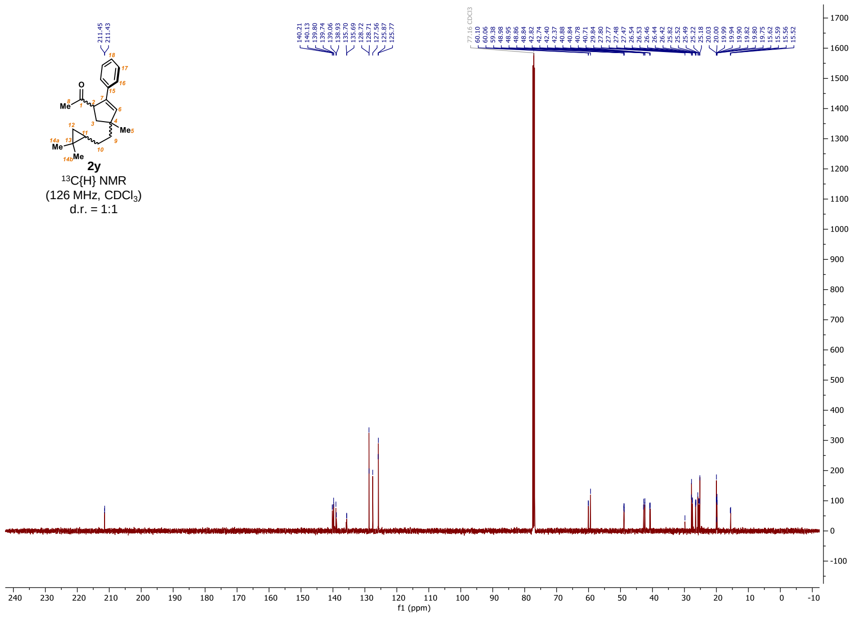
2y
¹³C{H} NMR
(126 MHz, CDCl3)
d.r. = 1:1
f1 (ppm)

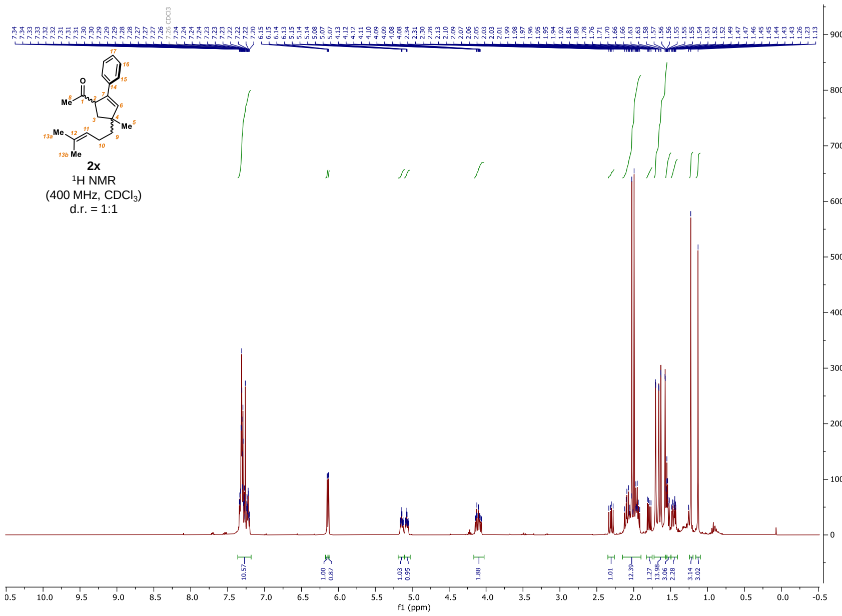

2x

^{1}H NMR (400 MHz, $CDCl_3$)

d.r. = 1:1

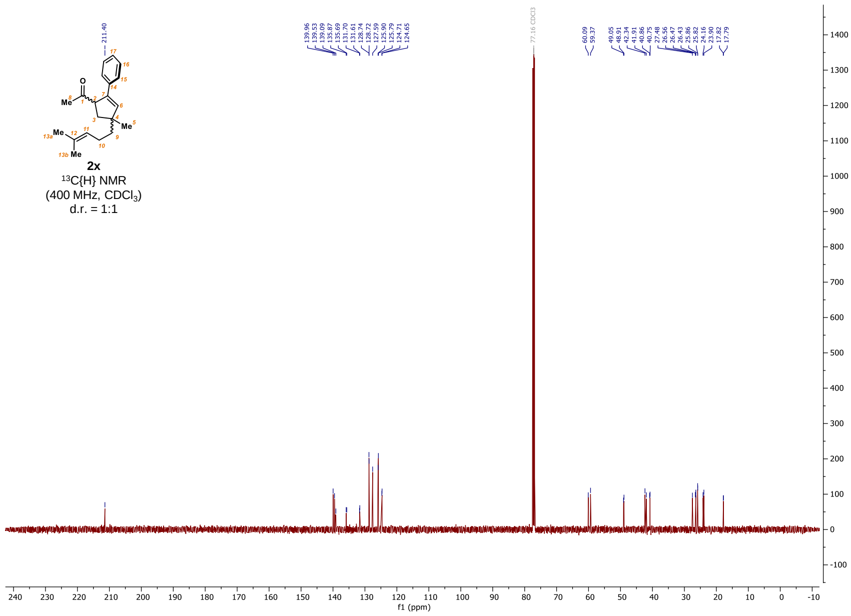

2x

^{13}C{H} NMR (400 MHz, $CDCl_3$)

d.r. = 1:1

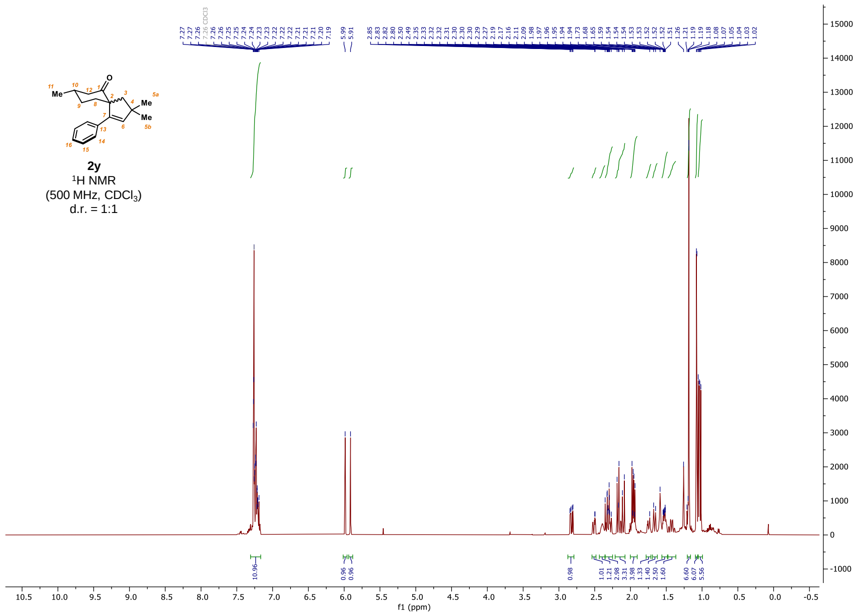
2y
^{1}H NMR
(500 MHz, $CDCl_3$)
d.r. = 1:1
f1 (ppm)

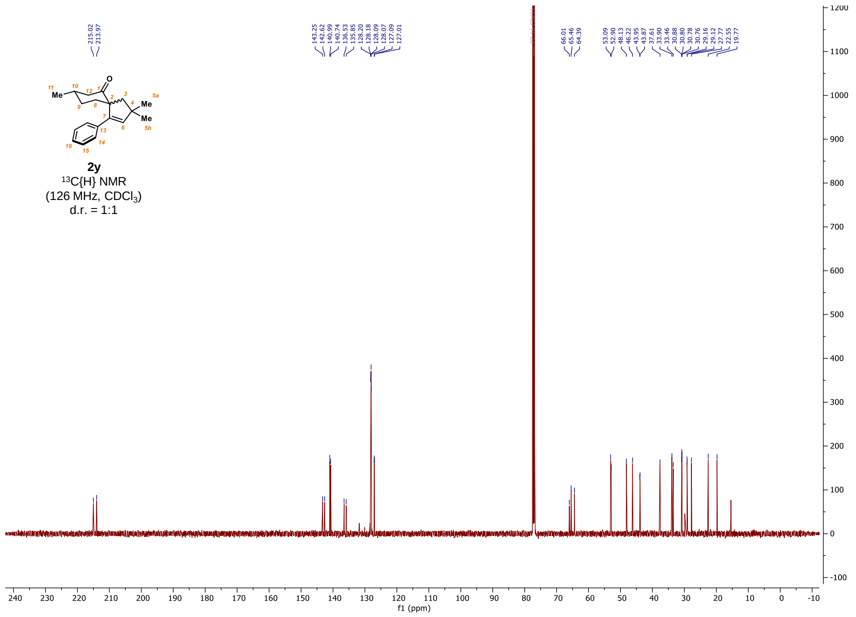
2y
^{13}C{H} NMR
(126 MHz, $CDCl_3$)
d.r. = 1:1
f1 (ppm)

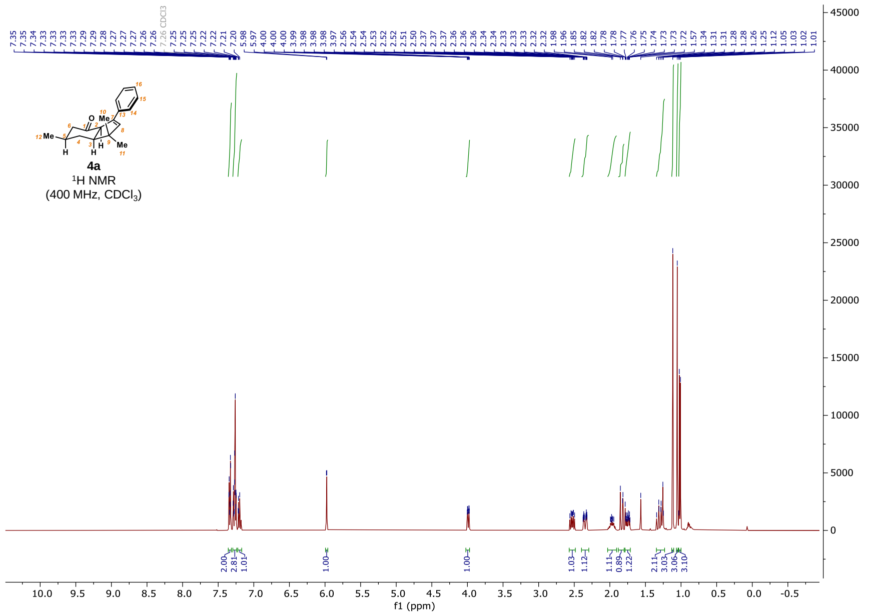
4a
1H NMR
(400 MHz, CDCl3)
f1 (ppm)

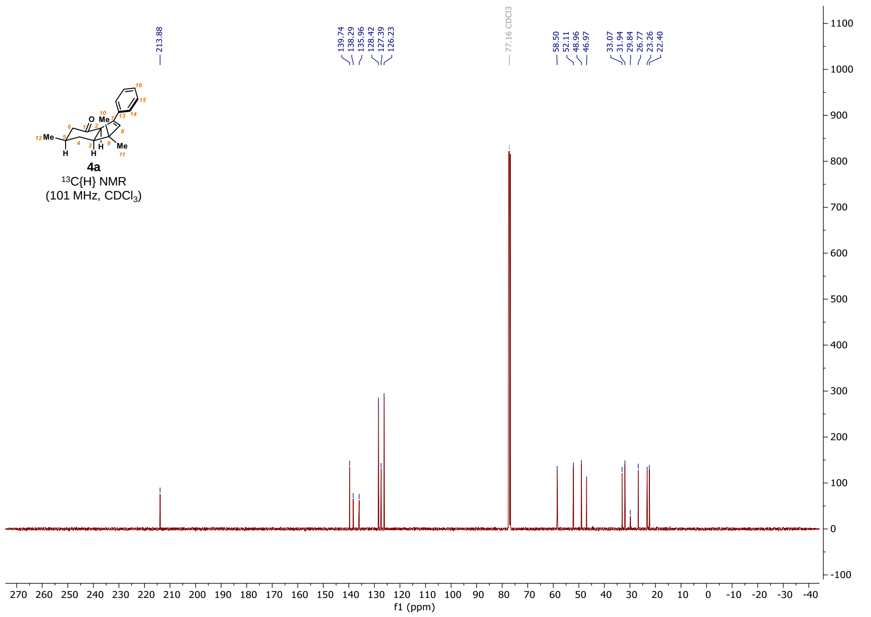
213.88
139.74
138.29
135.96
128.42
127.39
126.23
77.16 CDCl3
58.50
52.11
48.96
46.97
33.07
31.94
29.84
26.77
23.26
22.40
4a
13C{H} NMR
(101 MHz, CDCl3)
f1 (ppm)

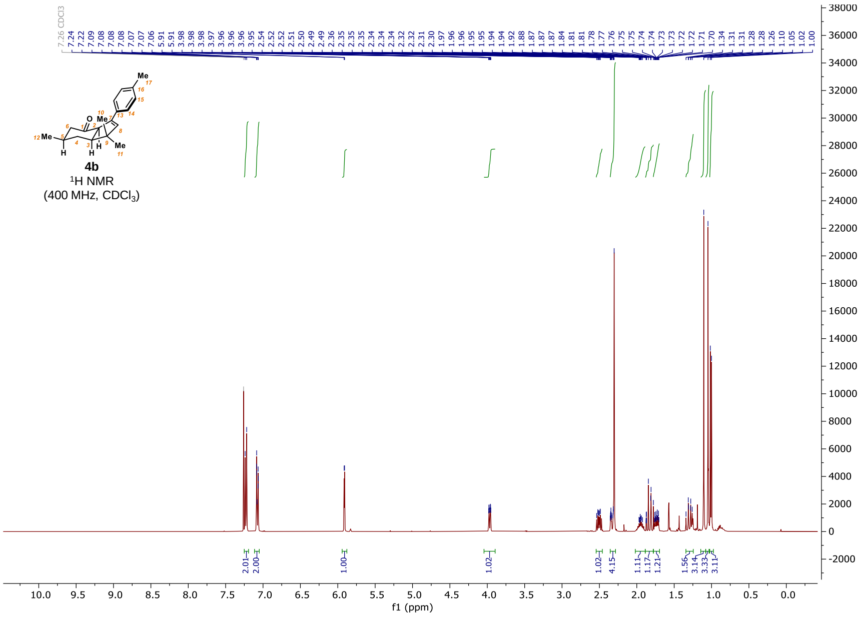
4b
^{1}H NMR
(400 MHz, $CDCl_3$)
f1 (ppm)

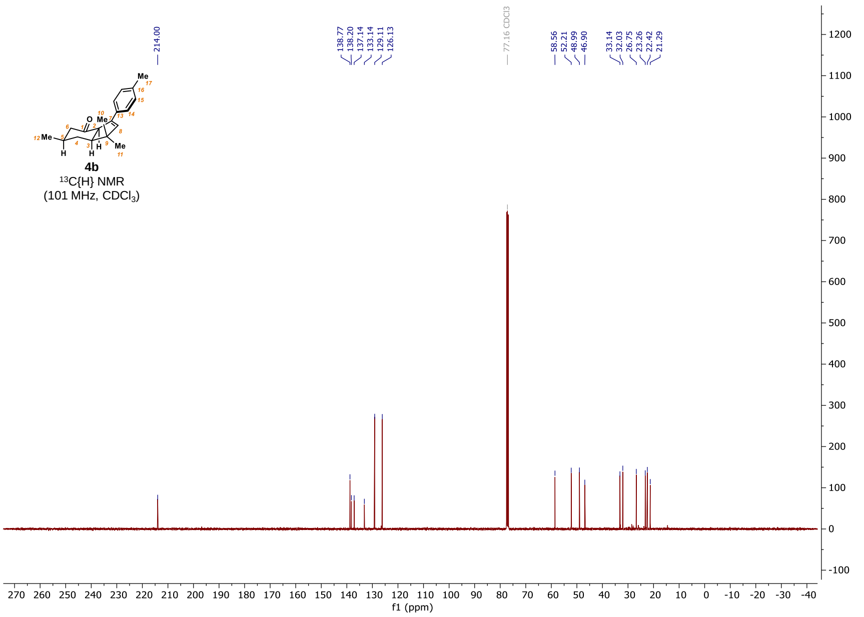
4b
^{13}C{H} NMR
(101 MHz, $CDCl_3$)
f1 (ppm)

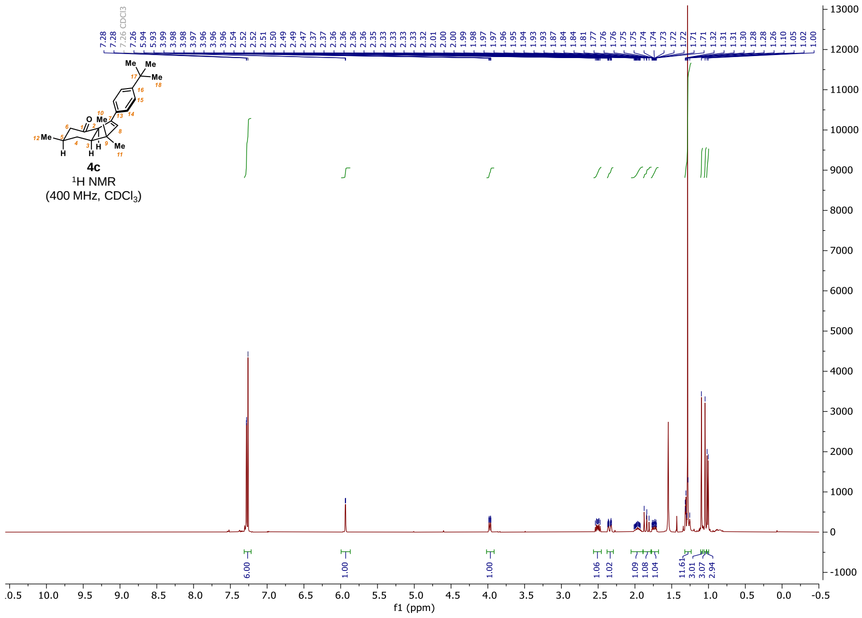
4c
^{1}H NMR
(400 MHz, CDCl$_3$)
f1 (ppm)

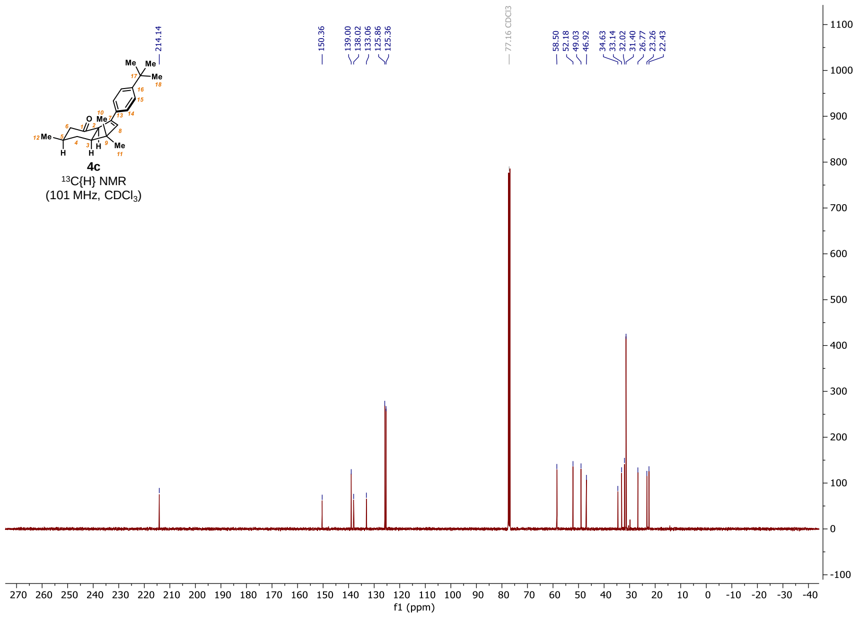
214.14
150.36
139.00
138.02
133.06
125.86
125.36
77.16 CDCl3
58.50
52.18
49.03
46.92
34.63
33.14
32.02
31.40
26.77
23.26
22.43
4c
^{13}C{H} NMR
(101 MHz, CDCl$_3$)
f1 (ppm)

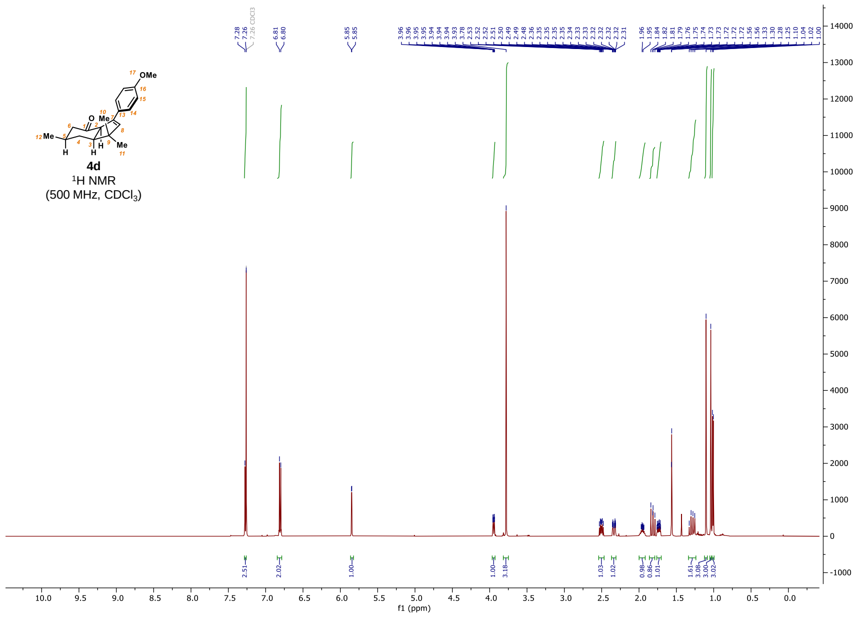
4d
^{1}H NMR
(500 MHz, CDCl$_3$)
f1 (ppm)

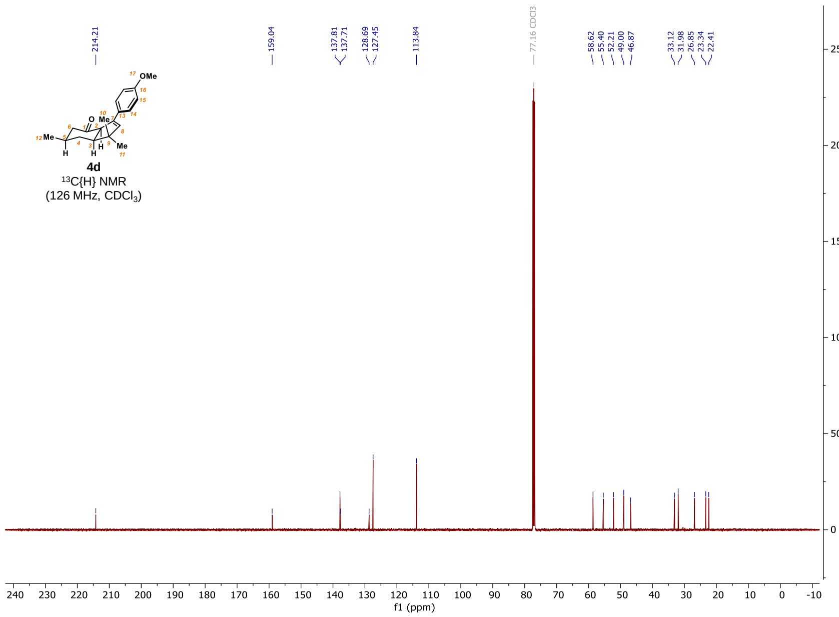
214.21
159.04
137.81
137.71
128.69
127.45
113.84
77.16 CDCl3
58.62
55.40
52.21
49.00
46.87
33.12
31.98
26.85
23.34
22.41
4d
^{13}C{H} NMR
(126 MHz, CDCl$_3$)
f1 (ppm)

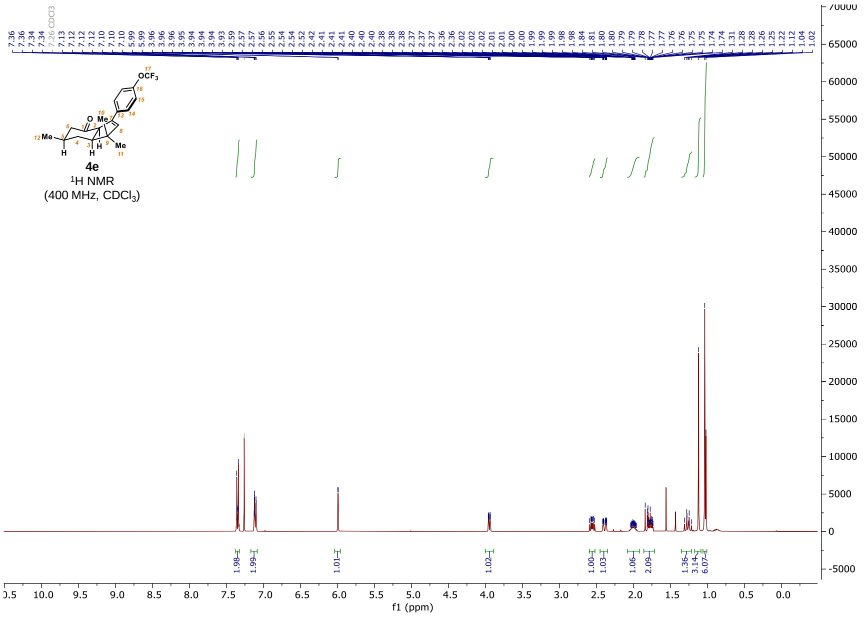
4e
^{1}H NMR
(400 MHz, CDCl$_3$)
f1 (ppm)

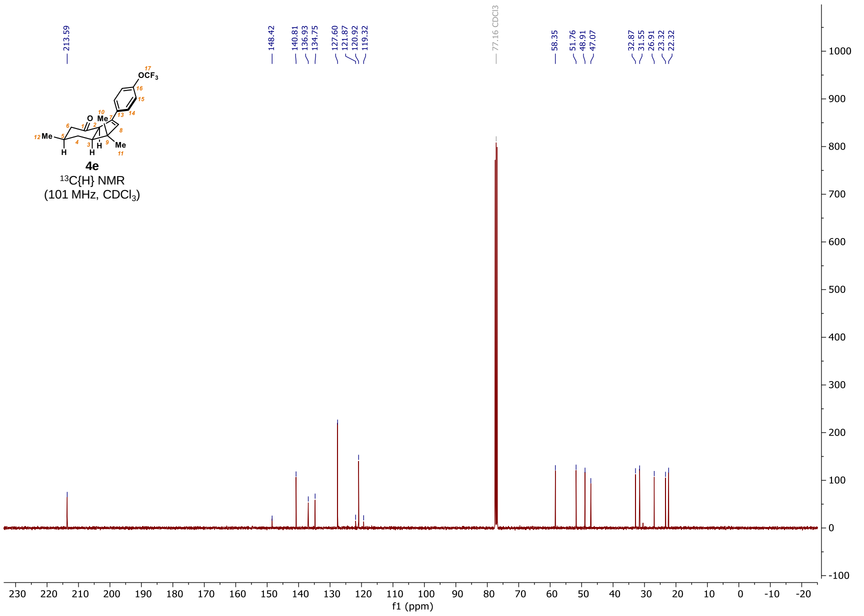
4e
^{13}C{H} NMR
(101 MHz, CDCl$_3$)
213.59
148.42
140.81
136.93
134.75
127.60
121.87
120.92
119.32
77.16 CDCl3
58.35
51.76
48.91
47.07
32.87
31.55
26.91
23.32
22.32
f1 (ppm)

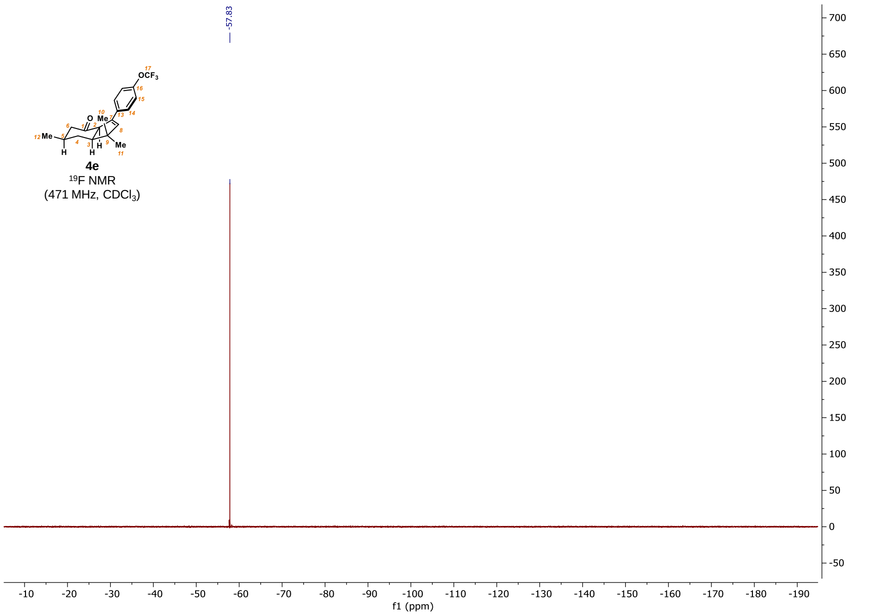
-57.83
OCF3
Me
O
H
4e
19F NMR
(471 MHz, CDCl3)
f1 (ppm)

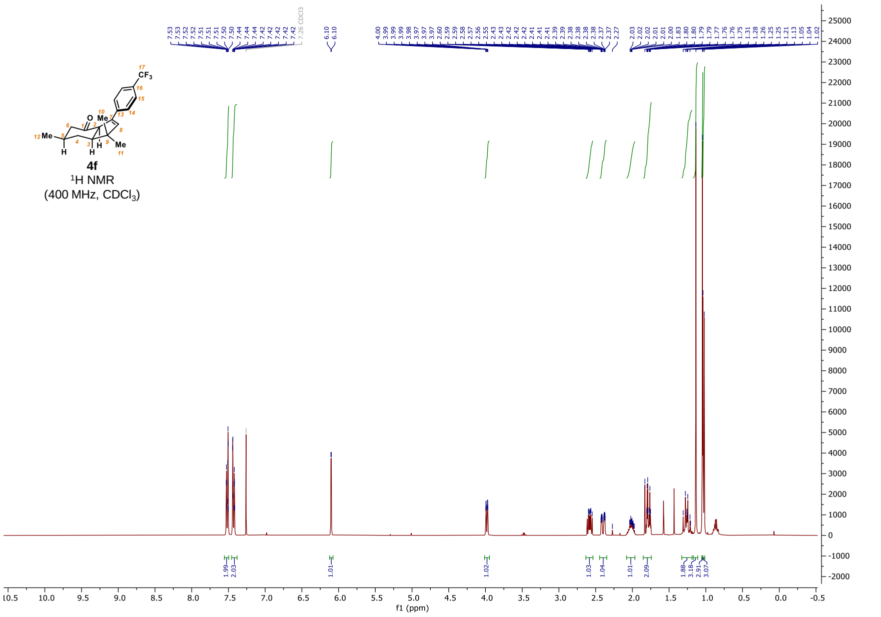
CF3
Me
O
H
4f
1H NMR
(400 MHz, CDCl3)
f1 (ppm)

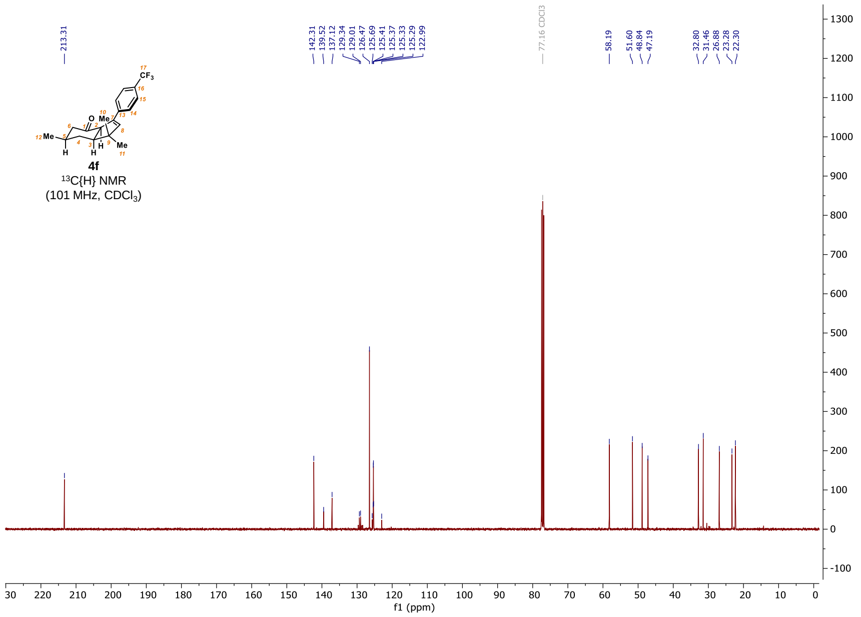
213.31
142.31
139.52
137.12
129.34
129.01
126.47
125.69
125.41
125.37
125.33
125.29
122.99
77.16 CDCl3
58.19
51.60
48.84
47.19
32.80
31.46
26.88
23.28
22.30
4f
13C{H} NMR
(101 MHz, CDCl3)
f1 (ppm)

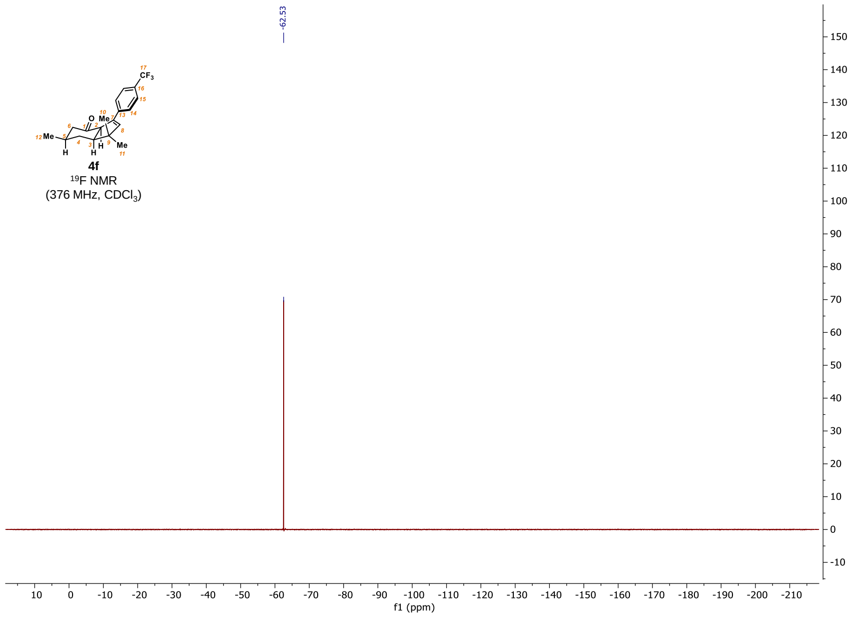
-62.53
4f
19F NMR
(376 MHz, CDCl3)
f1 (ppm)

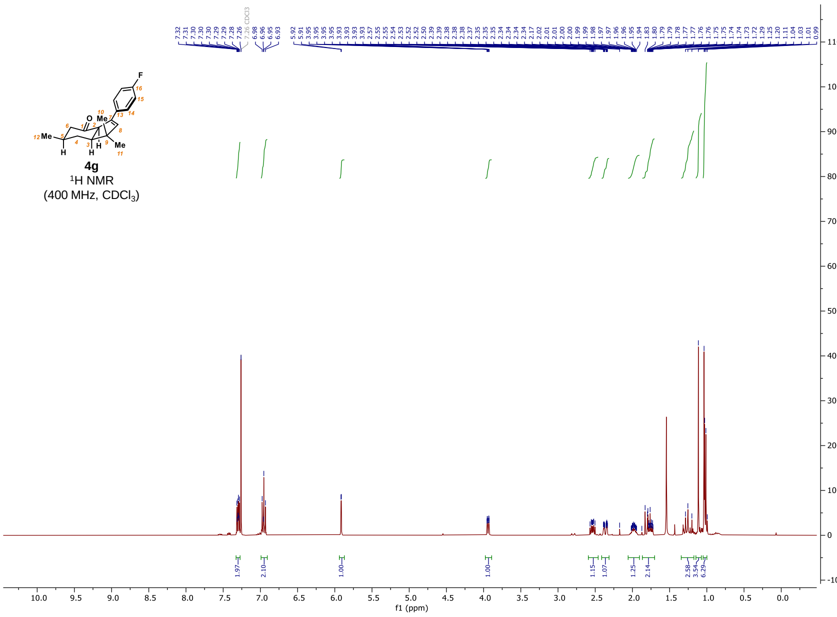
4g
1H NMR
(400 MHz, CDCl3)
f1 (ppm)

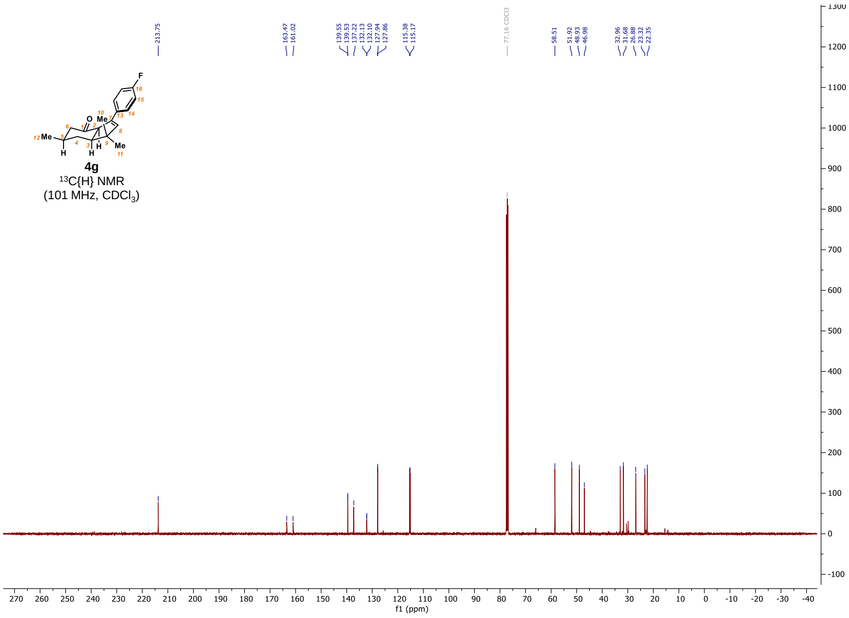
4g
13C{H} NMR
(101 MHz, CDCl3)
f1 (ppm)

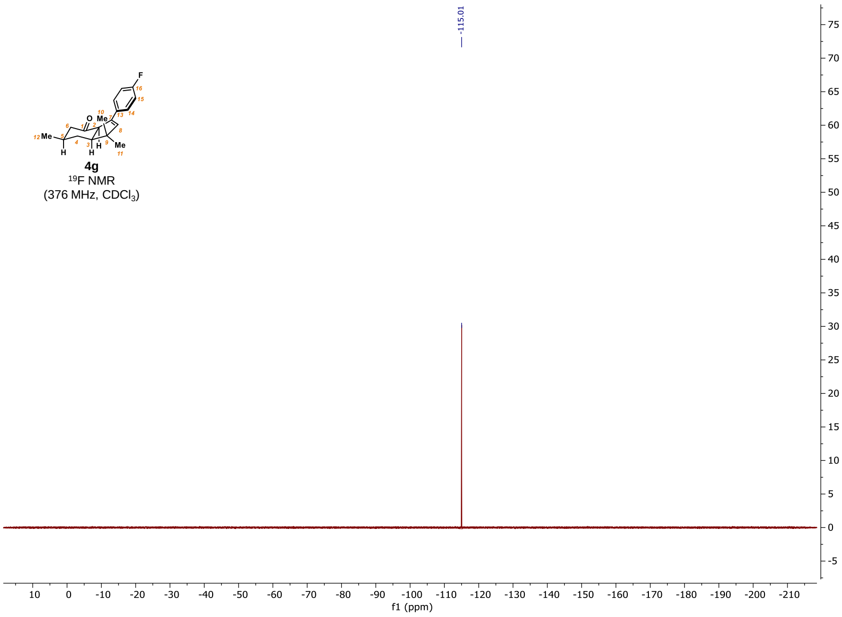
4g
^{19}F NMR
(376 MHz, CDCl$_3$)
-115.01
f1 (ppm)

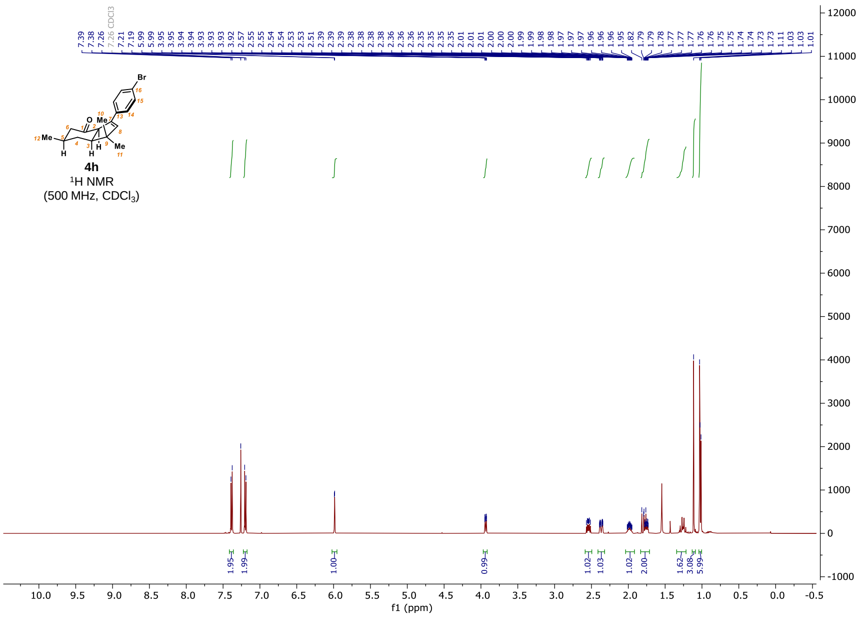
4h
^{1}H NMR
(500 MHz, CDCl$_3$)
f1 (ppm)

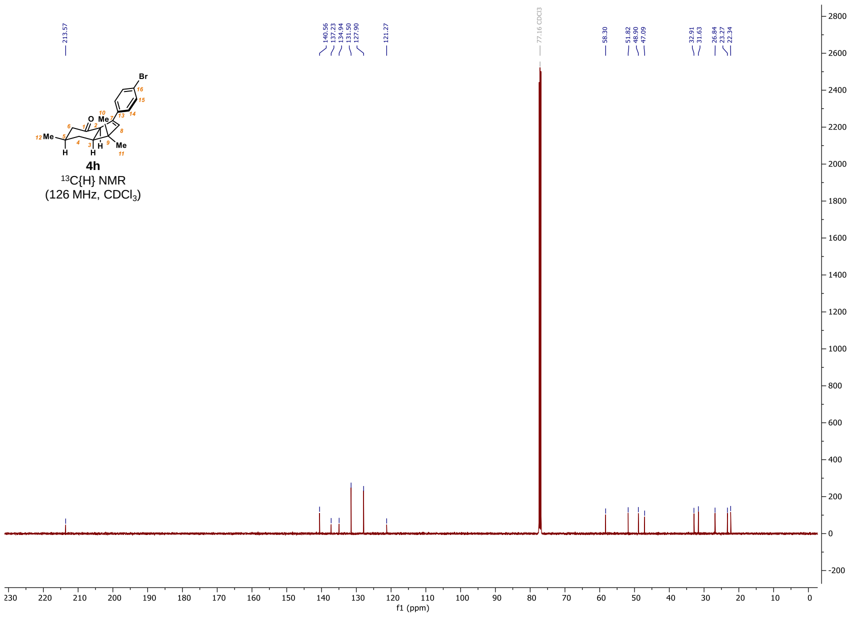
4h
13C{H} NMR
(126 MHz, CDCl3)
213.57
140.56
137.23
134.94
131.50
127.90
121.27
77.16 CDCl3
58.30
51.82
48.90
47.09
32.91
31.63
26.84
23.27
22.34
f1 (ppm)

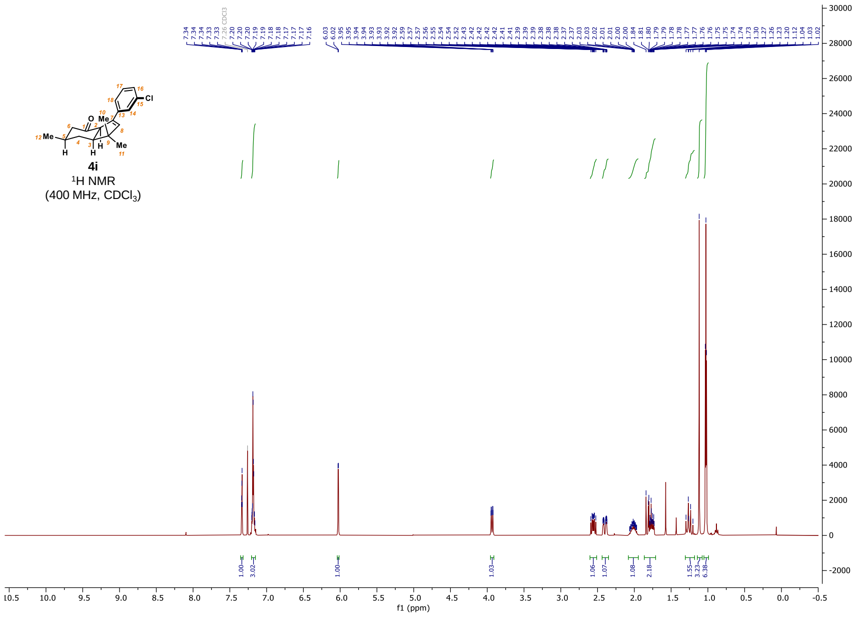
4i
1H NMR
(400 MHz, CDCl3)
f1 (ppm)

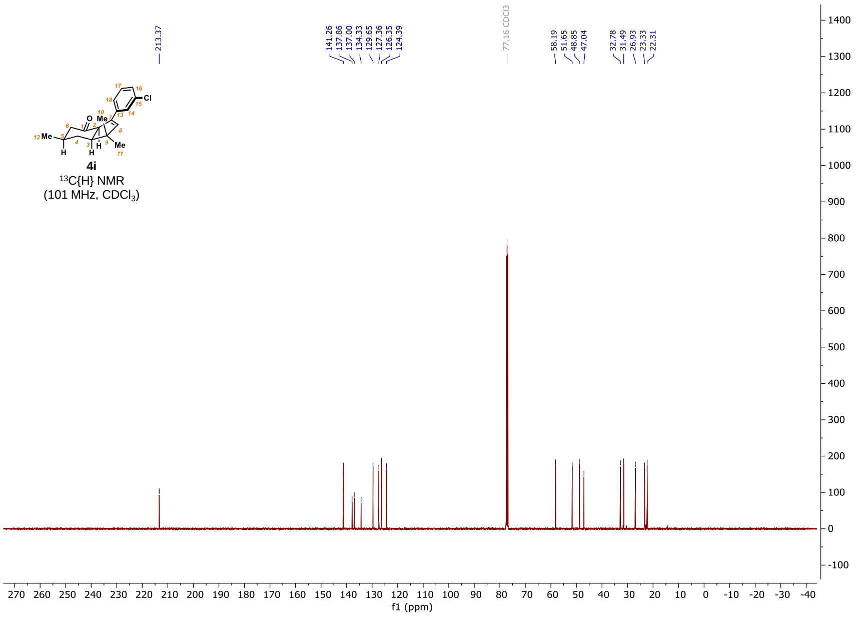
213.37
141.26
137.86
137.00
134.33
129.65
127.36
126.35
124.39
77.16 CDCl3
58.19
51.65
48.85
47.04
32.78
31.49
26.93
23.33
22.31
4i
13C{H} NMR
(101 MHz, CDCl3)
f1 (ppm)

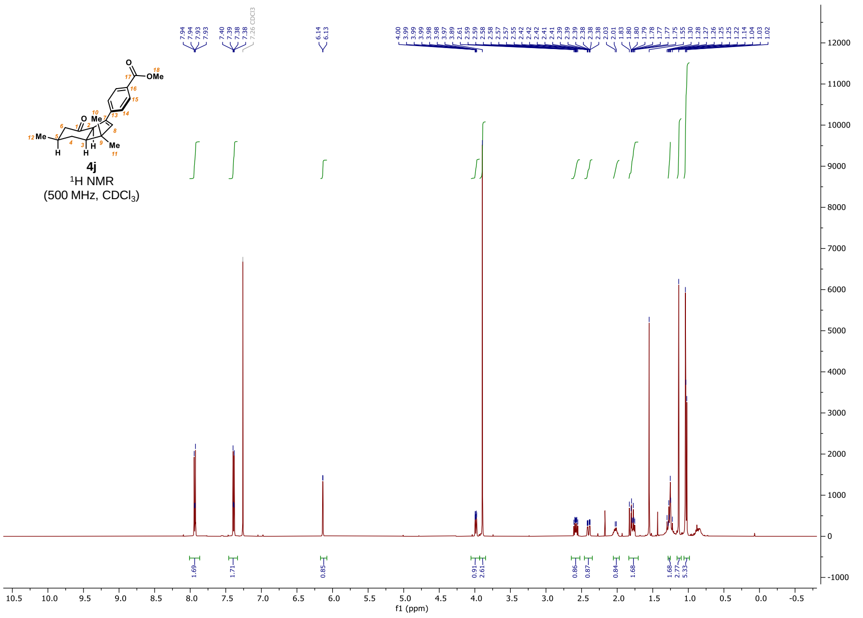
4j
1H NMR
(500 MHz, CDCl3)
f1 (ppm)

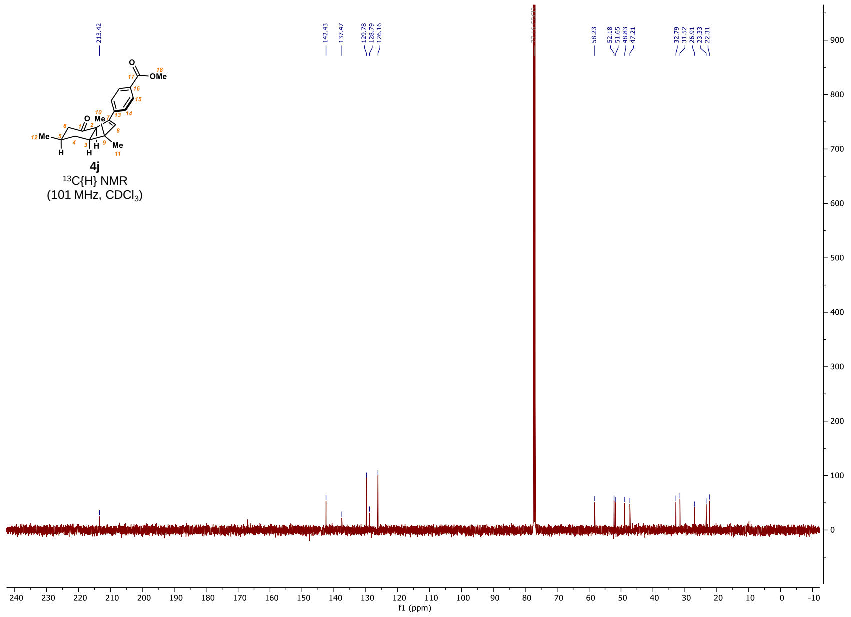
4j
13C{H} NMR
(101 MHz, CDCl3)
213.42
142.43
137.47
129.78
128.79
126.16
58.23
52.18
51.65
48.83
47.21
32.79
31.52
26.91
23.33
22.31
f1 (ppm)

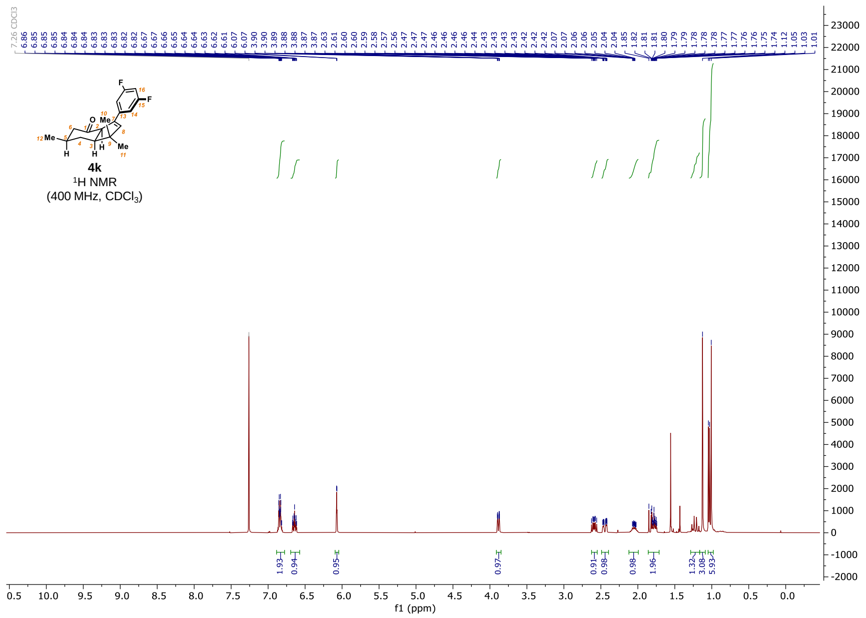
4k
1H NMR
(400 MHz, CDCl3)
1.93
0.94
0.95
0.97
0.91
0.98
0.98
1.96
1.32
3.08
5.93
f1 (ppm)

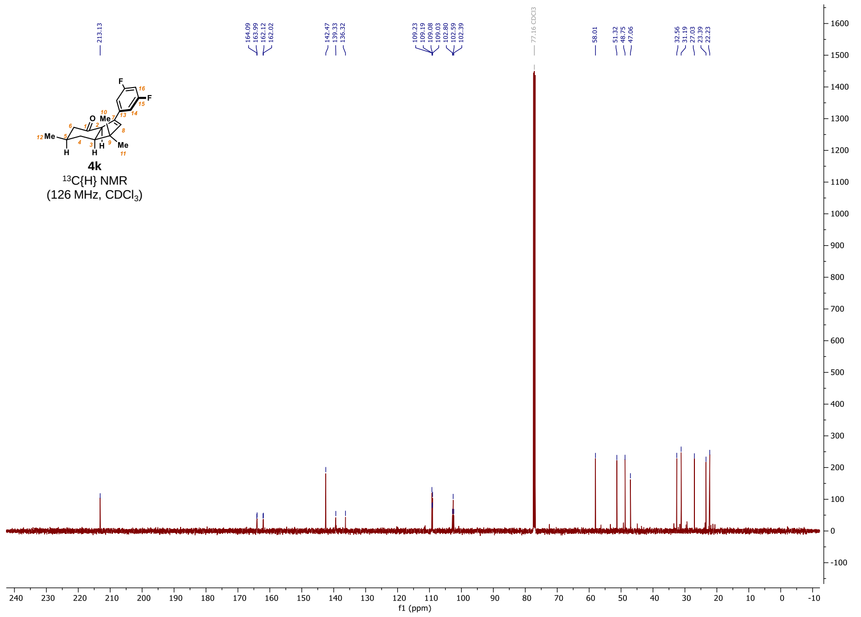
213.13
164.09
163.99
162.12
162.02
142.47
139.33
136.32
109.23
109.19
109.08
109.03
102.80
102.59
102.39
77.16 CDCl3
58.01
51.32
48.75
47.06
32.56
31.19
27.03
23.39
22.23
4k
13C{H} NMR
(126 MHz, CDCl3)
f1 (ppm)

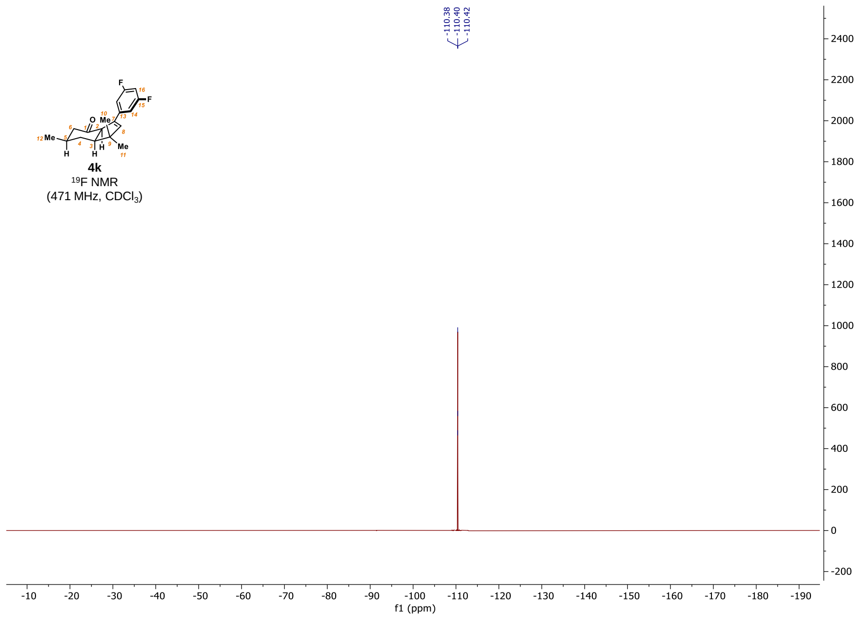
-110.38
-110.40
-110.42
4k
19F NMR
(471 MHz, CDCl3)
f1 (ppm)

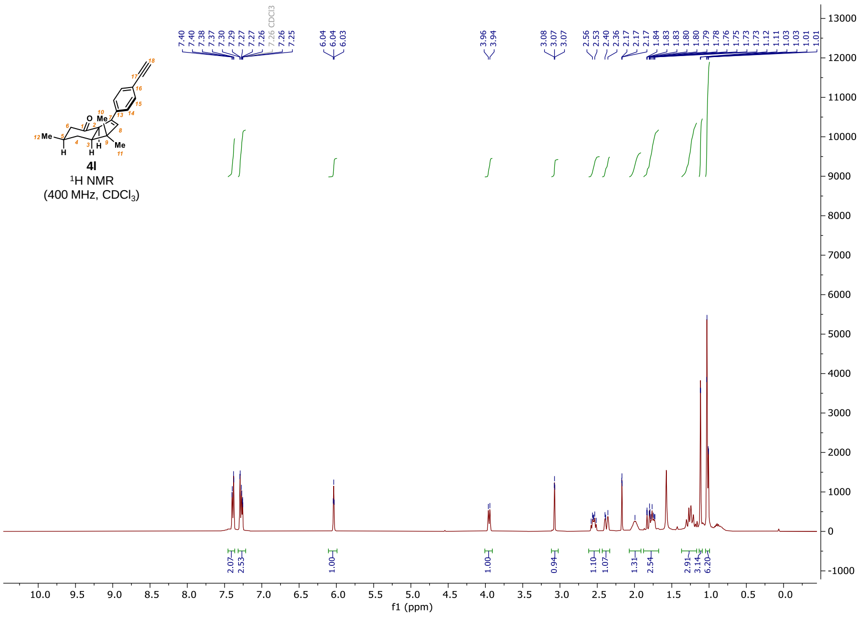

4l
^{1}H NMR
(400 MHz, $CDCl_3$)

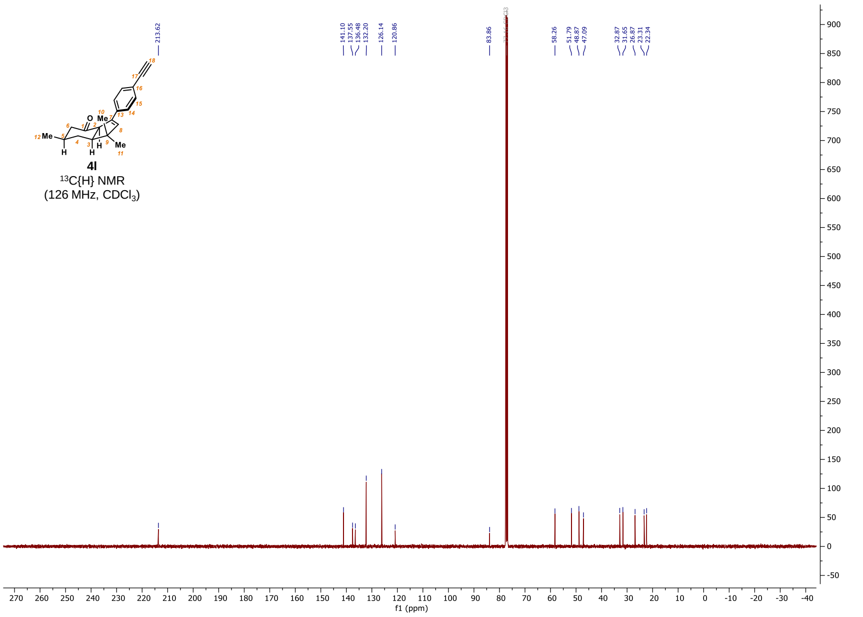

4l
^{13}C{H} NMR
(126 MHz, $CDCl_3$)

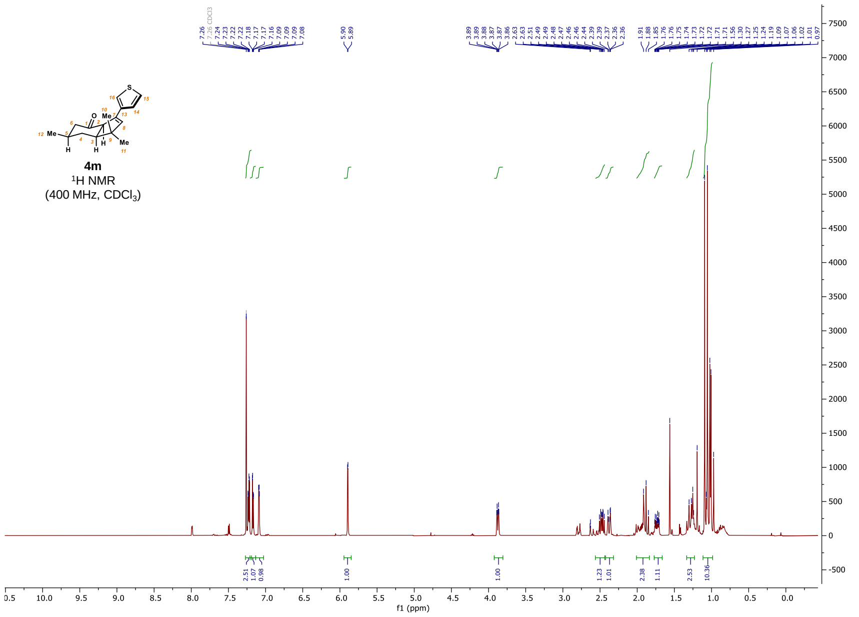
4m
^{1}H NMR
(400 MHz, CDCl$_3$)
f1 (ppm)

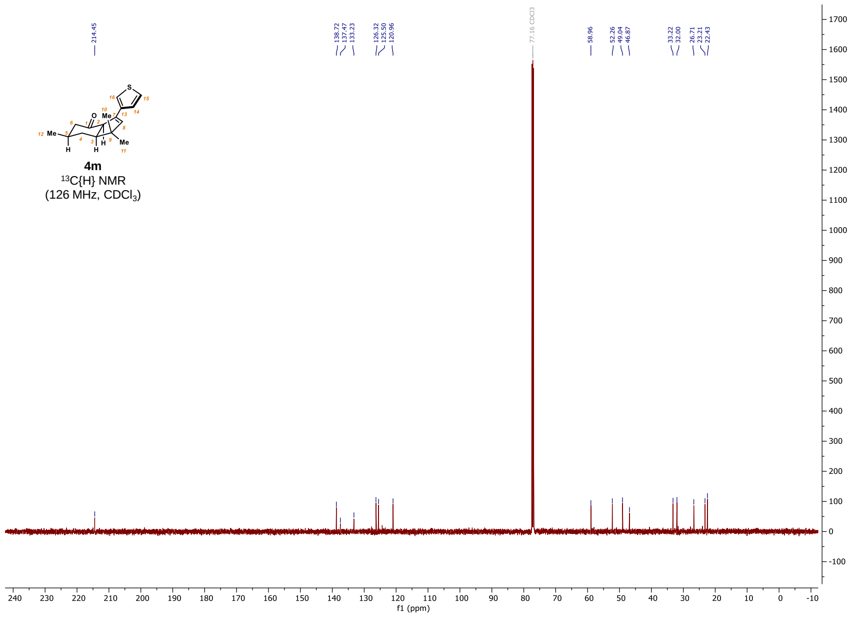
4m
^{13}C{H} NMR
(126 MHz, CDCl$_3$)
f1 (ppm)

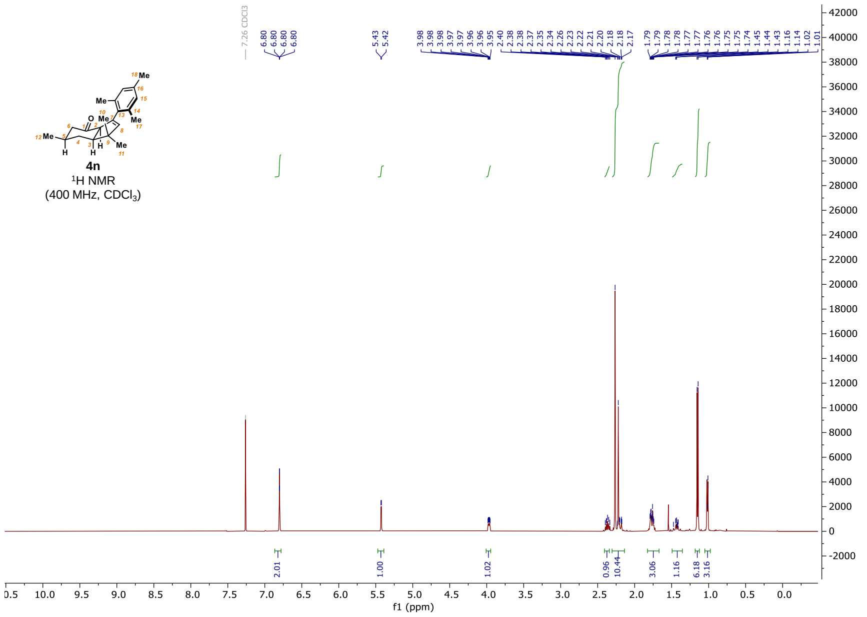
4n
1H NMR
(400 MHz, CDCl3)
f1 (ppm)

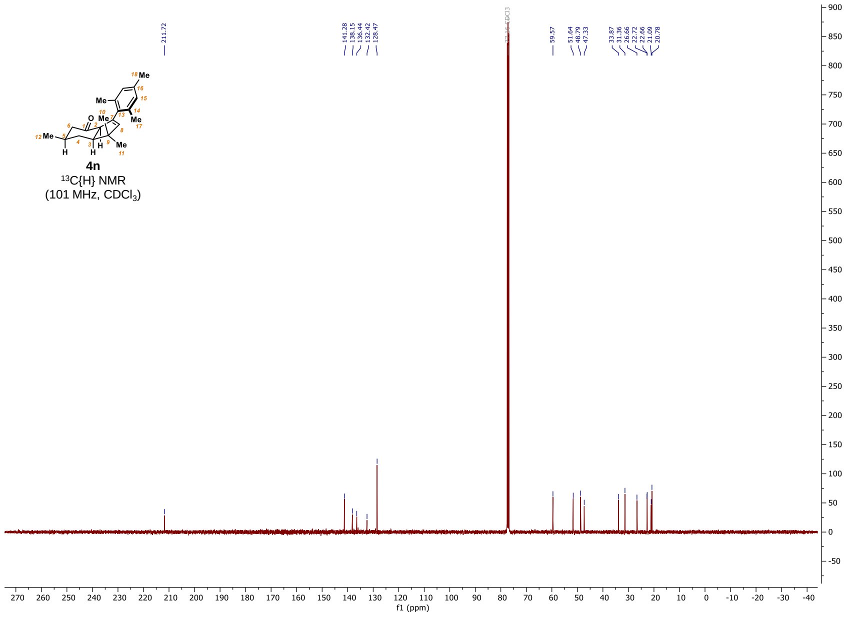
4n
13C{H} NMR
(101 MHz, CDCl3)
f1 (ppm)

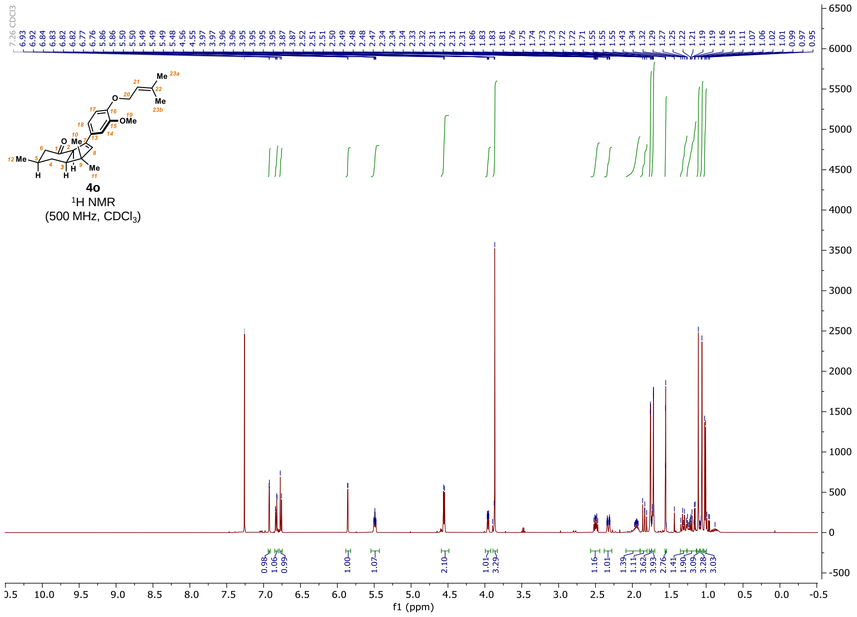
4o
^{1}H NMR
(500 MHz, $CDCl_3$)
f1 (ppm)

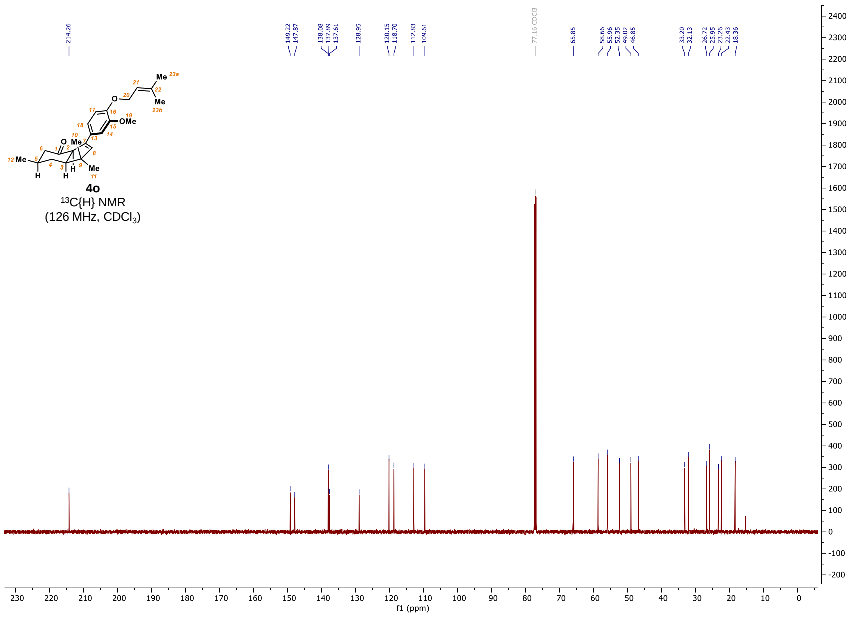
4o
^{13}C{H} NMR
(126 MHz, $CDCl_3$)
f1 (ppm)

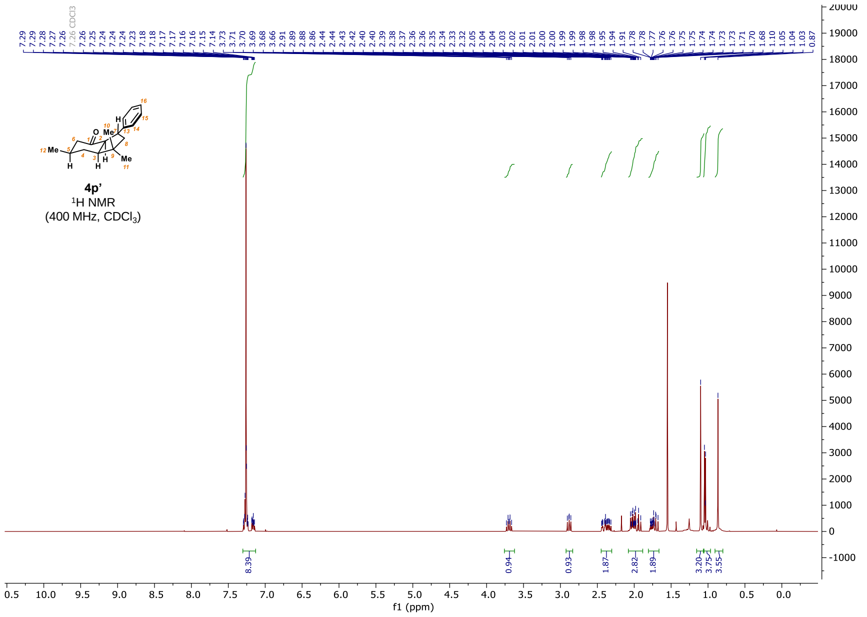
4p’
¹H NMR
(400 MHz, CDCl3)
f1 (ppm)

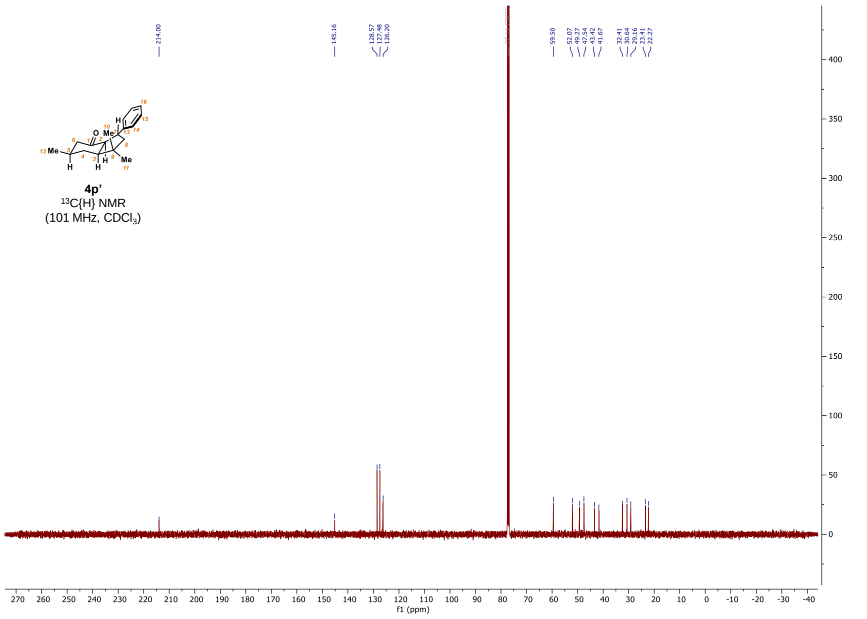
4p’
¹³C{H} NMR
(101 MHz, CDCl3)
214.00
145.16
128.57
127.48
126.20
59.50
52.07
49.27
47.54
43.42
41.67
32.41
30.64
29.16
23.41
22.27
f1 (ppm)

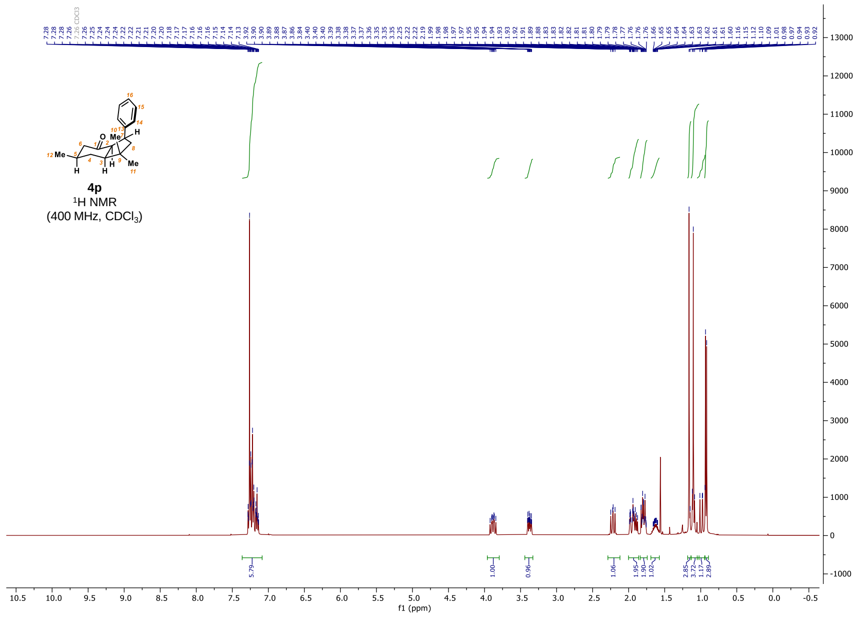
4p
1H NMR
(400 MHz, CDCl3)
f1 (ppm)

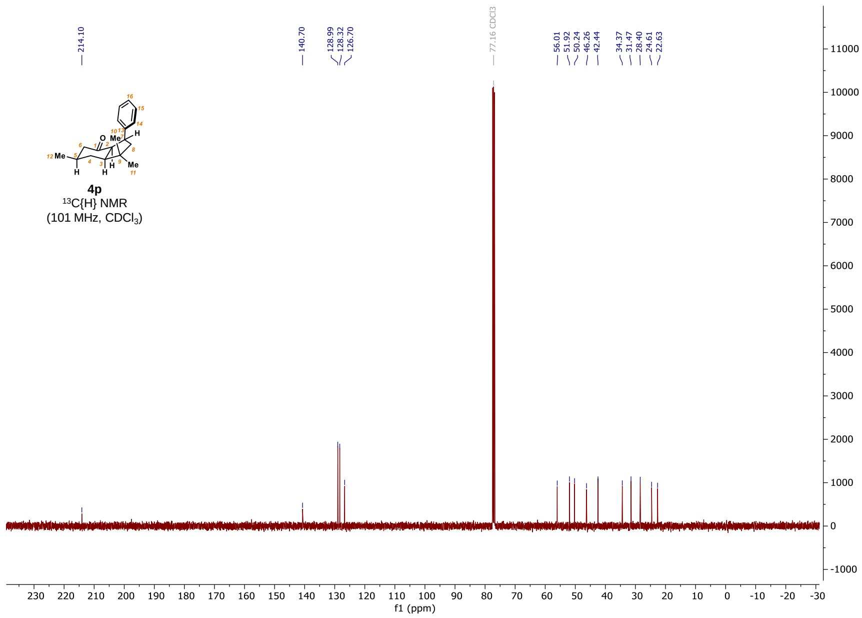
214.10
140.70
128.99
128.32
126.70
77.16 CDCl3
56.01
51.92
50.24
46.26
42.44
34.37
31.47
28.40
24.61
22.63
4p
13C{H} NMR
(101 MHz, CDCl3)
f1 (ppm)

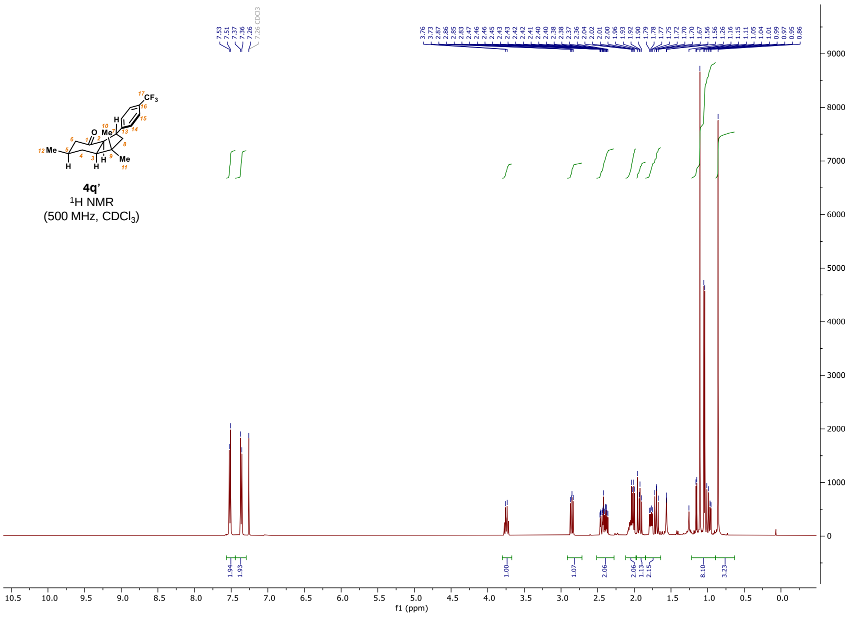
4q'
^{1}H NMR
(500 MHz, $CDCl_3$)
f1 (ppm)

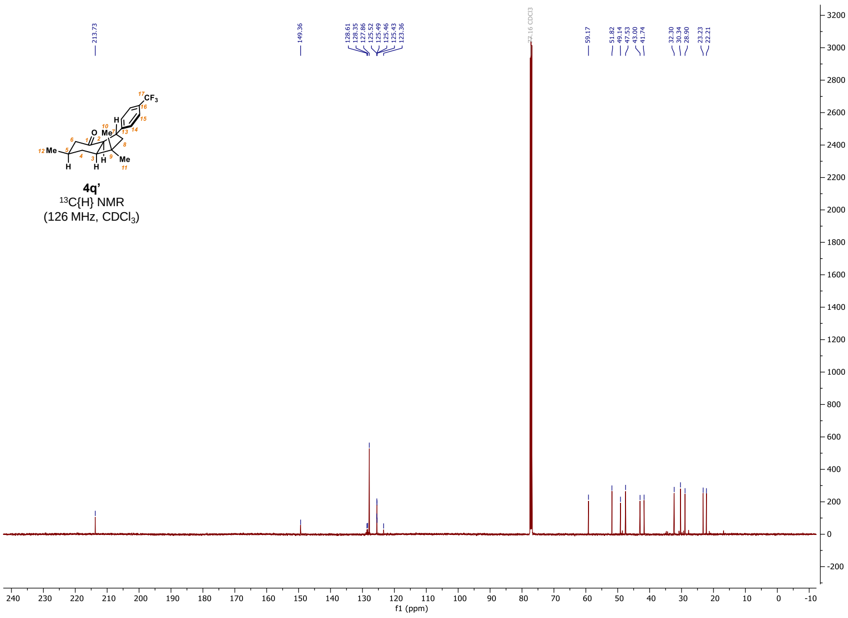
213.73
149.36
128.61
128.35
127.86
125.52
125.49
125.46
125.43
123.36
77.16 CDCl3
59.17
51.82
49.14
47.53
43.00
41.74
32.30
30.34
28.90
23.23
22.21
4q'
^{13}C{H} NMR
(126 MHz, $CDCl_3$)
f1 (ppm)

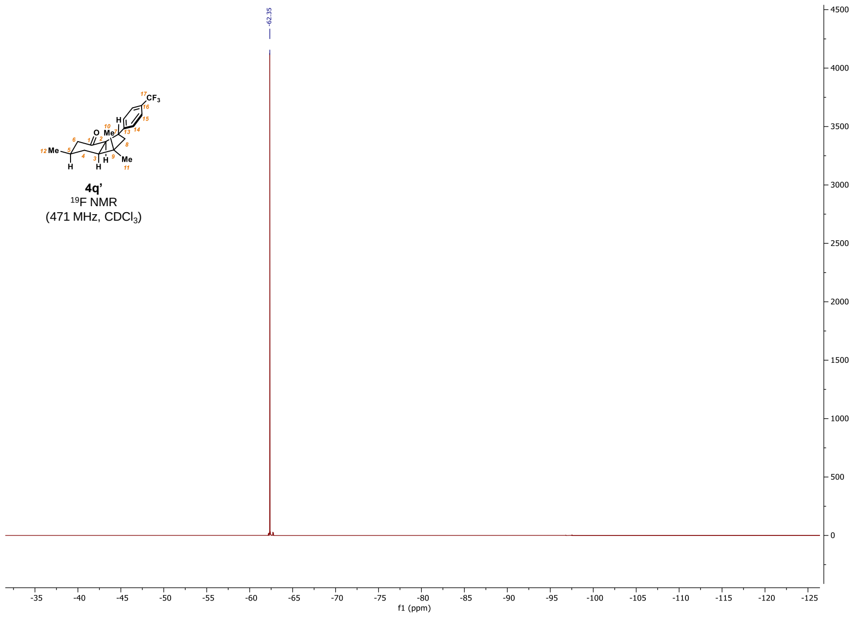
-62.35
4q'
19F NMR
(471 MHz, CDCl3)
f1 (ppm)

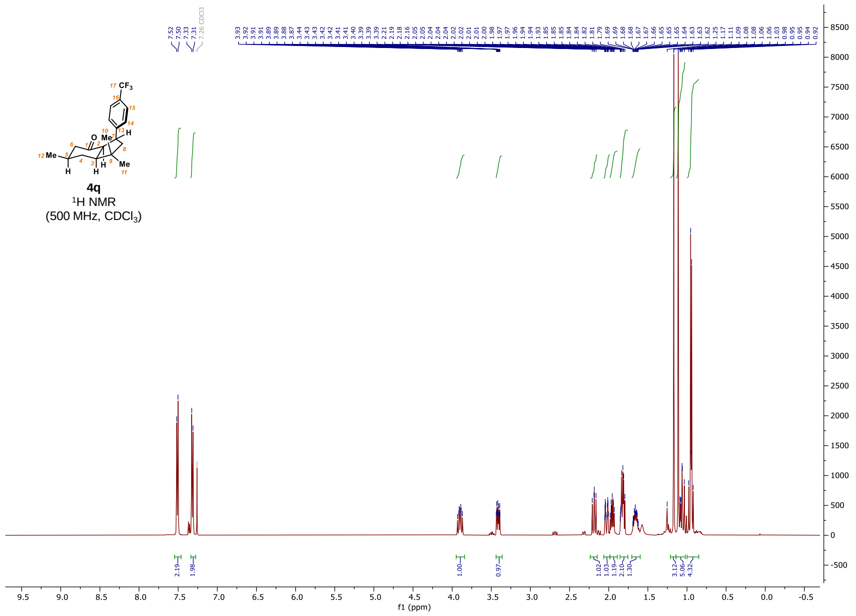
4q
1H NMR
(500 MHz, CDCl3)
f1 (ppm)

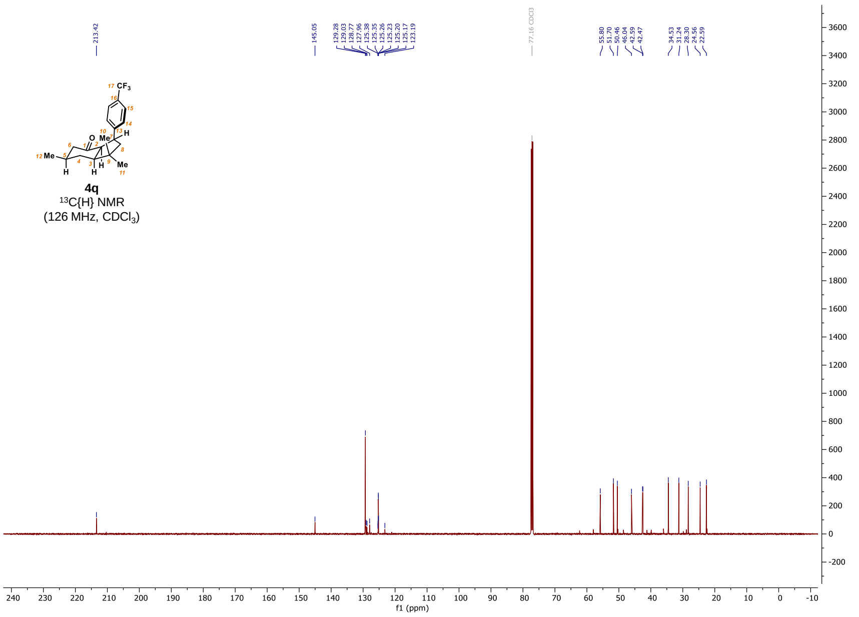
213.42
145.05
129.28
129.03
128.77
127.96
125.38
125.35
125.26
125.23
125.20
125.17
123.19
77.16 CDCl3
55.80
51.70
50.46
46.04
42.59
42.47
34.53
31.24
28.30
24.56
22.59
4q
$^{13}C\{H\}$ NMR
(126 MHz, $CDCl_3$)
f1 (ppm)

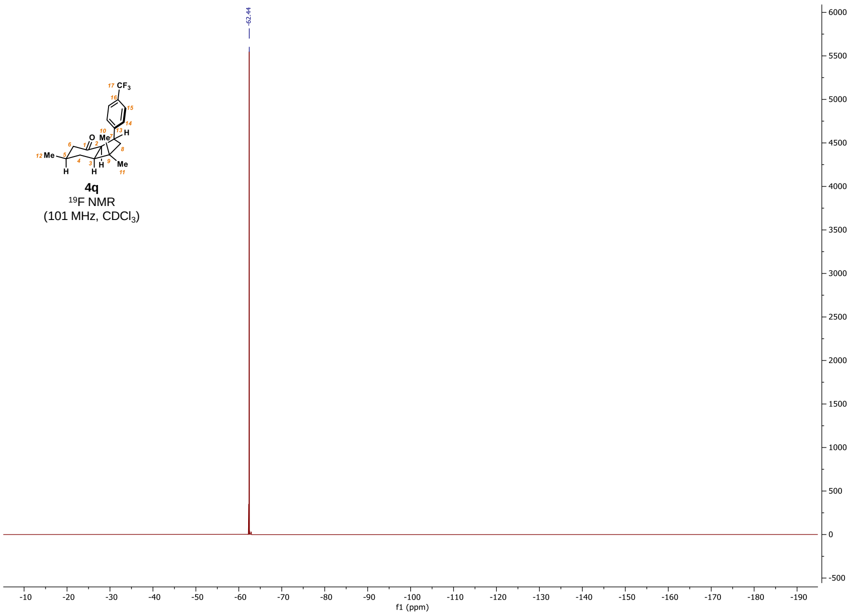
-62.44
4q
^{19}F NMR
(101 MHz, $CDCl_3$)
f1 (ppm)

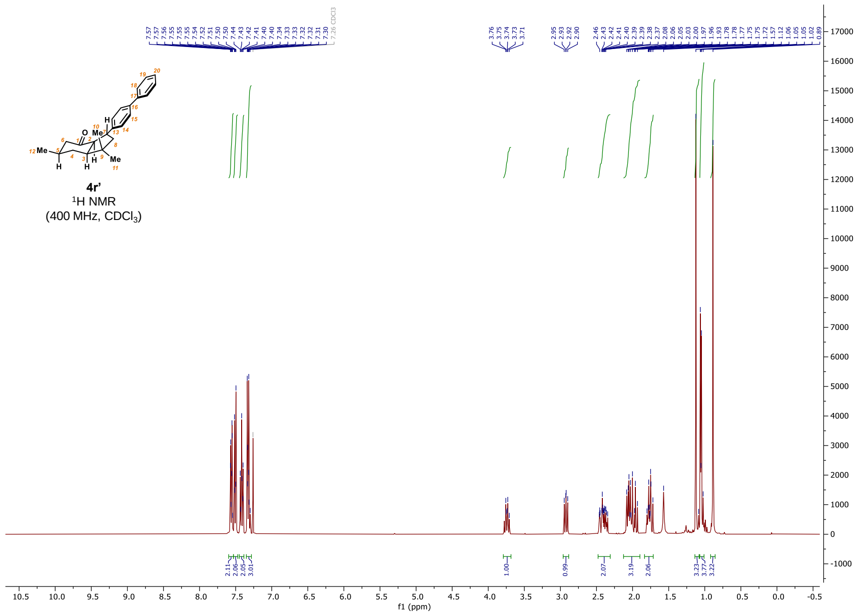
4r'
^{1}H NMR
(400 MHz, $CDCl_3$)
f1 (ppm)

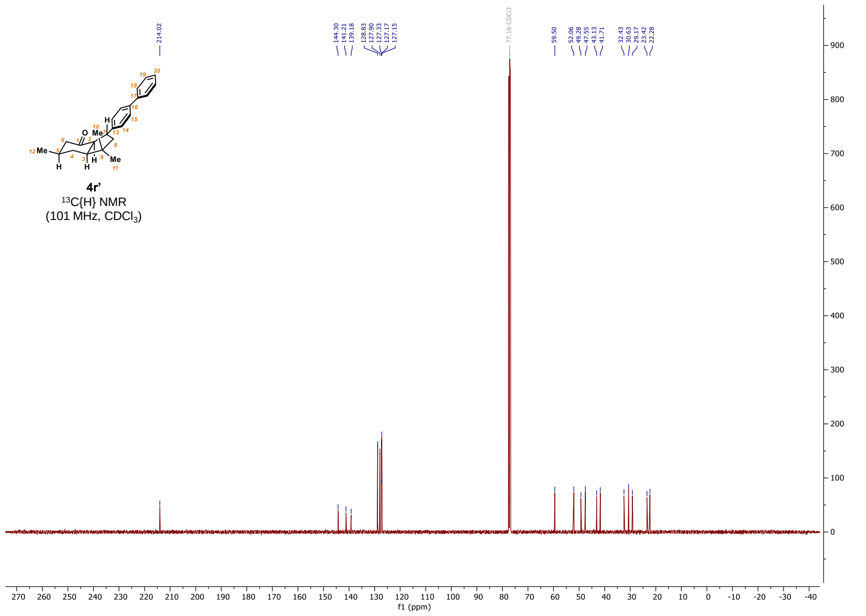
4r'
$^{13}C\{H\}$ NMR
(101 MHz, $CDCl_3$)
f1 (ppm)

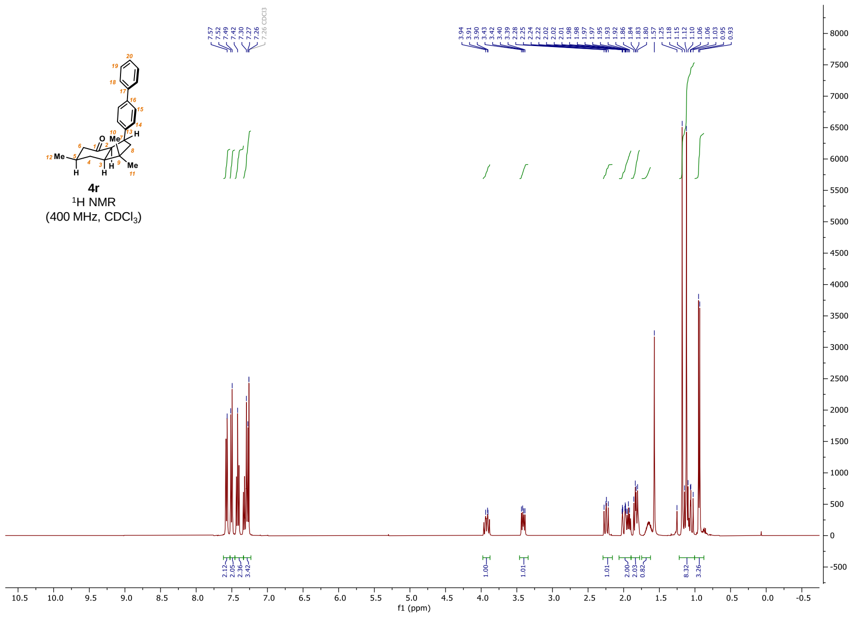
7.57
7.52
7.49
7.42
7.30
7.27
7.26
7.26 CDCl3
3.94
3.91
3.90
3.43
3.42
3.40
3.39
2.28
2.25
2.24
2.22
2.02
2.02
2.01
1.98
1.98
1.97
1.97
1.95
1.93
1.92
1.86
1.84
1.83
1.80
1.57
1.25
1.18
1.15
1.12
1.10
1.06
1.06
1.03
0.95
0.93
4r
1H NMR
(400 MHz, CDCl3)
2.12
2.05
2.36
3.42
1.00
1.01
1.01
2.00
2.03
0.82
8.32
3.26
f1 (ppm)

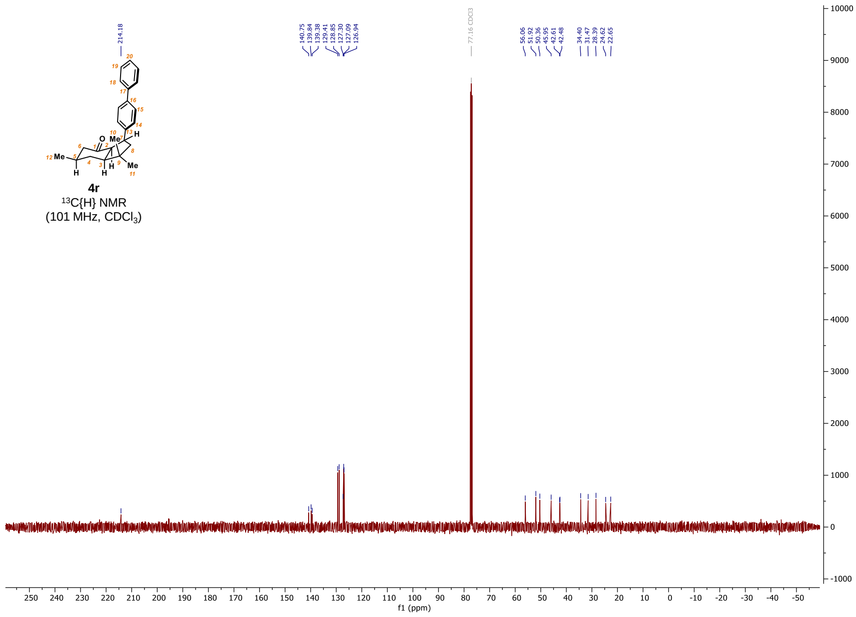
214.18
140.75
139.84
139.38
129.41
128.85
127.30
127.09
126.94
77.16 CDCl3
56.06
51.92
50.36
45.95
42.61
42.48
34.40
31.47
28.39
24.62
22.65
4r
13C{H} NMR
(101 MHz, CDCl3)
f1 (ppm)

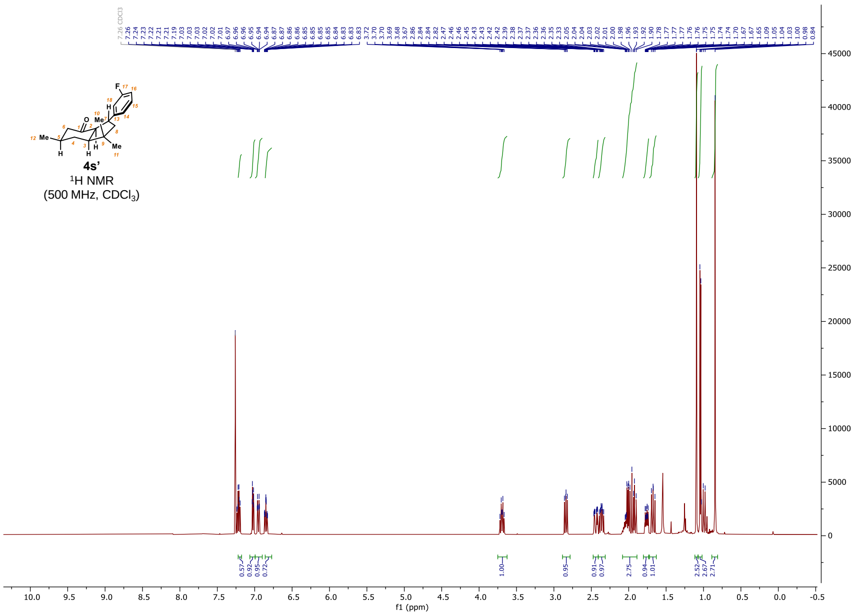
4s'
1H NMR
(500 MHz, CDCl3)
f1 (ppm)

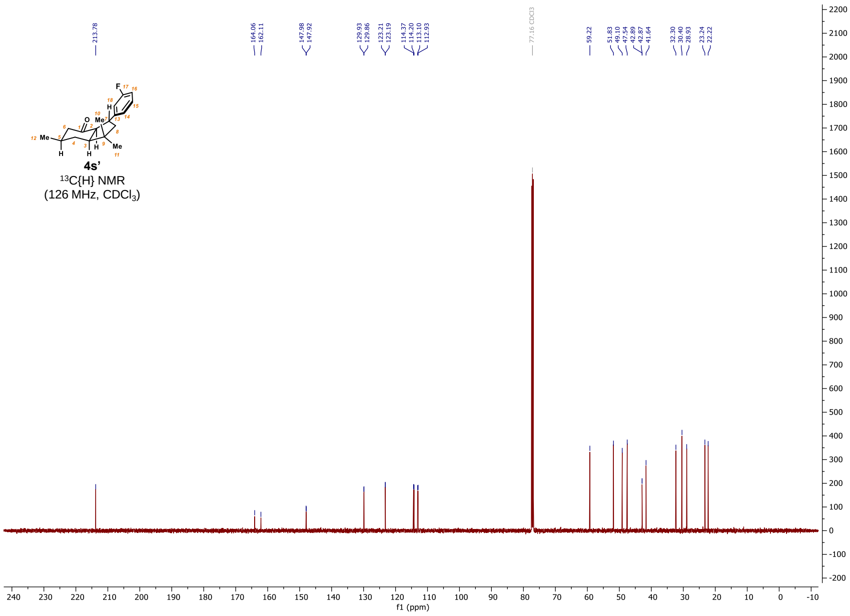
213.78
164.06
162.11
147.98
147.92
129.93
129.86
123.21
123.19
114.37
114.20
113.10
112.93
77.16 CDCl3
59.22
51.83
49.10
47.54
42.89
42.87
41.64
32.30
30.40
28.93
23.24
22.22
4s'
13C{H} NMR
(126 MHz, CDCl3)
f1 (ppm)

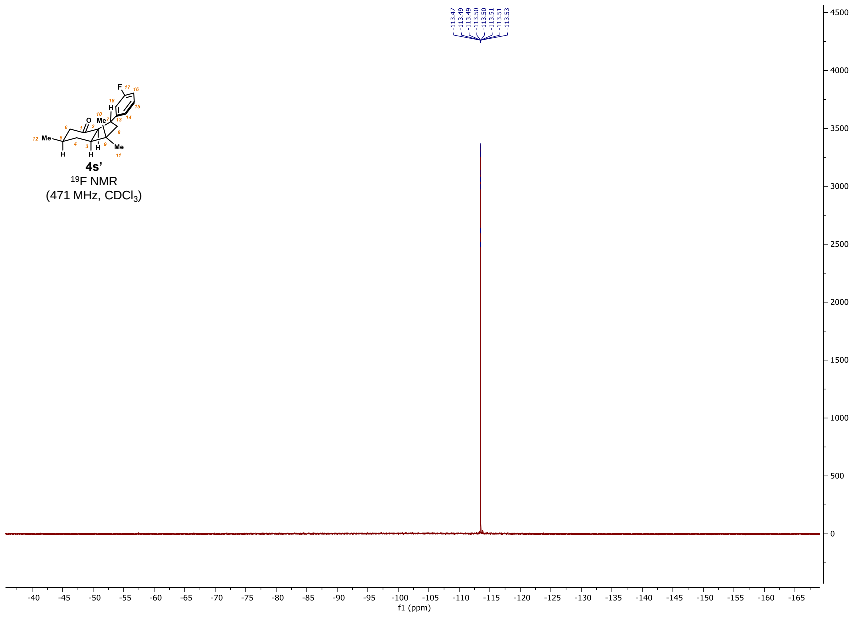
4s'
^{19}F NMR
(471 MHz, $CDCl_3$)
f1 (ppm)

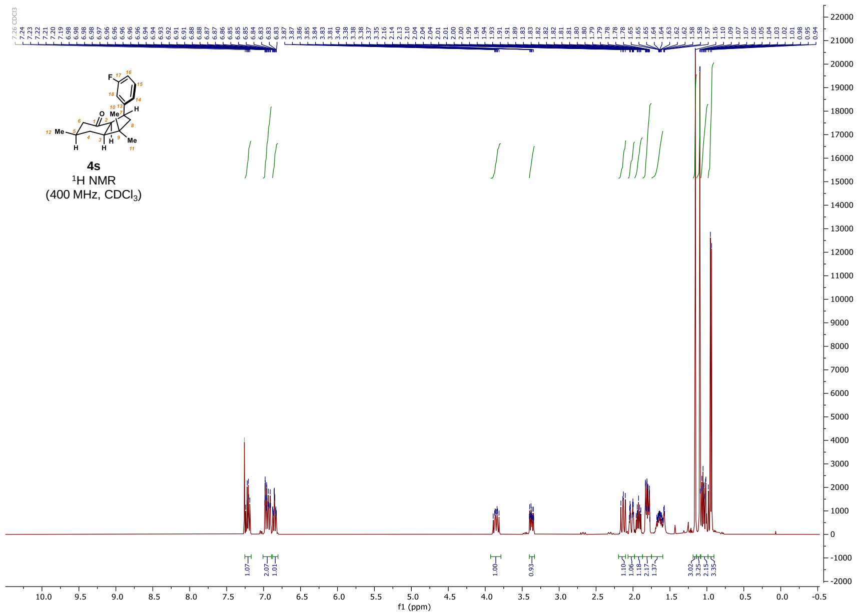
4s
^{1}H NMR
(400 MHz, $CDCl_3$)
f1 (ppm)

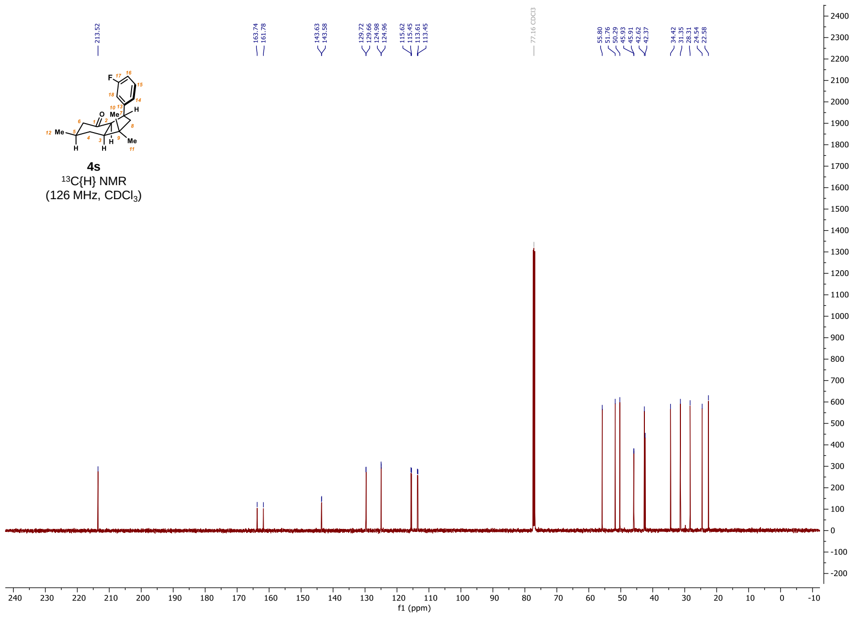
213.52
163.74
161.78
143.63
143.58
129.72
129.66
124.98
124.96
115.62
115.45
113.61
113.45
77.16 CDCl3
55.80
51.76
50.29
45.93
45.91
42.62
42.37
34.42
31.35
28.31
24.54
22.58
4s
$^{13}C\{H\}$ NMR
(126 MHz, $CDCl_3$)
f1 (ppm)

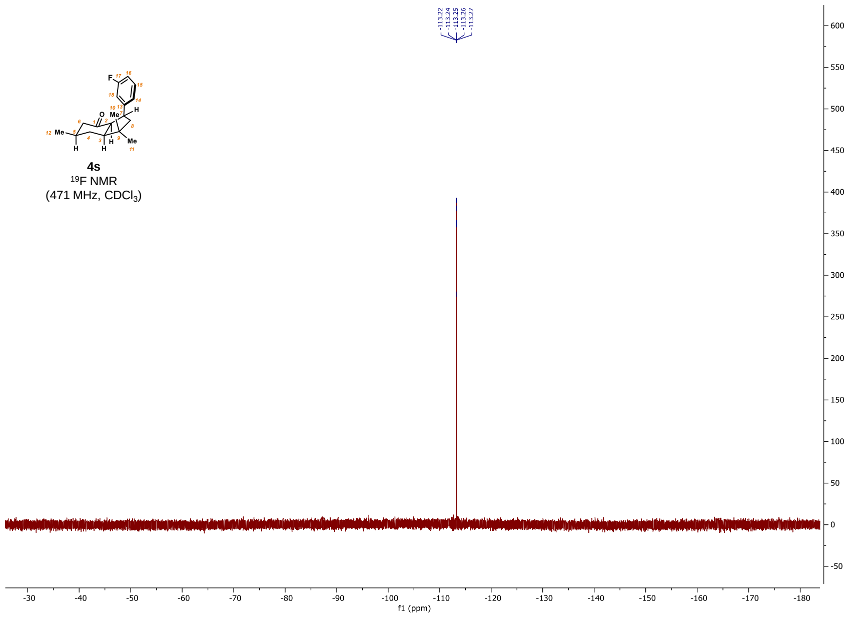
-113.22
-113.24
-113.25
-113.26
-113.27
4s
^{19}F NMR
(471 MHz, $CDCl_3$)
f1 (ppm)

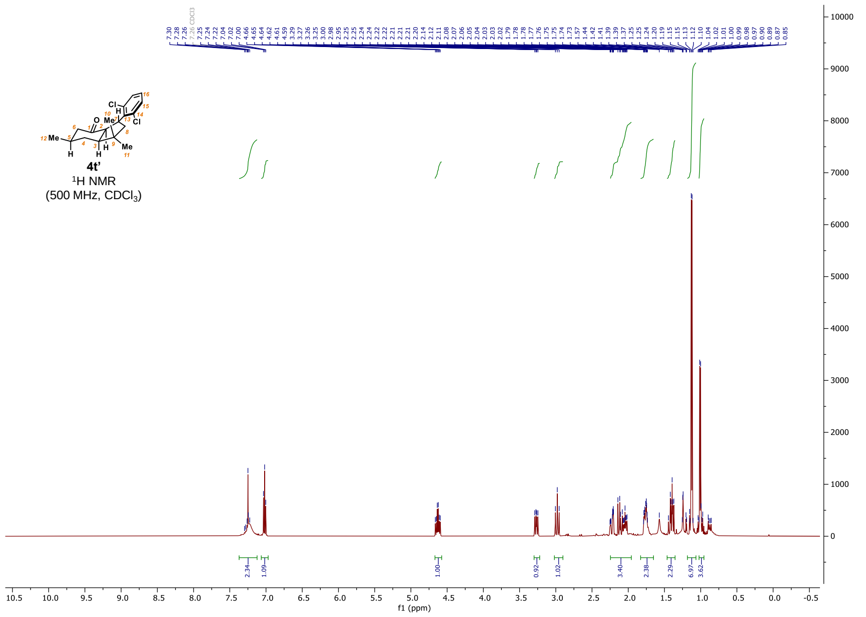
4t'
¹H NMR
(500 MHz, CDCl₃)
f1 (ppm)

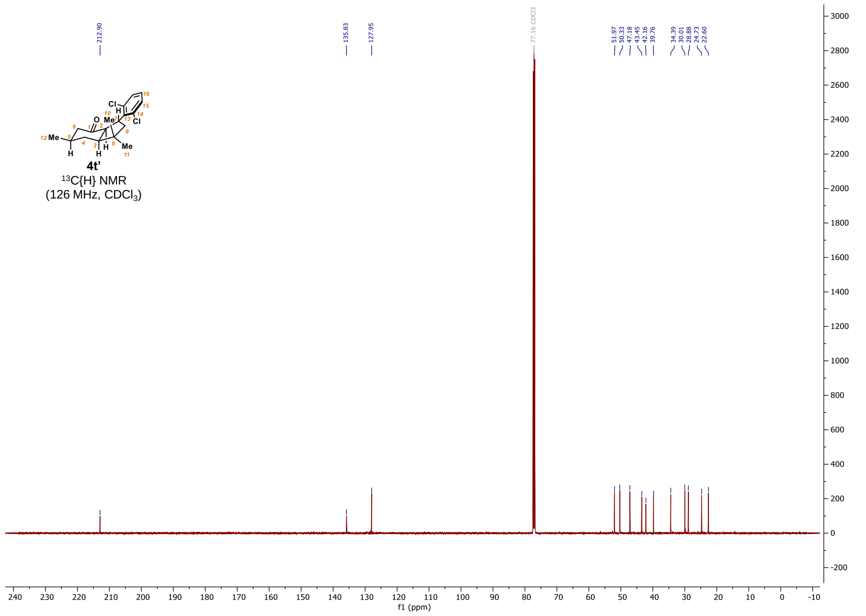
4t'
¹³C{H} NMR
(126 MHz, CDCl₃)
f1 (ppm)

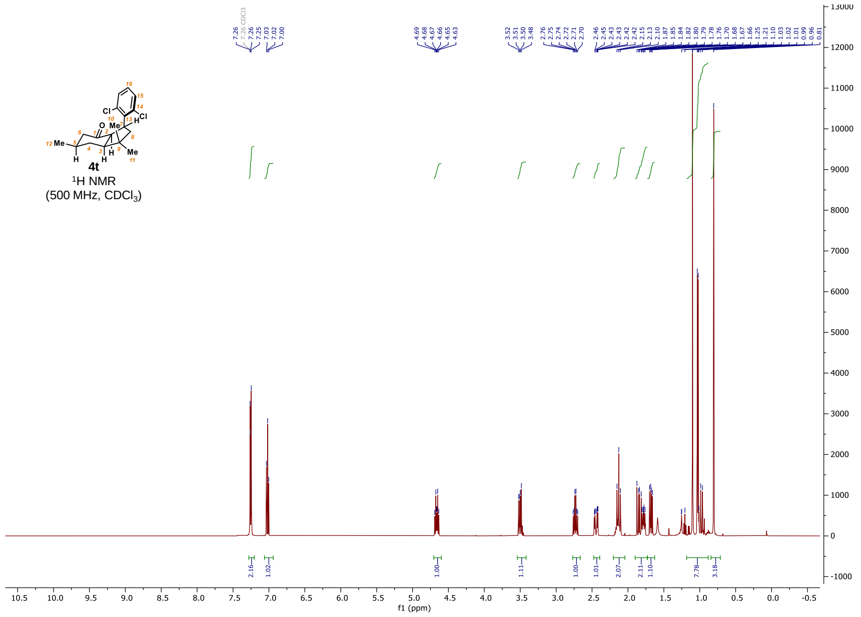

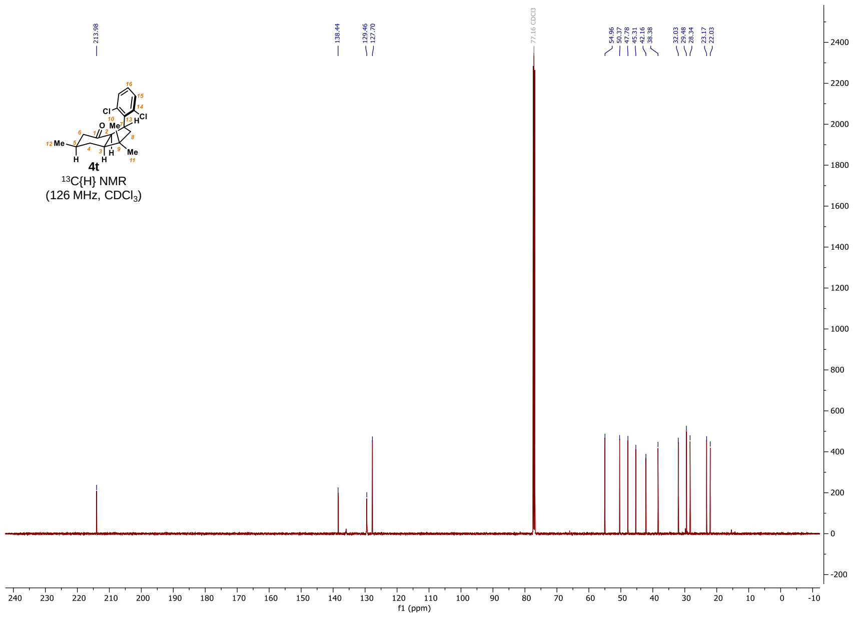

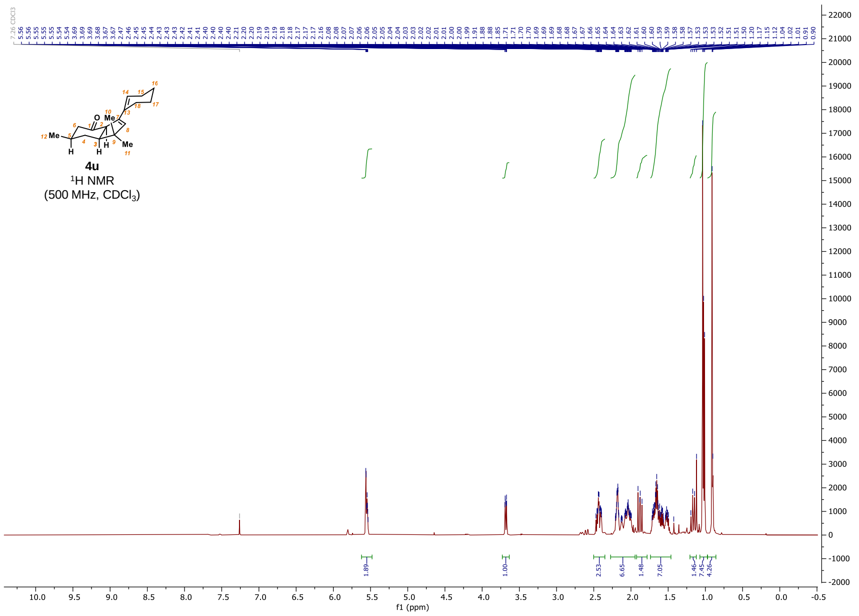
4u
^{1}H NMR
(500 MHz, $CDCl_3$)
f1 (ppm)

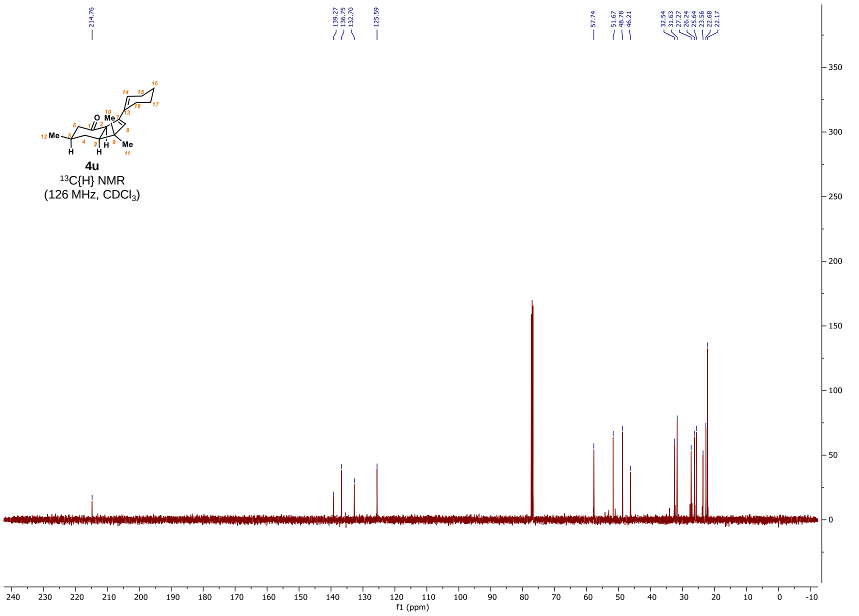
214.76
139.27
136.75
132.70
125.59
57.74
51.67
48.78
46.21
32.54
31.63
27.27
26.24
25.64
23.56
22.68
22.17
4u
^{13}C{H} NMR
(126 MHz, $CDCl_3$)
f1 (ppm)

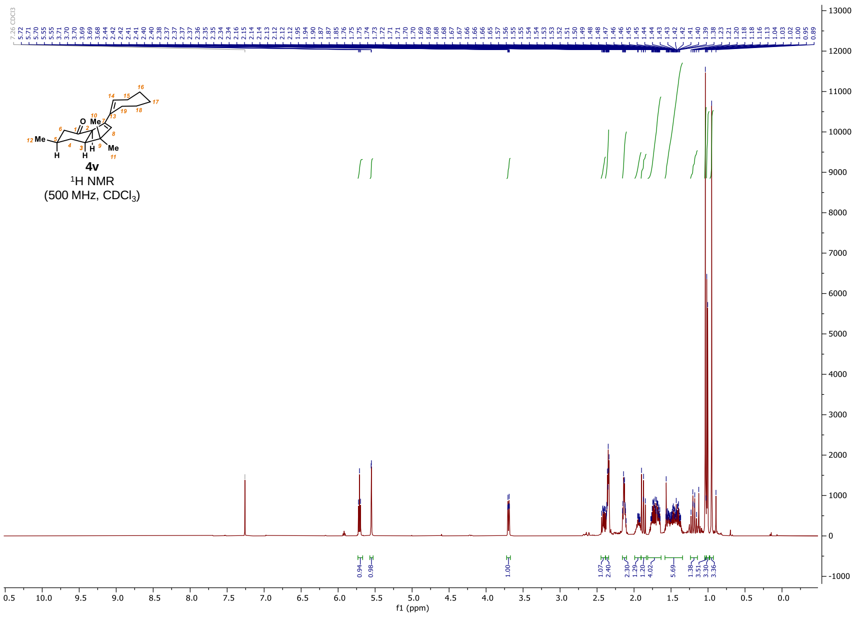
4v
1H NMR
(500 MHz, CDCl3)
f1 (ppm)

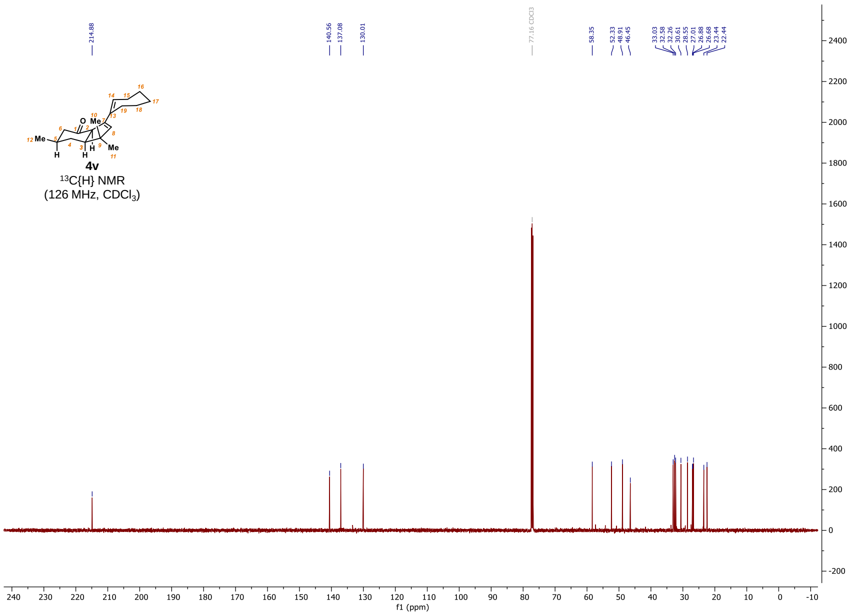
4v
13C{H} NMR
(126 MHz, CDCl3)
214.88
140.56
137.08
130.01
77.16 CDCl3
58.35
52.33
48.91
46.45
33.03
32.58
32.26
30.61
28.55
27.01
26.68
23.44
22.44
f1 (ppm)

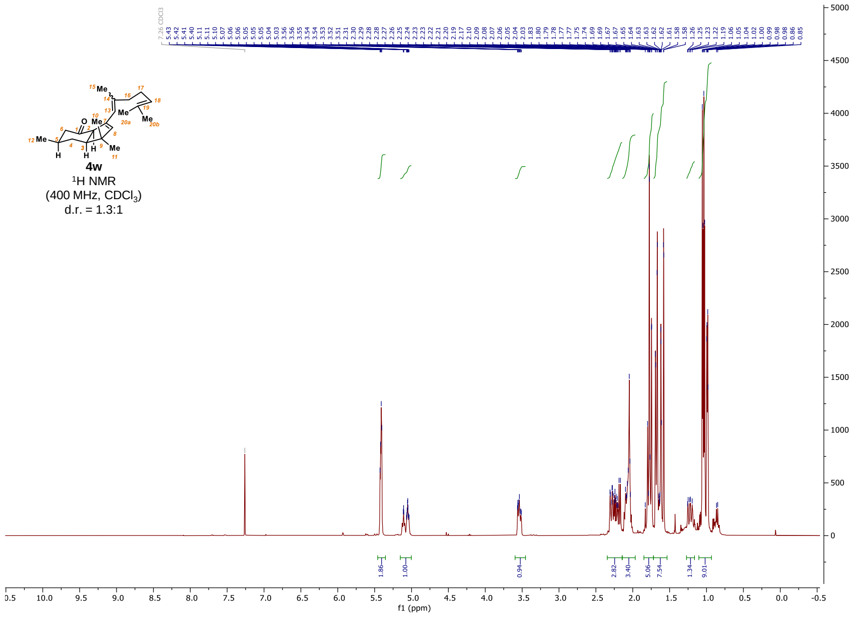
4w
¹H NMR
(400 MHz, CDCl3)
d.r. = 1.3:1
f1 (ppm)

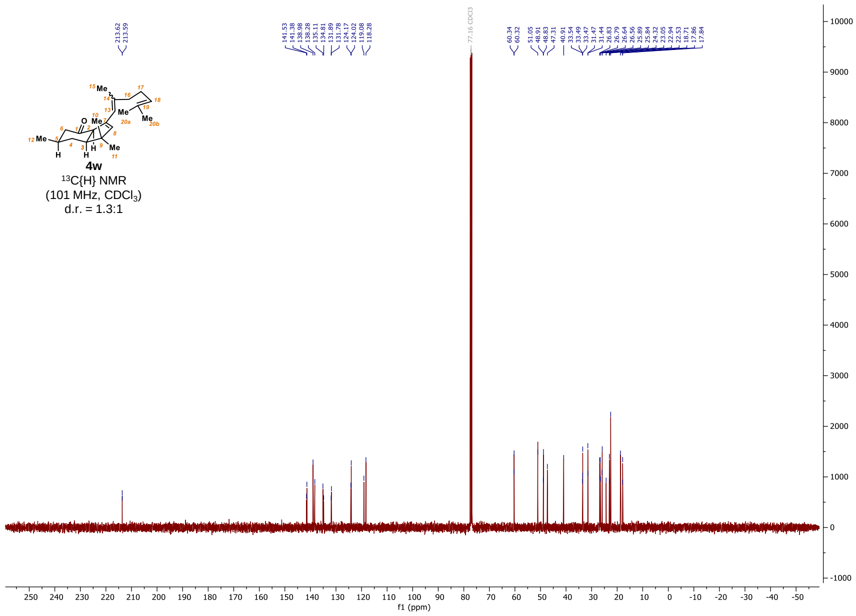
4w
¹³C{H} NMR
(101 MHz, CDCl3)
d.r. = 1.3:1
f1 (ppm)

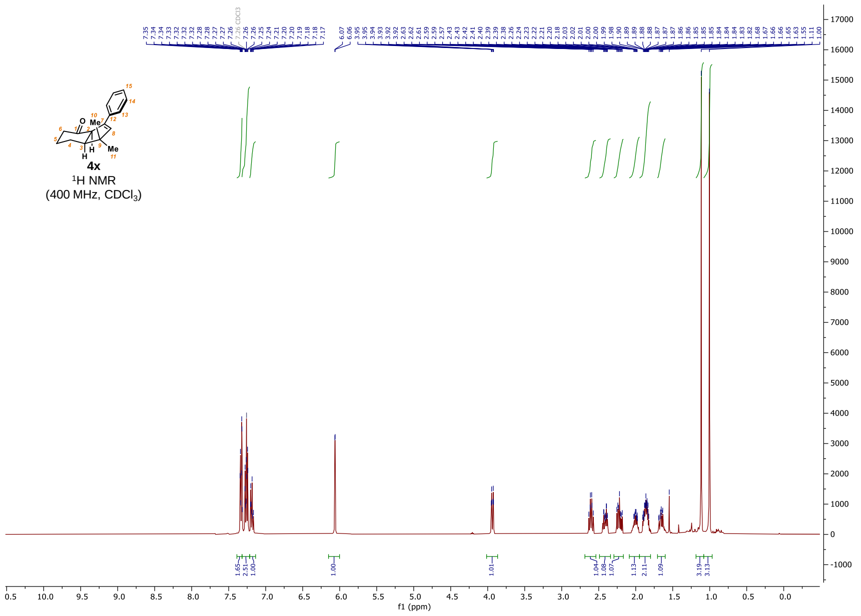
4x
^{1}H NMR
(400 MHz, CDCl$_3$)
f1 (ppm)

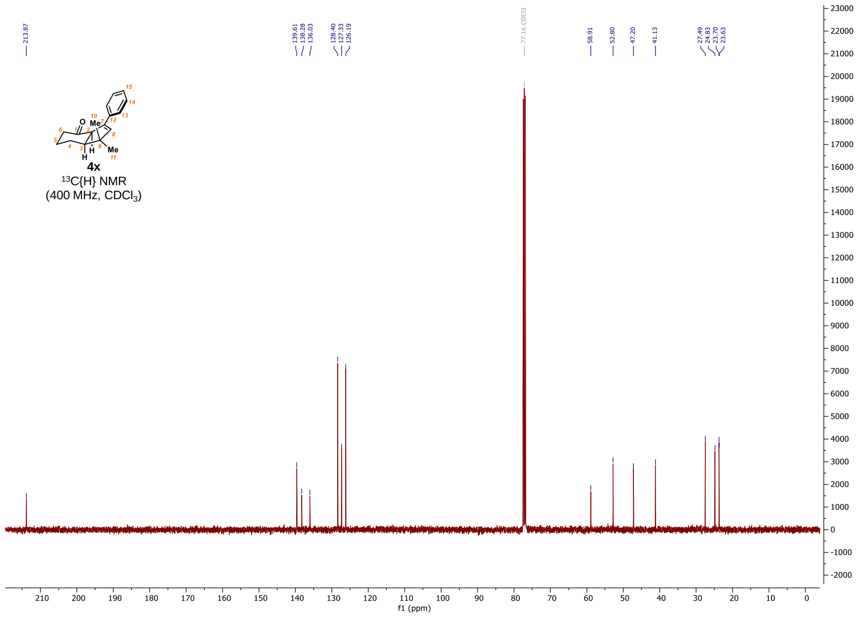
4x
^{13}C{H} NMR
(400 MHz, CDCl$_3$)
213.87
139.61
138.28
136.03
128.40
127.33
126.19
77.16 CDCl3
58.91
52.80
47.20
41.13
27.49
24.83
23.70
23.63
f1 (ppm)

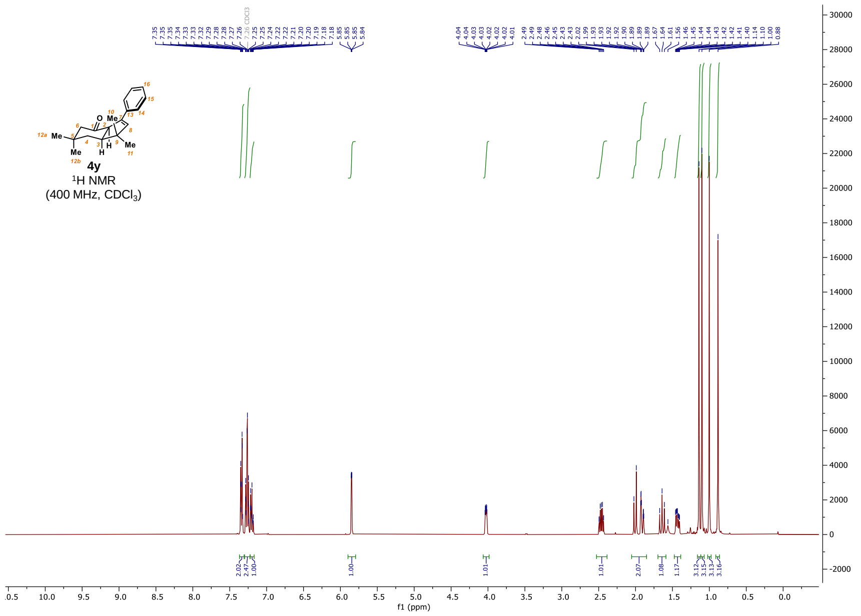
4y
^{1}H NMR
(400 MHz, CDCl$_3$)
f1 (ppm)

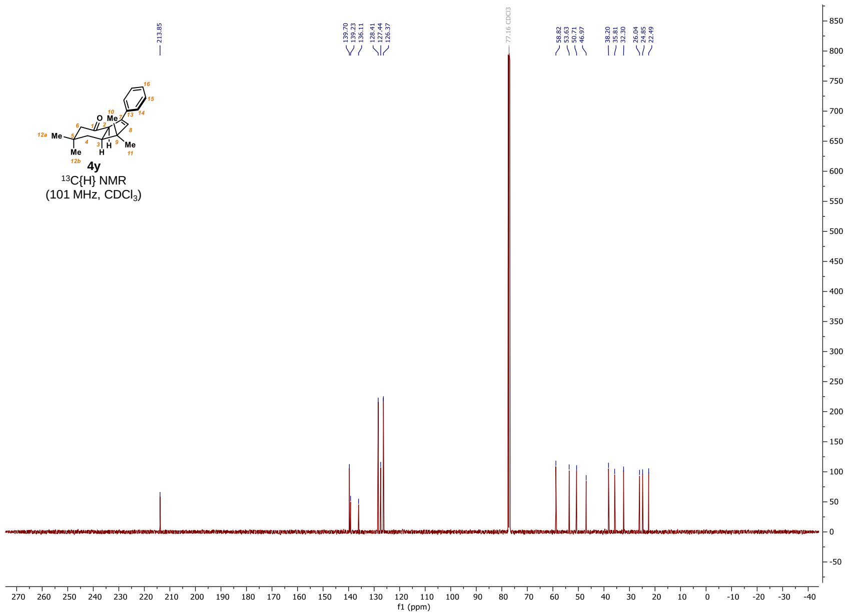
4y
^{13}C{H} NMR
(101 MHz, CDCl$_3$)
213.85
139.70
139.23
136.11
128.41
127.44
126.37
77.16 CDCl3
58.82
53.63
50.71
46.97
38.20
35.81
32.30
26.04
24.85
22.49
f1 (ppm)

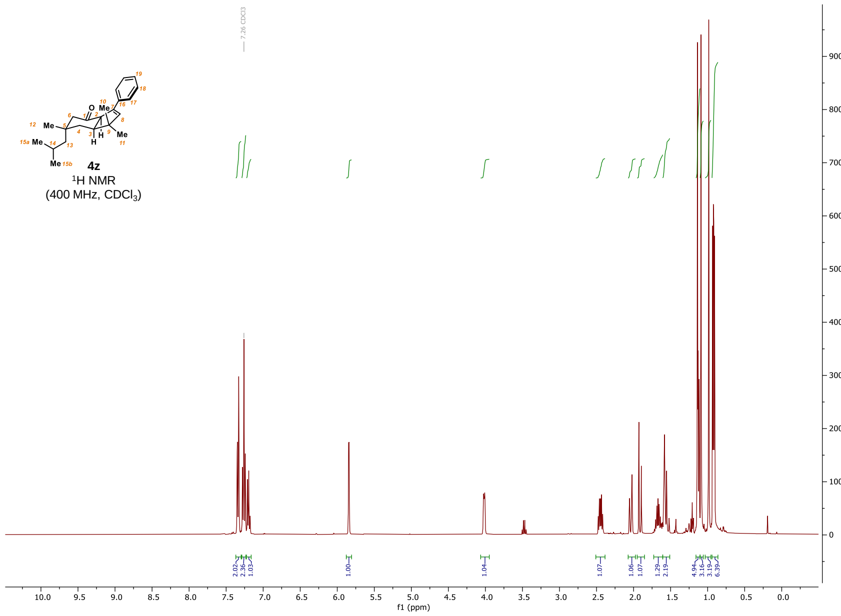

7.26 CDCl3
4z
¹H NMR
(400 MHz, CDCl3)
f1 (ppm)

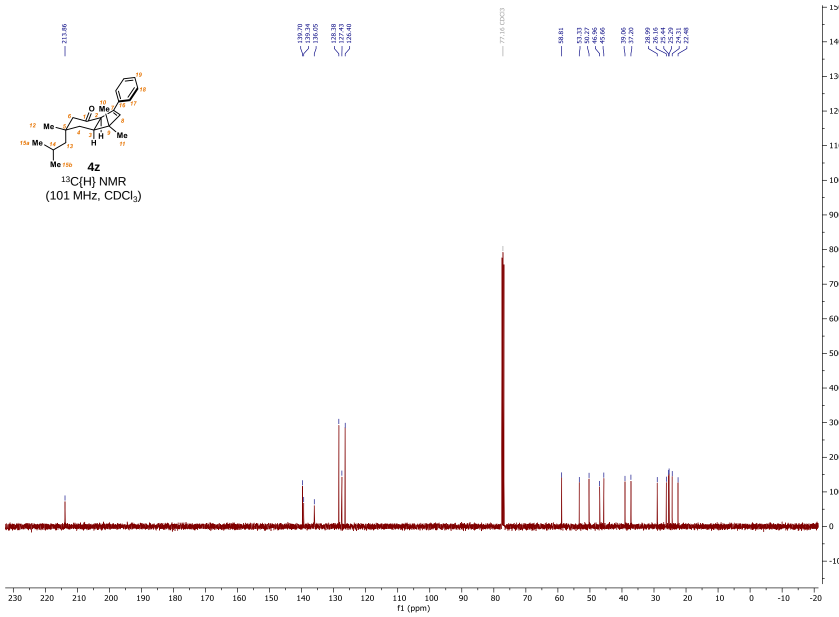

213.86
139.70
139.34
136.05
128.38
127.43
126.40
77.16 CDCl3
58.81
53.33
50.27
46.96
45.66
39.06
37.20
28.99
26.16
25.44
25.29
24.31
22.48
4z
¹³C{H} NMR
(101 MHz, CDCl3)
f1 (ppm)

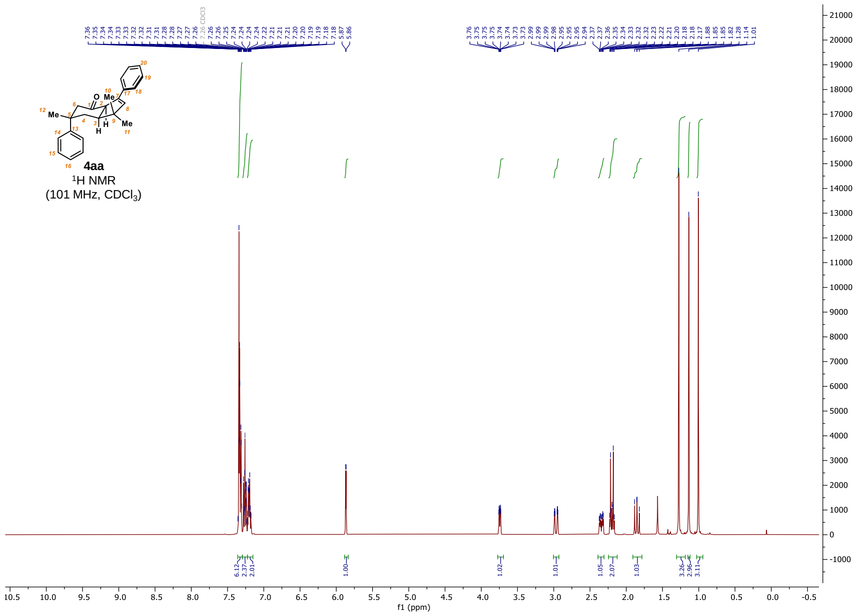
4aa
1H NMR
(101 MHz, CDCl3)
f1 (ppm)

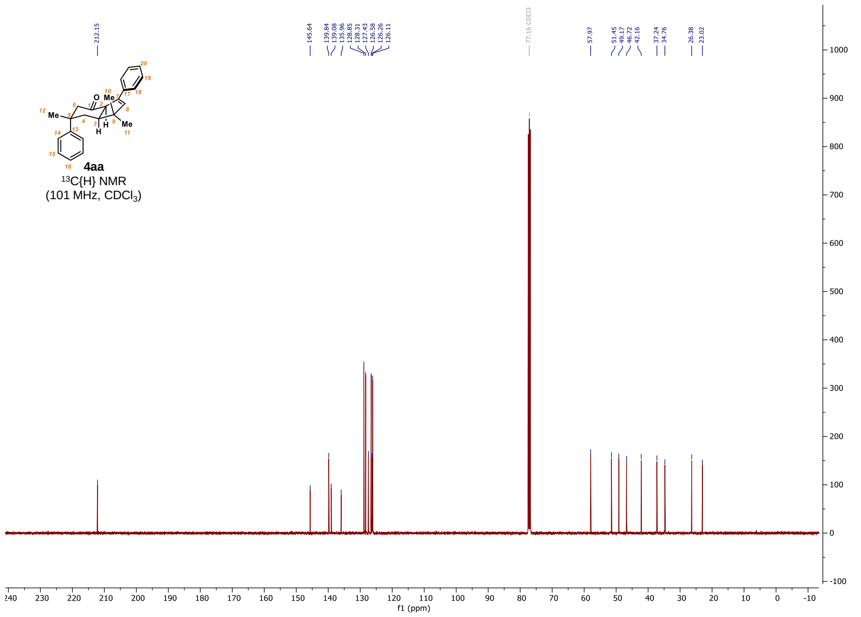
4aa
13C{H} NMR
(101 MHz, CDCl3)
f1 (ppm)

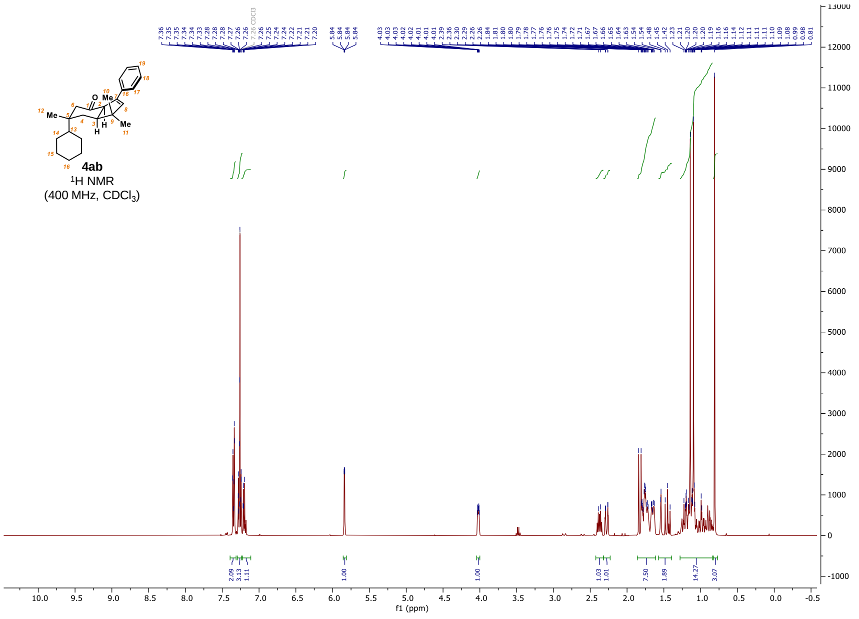
4ab
^{1}H NMR
(400 MHz, $CDCl_3$)
f1 (ppm)

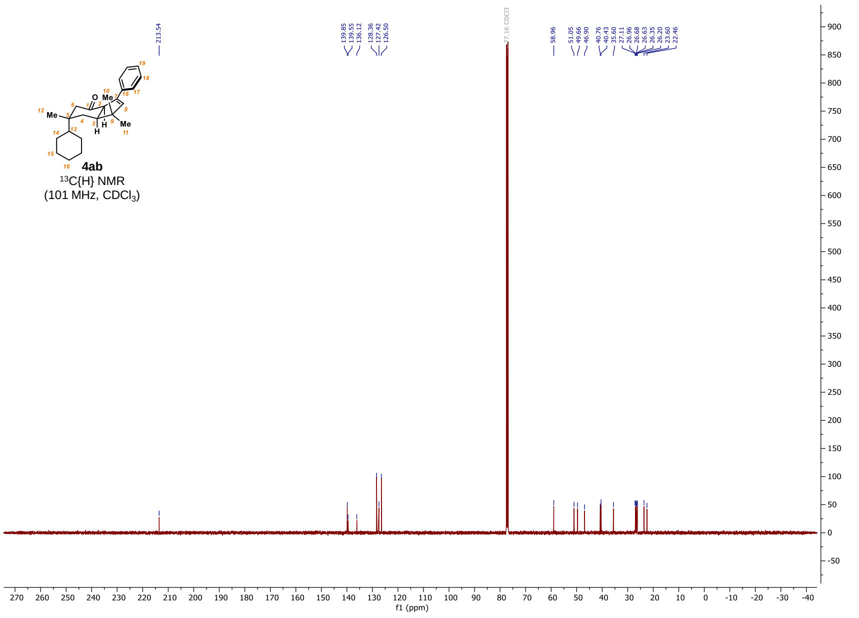
4ab
^{13}C{H} NMR
(101 MHz, $CDCl_3$)
f1 (ppm)

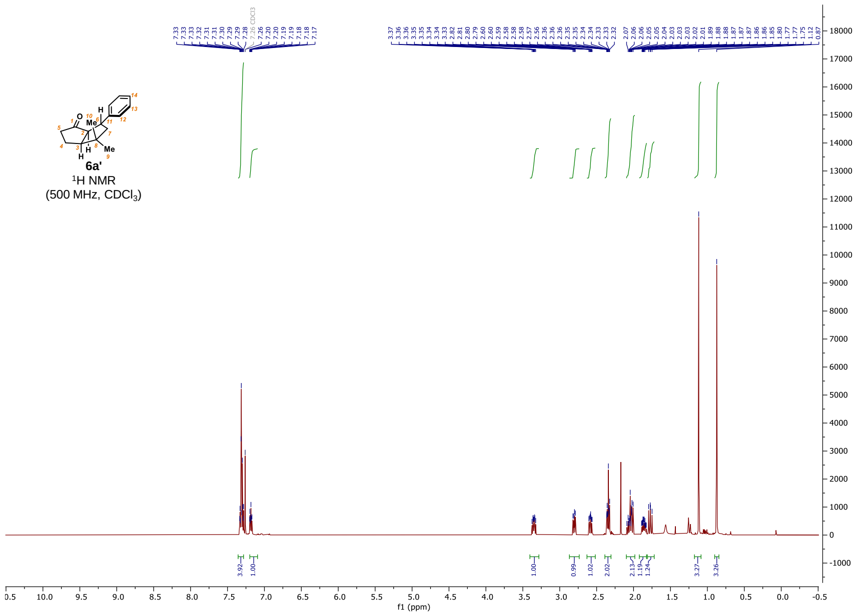
6a'
^{1}H NMR
(500 MHz, $CDCl_3$)
f1 (ppm)

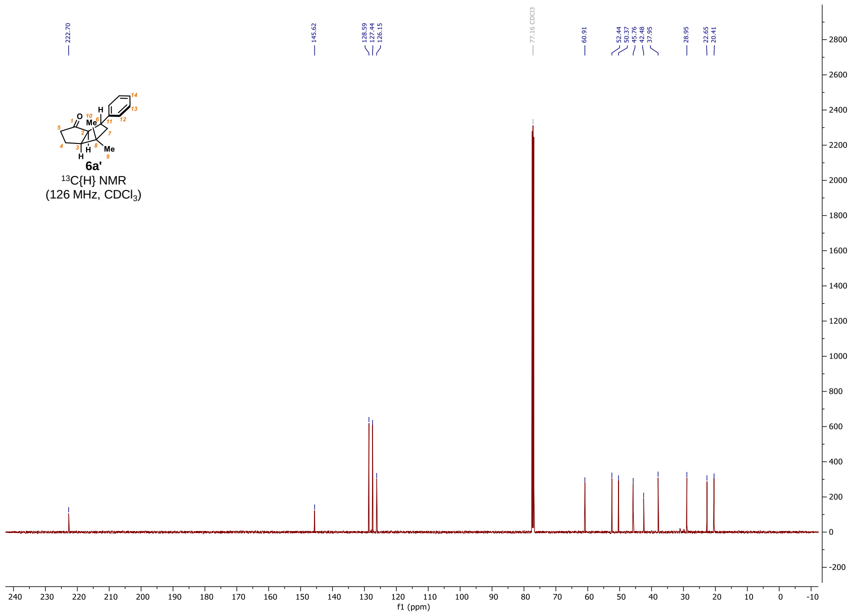
6a'
^{13}C{H} NMR
(126 MHz, $CDCl_3$)
222.70
145.62
128.59
127.44
126.15
60.91
52.44
50.37
45.76
42.48
37.95
28.95
22.65
20.41
f1 (ppm)

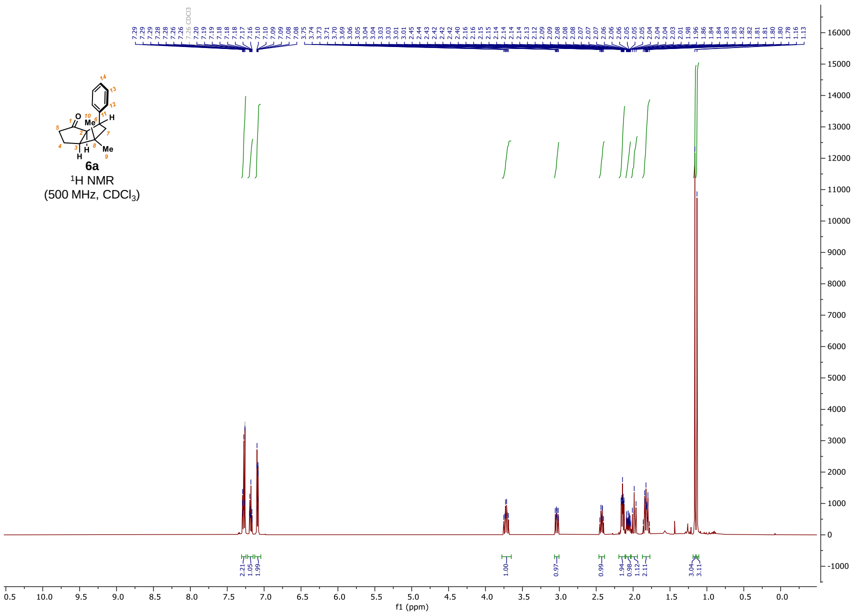
6a
¹H NMR
(500 MHz, CDCl3)
f1 (ppm)

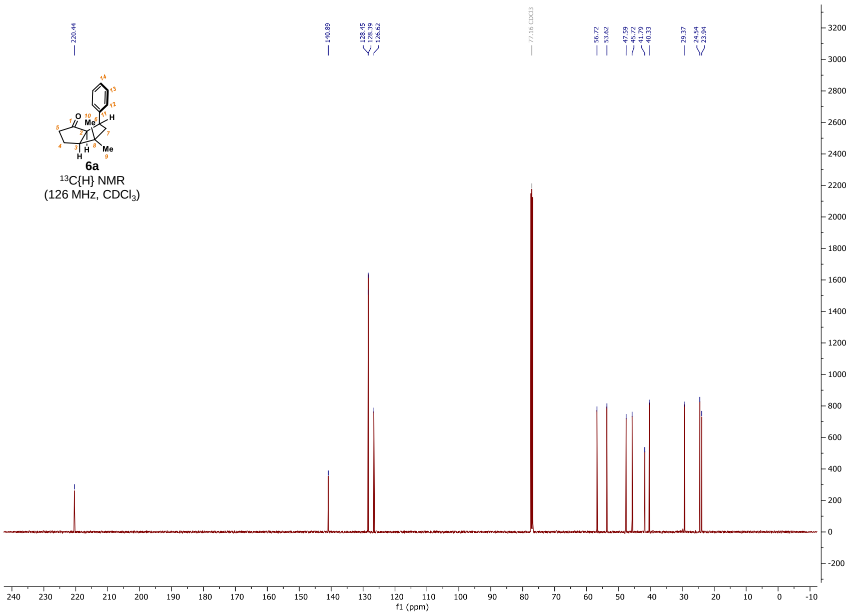
220.44
140.89
128.45
128.39
126.62
77.16 CDCl3
56.72
53.62
47.59
45.72
41.79
40.33
29.37
24.54
23.94
6a
¹³C{H} NMR
(126 MHz, CDCl3)
f1 (ppm)

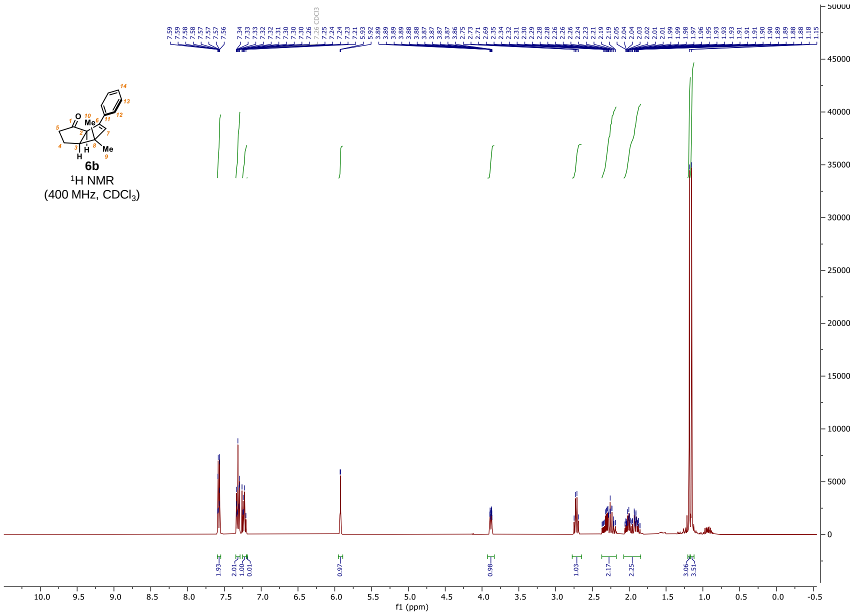
6b
^{1}H NMR
(400 MHz, CDCl$_3$)
f1 (ppm)

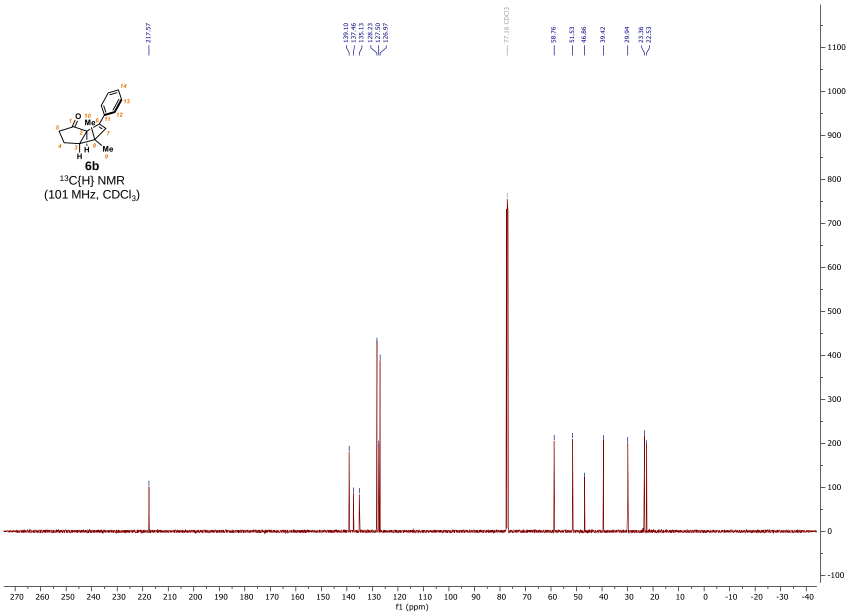
217.57
139.10
137.46
135.13
128.23
127.50
126.97
77.16 CDCl3
58.76
51.53
46.86
39.42
29.94
23.36
22.53
6b
^{13}C{H} NMR
(101 MHz, CDCl$_3$)
f1 (ppm)

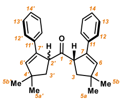

8
^{1}H NMR
(400 MHz, $CDCl_3$)
d.r. = 1.6:1

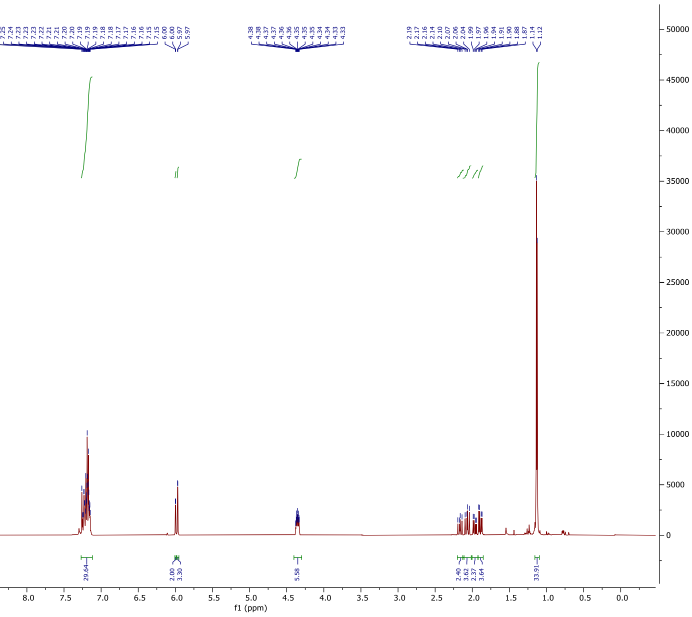

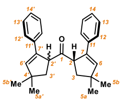

8
$^{13}C\{H\}$ NMR
(126 MHz, $CDCl_3$)
d.r. = 1.6:1

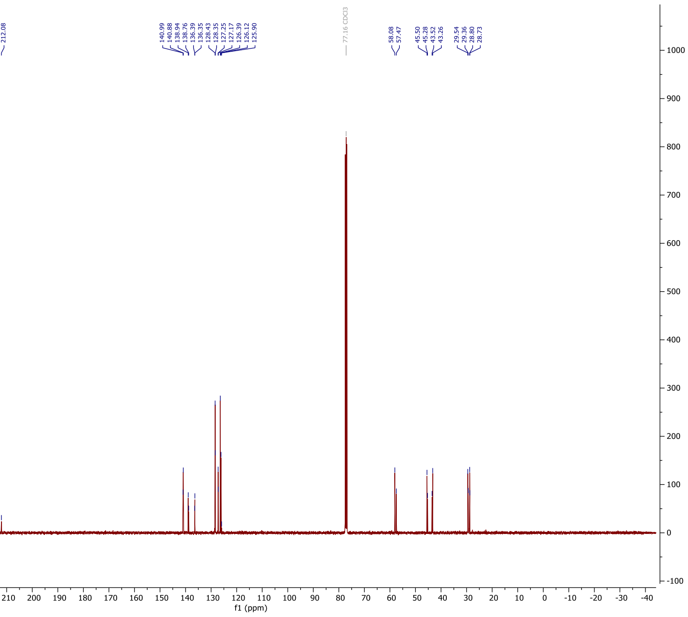

Supplement: Supplementary file 1 — ja4c03073_si_001.pdf [file ja4c03073_si_001.pdf]
